# Population in floodplains or close to sea level increased in US but declined in some counties—especially among Black residents

**DOI:** 10.1088/1748-9326/acadf5

**Published:** 2023-02-16

**Authors:** James G Titus

**Affiliations:** Climate Change Division (6207-A), US Environmental Protection Agency, Washington, DC 20460, United States of America

**Keywords:** sea level rise, climate change indicator, climate refugee, redlining, social vulnerability, hazard zone

## Abstract

Previous studies estimating US population vulnerable to climate change have assumed that population is uniformly distributed within the geographical unit of analysis, typically a census block. That assumption overstates vulnerable population in places where people have avoided the most hazardous areas. After using independent samples of housing location and building footprints to validate a revised approach, this letter estimates the US population close to sea level and in floodplains based on the assumption that population in a hazard zone is proportional to the fraction of buildings in the hazard zone, for the period 1990–2020. The building-based assumption reduces population estimates about 30%. Over the 30-year period, the US population below 1 m increased 14%–18% from 1.85 (±0.19) million in 1990 to 2.2 (±0.25) million; population below 3 m increased 31%. Sea level rise accounts for 75% of the increase in population below 1 m, but only 14% of the increase below 3 m. Nevertheless, in 21 counties, net emigration from land below 1 m was greater than 1% of county population. Although this emigration was particularly great in the 2000s after Hurricane Katrina, it totaled 170 000 for the entire 30-year period. Similarly, the US population of inland 100-year floodplains increased 7%, but several hundred counties saw a steady emigration throughout the 30-year period which totaled about 240 000. Black residents accounted for 19% of the population below 1 m but only 12% of the nation’s population in 2020, meaning that Black residents were 63% more likely to live below 1 m than the general population nationwide. This disproportionality is more attributable to high Black populations in the most vulnerable localities (especially New Orleans) than to historic segregation. Black people also are almost five times as likely to have emigrated from land below 1 m than the general population since 1990.

## 1. Introduction

For the last few decades researchers and government agencies have warned that rising sea level and increasing coastal development are on a collision course likely to harm coastal environments and threaten people and property [1–3]. Increasingly intense rainstorms combined with development in poorly mapped floodplains will create similar problems inland [4]. Adaptation programs to prepare for these consequences are becoming commonplace [5, 6].

The suggested responses generally fall into three categories: structural solutions to limit threats to life and property (‘protection’), curtailing development and removing the human footprint from the most vulnerable areas (‘retreat’), and hybrid approaches in which people learn to live with the increasingly wet environments (‘accommodation’) [7]. Land use planners and researchers generally expect shore protection in densely developed areas, and retreat in areas where either development is limited, protection costs are especially high, or socially vulnerable inhabitants lack the resources needed for structural solutions—at least in the long run [8]. Meanwhile, the decisions people make about where to build their lives depend on more than how they perceive various risks. Familial ties, land already owned, the attractiveness of the water, or engagement in water-dependent activities can discourage people from leaving a hazardous home [9]. Nevertheless, increasing awareness, actual experience, and government policies are likely to eventually lead people to move out of the most vulnerable areas [10]. Is that happening yet?

This letter estimates changes in the number of people living close to sea level and in floodplains in the United States for the period 1990–2020, by overlaying elevation and census block data. We differentiate the effects of sea level rise from natural changes in population due to births, deaths, and migration. Although the block data do not include most measures of social vulnerability, they do report population by race, allowing us to examine changes in the numbers of Black and other African American residents, and Latino or Hispanic (*hereinafter* ‘Hispanic’) residents in these hazardous areas.

Previous studies assumed that residents are uniformly distributed within census blocks. We use available building footprint data to consider an alternative assumption: the fraction of a block’s population in a hazard zone equals the fraction of buildings in that zone. A preliminary analysis compared results of both assumptions to counts of housing units derived from remote sensing, for a sample of blocks in the US Mid-Atlantic region. We found that building-based density is significantly more accurate than uniform density, so our reported results mostly use the building-based assumption.

## 2. Methods

We use the term ‘hazard zone’ for floodplains or land below a threshold elevation. Estimating the population within a hazard zone requires:

- Data on where people live,
- Data that define the land within the hazard zone, and
- An interpolation method if those two data sets have different geographical precisions.

Previous studies [11, 12] estimated the population in hazard zones such as land within 1 m above mean higher high water (*hereinafter* ‘below 1 m’). Like those efforts, this study uses (§ 2.1) block data from the US decennial census, which counts the number of people in each census block; and (§ 2.2) a grid elevation data set adjusted so that elevations are measured relative to mean high higher water (*hereinafter* ‘sea level’) rather than the fixed geodetic datum. But (§ 2.3) our interpolation approach is different; see supplemental methods §3.

### 2.1. Census block data

The US Constitution requires a decennial census to define the number of Representatives allocated to each state [13]. Since the 1960s, courts have recognized a legal requirement that legislative districts each represent approximately the same number of people [14, 15], which led the Census Bureau to publish population data for small areas known as ‘census blocks’ for an increasing portion of the nation [16]. Since 1990, block population data [17] have been available for the entire United States [16].

**Figure 1.**
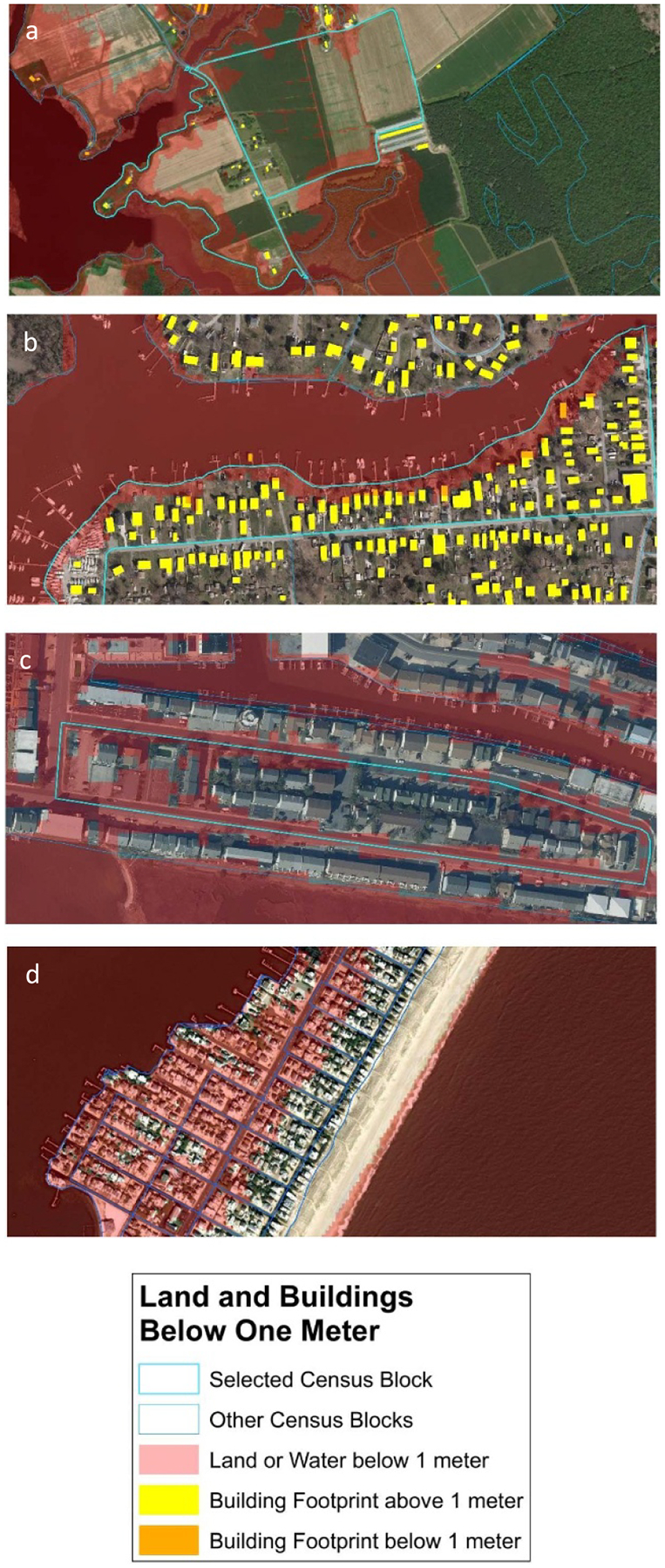
Examples illustrating where the assumption of uniform density within a census block may or may not be reasonable. (a) All but two homes are on the higher ground within two rural blocks in Dorchester County, Maryland; (b) land along the shore below 1 m accounts for 25% of the land area of this block in Baltimore County, MD, but only a few structures are below 1 m; (c) streets and parking areas below 1 m account for almost 40% of this block but only one home is below 1 m in this block in Ocean City, MD; (d) uniform density is a reasonable assumption for these blocks in Brant Beach, New Jersey. The 1 m contour is parallel to the ocean and cuts through the blocks on the ocean side.

In cities, a census block is generally a city block; in less populated places, census blocks cover larger areas. See figure 1. For each census block, the Census publishes the total population of residents and households, and then subdivides those variables into various racial and age classes. For reasons explained in supplemental methods §2, our results are limited to Hispanic, non-Hispanic Black (*hereinafter* ‘Black’), and total populations.

### 2.2. Defining an elevation-based hazard zone

Two widely distributed US elevation datasets are the USGS National Elevation Dataset [18] and NOAA’s sea level rise viewer dataset [19]. The NOAA data were developed with a focus on coastal elevations, but they do not extend far enough inland to capture all low land; so we used the NOAA data where possible and the USGS data elsewhere. We omitted Alaska because data with sufficient vertical precision were not available.

The elevation datasets provide a grid (raster) layer in which each pixel measures elevation relative to the fixed North American Vertical Datum of 1988 (NAVD). Converting that to a grid of elevations relative to sea level involved two steps. First, we subtracted a data layer based on NOAA’s VDatum [20] dataset, which quantifies the height of sea level relative to NAVD for the tidal epoch of 1983–2001. Because 1992 is the midpoint of that tidal epoch, we treat this result as elevation relative to sea level of 1992. Second, we created a data layer representing the annual rate of sea level rise by interpolating tide-gauge data provided by the NOAA’s National Ocean Service [21]. We then subtracted multiples of that layer from the elevation layer to define elevations relative to sea level for a given year.

Few people live in wetlands or open water, so like previous studies [11, 12], we consider only low dry land, by excluding wetlands and open water from the hazard zone. See supplemental methods §§1 and 7.1 for additional details.

### 2.3. Interpolating vulnerability data to match the hazard zone

Our population data have less horizontal resolution than our elevation data. The boundaries of the blocks themselves are precisely defined, but the census only counts how many people live in an entire block, which is rarely smaller than 100 × 100 m and usually much larger. This lack of precision is not a problem where blocks are either entirely within or entirely outside the hazard zone. For example, in New Orleans, 364 000 people live in blocks with at least some land below 1 m, and 313 000 (85%) of them are in blocks that are entirely below that elevation. But outside Louisiana, more than 10 million people live in blocks with some land below 1 m, while only 230 000 (2%) are in blocks entirely below that elevation. (See table 1, columns 1 and 2.)

Previous US studies^2^ [22–26] generally assumed that the fraction of population of each block [11, 27] (or other census areal unit [28, 29]) within a hazard zone equals the fraction of land within the hazard zone. This assumption is often called ‘uniform population density’. Some [11, 27, 30] operationalized this assumption by using (or creating) a raster layer of population density whose grid matched the elevation data, based on the assumption that the population within a given census block is uniformly distributed throughout the block. By contrast, this study (like some older studies [12, 29]) defines each hazard zone as a set of polygons representing the portions of census blocks within the hazard zone.

The third column of table 1 shows the population below 1 m assuming uniform density for various groupings of US states, along with Orleans and Jefferson parishes, Louisiana. (See table S1-A for all states^3^.) Outside Louisiana, uniform density implies that 21% of the residents in the partly vulnerable blocks live below 1 m.^4^

Assuming uniform population density within a census block is reasonable in some areas, but it significantly overstates the vulnerable population in others. In urban areas where census blocks are city blocks, development usually is fairly uniform within a given block. But wherever residential lots are large, people usually build on the high part of their lots. And in rural areas, the high ground may be more developable due to septic tank or floodplain regulations. See figure 1. *A priori*, one does not know whether the upward bias of a population estimate from uniform density is significant.

This study considers an alternative approach based on building footprint data [31]: the fraction of a block’s population in the hazard zone is equal to the fraction of the block’s buildings in the hazard zone. Buildings logically are a better indicator than land area of the fraction of a block’s population living in the hazard zone. (Others have considered road centerlines [32] or occupied parcels [28]). In addition, this ‘building-based’ density approach avoids some spurious trends: blocks from an early census are often subdivided in a later census into a developed block and an undeveloped block. Such remapping changes the population estimates based on uniform density. But the remapping has no effect on an estimate based on building density, because all the buildings remain in the populated block [33]. The fourth column of table 1 shows that the population below 1 m from building-based density is about 2/3 of the estimate from uniform density, outside Louisiana.

To test whether using the building data is worthwhile, we compared the results from both approaches to an independent set of data. To do this, we (a) created two stratified random samples of census blocks, from Maryland and a group of other Mid-Atlantic states, (b) counted the number of housing units at various elevations in each sample block using available imagery, resolving ambiguities with Google Streetview, and (c) estimated total population of units following standard [34] statistical techniques. Table 2 compares the total population estimates from this sample-based method with results from uniform and building-based density. For land below 1 m, uniform density yields an estimate of 331 ± 7 thousand homes, almost twice the estimate from sampling (178 ± 20). The uniform density assumption predicts 73 thousand residents below 1 m in blocks that actually have no homes below 1 m (table 3). The building-footprint estimate of 207 ± 27 is about one standard deviation higher than the actual count. Above 1 m, however, the differences between the three approaches are small.

The preliminary test confirmed that building density provides a better estimate than uniform density. Therefore, the ‘best estimates’ in § 3 rely on the building-based assumption. Extending the sampling to the US coastal zone to measure the nationwide accuracy of the approach was not practicable. Nevertheless, the estimates in table 3 and an error assessment using the same data provide a plausible basis for some general statements about the impact of measurement error. See supplemental methods §8. The coefficients of variation (corresponding to a 66% confidence range) of our results for population below 1 m are approximately:

- 9% for *total population* nationwide, though they range from about 3% in Louisiana to 13%–17% in most states (table S2-A),
- 36% nationwide for *population change* over the period 1990–2020, though they range between 25% and 50% across most states (table S2-B),
- 4% and 12%, respectively, for Black and total population change *in counties with net migration* out of land below 1 m,
- 10% for *ratios of the Black population to total population* (table S2-E); but the ratios are statistically different from 1.0 at the 90% level where the ratio is less than 0.7 or greater than 1.1.

The measurement standard errors for population below 3 m are similar to error below 1 m; but because more people live below 3 m than 1 m, the coefficients of variation are correspondingly less. A key limitation of the error assessment is that the sampling was designed to test the building density assumption, not to detect racial disparity; so uncertainty estimates are imprecise for the relative vulnerability of Black residents and lacking for Hispanic residents. (See supplemental methods §7.5).

### 2.4. Floodplains

We estimated the population living in the mapped 100-year and 500-year floodplains in the same manner, using FEMA’s National Flood Hazard Layer [35] for the A and X500 zones, respectively. This layer is static, has incomplete coverage in many states, and has been widely questioned [36–38]. We characterize it as the ‘mapped floodplain,’ to allow us to calculate an indicator of changing flood hazard from population growth; but we do not claim that it accurately represents the actual number of people at risk. We distinguish coastal from inland floodplains based on land elevation relative to FEMA’s published base flood elevations for a given state (supplemental methods §3.3). We evaluated the same approaches to interpolation as with land close to sea level (see table S2-B), but we have not compared results to an independent data set; so we have no basis for an uncertainty range.

## 3. Results

This section *summarizes* the results of our analysis. Our complete set of results combines 7 hazard zones, 4 decennial censuses, 3 racial classes, 2 interpolation methods, 3 scales (50 states and 2500 counties), and several different ratios of interest. An exhibit for every interesting combination is not practical, so this section uses figures or tables for only some of our results. For other results, we report only one or two numbers, and refer to supplemental tables with more detail.

### 3.1. Total vulnerable population

**Figure 2.**
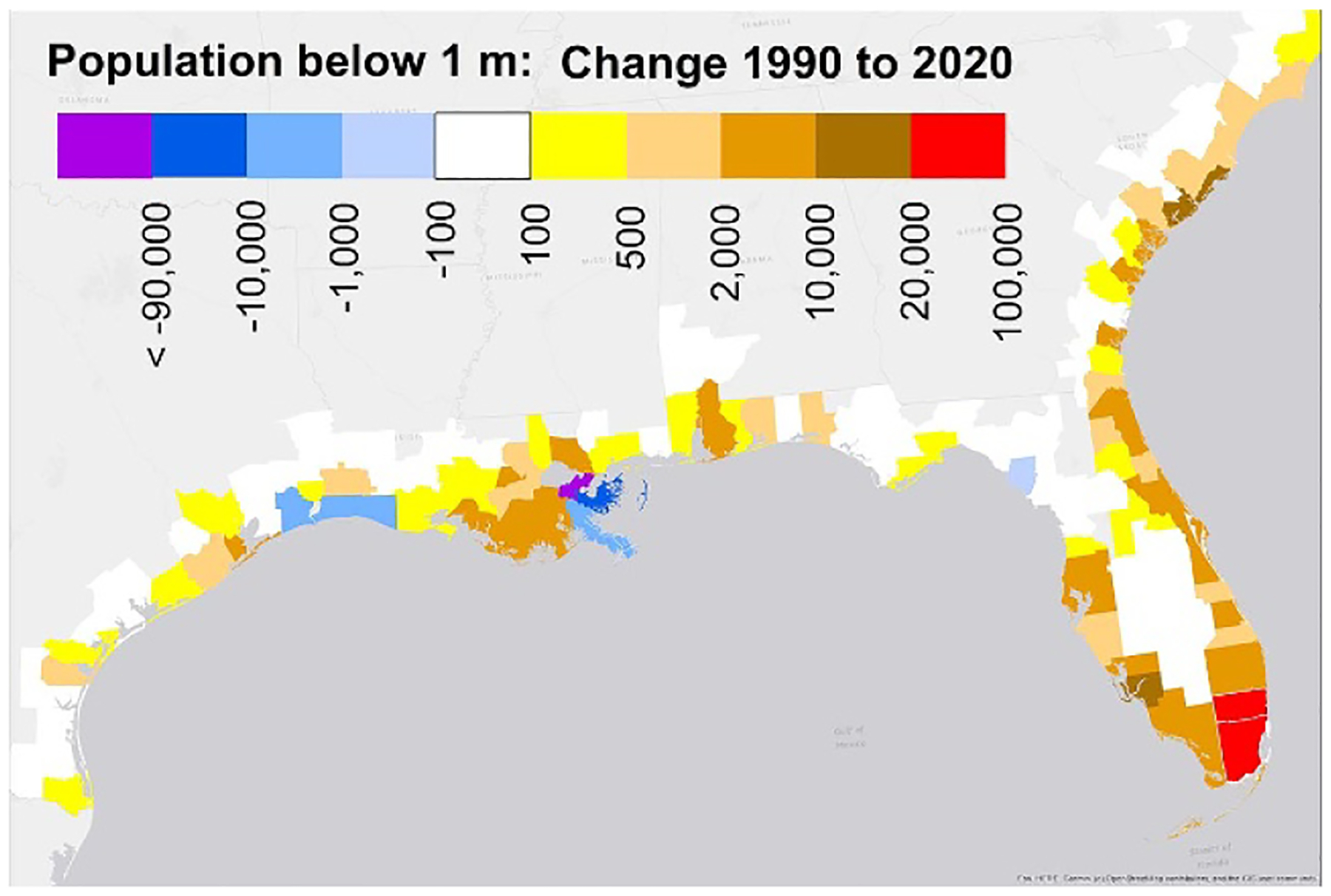
Change in population less than 1 m above MHHW, by county, along the Southeastern US Coast 1990–2020. The standard error is approximately 25%–50% of these estimates.

The total population below 1 m increased 14%–18% from 1.85 (±0.14) million in 1990 to 2.16 (±0.19) million over the 30-year period (table S2-A); population below 3 m increased 31%. Nevertheless, population below 1 m declined 2%–4% between 2000 and 2010, because Hurricane Katrina reduced the total population of New Orleans from 485 000 to 344 000 during that decade, and the population below 1 m from 417 000 to 289 000 (table S3-A). Even today the population of New Orleans is less than 384 000. Mississippi, Texas, Washington and three New England states also saw declines during the 2000s, and the rate of increase dipped that decade in another eight states (table S3-D). But during the 2010s, the population increased below 3 m in every state, and below 1 m in every state except Oregon and Pennsylvania (table S3-A). And over the entire 30-year period, population has increased in the low areas of South Florida by more than the exodus triggered by Katrina (figure 2 and table S3-A). The population along Florida’s Atlantic Coast increased from about 400 000 in 1990 to 600 000 in 2020 below 1 m, and from 3.4 to 5 million below 3 m (table S3-A).

**Figure 3.**
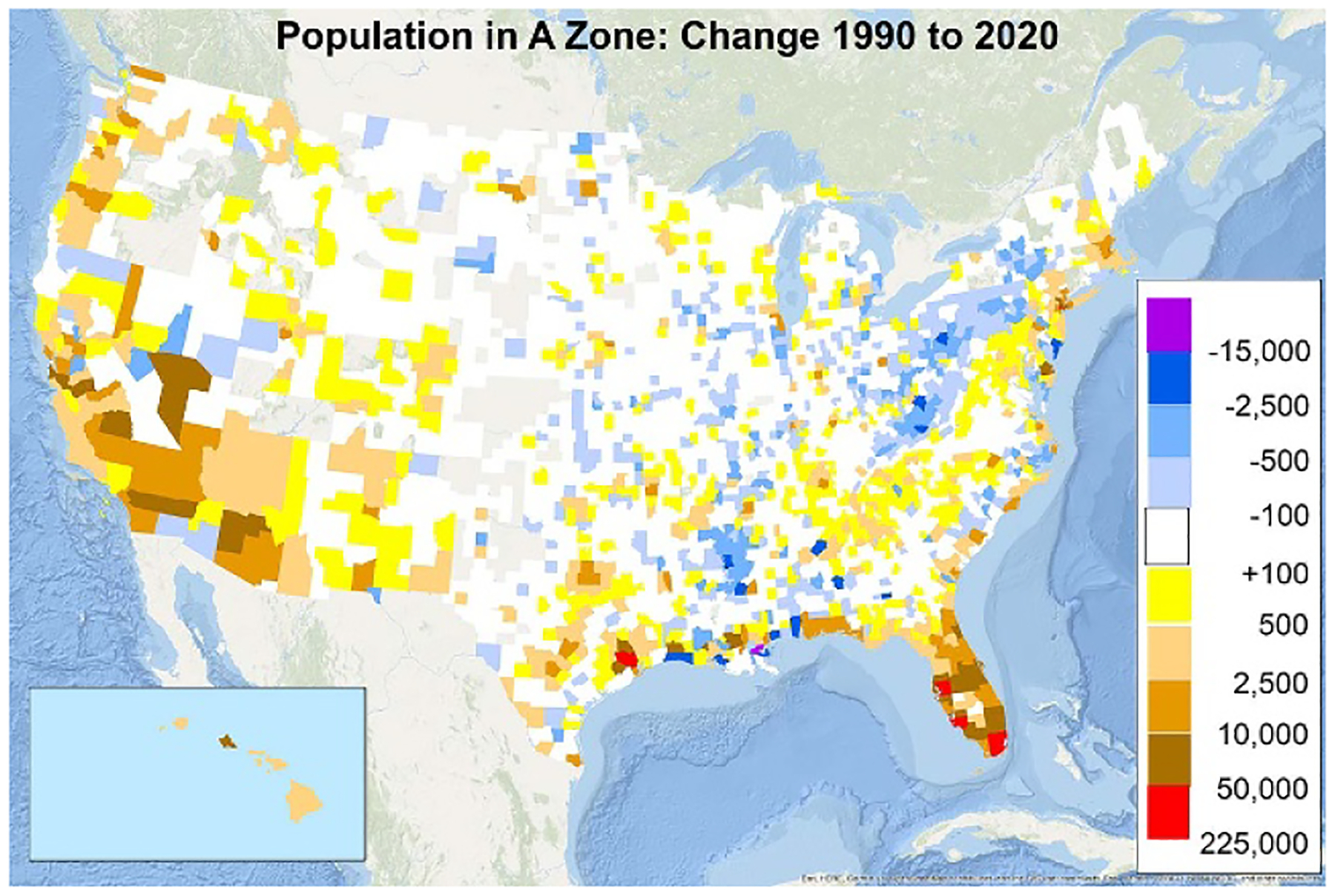
Population change in the 100-year mapped floodplain, by county, 1990–2020.

Changes in the population of coastal floodplains have followed a similar pattern: the population in the 100- and 500-year coastal floodplains increased 22% and 44%, respectively. The greatest declines were around New Orleans, while the greatest increases were in Southern Florida (figure 3); all Gulf Coast states and three New England states experienced a decline in the 2000s; and all but two states saw an increase during the last decade (table S4-C). In coastal states, the population of riverine 100-year and 500-year floodplains increased 27% and 35%, respectively (table S4-E).

As figure 3 shows, however, populations in the 100-year floodplain have declined in many areas along the Mississippi River and Appalachian Mountains. In 234 counties, net emigration from the floodplain was more than 1% of the county’s total population (table S8-E). These declines partly offset increases in the southwest: the total population of 100-year floodplains in non-coastal states only increased by 7%. Nevertheless the population of 500-year floodplains increased by 67`% (table S4-E).

**Figure 4.**
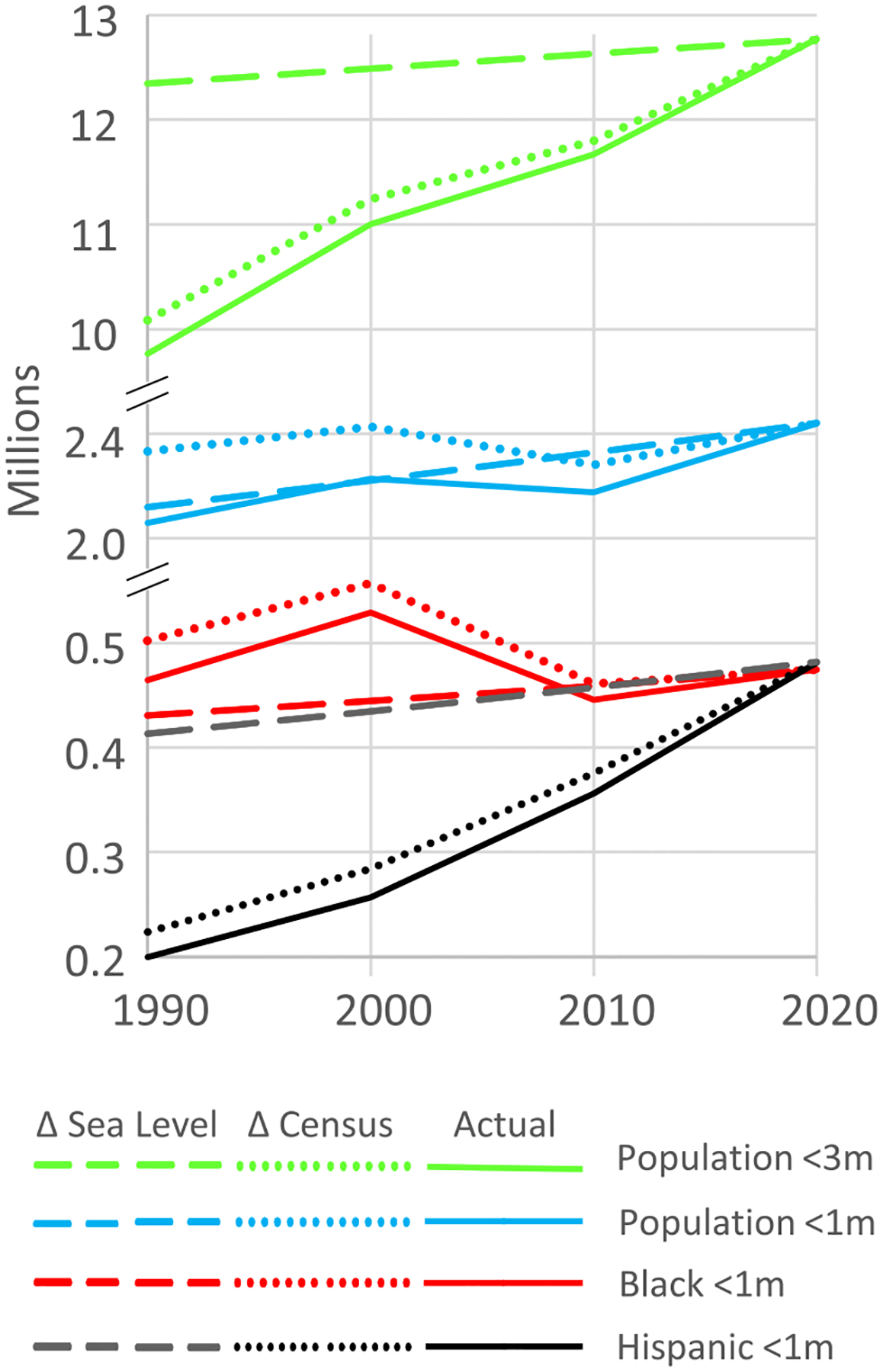
US population close to sea level. Δ *sea level* shows population based on the 2020 census within 1 m or 3 m above sea level for the year depicted. Δ *census* represents population for the census from the year depicted within 1 m or 3 m of sea level for the year 2020. Thus, the dotted blue line shows that the population below the 1 m contour of 2020 declined 150 000 between 2000 and 2010 (because of Hurricane Katrina), while the dashed blue line shows that rising sea level brought an additional 100 000 people below the 1 meter contour, for a net (‘Actual’) decline of 50 000.

Figure 4 differentiates the nationwide increase in low-lying population due to sea level rise from the effect of population changes. As the blue lines show, sea level rise accounts for 75% of the increase in population below 1 m (mainly because the people moving out of New Orleans largely offset people moving into low areas elsewhere). But as the green lines show, sea level rise accounts for only 14% of the increased population below 3 m.

### 3.2. Racial composition of population change

Both the total and Black population below 1 m declined during the 2000s (figure 4). During the entire 30-year period, rising sea level brought another 30 000 Black residents below the 1 m contour, but that increase was approximately offset by the net emigration of Black people from low-lying areas, driven by more than 100 000 Black people leaving New Orleans after Katrina. The Hispanic population below 1 m more than doubled, commensurate with the 120% increase in the Hispanic population of coastal counties (tables S6A and S6C).

These nationwide estimates imply that Black residents disproportionately inhabit land below 1 m. Black residents account for 19% of the population below the 1 m contour but only 12% of the nation’s population (table S6-A). If we define the ‘nationwide ratio of disproportionality’ as simply the ratio of these two percentages, the ratio is 1.63, meaning that Black residents are 63% more likely to live below 1 m than the general population nationwide. While Hispanic residents are not disproportionately likely to live below 1 m, Black and Hispanic residents are each 38% more likely to live below 3 m than the general population. Moreover, Hispanic residents are 50% more likely to live in a 500-year floodplain (table S7).

**Figure 5.**
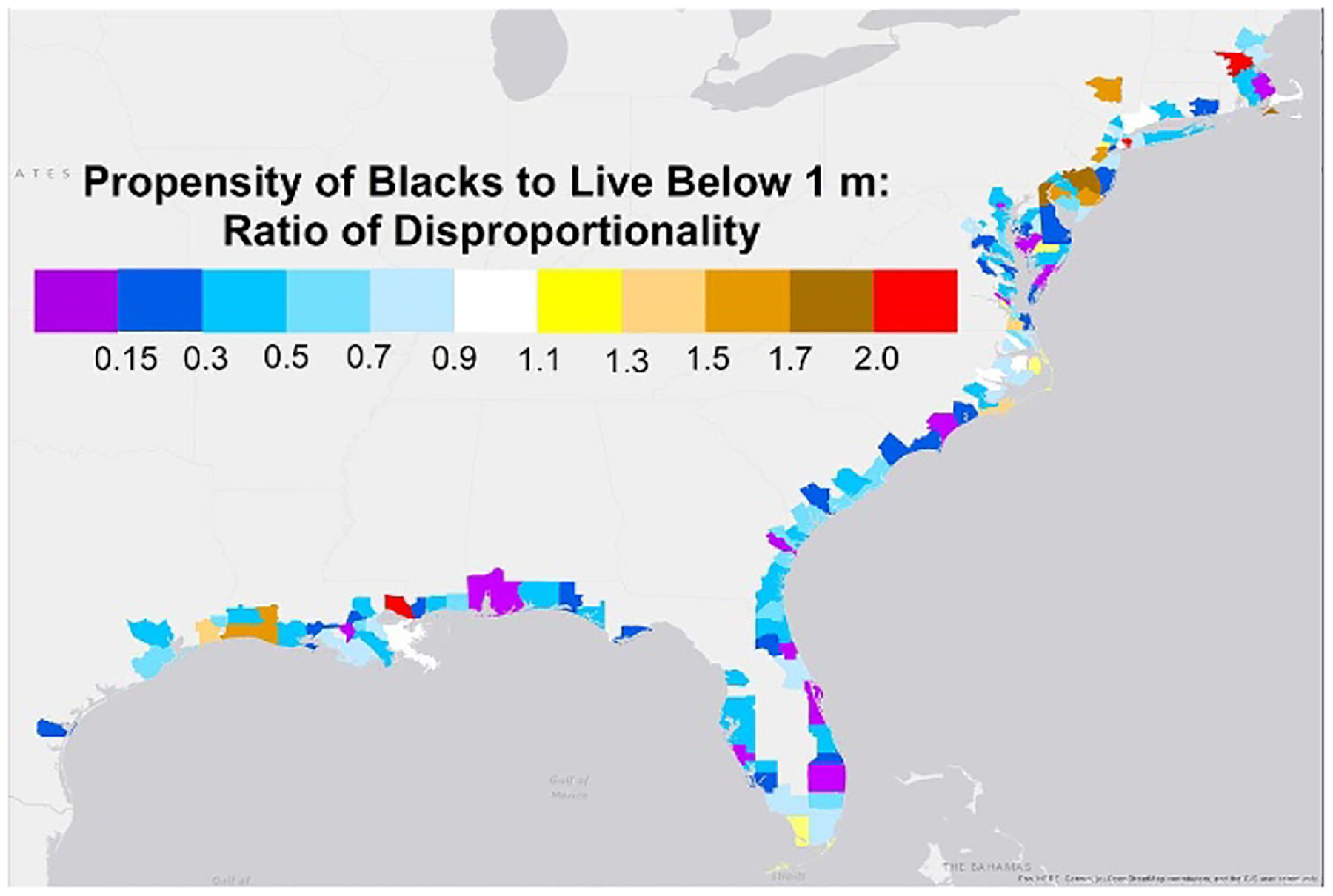
Percent of Black residents who live below 1 m, divided by percent of all residents who live below 1 m, by county, 2020 Census. Ratios greater than 1.1 or less than 0.7 are statistically significant.

Several studies [39–43] have associated a disproportionate exposure of Black people to environmental stressors with the legacy of discriminatory housing policies from the 19th and 20th centuries such as race-based zoning [44], race-based deed restrictions [45], and federal housing programs [46]. Those studies often used the so-called ‘redlining’ maps of the US Home Owners’ Loan Corporation created between 1935 and 1940, which assigned the lowest grade (red) to properties that were vulnerable to flooding or populated by minorities [47, 48]. At first glance, the disproportionate *nationwide* tendency for Black residents to inhabit low-lying land might appear to be another instance of vulnerability resulting from housing discrimination. But figure 5 shows that in most counties, Black residents are *less* likely to live below 1 m than the general population. That is the opposite of what one might expect if housing discrimination caused the disproportionality. Instead, the nationwide disproportionality is primarily because Black (and Hispanic) people tend to live in the counties that are most vulnerable to sea level rise (and flooding). New Orleans is again the most significant example, accounting for 1/7 of the people living below 1 m (table S3-A); and its population is more than half Black. Similarly, Miami-Dade accounts for about 1/5 of the total population below 3 m (tables S3-A) and its population is 2/3 Hispanic. Conversely, Black and Hispanic residents account for only 11% and 12% of the population of localities with no land below 1 m, which is less than their proportions of the nationwide population (12% and 20%, respectively).

**Figure 6.**
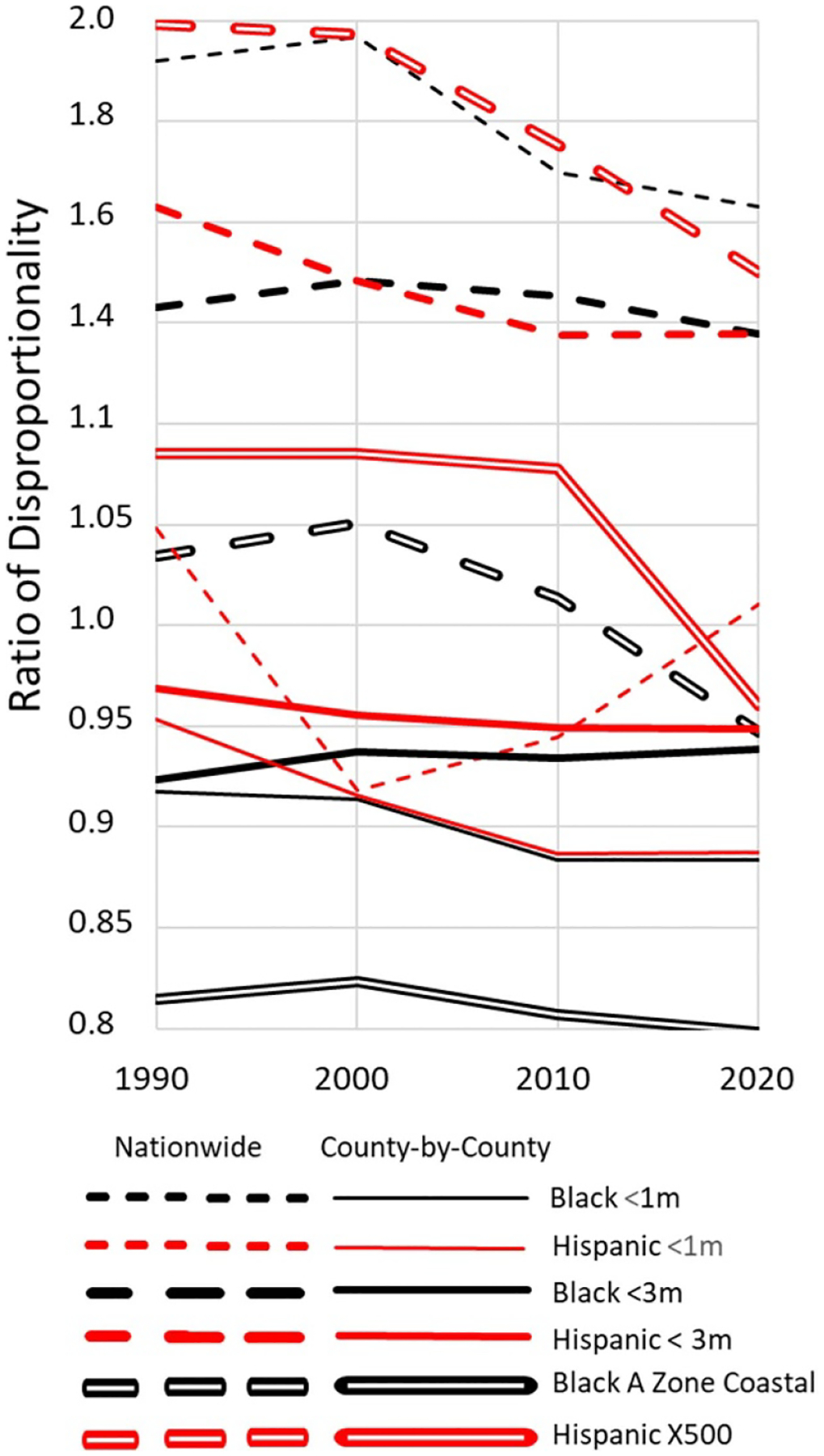
Black and Hispanic residents account for (declining) disproportionate shares of the population living close to sea level or in floodplains.

Figure 6 compares nationwide ratios of disproportionality (dashed lines) with county-weighted ratios of disproportionality (solid lines), which filter out the effects of the different racial compositions of different counties. This county-level measure is the ratio of the nationwide total of Black (or Hispanic) residents in the hazard zone to what the nationwide total would be if, in every county, the number of Black (or Hispanic) residents in the hazard zone was proportional to the number of Black (or Hispanic) county residents. (See supplemental methods §5.) Mathematically this county-level measure can be viewed as an inverse weighted average of the county-specific ratios of disproportionality depicted in figure 5. Intuitively, this ratio indicates the extent to which Black and Hispanic people disproportionately reside in the hazard zones at the county scale. Figure 6 shows the two measures of disproportionality for land below 1 m and 3 m, as well as coastal 100-year flood-plains (Black only) and the 500-year floodplain (Hispanic only). (See table S7 for other floodplains.)

The disproportionate vulnerability of Hispanic residents has declined since 1990, whether aggregated at the national or the county level. The solid lines show that measured at the county scale, Black residents have consistently been about 20% less likely to inhabit a coastal floodplain than the general population of the same county, and about 10% less likely to live below 1 m. Because of the large emigration out of New Orleans after Hurricane Katrina, the Black share of residents living below 1 m nationwide declined from 24% in 2000 to 19.5% in 2020 (table S6-A); the thin black dashed line shows the nationwide disproportionality declining from 1.97 to 1.63. The thin solid line shows that at the county scale, the decline was more moderate: within New Orleans the disproportionality was about 1.09 (cf figure 5), and hence the decline of population there has a small impact on a measure based on county ratios. At either scale, Black residents are less likely than the general population to inhabit coastal floodplains; that result may be an artifact of how FEMA defines the floodplain in New Orleans, which is mostly protected by levees and not mapped as part of the 100-year floodplain. The Black share of residents in coastal floodplains declined because the total population in these flood-plains increased 28% while the Black floodplain population increased only 19% (tables S4-A and S7).

Overlaying our results with the ‘redline maps’ also provides ambiguous evidence of racial disparity. Those redline maps divided 45 coastal and 194 inland urban areas into four risk classifications, including the so-called ‘redlined’ area, which was viewed as the riskiest area for mortgage lenders because of environmental hazards [49, 50] or minority populations [47, 50–53]. At the national scale, people living in redlined areas are 60% more likely than the general population to also be below 3 m (table S12-B, line 11); and this disparity is even greater at the county scale (table S11-B). The coastal redlined areas are now 23% Black (down from 33% in 1990). Yet at the county scale, racial segregation defined by the ‘redlined’ maps have a negligible effect on the proportion of Black residents below 3 m and in 100-year floodplains (tables S12-B and C, lines 17–18). More than 80% of New Orleans and Miami are below 3 m, for example, so most Black residents there would live below 3 m regardless of racial segregation. Although Black residents are concentrated in low-lying red zones around New York, the red zones have a greater share of high ground than other zones around Norfolk, Virginia (table S11-B).

### 3.3. Apparent migration into and out of hazard zones

**Figure 7.**
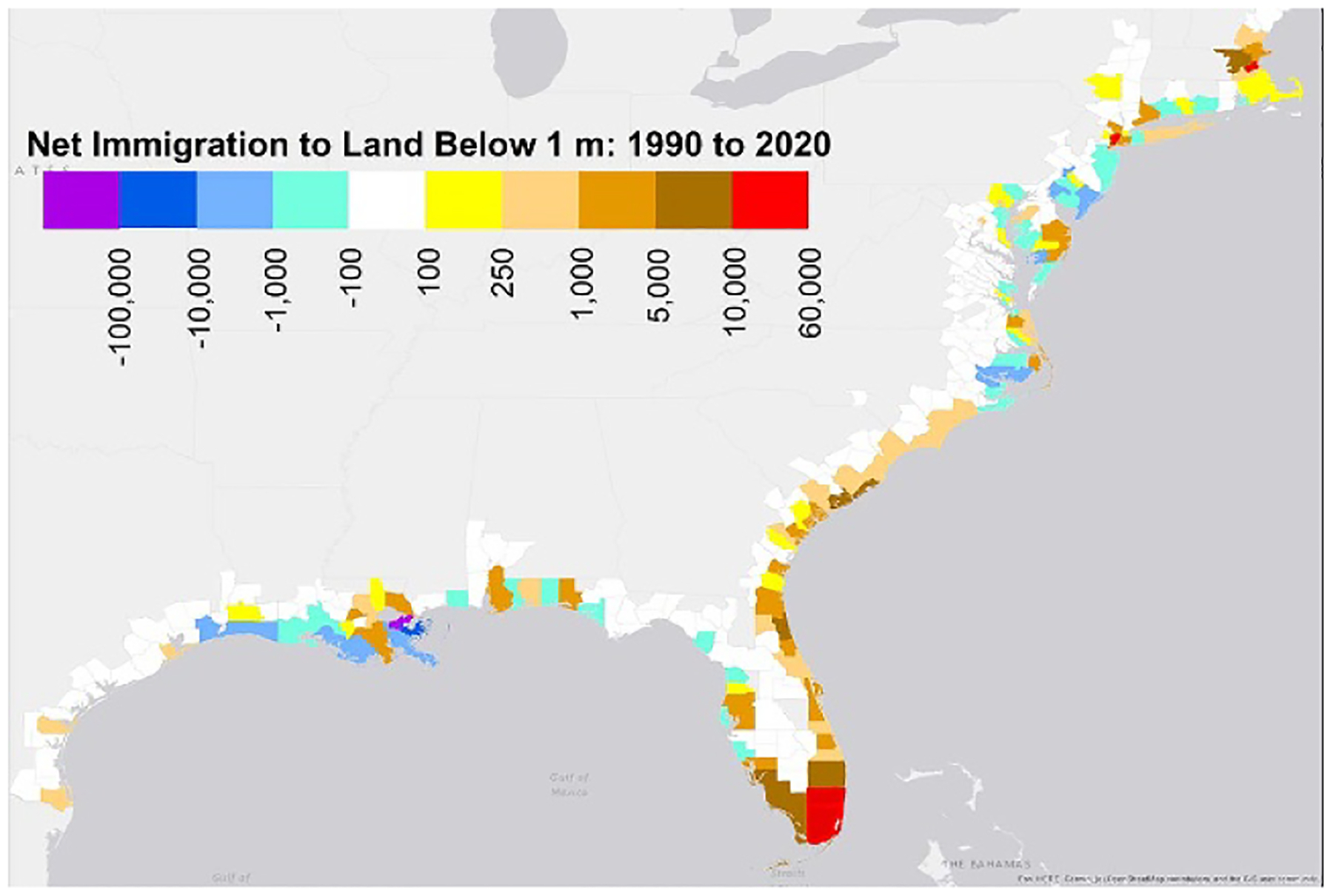
Apparent emigration from land below 1 m in Louisiana, North Carolina, and several other counties, but net immigration into most counties—especially in South Florida.

The population residing in hazard zones has increased nationwide, but people are migrating out of the most vulnerable land in many low-density areas. In figure 7, purple and blue signify counties where the land below the (2020) 1 m contour has lost population since 1990. Table 4 shows apparent emigration by decade below the 1 m contour for the 21 counties where such emigration is greater than 1% of county population. (We say ‘apparent emigration’ because lower population could also reflect more deaths than births.) For the most part, these are counties where one would expect emigration: eight counties from Louisiana where land loss and low elevations are greatest in the nation [9, 54] and ten counties in the middle Atlantic whose planners indicated a decade ago that shore protection is unlikely as sea level rises [8]. See table S14. Nationwide, apparent emigration was approximately 169 000 from land below 1 m and 243 000 from the 100-year floodplain (table 5). See tables S8-A to S8-H for additional details by county.

The two columns along the right side of table 4 show that the disproportionate impact on Black residents is not limited to New Orleans^5^. Black residents of Cape May (NJ), Jefferson (TX), Hyde (NC) and Tyrell (NC) counties emigrated from lands below 1 m at more than twice the rate as the general population. Conversely, Black people showed net immigration into Jefferson and St. Bernard Parishes, replacing a portion of the people from other races who left after Hurricane Katrina.

Nationwide, Black people have emigrated from land below 1 m at almost five times the rate of the general population since 1990. As table 5 shows, even excluding New Orleans, Black emigration nationwide from land below 1 m and 3 m was 91% and 77% greater than the general population, respectively. Black people also migrated from coastal and inland 100-year floodplains at 2.9 and 1.6 times the rates for the general population. Moreover, as the bottom line of table 5 shows, the county-weighted ratios of disproportionality are all between 1.17 and 1.43, which means that the disproportionality cannot be entirely attributed to the tendency of Black residents to live in the most vulnerable localities. Even within a given county (other than Orleans Parish), for example, Black people are 43% more likely than the general population to have emigrated from land below 1 m. For comparison, table 5 also includes the ratios that measure the extent to which Black people reside in the hazard zones (also depicted graphically in figure 6). Whether or not Black residents disproportionately *inhabit* low lying areas depends on whether one views the question at the national or county scale. But at either scale, Black people have disproportionately *emigrated* from floodplains and lands close to sea level since 1990.

## 4. Concluding discussion

This study modestly refined the general approach for estimating population in small hazard zones such as floodplains and lands close to sea level, by assuming that the cumulative distribution of population in a census block follows the *known* shape of the cumulative distribution of buildings in that block. We tested the accuracy of that assumption against the more standard assumption of uniform density on an independent set of observations; and found that the building-based assumption is more accurate. As a byproduct of that test, we also derived ratio estimates and uncertainty for the population below 1 m.

A decade ago, Strauss *et al* [11] estimated the contiguous US population in 2010 within 1 m, 2 m, and 3 m above NOAA’s official mean higher high water to be 3.7, 7.7, and 12.1 million people, respectively. Our comparable estimates—based on uniform density—are 2.6, 7.8, and 12.1 million (table S2-G). The difference for land below 1 m is probably explained by improvements in elevation data, about whose limitations Strauss *et al* warned [55]. Our corresponding best estimates using the preferred building-based density assumption are 2.0, 7.0, and 11.2 million, respectively; and our corresponding ratio estimate is 1.8 ± 0.2 million people below 1 m. Thus, our results generally confirm the accuracy of Strauss *et al*, for population below 2 m and 3 m; but the population below 1 m was probably about half of what they reported. More generally, accuracy can be improved by using building footprint data as an auxiliary variable for interpolating the vulnerable population in blocks that are only partly within a hazard zone of interest.

As one would expect, our estimates for the year 2020 are somewhat higher: a ratio estimate of 2.2 ± 0.2 million people living below 1 m, and best estimates of 2.4, 8.1, and 12.8 million people inhabiting land below 1 m, 2 m, and 3 m. Over the period 1990–2020, the population below 1 m increased 14–18%, with rising sea level accounting for 75% of this increase. Away from the coast, the population of inland 100-year floodplains increased 7%, while the 500-year floodplain population increased 66%.

Increasing population of areas vulnerable to changing climate is not surprising. That finding is generally consistent with the concern that Americans continue to develop the coast with insufficient regard to the fact that sea level is rising while rainstorms become more intense [1, 3]. Nevertheless, the population below 1 m (and 3 m) has declined in a few dozen localities whose vulnerability to sea level rise is well known, notably New Orleans and some low-density counties in the Mid-Atlantic states. In about 100 inland counties, where flood hazard regulations discourage new development in floodplains [56, 57], the population in the mapped 100-year floodplains has declined by more than 2% of the entire population of the county. We estimate a net emigration of approximately 600 000 people from these vulnerable coastal zones and inland floodplains since 1990 (table S8-J).

This letter generally confirms the previous observation that Black and Hispanic residents of the United States disproportionately inhabit land close to sea level [58] or prone to flooding [59]. But we add a nuance: county by county, Black and Hispanic residents are *less* likely to inhabit these hazard zones than the general population in the same county. The nationwide disproportionality exists because Black and Hispanic residents are more likely than the general population to inhabit the most vulnerable counties, such as New Orleans (Black) and Miami-Dade (Hispanic). Even at the county level, however, Black residents have accounted for a disproportionate share of apparent emigration out of floodplains and lands close to sea level. Though Black people are less than 12% of the nation’s population, we found that they accounted for 58% of emigration from land below 1 m, and 28% of the emigration from mapped floodplains. Our overlay with the redlining maps did not detect a link between historical racial segregation and Black people disproportionately *residing* in hazard zones; most of the disproportionate *emigration* took place outside of the urban areas covered by those maps.

This study was designed to measure population trends, not to detect or verify the racial disparity that became evident as we examined our results. Other researchers should investigate whether that finding is accurate and, if so, whether existing policies are contributing to such a disparity.

## Supplementary Material

Preliminary Test of Method

Supplemental Figures

Table of Supplemental Contents

State and County Results

Supplemental Tables (portait)

Supplemental Tables (landscape)

County Population below One Meter

Supplemental Methods

County Thematic Maps

## Figures and Tables

**Table 1.** Population in hazard zone for alternative assumptions for how population is distributed within census blocks (thousands of people, 2020 Census).

	(1)	(2)	(3)	(4)	
Land less than 1 m above sea level					
State or locality	Entirely in zone^a^	All or part in zone^b^	Uniform density^c^	Building-based density^d^	
New England	5.6	856	155		113
NY	3.2	862	148		105
NJ, PA, DE	24	749	201		141
MD, DC, VA	5	986	118		62
NC, SC, GA	12	881	174		103
FL	103	4 682	1 168		753
AL, MS, TX	28	407	86		65
LA	750	1 364	1 003		940
Orleans Parish	313	364	343		333
Jefferson Parish	343	418	396		392
CA	49	459	154		134
OR, WA, HI	0.4	367	47		25
USA	980	11 613	3 253		2 442
USA excluding LA	230	10 249	2 250		1 502
Land within the 100-year floodplain					
USA (riverine only)	1 276	59 930	8 965		4 971
USA (all)	3 384	73 851	14 257		9 353

a Population of blocks that are entirely in the hazard zone.

b Population of blocks with at least 0.5% of dry land in zone.

c Fraction of people in hazard zone equals fraction of block dry land area in zone.

d Fraction of people in hazard zone equals fraction of buildings in zone.

**Table 2.** Estimated housing units close to sea level based on sample of blocks and different estimation methods: Mid-Atlantic States^a^ (thousands of units).

	Elevation relative to MHHW		
	<1 m	1–2 m	2–3 m
Actual count^b^			
Total	178	437	477
${\hat{\sigma }}_{X}$	20	45	50
Uniform density^c^			
Total	331	533	429
${\hat{\sigma }}_{X}$	70	52	50
Building-based density^d^			
Total	207	498	445
${\hat{\sigma }}_{X}$	27	51	49

a New York to Virginia.

b Population estimate of units based on counting homes in overlay of elevation contours and land imagery in sample blocks.

c Based on elevation data and census counts of housing units in the sample blocks.

d Based on elevation data, building data, and census counts of housing units in the sample blocks.

Source: ‘Preliminary Test of Assumptions about Population Distributions within Census Blocks Close to Sea Level’, supplemental methods appendix, table SA-8.

**Table 3.** Ratio estimators of observed to estimated housing units in land below 1 m by stratum (Maryland and rest of Mid-Atlantic).

Density or other defining trait of stratum^a^	*n*	Ratio: observed to estimated^b^			
		Uniform density		Building density	
		$\hat{R}$	${\hat{\sigma }}_{R}$	$\hat{R}$	${\hat{\sigma }}_{R}$
Maryland					
>10	22	0.58	0.11	0.91	0.08
3–10	25	0.17	0.08	0.30	0.19
<3	58	0.44	0.08	0.81	0.09
Tiny block	7	1.00	0.00	*n/a*	*n/a*
Total^d^	112	0.43	0.19	0.72	0.23
Rest of Mid-Atlantic (NY, NJ, PA, DE, DC, VA)					
>35	39	0.49	0.18	0.62	0.40
8–35	40	0.76	0.07	0.97	0.04
<8 & homes	7	0.42	0.17	1.00	0.52
<8 no homes^c^	15	0.00^c^	0.00	0.00	0.00
Tiny block	10	0.90	0.23	1.01	0.26
Diked blocks	19	0.86	0.32	0.98	0.05
Total^d^	130	0.44	0.13	0.82	0.14
Combined Mid-Atlantic					
Total^d^	242	0.44	0.12	0.80	0.13

a Numbers refer to number of housing units per hectare in a census block. ‘Tiny’ refers to blocks smaller than 0.25 ha. ‘Diked’ refers to blocks along the Delaware River with at least 0.5 ha of dry land below mean higher high water. No ratio estimator calculated for other strata, which are entirely below or entirely above 1 m.

b Ratio estimators based on data underlying table 2.

c No homes < 1 m. Uniform and building density predict populations of 73 000 and 700, respectively, for this stratum. Supplemental methods appendix, table 6.

d Combined ratio estimators as defined in Cochran [34, §6.11 and 6.12].

Source: ‘Preliminary Test of Assumptions about Population Distributions within Census Blocks Close to Sea Level’, supplemental methods appendix, table SA-7.

**Table 4.** Counties where apparent emigration^a^ from land below 1 m is at least 1% of county population.

						% County Population^b^	
County and state		1990–2020	1990s	2000s	2010s	Total	Black
Cameron	LA	−2656	−61	−2255	−340	−28.7	−42.9
St. Bernard	LA	−18 356	319	−25 638	6963	−27.5	249.2
Hyde	NC	−1117	−170	−238	−708	−20.6	−43.6
Orleans	LA	−100 979	−11 361	−130 806	41 188	−20.3	−27.3
Tyrrell	NC	−560	257	257	−1073	−14.5	−34.9
Plaquemines	LA	−2040	1076	−3690	574	−8.0	−15.8
St. Mary	LA	−2713	−1997	1172	−1889	−4.7	−3.7
Somerset	MD	−1028	−213	−419	−395	−4.4	−3.9
Pamlico	NC	−408	−129	−109	−170	−3.6	−3.1
Cape May	NJ	−2819	840	−3025	−634	−3.0	−15.3
Beaufort	NC	−1136	−502	−193	−441	−2.7	−7.5
Jefferson	TX	−6393	−2225	−3182	−986	−2.7	−10.2
Accomack	VA	−703	455	−1137	−20	−2.2	0.1
Dorchester	MD	−642	−3	−335	−305	−2.1	−1.1
Jefferson	LA	−8454	5644	−21 027	6929	−1.9	43.3
Salem	NJ	−1220	−631	−548	−41	−1.9	0.6
Vermilion	LA	−933	−174	−501	−258	−1.9	0.5
Mathews	VA	−141	173	−227	−87	−1.7	0.4
Dixie	FL	−171	−53	−61	−58	−1.6	0.2
Gloucester	VA	−416	−147	−267	−1	−1.4	−0.4
Terrebonne	LA	−1024	−241	1991	−2773	−1.1	7.1
Total USA^c^		−169 312^d^	−24 887	−226 444	−29 611	−0.07	−0.37

a Change in population of land less than 1 m above the sea level of 2020. Calculations use building-based density assumption.

b ‘Apparent migration’ for 1990–2020 as a percent of the total or Black population of 1990. Positive values if Black population increased.

c Total of all counties with apparent migration.

d The standard error of this estimate is approximately 19 500.

**Table 5.** Black share of residents and emigration from hazard zones, 1990–2020.

Hazard zone→	Nationwide		Exclude New Orleans		100-year floodplain		All flood-plains
	<1 m	<3 m	<1 m	<3 m	Coast	Inland	
Population migrating out (thousands)^a^							
All races	169	226	68	116	78	243	557
Black	98	119	15	24	26	47	153
Black share of….							
Residents	0.23	0.17	0.12	0.14	0.12	0.11	0.12
Emigration	0.58	0.53	0.22	0.21	0.34	0.19	0.32
Nationwide ratios of disproportionality^b^							
Residents	1.63	1.38	1.12	1.29	0.95	0.96	0.95
Emigration	4.94	4.48	1.91	1.77	2.87	1.65	2.73
County-weighted ratio of disproportionality^c^							
Residents	0.89	0.94	0.78	0.93	0.80	0.98	0.91
Emigration	1.35	1.36	1.43	1.21	1.24	1.17	1.18

a Sum of change in population in hazard zone over all counties where change is negative.

b Ratio of Black share of emigration and vulnerable residents to Black share of population, 2020 and 1990, respectively.

c This measure filters out effect of different racial compositions in different counties. Same as county-by-county disproportionality in figure 6.

## Data Availability

The data that support the findings of this study will be openly available following an embargo of 30 d at the following URL: https://doi.org/10.23719/1527848.

## Footnotes

2 Outside the United States, most studies use population data with a 1 km grid, and assume uniform density within the cells [22–24]. Those data are often created by interpolating larger (e.g. county-scale) population data using remote-sensing indicators of population density, such as land use or night-time light [25, 26].

3 Tables S1 to S14 are available online.

4 (Column [3]–Column [1])/(Column [2]–Column [1]) ranges from 0.115 to 0.256 outside Louisiana for the groupings in table 1.

5 We focus on Black residents because the Hispanic population below 1 m declined by more than 100 in only one county, as the nationwide Hispanic population tripled.
