# Supplementary material for: Population in floodplains or close to sea level increased in US but declined in some counties—especially among Black residents
Source: Environ Res Lett. Author manuscript; Available in PMC 2025 Mar 14. (PMC11908447; doi:10.1088/1748-9326/acadf5)
Supplement: Preliminary Test of Method [file NIHMS1876714-supplement-Preliminary_Test_of_Method.pdf]

PRELIMINARY TEST OF METHOD

Preliminary Test of Assumptions about Population Distributions within Census Blocks Close to Sea Level: Supplemental Methods Appendix

James G. Titus

February 2023

Appendix to Supplemental Methods:
POPULATION IN FLOODPLAINS OR CLOSE TO SEA LEVEL INCREASED IN US BUT DECLINED IN SOME COUNTIES–ESPECIALLY AMONG BLACK RESIDENTS

Environmental Research Letters
2023

Supplemental Methods Appendix: Preliminary Test of Method

Preliminary Test of Assumptions about Population Distributions within Census Blocks Close to Sea Level

1.0 Background

Studies estimating population vulnerable to an effect of climate change based on Census block data usually assume that population is uniformly distributed within any census blocks that are partly in the vulnerable zone. Authors have generally recognized that this assumption may be inappropriate, and some have tested the sensitivity of the assumption. But reported results tend to use the assumption anyway due to the lack of a better alternative.

Here we evaluate the accuracy of the assumption using an alternative data set which we create for a (stratified) random sample of Census blocks in the mid-Atlantic, as a precursor to a planned study estimating population changes in coastal areas vulnerable to sea level rise. For each block in the sample, we then use the digital imagery provided by ArcGIS online and overlay elevation "masks" representing mean high higher water, as well as 1, 2, and 3 meters above MHHW. For moderate- and low-density residential areas, we simply count and record the number of houses within each elevation band; we also count structures crossed by an elevation contour as having 0.5 if, by visual inspection, between 20 and 80 percent of the structure was below a contour. In high-density areas, farmland, commercial or mixed-use areas, we used Google Street view to determine whether a given structure was residential.

For New York, Philadelphia, Ocean City (MD), and a few other high-density areas, we also used Google Streetview to count the number of units in (low-rise) multi-dwelling buildings, where feasible. In very high-density areas, where high-rises made it infeasible to count the number of units in a building, we assigned the number of units to each building in the block based on footprint size and the number of stories; estimated the fraction of a building within the elevation zone to the nearest 10% of the footprint; and assigned units accordingly.

Our original plan was to first conduct this analysis for Maryland alone. We would then compare these observations to the estimates from the uniform density assumption for each of the census blocks in the samples. We would then use what we learned from that analysis to define a sample for the rest of the Mid-Atlantic, repeat the analysis for that region, and then use that analysis to construct a sample for the rest of the United States. If, as expected, the actual count of units was significantly less than estimated by the uniform density assumption, we planned to estimate a Ratio Estimator based on the ratio of counted units to estimated units. We would then provide an alternate estimate of nationwide vulnerability based on the ratio estimator, with confidence limits, based om the sample variance of the ratio estimator.

Here we report only the results of the planned Maryland and Mid-Atlantic samples. In addition to comparing the observations to estimates based on uniform density, we also compare results to estimates based on the assumption that population of households within an elevation band is proportional to the number of building within the elevation band.

This analysis was undertaken in 2019, before we began the analysis of population trends reported in the study to which thus analysis is attached. Therefore, we used the 2010 census. Because most of the available imagery provided by ESRI at the time was from the middle of the decade, the overlay is not perfect; but it would have been no better if matched to the 2020 Census. While coastal development has continued, there is no reason to expect that it has become more concentrated in lower land.

2.0 Sample Creation

2.1 Maryland

We first present the steps we took in creating the sample for Maryland, and then add a brief explanation.

2.1.1 Procedure.

1. Using ArcGIS, created a feature class of census blocks from the 2010 census in Maryland with any dry land below 3 meters and calculated the area of each block, following procedures in Methods Summary.
2. Using ArcGIS, clipped the census blocks with elevation masks that identify land surfaces below the 0, 1, 2, and 3-meter contours, and calculated the area of land below each of those contours. Joined population and housing unit data to the tables associated with those feature classes.
3. Calculated the population density and housing unit density for each block, as well as the estimated population below each of the contours assuming uniform density,
4. Rank ordered the blocks by population density. Created three strata
   a. (1) Blocks with density greater than 10 units/ha, representing about 20% of the estimated population,
   b. (2) a middle category with density between 3 and 10 units/ha, representing the next 40% of population, and
   c. (3) a final category with the lowest density representing 40% of the population.
5. Began counting structures below given elevations for each block, starting with the lowest density where the counting would be easiest. Quickly noticed that many of the low-density blocks were very small, and either had no structures or a single structure according to both visual observation and the census data—but that often the census data was incorrect, typically by mis-attributing a single housing unit to an adjacent block. Concluded that the very small blocks are categorically different and should thus be treated as a separate stratum (which is called strata 4 in Table 1 and stratum 5 in Table 2.
6. Completed the counting. Also noticed that most blocks in the first and second strata either were entirely below one meter or had no land below one meter. As Table 1 shows, Strata 1 and 2 only had 12 and 15 observations, respectively, that would provide any useful information regarding the accuracy of the uniform density assumption for land below 1 meter.

Table 1. Initial Strata of Maryland Analysis: Number of Observations and Characteristics of the associated Unit Population Estimates by Elevation

			Number of blocks in each subsample for which all units (as estimated based on the assumption of uniform population density) are within a given elevation band				
	n	N	100% < 1m	100%>1m	100%<2m	100%>2m	100%<3m
Strata1	39	1377	2	25	9	11	17
Strata2	36	2104	0	21	1	9	6
Strata3	109	10115	5	35	12	15	23
Strata4	26	2727	8	11	14	2	21

Note: 1. Number of observations (blocks) in the subsample
2. Number of blocks in the entire strata.
3. Indicates the number of blocks for which observations will provide no useful information regarding the relationship between actual counts of units and the estimate based on the uniform density. If all (or none) of the land in a block is below 1 meter, for example, then we already know that all (or none), of the units in that block must be below 1 meter, without considering the observations.
4. These two columns indicate the number of blocks in the sample where counting provides no useful information about the population below 1 meter.
5. Counting provides no information about the population below 2 meters.

.

7. Statisticians generally assume that even for a well-behaved distribution, one needs at least thirty observations. So clearly, we needed more observations for the high and moderate density strata.
    a. To ensure that all observations would provide useful information, we subdivided strata 1 and 2. substrata 1a and 2a consist of blocks that are either entirely below 1m or entirely above 2m, while substrata 1b and 2b are blocks that are neither entirely above below 1m nor entirely above 2m. We then boosted the number of observations from substrata 1b and 2b by 27 and 18, respectively, defining these new observations as strata 5 and 7, respectively.
    b. We also subdivided strata 3 based on whether blocks had any residential units. Those blocks with at least one residential can be defined as stratum 3a, while those with no units are stratum 3b. Stratum 3b seemed unlikely to contribute information to the question as to whether the uniform density assumption is appropriate, unless (possibly) those observations were to show that the census had overlooked residential structures that actually exist in those blocks.
    c. We rename the strata as follows:
        i. New strata 1 is what had been called substrata 1a.
        ii. New Strata 2 includes old strata 1b and 5

iii. New Strata 3 is old stratum 2a.
iv. New strata 4 includes old strata 2b and 6.
v. New stratum 5 is old stratum 3a
vi. New stratum 6 is old stratum 3b
vii. New stratum 7 is old stratum 4.

d. Table 3 summarizes the seven strata and the number of blocks for which the observations would provide no useful information given the distribution of elevations in the blocks. Strata 2, 4, and 5 have almost all the relevant observations; and the relative number of observations for these three strata are roughly proportional to the estimated number of units, although strata 5 is slightly over-represented.

Table 2 Subdivision and additional sampling with Initial Stratification for Maryland

Strata number	n	Census Density (units/ha)	Block area (ha)	Is cell either entirely <1m or >2m?
1a	13	>10	>0.25	Yes
1b	26	>10	>0.25	No
5	27	>10	>0.25	No
2a	9	3–10	>0.25	Yes
2b	25	3–10	>0.25	No
6	18	3–10	>0.25	No
3a	85	>0 and <3	>0.25	Maybe
3b	24	0	>0.25	Maybe
4	26	Any	<0.25	Maybe

Table 3. Characteristics of Revised Stratification

Strata number	n	N	Census Density (units/ha)	Block area (ha)	Is cell either entirely <1m or >2m?	Number of cells with no useful info for		
						<1m	1m to 2m	2m to 3m
1	13	371	>10	>0.25	Yes	13	13	3
2	53	997	>10	>0.25	No	31	16	31
3	9	562	3–10	>0.25	Yes	9	9	0
4	45	1525	3–10	>0.25	No	20	2	12
5	85	5073	>0 and <3	>0.25	Maybe	27	15	15
6	24	5068	0	>0.25	Maybe	13	12	8
7	26	2727	Any	<0.25	Maybe	19	16	21

"No useful info" means that blocks are above or below the elevation, so that the block information provides no basis for comparing relative densities. Blocks with a density of zero do provide a comparison, though it is arguable that they cannot be included in a ratio of ratios estimate since the ratio will be 0/0. This is an issue for strata 6 and 7; the others are based on density>0 and this not an issue. Strata 7 accounts for so few structures that we do not bother to stratify it based on density. Strata 6 simply has its own estimate (of zero).

2.1.2 Explanation

We divided the sample into strata based on density because it seemed likely that uniform density would be a reasonable assumption in dense areas, given that city blocks tend to be uniformly developed. Conversely, on very large lots people can choose to build on high ground; and low-density blocks may have a combination of developable and undevelopable blocks. We had intended for the number of observations to be roughly proportional to the population in the various strata, which would mean that the sampling fraction would be greatest in the higher density areas.

We did not foresee that a large number of blocks would provide no useful information because either they are entirely in a single elevation band or they are unpopulated; nor had we expected that more than 20% of the blocks are extremely small with either zero or one residential unit.

2.2 Rest of Mid-Atlantic

We collected the observations and calculated statistics for Maryland before proceeding with the rest of the mid-Atlantic, which we define as New York, New Jersey, Delaware, Pennsylvania, District of Columbia, and Virginia. That experience provided a better idea of what might be an appropriate stratification, so that we would not need to supplement or redefine strata in the middle of the data collection. This time, at the outset, we defined the stratification to differentiate blocks that are entirely in a single elevation band from those that cross elevation bands, and populated blocks from unpopulated blocks, in addition to giving the tiny blocks their own stratum. We also defined a separate stratum for blocks with significant land below mean high water along the Delaware River, where dikes have prevented tidal flooding for centuries.

Unlike Maryland, the rest of the Mid-Atlantic have very densely populated areas with land close to sea level, both in cities such as New York and Philadelphia, and along densely developed barrier islands. While Ocean City, Maryland is a densely populated (in housing units) barrier island, it has relatively little very low land because substantial fill was brought in during the initial development, which came late compared to the New Jersey barrier islands. Therefore, our three density categories had boundaries of 8 and 35 units/ha, instead of 3 and 10 units/ha as was the case for Maryland. We picked these cutoffs based one the following reconnaissance:

- Rank order the blocks by density
- Calculate cumulative population for each block, defined as the population of that block and all blocks with greater density.
- Define the cutoff with 50% and 90% of the cumulative population, which were roughly 35 and 8 units/ha, respectively.
- Collect 50, 50, and 20 observations for each of these strata, respectively.

After further subdividing these density categories to consider different elevation bands, we increased the planned number of observations in some strata to ensure that the subsamples were not unreasonably small. In particular, we made sure that we had 40, 40, and 20 observations for the blocks with some land below one meter within each of the three density-based strata. (For A1bi this was reduced to 39 when the random number generator gave one number twice; some literature suggests counting a duplicate twice, but we did not do so.)

As a practical matter, the higher density (3000-square foot lots) would be row houses and multi-unit structures. We might expect that our ability to count units would be poor with the greatest density blocks (and thus most of the total units in the strata) being multi units' structures, including high rises.

Table 4 defines the different strata. Of the 18 strata we defined, no observations would be useful for 7 of the strata. Two of those strata (A1a, A2a) account for a significant fraction of the population living close to sea level. Although there was no need to collect data because the blocks are entirely in a particular elevation band, they would be included in any estimates of total vulnerable population based on the observations. The other five blocks have no residential units.

Table 4. Stratification for Rest of Mid-Atlantic (NY, NJ, PA, DE, DC, VA)

Stratus	n	N	Stratification Based on Excluding small blocks and dry land behind dikes	Land Elevations in Census Block	Density: units/ha
A1a	[a]	2809	Blocks not excluded by criteria for strata B or C, below.	All land in one elevation band	>8a
A2a	[a]	506			<8
A3a	[a]	2902			0
A1bi	39[b]	1502		Some land <1m	>35
A1bii	40	8705			8 to 35
A2bi	14	10258			<8[c]
A2bii	7	4710			<8[d]
A3b	[a]	11838			0
A1ci	20	2697		No land <1m, some < 2m	>35
A1cii	20	11273			8 to 35
A1di	5	87		No land <2m, some < 3m	>35
A1dii	5	403			8 to 35
A2c	10	10146		No land <1m; some <3m	<8
A3c	[a]	9823			0
B1	[a]	6859	Blocks smaller than 0.25ha	All elevations	0
B2	10	1013		All elevations	>0
C1	19[b]	350	Blocks with >0.5ha below MHHW in counties along Delaware River	All elevations	>0
C2	[a]	242			0
Total		86123[e]			

a. No sampling for strata where sampling would contribute no information.
b. Random sampling within strata gave one observation twice so sample size reduced by one.
c. At least 0.5 buildings in block
d. Fewer than 0.5 buildings in block
e. The total population for sampled strata is 44237.

3.0 Observations

Tables A1, A2, and A3 provide all of data we collected from the procedure described in section 2, except that to save space they do not show the column for units above 3 meters, which can be derived from the columns that are shown. Tables A1 and A2 show comparable results for Maryland and all but stratum C1 from the rest of the Mid-Atlantic. Table A3 shows stratum C1 separately because, as expected, we found structures below mean high higher water in dikes areas for several blocks. As a result, the column for land below 1 meter is subdivided into land below MHHW and land between MHHW and 1 meter.

The tables also show for each block

- The total area of dry land and the area of dry land below the 1-, 2-, and 3- meter contours (and MHHW in the case of table A3) for each block
- The total number of buildings and the number of buildings below the 1-, 2-, and 3-meter contours (and MHHW in the case of table A3) for each block, based on the Microsoft building footprint data.
- The number of residential units and the population for the entire block according to the 2010 Census.

These are the entirety of the data used to estimate the statistics presented in the next section.

4.0 Statistics

Tables 5 and 6 shows the stratum-specific results for Maryland and the rest of the Mid-Atlantic, respectively. The top half of the table shows the sample mean and standard deviations for each stratum. The columns for count are somewhat different than the raw counts, because we multiply the raw counts by the ratio of units in the block according to the census data to the number of units that we observed. Applying this ratio makes our statistics for "count" comparable to those for "area" (uniform density) and "building" (building-based density). For most strata, the sample standard deviation is two to three times the mean. The distribution of population is very skewed across blocks, with most blocks having a small number of units but a few blocks having far more units than the others, which disproportionately increases variance (and standard deviation) by more than they increase the mean. An especially extreme case is caused by block G51017900101072007 in Reedville VA, where there are no homes below 1 meter, but because half the block is below one meter and the block has about half the units of the entire strata, this single block has an estimated 95 units below 1m in stratum A2-b(i), which otherwise averages less than one unit per block.

The second parts of tables 5 and 6 show the population estimates and associated standard deviations for each of the strata. (In this context, "population" means the total for the variable we are estimating, housing units, rather than human population.) For Maryland, we sampled a small number of observations in strata 1 and 3 even though the blocks there are all either entirely below 1 meter or higher than 2 meters; for the rest of the Mid-Atlantic, by contrast, we did not sample the strata of blocks where all land is in one of the elevation bands and thus—for purposes of our population estimate—we can assume that the standard deviation of the population estimate is zero. In the mid-Atlantic, stratum A2(b)(i) contributes almost half of the total variance of the uniform density for land below 1 meter.

TABLE 5. COMPARING NUMBER OF HOUSING UNITS CLOSE TO SEA LEVEL FROM COUNT BASED ON REMOTE SENSING WITH THE NUMBER OF UNITS ESTIMATED WITH CENSUS BLOCK DATA AND BUILDING FOOTPRINT DATA: MARYLAND

(by stratum and elevation above MHHW)

		Below 1 m			Between 1 m and 2m			Between 2m and 3 m			
Stra-tum	a.	Count[b]	Area[b]	Build-ing[b]	Count	Area	Building	Count	Area	Building	n
**Sample by Strata**											
1	$\bar{x}$	5.5	5.5	5.5	0.0	0.0	0.0	4.2	8.9	4.8	13
	$\hat{\sigma}$	14.3	14.3	14.3	0.0	0.0	0.0	8.4	13.5	9.2	
2	$\bar{x}$	1.5	2.7	1.7	11.5	13.4	11.0	11.9	12.5	12.9	53
	$\hat{\sigma}$	5.9	7.6	6.0	18.3	16.3	17.9	19.9	19.3	20.9	
3	$\bar{x}$	0.0	0.0	0.0	0.0	0.0	0.0	1.5	1.3	1.4	9
	$\hat{\sigma}$	0.0	0.0	0.0	0.0	0.0	0.0	2.0	1.7	2.1	
4	$\bar{x}$	0.9	5.1	2.9	14.4	13.0	13.5	9.8	8.1	8.8	45
	$\hat{\sigma}$	3.0	20.7	13.4	50.6	43.6	52.1	26.4	20.9	21.1	
5	$\bar{x}$	0.7	1.4	0.8	2.4	2.3	2.1	3.1	3.2	3.4	85
	$\hat{\sigma}$	2.4	3.4	2.5	8.4	7.2	7.6	13.1	10.8	14.6	
6	$\bar{x}$	0.0	0.0	0.0	0.0	0.0	0.0	0.0	0.0	0.0	24
	$\hat{\sigma}$	0.0	0.0	0.0	0.0	0.0	0.0	0.0	0.0	0.0	
7	$\bar{x}$	0.1	0.1	0.0	0.0	0.1	0.0	0.0	0.0	0.0	26
	$\hat{\sigma}$	0.3	0.3	0.0	0.2	0.3	0.2	0.0	0.0	0.0	
Total Population by Strata[c]											N
1	$\hat{X}$	2,055	2,055	2,055	0	0	0	1,564	3,309	1,783	371
	$\hat{\sigma}_X$	1,468	1,468	1,468	0	0	0	867	1,387	948	
2	$\hat{X}$	1,530	2,655	1,673	11,455	13,409	10,957	11,871	12,476	12,834	997
	$\hat{\sigma}_X$	800	1,023	813	2,505	2,233	2,454	2,730	2,639	2,872	
3	$\hat{X}$	0	0	0	0	0	0	871	721	801	562
	$\hat{\sigma}_X$	0	0	0	0	0	0	379	312	395	
4	$\hat{X}$	1,302	7,799	4,404	21,965	19,841	20,659	15,004	12,326	13,474	1525
	$\hat{\sigma}_X$	671	4,670	3,015	11,508	9,903	11,840	6,005	4,743	4,801	
5	$\hat{X}$	3,642	7,062	4,284	12,065	11,640	10,708	15,501	16,387	17,254	5073
	$\hat{\sigma}_X$	1,310	1,858	1,346	4,644	3,948	4,180	7,218	5,925	8,048	
6	$\hat{X}$	0	0	0	0	0	0	0	0	0	5068
	$\hat{\sigma}_X$	0	0	0	0	0	0	0	0	0	
7	$\hat{X}$	210	210	0	105	196	105	0	14	0	2727
	$\hat{\sigma}_X$	145	145	0	104	135	104	0	14	0	
Total	$\hat{X}$	8,739	19,780	12,416	45,589	45,086	42,429	44,811	45,233	46,146	16323
	$\hat{\sigma}_X$	3,275	9,152	6,227	21,852	18,421	22,324	15,560	12,635	15,144	

Notes

a. $\bar{x}$ refers to the sample mean of a stratum; $\hat{\sigma}$ is the unbiased estimated standard deviation; $\hat{X}$ is the estimate of the total population (i.e., N $\bar{x}$), $\hat{\sigma}_X$ is the estimated standard deviation of $\hat{X}$

b. Count refers to the data collected by observation of the housing units in the sample blocks; Area refers the estimate of units based on the assumption of uniform density; Building refers to the estimate of units assuming that the fraction of units in a block that are below a target elevation is equal to the fraction of buildings in the block below that elevation.

c. Population estimates based solely on the blocks in the sample.

At the bottom of Tables 5 and 6 show, the population estimate for the uniform density assumption is about twice the estimate from counting the structures for land below one meter. The estimates using building footprint data are greater than with counting, but only by about one standard deviation. For land above one meter, the differences between the approaches are negligible in Maryland. For the rest of the mid-Atlantic, the uniform density and building-based density assumptions are less than 10 percent apart, far less than one standard deviation, for both the 1–2 and 2–3 meter elevation bands. The population estimate based on the count is about two standard deviations less than uniform density for the 1–2 meter elevation ban, but about 2 standard deviations more than uniform density for the 2–3 meter band; so the estimates for 1–3 meters differ by less than 5 percent.

TABLE 6. COMPARING NUMBER OF HOUSING UNITS CLOSE TO SEA LEVEL FROM COUNT BASED ON REMOTE SENSING WITH THE NUMBER OF UNITS ESTIMATED WITH CENSUS BLOCK DATA AND BUILDING FOOTPRINT DATA: REST OF MID-ATLANTIC

(by stratum and elevation above MHHW)

		Below 1 m			Between 1 m and 2m			Between 2m and 3 m			
Stra-tum	a.	Count[b]	Area[b]	Build-ing[b]	Count	Area	Building	Count	Area	Building	n
**Sample by Strata**											
A1bi	$\bar{x}$	23.77	48.31	38.36	57.90	61.46	72.77	11.86	15.64	16.24	39
	$\hat{\sigma}$	51.88	89.15	94.46	95.16	87.53	95.82	52.93	51.32	55.42	
A1bii	$\bar{x}$	7.70	10.15	7.92	11.42	9.86	10.88	2.34	1.88	2.58	40
	$\hat{\sigma}$	11.22	11.45	10.77	15.44	11.96	15.15	4.64	3.64	4.72	
A1ci	$\bar{x}$	0.00	0.04	0.00	15.94	37.57	32.10	55.36	41.55	43.08	20
	$\hat{\sigma}$	0.00	0.15	0.00	30.43	46.11	46.90	65.17	60.78	64.52	
A1cii	$\bar{x}$	0.00	0.01	0.01	7.47	10.28	7.79	7.06	6.87	6.97	20
	$\hat{\sigma}$	0.00	0.03	0.03	9.32	10.30	10.08	8.68	7.44	7.84	
A1di	$\bar{x}$	0.000	0.000	0.000	0.000	0.314	0.189	55.76	70.58	72.40	5
	$\hat{\sigma}$	0.000	0.000	0.000	0.000	0.158	0.423	72.09	61.74	71.13	
A1dii	$\bar{x}$	0.000	0.000	0.000	0.000	0.078	0.000	3.91	6.12	3.01	5
	$\hat{\sigma}$	0.000	0.000	0.000	0.000	0.065	0.000	5.40	7.18	4.17	
A2bii	$\bar{x}$	1.472	3.507	1.478	4.06	2.68	3.73	2.23	3.73	2.66	7
	$\hat{\sigma}$	1.754	2.613	1.368	5.73	3.94	4.74	4.58	4.70	4.95	
A2bi	$\bar{x}$	0.000	7.130	0.069	1.079	2.739	1.210	9.92	7.92	9.76	15
	$\hat{\sigma}$	0.000	24.52	0.254	2.132	4.244	2.055	26.00	21.97	25.19	
A2c	$\bar{x}$	0.000	0.002	0.000	0.050	0.500	0.025	2.32	2.93	1.39	10
	$\hat{\sigma}$	0.000	0.006	0.000	0.158	0.863	0.078	4.39	3.50	2.78	
B2	$\bar{x}$	0.170	0.877	0.857	0.180	0.746	0.143	0.05	0.60	0.00	10
	$\hat{\sigma}$	0.365	2.181	2.183	0.336	1.872	0.317	0.16	0.72	0.00	
C	$\bar{x}$	8.305	9.585	8.433	4.163	3.312	4.686	2.22	1.75	1.90	19
	$\hat{\sigma}$	21.77	13.90	20.70	7.779	3.897	8.019	2.69	2.24	2.24	

Notes

a. $\bar{x}$ refers to the sample mean of a stratum; $\hat{\sigma}$ is the unbiased estimated standard deviation; $\hat{X}$ is the estimate of the total population (i.e., N $\bar{x}$), $\hat{\sigma}_X$ is the estimated standard deviation of $\hat{X}$

b. Count refers to the data collected by observation of the housing units in the sample blocks; Area refers the estimate of units based on the assumption of uniform density; Building refers to the estimate of units assuming that the fraction of units in a block that are below a target elevation is equal to the fraction of buildings in the block below that elevation.

TABLE 6. COMPARING NUMBER OF HOUSING UNITS CLOSE TO SEA LEVEL FROM COUNT BASED ON REMOTE SENSING WITH THE NUMBER OF UNITS ESTIMATED WITH CENSUS BLOCK DATA AND BUILDING FOOTPRINT DATA: REST OF MID-ATLANTIC

(continued, by stratum and elevation above MHHW)

		Below 1 m			Between 1 m and 2m			Between 2m and 3 m			
Stra-tum	[a.]	Count[b]	Area[b]	Build-ing[b]	Count	Area	Building	Count	Area	Building	N
**Total Population by Strata[c]**											
A1a	$\hat{X}$	54,821	54,821	54,821	44,724	44,724	44,724	21,779	21,779	21,779	2809[e]
	$\hat{\sigma}_X$	[d]	[d]	[d]	[d]	[d]	[d]	[d]	[d]	[d]	
A2a	$\hat{X}$	1,166	1,166	1,166	523	523	523	788	788	788	506[e]
	$\hat{\sigma}_X$	[d]	[d]	[d]	[d]	[d]	[d]	[d]	[d]	[d]	
A1bi	$\hat{X}$	35,704	72,555	57,620	86,962	92,310	23,486	17,809	23,486	24,387	1502
	$\hat{\sigma}_X$	12,314	21,161	22,422	22,588	20,777	22,746	12,564	12,182	13,155	
A1bii	$\hat{X}$	67,060	88,398	68,975	99,373	85,854	16,349	20,350	16,349	22,417	8705
	$\hat{\sigma}_X$	15,407	15,729	14,784	21,202	16,425	20,806	6,377	4,993	6,486	
A1ci	$\hat{X}$	0	106	0	42,979	101,337	112,059	149,312	112,059	116,181	2697
	$\hat{\sigma}_X$	0	89	0	18,282	27,701	28,181	39,154	36,521	38,768	
A1cii	$\hat{X}$	0	131	64	84,178	115,886	77,472	79,622	77,472	78,629	11273
	$\hat{\sigma}_X$	0	87	64	23,477	25,946	25,387	21,856	18,741	19,743	
A1di	$\hat{X}$	0	0	0	0	27	6,140	4,851	6,140	6,299	87
	$\hat{\sigma}_X$	0	0	0	0	6	16	2,723	2,332	2,687	
A1dii	$\hat{X}$	0	0	0	0	32	2,465	1,574	2,465	1,212	403
	$\hat{\sigma}_X$	0	0	0	0	12	0	967	1,286	746	
A2bii	$\hat{X}$	6,934	16,517	6,960	19,120	12,606	10,787	10,495	10,787	12,547	4710
	$\hat{\sigma}_X$	3,119	4,649	2,434	10,195	6,370	8,432	315	322	340	
A2bi	$\hat{X}$	0	73,136	708	11,068	28,093	81,268	101,736	81,268	100,093	10258
	$\hat{\sigma}_X$	0	64,892	671	5,642	11,232	5,439	2,634	2,225	2,551	
A2c	$\hat{X}$	0	19	0	507	5,075	29,767	23,526	29,767	14,097	10146
	$\hat{\sigma}_X$	0	19	0	507	2,768	249	14,071	11,212	8,906	
B2	$\hat{X}$	172	889	868	182	756	606	51	606	0	1013
	$\hat{\sigma}_X$	116	695	696	107	597	101	50	228	0	
C	$\hat{X}$	2,907	3,355	2,952	1,457	1,159	614	777	614	666	350
	$\hat{\sigma}_X$	1,700	1,086	1,617	607	304	626	210	175	175	
Total	$\hat{X}$	168,765	311,048	194,112	391,074	488,381	455,709	432,669	383,580	399,095	
	$\hat{\sigma}_X$	20,041	70,210	27,033	44,515	48,134	49,900	49,220	44,679	46,921	

Notes

a. $\bar{x}$ refers to the sample mean of a stratum; $\hat{\sigma}$ is the unbiased estimated standard deviation; $\hat{X}$ is the estimate of the total population (i.e., N $\bar{x}$), $\hat{\sigma}_X$ is the estimated standard deviation of $\hat{X}$.

b. Count refers to the data collected by observation of the housing units in the sample blocks; Area refers the estimate of units based on the assumption of uniform density; Building refers to the estimate of units assuming that the fraction of units in a block that are below a target elevation is equal to the fraction of buildings in the block below that elevation.

c. Population estimates based solely on the blocks in the sample, except for strata A1a and A2a, where there was no sampling.

d. $\hat{X}$ = X for this stratum. X is defined as the total number of units in blocks in this stratum, which are entirely below 1 meter, and hence there is no sampling error for this stratum.

e. While this is the number of blocks in the stratum, N is not used to extrapolate a sample mean to the entire population for this stratum, because there was no sampling in this stratum.

The fact that the count is well less than predicted by uniform density below 1 meter—but similar to uniform density above 1 meter—is consistent with what we would have expected: people tend to avoid developing the lowest flood prone lands, and streets tend to be lower than surrounding lots. But most of these lowest lands are below one meter. It is possible that this inflexion point would be lower than one meter had we counted structures below 50 centimeters; and it is also possible that in other parts of the United States it may be higher than one meter, for example, if storms have made development below 1.5 meters impractical.

The only place where we tested for a lower inflexion point was the stratum of diked lands along the Delaware River, where some homes are below MHHW. As Table 7 shows, the ratio estimator for

TABLE 7. RATIO ESTIMATORS FOR OBSERVED HOUSING UNITS BELOW ONE METER AS A MULTIPLE OF THE ESTIMATED HOUSING UNITS ASSUMING UNIFORM DENSITY AND BUILDING-BASED DENSITY

						Ratio of Observed Units to Units Estimated[c] Assuming that Population is Proportional to:				
						Area below 1 meter			Buildings below 1 meter	
	Strata[a]	Density units/ha	n[b]	N[b]		$\hat{R}$	$\hat{\sigma}_R$		$\hat{R}$	$\hat{\sigma}_R$
Maryland										
	2	>10	22	492		0.579	0.109		0.91	0.0756
	4	3 to 10	25	1015		0.167	0.078		0.296	0.1865
	5	<3	58	3810		0.440	0.079		0.807	0.0871
	7	all	7	637		1.000	0.000		n/a	n/a
	Total[d]		112	5954		0.431	0.193		0.722	0.233
New York, New Jersey, Pennsylvania, Delaware, District of Columbia, and Virginia										
	A1bi	>35	39	1461		0.492	0.183		0.620	0.404
	A1bii	8 to 35	40	8510		0.759	0.073		0.972	0.036
	A2bi	<8	7	4686		0.420	0.166		0.996	0.524
	A2bii	<8	15	10205		0.000	0.000		0.000	0.000
	B2	all	10	1016		0.905	0.229		1.012	0.256
	C	all	19	350		0.855	0.318		0.983	0.050
	Total[d]		129	26228		0.442	0.138		0.816	0.136
Combined MidAtlantic										
	Total[d]		241	32182		0.438	0.122		0.804	0.129

Notes:

a. Strata in which all blocks are either entirely above or entirely below 1 meter are omitted.

b. The values of n and N may be different than in Tables 5 and 6 because these calculations exclude blocks that are either entirely above or entirely below 1 meter.

c. Except for the totals across all strata, $\hat{R}$ is estimated as the ratio sample means and

$$\hat{\sigma}_R =, \quad \sqrt{\frac{(1-f)}{n\bar{x}^2}\ (s_y^2 + \hat{R}^2\, s_x^2 - 2\hat{R}s_{xy}}$$

where f is the sampling fraction; s^2_y and s^2_x are the sample variances of the units counted and buildings estimate to be below 1 meter, respectively; and s_{yx} is the comparable sample covariance.

d. $\hat{R}$ and $\hat{\sigma}_R$ are the combined ratio estimators and the associated standard deviation across all strata. See Cochran, W.G. 2011. Sampling Statistics, John Wiley and Sons, §6.11 and 6.12.

uniform density is only 0.85 ± 0.318 below one meter, that is, the actual count tends to be about 15 percent of the amount predicted by uniform density. Here there may be countervailing factors. The high tide range along the Delaware River would tend to discourage development at an elevation above what be acceptable elsewhere, while the dikes mitigate that hazard.

Table 7 shows the ratio estimators (and approximate standard deviations) for the observed building count as a fraction of the number of units predicted by uniform density and building-based density, along with the combined estimators for Maryland, the rest of the Mid-Atlantic, and the entire set of Mid-Atlantic states. For each stratum, the estimator of the ratio is calculated as

$$\hat{R}_h = \bar{y}_h / \bar{x}_h$$

where $\bar{y}_h$ is the sample mean of the number of homes counted[1] in stratum h and $\bar{x}_h$ is the sample mean of the number of residential units estimated by assuming uniform or building-based density.

The standard deviation for the ratio estimator (omitting the stratum-specific subscript *h* for simplicity) is calculated as follows:

$$\hat{\sigma}_R = \sqrt{\frac{(1-f)}{n\bar{x}^2}\ (s_y^2 + \hat{R}^2 s_x^2 - 2\hat{R}s_{xy})}$$

where f is the sampling fraction; s^2_y and s^2_x are the sample variances of the buildings counted and the buildings estimate to be below 1 meter, respectively; and s_{yx} is the comparable sample covariance.

The strata in which blocks all have either no land above one meter or no land below one meter or zero structures are omitted from this table because one does not need to collect data to know that the ratio would either be 1.0 or undefined.

In Maryland, for land with densities between 3 and 10 units/ha, the area-based ratio estimator is only 0.167 ± 0.078, and even the building-based ratio estimator is only 0.296 ±0.1865. The block labeled 24003508107002060 accounts for much of this disparity: As shown in Table A1, about 60 percent of the land in this block but we only counted 5 units (3% of the housing units) below one meter. Most of the housing units are condominiums between one and two meters, while most of the low land is either parking lot or undeveloped. The building footprint data provide a better—but still poor—assessment of this block, for two reasons. First, it counts various utility and commercial buildings and boat houses below the 1-meter contour, and second, most of the housing units are waterfront condominiums in multi-dwelling buildings above the one-meter contour; in this block, a building count overstates residential units in the lowest land while understating units on the higher ground. The block labeled G24004709511001012 accounts for 15 of the 36 units we counted below 1 meter in this stratum. The footprint data found 13.6 buildings in the same zone; but uniform density implies about 55 units. Similarly, in the rest of the mid-Atlantic, the block labeled G34001100101013073. In the rest of the Mid-Atlantic, the uniform density assumption more consistently overstates the number of units; and hence the standard deviation of the ratio estimator R for the Mid-Atlantic is only 0.137, significantly less than the 0.193 standard deviation for Maryland. Table 7 also includes the combined ratio estimators for

[1] As previously mentioned, this total is multiplied by the number of units in the block according to census data to the total number of units counted.

the entire Maryland and rest of the Mid-Atlantic samples, as well as for the entire Mid-Atlantic and associated standard deviations.

Finally, Table 8 compares the estimates of the total units using all the methods examined in this analysis, with a focus on units below one meter. The estimates from the sampled blocks at the top of the table bring together the estimates from the bottom of Tables 5 and 6. For the entire mid-Atlantic, the three sample-based population estimates for units below 1 meter are 177±20, 332±70, and 206±27 thousand units, based on our count, uniform density, and building-based density, respectively. Thus, the count and building-based estimates are almost within a standard deviation of each other, while the area-based estimate is significantly higher. Simply adding the comparable estimates across all census blocks gives us estimates of 319 and 213 for the area- and building-based estimates, well within the range we would have expected from the sample.[2]

The aforementioned estimates have different limitations. Extrapolating a sample to the entire population has the limitation that the sample might not be as representative as one hopes, and even if it is, there is always sampling error. We can avoid the sampling problem by applying our uniform-density

TABLE 8. ESTIMATES OF POPULATION CLOSE TO SEA LEVEL BASED ON VARIOUS STATISTICAL ESTIMATORS

			Maryland			Other Mid-Atlantic States				All MidAtlantic	
			Count	Area	Building	Count	Area	Building	Count	Area	Building
**Estimate from Sampled Blocks Only**[a]											
<1m	$\hat{X}$		8.7	19.8	12.4	168.8	311.0	194.1	177.5	330.8	206.5
	$\hat{\sigma}_X$		3.3	9.2	6.2	20.0	70.2	27.0	20.0	70.2	27.0
1 to 2m	$\hat{X}$		45.6	45.1	42.4	391.1	488.4	455.7	436.7	533.5	498.1
	$\hat{\sigma}_X$		21.9	18.4	22.3	44.5	48.1	49.9	45.4	51.5	51.5
2 to 3m	$\hat{X}$		44.8	45.2	46.1	432.7	383.6	399.1	477.5	428.8	445.2
	$\hat{\sigma}_X$		15.6	12.6	15.1	49.2	44.7	46.9	49.6	49.9	49.3
**Estimate Using all Census Blocks**[b]											
	From a Simple Sum from all blocks										
<1m	.			22.0	15.0		296.6	198.2		318.6	213.2
1 to 2m	.			42.2	40.3		463.5	442.0		505.7	482.3
2 to 3m				39.4	37.9		419.7	414.2		459.1	452.1
	Using Ratio Estimators for Maryland and Rest of Mid-Atlantic[c]										
<1m	$\hat{X}$			10.6	11.2		201.0	176.0		211.5	187.2
	$\hat{\sigma}_X$			3.9	3.2		22.1	22.4		22.4	22.6
	Using Combined Ratio Estimator for Mid-Atlantic[c]										
<1m	$\hat{X}$									210.9	186.8
	$\hat{\sigma}_X$									23.6	24.8

Notes

a. Totals from tables 5 and 6
b. Equal to the population estimate based on uniform density (or building-based density) for each block
c. Equal to R times the unit population estimate based on uniform density (or building-based density) for each block in the strata listed in Table 7, plus the total population of all other blocks.

[2] For land between 1 and 3 meters, the totals are also within the expected range.

or building-based density assumptions to calculate the population in every block, but we know that those assumptions tend to overstate the population of units below one meter.

Ratio estimates can combine the information from both estimates. The bottom of Table 8 calculates ratio estimates of the population for uniform density and building-based density as follows:

$$\hat{Y} = \sum_{h \in H} R_c X_h + \sum_{h \notin H} X_h$$

where $\hat{Y}$ is the ratio estimate of the population of units below 1 meter, R_c represents the combined ratio estimator for the particular sample (from Table 7), and X_h is the estimated total number of units below the one-meter contour for all the blocks (not just the sampled blocks) in strata h. Because of how the combined ratio estimators are calculated, H= {2,4,5,7, A1bi} for Maryland, and H= {A2bii, A2bi, A2bii, B2, C} for the rest of the Mid-Atlantic; that is, we apply the combined ratio estimator R_c only for those strata used to calculate the combined ratio estimator. For the other strata, all methods yield the same estimate of $X_h$because either all or none of the land is below 1 meter.

The standard deviation for the combined population estimate is calculated as follows.

$$\hat{\sigma}_Y = \sqrt{\sum_{h \in H} \frac{N_h^2(1-f_h)}{n_h} \left(s_{y,h}^2 + \hat{R}_c^2 s_{x,h}^2 - 2\hat{R}_c\, s_{xy,h}\right)}$$

where N_h is the number of blocks in the entire population of blocks in stratum h, n_h is the sample size for stratum h, $f_h = n_h/N_h$, and $s_{xy,h}$ is the sample covariance between of x and y in stratum h.[3]

Using the ratio estimators, we get 210±24, or 186±25 thousand units, depending on whether we use the area-based or building-based estimate as the auxiliary variable *x* for calculating the ratio estimate. These two estimates are both significantly less than the "raw" result from a simple sum of all blocks (319 and 213), but greater than merely extrapolating the count (174±20).

Concluding, the ratio estimator has two advantages over simply adding all the blocks: it removes the upward bias from using either of our density assumptions, and it provides an uncertainty range. But unlike simply relying on the random sample, we use all the data and thus could make estimates at a more disaggregated level for jurisdictions within the sample.

We cannot say for certain whether ratio estimators would apply across other regions. The Maryland and rest-of-Mid-Atlantic ratio estimators in Table 7 are similar. That does not necessarily mean that ratio estimators would be same outside the mid-Atlantic; Maryland's development patterns, flood heights, and regulations may be more similar to those in adjacent states than to those in states elsewhere. Nevertheless, lacking an independent data source for the rest of the United States, the only practical alternative to this ratio estimate is the simple building-based density assumption, in effect, assuming that the ratio estimate is 1.0 with no uncertainty. The ratio estimate for the mid-Atlantic seems more plausible. By definition, the ratio estimate was less than 1.0 because in some blocks, the footprint data finds a greater proportion of buildings below 1m than a data set created with visual observation. That disparity occurred primarily because the footprint data include a disproportionate amount of structures near the shore that are not residential, such as large sheds, docks, boathouses, and piers. These types of structures are found in other states. Therefore, it makes sense to present

[3] From Cochran, W.G. 1999. Sampling Statistics. John Wiley and Sons. §6.11 and 6.12.

both the ratio estimates and estimates based on the straight proportionality of the building-based density assumption.

5.0 Extension of the Foregoing Analysis to Black Populations

One of the first people to review this analysis suggested that we use it to project Black and Hispanic population, as well as the total population. We did so, and in so doing noticed that the fraction of the Black population living below 1 meter about 60% greater than the fraction of all US residents living below one meter. After we submitted a paper describing the results to Environmental Research Letters, an anonymous reviewer for that journal advised that the 60% result should not be reported unless it is statistically significant.

Collecting another data set was not practical, so we instead used the same set of observations of housing units from remote sending within our stratified sample of census blocks, along with a single additional assumption: Withing a given census block, the fraction of Black residents living below 1 meter is the same as the fraction of housing units below 1 meter.

Table 9 displays results analogous to Table 7 for all of the strata with at least one Black resident. Comparing these two tables, only 91 of our 241 sample blocks have any Black residents, and only four of our strata have more than ten blocks with Black residents. Within the strata, the distributions are skewed, with 2 blocks accounting for more than half the Black population of all but stratum 5, where the top 4 blocks account for more than half the Black population. Fortunately, the strata that account for more than 90% of all structures below one meter are well-represented.

The building-based ratio estimators $\hat{R}$ for the strata with most of the residents, as well as the combined ratio estimators, are all close to 1.0, meaning that the fraction of buildings below one meter is a good proxy for the fraction on black residents below one meter. (The combined ratio estimators in tables 9 and 10 are slightly different because for table 10, strata A2bii and A2bi were combined to a single strata A2b.) Compared with the general population, Black residents of land below one meter tend to inhabit the more densely developed strata where buildings is a better proxy for habitation (e.g. barns, boat houses, and sheds close to the water are a lower fraction of all buildings).

Although values of R close to 1.0 are not surprising, the very low estimated variances are. One would normally expect a smaller sample size to yield a larger variance for estimated populations and parameters such as R. These very small variance estimates appear to have resulted from a confluence of two factors. First, the relatively few blocks with large Black populations dominate any calculation of a ratio estimator; and second, the observations and the building footprint data give very similar estimates of black population below one meter for those blocks, even though the ratios diverge for smaller blocks. The dominance of relatively few blocks should not undermine confidence in the ratio estimators themselves, as they are generally likely to be the best linear unbiased estimator.[4] But ratio estimates do tend to understate the true variance.[5]

[4] Cochran at 158.

[5] Cochran at 162–164.

TABLE 9. RATIO ESTIMATORS FOR BLACK RESIDENTS BELOW ONE METER BASED ON OBSERVED HOUSING UNITS AS A MULTIPLE OF THE ESTIMATED BLACK POPULATION BASED ON UNIFORM DENSITY AND BUILDING-BASED DENSITY										
						Ratio of Observed Units to Units Estimated[c] Assuming that Population is Proportional to:				
						Area below 1 meter			Buildings below 1 meter	
	Strata[a]	Density units/ha	n[b]	N[b]		$\hat{R}$	$\hat{\sigma}_R$		$\hat{R}$	$\hat{\sigma}_R$
Maryland										
	2	>10	9	414		0.797	0.009		1.000	0.0023
	4	3 to 10	9	847		0.059	0.0556		0.281	0.239
	5	<3	18	3462		0.138	0.0898		0.733	0.298
	Total[d]		36	4723		0.507	0.168		0.935	0.071
New York, New Jersey, Pennsylvania, Delaware, District of Columbia, and Virginia										
	A1bi	>35	26	1001		0.458	0.152		0.894	0.038
	A1bii	8 to 35	15	3264		0.911	0.064		1.040	0.038
	A2bi	<8	4	2721		0.000	0.000		[d]	[d]
	A2bii[e]	<8	1	673		0.347	1.568		0.377	2.082
	C	all	9	158		0.855	0.318		0.983	0.050
	Total[f]		55	7817		0.586	0.137		0.982	0.043
Combined MidAtlantic										
	Total[f]		91	12540		0.585	0.076		0.969	0.022

Notes:

a. As with Table 8, strata in which all blocks are either entirely above or entirely below 1 meter are omitted. In addition, stratum B2 is omitted because the Black population was zero for each block in the sample.

b. The values of n and N may be different than in Table 7 because these calculations exclude blocks with no black population.

c. See Table 7 note C for explanation of calculation of $\hat{R}$ and $\hat{\sigma}_R$, for each stratum.

d. Stratum omitted because the four blocks with a black population have no buildings below 1m.

e. Because only one block in this stratum has a Black population, the standard formula for a sample variance with unknown mean does not apply. Instead, variance for this stratum is calculated based on the assumption that the true mean is known, and equal to the mean from the sample of this strata in Table 7 times the ratio of the black population to housing units for the single observed block..

f. $\hat{R}$ and $\hat{\sigma}_R$ are the combined ratio estimators and the associated standard deviation across all strata. See Cochran, W.G. 2011. Sampling Statistics, John Wiley and Sons, §6.11 and 6.12.

Table A1: Housing Units Counted, Estimated Number of Buildings based on Footprint Data, and Area of Land by Elevation and Census Block: Maryland

GISJOIN	IFC001 (units)	Counted Housing Units <1m	1 to 2 m	2 to 3m	Total	Area of Block by Elevation (ha) Total Area	< 3m	<2m	< 1 m	Buildings Footprint Data by Elevation Total	<1m	1m to 2m	2m to 3m	Popula-tion
G24004709501002024	196	0	0	0	196	0.910	0.188	0.000	0.000	1		0	0.09	1
G24004709503001014	109	0	0	13.5	163	1.141	0.266	0.000	0.000	4		0	0.39	7
G24051000301001019	30	0	0	0	63	0.334	0.017	0.000	0.000	2		0	0	63
G24004709500002096	49	49	0	0	49	0.953	0.953	0.953	0.953	22	22	0	0	39
G24004709501002045	30	0	0	6	30	0.660	0.057	0.000	0.000	2		0	0.05	8
G24000504204011024	30	0	0	30	30	0.894	0.894	0.000	0.000	6		0	6	96
G24000504505033014	11	0	0	0	0	0.341	0.138	0.000	0.000			0	0	28
G24000307301002004	43	0	0	0	43	1.916	0.046	0.000	0.000	7		0	0	106
G24000307012004014	16	0	0	0	9	1.029	0.001	0.000	0.000			0	0	45
G24003909306003022	23	23	0	0	23	1.763	1.763	1.763	1.763	20	20	0	0	34
G24000307011024009	21	0	0	0	21	1.621	0.120	0.000	0.000	22		0	0	46
G24002909505002018	44	0	0	10	45	3.846	0.710	0.000	0.000	17		0	1.05	79
G24000307011024031	14	0	0	0	14	1.312	0.064	0.000	0.000	12		0	0	42
Total Stratum 1	616	72	0	59.5	686	16.718	5.216	2.716	2.716	115	42	0	7.5862759	594
												0	0	
G24051000104002023	62	0	0	30	36	0.383	0.294	0.003	0.000	3		0.03	2.64	103
G24004709501002122	99	0	0	99	99	0.914	0.914	0.131	0.000	6		0.00	6.00	3
G24051002201002035	288	0	19.2	16.8	288	3.138	0.581	0.325	0.000	4		0.24	0.14	307
G24004709501001015	124	1.5	91.5	2	95	2.413	2.413	2.393	0.263	15	0.504	14.25	0.25	32
G24004709500002026	47	10.5	36.5	0	47	0.983	0.983	0.983	0.314	35	8.32	26.68	0	7
G24004709503001023	88	0	1.5	88.5	115	1.867	1.578	0.289	0.000	10		0.18	8.80	3
G24051000203003004	33	0	0	31	33	0.808	0.784	0.006	0.000	1		0.00	0.96	39
G24000307063011018	136	0	0	0	136	3.427	0.429	0.206	0.001	5		0	0	244
G24004709503003035	22	0	22	0	22	0.794	0.794	0.794	0.000	7		7	0	9
G24001909704004006	14	12.5	2.5	0	15	0.583	0.583	0.583	0.501	4	3.07	0.93	0	15
G24000307061011024	28	0	3.5	3.5	29	1.200	0.249	0.191	0.057	15		1.24	1.22	45
G24004709503002012	38	0	38	0	38	1.730	1.730	1.730	0.000	38		38.00	0	17
G24004709503003021	33	0	2	12	14	1.503	1.503	0.481	0.000	13		1.82	11.18	31
G24000307063021002	414	0.5	1	45.5	389	18.909	2.896	1.036	0.346	70	6E-04	0.13	4.42	628
G24004709500001100	19	0	19	0	19	0.917	0.917	0.917	0.024	5	3E-04	4.99969	0	2
G24004709503002046	26	0	26	0	26	1.298	1.298	1.298	0.000	26		26	0	20

Note: IFC001 is the Census variable for residential units.

Table A1: Housing Units Counted, Estimated Number of Buildings based on Footprint Data, and Area of Land by Elevation and Census Block: Maryland

		Counted Housing Units				Area of Block by Elevation (ha)				Buildings Footprint Data by Elevation				
GISJOIN	IFC001 (units)	<1m	1 to 2 m	2 to 3m	Total	Total Area	< 3m	<2m	< 1 m	Total	<1m	1m to 2m	2m to 3m	Popula-tion
G24004709511003077	177	0	0	9	133	9.952	5.877	1.441	0.251	89		0.81537	24.29	192
G24001909704002035	21	0	7	7	18	1.561	0.774	0.408	0.148	7		0.66557	1.26	39
G24004709503003054	17	0	0.5	9.5	16	1.306	0.827	0.186	0.000	12		0.12033	6.53	12
G24004709501001091	15	0	14.5	0.5	15	1.155	1.155	1.143	0.000	15		14.7403	0.26	16
G24000908605021046	5	3.5	2.5	0	6	0.390	0.390	0.390	0.268	6	4.083	1.91719	0.00	6
G24004709517004087	9	0	3.5	5.5	9	0.747	0.747	0.456	0.000	9		3.4637	5.54	6
G24000307313062006	18	2.5	2	8.5	18	1.620	1.193	0.744	0.390	12	0.523	0.46109	5.45	31
G24004109609002024	27	0	8	20	28	2.444	2.444	1.715	0.000	28		18.9596	9.04	35
G24004709507001010	32	3	24	5	32	2.947	2.947	2.608	0.346	31	2.231	24.1164	4.65	52
G24000307012003022	10	0	0	6	9	0.975	0.494	0.021	0.000	9		0.06309	5.32	26
G24002503062001005	66	0	0	66	66	0.833	0.681	0.110	0.000	2		0	1.96	62
G24000307061012022	25	0	18.5	5.5	24	0.347	0.347	0.178	0.000	6		2.87763	3.12	21
G24004709500002066	17	10	13	0	23	0.511	0.511	0.511	0.366	4	2.527	1.47263	0.00	13
G24004709501002009	52	0	0	19	48	1.578	1.077	0.183	0.000	6		0.17299	4.81	3
G24051002402001012	70	0	0	11	77	2.152	0.611	0.003	0.000	9		0	1.82	149
G24051002303001051	10	0	0	0	0	0.503	0.426	0.062	0.000			0	0.00	15
G24004709503004031	18	0	18	0	18	0.947	0.947	0.947	0.033	7	0.189	6.81089	0.00	14
G24004709503004032	35	0	34.5	0.5	35	1.916	1.916	1.427	0.000	7		3.0385	3.96	18
G24004709503003065	20	0	16	2	18	1.161	1.161	1.127	0.000	18		16.9534	1.05	19
G24002909503004037	24	0	0	12.5	26	1.400	0.861	0.041	0.000	11		0	7.31	33
G24000307070013015	9	0	3	5	8	0.562	0.562	0.562	0.291	8	3.668	4.33241	0.00	0
G24004709517004086	12	0	2.5	9.5	12	0.761	0.761	0.268	0.000	12		1.9366	10.06	7
G24004709500002028	27	0	27	0	27	1.795	1.795	1.795	0.097	4		4	0.00	17
G24003909306001096	65	39	23	0	62	4.464	4.464	4.464	3.491	36	22.69	13.3132	0.00	197
G24000504213001003	24	0	25	0	25	1.689	1.689	1.689	0.000	26		26	0.00	68
G24000307070013025	16	0	17	0	17	1.140	1.140	1.140	0.021	17	0.003	16.997	0.00	33
G24001909703001057	5	0	0	3	4	0.376	0.309	0.078	0.000	4		0	2.58	13
G24000504203031044	17	0	0	4.5	18	1.280	0.419	0.009	0.000			0	0.00	43
G24003708761003073	10	0	0.5	10.5	11	0.762	0.762	0.118	0.000	11		0.15415	10.85	18
G24004709517001016	45	0	7.5	32.5	44	3.620	3.397	1.154	0.000	44		5.24871	34.45	62
G24004709511002018	55	0	23.5	19.5	43	4.463	4.463	2.502	0.090	47		28.4986	18.50	90

Note: IFC001 is the Census variable for residential units.

Table A1: Housing Units Counted, Estimated Number of Buildings based on Footprint Data, and Area of Land by Elevation and Census Block: Maryland

		Counted Housing Units				Area of Block by Elevation (ha)				Buildings Footprint Data by Elevation				
GISJOIN	IFC001 (units)	<1m	1 to 2 m	2 to 3m	Total	Total Area	< 3m	<2m	< 1 m	Total	<1m	1m to 2m	2m to 3m	Population
G24000908604013018	67	0	0	0	67	5.703	0.706	0.300	0.074	12		0	0.00	156
G24000307011021037	15	0	0	1.5	14	1.293	0.239	0.050	0.000	13		0	1.95	51
G24004709517001031	9	0	6	3	9	0.784	0.784	0.641	0.000	9		5.87082	3.13	13
G24000307070013027	12	0	12	0	12	1.137	1.137	1.137	0.057	13	0.031	12.9693	0.00	21
G24000504519002019	11	0	11	0	11	1.064	1.064	1.064	0.000	12		12	0	27
G24000307070012035	9	0	9	1	10	0.899	0.899	0.828	0.053	8		7.04752	0.95	18
Total Stratum 2	2547	83	592	606	2414	107.10	67.92	42.87	7.48			0	0	3101
												0	0	
G24000307302031015	8	0	0	0	8	0.868	0.017	0.000	0.000	8		0	0	37
G24004709507002031	221	0	0	4.5	221	29.210	0.715	0.000	0.000	159		0	4.77	286
G24004709507002035	87	0	0	0.5	86	12.265	0.144	0.000	0.000	56		0	1.02	130
G24004709515004002	2	0	0	4	4	0.393	0.388	0.000	0.000	4		0	4	6
G24001909704003022	18	0	0	1.5	16	3.782	0.296	0.000	0.000	17		0	1.72	25
G24001708512004124	13	0	0	0	13	2.916	0.004	0.000	0.000	18		0	0	22
G24003708758023012	10	0	0	0	10	2.476	0.013	0.000	0.000	9		0	0	22
G24003508101002080	6	0	0	7	8	1.887	0.249	0.000	0.000	7		0	0.9196555	14
G24001909707022096	1	0	0	0	1	0.330	0.238	0.000	0.000	1		0	0.00	2
Total Stratum 3	366	0	0	17.5	367	54.13	2.06	0.00	0.00			0	0	544
												0	0	
G24001909704003020	21	0	2	7.5	19	2.139	0.964	0.146	0.000	19		1.88823	7.39	53
G24004709500002041	9	0	9	0	9	0.919	0.919	0.919	0.260	1		1	0	0
G24004109609002021	5	2	2	1	5	0.543	0.543	0.520	0.220	8	3.443	4.23292	0.32	5
G24000908605021061	8	0	0.5	1.5	8	0.910	0.280	0.154	0.000	9		0.7434	1.26	10
G24000504213002013	108	0	33	60	93	12.338	12.299	7.434	0.485	34		10.0233	23.98	269
G24003508107002060	202	5.5	124.5	23	153	24.188	24.188	23.627	15.645	57	25.23	26.3152	5.45	206
G24000307012004007	15	0	0	2	15	1.841	0.231	0.041	0.000	15		0	1.59	38
G24000504519003025	21	0	4.5	15.5	20	2.786	2.753	0.925	0.048	17		2.87581	14.12	46
G24002503061002008	8	0	0	2	8	1.077	0.644	0.057	0.000	4		0	1.02	12
G24000307070022017	9	0	1.5	7.5	9	1.233	1.233	1.057	0.001	9		7.88086	1.12	20
G24004709506002002	44	0	16	14.5	31	6.378	6.236	2.724	0.015	30		10.9288	19.07	92
G24001909703001111	5	0	0	2	5	0.765	0.461	0.012	0.000	7		0.00597	3.64	11

Note: IFC001 is the Census variable for residential units.

Table A1: Housing Units Counted, Estimated Number of Buildings based on Footprint Data, and Area of Land by Elevation and Census Block: Maryland

GISJOIN	IFC001 (units)	Counted Housing Units <1m	1 to 2 m	2 to 3m	Total	Area of Block by Elevation (ha) Total Area	< 3m	<2m	< 1 m	Buildings Footprint Data by Elevation Total	<1m	1m to 2m	2m to 3m	Population
G24000908609003143	27	0	9	14.5	26	5.065	4.630	2.605	0.607	35	1.7	9.27008	16.65	51
G24000307511022017	8	0	0	0	8	1.612	0.191	0.008	0.000	7		0	0	23
G24002909503004008	9	0	0	0	9	1.884	0.248	0.151	0.000	5		0	0	22
G24003508109011019	21	0	0	8	21	4.455	2.471	0.321	0.000	21		0	9.42	51
G24000307012002005	20	0	0	0	18	4.336	0.852	0.610	0.257	19		0	0.05	39
G24004709517004068	20	3	6	10	19	4.336	4.336	3.619	1.129	16	3.855	7.14507	5	20
G24000307307004032	75	0	0	0	75	16.961	1.327	0.987	0.574	74	1	0	0	179
G24000307070021022	8	0	2.5	4.5	7	1.860	1.860	0.887	0.022	11	0.463	5.35423	5.18	14
G24003708754003033	3	0	0	2	4	0.734	0.255	0.015	0.000	5		0	2	5
G24000504525001009	26	3	8	29	52	6.372	4.876	1.474	0.488	54	4.181	9.00174	29.82	54
G24003508109022023	8	0	0	8	8	1.984	1.973	0.006	0.000	7		0.00134	6.76	28
G24004709512003133	5	0	0	2.5	6	1.266	0.645	0.058	0.000	8		0.03531	3.49	13
G24000504518022041	45	1.5	45.5	3	50	13.180	12.801	11.327	1.950	61	2.467	53.1687	5.36	80
G24002909504003119	1	0	0	0	0	0.321	0.290	0.011	0.000			0	0	5
G24004109608002059	2	0	1	0	1	0.662	0.662	0.662	0.273	1		1	0	2
G24000307012003044	8	0	0	6.5	8	0.846	0.682	0.029	0.000	9		0.01069	7.18	19
G24004709501001090	7	0	10	0	10	0.827	0.827	0.827	0.000	10		10	0	10
G24051000203002011	7	0	0	0	5	0.833	0.833	0.113	0.000	1		0.03818	0.96	11
G24004109607002069	8	0	4.5	3.5	8	1.153	1.153	1.015	0.000	9		5.9876	3.01	9
G24000307012002020	94	0	2.5	4	93	15.019	3.288	1.829	0.757	98		2.63518	5.30	160
G24004709511001012	494	15	253	138.5	406	79.865	77.974	55.641	8.958	401	13.6	280.267	100.99	712
G24000307312024022	29	0	0	3.5	31	6.016	1.394	0.690	0.281	26	1.196	0.49035	3.10	71
G24000307021001029	22	0	0	0	22	5.086	0.261	0.151	0.059	25	3	0.74231	0.26	37
G24000307066002022	44	0	0	0	44	10.300	0.664	0.502	0.342	42		0	0	96
G24004709501003004	15	0	0	0	0	3.632	2.552	1.236	0.000			0	0	0
G24000504517021001	20	0	11.5	13.5	33	4.940	3.472	1.896	0.049	35		12.8071	13.65	33
G24003708750002027	11	0	0	9.5	13	2.850	2.430	0.289	0.000	12		0.01623	8.90	28
G24004109607002023	3	0	0	0	0	0.781	0.781	0.546	0.185			0	0	1
G24000504517022014	6	0	0.5	11.5	16	1.691	1.306	0.209	0.000	15		0.06566	11.97	16
G24003708750002009	12	0	7	6	13	3.404	3.404	2.404	0.110	13		6.70509	6.29	36
G24000307311052009	46	0	0	0.5	46	13.459	0.402	0.125	0.033	41	1	0	0.64	105

Note: IFC001 is the Census variable for residential units.

Table A1: Housing Units Counted, Estimated Number of Buildings based on Footprint Data, and Area of Land by Elevation and Census Block: Maryland

		Counted Housing Units				Area of Block by Elevation (ha)				Buildings Footprint Data by Elevation				
GISJOIN	IFC001 (units)	<1m	1 to 2 m	2 to 3m	Total	Total Area	< 3m	<2m	< 1 m	Total	<1m	1m to 2m	2m to 3m	Population
G24004709511003089	1	0	0	0	0	0.312	0.299	0.190	0.000			0	0	2
G24001500309061100	9	6	3	2	11	0.907	0.907	0.827	0.385	10	5.798	2.16	2.04	5
Total Stratum 4	1569	36	556.5	417	1438	272.07	190.80	128.87	33.13			0	0.00	2699
												0	0	
												0	0	
G24003508103001033	5	1	2.5	1.5	5	1.783	1.783	1.535	0.555	7		5.39	1.61	7
G24003708753001036	4	0	0	0	5	1.612	0.085	0.000	0.000	6		0	0	5
G24003708752021057	6	0	0	2	6	2.471	0.966	0.048	0.000	7		0	2	9
G24003708753003077	7	0	0	7.5	9	3.248	2.783	0.000	0.000	9		0	7.50	12
G24001708514004042	1	0	0	2	2	0.470	0.470	0.000	0.000	3		0	3	1
G24000307070023030	31	0	3	24.5	29	14.704	14.594	2.288	0.000	29		2.24	26.17	88
G24004709513002015	33	0	4.5	6	39	16.193	5.955	2.460	0.803	44	9E-04	4.42	4.59	77
G24003508110001023	6	0	0	2	6	3.069	1.534	0.112	0.029	9		0	2.47	12
G24003708761004108	16	9.5	8.5	0	18	8.523	8.523	8.523	5.639	18	11.68	6.32	0	25
G24000908608023028	8	0	0	0	8	4.335	0.078	0.011	0.000	5		0	0	21
G24000908609002036	15	0	0.5	1	18	8.244	3.357	2.320	1.353	18	0.002	0.20632	1.34	23
G24003909301022083	1	0	0	0	1	0.594	0.370	0.104	0.068	1		0	0.00	3
G24000307070012006	91	7.5	49.5	33.5	93	55.959	55.497	29.251	5.606	79	6.083	42.61	29.16	173
G24003708759011017	149	0	0	0	149	92.033	15.784	6.863	2.559	21		0	0.00	381
G24004109609001234	2	0	1.5	0.5	2	1.286	1.286	0.965	0.110	1		0.20982	0.79	4
G24000908609004048	21	0	0	0	0	13.637	2.990	1.876	0.873	25	1	0	0.00	31
G24000307011024002	21	0	0	0	17	15.039	2.043	1.461	0.773	27		0	0.00	40
G24001909703004009	6	0	0	0	5	4.356	0.762	0.394	0.119	7		0	0.00	17
G24001909703003003	48	3	3	12	48	37.817	11.401	5.205	2.142	58	3.298	3.05	16.15	105
G24001909707024011	14	0	2.5	3	14	11.082	5.510	3.402	1.118	15		2.91	3.74	28
G24004109602013035	4	0	0	0	4	3.438	0.805	0.402	0.240	4		0.00	0.00	6
G24004109602013075	13	0	0	2.5	16	11.320	6.493	3.700	1.501	15	1	0.06	4.21	19
G24004109602013065	5	0	1	1.5	5	4.438	2.579	1.721	0.545	6		0.89	1.87	8
G24001909703001062	1	0	0	0	0	0.910	0.214	0.062	0.000			0.00	0.00	3
G24000307014001032	28	0.5	6	8.5	24	25.687	9.908	5.955	2.644	33	1.004	6.34	13.63	76
G24004109606001045	1	0	0.5	0.5	1	0.935	0.935	0.716	0.000	2		0.58	1.42	2

Note: IFC001 is the Census variable for residential units.

Table A1: Housing Units Counted, Estimated Number of Buildings based on Footprint Data, and Area of Land by Elevation and Census Block: Maryland

		Counted Housing Units				Area of Block by Elevation (ha)				Buildings Footprint Data by Elevation				
GISJOIN	IFC001 (units)	<1m	1 to 2 m	2 to 3m	Total	Total Area	< 3m	<2m	< 1 m	Total	<1m	1m to 2m	2m to 3m	Popula-tion
G24004500103004011	255	0	0	0	255	261.656	0.204	0.070	0.000	266		0.00	0.00	756
G24001708512001023	242	10	62	119.5	251	251.167	171.514	80.651	24.146	233	8.03	47.17	124.47	482
G24002909502002070	25	0	0	0	25	26.208	1.597	1.005	0.541	27		0.00	0.00	36
G24001909701002108	5	0	3	1	6	5.402	4.684	4.027	0.938	9		4.17	1.95	11
G24004109602014021	18	0	0	0	18	20.302	0.143	0.000	0.000	19		0.00	0.03	42
G24001109555001001	10	0	5.5	5	12	11.337	10.269	4.765	0.931	16	0.187	4.95	9.80	18
G24004709504002038	1	0	0	1	1	1.235	0.924	0.068	0.000	1		0.00	1.00	2
G24004109602013098	24	0	0	0	23	30.167	0.324	0.000	0.000	19		0.00	0.00	53
G24003708761002053	60	0	1	2	68	75.876	6.295	3.232	1.688	73	0.055	0.21	0.65	126
G24001909709002295	15	15	0	0	15	18.982	18.982	18.982	18.982	18	18	0.00	0.00	18
G24000504518023045	2	0	0	0	2	2.673	0.735	0.000	0.000	2		0.00	0.00	4
G24003708760013002	103	0	0	0	100	138.453	0.006	0.006	0.006			0.00	0.00	258
G24001500309041009	24	0	0	0.5	25	34.346	8.211	5.481	3.000	27	1	1.42	0.66	51
G24004109602012127	1	0	0	0	1	1.443	0.479	0.209	0.083	1		0.00	0.11	0
G24003909305003019	2	0.5	2.5	0	3	2.900	2.900	2.900	2.177	3	0.793	2.21	0.00	5
G24000908609002031	118	0	0.5	4	118	171.583	9.506	4.602	1.865	110	1	0.02	4.57	308
G24004500108001036	5	0	6	0	6	7.498	7.498	7.496	1.714	8	0.059	7.94	0	8
G24004709517001093	1	0	0	0	0	1.505	1.505	1.477	0.874			0.00	0	6
G24001708502023009	1	0	0	0	1	1.576	0.317	0.111	0.000	1		0.00	0	2
G24004709517003008	2	0	0	1	1	3.474	3.403	1.904	0.000			0	0	0
G24003909305001057	6	0	2.5	2.5	5	11.908	11.699	5.867	1.150	8	0.134	4.83827	3.03	4
G24000908605012014	173	0	0	0	173	352.454	1.299	0.323	0.011	202		0	0.00	543
G24000908606002067	23	0	0	0	22	47.690	3.835	2.473	1.549	33	2	0	0.00	53
G24003708762004031	45	4	9	1	45	97.316	29.523	22.911	6.936	53	6.7	15.1736	0.13	103
G24000908607032085	6	0	1	1	6	13.178	3.334	1.590	0.471	12	0.089	2.11147	1.80	18
G24004109602011074	32	0	1	3	55	70.405	9.082	4.084	1.182	48		0.54762	3.03	61
G24003909305002065	3	0	4	0	4	6.742	6.742	6.664	0.661	5	0.206	4.79432	0.00	4
G24001909701003100	1	0	0	0	1	2.257	1.140	0.464	0.167	1		0	0.00	4
G24001109555003003	51	0	0	0	51	128.802	0.907	0.352	0.014	62		0	0.00	114
G24001500304004004	5	0	0	0	2	13.369	9.621	8.733	2.180	7	1.779	1.22075	0.66	6
G24001909707022093	2	0	0	3.5	11	5.659	3.743	0.006	0.000	14		0	5.07	2

Note: IFC001 is the Census variable for residential units.

Table A1: Housing Units Counted, Estimated Number of Buildings based on Footprint Data, and Area of Land by Elevation and Census Block: Maryland

		Counted Housing Units				Area of Block by Elevation (ha)				Buildings Footprint Data by Elevation				
GISJOIN	IFC001 (units)	<1m	1 to 2 m	2 to 3m	Total	Total Area	< 3m	<2m	< 1 m	Total	<1m	1m to 2m	2m to 3m	Popula-tion
G24001500305051007	1	0	0	0	1	2.889	0.281	0.000	0.000	1		0	0.00	3
G24000504524001123	2	0	0	0	2	6.183	2.530	0.000	0.000	12		0	6.00	2
G24003708752012037	160	0	0	0	160	682.987	0.282	0.010	0.000	270		0	0.00	431
G24004109608001066	1	0	0	0	0	4.511	4.511	3.035	0.642			0	0.00	1
G24003708761004012	4	0	0	0	4	18.894	5.483	1.347	0.161	5		0	0.00	9
G24003909305001161	1	1	0	0	1	4.854	4.854	4.854	4.854	3	3	0	0.00	4
G24002503024003025	1	0	0	0	1	5.297	0.186	0.011	0.000	6		0	0.00	3
G24004500108003050	41	0	10	9	48	219.551	55.750	26.136	8.069	68		1.87112	17.85	115
G24003708759011016	23	0	0	0	23	123.406	12.257	5.422	1.715	28		0	0.13	60
G24004109606001227	2	0	0	0	0	12.889	12.889	7.970	1.084	3	1.317	1.68293	0.00	2
G24004109602012088	2	8	13	0	21	13.099	11.923	4.354	0.945	3		0	0.01	3
G24004109608001005	12	0	0	2.5	15	81.105	31.059	15.344	6.316	18		0.29642	3.70	17
G24002909502005154	4	0	0	0	5	28.168	2.680	2.195	1.278	6		0	1.00	7
G24003909301021164	1	0	0	0	0	7.244	4.952	3.469	1.578	1	6E-04	0.02733	0.47	4
G24002909501001012	18	0	0	0	18	160.301	4.030	2.923	1.894	23		0	0.00	24
G24001909707023166	1	1	0	0	1	8.915	8.915	8.915	8.039	1	1	0	0.00	2
G24000908608013000	54	0	0	0	52	511.071	10.255	6.793	3.494	75		0	0.00	152
G24001909701003085	2	0	0	0	2	26.237	0.100	0.000	0.000	4		0	0.00	2
G24004109605012111	2	0	0	0.5	2	26.501	5.235	2.426	0.576	9		0.01	1.05	2
G24002909504003129	3	1	0.5	2.5	8	40.070	14.951	7.263	2.063	5	0.795	1.61	1.07	5
G24003909301011162	2	0	0	0	2	32.087	0.446	0.006	0.000	4		0.00	0.00	4
G24002909501004039	1	0	0	0	1	16.770	0.016	0.000	0.000			0	0.00	1
G24001909707022003	26	6.5	14	1.5	23	437.612	428.841	284.535	130.089	44	12.82	20.8601	6.35	37
G24004500108003112	15	0	0.5	2.5	17	291.586	188.319	87.101	29.398	29		2.10	9.96	32
G24001109552022088	8	0	0	0	5	165.948	13.385	7.535	2.474	7		0	0.00	5
G24003308008001002	4	0	0	0	4	171.081	0.066	0.004	0.000	22		0	0.00	12
G24001909709001200	6	1	2.5	1	5	269.415	268.880	267.487	252.142	20	11.74	6.74	1.09	5
Total Stratum 5	2195	69.5	221.5	273	2251	5529.42	1580.20	1018.96	554.75			0	0.00	5218

Note: IFC001 is the Census variable for residential units.

Table A1: Housing Units Counted, Estimated Number of Buildings based on Footprint Data, and Area of Land by Elevation and Census Block: Maryland

GISJOIN	IFC001 (units)	Counted Housing Units <1m	1 to 2 m	2 to 3m	Total	Area of Block by Elevation (ha) Total Area	< 3m	<2m	< 1 m	Buildings Footprint Data by Elevation Total	<1m	1m to 2m	2m to 3m	Population
G24001109556004037	0	0	0	0	0	48.473	5.501	3.012	0.155			0	0.00	0
G24003909301011236	0	0	0	0	0	22.888	1.279	0.743	0.060	3		0	0.00	0
G24003909305002026	0	0	0	0	0	19.573	19.573	19.562	0.908			0	0.00	0
G24000504301012033	0	0	0	0	0	15.406	2.280	1.699	1.129	4		0	0.00	0
G24001909709002226	0	0	0	0	0	8.515	8.515	8.515	8.477			0	0.00	0
G24001909708041141	0	0	0	0	0	5.426	5.426	5.426	5.340			0	0.00	0
G24000504202002014	0	0	0	0	0	4.135	0.188	0.119	0.060	5		0	0.00	0
G24051002604041022	0	0	0	0	0	4.076	0.059	0.011	<Null>	5		0	0.00	0
G24051002604041015	0	0	0	0	0	3.714	0.309	0.180	<Null>	6		0	0.00	0
G24000504524001082	0	0	0	0	0	2.948	0.386	<Null>	<Null>			0	0.00	0
G24004500101012128	0	0	0	0	0	2.627	0.147	<Null>	<Null>			0	0.00	0
G24003308040022014	0	0	0	0	0	2.485	1.373	0.452	0.116	1		0	0.00	0
G24000307502031032	0	0	0	0	0	2.322	0.168	0.030	<Null>	1		0	0.00	0
G24004500108002090	0	0	0	0	0	1.312	0.084	<Null>	<Null>			0	0.00	0
G24003508105002017	0	0	0	0	0	0.915	0.913	0.602	0.156			0	0.00	0
G24051000203002006	0	0	0	0	0	0.717	0.717	0.561	<Null>	2		1.37	0.60	0
G24003909301011058	0	0	0	0	0	0.664	0.023	<Null>	<Null>			0	0	0
G24001500301003197	0	0	0	0	0	0.659	0.082	<Null>	<Null>			0	0	0
G24002909505001034	0	0	0	0	0	0.639	0.639	0.639	0.507	1	0.516	0.48	0	0
G24000307013004021	0	0	0	0	0	0.411	0.368	0.064	<Null>			0	0	0
G24001909708041140	0	0	0	0	0	0.344	0.344	0.344	0.344			0	0	0
G24004109607003009	0	0	0	0	0	0.338	0.150	<Null>	<Null>			0	0	0
G24004709509001041	0	<Null>	0	0	0	0.309	0.309	0.309	0.309			0	0	0
G24004709517001095	0	0	0	0	0	0.307	0.307	0.307	0.120			0	0	0
Total Stratum 6	0	0	0	0	0	149.20	49.14	42.58	17.68			0	0	0

Note: IFC001 is the Census variable for residential units.

Table A1: Housing Units Counted, Estimated Number of Buildings based on Footprint Data, and Area of Land by Elevation and Census Block: Maryland

GISJOIN	IFC001 (units)	Counted Housing Units <1m	1 to 2 m	2 to 3m	Total	Area of Block by Elevation (ha) Total Area	< 3m	<2m	< 1 m	Buildings Footprint Data by Elevation Total	<1m	1m to 2m	2m to 3m	Popula-tion
G24051002606056112	0	0	0	0	0	0.221	0.090	<Null>	<Null>			0	0	0
G24000307063013010	0	0	0	0	0	0.209	0.208	0.199	0.137			0	0	0
G24003909301021162	0	0	0	0	0	0.203	0.203	0.014	0.003			0	0	0
G24003909305001025	0	0	0	0	0	0.202	0.202	0.202	0.202	2	2	0	0	0
G24001909708041143	0	0	0	0	0	0.177	0.177	0.177	0.099	1	0.59	0.41003	0	0
G24003708762004156	0	0	0	0	0	0.176	0.176	0.176	0.176			0	0	0
G24001909707023224	0	0	0	0	0	0.141	0.141	0.129	<Null>			0	0	0
G24051000302002041	0	0	0	0	0	0.133	0.115	<Null>	<Null>			0	0	0
G24003909302002032	1	1	0	0	1	0.128	0.128	0.128	0.128			0	0	1
G24003909305002042	1	0	1	0	1	0.125	0.125	0.125	<Null>	1		1	0	2
G24004500108003252	0	0	0	0	0	0.099	0.098	0.086	0.070			0	0	0
G24004109608001027	1	0	0	0	0	0.097	0.097	0.084	<Null>			0	0	2
G24001909708041095	0	0	0	0	0	0.097	0.097	0.097	0.097			0	0	0
G24000307309013039	0	0	0	0	0	0.089	0.089	0.055	<Null>			0	0	0
G24004109602014074	0	0	0	0	0	0.076	0.076	0.076	<Null>			0	0	0
G24003708753002056	0	0	0	0	0	0.071	0.071	0.070	0.023			0	0	0
G24004709517003074	0	0	0	0	0	0.070	0.070	0.070	0.037			0	0	0
G24001909708042011	0	0	0	0	0	0.069	0.069	0.069	<Null>			0	0	0
G24001708505002061	0	0	0	0	0	0.064	0.064	0.013	<Null>	1		0	1	0
G24051002201002014	0	0	0	0	0	0.063	0.063	0.063	<Null>			0	0	0
G24003909306003016	1	1	0	0	1	0.057	0.057	0.057	0.057			0	0	1
G24004500104001005	0	0	0	0	0	0.052	0.052	0.050	0.041			0	0	0
G24001909701003250	0	0	0	0	0	0.051	0.050	0.001	<Null>			0	0	0
G24003909302001038	0	0	0	0	0	0.051	0.051	0.051	0.051			0	0	0
G24001909708041108	0	0	0	0	0	0.012	0.012	0.012	0.012			0	0	0
G24001909701003230	0	0	0	0	0	0.002	0.002	0.002	0.002			0	0	0
Totals for Stratum 7	4	2	1	0	3	2.73	2.58	2.01	1.13					6

Note: IFC001 is the Census variable for residential units.

Table A2: Housing Units Counted, Estimated Number of Buildings based on Footprint Data, and Area of Land by Elevation and Census Block: New York, New Jersey, Pennsylvania, Delaware, District of Columbia, Virginia

				Housing Units Counted				Buildings from footprint data				Area of Block by Elevation (ha)					
GISJOIN	State	Location	IFC001	<1m	1m to 2 m	2m to 3m	Total	Entire Block	<1m	1m to 2m	2m to 3m	Area_of block	<1m	1m to 2m	2m to 3m	Pop	Density: units/ha
G34000100131023009	NJ	Ventnor	16	11	1		12	9	8.50	0.50	0	0.431	0.410	0.431	0.431	9	37.2
G34000100003003013	NJ	Atlantic City:	56	0	3		56	3	0.82	2.18	0	0.304	0.092	0.304	0.304	156	184.4
G34000100012003003	NJ	Atlantic City:	22	10.5	11.5		22	6	3.40	2.60	0	0.317	0.237	0.317	0.317	44	69.3
G34000100011002015	NJ	Atlantic City:	30	7.5	22.5		30	2	0.51	1.49	0	0.448	0.232	0.448	0.448	60	66.9
G34000100019001007	NJ	AC Oriental and Vermont	353	0	353		353	6	0	6.00	0	1.717	0.024	1.717	1.717	720	205.6
G34000900201021002	NJ	Ocean City, Central and 10th	28	0	23		23	12	0.04	11.96	0	0.798	0.050	0.798	0.798	43	35.1
G34000900202062075	NJ	Ocean City	29	0	12		12	12	0	12.00	0	0.743	0.016	0.740	0.743	3	39.0
G34000900213001028	NJ	North Wildwood`	52	25.5	8.5		34	19	15.35	3.65	0	1.477	1.376	1.477	1.477	43	35.2
G34000900213001055	NJ	North Wildwood`	89	1.5	17.5	0	19	16	0.71	15.29	0	1.439	0.245	1.439	1.439	8	61.8
G34000900214001026	NJ	Wildwood	43	20	1		21	22	21.36	0.64	0	1.175	1.103	1.175	1.175	32	36.6
G34000900214001039	NJ	Wildwood	40	0	40		40	15	0.03	14.97	0	0.960	0.134	0.960	0.960	17	41.7
G34001700022002006	NJ	Jersey City	83	7.5	72.5	0	80	5	1.15	3.85	0	1.140	0.226	1.140	1.140	119	72.8
G34002508084023010	NJ	Belmar Marina, Belmar	104	0	0	0	0	2	0	1.00	1	2.620	0.031	1.308	2.429	102	39.7
G34002508084011002	NJ	Avon by the sea	101	0	0	4	101	6	0	0	0.33	2.073	0.016	0.017	0.282	117	48.7
G34002907280005035	NJ	Seaside Heights	66	28	24	0	52	34	18.44	15.56	0	1.701	0.846	1.701	1.701	65	38.8
G34002907225003081	NJ	LaVallette	18	8	10		18	13	4.82	8.18	0	0.373	0.209	0.373	0.373	2	48.3
G34002907280006019	NJ	Seaside Heights	78	1	65.5	11.5	78	29	0.50	27.04	1.46	1.599	0.017	1.487	1.599	44	48.8
G34003300203002009	NJ	Penns Grove	30	17	14	0	31	1	0	1.00	0	0.770	0.446	0.770	0.770	78	39.0
G10000500507052038	DE	Millsboro	26	0	9	7.5	26	5	0.09	2.91	1.00	0.550	0.025	0.194	0.352	0	47.3
G10000500512052047	DE	Fenwick Island	120	0	0	0	0	1	0	1.00	0	0.941	0.531	0.941	0.941	38	127.5
G36000500274014025	NY	Bronx	60				0	12	2.00	1.83	1.43	0.994	0.128	0.223	0.300	143	60.4
G36004701010001000	NY	Brooklyn	74	0	0	1	74	19	0	0.27	1.02	2.104	0.011	0.289	0.458	221	35.2
G36004700956002002	NY	Brooklyn	154	0	24.5	15.5	48	15	0.02	8.19	4.57	1.979	0.011	1.081	1.680	498	77.8
G36004700610043000	NY	Brooklyn	230	180	25	2	207	4	0	2.43	1.36	1.616	0.726	1.167	1.412	384	142.4
G36004700348001019	NY	Brooklyn	164	164	0	0	164	2	2.00	0	0	2.394	2.228	2.394	2.394	379	68.5
G36005904130013019	NY	Oceanside, Nassau County	223	13	208.5	1.5	223	26	8.93	15.66	1.41	5.475	2.774	5.431	5.475	351	40.7

Note: IFC001 is the Census variable for residential units.

Table A2: Housing Units Counted, Estimated Number of Buildings based on Footprint Data, and Area of Land by Elevation and Census Block: New York, New Jersey, Pennsylvania, Delaware, District of Columbia, Virginia

				Housing Units Counted				Buildings from footprint data				Area of Block by Elevation (ha)					
GISJOIN	State	Location	IFC001	<1m	1m to 2 m	2m to 3m	Total	Entire Block	<1m	1m to 2m	2m to 3m	Area_of block	<1m	1m to 2m	2m to 3m	Pop	Density: units/ha
G36005904164021006	NY	Long Beach	50	21	28	0	49	47	19.22	27.78	0.00	1.225	0.737	1.225	1.225	103	40.8
G36006100210004005	NY	Manhattan	726	0	398	328	726	7	0	3.79	3.21	3.508	0.146	1.976	3.508	1917	206.9
G36006100152001001	NY	Manhattan	215	47.8	167.2	0	215	1	0.25	0.75	0	0.548	0.161	0.486	0.548	481	392.1
G36006100162003001	NY	Manhattan	398	217.1	180.9	0	398	4	2.62	1.38	0	2.084	1.046	1.952	2.084	1467	190.9
G36006100062001000	NY	Manhattan	289					2	0	2.00	0	4.932	1.455	3.255	3.803	736	58.6
G36006100024002000	NY	Manhattan, East Village	741					12	8.35	3.65	0	4.848	3.244	4.848	4.848	1943	152.8
G36008100942011008	NY	Queens	72	36	36		72	1	0.51	0.49	0	0.795	0.505	0.795	0.795	361	90.5
G36008500112022006	NY	Staten Island	44	0	44	0	44	19	0	19.00	0	1.189	0.011	1.189	1.189	118	37.0
G36008500112023002	NY	Staten Island	38	14.5	15.5	0	30	17	5.57	11.43	0	0.881	0.461	0.881	0.881	118	43.1
G36010301595101175	NY	Fire Island, Suffolk Co	25	0	5	20	25	5	1	0.55	3.45	0.458	0.027	0.122	0.436	0	54.6
G36011900057012007	NY	New Rochelle	359	21	228.2	35.4	359	18	6.93	3.25	3.38	8.215	4.157	5.401	6.065	752	43.7
G51071000038002019	VA	Norfolk	14	0	0	0	0		0	0.00	0	0.355	0.282	0.355	0.355	24	39.4
G51081000438002029	VA	Virginia Beach	48	0	31	1	32	3	0.02	2.92	0.06	0.587	0.145	0.584	0.589	64	81.8
Total Stratum A-1-b-i			5308	851.9	2080	427	3674	432	133.13	237.20	23.66	65.765	24.522	51.392	57.443	11760	80.7

Note: IFC001 is the Census variable for residential units.

Table A2: Housing Units Counted, Estimated Number of Buildings based on Footprint Data, and Area of Land by Elevation and Census Block: New York, New Jersey, Pennsylvania, Delaware, District of Columbia, Virginia

				Housing Units Counted				Buildings from footprint data				Area of Block by Elevation (ha)					
GISJOIN	State	Location	IFC001	<1m	1m to 2 m	2m to 3m	Total	Entire Block	<1m	1m to 2m	2m to 3m	Area_of block	<1m	1m to 2m	2m to 3m	Pop	Density: units/ha
G34000100014002014	NJ	Atlantic City	27	25.5	1.5	0	27	20	18.91	1.09	0	0.995	0.918	0.995	0.995	43	27.1
G34000100014001041	NJ	Atlantic City	12	0	0	0	0		0	0	0	0.504	0.175	0.505	0.505	16	23.8
G34000100135002016	NJ	Longport	20	11	8	0	19	19	12.13	6.87	0	0.858	0.633	0.858	0.858	16	23.3
G34000100101011052	NJ	Brigantine	19	18.5	0.5	0	19	18	17.45	0.55	0	1.389	1.371	1.389	1.389	31	13.7
G34000100001002023	NJ	Atlantic City	18	4.5	10.5	0	15	15	9.96	5.04	0	0.891	0.687	0.891	0.891	32	20.2
G34000300361002011	NJ	South Hackensack	35	4	19	0	23	25	4.16	20.84	0	2.391	0.809	2.391	2.391	90	14.6
G34000507012011013	NJ	Burlington	5	0	7	0	7	5	0	5.00	0	0.441	0.070	0.441	0.441	18	11.3
G34000900218032023	NJ	Villas	22	9	13	0	22	24	7.27	16.73	0	1.569	0.560	1.569	1.569	32	14.0
G34001300068002021	NJ	Newark	16	0	4	1	5	5	0	3.77	1.23	1.860	0.032	1.297	1.860	13	8.6
G34001505004005011	NJ	Paulsboro	6	0	5	0	5	8	0	7.92	0.08	0.566	0.028	0.564	0.570	15	10.6
G34002508006014012	NJ	Keansbury	32	20.5	10.5	0	31	29	19.68	9.32	0	1.741	1.402	1.741	1.741	87	18.4
G34002508041001005	NJ	Monmouth Beach	89	0	41	2	43	14	0.39	12.72	0.89	4.224	1.232	4.122	4.221	92	21.1
G34002508018005024	NJ	Union Beach, NJ	9	0.5	3.5	3	7	9	0.27	3.78	4.95	0.897	0.111	0.551	0.897	36	10.0
G34002907234001040	NJ	Toms River	39	12.5	23.5	0	36	36	13.90	22.10	0	2.848	1.198	2.843	2.843	54	13.7
G34002907321041003	NJ	Forked River	22	16	6	0	22	17	15.10	1.90	0	1.788	1.534	1.792	1.792	28	12.3
G34002907270022060	NJ	Seaside Heights	8	7	1		8	7	6.48	0.52	0	0.508	0.486	0.508	0.508	9	15.7
G34002907270012003	NJ	Lavalette	20	6.5	12.5		19	21	7.77	13.23	0	1.232	0.699	1.240	1.240	6	16.2
G34002907114001016	NJ	Point Pleasant	26	0	14	10.5	26	28	0	14.98	11.95	2.388	0.021	1.778	2.317	53	10.9
G34002907380022024	NJ	Holgate LBT	30	2.5	26.5	0	29	28	3.08	24.92	0	1.584	0.555	1.584	1.584	5	18.9
G34002907310022023	NJ	Bayville	41		41.5	1.5	43	5	0	4.42	0.58	4.959	0.654	4.868	4.941	76	8.3
G34002907380011039	NJ	Spray Beach LBT	14	10	4	0	14	17	13.14	3.86	0	1.136	0.842	1.136	1.136	0	12.3
G34002907380011041	NJ	Spray Beach LBT	16	9	6	0	15	13	7.49	5.51	0	1.331	1.012	1.331	1.331	15	12.0
G34002907260001039	NJ	Island Heights	15	0	0	0	10	14	0.00	0.00	0	1.268	0.091	0.210	0.299	18	11.8
G34002907234002015	NJ	Toms River	29	26.5	1.5	0	28	26	25.08	0.92	0	2.168	2.115	2.166	2.166	42	13.4
G10000300166082051	DE	Near Silver Run Wildlife Area	14	8.5	14.5	0	23	26	9.58	16.42	0	1.377	0.597	1.376	1.376	12	10.2
G36000500516001013	NY	Bronx	30	9	4	3	20	13	4.01	2.01	2.48	1.031	0.397	0.551	0.715	60	29.1
G36005905219023007	NY	East Massapequa	17	0	8.5	7.5	16	17	0.26	9.25	7.49	2.085	0.224	1.393	2.085	47	8.2
G36005904113023001	NY	Woodmere	30	3	15	11.5	30	31	4.24	14.17	12.09	3.038	0.923	2.344	3.022	107	9.9
G36008100972034003	NY	Queens	96	60	36		96	2	1.15	0.85	0	3.973	2.531	3.973	3.973	224	24.2

Note: IFC001 is the Census variable for residential units.

Table A2: Housing Units Counted, Estimated Number of Buildings based on Footprint Data, and Area of Land by Elevation and Census Block: New York, New Jersey, Pennsylvania, Delaware, District of Columbia, Virginia

				Housing Units Counted				Buildings from footprint data				Area of Block by Elevation (ha)					
GISJOIN	State	Location	IFC001	<1m	1m to 2 m	2m to 3m	Total	Entire Block	<1m	1m to 2m	2m to 3m	Area_of block	<1m	1m to 2m	2m to 3m	Pop	Density: units/ha
G36010301905023075	NY	West Hampton	6	6	0	0	6	7	7.00	0	0	0.366	0.355	0.355	0.355	1	16.4
G36010301470041224	NY	Ocean Beach	12	7	5.5	1.5	14	13	6.07	4.90	1.75	0.934	0.494	0.779	0.892	5	12.8
G36010301470041124	NY	Ocean Beach	14	3.5	5	3.5	12	12	4.94	3.00	4.05	1.196	0.391	0.693	1.078	0	11.7
G36010301104011004	NY	Huntington	19			0.5	16	17	0.11	0	1.39	1.545	0.294	0.383	0.510	54	12.3
G36010301238021029	NY	Lindenhurst	22	2	19.5	0.5	22	21	0.80	19.94	0.25	1.496	0.355	1.491	1.496	54	14.7
G51065000113002050	Va	Hampton	10		7	3	10	9	0.00	5.97	3.03	0.779	0.045	0.589	0.779	24	12.8
G51071000061001009	Va	Norfolk	25	0	3.5	22.5	26	27	0.00	3.68	23.32	2.299	0.080	0.678	2.298	60	10.9
G51081000418021012	Va	Virginia Beach	13	6.5	6.5	0	13	13	6.48	6.52	0	0.990	0.333	0.990	0.990	25	13.1
G51081000418021014	Va	Virginia Beach	4	0	2.5	1.5	4	5	0.00	3.85	1.15	0.360	0.040	0.310	0.360	5	11.1
G51081000440032017	VA	Virginia Beach	21		3.5	12.5	19	19	0.00	3.64	12.34	1.532	0.018	0.474	1.338	41	13.7
G51005904152004036	VA	Fairfax Co	12	11	1		12	12	11.76	0.24	0	1.213	1.180	1.213	1.213	20	9.9
Total A-1-bii			915	227.5	323.5	84.5	670	647	238.66	280.45	89.02	63.751	25.493	54.363	60.965	1566	14.4

Note: IFC001 is the Census variable for residential units.

Table A2: Housing Units Counted, Estimated Number of Buildings based on Footprint Data, and Area of Land by Elevation and Census Block: New York, New Jersey, Pennsylvania, Delaware, District of Columbia, Virginia

				Housing Units Counted				Buildings from footprint data				Area of Block by Elevation (ha)					
GISJOIN	State	Location	IFC001	<1m	1m to 2 m	2m to 3m	Total	Entire Block	<1m	1m to 2m	2m to 3m	Area_of block	<1m	1m to 2m	2m to 3m	Pop	Density: units/ha
G34000300120021009	NJ	E. Rutherford NJ	78		0	3	6		0	0	0	0.557		0.054	0.145	138	140.1
G34000706020004033	NJ	Camden	19		0.5	17	18	5	0	0.02	4.88	0.525		0.037	0.519	60	36.2
G34000900201013024	NJ	Ocean City	50	0	41	7	48	24	0	22.09	1.91	1.424		1.356	1.424	32	35.1
G34001300075012015	NJ	Newark	59	0	3	57	60	2	0	0.06	1.94	0.619		0.105	0.619	120	95.3
G34001700047001006	NJ	Jersey City	64	0	19	20	47	7	0	2.30	3.65	1.190		0.309	0.953	146	53.8
G34001700054001012	NJ	Jersey City	32	0	0	29	32	2	0	0	1.87	0.654		0.017	0.630	78	49.0
G34002300071031012	NJ	Sayreville	44	0	0	44	44	3	0	0	3.00	0.996		0.013	0.996	104	44.2
G34003900308022013	NJ	Elizabeth	107	0	0.5	7	52	36	0	0.59	5.44	3.040		0.092	0.392	349	35.2
G10000500507052009	DE	Millsbor	23	0	5	5	18	4	0	0	0	0.447	0.002	0.146	0.277	0	51.4
G36000500144005000	NY	Bronx	418	0	0	27	418	11	0	0	0.81	5.466		0.366	1.084	1170	76.5
G36004701024001001	NY	Brooklyn	119	0	0.5	119	119	32	0	31.87	0.13	2.366		2.326	2.366	404	50.3
G36004700610031002	NY	Brooklyn	34	0	2	36	38	15	0	14.19	0.81	0.497		0.479	0.497	75	68.4
G36004701078002011	NY	Brooklyn	400	0	120	280	400	4	0	1.27	2.73	3.174		1.090	3.174	663	126.0
G36004700974001002	NY	Brooklyn	136	0	0	0	136	15	0	14.65	0	1.746		1.654	1.739	451	77.9
G36004700408003003	NY	Brooklyn	81	0	0	81	81	16	0	3.00	13.00	1.162		0.514	1.165	186	69.7
G36006100015013010	NY	Manhattan	23	0	23	0	23	1	0	1.00	0	0.371		0.290	0.364	44	62.0
G36006100192001005	NY	Manhattan	151	0	10	141	151	4	0	0.06	3.94	1.808		0.125	1.808	364	83.5
G36006100033003003	NY	Manhattan	93	0	13.5	76.5	90	1	0	0.23	0.63	1.114		0.411	0.908	172	83.4
G36008100837001002	NY	Queens	61	0	0	19.5	32	10	0	0.00	5.74	1.037		0.078	0.666	179	58.8
G51074002109002030	VA	Portsmouth	140	0	4	4	8	5	0	3.54	1.46	1.160	0.005	0.911	1.160	171	120.7
Total A-1-c-i			2132	0	242	973	1821	197	0.00	94.88	52.30	29.354	0.008	10.373	20.886	4906	72.6

Note: IFC001 is the Census variable for residential units.

Table A2: Housing Units Counted, Estimated Number of Buildings based on Footprint Data, and Area of Land by Elevation and Census Block: New York, New Jersey, Pennsylvania, Delaware, District of Columbia, Virginia

				Housing Units Counted				Buildings from footprint data				Area of Block by Elevation (ha)					
GISJOIN	State	Location	IFC001	<1m	1m to 2 m	2m to 3m	Total	Entire Block	<1m	1m to 2m	2m to 3m	Area_of block	<1m	1m to 2m	2m to 3m	Pop	Density: units/ha
G42010100160006000		Philadelphia	12	0	0	10	14	5	0.05	0.29	2.75	0.536	0.006	0.059	0.337	36	22.4
G34000100004002002	NJ	Atlantic City	33	0	33	0	33	3	0	2.93	0.07	1.239		1.209	1.239	89	26.6
G34000300280012008	NJ	Leonia	7	0	0	0	7	7	0	0	0.06	0.696		0.010	0.138	24	10.1
G34000300234022011		Hackensack	33	0	0	0	33	4	0	0.27	0.34	0.978		0.204	0.298	82	33.7
G34000507026032007		Up Ronconcas Creek, near Mt Holly and Lumberton	9	0	9	0	9	5	0	0.06	4.94	0.450		0.134	0.450	15	20.0
G34000706016003001	NJ	Camden	24	0	20.5	12.5	33	4	0	3.25	0.75	0.801		0.683	0.801	58	30.0
G34000900216001055	NJ	Wildwood Crest	7	0	2.5	4.5	7	6	0	2.30	3.70	0.687		0.360	0.687	12	10.2
G34000900209021052	NJ	Avalon	19	0	18.5	0.5	19	21	0	20.76	0.24	1.539		1.522	1.539	0	12.3
G34002907224012004		Toms River, NJ	11	0	4.5	6.5	11	11	0	4.71	6.29	1.181		0.596	1.181	30	9.3
G34002907321014026	NJ	Lanoka Harbor	12	0	3.5	7.5	12	12	0	3.17	8.38	1.480		0.690	1.444	29	8.1
G34002907101003045	NJ	Point Pleasant	21	0	13.5	4.5	18	21	0	15.25	5.75	1.637		1.348	1.637	36	12.8
G10000300164043012	DE	Delaware City	8	0	6	5	11	10	0	6.50	3.50	0.622	0.008	0.328	0.623	22	12.9
G36005904116003033	NY	Lawrence	9	0	0.5	2	8	8	0	0.31	2.12	0.821		0.170	0.348	48	11.0
G36005904161001018	NY	Seaford	7	0	2.5	4.5	7	8	0	4.35	3.65	0.650	0.001	0.507	0.650	15	10.8
G36005904143034013	NY	Freeport	14	0	1.5	8.5	14	15	0	1.46	9.78	1.413		0.289	1.134	49	9.9
G36008500070004005	NY	Staten Island	44	0	7	22	29	31	0	10.00	21.00	1.510		0.482	1.510	103	29.1
G36011900103001004	NY	Hastings on Hudson	281	0	0	0	281	51	0	0	0	15.743		1.162	2.155	434	17.8
G51065000115001030	VA	Hampton	8	0	0	7	9	10	0	0	7.66	0.894		0.034	0.789	16	8.9
G51071000029004007	VA	Norfolk	28	0	4	25	29	17	0	1.51	15.49	1.338		0.380	1.338	68	20.9
G51074002109001040	VA	Portsmouth	41	0	22.5	15.5	38	22	0	15.57	6.43	4.173		3.437	4.173	71	9.8
Total A-1-c-ii			628					271	0.05	92.68	102.90	38.388	0.014	13.608	22.470	1237	16.4

Note: IFC001 is the Census variable for residential units.

Table A2: Housing Units Counted, Estimated Number of Buildings based on Footprint Data, and Area of Land by Elevation and Census Block: New York, New Jersey, Pennsylvania, Delaware, District of Columbia, Virginia

				Housing Units Counted				Buildings from footprint data				Area of Block by Elevation (ha)					
GISJOIN	State	Location	IFC001	<1m	1m to 2 m	2m to 3m	Total	Entire Block	<1m	1m to 2m	2m to 3m	Area_of block	<1m	1m to 2m	2m to 3m	Pop	Density: units/ha
G42010100027022003	PA	Philadelphia	54	0	0	7.5	55	4	0	0	0.20	0.758		0.002	0.146	89	71.2
G42010100042023008	PA	Philadelphia	57	0	0	28.5	62	4	0	0	2.46	0.714		0.005	0.457	153	79.9
G34001300070001001	NJ	Newark	106	0	0	33	101	8	0	0	5.24	1.139		0.002	0.765	259	93.1
G36004700568001000	NY	Brooklyn	203	0	0	75	83	4	0	0.02	3.75	2.096		0.006	1.785	381	96.8
G36010301246024007	NY	Babylon NY	162	0	0	25	151	28	0	0	11.15	3.532		0.006	1.385	257	45.9
Total A-1-d-i			582					48	0	0.02	22.80	8.239	0.000	0.021	4.538	1139	70.6
G34002300076003007	NJ	South Amboy	34	0	0	0	34	13	0	0	0.00	1.873		0.001	0.299	90	18.2
G34003900353005013	NJ	Linden NJ	17	0	0	0	17	3	0	0	0.00	0.489		0.004	0.020	41	34.8
G34003900357002015	NJ	Rahway	33	0	0	0	33	30	0	0	0.00	3.292		0.002	0.006		10.0
G36005904117003022	NY	Hewlett, NY	35	0	0	5.5	22	28	0	0	5.30	1.673		0.003	0.324	89	20.9
G11000100105001010	DC	3RD AND k, sw	49	0	0	11	50	7	0	0	1.20	1.513		0.005	0.556	210	32.4
Total A-1-d-ii			168	0	0	17	156	81	0	0	6.50	8.841		0.015	1.205	430	19.0
G34000100115003002	NJ	Mays Landing and Lake Lenape Western shore	4	0	0	0	5	4		0.00	0.00	72.427	0.013	0.022	3.205	0.01	0.1
G34000507029133001	NJ	Mt. Laurel	7	0	0	0	8	7		0.00	0.00	29.657	0.321	1.078	1.781	0.382	0.2
G34002508094002040	NJ	Brielle	16	0	2	16.5	19	16		0.00	5.17	4.327	0.450	1.799	4.299	0.524	3.7
G34003300201001001	NJ	Pedricktown	2	0	2	0	2	2		0.00	3.00	4.653	1.656	4.289	4.447	2.229	0.4
G36005904122002002	NY	East Rockaway	3	0	2	0	2	3		0.07	3.93	1.628	0.288	1.628	1.628	0.282	1.8
G36005905214003021	NY	S. Oyster Bay	22	0	8	14	22	22		0.00	7.46	3.030	0.099	2.090	3.024	0.095	7.3
G36010301904031022	NY	Riverhead	1	0	0	0	0	1		0.00	0.00	67.010	49.248	61.056	64.367	10.61	0.0
G36010301594062048	NY	Brookhaven	12	0	0	1	12	12		0.00	0.00	2.464	0.048	0.099	0.169	0.069	4.9
G51055000210102002	VA	Chesapeake	117	0	0	95	109	117		0.00	0.11	20.537	0.388	1.974	17.125	0.284	5.7
G51000100904004164	VA	Island near Wachapreague	1	0	0	0	0	1	2.69	6.89	0.11	1499.7	1472.0	1499.5	1499.7	25.49	0.0
G51000100902003178	VA	Assawoman	1	0	0.5	0.5	1	1		0.00	0.58	1.625	1.201	1.417	1.618	0.202	0.6

Note: IFC001 is the Census variable for residential units.

Table A2: Housing Units Counted, Estimated Number of Buildings based on Footprint Data, and Area of Land by Elevation and Census Block: New York, New Jersey, Pennsylvania, Delaware, District of Columbia, Virginia

				Housing Units Counted				Buildings from footprint data				Area of Block by Elevation (ha)					
GISJOIN	State	Location	IFC001	<1m	1m to 2 m	2m to 3m	Total	Entire Block	<1m	1m to 2m	2m to 3m	Area_of block	<1m	1m to 2m	2m to 3m	Pop	Density: units/ha
G51013109301004124	VA	Hare Valley	1	0	0	0	1	1		0.00	0.00	34.047	3.303	6.254	8.171	0.304	0.0
G51013300201002141	VA	Lottsburg	1	0	1	0	1	1		0.00	1.00	0.966	0.201	0.510	0.955	0.069	1.0
G51013300203002129	VA	Reedville	18	0	0	7.5	17	18		0.00	0.00	32.130	4.145	6.993	19.268	2.069	0.6
G51017900101072007	VA	Stafford Co	219	0	0	9.5	220	219		0.00	0.63	94.153	41.151	43.833	47.347	1.586	2.3
Total A-2-b-i			425	0	15.5	####	419.0	425	2.69	6.96	21.99	1868.4	1574.5	1632.6	1677.1	44.21	0.2
GISJOIN	Sta	where	IFC001	<1m	1 to 2 r	2 to 3	Total	Housii	Build b	Build <1	Building	Area_of bl	Area belc	Area bel	Area below_3m	Area below 1m	
G36010301698004001	NY	Suffolk	30	0.5	10.5	11	27	30		0.57	10.35	4.351	0.916	2.051	3.908		6.9
G36005905220002012	NY	Nassau	5	3.5	1.5	0	5	5		2.83	1.17	0.771	0.706	0.774	0.774		6.5
G36008101483004011	NY	Queens	27	2.0	2	3	24	27	0.53	1.49	2.91	4.760	1.142	1.376	1.738		5.7
G34000507012013007	NJ	Burlington Co	17	2.5	7.5	4	14	0		0.00	1.11	2.712	0.679	2.286	2.710		6.3
G34001100101013081	NJ	Cumberland Co	13	0.0	10	0	10	13		1.00	17.80	9.816	2.259	9.019	9.781		1.3
G51007301002012063	VA	Gloucester Co	4	0.0	0	0	4	4	2.00	2.00	0.07	8.843	0.626	0.830	1.199		0.5
G34001100101013073	NJ	Cumberland Co	4	4.0	0	0	4	4		8.01	1.99	22.100	21.567	22.120	22.125		0.2
Total A-2-b-ii			79	12.5	31.5	18.0	88.0	79	2.53	15.90	35.39	53.354	27.894	38.455	42.234	0	1.5
G42001701007001014	PA	Bristol	3	0	0	0	3	20	0	0.05	1.64	4.460		0.185	0.991	3	0.7
G34002508038002009	NJ	Belford	36	0	0	0	36	49	0	0	0	48.276			0.010	134	0.7
G10000300139043008	DE	Near Delaware House	123	0	0	0	123	106	0	0	0	48.468		0.410	1.826	315	2.5
G10000500501041012	DE	Milford	5	0	0	0	5	5	0	0	0	1.637	0.006	0.022	0.038	10	3.1
G36010301702023027	NY	Water Mill	13	0	0	0	2	7	0	0	0	4.523		0.355	0.406	16	2.9
G51055000212001007	VA	Chesapeake	28	0	0	0	28	71	0	0	0	156.025		0.099	1.927	69	0.2
G51065000103064004	VA	Hampton	9	0	0.5	8.5	9	10	0	0.27	9.73	1.463		0.432	1.463	15	6.2
G51070000316011029	VA	Newport News	234	0	0	0	234	233	0	0	0	30.469		0.006	0.389	668	7.7
G51071000014003021	VA	Norfolk	16	0	0	2.5	16	16	0	0	2.29	3.169			0.972	39	5.0
G51074002128011000	VA	Portsmouth	30	0	0	13	32	27	0	0	2.34	3.843		0	1.348	43	7.8
Total A-2-c			497	0	0.5	24	488	544	0	0.32	16.00	302.334	0.006	1.516	9.371	1312	1.6

Note: IFC001 is the Census variable for residential units.

Table A2: Housing Units Counted, Estimated Number of Buildings based on Footprint Data, and Area of Land by Elevation and Census Block: New York, New Jersey, Pennsylvania, Delaware, District of Columbia, Virginia

				Housing Units Counted				Buildings from footprint data				Area of Block by Elevation (ha)					
GISJOIN	State	Location	IFC001	<1m	1m to 2 m	2m to 3m	Total	Entire Block	<1m	1m to 2m	2m to 3m	Area_of block	<1m	1m to 2m	2m to 3m	Pop	Density: units/ha
G34000100135002056	NJ	Atlantic CO	7					4	4.00	0	0	0.161	0.161	0.161	0.161	2	43.6
G34002508090001012	NJ	Monmouth Co	2				0		0	0	0	0.164			0.125	2	12.2
G34003300215001064	NJ	Salem Co	1	0.0	1	0	1	1	0	1.00	0	0.241		0.242	0.243	5	4.2
G10000500513062010	DE	Sussex Co	6				0		0	0	0	0.145		0.145	0.145	2	41.3
G36011900071001006	NY	Westchester Co	1		0.25	0.25	0.5		0	0	0	0.182		0.023	0.150	4	5.5
G51071000017001039	VA	Norfolk	2				0		0	0	0	0.151			0.146	6	13.2
G51071000047002015	VA	Norfolk	1				0		0	0	0	0.083	0.006	0.009	0.083	3	12.1
G51081000454121043	VA	Virginia Beach	1	3.5	1.5		5	3	2.08	0.92	0	0.170	0.119	0.170	0.170	1	5.9
G51081000446004021	VA	Virginia Beach	1				0		0	0	0	0.154			0.144	4	6.5
G51000100906001092	VA	Accomac Co	1	2.5	0	0	2.5	5	4.39	0.61	0	0.149	0.149	0.149	0.149	2	6.7
Total B-2			23	6.0	2.8	0.3	9.0	13	10.47	2.53	0	1.600	0.435	0.898	1.515	31	14.4

Note: IFC001 is the Census variable for residential units.

Table A3: Housing Units Counted, Estimated Number of Buildings based on Footprint Data, and Area of Land by Elevation and Census Block: Blocks with at least 0.5 ha of dry land below MHHW along the Delaware River

				Housing Units Counted					Buildings from footprint data					Area of Block by Elevation (ha)						
GISJOIN	State	where	IFC001	<mhhw	mhhw to 1m	1 to 2m	2 to 3m	Total	Entire Block	<0m	<1m	1m to 2m	2m to 3m	Entire Block	< 3m	< 2m	<1m	<MHHW	Pop	Density units/ha
G34001505022001016	NJ	Racoon Creek	1					1	8		0.0	0.0	0.0	76.106	8.330	5.512	2.840	0.654	0	0.01
G34001505024002038	NJ	Logan Township	6	0	3	2.0		5	6		3.0	3.0	0.0	12.366	12.316	12.076	8.549	3.131	12	0.49
G34001505024001013	NJ	Bridgeport	3			1.5	0.5	3	2		0.0	1.0	0.3	235.703	34.296	20.241	3.566	0.647	6	0.01
G34001505004004001	NJ	Paulsboro	37	0	0.5	26.5	7	34	66	0.0	6.0	54.2	5.8	32.414	32.131	31.264	18.461	1.097	75	1.14
G34001505005004032	NJ	Gibbstown	32	12.5	16.5	4.5	0.5	34	34	12.4	29.0	4.7	0.3	4.805	4.805	4.774	4.419	2.616	70	6.66
G34001505005001016	NJ	Greenwich township	23	0.5	5.5	2.5	6.5	27	36	1.5	8.4	3.4	10.6	7.336	4.950	2.440	1.580	0.744	65	3.14
G34001505002051014	NJ	West Deptford	34	0	0	0.5	2	33	40		0.0	0.0	2.2	28.164	7.024	4.633	2.670	1.269	94	1.21
G34001505006003006	NJ	East Grenwich Township	16	0	0	0.0	0	16	46		0.0	0.0	0.0	114.742	7.546	5.469	3.445	1.092	135	0.14
G34003300221001011	NJ	Salem	96	18.0	75.5	2.5	0.0	96.0	43	14.3	39.9	3.1	0.0	36.346	26.523	24.109	20.526	15.510	160	2.64
G34003300213002012	NJ	Mannington Township	11	0	0.0	3	2	9	24		4.6	7.4	4.2	186.038	146.335	111.520	63.427	20.785	32	0.06
G34003300206002147	NJ	Carney's Point Township	11	0	0.5	4	6.5	11	11		0.3	5.6	4.1	11.797	10.620	7.722	3.461	0.716	34	0.93
G34003300206002042	NJ	Penns Grove	3	0	0	0.0	2	2	8		1.6	2.3	4.2	42.783	42.690	38.683	25.759	7.186	3	0.07
G34003300216003017	NJ	Pennsville Township	14	1	9	0.0	0	10	11	0.8	11.0	0.0	0.0	1.911	1.911	1.911	1.911	0.740	30	7.32
G34003300222012022	NJ	Harmersville	19		0	1	2	22	35		0.0	1.0	4.3	101.856	42.644	22.409	6.966	3.014	46	0.19
G34003300222011059	NJ	Lower Alloways Creek, NJ	18	0	1	5.0	5	17	27		3.1	8.3	8.2	58.346	55.996	47.792	27.964	7.400	28	0.31
G34003300219001001	NJ	Salem	8		1	1.0	6.5	8	9		0.1	1.9	7.0	1.892	1.884	1.409	0.969	0.669	23	4.23
G10000300168041068	DE	Townsend South of Odessa	28	0	0	0.0	0	8	45		0.0	0.0	0.0	152.133	7.615	5.666	3.652	1.619	63	0.18
G10000300164041050	DE	Delaware City	1	0.5	4			4	7	1.0	6.0	0.0	0.9	30.528	30.499	28.876	21.189	1.641	2	0.03
G10000300161002005	DE		34	0.5	11	21.5	0.5	33	17	0.3	5.8	11.1	0.1	2.848	2.843	2.815	2.055	1.360	64	11.94
			398						483	30.3	####	109.2	56.3	1180.9	523.6	418.0	249.2		945	

Note: IFC001 is the Census variable for residential units.
