# Supplementary material for: Population in floodplains or close to sea level increased in US but declined in some counties—especially among Black residents
Source: Environ Res Lett. Author manuscript; Available in PMC 2025 Mar 14. (PMC11908447; doi:10.1088/1748-9326/acadf5)
Supplement: Table of Supplemental Contents [file NIHMS1876714-supplement-Table_of_Supplemental_Contents.docx]

| erlacdf5supp1.pdf | Preliminary Test of Method | Source for tables 2 and 3 |
| --- | --- | --- |
| erlacdf5supp2.pdf | Supplemental Figures |  |
| erlacdf5supp3.pdf | Supplemental Methods | Sections 1 to 4 elaborate method section. Other sections elaborate on uncertainty, caveats, measures of disproportionality, and overlays with redlining and planning data. |
| erlacdf5supp4.pdf | Tables in Portrait Format | Tables S1–S7, S9E–S10, S13, and S14 |
| erlacdf5supp5.pdf | Tables in Landscape Format | Tables S11A–S12D |
| erlacdf5supp6.zip | State and County Results |  |
