# Supplementary material for: Population in floodplains or close to sea level increased in US but declined in some counties—especially among Black residents
Source: Environ Res Lett. Author manuscript; Available in PMC 2025 Mar 14. (PMC11908447; doi:10.1088/1748-9326/acadf5)
Supplement: State and County Results [file NIHMS1876714-supplement-State_and_County_Results.zip › state_and_county/county_results/Readme.pdf]

This directory has population by county for the following categories:

File names	Data included—by state by county
population_below_1m_shows_combined_effect_of_sea_rise_and_migration.xlsx<br>population_below_3m_shows_combined_effect_of_sea_rise_and_migration.xlsx<br>Black_population_below_1m_shows_combined_effect_of_sea_rise_and_migration.xlsx<br>Hispanic_population_below_1m_shows_combined_effect_of_sea_rise_and_migration	Population less than 1 or 3 meters above sea level for the years 1990, 2000, 2010, and 2020, for all, Black, or Hispanic Residents.
population_shows_net_migration_into_or_out_of_land_below_1m.xlsx<br>population_shows_net_migration_into_or_out_of_land_below_3m.xlsx<br>Black_population_shows_net_migration_into_or_out_of_land_below_1m.xlsx<br>Hispanic_population_shows_net_migration_into_or_out_of_land_below_1m.xlsx	Population for the years 1990, 2000, 2010, and 2020 , for all, Black, or Hispanic Residents, less than 1 or 3 meters above the sea level of 2020. Because the measure of sea level is fixed, changes in population represent apparent migration.
population_in_riverine_A_zone.xlsx<br>population_in_storm_surge_A_zone.xlsx<br>population_in_X500_zone.csv<br>Black_in_storm_surge_A_zone.xlsx<br>Hispanic_in_riverine_A.xlsx<br>Hispanic_population_in_X500_zone.xlsx	Population for the years 1990, 2000, 2010, and 2020 , for all, Black, or Hispanic Residents, within the riverine A-zone, storm-surge A zone, or X500-zone. Because the measure of the floodplain is fixed, changes in population represent apparent migration.

Source documentation: see Titus, James. G. "Population in floodplains or close to sea level increased in US but declined in some counties—especially among Black residents". Environmental Research Letters. https://doi.org/10.1088/1748-9326/acadf5
