# Supplementary material for: Population in floodplains or close to sea level increased in US but declined in some counties—especially among Black residents
Source: Environ Res Lett. Author manuscript; Available in PMC 2025 Mar 14. (PMC11908447; doi:10.1088/1748-9326/acadf5)
Supplement: State and County Results [file NIHMS1876714-supplement-State_and_County_Results.zip › state_and_county/state_results/Readme.pdf]

In this zipped file, state population by elevation provides an estimate of the number of people in a given year within one meter above sea level for that year. All files here use the building-based density approach. State population by elevation migration only provides an estimate of the number of people in a given year within one meter above the sea level of 2020. Because a fixed sea level is used, population changes represent apparent migration.

The flood zone files show 100-year, 500-year, and velocity zones, and population in land presumably protected by a levee or dike.

Source Documentation: J.G. Titus, Environmental Research Letters (2023)

https://doi.org/10.1088/1748-9326/acadf5
