# Supplementary material for: Population in floodplains or close to sea level increased in US but declined in some counties—especially among Black residents
Source: Environ Res Lett. Author manuscript; Available in PMC 2025 Mar 14. (PMC11908447; doi:10.1088/1748-9326/acadf5)
Supplement: Supplemental Tables (portait) [file NIHMS1876714-supplement-Supplemental_Tables__portait_.pdf]

Supplemental Tables (Part 1: Portrait) from
'Population in floodplains or close to sea level increased in US but declined in some counties–especially among Black residents'

JAMES G TITUS

ENVIRONMENTAL RESEARCH LETTERS

FEBRUARY 2023

Note: This document includes only those tables produced in portrait format; Part 2 has the tables formatted in landscape. See Table of Tables. For more complete results by state or by county, go to EPA's Science Hub at https://doi.org/10.23719/1527848

TABLE OF SUPPLEMENTAL TABLES

S1-A Population Below 1 Meter for Four Assumptions on How Population is Distributed within Census Blocks

S1-B Population in 100-Year Floodplain for Four Assumptions on How Population is Distributed within Census Blocks

S2-A Ratio Estimate Mean and Standard Deviation of Population within One Meter above MHHW, assuming building-based density

S2-B Uncertainty and Statistical Significance of Decadal Changes in Population

S2-C Black and All Residents Living within One Meter above MHHW as a proportion of All Residents based on ratio estimate

S2-D Are Black Residents Disproportionately Over- or Underrepresented among Residents Living on Land Less than One Meter above MHHW? (correlation based on estimates of population below one meter)

S2-E Are Black Residents Disproportionately Over- or Underrepresented among Residents Living on Land Less than One Meter above MHHW? (based on measurement error of building based density ratio estimates)

S2-F Comparing This Analysis to Strauss et al. (2012): Area of Dry Land Close to Sea Level

S2-G Comparing This Analysis to Strauss et al. (2012): Population Close to Sea Level assuming Uniform Density

S3-A Population within 1 and 3 meters above MHHW, assuming building-based density

S3-B Population within 1 and 3 meters above MHHW, assuming uniform density

S3-C Population Change within 0, 1, 2, and 3 meters above MHHW, assuming building-based density

S3-D Population Change within 0, 1, 2, and 3 meters above MHHW, assuming uniform density

S3-E Apparent Migration into Land Below 1 meter relative to the MHHW of 2020, assuming building-based density

S4-A Population within Mapped[1] 100- and 500-year Coastal[2] Floodplains by State, assuming building-based density

S4-B Population within Mapped[1] 100- and 500-year Coastal[2] Floodplains by State, assuming uniform density

S4-C Population within FEMA Mapped 100-year and 500-year Coastal Floodplains, the Coastal Velocity Zone, and Land Protected from Flooding by Dikes or Levees, assuming building-based density

S4-D Population within 100-year and 500-year Coastal Floodplains, the Coastal Velocity Zone, and Land Protected from Flooding by Dikes or Levees, assuming uniform population density

S4-E Population within FEMA Mapped[1] 100-year and 500-year Inland[2] Floodplains, assuming building-based density

S4-F Population within FEMA Mapped[1] 100-year and 500-year Inland[2] Floodplains, assuming uniform density

S4-G Population Change within FEMA Mapped[1] 100-year and 500-year Inland Floodplains and Land Protected from Flooding by Dikes or Levees, assuming building-based density

S4-H Population Change within FEMA Mapped[1] 100-year and 500-year Inland Floodplains and Land Protected from Flooding by Dikes or Levees, assuming uniform density (thousands of people)

S4-J Population within FEMA Mapped[1] 100-year and 500-year Floodplains[2], assuming building-based density

S5-A Black Population within 1 and 3 meters above MHHW, assuming building-based density

S5-B Hispanic Population within 1 and 3 meters above MHHW, assuming building-based density

S6-A Population by Race in Various Vulnerable Zones as a Percentage of Total Population in that Vulnerable Zone or Nationwide Population of that Race

S6-B Population by Race in Counties with Land in Various Vulnerable Zones as a Percentage of Total Population in those Counties or Nationwide Population of that Race

S6-C Cause of Nationwide Disproportionality of Black Residents below One Meter—Total and Black Population in Entire Counties and Below One Meter by County, 2020, ranked by Fraction of County Population below One Meter.

S6-D Cause of Nationwide Disproportionality of Hispanic Residents below Three Meters—Population in Entire Counties and Below Three Meters by County, 2020, ranked by Fraction of County Population below Three Meters.

S7 Ratios of How Disproportional the Population of Blacks and Hispanics in Vulnerable Zones Is Compared to the Total Population of Blacks and Hispanics.

See File of Landscape Tables for S8-A to S9-D (i.e. Titus 2023a).

S8-A Apparent Emigration 1990–2020 from Land Below 1 meter by County, Ranked as a Percent of 1990 Population

S8-B Apparent Emigration 1990–2020 from Land Below 3 meters by County, Ranked as a Percent of 1990 Population

S8-C Apparent Black Emigration 1990–2020 from Land Below 1 meter by County, Ranked as a Percent of 1990 Population

S8-D Apparent Black Emigration 1990–2020 from Land Below 3 meters by County, Ranked as a Percent of 1990 Population

S8-E Apparent Emigration 1990–2020 from Inland 100-Year Floodplain by County, Ranked as a Percent of 1990 Population

S8-F Apparent Emigration 1990–2020 from Coastal 100-Year Floodplains by County, Ranked as a Percent of 1990 Population

S8-G Apparent Emigration 1990–2020 from 500-Year Floodplain by County, Ranked[1] by Emigration from X500 Zone

S8-H Apparent Emigration 1990–2020 by County from 100-Year Floodplain, Ranked[1] by Total Emigration

S8-J Apparent Emigration 1990–2020 from Floodplains and Lands Close to Sea Level

S9-A Summary Statistics Measuring whether Blacks Account for a Disproportionate Share of People Inhabiting Low Land Who Emigrate from Low Land, in Counties with Net Emigration from Low Land, by Decade

S9-B Summary Statistics Measuring whether Blacks Account for a Disproportionate Share of People Inhabiting Floodplains Who Emigrate from Floodplains, in Counties with Net Emigration from Floodplains, by Decade

S9-C Summary Statistics Measuring whether Blacks Account for a Disproportionate Share of Emigration from Low Land in Counties with Net Emigration by Decade

S9-D Summary Statistics Measuring whether Blacks Account for a Disproportionate Share of Emigration from Floodplains in Counties with Net Emigration by Decade

S9-E Summary Statistics Measuring whether Blacks Account for a Disproportionate Share of Emigration from Floodplains in Counties with Net Emigration

S9-F Summary Statistics Measuring whether Blacks Account for a Disproportionate Share of Emigration from Vulnerable Areas in Counties with Net Emigration

S10 Summary Statistics Measuring the Extent to which Blacks Account for a Disproportionate Share of Residents in and Emigration from, Vulnerable Areas 1990 to 2020

S10-i Summary Statistics Measuring the Extent to which Blacks Account for a Disproportionate Share of Residents Emigration from, Vulnerable Areas 1990 to 2020 (condensed version of S10)

S10-ii Black Share of Residents and Emigration from Vulnerable Areas, 1990 to 2020 (condensed version of S10)

See File of Landscape Tables for S11-A to S12-B (i.e. Titus 2023a).

S11-A Impact on Black Population below One Meter from the Implicit Racial Segregation Captured By HOLC Mapping, Ranked by Impact in 2020

S11-B Impact on Black Population below One Meter from the Implicit Racial Segregation Captured By HOLC Mapping, Ranked by Impact in 2020.

S11-C Impact on Black Population in the 100-year Floodplain from the Implicit Racial Segregation Captured By HOLC Mapping, Ranked by County Impact in 2020.

S12-A Impact on Black Population Below One Meter from the Implicit Racial Segregation Captured By HOLC Mapping, by HOLC Zone and Year (thousands)

S12-B Impact on Black Population Below Three Meters from the Implicit Racial Segregation Captured By HOLC Mapping, by HOLC Zone and Year (thousands

S12-C Impact on Black Population in the 100-year Floodplain from the Implicit Racial Segregation Captured By HOLC Mapping, by HOLC Zone and Year (thousands)

S12-D Impact on Black Population in Hazard Zones, by HOLC Zone, from the Implicit Racial Segregation Captured By HOLC Mapping: Additional Explanations of Calculations from Tables S12-A, S12-B, and S12-C.

S13-A. Apparent Emigration 1990–2020 from 100-Year Floodplain in HOLD Red Zone by County

S13-B Apparent Emigration 1990–2020 from 100-Year Floodplain in HOLD Yellow Zone by County

S13-C Measurement Error and Statistical Significance for Emigration from Land Below One Meter

S14-A. Change in Population of Land Less than One Meter Above Sea Level Along US Atlantic Coast, by State and Likelihood of Shore Protection

S14-B. Change In Black Population of Land Less than One Meter Above Sea Level Along US Atlantic Coast, by State and Likelihood of Shore Protection

S14-C: Change in Population of Land Less than One Meter Above Sea Level in Atlantic Counties with Greatest Emigration, by Likelihood of Shore Protection

S14-D: Change In Black Population of Land Less than One Meter Above Sea Level in Atlantic Counties with Greatest Emigration, by Likelihood of Shore Protection

Table S1-A
Population Below 1 Meter for Four Assumptions on How Population is Distributed within Census Blocks
(thousands of people)

	**(1)**	**(2)**	**(3)**	**(4)**	**(5)**	**(6)**	**(7)**	**(8)**	**(9)**	**(10)**	**(11)**
	1990				2020						
State or Locality	Entirely in Zone[1]	Partly in Zone[2]	Uniform Density [3]	Building -Based[4]	Entirely in Zone[1]	Partly in Zone[2]	Uniform Density [3]	Building -Based[4]	Portion of Population below 1 m in Partly Vulnerable Blocks		Density of Blocks with Land <1m [7]
									Uniform[5]	Building[6]	
**ME**	0	82	4	1	0	99	4	2	0.04	0.02	0.5
**NH**	0	21	3	2	0	27	4	3	0.13	0.09	3.2
**MA**	3	411	80	62	4	528	117	94	0.22	0.17	6.9
**RI**	0.001	54	5	2	0.024	46	6	2	0.12	0.04	3.3
**CT**	0.6	142	19	11	1	157	25	14	0.15	0.08	5.1
**NY**	2	708	101	67	3	862	148	105	0.17	0.12	11.7
**NJ**	11	503	131	91	24	623	181	130	0.26	0.18	7.0
**PA**	0.026	35	5	2	0.021	31	5	2	0.16	0.05	9.5
**DE**	0.3	55	8	4	0.4	95	15	9	0.15	0.10	1.5
**DC**	0	13	0.29	0.23	0	4	0.7	0.5	0.16	0.12	18.9
**VA**	0.6	560	58	27	1	638	77	36	0.12	0.05	1.5
**MD**	3	273	32	21	4	343	40	25	0.11	0.06	0.9
**NC**	2	273	43	26	3	333	56	32	0.16	0.09	0.6
**SC**	12	237	55	33	8	386	85	52	0.21	0.12	1.5
**GA**	1.3	98	19	11	2	162	33	19	0.19	0.11	1.5
**FLE**[8]	68	2,155	561	388	85	3,221	876	585	0.25	0.16	8.0
**FLW**[8]	14	1,004	199	123	17	1,462	292	168	0.19	0.10	2.3
**AL**	0.3	33	4	3	0.39	47	7	6	0.15	0.12	0.9
**MS**	0.026	62	6	3	0.08	64	8	3	0.12	0.05	1.6
**LA**	817	1,377	1,070	1,008	750	1,364	1,003	940	0.41	0.31	2.3
Orleans	400	464	437	424	313	364	343	333	0.59	0.40	30.9
Jefferson	330	427	394	387	343	418	396	392	0.70	0.65	21.2
**TX**	28	220	58	50	27	295	71	56	0.16	0.11	0.6
**CA**	38	399	123	106	49	459	154	134	0.26	0.21	2.5
**OR**	0.001	26	2	2	0	33	2	2	0.06	0.05	0.2
**WA**	3	132	15	5	0.22	149	14	6	0.09	0.04	0.5
**HI**	1.4	188	27	11	0.16	185	31	18	0.17	0.09	0.7
**USA**	1,005	9,059	2,629	2,060	980	11,613	3,253	2,442	0.21	0.14	2.2
**USA** except LA	188	7,682	1,559	1,052	230	10,249	2,250	1,502	0.20	0.13	2.1

Notes
1. Population of blocks that are entirely in the vulnerable zone.
2. Population of blocks with at least 0.5% of dry land in zone.
3. In each block, the fraction of people in the floodplain equals the fraction of the block's dry land area that is in the floodplain.
4. In each block, the fraction of people in the floodplain equals the fraction of the block's buildings that are in the floodplain.
5. (Column[7]-Column[5])/(Column[6]- Column[5])
6. (Column[8]-Column[5])/(Column[6]- Column[5])
7. Population per hectare in populated blocks with dry land <1m. Column (6) divided by area of populated blocks with land <1m.
8. FLE includes all counties in Florida along the Atlantic Coast, including Monroe. FLW is the rest of the state.

End

Table S1-B: Population in 100-Year Floodplain for Four Assumptions on How Population is Distributed within Census Blocks

	(1)	(2)	(3)	(4)		(5)	(6)	(7)	(8)	(9)	(10)
	All A-Zones					River and Lake A-Zones					
State or Locality	Entirely in Zone[1]	Partly in Zone[2]	Uniform Density[3]	Building-Based[4]		Entirely in Zone[1]	Partly in Zone[2]	Uniform Density[3]	Building-Based[4]		
AK	1,691	111,869	14,119	5,308		1,691	111,869	14,119	5,308		
AL	18,548	1,778,078	229,717	125,875		9,585	1,683,632	198,866	98,268		
AR	16,893	966,850	168,861	92,865		16,893	966,850	168,861	92,865		
AZ	51,235	836,519	188,697	131,591		51,235	836,519	188,697	131,591		
CA	383,346	4,240,240	1,060,794	857,115		270,308	3,454,897	789,369	621,329		
CO	9,543	722,645	96,103	52,750		9,543	722,645	96,103	52,750		
CT	988	100,058	12,014	7,530		71	57,512	4,318	1,678		
DC	804	28,213	6,755	5,587		310	13,693	2,155	1,925		
DE	4,918	261,306	40,538	26,190		297	117,789	11,102	4,098		
FL	1,223,243	9,637,900	3,052,799	2,403,109		46,194	3,692,264	637,280	341,582		
GA	17,716	4,578,438	441,003	174,996		8,234	4,345,610	366,624	121,397		
HI	30,747	280,109	77,379	58,636		1,252	100,318	11,166	7,683		
IA	7,218	743,803	102,906	43,186		7,218	743,803	102,906	43,186		
ID	5,195	153,376	36,368	29,849		5,195	153,376	36,368	29,849		
IL	31,814	1,548,609	259,024	127,214		31,814	1,548,609	259,024	127,214		
IN	25,805	1,479,926	236,753	121,014		25,805	1,479,926	236,753	121,014		
KS	9,586	596,691	96,793	41,819		9,586	596,691	96,793	41,819		
KY	20,358	1,778,932	235,119	144,249		20,358	1,778,932	235,119	144,249		
LA	233,542	2,252,277	904,406	690,459		68,463	970,102	312,509	230,038		
MA	32,951	1,507,051	253,834	163,190		3,986	1,055,954	114,861	48,821		
MD	8,826	1,680,173	156,840	57,817		655	1,268,442	100,933	20,786		
ME	234	264,854	17,140	8,314		185	197,365	13,620	6,517		
MI	30,599	1,417,839	197,376	120,106		30,599	1,417,839	197,376	120,106		
MN	2,635	675,752	68,840	26,401		2,635	675,752	68,840	26,401		
MO	9,194	1,632,769	165,878	64,988		9,194	1,632,769	165,878	64,988		
MS	62,720	1,333,555	297,392	190,062		23,142	1,132,496	211,960	115,671		
MT	6,845	138,747	30,473	23,256		6,845	138,747	30,473	23,256		
NC	41,495	3,712,555	399,666	200,261		6,684	3,223,281	263,450	88,086		
ND	7,023	143,684	51,637	29,370		7,023	143,684	51,637	29,370		
NE	24,073	311,906	84,592	57,615		24,073	311,906	84,592	57,615		
NH	1,870	462,658	48,519	26,484		694	430,757	43,233	22,099		
NJ	208,883	1,871,535	512,727	420,941		18,264	1,110,631	143,830	93,094		
NM	50,158	523,444	125,121	95,554		50,158	523,444	125,121	95,554		
NV	21,999	274,363	59,211	43,564		21,999	274,363	59,211	43,564		
NY	184,156	2,312,216	645,116	515,607		9,436	1,309,758	180,539	92,055		
OH	37,342	2,164,586	322,576	166,353		37,342	2,164,586	322,576	166,353		
OK	13,273	1,095,445	169,723	79,996		13,273	1,095,445	169,723	79,996		
OR	14,187	700,886	136,056	93,132		12,475	606,252	117,756	80,012		
PA	41,816	2,824,974	368,494	214,497		37,788	2,779,432	350,871	203,178		
RI	3,800	192,360	35,909	22,778		207	119,298	13,780	5,662		
SC	5,765	1,361,661	123,334	46,875		2,373	1,265,699	97,438	30,822		
SD	2,637	147,591	25,929	16,612		2,637	147,591	25,929	16,612		
TN	6,671	1,739,000	231,124	105,747		6,671	1,739,000	231,124	105,747		
TX	387,423	7,455,411	1,611,058	944,840		314,752	6,993,385	1,419,537	791,888		
UT	1,655	242,047	30,419	19,015		1,655	242,047	30,419	19,015		
VA	30,211	2,994,227	458,731	195,670		5,318	2,162,377	226,282	68,079		
VT	940	154,105	19,542	11,633		940	154,105	19,542	11,633		
WA	25,842	618,176	97,119	67,605		14,595	436,072	63,410	40,424		
WI	6,162	1,023,687	114,579	56,845		6,162	1,023,687	114,579	56,845		
WV	19,009	704,022	127,604	121,262		19,009	704,022	127,604	121,262		
WY	901	74,332	10,281	7,466		901	74,332	10,281	7,466		
USA	3,384,485	73,851,450	14,256,987	9,353,199		1,275,722	59,929,555	8,964,536	4,970,822		

Notes:
1. Population of blocks that are entirely in the vulnerable zone.
2. Population of blocks with at least 0.5% of dry land in zone.
3. In each Census block, the fraction of people in the floodplain equals the fraction of the block's dry land area that is in the floodplain.
4. In each Census block, the fraction of people in the floodplain equals the fraction of the block's buildings that are in the floodplain.

Table S2-A
Ratio Estimate Mean and Standard Deviation of Population within One Meter above MHHW, assuming building-based density
(thousands of people)

	1990		2000		2010		2020		Coeff Var (%)	
State or Locality	$\hat{X}$	$\hat{\sigma}_X$	$\hat{X}$	$\hat{\sigma}_X$	$\hat{X}$	$\hat{\sigma}_X$	$\hat{X}$	$\hat{\sigma}_X$	**1990**	**2020**
**ME**	1.2	0.2	1.4	0.2	1.2	0.2	1.3	0.2	16	16
**NH**	1.5	0.2	1.9	0.3	1.8	0.3	2.1	0.3	15	15
**MA**	51	8	58	9	63	10	76	12	15	15
**RI**	1.3	0.2	1.4	0.2	1.3	0.2	1.5	0.2	16	16
**CT**	8.6	1.3	9.1	1.3	9.8	1.4	11.2	1.5	15	14
**NY**	54	8	62	10	70	11	85	13	16	15
**NJ**	75	10	88	12	94	12	109	13	14	12
**PA**	1.7	0.3	1.1	0.2	1.4	0.2	1.3	0.2	16	16
**DE**	3.6	0.5	5.5	0.8	6.7	1.0	7.8	1.1	15	15
**DC**	0.2	0.0	0.1	0.0	0.3	0.0	0.4	0.1	16	16
**VA**	22	3	25	4	25	4	29	4.4	15	15
**MD**	18	2	20	2	21	3	21	2.7	12	13
**NC**	22	3	24	3	26	4	26	3.8	14	14
**SC**	28	3	32	4	37	5	43	5.6	13	13
**GA**	9.4	1.3	11.6	1.7	12.7	1.8	15.9	2.3	14	14
**FLE**[1]	326	41	372	48	414	52	489	63	13	13
**FLW**[1]	102	14	119	16	126	17	139	19	14	14
**AL**	2.9	0.4	4.0	0.6	4.1	0.6	4.8	0.7	14	15
**MS**	2.1	0.3	3.3	0.5	2.3	0.4	2.5	0.4	16	16
**LA**[2]	971	24	989	24	837	23	904	23	2.5	2.6
Orleans	419	3.9	411	3.6	285	3.2	329	3.1	0.9	0.9
Jefferson	0.15	0.02	0.15	0.02	0.16	0.02	0.19	0.03	15	13.2
St. Bernard	48	1.6	50	1.3	26	0.7	33	0.9	3.4	2.5
**TX**	46	2.8	49	3.4	48	3.2	51	3.7	6.1	7.3
**CA**	93	8	105	10	113	10	118	11	9	9
**OR**	1.7	0.3	1.7	0.3	1.6	0.3	1.3	0.2	16	16
**WA**	3.9	0.6	4.8	0.7	4.2	0.6	4.7	0.7	16	15
**HI**[3]	9.2	1.5	9.2	1.5	12.7	2.0	14.2	2.2	16	16
**USA**	1,853	136	1,996	153	1,933	162	2,160	185	7.3	9

Notes
1. FLE includes all counties in Florida that drain into the Atlantic Ocean, including Monroe. FLW is the rest of the state.
2. Statewide total. Also shown are the three Parishes with the most significant decline during the 2000s after Hurricane Katrina.
3. Significant errors in the mapping of the 1990 census block boundaries were corrected for the 2000 census; so changes between 1990 and 2000 are likely to reflect that correction as well as actual population changes.

The purpose of this table is to answer: "How many people live below one meter" with an uncertainty range.

Table S2-B
Uncertainty and Statistical Significance of Decadal Changes in Population
(based on measurement error of building based density ratio estimates)

State or City	Population[a]		Standard Error[a]		2010–2020					1990-2020		
	2010	2020	2010	2020	Δ^b	$\Delta\%$ [c]	$\sigma_{\Delta,10}$ [d]	$\frac{\sigma_\Delta}{X_{2020}}$ [e]	t statistic[f]	σ_Δ [d]	$\frac{\sigma_\Delta}{X_{2020}}$ [e]	$1/t^g$
**ME**	1,171	1,263	187	199	91	7.8	98	7.8	0.93	158	12.5	1.75
**NH**	1,765	2,080	261	304	315	17.8	148	7.1	2.12	234	11.3	0.37
**MA**	63,073	76,341	9,570	11,540	13,268	21.0	5,651	7.4	2.35	8,820	11.6	0.34
**RI**	1,348	1,505	214	238	157	11.6	116	7.7	1.35	186	12.4	0.76
**CT**	9,798	11,164	1,393	1,548	1,365	13.9	756	6.8	1.81	1,212	10.9	0.48
**NY**	69,511	85,257	10,647	13,119	15,746	22.7	6,449	7.6	2.44	9,980	11.7	0.32
**NJ**	94,328	109,332	11,722	13,318	15,004	15.9	6,496	5.9	2.31	10,346	9.5	0.30
**PA**	1,379	1,273	217	201	-106	-7.7	107	8.4	-0.99	172	13.5	-0.40
**DE**	6,669	7,765	961	1,138	1,096	16.4	556	7.2	1.97	873	11.2	0.21
**DC**	265	424	43	68	159	59.8	37	8.8	4.26	51	12.0	0.21
**VA**	25,436	29,167	3,767	4,416	3,731	14.7	2,155	7.4	1.73	3,399	11.7	0.44
**MD**	20,627	21,392	2,549	2,698	765	3.7	1,330	6.2	0.57	2,150	10.1	0.57
**NC**	25,702	26,391	3,627	3,758	690	2.7	1,865	7.1	0.37	3,023	11.5	0.64
**SC**	36,868	43,477	4,802	5,579	6,609	17.9	2,722	6.3	2.43	4,305	9.9	0.28
**GA**	12,749	15,888	1,818	2,301	3,139	24.6	1,138	7.2	2.76	1,741	11.0	0.27
**FL**	539,267	627,306	1,266	1,844	88,040	16.3	963	6.4	2.18	63,330	10.1	0.32
**FLE**	413,674	488,737	52,298	63,483	75,063	18.1	31,119	6.4	2.41	48,449	9.9	0.30
**FLW**	125,593	138,569	17,043	19,134	12,976	10.3	9,338	6.7	1.39	14,921	10.8	0.41
**AL**	4,063	4,843	589	705	779	19.2	345	7.1	2.26	540	11.2	0.27
**MS**	2,326	2,487	352	387	161	6.9	189	7.6	0.85	304	12.2	0.78
**LA**[2]	836,818	903,989	23,042	23,489	67,171	8.0	11,733	1.3	5.72	19,038	2.1	-0.28
**TX**	47,819	50,564	3,171	3,698	2,744	5.7	1,804	3.6	1.52	2,850	5.6	0.56
**CA**	113,344	117,905	10,330	10,767	4,560	4.0	5,333	4.5	0.86	8,639	7.3	0.34
**OR**	1,618	1,280	257	206	-338	-20.9	127	9.9	-2.67	195	15.2	-0.45
**WA**	4,207	4,721	638	715	515	12.2	349	7.4	1.47	558	11.8	0.65
**HI**[3]	12,696	14,232	2,021	2,246	1,536	12.1	1,097	7.7	1.40	1,758	12.4	0.35
**USA**	1,932,847	2,160,044	161,521	185,255	227,197	11.8	90,353	4.2	2.51	143,496	6.6	0.47
New Orleans	284,570	328,665	3,180	3,085	44,094	15.5	1,581	0.5	27.88	2,564	0.8	-0.03
Rest of USA	1,648,277	1,831,379	158,340	182,170	183,102	11.1	88,851	4.9	2.06	140,976	7.7	0.36

Notes

a. Ratio estimates of population and Standard error of population estimates. Derived from Table S2-A.
b. Change in Population
c. Percentage Change in Population
d. Standard error of estimated change in population over a 10 or 30 year period ending in 2020, depending on subscript.
e. Standard error of population change as a percentage of the population of 2020.
f. Estimated population change divided by standard error of population change $\Delta / \sigma_{\Delta,10}$ The ratio is a t-statistic whose statistical significance can be evaluated assuming 14 degrees of freedom. Roughly, the change is statistically significant when the ratio is greater than 2.0. A 90% confidence range is roughly Δ^b plus or minus $\sigma_{\Delta,10}{}^d$ times 1.7613, Comparable 95% threshold would replace 1.7613 with 2.1448.
g. The reciprocal of the t-statistic in note f but for 30-year changes, $\sigma_{\Delta,30}$ / Δ_{30}.. Result is presented in this format to better support text associated with Figure 2.

The purpose of this table is to show the uncertainty of estimates of changes in population vulnerable to sea level rise

Table S2-C<br>Black and All Residents Living within One Meter above MHHW as a proportion of All Residents based on ratio estimate[1]<br>(percent)										
	1990		2000		2010		2020		σ Difference[2]	
State or Locality	**All**	**Black**	**All**	**Black**	**All**	**Black**	**All**	**Black**	**1990**	**2020**
**ME**	0.095	0.050	0.107	0.132	0.088	0.054	0.093	0.073	0.015	0.015
**NH**	0.131	0.114	0.150	0.142	0.134	0.084	0.151	0.057	0.020	0.022
**MA**	0.843	1.676	0.909	1.370	0.963	1.152	1.086	1.161	0.129	0.157
**RI**	0.125	0.050	0.135	0.058	0.128	0.032	0.137	0.053	0.020	0.021
**CT**	0.262	0.422	0.268	0.388	0.274	0.289	0.310	0.292	0.038	0.042
**NY**	0.301	0.342	0.329	0.419	0.359	0.437	0.422	0.587	0.044	0.063
**NJ**	0.974	0.986	1.045	0.993	1.073	0.946	1.177	0.980	0.130	0.142
**PA**	0.014	0.089	0.009	0.067	0.011	0.069	0.010	0.062	0.004	0.003
**DE**	0.540	0.415	0.702	0.258	0.743	0.328	0.784	0.336	0.076	0.113
**DC**	0.030	0.007	0.014	0.007	0.044	0.046	0.061	0.030	0.005	0.009
**VA**	0.348	0.280	0.354	0.300	0.318	0.286	0.338	0.330	0.052	0.049
**MD**	0.368	0.196	0.369	0.156	0.357	0.131	0.346	0.119	0.044	0.043
**NC**	0.327	0.365	0.293	0.304	0.270	0.233	0.253	0.164	0.044	0.036
**SC**	0.799	0.695	0.801	0.581	0.797	0.514	0.849	0.455	0.094	0.106
**GA**	0.145	0.063	0.141	0.063	0.132	0.052	0.148	0.061	0.020	0.021
**FL**	3.307	1.816	3.069	1.879	2.868	1.907	2.913	1.976	0.421	0.375
**AL**	0.071	0.006	0.089	0.008	0.085	0.008	0.096	0.008	0.010	0.014
**MS**	0.081	0.012	0.117	0.022	0.078	0.016	0.084	0.022	0.013	0.013
**LA**	23.01	27.84	22.14	28.22	18.46	21.84	19.41	22.89	0.542	0.475
**TX**	0.268	1.039	0.233	0.820	0.190	0.552	0.173	0.431	0.017	0.012
**CA**	0.312	0.209	0.309	0.230	0.304	0.277	0.298	0.257	0.027	0.026
**OR**	0.060	0.031	0.050	0.018	0.042	0.024	0.030	0.014	0.009	0.005
**WA**	0.079	0.018	0.081	0.093	0.063	0.020	0.061	0.048	0.012	0.009
**HI[3]**	0.832	0.802	0.758	0.880	0.933	1.145	0.978	1.305	0.124	0.140
**USA**	0.745	1.576	0.709	1.545	0.619	1.171	0.645	1.175	0.052	0.054
New Orleans	84.234	90.01	84.820	91.046	82.765	91.795	85.59	94.385	0.714	0.751
USA except N. Orleans	0.578	0.643	0.564	0.684	0.528	0.675	0.548	0.692	0.052	0.053

Notes

1. Calculated as 100 times the ratio estimate of Black or all residents living below one meter to Black or total population. See Table S2-A and "Preliminary Test of Assumptions about Population Distributions within Census Blocks Close to Sea Level", Supplemental Methods Appendix for additional details of the ratio estimates.

2. Standard deviation of the difference between the Black and all proportions based on (a) standard deviations from Table S2-A and comparable estimates for Black populations and correlation between Black and total population in Table S2-C.

3. Significant errors in the mapping of the 1990 census block boundaries were corrected for the 2000 census; so changes between 1990 and 2000 are likely to reflect that correction as well as actual population changes.

The purposes of this table is to answer: What percentage of all people (or all Black people) live within one meter of sea level?

Table S2-D
Are Black Residents Disproportionately Over- or Underrepresented
among Residents Living on Land Less than One Meter above MHHW?
(correlation based on estimates of population below one meter)

State or City	**Correlation between Black and Total Population**				**Statistical Significance of Disproportionality[2]**				**Ratio of Disproportionality if significantly different from 1.0[3]**			
	**1990**	**2000**	**2010**	**2020**	**1990**	**2000**	**2010**	**2020**	**1990**	**2000**	**2010**	**2020**
**ME**	0.47	0.43	0.38	0.04	-3.06		-2.47		0.523		0.612	
**NH**	0.28	0.73	0.65	0.17			-2.65	-4.29			0.625	0.375
**MA**	0.61	0.56	0.52	0.60	6.48	3.40			1.989	1.507		
**RI**	0.28	0.19	0.53	0.32	-3.76	-3.58	-4.83	-3.93	0.402	0.426	0.247	0.385
**CT**	0.57	0.60	0.46	0.47	4.20	3.22			1.610	1.452		
**NY**	0.68	0.73	0.61	0.58		1.89		2.63		1.271		1.391
**NJ**	0.41	0.45	0.43	0.35								
**PA**	0.86	0.98	0.92	0.95	19.41	21.00	20.02	20.06	6.185	7.409	6.374	6.351
**DE**	0.52	0.21	0.50	0.34		-4.52	-3.98	-3.96		0.367	0.442	0.428
**DC**	0.33	0.77	0.77	0.96	-4.79	-3.16		-3.43	0.238	0.516		0.482
**VA**	0.48	0.57	0.50	0.55								
**MD**	0.67	0.59	0.62	0.51	-3.94	-4.74	-5.28	-5.32	0.532	0.422	0.365	0.342
**NC**	0.55	0.64	0.63	0.26				-2.48				0.650
**SC**	0.68	0.49	0.45	0.44		-2.24	-2.80	-3.70		0.726	0.645	0.536
**GA**	0.34	0.39	0.45	0.56	-4.14	-3.95	-4.32	-4.20	0.437	0.444	0.396	0.409
**FL**	0.30	0.34	0.40	0.43	-3.54	-3.02	-2.66	-2.50	0.549	0.612	0.665	0.678
**AL**	0.12	0.13	0.13	0.07	-6.42	-6.21	-6.29	-6.28	0.081	0.090	0.089	0.085
**MS**	0.21	0.55	0.41	0.40	-5.42	-5.31	-5.33	-4.82	0.142	0.189	0.202	0.259
**LA**[2]	0.68	0.74	0.71	0.69	8.90	11.89	7.06	7.33	1.210	1.275	1.183	1.179
**TX**	0.70	0.80	0.72	0.71	45.99	36.51	30.18	21.37	3.879	3.514	2.904	2.485
**CA**	0.60	0.61	0.74	0.54	-3.79	-2.87			0.669	0.745		
**OR**	0.84	0.54	0.99	0.92	-3.24	-4.14	-2.87	-3.63	0.521	0.369	0.577	0.448
**WA**	0.41	0.83	0.48	0.78	-5.07		-4.60		0.220		0.316	
**HI**[3]	0.71	0.56	0.84	0.83				2.33				1.334
**USA**	0.57	0.64	0.55	0.52	15.84	16.19	11.08	9.89	2.115	2.177	1.893	1.821
New Orleans	0.90	0.96	0.87	0.78	8.09	9.07	10.53	11.71	1.069	1.073	1.109	1.103
Rest of USA	0.45	0.49	0.53	0.51		2.31	3.01	2.74		1.212	1.279	1.264

Notes
1. Pearson correlation coefficient between Black and total population per block projected based on building-based density for every block with land and Black population below 1 meter.
2. Student's t statistic based on the differences between the proportions of Black and all residents living below 1m, and the standard deviations of that difference (see Table S2-B). The table does not show values less than 1.7613, which is the Student's t upper tail for 90% confidence interval and 14 degrees of freedom. Comparable 95% threshold would be 2.1448.
3. Ratio of disproportionality where the differences in proportions are statistically significant. Because ratio estimates of total and Black population used to assess statistical significance are about 20% and 2% lower than the building-based density estimates in blocks that are partly below one meter, these ratios are larger than the ratios reported in the main paper.

The purpose of this table is to answer for each state: Do Black people disproportionately live in land vulnerable to sea level rise (i.e. land below one meter), to what extent, and is the disproportionality statistically significant?

Table S2-E
Are Black Residents Disproportionately Over- or Underrepresented among Residents Living on Land Less than One Meter above MHHW?
(based on measurement error of building based density ratio estimates)

State or City	Difference in Proportions				Statistical Significance of Disproportionality[3]				Ratio of Disproportionality if significantly different from 1.0[4]			
	Estimate[1]		Stand. Dev[2]									
	1990	2020	1990		1990	2000	2010	2020				
ME	-0.046	-0.019	0.015	0.014	-3.03		-2.46		0.52		0.61	
NH	-0.017	-0.094	0.020	0.022			-2.57	-4.34			0.63	0.37
MA	0.834	0.076	0.139	0.164	6.00	3.23			1.99	1.51		
RI	-0.075	-0.084	0.020	0.021	-3.77	-3.62	-4.79	-3.94	0.40	0.43	0.25	0.39
CT	0.160	-0.017	0.040	0.043	3.98	3.03			1.61	1.45		
NY	0.041	0.165	0.047	0.066				2.50				1.39
NJ	0.012	-0.197	0.132	0.142								
PA	0.074	0.052	0.004	0.003	16.75	17.89	17.00	16.88	6.19	7.41	6.37	6.35
DE	-0.125	-0.449	0.078	0.113		-4.56	-3.93	-3.96		0.37	0.44	0.43
DC	-0.023	-0.032	0.005	0.010	-4.79	-3.06		-3.27	0.24	0.52		0.48
VA	-0.069	-0.008	0.052	0.051								
MD	-0.172	-0.228	0.045	0.043	-3.84	-4.68	-5.20	-5.27	0.53	0.42	0.37	0.34
NC	0.038	-0.089	0.045	0.035				-2.50				0.65
SC	-0.104	-0.394	0.099	0.107		-2.20	-2.76	-3.67		0.73	0.64	0.54
GA	-0.082	-0.088	0.020	0.021	-4.14	-3.94	-4.29	-4.14	0.44	0.44	0.40	0.41
FL	-1.491	-0.937	0.420	0.379	-3.55	-3.02	-2.64	-2.47	0.55	0.61	0.66	0.68
AL	-0.065	-0.088	0.010	0.014	-6.44	-6.23	-6.31	-6.30	0.08	0.09	0.09	0.09
MS	-0.070	-0.062	0.013	0.013	-5.43	-5.28	-5.32	-4.80	0.14	0.19	0.20	0.26
LA[2]	4.826	3.482	0.568	0.498	8.50	11.26	6.74	6.99	1.21	1.27	1.18	1.18
TX	0.771	0.258	0.019	0.013	41.14	32.13	27.38	19.55	3.88	3.51	2.90	2.48
CA	-0.103	-0.041	0.028	0.027	-3.66	-2.78			0.67	0.74		
OR	-0.029	-0.017	0.009	0.005	-3.10	-4.09	-2.70	-3.49	0.52	0.37	0.58	0.45
WA	-0.062	-0.013	0.012	0.009	-5.06		-4.56		0.22		0.32	
HI[3]	-0.030	0.327	0.131	0.156				2.09				1.33
USA	0.831	0.530	0.055	0.055	15.20	15.36	10.69	9.60	2.12	2.18	1.89	1.82
New Orleans	5.780	8.795	0.768	0.793	7.52	8.44	9.90	11.09	1.07	1.07	1.11	1.10
Rest of USA	0.065	0.145	0.053	0.054		2.25	2.91	2.67		1.21	1.28	1.26

Notes
1. Percent of black residents below one meter (from Table S2-B) minus percent of all residents below one meter (Table S2-B).
2. Standard deviation of the percentages.
3. Student's t statistic based on the differences between the proportions of Black and all residents living below 1m, and the standard deviations of that difference (see Table S2-B). The table does not show values less than 1.7613, which is the Student's t upper tail for 14 degrees of freedom and 90% confidence interval. Comparable 95% threshold would be 2.1448.
4. Ratio of disproportionality where the differences in proportions are statistically significant. Because ratio estimates of total and Black population used to assess statistical significance are about 20% and 2% lower than the building-based density estimates in blocks that are partly below one meter, these ratios are larger than the ratios reported in the main paper.

The purpose of this table is to answer for each state: Do Black people disproportionately live in land vulnerable to sea level rise (i.e. land below one meter), to what extent, and is the disproportionality statistically significant?

beg

Table S2-F
Comparing This Analysis to Strauss et al. (2012): Area of Dry Land Close to the Sea Level of 1990 or 1992
(square kilometers)

	<0m		<1m		<2m		<3m	
State or Locality	Strauss[1] (1992)	This paper[2] (1990)	Strauss[1] (1992)	This paper[2] (1990)	Strauss[1] (1992)	This paper[2] (1990)	Strauss [1] (1992)	This paper[2] (1990)
**Maine**	29	14	54	42	96	95	141	163
**New Hampshire**	2	1	5	6	11	14	15	22
**Massachusetts**	29	18	86	95	199	227	312	343
**Rhode Island**	3	3	11	17	23	40	42	63
**Connecticut**	13	4	27	32	48	82	74	130
**New York**	51	14	155	109	294	298	468	466
**New Jersey**	45	36	174	210	454	506	720	746
**Pennsylvania**	3	4	7	13	25	36	40	61
**Delaware**	6	10	90	99	195	216	316	336
**DC**	1	0.4	2	2	5	4	9	7
**Virginia**	23	33	410	284	923	745	1,383	1,358
**Maryland**	25	37	315	466	730	959	1,504	1,395
**North Carolina**	128	196	1,288	1,433	2,614	2,810	3,835	4,160
**South Carolina**	62	106	439	490	1,006	1,089	1,421	1,686
**Georgia**	106	34	331	235	830	678	1,379	1,226
**Florida**	74	558	1,654	3,107	4,238	7,553	8,646	12,436
Atlantic[3]	-	117	-	868	-	3,162	-	5,598
Gulf[3]	-	440	-	2,239	-	4,391	-	6,839
**Alabama**	2	6	35	33	112	106	218	179
**Mississippi**	3	3	34	39	151	130	289	248
**Louisiana**[2]	1,077	1,074	3,058	3,021	4,998	4,934	6,620	6,495
**Texas**	17	129	284	657	2,117	2,012	3,894	3,705
**California**	93	232	378	335	686	667	1,203	1,078
**Oregon**	27	25	54	51	168	88	218	147
**Washington**	185	52	289	205	477	346	590	493
**Hawaii**	-	11	-	25	-	78	-	138
**USA except AK**		2,598	-	11,005	-	23,712	-	37,083
Contiguous	2,002	2,587	9,181	10,980	20,399	23,634	33,336	36,945

Notes

1. From Strauss et al., 2012. "Tidally adjusted estimates of topographic vulnerability to sea level rise and flooding for the contiguous United States." *Environmental Research Letters* 7, no. 1 (2012): 014033, Table A.1. Elevations are relative to NOAA's mean higher high water for the National Tidal Datum Epoch of 1983-2001.
2. This column shows the area estimates that underly all our reported results based on sea level of 1990; that is, we have not prepared a table of estimates based on sea level of the Tidal Datum epoch, which Strauss et al. used. The differences are small. Sea level for 1990 was calculated by subtracting two times the annual rate of relative sea level rise from the tidal datum epoch sea level, because 1992 is the midpoint of 1983-2001.
3. Atlantic includes all counties in Florida that drain into the Atlantic Ocean, including Monroe. Gulf is the rest of the state.

This table compares how many people live below sea level (or 1, 2, or 3 meters) according to two studies.

Table S2-G: Comparing This Analysis to Strauss et al. (2012):
Population Close to Sea Level in the year 2010 assuming Uniform Density
(thousands)

	Directly Comparable			Roughly Comparable[4]					
	<1m			<0m		<2m		<3m	
State or Locality	Strauss[1]	This paper[2]	Building Based[3]	Strauss[1]	This paper	Strauss[1]	This paper	Strauss[1]	This paper
**Maine**	7.4	3.8	1.4	4.1	1.0	11	10	16	18
**New Hampshire**	2.7	2.9	2.0	0.8	0.2	6.1	7	8.4	10
**Massachusetts**	52	88	71	20	14.4	190	288	319	427
**Rhode Island**	3.8	4.8	1.5	1.2	0.7	9.0	14	20	27
**Connecticut**	23	19	11	13	2.2	37	60	54	101
**New York**	301	107	72	111	6.8	612	532	1,009	1006
**New Jersey**	155	140	94	37	9.6	353	407	552	622
**Pennsylvania**	0.8	3.4	1.4	0.3	0.8	6.0	11	14	31
**Delaware**	7	11	7	0.4	1.0	26	28	44	47
**DC**	28	34	21	1.5	3.5	81	89	145	141
**Virginia**	0.8	0.2	0.3	0.1	0.02	1.6	0.8	5.9	3.3
**Maryland**	76	60	25	4.4	7.3	206	238	641	600
**North Carolina**	59	48	27	6.6	3.2	145	128	232	216
**South Carolina**	61	64	40	18	14	153	180	226	297
**Georgia**	28	25	14	4.3	2.4	89	83	155	152
**Florida**	1,609	890	564	66	93	3,763	3,943	5,797	5,895
Atlantic[5]	-	662	432	-	63	-	3,170	-	4,614
Gulf[5]	-	228	132	-	29	-	773	-	1,280
**Alabama**	3.3	4.9	4.6	0.5	1.2	12	16	33	29
**Mississippi**	4.4	5.9	2.4	0.4	0.7	21	17	49	38
**Louisiana**[2]	889	896	834	582	603	1,170	1,177	1,384	1,376
**Texas**	20	54	45	1.5	6.2	175	164	312	303
**California**	325	136	123	63	37	569	362	994	668
**Oregon**	9.3	1.6	2.0	6.0	0.4	15	6.1	21	12
**Washington**	18	12	5	9.9	3.8	59	41	83	71
**Hawaii**	-	26	15	-	3.0	-	110	-	182
**USA except AK**	-	2,638	1,982	-	815	-	7912	-	12,272
**Contiguous**	3,683	2,613	1,967	952	812	7,710	7,801	12,112	12,091
Building Based Contiguous[3]			1,967		670		6,951		11,246

Notes

1. From Strauss et al., 2012, Table A.4. Elevations are relative to NOAA's mean higher high water for the National Tidal Datum Epoch of 1983-2001. Population based on the 2010 Census.
2. This column presents our reported results for 2010 population with 1990 sea level, also depicted in Figure 4 of the main letter. See Table S2-F note 2.
3. For comparison, this paper's estimates using the preferred building-based density assumption. The Strauss et al. analysis and all other estimates in this table use the uniform density assumption.
4. We did not clip the 2010 census blocks with 0m, 2m, or 3m contours relative to the 1990 sea level, though we did clip the 2020 blocks relative to those contours. Results for "This Paper" are equal to the 2010 population estimated using the 0m, 2m, and 3m contours relative to 2010 sea level, times the ratio of 2020 population below 0m, 2m, and 3m contours relative to 1990 sea level, to the 2020 population below 0m, 2m, and 3m contours relative to 2010 sea level.
5. Atlantic includes all counties in Florida that drain into the Atlantic Ocean, including Monroe. Gulf is the rest of the state.

begin

Table S3-A
Population within 1 and 3 meters above MHHW, assuming building-based density
(thousands of people)

	Below 1m					Below 3m			
State or Locality	1990	2000	2010	2020		1990	2000	2010	2020
**ME**	1.5	1.7	1.5	1.6		13	14	13	14
**NH**	1.8	2.2	2.2	2.5		7	8	8	9
**MA**	62	71	78	94		324	346	385	444
**RI**	1.6	1.8	1.7	1.9		19	19	20	21
**CT**	10.6	11.1	11.9	13.5		87	86	87	91
**NY**	67	77	86	105		854	908	971	1087
**NJ**	91	105	112	130		515	560	575	633
**PA**	2.1	1.4	1.7	1.6		27	23	27	28
**DE**	4.4	6.7	8.1	9.5		22	34	40	48
**DC**	0.2	0.1	0.3	0.5		2	2	3	4
**VA**	27	31	31	36		463	509	543	569
**MD**	21	23	24	25		111	125	132	135
**NC**	26	29	31	32		153	170	192	197
**SC**	33	38	44	52		200	234	279	320
**GA**	11	14	16	19		100	120	139	155
**FLE**[1]	388	444	493	585		3,452	4,118	4,504	4,944
Broward	101	121	137	172		1,044	1,334	1,455	1,624
Miami-Dade	176	193	223	259		1,745	2,019	2,244	2,426
**FLW**[1]	123	143	151	168		909	1,087	1,196	1,350
**AL**	3.5	4.8	5.0	5.9		25	29	29	33
**MS**	2.6	4.1	2.9	3.1		33	39	29	32
**LA**[2]	1008	1027	872	940		1452	1485	1343	1405
Jefferson	387	397	381	392		439	446	425	432
Orleans	424	417	289	333		485	469	334	375
St. Bernard	50	52	27	35		66	66	36	44
**TX**	50	54	53	56		269	292	286	308
**CA**	106	119	129	134		531	591	637	698
**OR**	2.1	2.1	2.0	1.6		11	11	11	12
**WA**	4.8	5.9	5.2	5.8		58	64	66	69
**HI**[3]	11	11	16	18		128	131	159	168
**USA**	2060	2229	2178	2442		9765	11005	11674	12775

Notes
1. FLE includes all counties in Florida that drain into the Atlantic Ocean, including Monroe. Two counties accounting for most of the population are also shown. FLW is the rest of the state.
2. Statewide total. Also shown are the three Parishes with the most significant decline during the 2000s after Hurricane Katrina.
3. Significant errors in the mapping of the 1990 census block boundaries were corrected for the 2000 census; so changes between 1990 and 2000 are likely to reflect that correction as well as actual population changes.

NOTE: "MHHW" and "sea level" are used interchangeably in this table. A sea level rise of one meter would not necessarily submerge all land within one meter above sea level. The purpose of this table is to answer: How many people in each state are vulnerable to sea level rise (i.e., live below one meter or below three meters)?

Table S3-B
Population within 1 and 3 meters above MHHW, assuming uniform density
(thousands of people)

	**Below 1m**					**Below 3m**			
State or Locality	**1990**	**2000**	**2010**	**2020**		**1990**	**2000**	**2010**	**2020**
**ME**	3.9	4.4	3.9	4.4		19	20	19	20
**NH**	2.5	3.2	3.1	4		9	11	11	12
**MA**	80	89	96	117		377	403	433	498
**RI**	5.3	5.6	5.2	6		29	29	28	30
**CT**	19.3	20.5	21.3	25		105	102	103	108
**NY**	101	116	126	148		922	1000	1037	1154
**NJ**	131	151	160	181		570	621	635	695
**PA**	4.9	4.0	3.8	4.9		38	32	33	37
**DE**	7.6	10.6	12.6	15		29	42	49	59
**DC**	0.3	0.2	0.2	0.7		3.6	3.2	3.6	5.1
**VA**	58	68	69	77		571	604	640	672
**MD**	32	37	38	40		132	148	155	160
**NC**	43	49	54	56		175	197	222	229
**SC**	55	59	70	85		224	257	304	360
**GA**	19	25	27	33		113	137	156	177
**FLE**[1]	561	669	742	876		3595	4311	4672	5120
Broward	162	209	241	290		1,079	1,385	1,493	1,665
Miami-Dade	242	278	311	371		1,787	2,084	2,289	2,462
**FLW**[1]	199	242	253	292		992	1203	1313	1493
**AL**	3.9	5.2	5.4	7.2		28	32	31	35
**MS**	6.1	8.5	6.7	7.6		47	54	41	46
**LA**[2]	1070	1098	936	1003		1506	1553	1400	1468
Jefferson	394	404	386	396		444	450	427	434
Orleans	437	434	300	343		496	484	343	383
St. Bernard	53	55	29	36		66	67	36	44
**TX**	58	63	63	71		295	325	318	347
**CA**	123	135	142	154		592	636	680	748
**OR**	1.8	1.8	1.6	1.9		11.8	12.3	12.6	14.2
**WA**	15	13	13	14		68	71	71	76
**HI**[3]	27	30	28	31		158	166	183	195
**USA**	2629	2907	2878	3253		10610	11968	12552	13757

Notes
1. FLE includes all counties in Florida that drain into the Atlantic Ocean, including Monroe. Two counties accounting for most of the population are also shown FLW is the rest of the state.
2. Statewide total. Also shown are the three Parishes with the most significant decline during the 2000s after Hurricane Katrina.
3. Significant errors in the mapping of the 1990 census block boundaries were corrected for the 2000 census; so changes between 1990 and 2000 are likely to reflect that correction as well as actual population changes.

Table S3-C

Population Change within 0, 1, 2, and 3 meters above MHHW, assuming building-based density (thousands of people)

	Population 2020					Total Change 1990–2020					Change Population <1m		
State or Locality	<0m	<1m	<2m	<3m		<0m	<1m	<2m	<3m		1990s	2000s	2010s
**ME**	0.3	1.6	6.6	14		-0.1	0.1	1.0	1.3		0.2	-0.2	0.1
**NH**	0.1	2.5	6.1	9		0.1	0.8	1.4	2.0		0.5	-0.1	0.4
**MA**	15	94	297	444		6	31	89	119		9	7	16
**RI**	0.1	1.9	9.2	21		0.0	0.3	1.1	2.4		0.2	-0.1	0.2
**CT**	1.0	13.5	50.5	91		0.2	3.0	5.0	4.1		0.6	0.8	1.6
**NY**	7	105	567	1087		5	38	139	233		10	9	20
**NJ**	5	130	407	633		3	39	75	118		15	7	17
**PA**	0.0	1.6	6.4	28		-0.2	-0.5	-2.4	1.1		-0.7	0.3	-0.1
**DE**	0.2	9.5	27.2	48		0.1	5.1	14.7	25.2		2.3	1.5	1.4
**DC**	0.2	0.5	1.4	4		0.1	0.3	0.8	1.5		-0.1	0.2	0.2
**VA**	1	36	203	569		-1	9	48	106		4	0	5
**MD**	1	25	78	135		-1	5	14	23		2	1	1
**NC**	1	32	109	197		0	6	23	44		2	3	1
**SC**	10	52	184	320		2	19	73	120		5	6	8
**GA**	2	19	79	155		1	8	27	55		3	1	4
**FLE**[1]	22	585	3404	4944		7	197	1154	1492		56	49	92
**FLW**[1]	11	168	766	1350		1	44	216	441		20	8	16
**AL**	2.1	5.9	18.1	33		0.6	2.4	6.5	8.0		1.4	0.1	1.0
**MS**	0.5	3.1	11.7	32		-0.2	0.5	0.4	-1.1		1.5	-1.2	0.2
**LA**[2]	662	940	1207	1405		-51	-68	-70	-47		19	-155	68
Jefferson		392		432			5		-7		10	-16	11
Orleans		333		375			-91		-110		-8	-127	44
St. Bernard		35		44			-16		-22		1	-24	8
**TX**	8	56	172	308		3	6	28	40		4	-1	4
**CA**	33	134	375	698		4	29	78	167		14	10	5
**OR**	0.8	1.6	5.8	12		-0.6	-0.5	0.0	1.3		0.0	-0.1	-0.4
**WA**	1.5	5.8	35.2	69		0.2	1.0	4.9	11.5		1.1	-0.7	0.6
**HI**[3]	1	18	97	168		-1	6	30	40		0	4	2
**USA**	787	2442	8124	12775		-21	382	1956	3011		169	-51	263
**USA** except LA	125	1502	6917	11370		29	450	2026	3057		151	104	195

Notes

1. FLE includes all counties in Florida that drain into the Atlantic Ocean, including Monroe. FLW is the rest of the state.
2. Statewide total. Also shown are the three Parishes with the most significant decline during the 2000s after Hurricane Katrina
3. Significant errors in the mapping of the 1990 census block boundaries were corrected for the 2000 census; so changes between 1990 and 2000 are likely to reflect that correction as well as actual population changes.

The purpose of this table is to answer: How did the population below sea level (or below 1, 2, or 3 meters) change?

Table S3-D
Population Change within 0, 1, 2, and 3 meters above MHHW, assuming uniform density
(thousands of people)

	**Population 2020**					**Total Change 1990–2020**					**Change Population <1m**		
State or Locality	**<0m**	**<1m**	**<2m**	**<3m**		**<0m**	**<1m**	**<2m**	**<3m**		**1990s**	**2000s**	**2010s**
**ME**	1.2	4.4	12	20		0.2	0.5	0.6	0.8		0.5	-0.4	0.5
**NH**	0	4	8	12		0.2	1.0	1.8	2.2		0.7	-0.1	0.5
**MA**	21	117	345	498		7	36	93	120		8	7	21
**RI**	0.8	6	16	30		-0.1	0.4	-0.8	0.9		0.3	-0.4	0.5
**CT**	3.8	25	67	108		1.6	5.3	3.9	2.5		1.2	0.8	3.3
**NY**	11	148	632	1154		4	46	142	232		14	10	22
**NJ**	13	181	467	695		5	50	87	125		20	9	21
**PA**	1.7	4.9	12	37		0.6	0.0	-3.2	-1.5		-0.9	-0.2	1.1
**DE**	1.4	15	36	59		0.5	7.3	18.4	29.6		3.1	2.0	2.3
**DC**	0.3	0.7	1.4	5.1		0.1	0.4	0.4	1.5		-0.1	0.0	0.5
**VA**	9	77	280	672		3	19	44	101		10	1	7
**MD**	4	40	99	160		1	8	17	28		5	1	2
**NC**	4	56	140	229		2	13	33	55		6	5	2
**SC**	18	85	225	360		7	31	88	136		4	11	16
**GA**	5	33	98	177		3	13	34	64		6	2	6
**FLE**[1]	84	876	3622	5120		29	315	1191	1525		108	73	134
**FLW**[1]	40	292	909	1493		10	93	279	501		43	11	39
**AL**	2.0	7.2	20	35		1.2	3.3	6.3	7.2		1.3	0.2	1.8
**MS**	0.9	7.6	21	46		0.1	1.5	0.1	-1.4		2.3	-1.8	0.9
**LA**[2]	703	1003	1267	1468		-54	-67	-64	-38		29	-163	67
Jefferson		396		434			2		-10		10	-18	10
Orleans		343		383			-94		-113		-4	-134	43
St. Bernard		36		44			-17		-23		2	-26	8
**TX**	10	71	200	347		4	13	35	51		5	0.5	7
**CA**	40	154	406	748		4	30	78	156		11	7	12
**OR**	0.5	1.9	7.0	14.2		0.0	0.1	0.9	2.3		-0.1	-0.2	0.3
**WA**	5	14	44	76		-2	0	3	8		-1	-1	2
**HI**[3]	4	31	122	195		-3	4	29	38		3	-2	4
**USA**	984	3253	9056	13757		24	624	2117	3146		278	-29	374
**USA** except LA	281	2250	7790	12289		78	691	2181	3184		250	134	308

Notes
1. FLE includes all counties in Florida that drain into the Atlantic Ocean, including Monroe. FLW is the rest of the state.
2. Statewide total. Also shown are the three Parishes with the most significant decline during the 2000s after Hurricane Katrina
3. Significant errors in the mapping of the 1990 census block boundaries were corrected for the 2000 census; so changes between 1990 and 2000 are likely to reflect that correction as well as actual population changes.

	Table S3-E<br>Apparent Migration into Land Below 1 meter relative to the MHHW of 2020<br>(assuming building-based density, (thousands of people)									
	**Population Below 1m**						**Changes**			
State or Locality	**1990**	**2000**	**2010**	**2020**		**1990s**	**2000s**	**2010s**		**2020**
**ME**	1.6	1.8	1.5	1.6		0.2	-0.3	0.1	0.0	-0.2
**NH**	2.0	2.4	2.2	2.5		0.4	-0.2	0.3	0.6	0.1
**MA**	71	77	81	94		6.2	3.8	12.8	22.8	16.6
**RI**	2	2	2	2		0.1	-0.2	0.1	0.0	-0.1
**CT**	13	12	13	14		-0.1	0.1	0.9	1.0	1.1
**NY**	88	92	94	105		3.5	1.8	11.5	16.8	13.3
**NJ**	118	125	121	130		6.9	-3.4	8.3	11.9	5.0
**PA**	2.8	1.7	1.9	1.6		-1.1	0.2	-0.3	-1.2	-0.1
**DE**	5.3	7.6	8.8	9.5		2.3	1.1	0.7	4.2	1.8
**DC**	25	27	26	25		1.3	-0.5	-0.7	0.2	-1.1
**VA**	0.2	0.1	0.3	0.5		-0.1	0.2	0.2	0.3	0.4
**MD**	36	38	34	36		1.8	-3.1	1.4	0.1	-1.7
**NC**	32	33	34	32		0.6	0.6	-1.5	-0.2	-0.9
**SC**	38	42	47	52		4.2	4.2	5.3	13.8	9.6
**GA**	13	16	16	19		2.6	0.7	3.0	6.3	3.8
**FLE**[1]	468	505	526	585		37.0	21.1	59.1	117.2	80.2
Broward	134	146	150	172		12	4	22	39	27
Miami-Dade	206	216	236	259		10	20	23	53	43
**FLW**[1]	150	164	162	168		13.4	-1.7	5.8	17.4	4.1
**AL**	3.9	5.2	5.2	5.9		1.3	-0.1	0.7	2.0	0.7
**MS**	3.4	4.9	3.1	3.1		1.5	-1.7	0.0	-0.3	-1.8
**LA**[2]	1,065	1,066	891	940		0.5	-174.5	48.3	-125.7	-126.2
Jefferson	400	406	385	392		6	-21	7	-8	-14
Orleans	434	423	292	333		-11	-131	41	-101	-90
St. Bernard	53	53	28	35		0.3	-26	7	-18	-19
**TX**	62	63	57	56		1.0	-5.7	-0.9	-5.6	-6.6
**CA**	113	125	132	134		11.6	7.2	2.5	21.3	9.7
**OR**	2.1	2.1	2.0	1.6		0.0	-0.1	-0.4	-0.5	-0.5
**WA**	5.0	6.1	5.3	5.8		1.1	-0.8	0.5	0.8	-0.3
**HI**[3]	13	12	17	18		-0.3	4.2	1.1	5.0	5.3
**USA**	2,334	2,429	2,282	2,442		95.8	-146.9	159.1	107.9	12.2

Notes
1. FLE includes all counties in Florida that drain into the Atlantic Ocean, including Monroe. Two counties accounting for most of the population are also shown. FLW is the rest of the state.
2. Significant errors in the mapping of the 1990 census block boundaries were corrected for the 2000 census; so changes between 1990 and 2000 are likely to reflect that correction as well as actual population changes.

The purpose of this table is to answer: How many people moved into (or out of) the land vulnerable to sea level rise (i.e., below one meter) in each state?

Table S4-A

Population within Mapped[1] 100- and 500-year Coastal[2] Floodplains by State, assuming building-based density (thousands of people)

	A-Zone					X500 Zone			
State	1990	2000	2010	2020		1990	2000	2010	2020
**ME**	1.7	1.7	1.7	1.8		0.3	0.4	0.3	0.3
**NH**	3.5	4.2	3.8	4.4		0.8	0.9	0.9	0.9
**MA**	86	94	96	114		31	33	35	39
**RI**	18	18	16	17		27	26	25	26
**CT**	6	6	6	6		10	9	9	9
**NY**	352	366	387	424		262	278	294	329
**NJ**	263	284	288	328		117	123	128	135
**PA**	12	10	12	11		15	13	15	17
**DE**	11	16	19	22		5	8	7	8
**DC**	2.8	2.7	3.2	3.7		4.3	4.5	4.7	7.9
**VA**	120	128	123	128		124	128	135	136
**MD**	34	35	35	37		17	17	17	19
**NC**	94	102	113	112		42	50	58	60
**SC**	6	7	11	16		4	5	9	12
**GA**	39	46	49	54		52	62	70	78
**FL**	1473	1699	1857	2062		1833	2256	2445	2706
**AL**	24	27	25	28		14	15	14	16
**MS**	75	83	71	74		37	41	46	45
**LA**[2]	424	452	434	460		500	495	395	425
**TX**	126	141	139	153		132	157	178	198
**CA**	196	212	212	236		391	431	449	503
**OR**	11	12	12	13		4	7	9	12
**WA**	23	25	24	27		4	6	6	7
**HI**[3]	31	31	49	51		14	13	17	18
**USA**	3434	3803	3987	4382		3637	4178	4367	4805

Notes

1. Based on National Flood Hazard Layer, which covers about 90% of US population, and is based on National Flood Insurance Rate maps. https://www.fema.gov/flood-maps/national-flood-hazard-layer. The effective dates of those maps vary.
2. Coastal floodplains are defined by clipping the portion of all census blocks within a floodplain, and designating the floodplain part of the block as coastal if any land in that block is below the highest base flood elevation in a given state.
3. Significant errors in the mapping of the 1990 census block boundaries were corrected for the 2000 census; so changes between 1990 and 2000 are likely to reflect that correction as well as actual population changes.

The purpose of this table is to answer: How many people moved into (or out of) coastal floodplains in each state?

Table S4-B

Population within Mapped[1] 100- and 500-year Coastal[2] Floodplains by State, assuming uniform density

(thousands of people)

State	**1990**	**2000**	**2010**	**2020**		**1990**	**2000**	**2010**	**2020**
**ME**	3.8	3.6	3.4	3.5		0.5	0.5	0.5	0.5
**NH**	4.3	5.0	4.7	5.3		0.7	0.8	0.9	0.9
**MA**	107	118	119	139		34	37	39	44
**RI**	25	23	22	22		26	26	25	26
**CT**	11.5	8.1	8.0	7.7		9.3	8.8	8.9	8.6
**NY**	385	408	424	465		274	290	303	341
**NJ**	296	320	329	369		119	126	129	137
**PA**	20	15	16	18		17	15	16	17
**DE**	17	23	26	29		6	8	8	9
**DC**	4.1	3.9	4.0	4.6		5.3	5.4	4.8	8.4
**VA**	229	231	227	232		134	125	137	136
**MD**	49	52	52	56		17	18	17	19
**NC**	112	124	137	136		42	50	57	60
**SC**	10	12	17	26		4	5	9	12
**GA**	51	61	67	74		50	60	68	76
**FL**	1684	1987	2173	2416		1891	2332	2522	2801
**AL**	29	32	29	31		14	15	15	16
**MS**	86	94	81	85		35	40	43	43
**LA**[2]	550	584	551	592		468	469	375	402
**TX**	154	173	168	192		125	149	170	195
**CA**	230	249	243	271		393	433	451	506
**OR**	14	16	16	18		4	7	8	11
**WA**	32	30	30	34		4	6	6	7
**HI**[3]	49	51	67	66		14	15	18	18
**USA**	4152	4624	4814	5292		3688	4242	4432	4894

Notes

1. Based on National Flood Hazard Layer, which covers about 90% of US population, and is based on National Flood Insurance Rate maps. https://www.fema.gov/flood-maps/national-flood-hazard-layer. The effective dates of those maps vary. .
2. Coastal floodplains are defined by clipping the portion of all census blocks within a floodplain, and designating the floodplain part of the block as coastal if any land in that block is below the highest base flood elevation in a given state.
3. Significant errors in the mapping of the 1990 census block boundaries were corrected for the 2000 census; so changes between 1990 and 2000 are likely to reflect that correction as well as actual population changes.

Table S4-C
Population within FEMA Mapped[1] 100-year and 500-year Coastal Floodplains, the Coastal Velocity Zone, and Land Protected from Flooding by Dikes or Levees, assuming building-based density
(thousands of people)

	**2020**				**Total Change 1990–2020**				**Change in A-Zone Population by Decade**		
State	A-zone	X500	V-zone	Dike/ Levee[2]	A-zone	X500	V-zone	Dike/ Levee[2]	**1990s**	**2000s**	**2010s**
**ME**	1.8	0.3	0.8	0.0	0.0	0.0	0.1	0.0	-0.1	0.0	0.1
**NH**	4.4	0.9	0.0	0.0	0.9	0.1	0.0	0.0	0.7	-0.4	0.6
**MA**	114	39	12	10	28	8	2	1	8	1	19
**RI**	17	26	2	3	0	-1	0	3	0.3	-1.5	0.7
**CT**	6	9	1	0.3	0	-1	0	0.1	-0.5	0.0	0.0
**NY**	424	329	6	0	71	67	1	0	14	21	36
**NJ**	328	135	1	0	64	18	0	0	21	3	40
**PA**	11	17	0	0	-1	2	0	0	-2	2	-1
**DE**	22	8	1	0	11	3	0	0	5	3	3
**DC**	3.7	7.9	0.0	2.9	0.9	3.6	0.0	-0.2	-0.1	0.4	0.5
**VA**	128	136	2	1	8	12	0	0	8	-5	4
**MD**	37	19	0	2	3	3	0	1	2	0	2
**NC**	112	60	5	0	18	17	1	0	9	11	-1
**SC**	16	12	0	0	10	8	0	0	2	3	5
**GA**	54	78	0	0	15	26	0	0	7	3	4
**FL**	2062	2706	72	0	588	874	16	0	226	159	204
**AL**	28	16	2	0	4	2	0	0	3	-1	2
**MS**	74	45	3	0	-1	9	-2	0	7	-11	3
**LA**	460	425	6	661	36	-75	-2	53	27	-17	26
**TX**	153	198	9	37	27	66	2	0	15	-2	14
**CA**	236	503	2	1033	40	112	0	244	15	0	24
**OR**	13	12	0	9	2	8	0	4	0.4	-0.2	1.6
**WA**	27	7	1	51	4	2	0	6	1	0	3
**HI[3]**	51	18	9	8	20	4	2	1	0	18	2
**USA**	4382	4805	136	1819	948	1169	20	312	369	185	395
**USA** except FL	2321	2099	64	1819	360	295	4	312	143	26	191

Notes
1. Based on National Flood Hazard Layer, which covers about 90% of US population, and is based on National Flood Insurance Rate maps. https://www.fema.gov/flood-maps/national-flood-hazard-layer. The effective dates of those maps vary.
2. Based on areas the NFHL designates as "Area with Reduced Flood Risk Due to Levee". The meaning of this designation varies by state. In some states it includes protection from dikes as well as levees.
3. Significant errors in the mapping of the 1990 census block boundaries were corrected for the 2000 census; so changes between 1990 and 2000 are likely to reflect that correction as well as actual population changes.

The purpose of this table is to answer: How many people are vulnerable to flooding in each coastal state?

Table S4-D
Population within 100-year and 500-year Coastal Floodplains, the Coastal Velocity Zone, and Land Protected from Flooding by Dikes or Levees, assuming uniform population density
(thousands of people)

	(1)	(2)	(3)	(4)	(5)	(6)	(7)	(8)	(9)		(10)
	2020				Total Change 1990–2020				Change Population in A Zone		
State or Locality	A-zone	X500	V-zone	Dike/ Levee	A-zone	X500	V-zone	Dike/Levee	**1990s**	**2000s**	**2010s**
**ME**	3.5	0.5	1.1	0.0	-0.3	0.0	-0.1	0.0	-0.2	-0.2	0.1
**NH**	5.3	0.9	0.1	0.0	1.0	0.2	0.0	0.0	0.8	-0.3	0.6
**MA**	139	44	17	10	32	9	3	0	11	1	20
**RI**	22	26	5	4	-2	-1	0	3	-1	-2	0
**CT**	7.7	8.6	1.3	0.4	-3.8	-0.7	-0.8	0.1	-3.4	-0.2	-0.3
**NY**	465	341	10	0	80	66	2	0	23	17	40
**NJ**	369	137	3	0	73	18	-3	0	25	9	40
**PA**	18	17	0	0	-3	0	0	0	-6	1	2
**DE**	29	9	1	0	13	3	0	0	6	3	4
**DC**	4.6	8.4	0	3.5	0.5	3.1	0	0.4	-0.2	0.1	0.6
**VA**	232	136	9	1	4	3	-2	0	2	-3	5
**MD**	56	19	1	2	7	2	0	0	4	0	4
**NC**	136	60	6	0	25	18	1	0	13	13	-1
**SC**	26	12	0	0	16	8	0	0	2	5	9
**GA**	74	76	1	0	23	26	0	0	10	5	8
**FL**	2416	2801	88	0	731	910	14	0	303	186	243
**AL**	31	16	2	0	2	2	0	0	3	-4	2
**MS**	85	43	5	0	-1	8	-3	0	8	-13	4
**LA**	592	402	8	599	42	-67	-2	53	34	-32	40
**TX**	192	195	12	37	38	70	2	0	19	-5	23
**CA**	271	506	6	1046	42	113	0	261	19	-5	28
**OR**	18	11	1	9	4	7	0	4	2	0	2
**WA**	34	7	2	51	1	3	0	6	-2	0	4
**HI**[3]	66	18	11	8	17	4	-2	1	2	16	-1
**USA**	5292	4894	190	1770	1141	1206	9	329	472	190	478
**USA** except FL	2877	2093	102	1770	409	296	-5	329	169	4	236

Notes
1. Based on National Flood Hazard Layer, which covers about 90% of US population, and is based on National Flood Insurance Rate maps. https://www.fema.gov/flood-maps/national-flood-hazard-layer. The effective dates of those maps vary.
2. Based on areas the NFHL designates as "Area with Reduced Flood Risk Due to Levee". The meaning of this designation varies by state. In some states it includes protection from dikes as well as levees.
3. Significant errors in the mapping of the 1990 census block boundaries were corrected for the 2000 census; so changes between 1990 and 2000 are likely to reflect that correction as well as actual population changes.

Table S4-E: Population within FEMA Mapped[1] 100-year and 500-year Inland[2] Floodplains, assuming building-based density (thousands of people)

	A-Zones				X500			
State	1990	2000	2010	2020	1990	2000	2010	2020
**ME[3,6]**	6	6	6	7	3	3	3	3
**NH**	19	21	21	22	13	14	14	15
**MA**	42	43	44	49	51	52	54	59
**RI**	5	5	5	6	4	4	4	5
**CT[4]**	1	1	2	2	1	1	1	1
**NY[6]**	92	92	90	92	95	96	96	97
**NJ**	81	84	86	93	71	75	75	81
**PA**	219	211	202	203	137	132	132	133
**DE**	3	3	4	4	2	2	2	3
**DC**	2	2	2	2	1	0	0	0
**VA**	61	64	63	68	29	30	33	36
**MD**	17	17	18	21	12	14	16	15
**NC**	76	79	86	88	48	51	58	58
**SC[3]**	28	29	30	31	10	10	12	13
**GA**	101	111	117	121	49	58	61	67
**FL**	174	227	291	342	64	79	97	110
**AL**	102	101	98	98	43	42	46	47
**MS**	134	135	128	116	77	79	77	69
**LA[3]**	187	208	230	230	112	111	116	116
**TX[4]**	597	689	730	792	605	720	845	949
**CA**	496	561	600	621	2799	3248	3546	3748
**OR[5,6]**	60	68	72	80	74	92	105	116
**WA[5,6]**	28	35	37	40	17	22	25	27
**HI**	7	7	8	8	4	4	6	6
**VT[6]**	11	11	11	12	5	5	5	5
**OH**	178	167	166	166	97	95	96	98
**IN**	110	112	114	121	73	79	87	92
**MI**	123	123	119	120	89	90	88	90
**IL**	134	136	134	127	136	135	138	137
**WI**	52	54	54	57	51	56	56	57
**MN[6]**	20	23	25	26	37	39	44	50
**WV**	144	134	130	121	84	76	74	70
**KY**	145	147	144	144	49	50	52	52
**TN**	83	91	98	106	57	61	66	71
**IA**	46	45	41	43	31	32	29	30
**MO**	70	68	66	65	45	45	48	50
**AR**	89	92	96	93	130	125	120	110
**ND[6]**	18	20	22	29	89	99	108	118
**SD[6]**	17	17	17	17	21	22	21	23
**NE[6]**	47	51	54	58	69	76	78	80
**KS[6]**	48	45	42	42	104	107	109	108
**OK**	75	81	79	80	90	95	96	99
**NM[6]**	78	91	97	96	54	61	61	59
**ID[5]**	18	23	24	30	50	60	65	71
**MT[6]**	21	23	23	23	20	22	24	26
**WY[6]**	6	7	8	7	13	14	15	14
**UT[6]**	12	15	17	19	42	55	63	70
**CO**	43	51	51	53	73	87	97	108
**AZ**	77	109	129	132	2128	3081	3875	4351
**NV**	23	33	39	44	82	147	231	264
**AK[5]**	4	4	5	5	2	2	2	2
**USA**	4227	4573	4775	4971	8039	9753	11173	12077
**Coastal[7]**	2317	2588	2767	2932	4183	4807	5292	5641
**Inland[8]**	1910	1985	2007	2039	3855	4946	5881	6437

Notes

1. Based on National Flood Hazard Layer, which covers about 90% of US population, and is based on National Flood Insurance Rate maps. https://www.fema.gov/flood-maps/national-flood-hazard-layer. The effective dates of those maps vary.

2. Coastal floodplains are defined by clipping the portion of all census blocks within a floodplain, and designating the floodplain part of the block as coastal if any land in that block is below the highest base flood elevation in a given state; all other floodplains are designated as inland.

3. A few important coastal areas are omitted from the NFHL for this state.

4. A large fraction of coastal areas are omitted.

5. The NFHL includes a very small portion of this state.

6. Less than half of this state is in the NFHL

7. All states with a coast along the Atlantic Ocean, Pacific Ocean, or Gulf of Mexico except Alaska; excludes Pennsylvania.

8. All states other than those included as coastal.

The purpose of this table is to answer: How many people are vulnerable to rain-driven (riverine) flooding in each state?

Table S4-F: Population within FEMA Mapped[1] 100-year and 500-year Inland[2] Floodplains, assuming uniform density (thousands of people)

	A-Zones				X500			
State	1990	2000	2010	2020	1990	2000	2010	2020
**ME[3,6]**	13	13	13	14	3	3	3	3
**NH**	37	41	42	43	16	17	17	18
**MA**	96	103	105	115	70	73	75	81
**RI**	12	13	13	14	6	6	6	6
**CT[4]**	4	4	4	4	3	3	3	3
**NY[6]**	173	176	178	181	102	103	104	106
**NJ**	123	132	135	144	79	84	86	92
**PA**	351	353	342	351	143	139	140	142
**DE**	8	9	10	11	3	3	3	3
**DC**	2.7	2.2	2.3	2.2	0.6	0.5	0.5	0.6
**VA**	165	193	205	226	35	40	47	54
**MD**	75	83	88	101	18	20	23	24
**NC**	186	220	248	263	56	60	69	72
**SC[3]**	68	80	89	97	12	13	16	17
**GA**	233	290	334	367	68	84	93	102
**FL**	304	415	532	637	70	88	109	129
**AL**	184	196	195	199	48	48	50	51
**MS**	219	231	225	212	77	79	76	69
**LA[3]**	259	286	311	313	111	111	114	114
**TX[4]**	940	1131	1250	1420	623	746	881	1005
**CA**	621	706	747	789	2861	3328	3624	3848
**OR[5,6]**	84	100	106	118	73	91	104	117
**WA[5,6]**	41	53	57	63	20	26	30	33
**HI**	13	13	11	11	6	6	6	7
**VT[6]**	19	19	19	20	5	5	5	5
**OH**	323	320	314	323	106	105	106	109
**IN**	205	218	223	237	83	92	99	102
**MI**	192	198	193	197	98	100	99	101
**IL**	248	262	264	259	153	156	159	158
**WI**	101	110	109	115	58	63	63	64
**MN[6]**	51	60	63	69	46	49	54	60
**WV**	133	134	134	128	74	69	67	64
**KY**	213	226	228	235	42	43	46	47
**TN**	166	193	208	231	59	64	67	75
**IA**	99	102	97	103	36	38	34	36
**MO**	154	159	162	166	54	57	59	63
**AR**	151	164	170	169	131	126	120	110
**ND[6]**	29	35	40	52	87	93	99	108
**SD[6]**	23	25	25	26	21	22	22	23
**NE[6]**	70	76	79	85	70	79	80	81
**KS[6]**	94	96	94	97	104	109	114	113
**OK**	149	163	165	170	93	99	100	106
**NM[6]**	100	118	125	125	56	62	62	61
**ID[5]**	20	26	29	36	49	60	64	71
**MT[6]**	26	29	30	30	21	24	25	27
**WY[6]**	8	10	11	10	13	14	15	15
**UT[6]**	17	23	27	30	44	58	65	73
**CO**	69	86	89	96	79	95	105	117
**AZ**	99	144	179	189	2134	3092	3870	4449
**NV**	29	45	53	59	82	147	232	273
**AK[5]**	14	14	14	14	3	3	4	4
**USA**	7015	7899	8384	8965	8305	10100	11517	12614
**Coastal[7]**	3862	4491	4899	5344	4359	5034	5540	5955
**Inland[8]**	3153	3408	3485	3621	3946	5066	5977	6659

Notes: 1

Based on National Flood Hazard Layer, which covers about 90% of US population, and is based on National Flood Insurance Rate maps. https://www.fema.gov/flood-maps/national-flood-hazard-layer. The effective dates of those maps vary.

2. Coastal floodplains are defined by clipping the portion of all census blocks within a floodplain, and designating the floodplain part of the block as coastal if any land in that block is below the highest base flood elevation in a given state; all other floodplains are designated as inland.

3. A few important coastal areas are omitted from the NFHL for this state.

4. A large fraction of coastal areas are omitted.

5. The NFHL includes a very small portion of this state.

6. Less than half of this state is in the NFHL

7. All states with a coast along the Atlantic Ocean, Pacific Ocean, or Gulf of Mexico except Alaska; excludes Pennsylvania.

8. All states other than those included as coastal.

Table S4-G: Population Change within FEMA Mapped[1] 100-year and 500-year Inland Floodplains and Land Protected from Flooding by Dikes or Levees, assuming building-based density (thousands of people)

	Population 2020			Change 1990–2020			Inland 100-year			Inland 500-year		
State	A-zone	X500	Dike or Levee	A-zone	X500	Dike or Levee	1990s	2000s	2010s	1990s	2000s	2010s
**ME[3,6]**	7	3	0	0	0	0	-0.2	0.3	0.3	0	0	0
**NH**	22	15	1	3	2	0	2.0	0.2	1.2	1	0	1
**MA**	49	59	24	7	8	-1	1	1	5	1	2	5
**RI**	6	5	0	0	1	0	-0.1	-0.5	0.9	0	0	1
**CT[4]**	2	1	0.0	0	0	0.0	0.0	0.2	0.0	0	0	0
**NY[6]**	92	97	0	0	2	0	0	-1	2	1	0	1
**NJ**	93	81	4	12	10	1	3	3	7	4	0	6
**PA**	203	133	69	-16	-3	-7	-7	-9	1	-5	0	1
**DE**	4	3	0	1	0	0	0	0	0	0	0	0
**DC**	2	0	0	0	0	0	-0.3	0.1	0.2	0	0	0
**VA**	68	36	8	7	8	4	3	-1	6	1	3	3
**MD**	21	15	2	4	3	0	0	0	3	1	3	-1
**NC**	88	58	2	12	10	-1	2	8	2	3	6	0
**SC[3]**	31	13	0	3	3	0	1	1	1	0	2	1
**GA**	121	67	6	21	18	-2	10	6	4	9	3	6
**FL**	342	110	0	168	46	0	54	64	50	16	18	13
**AL**	98	47	5	-3	4	1	0	-4	1	-1	4	1
**MS**	116	69	97	-18	-8	-34	2	-7	-12	2	-2	-8
**LA[3]**	230	116	208	43	4	16	21	22	0	-1	5	0
**TX[4]**	792	949	242	195	344	154	92	41	62	115	125	105
**CA**	621	3748	1564	126	949	301	65	39	21	449	298	201
**OR[5,6]**	80	116	10	21	41	2	8.3	4.3	7.9	18	13	11
**WA[5,6]**	40	27	13	13	10	7	8	2	3	5	3	2
**HI**	8	6	1	1	2	0	0	1	0	0	1	0
**VT[6]**	12	5	0	0	0	0	0	0	0	0	0	0
**OH**	166	98	41	-12	1	-10	-11	-1	0	-2	2	1
**IN**	121	92	62	11	19	-7	2	2	7	7	7	5
**MI**	120	90	0	-3	1	0	-1	-4	1	1	-2	2
**IL**	127	137	27	-6	2	-5	2	-2	-6	-1	3	-1
**WI**	57	57	3	5	6	0	3	0	3	5	0	1
**MN[6]**	26	50	6	6	12	0	2	2	2	2	5	5
**WV**	121	70	17	-22	-14	-4	-9	-5	-9	-8	-2	-4
**KY**	144	52	101	-1	3	-7	2	-3	0	1	3	0
**TN**	106	71	8	23	13	0	8	7	8	4	5	4
**IA**	43	30	72	-3	-1	2	-1	-4	2	0	-3	1
**MO**	65	50	33	-5	5	-2	-2	-2	-1	1	3	2
**AR**	93	110	46	4	-20	-16	4	4	-3	-5	-5	-10
**ND[6]**	29	118	114	11	28	45	2	2	8	10	9	9
**SD[6]**	17	23	17	-1	2	4	0	0	0	1	0	2
**NE[6]**	58	80	32	11	11	4	5	2	4	7	2	2
**KS[6]**	42	108	220	-6	4	-1	-3	-3	-1	3	3	-2
**OK**	80	99	1	5	9	0	6	-1	1	5	1	3
**NM[6]**	96	59	69	17	5	1	12	6	-1	7	0	-2
**ID[5]**	30	71	1	12	21	0	4	2	6	10	5	6
**MT[6]**	23	26	3	2	6	0	2	0	1	2	2	2
**WY[6]**	7	14	4	1	2	1	1	1	0	1	1	0
**UT[6]**	19	70	11	7	28	5	3	3	2	13	8	7
**CO**	53	108	6	10	34	2	8	0	2	14	10	10
**AZ**	132	4351	56	55	2223	36	32	20	2	953	794	476
**NV**	44	264	88	21	183	49	10	6	5	65	84	33
**AK[5]**	5	2	50	2	1	6	0	1	0	0	0	0
**USA**	4971	12077	3343	744	4039	546	346	202	196	1714	1420	905
**Coast[7]**	2932	2932	2186	615	1457	449	272	179	165	624	485	349
**Inlnd[8]**	2039	9145	1157	129	2581	97	75	23	32	1090	935	556

Notes

1. Based on National Flood Hazard Layer, which covers about 90% of US population, and is based on National Flood Insurance Rate maps. https://www.fema.gov/flood-maps/national-flood-hazard-layer. The effective dates of those maps vary.

2. Based on areas the NFHL designates as "Area with Reduced Flood Risk Due to Levee". The meaning of this designation varies by state. In some states it includes protection from dikes as well as levees.

3. A few important coastal areas are omitted from the NFHL for this state.

4. A large fraction of coastal areas are omitted.

5. The NFHL includes a very small portion of this state.

6. Less than half of this state is in the NFHL

7. All states with a coast along the Atlantic Ocean, Pacific Ocean, or Gulf of Mexico except Alaska; excludes Pennsylvania.

8. All states other than those included as coastal.

Table S4-H: Population Change within FEMA Mapped[1] 100-year and 500-year Inland Floodplains and Land Protected from Flooding by Dikes or Levees, assuming uniform density (thousands of people)

	Population 2020			Change 1990–2020			Inland 100-year Decadal Change			Inland 500-year Decadal Change		
State	A-zone	X500	Dike or Levee	A-zone	X500	Dike or Levee	1990s	2000s	2010s	1990s	2000s	2010s
**ME[3,6]**	14	3	0	0.6	-0.2	-0.1	0.4	-0.5	0.7	-0.2	-0.1	0.0
**NH**	43	18	1	5.9	2.6	0.4	4.2	0.0	1.7	1.6	-0.2	1.2
**MA**	115	81	23	18	12	-1	6.3	2.2	10.0	3.6	2.0	6.2
**RI**	14	6	0	2.0	0.5	0.1	0.8	0.1	1.1	0.3	-0.2	0.4
**CT[4]**	4	3	0	0.7	0.3	0.0	0.3	0.2	0.2	0.2	0.0	0.2
**NY[6]**	181	106	0	7.8	3.7	-0.1	3.6	1.2	2.9	1.1	0.6	2.0
**NJ**	144	92	4	21	13	1	9.1	3.2	8.4	5.4	1.2	6.2
**PA**	351	142	68	0.2	-0.6	-7.0	2.1	-11.0	9.0	-3.4	0.6	2.3
**DE**	11	3	0	2.6	0.5	0.0	0.9	0.7	1.0	0.0	0.2	0.3
**DC**	2.2	0.6	0.0	-0.5	0.0	0.0	-0.5	0.1	-0.1	-0.1	0.0	0.1
**VA**	226	54	8	62	18	5	28	12	22	5.1	6.1	7.1
**MD**	101	24	2	26	6	0	8	4	13	1.8	3.2	0.5
**NC**	263	72	1	78	16	-1	35	27	16	4.2	9.2	2.7
**SC[3]**	97	17	0	29	5	0	12	9	8	1.1	2.8	1.3
**GA**	367	102	6	134	34	-2	57	45	32	17	9	9
**FL**	637	129	1	333	59	1	111	117	106	17	22	20
**AL**	199	51	5	15	3	1	12	-1	3	0.6	1.7	1.0
**MS**	212	69	94	-7	-8	-35	12	-6	-13	1.8	-3.3	-6.4
**LA[3]**	313	114	203	54	4	16	27	25	1	-0.1	3.8	-0.2
**TX[4]**	1420	1005	256	479	382	168	190	119	169	123	136	123
**CA**	789	3848	1570	168	988	309	85	41	42	467	296	224
**OR[5,6]**	118	117	10	33	43	2	16	6	11	18	13	13
**WA[5,6]**	63	33	13	22	13	7	11	4	7	6.2	3.2	3.5
**HI**	11	7	1	-1.6	0.7	-0.2	0.1	-1.9	0.3	0.3	0.2	0.3
**VT[6]**	20	5	0	0.6	0.2	0.0	-0.4	0.3	0.7	0.1	0.0	0.2
**OH**	323	109	42	0	3	-13	-2.8	-6.1	8.4	-1.1	0.4	3.6
**IN**	237	102	63	32	19	-7	13	5	14	8.6	7.3	3.3
**MI**	197	101	0	4.9	2.3	0.0	5.3	-4.5	4.2	1.8	-1.0	1.6
**IL**	259	158	27	11	5	-5	14	2	-5	3.6	2.8	-1.1
**WI**	115	64	3	13	7	0	9.1	-1.1	5.3	4.9	0.1	1.5
**MN[6]**	69	60	7	18	14	0	8.8	3.6	5.5	2.7	5.3	5.9
**WV**	128	64	17	-4.9	-10.3	-3.3	1.7	-0.7	-6.0	-4.8	-1.8	-3.7
**KY**	235	47	99	21.8	5.5	-7.2	13	2	7	1.7	2.4	1.3
**TN**	231	75	9	65	16	0	27	15	23	4.3	3.8	7.6
**IA**	103	36	74	3.7	-0.5	2.6	2.9	-5.0	5.8	2.2	-4.2	1.6
**MO**	166	63	32	12	9	-2	4.8	3.1	3.8	2.9	2.2	4.2
**AR**	169	110	45	18	-20	-15	13	6	-1	-4.5	-5.8	-10.1
**ND[6]**	52	108	119	23	21	49	6.3	4.7	11.9	6.3	6.4	8.6
**SD[6]**	26	23	17	3	2	4	2.2	0.3	0.5	0.7	-0.3	1.5
**NE[6]**	85	81	33	15	11	5	6.0	2.7	6.1	8.6	0.8	1.4
**KS[6]**	97	113	227	3	9	0	1.4	-1.5	2.6	4.9	4.6	-0.2
**OK**	170	106	1	20	12	0	14	2	5	5.6	1.3	5.6
**NM[6]**	125	61	70	25	5	1	18	8	0	6.5	0.1	-1.2
**ID[5]**	36	71	1	17	22	0	6.7	2.4	7.6	10.8	4.7	6.5
**MT[6]**	30	27	3	4.7	5.7	0.2	3.5	0.2	1.0	2.6	1.2	1.9
**WY[6]**	10	15	4	1.8	1.6	1.5	1.5	0.7	-0.4	1.3	0.7	-0.4
**UT[6]**	30	73	10	13	29	4	5.9	3.4	3.7	14	7	9
**CO**	96	117	6	27	38	3	17	2	8	16	10	12
**AZ**	189	4449	57	90	2315	37	45	35	9	958	778	578
**NV**	59	273	92	30	191	53	16	9	6	65	84	42
**AK[5]**	14	4	50	0.2	0.7	6.5	-0.1	0.0	0.2	0	0	0
**Coast[7]**	5344	5955	2198	1482	1596	471	629	408	445	675	506	415
**Inland[8]**	3621	6659	1176	467	2713	107	255	77	136	1120	911	683
**USA**	8965	12614	3375	1949	4309	578	884	485	581	1795	1417	1097

Notes

1. Based on National Flood Hazard Layer, which covers about 90% of US population, and is based on National Flood Insurance Rate maps. https://www.fema.gov/flood-maps/national-flood-hazard-layer. The effective dates of those maps vary.

2. Based on areas the NFHL designates as "Area with Reduced Flood Risk Due to Levee". The meaning of this designation varies by state. In some states it includes protection from dikes as well as levees.

3. A few important coastal areas are omitted from the NFHL for this state.

4. A large fraction of coastal areas are omitted.

5. The NFHL includes a very small portion of this state.

6. Less than half of this state is in the NFHL.

7. All states with a coast along the Atlantic Ocean, Pacific Ocean, or Gulf of Mexico except Alaska; excludes Pennsylvania.

8. All states other than those included as coastal.

Table S4-J: Population within FEMA Mapped[1] 100-year and 500-year Floodplains[2], assuming building-based density (thousands of people)

	A-Zones				X500			
State	1990	2000	2010	2020	1990	2000	2010	2020
**ME[3,6]**	8	8	8	8	3	3	3	3
**NH**	22	25	25	26	13	15	15	16
**MA**	128	137	140	163	82	85	89	97
**RI**	23	23	21	23	31	30	30	31
**CT[4]**	8	7	8	8	11	10	10	10
**NY[6]**	444	458	478	516	357	374	390	426
**NJ**	344	368	374	421	188	198	203	216
**PA**	231	221	214	214	152	145	147	150
**DE**	15	20	23	26	7	10	9	11
**DC**	5	4	5	6	5	5	5	8
**VA**	181	192	186	196	153	158	168	173
**MD**	51	53	53	58	29	31	33	35
**NC**	170	181	199	200	90	102	115	117
**SC[3]**	33	36	41	47	14	15	21	25
**GA**	140	157	167	175	101	119	131	145
**FL**	1647	1926	2149	2403	1896	2335	2542	2816
**AL**	126	128	123	126	57	57	60	63
**MS**	209	218	199	190	114	120	122	114
**LA[3]**	611	660	664	690	611	606	511	541
**TX[4]**	723	829	868	945	737	877	1023	1148
**CA**	692	773	812	857	3189	3679	3996	4251
**OR[5,6]**	71	80	84	93	78	99	113	127
**WA[5,6]**	51	60	61	68	21	27	31	34
**HI**	39	38	56	59	17	17	23	24
**VT[6]**	11	11	11	12	5	5	5	5
**OH**	178	167	166	166	97	95	96	98
**IN**	110	112	114	121	73	79	87	92
**MI**	123	123	119	120	89	90	88	90
**IL**	134	136	134	127	136	135	138	137
**WI**	52	54	54	57	51	56	56	57
**MN[6]**	20	23	25	26	37	39	44	50
**WV**	144	134	130	121	84	76	74	70
**KY**	145	147	144	144	49	50	52	52
**TN**	83	91	98	106	57	61	66	71
**IA**	46	45	41	43	31	32	29	30
**MO**	70	68	66	65	45	45	48	50
**AR**	89	92	96	93	130	125	120	110
**ND[6]**	18	20	22	29	89	99	108	118
**SD[6]**	17	17	17	17	21	22	21	23
**NE[6]**	47	51	54	58	69	76	78	80
**KS[6]**	48	45	42	42	104	107	109	108
**OK**	75	81	79	80	90	95	96	99
**NM[6]**	78	91	97	96	54	61	61	59
**ID[5]**	18	23	24	30	50	60	65	71
**MT[6]**	21	23	23	23	20	22	24	26
**WY[6]**	6	7	8	7	13	14	15	14
**UT[6]**	12	15	17	19	42	55	63	70
**CO**	43	51	51	53	73	87	97	108
**`AZ**	77	109	129	132	2128	3081	3875	4351
**NV**	23	33	39	44	82	147	231	264
**AK[5]**	4	4	5	5	2	2	2	2
**USA**	7661	8376	8762	9353	11675	13931	15540	16883
**Coastal[7]**	5739	6381	6743	7303	7805	8972	9644	10429
**Inland[8]**	1922	1994	2019	2050	3870	4959	5896	6454

Notes

1. Based on National Flood Hazard Layer, which covers about 90% of US population, and is based on National Flood Insurance Rate maps. https://www.fema.gov/flood-maps/national-flood-hazard-layer. The effective dates of those maps vary.

2 All estimates in this table are equal to the sum of the corresponding estimates in tables S4A and S4E

3. A few important coastal areas are omitted from the NFHL for this state.

4. A large fraction of coastal areas are omitted.

5. The NFHL includes a very small portion of this state.

6. Less than half of this state is in the NFHL.

7. All states with a coast along the Atlantic Ocean, Pacific Ocean, or Gulf of Mexico except Alaska; excludes Pennsylvania.

8. All states other than those included as coastal.

Table S5-A<br>Black Population within 1 and 3 meters above MHHW, assuming building-based density<br>(thousands of people)										
	**Below 1m**					**Below 3m**				**Black Population**
State or Locality	**1990**	**2000**	**2010**	**2020**		**1990**	**2000**	**2010**	**2020**	**2020**
**ME**	0.0	0.0	0.0	0.0		0.1	0.1	0.3	0.6	25.1
**NH**	0.0	0.0	0.0	0.0		0.1	0.1	0.0	0.0	18.7
**MA**	4.8	4.5	4.7	5.5		26.4	26.9	31.0	32.5	457.1
**RI**	0.0	0.0	0.0	0.0		0.3	0.4	0.4	0.6	55.4
**CT**	1.1	1.2	1.0	1.1		11.7	10.7	10.4	10.2	360.9
**NY**	9.1	12.2	12.6	16.8		138.5	172.4	186.1	204.3	2,759.0
**NJ**	10.0	11.2	11.0	11.7		64.9	69.9	69.0	69.2	1,154.1
**PA**	1.0	0.8	1.0	0.9		5.5	6.0	6.8	6.3	1,369.0
**DE**	0.5	0.4	0.6	0.7		3.8	5.0	5.1	5.4	213.0
**DC**	0.0	0.0	0.1	0.1		1.1	1.2	1.6	1.4	282.1
**VA**	3.3	4.3	4.5	5.4		133.2	154.2	163.6	162.5	1,578.1
**MD**	2.4	2.3	2.2	2.2		13.6	13.5	12.4	12.2	1,795.0
**NC**	5.5	5.4	4.8	3.6		32.7	33.8	33.2	27.7	2,107.5
**SC**	7.4	7.1	6.8	6.0		61.2	60.9	58.5	48.5	1,269.0
**GA**	1.1	1.5	1.6	2.1		26.7	29.3	33.2	33.7	3,278.1
**FLE**[1]	28.9	40.1	51.1	58.5		531.0	721.7	841.6	867.9	3,127.1
**FLW**[1]	3.0	3.9	5.0	5.3		30.7	45.2	59.7	65.1	
**AL**	0.1	0.1	0.1	0.1		3.4	4.1	4.3	4.5	1,288.2
**MS**	0.1	0.2	0.2	0.2		5.6	6.6	6.0	6.9	1,079.0
**LA**[2]	360.9	408.7	316.3	333.8		458.9	513.1	430.3	448.5	1,452.4
Orleans	275.0	294.8	188.4	194.6		297.7	314.0	199.8	202.9	205.9
Jefferson	65.3	89.7	98.7	102.7		76.5	100.0	109.8	113.3	115.9
St. Bernard	1.9	3.7	5.2	9.9		3.1	4.9	6.3	11.3	11.4
**TX**	20.7	19.6	16.1	15.0		55.0	55.2	48.0	48.8	3,444.7
**CA**	4.5	5.1	6.1	5.5		32.9	35.8	41.0	41.2	2,119.3
**OR**	0.0	0.0	0.0	0.0		0.0	0.0	0.1	0.1	78.7
**WA**	0.0	0.2	0.0	0.1		0.3	0.8	0.9	1.1	296.2
**HI**	0.2	0.2	0.2	0.3		2.7	2.3	2.2	2.6	21.9
**USA**[4]	464.6	529.2	446.2	474.9		1640.6	1969.3	2045.7	2101.8	29,629.4

Notes

1. FLE includes all counties in Florida that drain into the Atlantic Ocean, including Monroe. FLW is the rest of the state.
2. Statewide total. Also shown are the three Parishes with the most significant decline during the 2000s after Hurricane Katrina.
3. Significant errors in the mapping of the 1990 census block boundaries were corrected for the 2000 census; so changes between 1990 and 2000 are likely to reflect that correction as well as actual population changes.
4. Coastal states other than Alaska

The purpose of this table is to answer: "How many Black people live on land vulnerable to sea level rise (e.g., below one meter or below three meters) and how has that changed?

Table S5-B<br>Hispanic Population within 1 and 3 meters above MHHW, assuming building-based density<br>(thousands of people)									
	**Below 1m**					**Below 3m**			
State or Locality	**1990**	**2000**	**2010**	**2020**		**1990**	**2000**	**2010**	**2020**
**ME**	0.01	0.02	0.02	0.04		0.08	0.15	0.20	0.37
**NH**	0.02	0.03	0.04	0.06		0.05	0.07	0.13	0.25
**MA**	4.3	7.0	9.9	13.6		23.0	36.5	51.4	66.7
**RI**	0.0	0.0	0.0	0.1		0.3	0.4	0.7	1.3
**CT**	0.71	1.15	1.86	2.40		8.53	10.25	14.86	18.20
**NY**	6.8	10.0	13.4	19.7		122	142	170	207
**NJ**	8.3	12.1	16.5	21.4		52	74	98	123
**PA**	0.08	0.04	0.06	0.08		0.68	0.68	1.67	2.50
**DE**	0.05	0.15	0.28	0.48		0.33	0.78	1.53	2.64
**DC**	0.01	0.00	0.02	0.06		0.07	0.07	0.15	0.30
**VA**	0.36	0.63	1.09	2.15		9.81	15.91	28.69	42.93
**MD**	0.19	0.32	0.70	1.05		1.15	1.81	4.08	6.15
**NC**	0.14	0.47	1.37	1.70		1.35	3.03	7.75	10.15
**SC**	0.46	1.00	1.93	2.58		2.41	7.50	14.29	18.70
**GA**	0.12	0.29	0.82	1.35		1.02	2.87	7.29	10.83
**FLE**[1]	101	132	173	224		990	1433	1903	2286
Broward	6.8	15.9	28.7	48.5		93.7	229.6	374.5	521.6
Miami-Dade	86.6	105.3	130.3	154.9		870.0	1161.6	1461.0	1666.4
**FLW**[1]	3.7	7.0	10.9	15.4		37	79	138	191
**AL**	0.0	0.1	0.1	0.2		0.3	0.3	0.6	1.1
**MS**	0.0	0.1	0.1	0.2		0.4	0.8	1.5	2.5
**LA**[2]	44	48	75	119		55	59	97	152
Orleans	14.3	12.2	14.9	26.4		16.8	14.3	17.4	29.9
Jefferson	24.5	30.0	50.1	74.0		26.2	31.9	53.1	78.0
**TX**	7.2	12.5	17.2	21.2		40	60	80	95
**CA**	15	23	30	33		72	105	143	173
**OR**	0.0	0.1	0.1	0.2		0.2	0.5	0.8	1.2
**WA**	0.2	0.4	0.4	0.7		1.7	4.6	7.8	9.2
**HI**	0.7	0.6	1.0	1.3		8.9	8.7	12.8	15.4
**USA**	194.1	256.8	356.6	481.6		1430	2047	2784	3438

Notes
1. FLE includes all counties in Florida that drain into the Atlantic Ocean, including Monroe. FLW is the rest of the state.
2. Statewide total. Also shown are the three Parishes with the most significant decline during the 2000s after Hurricane Katrina.
3. Significant errors in the mapping of the 1990 census block boundaries were corrected for the 2000 census; so changes between 1990 and 2000 are likely to reflect that correction as well as actual population changes.

The purpose of this table is to answer: "How many Hispanic people live on land vulnerable to sea level rise (e.g. below one meter or below three meters) and how has that changed?

Table S6-A: Population by Race in Various Vulnerable Zones as a Percentage of Total Population in that Vulnerable Zone or Nationwide Population of that Race

Zone	Other Defining Factor	Population of Racial Group in Vulnerable Zone as a percent…							
		of Total Population in Zone[1]				…of Nationwide Population of the same Racial Group[2]			
Black		1990	2000	2010	2020	1990	2000	2010	2020
<1 m	Actual[3]	22.6	23.7	20.5	19.5	1.6	1.6	1.2	1.2
<1 m	Δ Census[4]	21.5	23.0	20.2	19.5	1.7	1.6	1.2	1.2
<1m	Δ Sea Level [5]	20.3	20.0	19.7	19.5	1.1	1.1	1.1	1.2
<3m	Actual	16.8	17.9	17.5	16.5	5.6	5.8	5.4	5.3
<3m	Δ Census	16.9	17.9	17.6	16.5	5.8	5.9	5.5	5.3
<3m	Δ Sea Level	16.4	16.4	16.4	16.5	5.1	5.1	5.2	5.3
<1m	Except New Orleans[6]	11.6	12.9	13.7	13.3	0.7	0.7	0.7	0.7
<3m	Except New Orleans[6]	14.5	15.7	16.3	15.3	4.6	4.9	4.9	4.8
A Zone	All	11.7	12.1	12.0	11.3	3.1	3.0	2.8	2.6
A Zone	Coastal	12.2	12.7	12.2	11.3	1.4	1.4	1.3	1.2
A Zone	Inland	11.3	11.6	11.8	11.5	1.6	1.6	1.5	1.4
X Zone	All	12.1	12.0	11.8	11.4	4.8	4.9	4.9	4.8
Nation		11.7	12.1	12.1	11.9	100	100	100	100
Hispanic									
<1 m	Actual	9.4	11.5	16.4	19.7	0.87	0.73	0.66	0.74
<1 m	Δ Census	9.6	11.7	16.5	19.7	1.00	0.81	0.69	0.74
<1m	Δ Sea Level	19.5	19.6	19.6	19.7	0.63	0.67	0.70	0.74
<3m	Actual	14.6	18.6	23.8	26.9	6.4	5.8	5.1	5.3
<3m	Δ Census	14.5	18.5	23.8	26.9	6.5	5.9	5.2	5.3
<3m	Δ Sea Level	27.2	27.1	27.0	26.9	5.1	5.2	5.2	5.3
A Zone	All	11.1	15.1	19.7	21.1	3.8	3.6	3.2	3.0
A Zone	Coastal	11.6	14.7	19.1	21.2	1.8	1.6	1.4	1.4
A Zone	Inland	10.7	15.6	20.1	20.9	2.0	2.0	1.8	1.6
X Zone	All	17.9	24.7	30.4	29.3	9.4	9.8	8.7	7.6
Nation		9.0	12.5	17.3	19.5	100	100	100	100
All Racial Categories									
<1 m	Actual					0.83	0.79	0.70	0.73
<1 m	Δ Census					0.94	0.86	0.73	0.73
<1m	Δ Sea Level					0.63	0.66	0.70	0.73
<3m	Actual					3.9	3.9	3.7	3.8
<3m	Δ Census					4.1	4.0	3.8	3.8
<3m	Δ Sea Level					3.7	3.7	3.8	3.8
A Zone	All					3.1	3.0	2.8	2.8
A Zone	Coastal					1.4	1.4	1.3	1.3
A Zone	Inland					1.7	1.6	1.5	1.5
X Zone	All					4.7	5.0	5.0	5.0

1. For example, in 2020, Blacks were 19.5 percent of the population living below 1 meter.
2. For example, in 2020, 1.2 percent of the US Black population lived below 1 meter.
3. *Actual* means that elevations are relative to year in column heading and populations are based on Census of that year.
4. *Δ Census* means that the 1m or 3m elevation is relative to sea level of 2020, not the year in the column heading.
5. *Δ Sea Level* means that population based on the Census of 2020, not the year in the column heading. Changes reflect the effect of sea level rise.
6. Same as "actual" except the results exclude New Orleans.

Begin

Table S6-B. Population by Race in Counties with Land in Various Vulnerable Zones as a Percentage of Total Population in those Counties or Nationwide Population of that Race											
Zone	Other Defining Factor	Population of Racial Group in Counties with some Land in the Vulnerable Zone as a percent…									
		of Total Population in those Counties[1]					…of Nationwide Population of the same Racial Group[2]				
Black		1990	2000	2010	2020		1990	2000	2010	2020	
<1 m	Actual	13.7	13.9	13.7	13.1		42	41	39	39	
<3m	Actual	13.8	13.9	13.8	13.2		43	42	40	40	
<1m	Except New Orleans[3]	13.5	13.6	13.6	13.0		42	40	39	38	
A Zone	All	12.2	12.5	12.6	12.4		96	96	96	96	
A Zone	Coastal	14.1	14.2	14.2	13.7		43	42	41	40	
A Zone	Inland	11.5	11.8	12.0	12.0		86	85	87	89	
X Zone	All	12.1	12.4	12.6	12.5		93	93	93	94	
Nation	Anywhere	11.7	12.1	12.1	11.9		100	100	100	100	
Hispanic											
<1 m	Actual	14.1	18.0	21.9	24.1		57	51	44	43	
<3m	Actual	14.0	17.8	21.8	23.8		57	51	44	44	
A Zone	All	9.1	12.7	16.5	18.9		94	94	88	89	
A Zone	Coastal	14.1	18.1	22.1	24.6		56	51	44	44	
A Zone	Inland	8.4	11.8	15.7	18.3		82	83	79	82	
X Zone	All	9.4	13.0	16.9	19.2		93	94	87	88	
Nation	Anywhere	9.0	12.5	17.3	19.5		100	100	100	100	
All Racial Categories											
<1 m	Actual						36	35	35	35	
<3m	Actual						37	36	35	36	
A Zone	All						93	93	92	92	
A Zone	Coastal						35	35	35	35	
A Zone	Inland						88	88	87	88	
X Zone	All						90	90	89	90	

1. For example, in 2020, Blacks people were 13.1 percent of the population living in counties with at least some land below 1 meter. Estimates reflect both sea level rise and population shifts.
2. For example, in 2020, 39 percent of the Nation's Black population lived in a county with land below 1 meter.
3. Same as "actual" except the results exclude New Orleans.

Table S6-C: Cause of Nationwide Disproportionality of Black Residents below One Meter—Total and Black Population in Entire Counties and Below One Meter by County, 2020, ranked by Fraction of County Population below One Meter

		Population <1m			%Black[2]		Ratio[4] of	% Nationwide Population below 1m		
County and States[1]		Black	All races	As % of total[3]	County	<1m	Dispropor-tionality	This County[5]	Cumulative[6]	% Black of Cumulative[7]
Plaquemines	LA	4,543	21,610	91.9	20.7	21.0	1.02	0.9	0.9	20.7
Jefferson	LA	102,668	391,888	88.9	26.3	26.2	1.00	16.1	16.9	26.0
Orleans	LA	194,603	333,352	86.8	53.6	58.4	1.09	13.7	30.6	38.5
St. Bernard	LA	9,865	34,743	79.4	26.0	28.4	1.09	1.4	32.0	37.9
Monroe	FL	3,495	57,820	69.8	5.5	6.0	1.11	2.4	34.4	35.1
Hyde	NC	630	3,036	66.2	25.1	20.7	0.83	0.1	34.5	35.1
Tyrrell	NC	623	2,030	62.5	28.8	30.7	1.07	0.1	34.6	35.1
St. Charles	LA	4,434	23,875	45.4	24.8	18.6	0.75	1.0	35.6	34.5
Lafourche	LA	3,117	42,766	43.8	14.9	7.3	0.49	1.8	37.3	32.9
St. Mary	LA	4,971	21,632	43.8	30.3	23.0	0.76	0.9	38.2	32.7
Terrebonne	LA	5,777	38,894	35.5	19.2	14.9	0.77	1.6	39.8	31.6
Poquoson	VA	29	3,835	30.8	0.9	0.8	0.89	0.2	40.0	31.3
Assumption	LA	73	5,934	28.2	28.4	1.2	0.04	0.2	40.2	31.3
Cameron	LA	31	1,523	27.1	1.3	2.0	1.60	0.1	40.3	31.1
Somerset	MD	1,048	6,380	25.9	38.4	16.4	0.43	0.3	40.5	31.3
Mathews	VA	54	1,585	18.6	7.7	3.4	0.44	0.1	40.6	31.1
Cape May	NJ	466	16,124	16.9	3.5	2.9	0.83	0.7	41.2	29.3
Accomack	VA	141	5,010	15.0	25.9	2.8	0.11	0.2	41.5	29.2
Dare	NC	118	5,494	14.9	1.8	2.2	1.17	0.2	41.7	28.6
Salem	NJ	1,811	8,086	12.5	14.0	22.4	1.60	0.3	42.0	28.0
Jefferson	TX	12,940	29,963	11.7	32.7	43.2	1.32	1.2	43.2	28.6
Dorchester	MD	112	3,318	10.2	27.7	3.4	0.12	0.1	43.4	28.6
Beaufort	SC	1,825	18,993	10.2	14.7	9.6	0.65	0.8	44.1	27.4
Glynn	GA	664	8,508	10.1	24.2	7.8	0.32	0.3	44.5	27.2
Miami-Dade	FL	25,776	259,118	9.6	14.0	9.9	0.71	10.6	55.1	19.9
Atlantic	NJ	5,589	24,514	8.9	14.2	22.8	1.60	1.0	56.1	19.6
Broward	FL	24,662	172,398	8.9	26.6	14.3	0.54	7.1	63.2	21.5
St. James	LA	550	1,722	8.5	47.0	31.9	0.68	0.1	63.2	21.6
Worcester	MD	168	4,380	8.3	11.8	3.8	0.33	0.2	63.4	21.5
Currituck	NC	100	2,310	8.2	4.9	4.3	0.88	0.1	63.5	21.5
Carteret	NC	388	5,547	8.2	4.7	7.0	1.48	0.2	63.7	21.3
San Mateo	CA	1,212	60,094	7.9	1.9	2.0	1.05	2.5	66.2	19.4
Beaufort	NC	738	3,484	7.8	22.8	21.2	0.93	0.1	66.4	19.5
Suffolk	MA	3,216	58,996	7.4	17.0	5.5	0.32	2.4	68.8	19.2
Pamlico	NC	109	888	7.2	16.7	12.3	0.74	0.0	68.8	19.2
Charlotte	FL	225	13,214	7.1	4.7	1.7	0.36	0.5	69.3	18.9
Georgetown	SC	613	4,168	6.6	28.5	14.7	0.52	0.2	69.5	19.0
St. Johns	FL	395	17,676	6.5	4.7	2.2	0.47	0.7	70.2	18.6
Camden	NC	42	637	6.2	10.1	6.5	0.65	0.0	70.3	18.6
Charleston	SC	3,261	24,785	6.1	22.5	13.2	0.59	1.0	71.3	18.7
Collier	FL	893	18,350	4.9	6.0	4.9	0.81	0.8	72.0	18.3
Vermilion	LA	114	2,706	4.7	13.7	4.2	0.31	0.1	72.1	18.2

Notes

Notes

1. In this table, "county" includes parishes in Louisiana, City of Baltimore, Maryland, and independent cities in Virginia.
2. Signifies the fraction of all residents identifying as non-Hispanic Black.
3. Percent of County residents who live below 1m.
4. Ratio of the percentage Black <1m and the percentage Black of county population.
5. The percentage of nationwide residents living below one meter in this county alone.
6. The percentage of nationwide residents living below one meter who live either in this county or counties listed above this county in this table.
7. The number of Black residents as a percentage of all residents, for this county and counties listed above this county in the table. For example,1.2 million people in the first ten our counties, of whom 32.7% are Black.

Table S6-C: Cause of Nationwide Disproportionality of Black Residents below One Meter—Total and Black Population in Entire Counties and Below One Meter by County, 2020, ranked by Fraction of County Population below One Meter

		Population <1m			%Black[2]		Ratio[4] of	% Nationwide Population below 1m			% Black of
County and States[1]		Black	All races	As % of total[3]	County	<1m	Dispropor-tionality		This County[5]	Cumulative[6]	Cumulative[7]
Pinellas	FL	2,035	43,872	4.6	9.5	4.6	0.49		1.8	73.9	17.5
Gloucester	VA	41	1,767	4.6	7.0	2.3	0.33		0.1	74.0	17.5
Hudson	NJ	1,090	32,919	4.5	9.8	3.3	0.34		1.3	75.4	17.0
Manatee	FL	517	16,818	4.2	7.8	3.1	0.39		0.7	76.0	16.7
Marin	CA	128	10,787	4.1	2.3	1.2	0.51		0.4	76.5	16.4
Pasquotank	NC	527	1,616	4.0	35.3	32.6	0.92		0.1	76.6	16.5
Lee	FL	484	29,829	3.9	7.4	1.6	0.22		1.2	77.8	15.9
Franklin	FL	11	446	3.6	11.3	2.5	0.22		0.0	77.8	15.9
Lancaster	VA	34	389	3.6	27.0	8.8	0.33		0.0	77.8	16.0
St. John the Baptist	LA	647	1,425	3.4	56.7	45.4	0.80		0.1	77.9	16.1
Norfolk	VA	1,922	7,850	3.3	39.3	24.5	0.62		0.3	78.2	16.5
Dixie	FL	2	544	3.2	9.3	0.4	0.04		0.0	78.2	16.5
Sussex	DE	179	7,558	3.2	10.5	2.4	0.23		0.3	78.5	16.4
San Joaquin	CA	3,063	24,267	3.1	7.3	12.6	1.73		1.0	79.5	15.9
Hancock	MS	29	1,433	3.1	8.5	2.0	0.24		0.1	79.6	15.9
Galveston	TX	890	10,455	3.0	12.3	8.5	0.69		0.4	80.0	15.8
Brazoria	TX	1,070	10,955	2.9	14.4	9.8	0.68		0.4	80.5	15.8
Chatham	GA	1,220	8,644	2.9	36.6	14.1	0.39		0.4	80.8	16.1
Sarasota	FL	67	12,160	2.8	3.7	0.6	0.15		0.5	81.3	15.8
Martin	FL	54	4,273	2.7	4.6	1.3	0.27		0.2	81.5	15.7
Queen Anne's	MD	37	1,344	2.7	5.6	2.7	0.49		0.1	81.5	15.7
Ocean	NJ	118	16,855	2.6	2.8	0.7	0.25		0.7	82.2	15.2
St. Tammany	LA	1,868	6,961	2.6	12.8	26.8	2.09		0.3	82.5	15.2
Ascension	LA	210	3,325	2.6	23.9	6.3	0.26		0.1	82.6	15.2
Flagler	FL	40	2,982	2.6	9.1	1.4	0.15		0.1	82.8	15.2
Citrus	FL	35	3,958	2.6	2.5	0.9	0.35		0.2	82.9	15.1
McIntosh	GA	52	281	2.6	28.9	18.4	0.63		0.0	82.9	15.1
Colleton	SC	66	957	2.5	34.7	6.9	0.20		0.0	83.0	15.1
Iberville	LA	6	729	2.4	44.0	0.8	0.02		0.0	83.0	15.2
St. Martin	LA	4	1,244	2.4	29.1	0.4	0.01		0.1	83.1	15.2
St. Lucie	FL	567	7,587	2.3	19.6	7.5	0.38		0.3	83.4	15.3
Walton	FL	21	1,732	2.3	4.4	1.2	0.28		0.1	83.4	15.2
Talbot	MD	27	859	2.3	11.2	3.2	0.28		0.0	83.5	15.2
Nassau	FL	53	1,973	2.2	5.5	2.7	0.49		0.1	83.6	15.2
Northumber-land	VA	14	255	2.2	22.6	5.6	0.25		0.0	83.6	15.2
Hampton	VA	821	2,937	2.1	48.6	28.0	0.58		0.1	83.7	15.4
Nassau	NY	2,541	29,109	2.1	10.5	8.7	0.83		1.2	84.9	15.1
Cowlitz	WA	17	2,275	2.1	0.7	0.8	1.13		0.1	85.0	15.0
Perquimans	NC	39	264	2.0	20.7	14.7	0.71		0.0	85.0	15.0
York	VA	25	1,412	2.0	12.3	1.7	0.14		0.1	85.1	15.0
Putnam	FL	37	1,401	1.9	15.4	2.6	0.17		0.1	85.1	15.0

Notes

1. In this table, "county" includes parishes in Louisiana, City of Baltimore, Maryland, and independent cities in Virginia.
2. Signifies the fraction of all residents identifying as non-Hispanic Black.
3. Percent of County residents who live below 1m.
4. Ratio of the percentage Black <1m and the percentage Black of county population.
5. The percentage of nationwide residents living below one meter in this county alone.
6. The percentage of nationwide residents living below one meter who live either in this county or counties listed above this county in this table.
7. The number of Black residents as a percentage of all residents, for this county and counties listed above this county in the table. For example,1.2 million people in the first ten our counties, of whom 32.7% are Black.

Table S6-C: Cause of Nationwide Disproportionality of Black Residents below One Meter—Total and Black Population in Entire Counties and Below One Meter by County, 2020, ranked by Fraction of County Population below One Meter

		Population <1m			%Black[2]		Ratio[4] of	% Nationwide Population below 1m		
County and States[1]		Black	All races	As % of total[3]	County	<1m	Dispropor-tionality	This County[5]	Cumulative[6]	% Black of Cumulative[7]
Brevard	FL	138	11,535	1.9	9.3	1.2	0.13	0.5	85.6	14.8
Baldwin	AL	44	4,387	1.9	7.8	1.0	0.13	0.2	85.8	14.8
Indian River	FL	85	3,000	1.9	8.2	2.8	0.34	0.1	85.9	14.7
Camden	GA	69	1,019	1.9	17.3	6.8	0.39	0.0	85.9	14.7
Aransas	TX	2	442	1.9	1.0	0.4	0.44	0.0	85.9	14.7
Washington	NC	74	199	1.8	48.6	37.1	0.76	0.0	86.0	14.7
Northampton	VA	19	217	1.8	30.6	8.8	0.29	0.0	86.0	14.7
Volusia	FL	707	9,545	1.7	10.0	7.4	0.74	0.4	86.4	14.6
Monmouth	NJ	585	11,028	1.7	6.1	5.3	0.87	0.5	86.8	14.4
Livingston	LA	64	2,431	1.7	7.9	2.6	0.34	0.1	86.9	14.3
Middlesex	VA	8	179	1.7	14.3	4.4	0.30	0.0	86.9	14.3
Taylor	FL	0	363	1.7	19.3	0.1	0.00	0.0	86.9	14.3
Iberia	LA	98	1,155	1.7	32.9	8.5	0.26	0.0	87.0	14.4
Pasco	FL	178	9,049	1.6	5.6	2.0	0.35	0.4	87.3	14.2
Honolulu	HI	288	16,319	1.6	1.9	1.8	0.93	0.7	88.0	13.7
Palm Beach	FL	497	23,334	1.6	17.1	2.1	0.12	1.0	89.0	13.9
Wakulla	FL	4	488	1.4	12.4	0.7	0.06	0.0	89.0	13.9
Kent	MD	9	276	1.4	14.3	3.4	0.24	0.0	89.0	13.9
Chesapeake	VA	1,332	3,578	1.4	28.4	37.2	1.31	0.1	89.1	14.0
Portsmouth	VA	470	1,379	1.4	52.7	34.1	0.65	0.1	89.2	14.2
Levy	FL	4	557	1.3	8.4	0.6	0.08	0.0	89.2	14.1
Gulf	FL	3	181	1.3	11.9	1.4	0.12	0.0	89.2	14.1
Essex	VA	11	134	1.3	35.3	8.1	0.23	0.0	89.2	14.2
Bergen	NJ	522	11,966	1.3	5.2	4.4	0.84	0.5	89.7	13.8
Pender	NC	11	732	1.2	12.5	1.5	0.12	0.0	89.8	13.8
Suffolk	NY	465	18,223	1.2	7.0	2.5	0.36	0.7	90.5	13.5
Richmond	NY	100	5,807	1.2	9.4	1.7	0.18	0.2	90.7	13.4
New Hanover	NC	42	2,554	1.1	12.0	1.7	0.14	0.1	90.8	13.4
Clatsop	OR	1	462	1.1	0.5	0.3	0.52	0.0	90.9	13.4
Bristol	RI	11	550	1.1	1.4	2.1	1.43	0.0	90.9	13.4
Chowan	NC	9	148	1.1	31.9	5.9	0.19	0.0	90.9	13.4
Nantucket	MA	11	152	1.1	7.1	7.5	1.06	0.0	90.9	13.4
Duval	FL	1,969	10,556	1.1	28.8	18.7	0.65	0.4	91.3	13.9
Brunswick	NC	22	1,430	1.0	8.3	1.5	0.18	0.1	91.4	13.8
St. Mary's	MD	79	1,143	1.0	14.1	6.9	0.49	0.0	91.4	13.8
Plymouth	MA	40	5,227	1.0	8.2	0.8	0.09	0.2	91.7	13.7
Middlesex	MA	1,831	16,068	1.0	5.0	11.4	2.30	0.7	92.3	13.3
Calvert	MD	72	897	1.0	12.8	8.0	0.62	0.0	92.3	13.3
Bryan	GA	30	422	0.9	14.1	7.1	0.50	0.0	92.4	13.3
Hernando	FL	8	1,758	0.9	4.9	0.4	0.09	0.1	92.4	13.3
Fairfield	CT	935	8,628	0.9	10.4	10.8	1.04	0.4	92.8	13.2
Queens	NY	8,109	21,501	0.9	15.9	37.7	2.38	0.9	93.7	13.4

Notes

1. In this table, "county" includes parishes in Louisiana, City of Baltimore, Maryland, and independent cities in Virginia.
2. Signifies the fraction of all residents identifying as non-Hispanic Black.
3. Percent of County residents who live below 1m.
4. Ratio of the percentage Black <1m and the percentage Black of county population.
5. The percentage of nationwide residents living below one meter in this county alone.
6. The percentage of nationwide residents living below one meter who live either in this county or counties listed above this county in this table.
7. The number of Black residents as a percentage of all residents, for this county and counties listed above this county in the table. For example,1.2 million people in the first ten our counties, of whom 32.7% are Black.

Table S6-C: Cause of Nationwide Disproportionality of Black Residents below One Meter—Total and Black Population in Entire Counties and Below One Meter by County, 2020, ranked by Fraction of County Population below One Meter

		Population <1m			%Black[2]		Ratio[4] of Dispropor-tionality	% Nationwide Population below 1m			% Black of Cumulative[7]	Notes
County and States[1]		Black	All races	As % of total[3]	County	<1m			This County[5]	Cumulative[6]		
Matagorda	TX	7	324	0.9	10.3	2.1	0.21		0.0	93.7	13.4	
Skagit	WA	1	1,149	0.9	0.6	0.1	0.20		0.0	93.7	13.3	
Cumberland	NJ	114	1,249	0.8	17.1	9.1	0.53		0.1	93.8	13.3	
Rockingham	NH	10	2,499	0.8	0.7	0.4	0.63		0.1	93.9	13.2	
Virginia Beach	VA	118	3,632	0.8	18.0	3.2	0.18		0.1	94.0	13.3	
New York	NY	1,391	13,388	0.8	11.8	10.4	0.88		0.5	94.6	13.2	
Barnstable	MA	51	1,798	0.8	2.8	2.8	1.02		0.1	94.7	13.2	
Liberty	GA	22	509	0.8	41.8	4.3	0.10		0.0	94.7	13.2	
Bay	FL	55	1,292	0.7	10.0	4.3	0.42		0.1	94.7	13.2	
Westmoreland	VA	16	134	0.7	24.2	12.0	0.50		0.0	94.7	13.2	
Jefferson Davis	LA	58	229	0.7	15.9	25.4	1.60		0.0	94.7	13.2	
Kauai	HI	2	516	0.7	0.5	0.5	0.96		0.0	94.8	13.2	
Jackson	MS	136	1,003	0.7	20.7	13.6	0.66		0.0	94.8	13.2	
Dukes	MA	11	144	0.7	3.9	7.6	1.97		0.0	94.8	0.1	
Jasper	SC	44	195	0.7	33.2	22.7	0.68		0.0	94.8	13.2	
Norfolk	MA	162	4,858	0.7	6.8	3.3	0.49		0.2	95.0	13.1	
Essex	MA	152	5,406	0.7	3.3	2.8	0.84		0.2	95.2	12.9	
Hillsborough	FL	648	9,716	0.7	15.4	6.7	0.43		0.4	95.6	13.0	
Santa Rosa	FL	22	1,227	0.7	5.7	1.8	0.32		0.1	95.7	13.0	
King William	VA	8	116	0.7	14.5	7.1	0.49		0.0	95.7	13.0	
Orange	CA	133	20,016	0.6	1.5	0.7	0.43		0.8	96.5	12.2	
Onslow	NC	29	1,246	0.6	13.2	2.3	0.18		0.1	96.6	12.2	
Gates	NC	7	59	0.6	28.8	12.4	0.43		0.0	96.6	12.2	
Newport	RI	5	474	0.6	3.1	1.1	0.36		0.0	96.6	12.2	
Wicomico	MD	182	569	0.5	26.6	31.9	1.20		0.0	96.6	12.2	
Kings	NY	3,870	14,803	0.5	26.7	26.1	0.98		0.6	97.2	13.0	
Gloucester	NJ	77	1,613	0.5	10.4	4.8	0.46		0.1	97.3	13.0	
Calcasieu	LA	101	1,154	0.5	25.3	8.7	0.35		0.0	97.3	13.0	
Berkeley	SC	101	1,216	0.5	22.5	8.3	0.37		0.0	97.4	13.1	
Clay	FL	42	1,140	0.5	11.7	3.7	0.32		0.0	97.4	13.1	
Calhoun	TX	0	105	0.5	1.8	0.3	0.19		0.0	97.4	13.1	
Burlington	NJ	592	2,101	0.5	16.2	28.2	1.74		0.1	97.5	13.1	
Horry	SC	45	1,586	0.5	11.2	2.9	0.25		0.1	97.6	13.1	
Washington	RI	4	582	0.4	1.1	0.7	0.64		0.0	97.6	13.1	
Middlesex	CT	2	730	0.4	4.9	0.3	0.05		0.0	97.6	13.0	
Nueces	TX	12	1,570	0.4	3.6	0.8	0.22		0.1	97.7	13.0	
Kent	DE	54	801	0.4	25.2	6.8	0.27		0.0	97.7	13.0	
Orange	TX	19	362	0.4	9.4	5.3	0.57		0.0	97.8	13.0	
Maui	HI	5	690	0.4	0.6	0.7	1.16		0.0	97.8	13.0	
Anne Arundel	MD	135	2,350	0.4	17.4	5.7	0.33		0.1	97.9	13.0	
Sagadahoc	ME	3	146	0.4	0.8	1.9	2.34		0.0	97.9	13.0	

Notes

1. In this table, "county" includes parishes in Louisiana, City of Baltimore, Maryland, and independent cities in Virginia.

2. Signifies the fraction of all residents identifying as non-Hispanic Black.

3. Percent of County residents who live below 1m.

4. Ratio of the percentage Black <1m and the percentage Black of county population.

5. The percentage of nationwide residents living below one meter in this county alone.

6. The percentage of nationwide residents living below one meter who live either in this county or counties listed above this county in this table.

7. The number of Black residents as a percentage of all residents, for this county and counties listed above this county in the table. For example,1.2 million people in the first ten our counties, of whom 32.7% are Black.

Table S6-C: Cause of Nationwide Disproportionality of Black Residents below One Meter—Total and Black Population in Entire Counties and Below One Meter by County, 2020, ranked by Fraction of County Population below One Meter

		Population <1m			%Black[2]		Ratio[4] of Dispropor-tionality	% Nationwide Population below 1m		% Black of Cumulative[7]
County and States[1]		Black	All races	As % of total[3]	County	<1m		This County[5]	Cumulative[6]	
New Haven	CT	141	3,383	0.4	12.8	4.2	0.33	0.1	98.0	13.0
Mobile	AL	66	1,527	0.4	35.1	4.3	0.12	0.1	98.1	13.2
Okaloosa	FL	24	768	0.4	8.9	3.2	0.35	0.0	98.1	13.2
Santa Clara	CA	106	6,985	0.4	2.2	1.5	0.70	0.3	98.4	12.8
Escambia	FL	25	1,129	0.4	21.2	2.2	0.11	0.0	98.4	12.8
York	ME	9	739	0.3	0.9	1.2	1.28	0.0	98.5	12.8
Columbia	OR	1	177	0.3	0.6	0.5	0.89	0.0	98.5	12.8
San Patricio	TX	1	228	0.3	1.4	0.3	0.22	0.0	98.5	12.8
Craven	NC	29	314	0.3	19.8	9.1	0.46	0.0	98.5	12.8
Harrison	MS	78	639	0.3	24.5	12.2	0.50	0.0	98.5	12.8
Caroline	MD	11	102	0.3	13.1	10.4	0.79	0.0	98.5	12.8
Kitsap	WA	108	831	0.3	2.7	12.9	4.86	0.0	98.6	12.8
Hendry	FL	1	119	0.3	10.6	0.6	0.05	0.0	98.6	12.8
Baltimore	MD	240	2,549	0.3	29.6	9.4	0.32	0.1	98.7	13.0
Lincoln	ME	1	102	0.3	0.4	1.3	3.03	0.0	98.7	13.0
New London	CT	11	775	0.3	5.4	1.5	0.28	0.0	98.7	13.0
Hertford	NC	10	61	0.3	56.7	17.1	0.30	0.0	98.7	13.0
Contra Costa	CA	202	3,215	0.3	8.4	6.3	0.75	0.1	98.8	12.9
Coos	OR	1	179	0.3	0.4	0.6	1.49	0.0	98.9	12.9
Cecil	MD	7	274	0.3	7.2	2.5	0.35	0.0	98.9	12.9
Tangipahoa	LA	7	341	0.3	29.9	2.2	0.07	0.0	98.9	12.9
Knox	ME	0	102	0.3	0.6	0.2	0.26	0.0	98.9	12.9
Newport News	VA	232	452	0.2	41.3	51.3	1.24	0.0	98.9	13.0
Camden	NJ	400	1,237	0.2	18.2	32.4	1.78	0.1	99.0	13.0
Sacramento	CA	243	3,664	0.2	9.2	6.6	0.72	0.2	99.1	12.9
Chambers	TX	5	106	0.2	6.8	4.3	0.64	0.0	99.1	12.9
Bristol	MA	24	1,224	0.2	4.0	1.9	0.49	0.1	99.2	12.9
Grays Harbor	WA	1	158	0.2	1.3	0.6	0.49	0.0	99.2	12.9
James City	VA	5	157	0.2	12.6	3.2	0.25	0.0	99.2	12.9
New Castle	DE	507	1,134	0.2	24.9	44.7	1.79	0.0	99.2	12.9
Cameron	TX	2	784	0.2	0.3	0.2	0.62	0.0	99.3	12.9
Clallam	WA	0	128	0.2	0.7	0.4	0.49	0.0	99.3	12.9
Lake	FL	1	623	0.2	9.9	0.2	0.02	0.0	99.3	12.8
Humboldt	CA	3	195	0.1	1.3	1.5	1.20	0.0	99.3	12.8
Solano	CA	66	622	0.1	13.2	10.6	0.80	0.0	99.3	12.8
Baltimore	MD	33	796	0.1	57.3	4.2	0.07	0.0	99.4	13.2
Alexandria	VA	26	179	0.1	19.6	14.4	0.73	0.0	99.4	13.2
Ulster	NY	17	201	0.1	5.6	8.5	1.53	0.0	99.4	13.2
Kent	RI	4	187	0.1	1.7	2.3	1.37	0.0	99.4	13.2
Cumberland	ME	4	310	0.1	3.9	1.2	0.31	0.0	99.4	13.1
Essex	NJ	134	868	0.1	37.5	15.5	0.41	0.0	99.4	13.4

Notes

1. In this table, "county" includes parishes in Louisiana, City of Baltimore, Maryland, and independent cities in Virginia.

2. Signifies the fraction of all residents identifying as non-Hispanic Black.

3. Percent of County residents who live below 1m.

4. Ratio of the percentage Black <1m and the percentage Black of county population.

5. The percentage of nationwide residents living below one meter in this county alone.

6. The percentage of nationwide residents living below one meter who live either in this county or counties listed above this county in this table.

7. The number of Black residents as a percentage of all residents, for this county and counties listed above this county in the table. For example,1.2 million people in the first ten our counties, of whom 32.7% are Black.

Table S6-C: Cause of Nationwide Disproportionality of Black Residents below One Meter—Total and Black Population in Entire Counties and Below One Meter by County, 2020, ranked by Fraction of County Population below One Meter

		Population <1m			%Black[2]		Ratio[4] of Dispropor-tionality	% Nationwide Population below 1m			% Black of Cumulative[7]
County and States[1]		Black	All races	As % of total[3]	County	<1m			This County[5]	Cumulative[6]	
Charles	MD	16	166	0.1	48.5	9.6	0.20		0.0	99.4	13.4
Middlesex	NJ	119	831	0.1	9.1	14.3	1.57		0.0	99.5	13.5
Rockland	NY	14	319	0.1	10.0	4.4	0.44		0.0	99.5	13.4
Napa	CA	0	128	0.1	1.7	0.0	0.02		0.0	99.5	13.4
Multnomah	OR	8	655	0.1	5.4	1.2	0.22		0.0	99.5	13.3
Westchester	NY	100	779	0.1	13.0	12.9	0.99		0.0	99.6	13.4
District of Columbia	DC	87	527	0.1	40.9	16.4	0.40		0.0	99.6	13.6
Philadelphia	PA	859	1,176	0.1	38.3	73.1	1.91		0.0	99.6	14.1
Alameda	CA	67	1,155	0.1	9.5	5.8	0.61		0.0	99.7	14.0
Bronx	NY	148	909	0.1	28.5	16.3	0.57		0.0	99.7	14.3
Snohomish	WA	10	509	0.1	3.4	2.0	0.59		0.0	99.7	14.2
Hawaii	HI	1	119	0.1	0.6	1.0	1.72		0.0	99.7	14.2
Yolo	CA	1	127	0.1	2.6	0.8	0.32		0.0	99.8	14.2
Seminole	FL	21	227	0.0	10.7	9.2	0.87		0.0	99.8	14.1
Delaware	PA	19	254	0.0	22.0	7.5	0.34		0.0	99.8	14.1
San Diego	CA	259	1,423	0.0	4.4	18.2	4.15		0.1	99.8	13.8
San Francisco	CA	12	251	0.0	5.2	4.6	0.89		0.0	99.9	13.6
Pierce	WA	9	263	0.0	6.8	3.3	0.48		0.0	99.9	13.6
Union	NJ	40	163	0.0	19.5	24.6	1.26		0.0	99.9	13.6
Stafford	VA	3	37	0.0	18.8	7.6	0.41		0.0	99.9	13.6
Bucks	PA	3	147	0.0	3.9	2.3	0.59		0.0	99.9	13.6
Harris	TX	67	865	0.0	18.7	7.7	0.41		0.0	99.9	13.8
Fairfax	VA	12	177	0.0	9.4	6.7	0.71		0.0	99.9	13.8
Ventura	CA	3	116	0.0	1.6	2.9	1.78		0.0	99.9	13.8
Providence	RI	5	74	0.0	7.2	7.0	0.97		0.0	99.9	13.8
Los Angeles	CA	33	1,095	0.0	7.6	3.0	0.40		0.0	100.0	13.0
King	WA	1	186	0.0	6.5	0.5	0.08		0.0	100.0	13.0

Notes

1. In this table, "county" includes parishes in Louisiana, City of Baltimore, Maryland, and independent cities in Virginia.
2. Signifies the fraction of all residents identifying as non-Hispanic Black.
3. Percent of County residents who live below 1m.
4. Ratio of the percentage Black <1m and the percentage Black of county population.
5. The percentage of nationwide residents living below one meter in this county alone.
6. The percentage of nationwide residents living below one meter who live either in this county or counties listed above this county in this table.
7. The number of Black residents as a percentage of all residents, for this county and counties listed above this county in the table. For example,1.2 million people in the first ten our counties, of whom 32.7% are Black.

The purpose of this table is to illustrate the high proportion of Black residents in localities most vulnerable to sea level rise.

Table S6-D: Cause of Nationwide Disproportionality of Hispanic Residents below Three Meters— Population in Entire Counties and Below One Meter by County, 2020, ranked by Fraction of County Population below Three Meters

		Population <1m			%Hispanic[2]		Ratio[4] of	% Nationwide Population below 1m		Percent	Notes
County and States[1]		Hispanic	All races	As % of total[3]	County	<1m	Dispropor-tionality	his County[5]	Cumulative[6]	Hispanic of Cumulative[7]	
Poquoson	VA	460	12,452	99.9	3.7	3.7	0.99	0.1	0.1	3.7	
St. Bernard	LA	5,956	43,552	99.5	13.7	13.7	1.00	0.3	0.4	11.5	
Jefferson	LA	78,038	432,407	98.1	17.9	18.0	1.01	3.4	3.8	17.2	
Orleans	LA	29,897	375,491	97.8	8.1	8.0	0.99	2.9	6.8	13.2	
Monroe	FL	18,789	80,871	97.6	23.4	23.2	0.99	0.6	7.4	14.1	
Hyde	NC	342	4,456	97.1	7.6	7.7	1.02	0.0	7.4	14.1	
Plaquemines	LA	2,144	22,802	97.0	9.5	9.4	0.99	0.2	7.6	14.0	
Terrebonne	LA	7,124	105,681	96.4	6.7	6.7	1.00	0.8	8.4	13.2	
Tyrrell	NC	263	3,128	96.4	8.4	8.4	1.00	0.0	8.5	13.2	
St. Mary	LA	4,374	45,606	92.3	9.2	9.6	1.05	0.4	8.8	13.1	
Miami-Dade	FL	1,666,358	2,425,592	89.8	68.7	68.7	1.00	19.0	27.8	52.1	
Cameron	LA	170	5,008	89.2	3.5	3.4	0.97	0.0	27.8	52.0	
St. Charles	LA	3,695	46,652	88.8	7.9	7.9	1.00	0.4	28.2	51.4	
Lafourche	LA	5,006	83,693	85.8	5.8	6.0	1.03	0.7	28.9	50.3	
Broward	FL	521,583	1,624,005	83.5	31.3	32.1	1.03	12.7	41.6	44.1	
Camden	NC	229	7,580	73.2	3.3	3.0	0.92	0.1	41.6	44.0	
Pasquotank	NC	1,684	29,102	71.7	5.5	5.8	1.05	0.2	41.9	43.8	
St. John the Baptist	LA	2,677	30,307	71.3	7.7	8.8	1.14	0.2	42.1	43.5	
Mathews	VA	144	5,963	69.9	2.3	2.4	1.04	0.0	42.1	43.5	
Dare	NC	1,760	25,746	69.7	6.9	6.8	0.99	0.2	42.3	43.2	
Currituck	NC	808	18,956	67.5	4.3	4.3	0.98	0.1	42.5	43.1	
Norfolk	VA	13,478	146,702	61.6	9.7	9.2	0.95	1.1	43.6	41.8	
Hampton	VA	4,734	78,656	57.4	6.1	6.0	0.98	0.6	44.3	41.1	
Charlotte	FL	6,957	107,019	57.3	7.5	6.5	0.86	0.8	45.1	40.1	
Portsmouth	VA	2,699	54,032	55.2	4.5	5.0	1.11	0.4	45.5	39.6	
Glynn	GA	3,370	45,765	54.2	7.5	7.4	0.98	0.4	45.9	39.2	
Cape May	NJ	4,308	50,241	52.7	7.8	8.6	1.09	0.4	46.3	38.8	
St. James	LA	176	10,630	52.6	1.7	1.7	0.97	0.1	46.4	38.7	
Charleston	SC	8,215	196,024	48.0	7.2	4.2	0.58	1.5	47.9	36.9	
Worcester	MD	973	24,949	47.6	4.0	3.9	0.98	0.2	48.1	36.7	
Assumption	LA	524	9,948	47.3	4.3	5.3	1.21	0.1	48.2	36.6	
Pamlico	NC	165	5,698	46.4	4.0	2.9	0.72	0.0	48.2	36.6	
Vermilion	LA	1,156	26,309	45.9	4.0	4.4	1.10	0.2	48.4	36.3	
Somerset	MD	497	10,864	44.1	4.4	4.6	1.05	0.1	48.5	36.2	
Salem	NJ	3,525	28,001	43.2	10.1	12.6	1.25	0.2	48.7	36.0	
Collier	FL	32,076	157,544	41.9	27.2	20.4	0.75	1.2	50.0	35.6	
Lee	FL	54,199	317,445	41.7	22.8	17.1	0.75	2.5	52.4	34.5	
Perquimans	NC	106	5,403	41.5	2.4	2.0	0.83	0.0	52.5	34.4	
Carteret	NC	1,033	27,449	40.6	4.6	3.8	0.82	0.2	52.7	34.2	
Beaufort	SC	7,836	71,514	38.2	12.5	11.0	0.88	0.6	53.3	33.7	
Aransas	TX	2,002	8,021	33.7	25.8	25.0	0.97	0.1	53.3	33.7	
Beaufort	NC	774	14,118	31.6	7.7	5.5	0.71	0.1	53.4	33.6	
Galveston	TX	32,675	109,330	31.2	25.3	29.9	1.18	0.9	54.3	33.3	
Jefferson	TX	27,010	78,343	30.5	23.0	34.5	1.50	0.6	54.9	33.0	
Virginia Beach	VA	11,838	140,053	30.5	8.8	8.5	0.96	1.1	56.0	31.9	
Martin	FL	4,847	48,016	30.3	15.3	10.1	0.66	0.4	56.4	31.7	
Orange	TX	2,850	25,486	30.1	8.6	11.2	1.31	0.2	56.6	31.5	
Accomack	VA	430	9,749	29.2	10.3	4.4	0.43	0.1	56.6	31.4	

Notes

1. In this table, "county" includes parishes in Louisiana, City of Baltimore, Maryland, and independent cities in Virginia.

2. Signifies the fraction of all residents identifying as Hispanic.

3. Percent of County residents who live below 1m.

4. Ratio of the percentage Hispanic <1m and the percentage Hispanic of county population.

5. The percentage of nationwide residents living below one meter in this county alone.

6. The percentage of nationwide residents living below one meter who live either in this county or counties listed above this county in this table.

7. The number of Hispanic residents as a percentage of all residents for this county and counties listed above this county in the table. For example, 6 million people live in the first 15 counties in this table, of whom 44% are Hispanic.

Table S6-D: Cause of Nationwide Disproportionality of Hispanic Residents below Three Meters— Population in Entire Counties and Below One Meter by County, 2020, ranked by Fraction of County Population below Three Meters

		Population <1m			%Hispanic[2]		Ratio[4] of	% Nationwide Population below 1m			Percent
County and States[1]		Hispanic	All races	As % of total[3]	County	<1m	Dispropor-tionality		his County[5]	Cumulative[6]	Hispanic of Cumulative[7]
Pinellas	FL	21,795	266,748	27.8	10.7	8.2	0.76		2.1	58.7	29.7
Atlantic	NJ	15,551	75,931	27.7	19.6	20.5	1.05		0.6	59.3	29.4
Washington	NC	168	3,032	27.6	3.4	5.6	1.65		0.0	59.3	29.4
Chesapeake	VA	5,736	66,953	26.8	7.1	8.6	1.20		0.5	59.9	28.9
Franklin	FL	117	3,328	26.7	5.4	3.5	0.65		0.0	59.9	28.9
Grays Harbor	WA	3,648	20,189	26.7	10.4	18.1	1.74		0.2	60.1	28.8
Camden	GA	757	14,608	26.7	6.7	5.2	0.78		0.1	60.2	28.7
Gulf	FL	166	3,772	26.6	3.9	4.4	1.13		0.0	60.2	28.7
Ascension	LA	3,261	33,446	26.4	8.2	9.7	1.19		0.3	60.5	28.4
Suffolk	MA	42,606	210,333	26.4	22.4	20.3	0.91		1.6	62.1	28.1
Bryan	GA	909	11,757	26.3	7.3	7.7	1.06		0.1	62.2	28.0
Chatham	GA	5,382	73,783	25.0	8.1	7.3	0.91		0.6	62.8	27.6
St. Johns	FL	3,973	66,909	24.5	8.3	5.9	0.72		0.5	63.3	27.2
Talbot	MD	285	8,864	23.6	8.9	3.2	0.36		0.1	63.4	27.1
Georgetown	SC	560	14,576	23.0	3.5	3.8	1.10		0.1	63.5	27.0
Volusia	FL	8,443	126,916	22.9	14.9	6.7	0.45		1.0	64.5	26.5
Queen Anne's	MD	503	11,147	22.4	5.1	4.5	0.89		0.1	64.6	26.5
Dorchester	MD	231	6,911	21.2	5.5	3.3	0.61		0.1	64.6	26.4
Hudson	NJ	24,533	153,586	21.2	40.4	16.0	0.40		1.2	65.8	27.1
Iberia	LA	1,272	14,701	21.0	5.6	8.7	1.55		0.1	65.9	27.0
Northumberland	VA	58	2,470	20.9	3.0	2.3	0.79		0.0	66.0	27.0
Indian River	FL	1,972	32,251	20.2	13.0	6.1	0.47		0.3	66.2	26.8
York	VA	687	13,963	19.9	7.3	4.9	0.67		0.1	66.3	26.7
Cowlitz	WA	2,826	21,984	19.9	9.8	12.9	1.32		0.2	66.5	26.6
St. Tammany	LA	4,069	52,390	19.8	7.9	7.8	0.99		0.4	66.9	26.3
Calcasieu	LA	2,111	42,021	19.4	5.3	5.0	0.96		0.3	67.2	26.0
Walton	FL	1,501	14,456	19.2	8.2	10.4	1.26		0.1	67.3	25.9
Nassau	FL	828	17,023	18.8	4.9	4.9	0.99		0.1	67.5	25.8
Gloucester	VA	231	7,197	18.6	3.6	3.2	0.88		0.1	67.5	25.8
McIntosh	GA	35	2,005	18.3	2.1	1.7	0.82		0.0	67.5	0.3
Sarasota	FL	3,397	79,284	18.3	10.0	4.3	0.43		0.6	68.2	25.3
Brevard	FL	7,809	109,654	18.1	11.2	7.1	0.64		0.9	69.0	24.8
Manatee	FL	9,281	71,502	17.9	17.8	13.0	0.73		0.6	69.6	24.7
Flagler	FL	1,194	20,310	17.6	10.7	5.9	0.55		0.2	69.7	24.6
Wahkiakum	WA	32	778	17.6	4.1	4.1	1.00		0.0	69.8	24.6
San Mateo	CA	35,935	131,115	17.2	25.0	27.4	1.09		1.0	70.8	24.6
Northampton	VA	120	1,974	16.1	8.7	6.1	0.70		0.0	70.8	24.6
Marin	CA	15,433	41,114	15.7	18.8	37.5	1.99		0.3	71.1	24.5
Iberville	LA	146	4,565	15.1	4.7	3.2	0.68		0.0	71.2	24.5
Palm Beach	FL	36,684	223,057	14.9	23.5	16.4	0.70		1.7	72.9	24.4
Honolulu	HI	13,298	150,404	14.8	9.1	8.8	0.97		1.2	74.1	23.7
Nassau	NY	31,101	204,256	14.6	18.4	15.2	0.83		1.6	75.7	23.4
Sussex	DE	1,505	34,228	14.4	11.3	4.4	0.39		0.3	75.9	23.2
Ocean	NJ	7,056	91,471	14.4	10.4	7.7	0.74		0.7	76.7	22.9
Bay	FL	1,661	25,040	14.3	7.9	6.6	0.84		0.2	76.9	22.8
Hancock	MS	301	6,466	14.0	4.2	4.7	1.12		0.1	76.9	22.7
San Joaquin	CA	40,274	100,315	12.9	41.8	40.1	0.96		0.8	77.7	23.3
Lancaster	VA	6	1,387	12.7	1.1	0.4	0.38		0.0	77.7	23.3
Bristol	RI	250	6,409	12.6	3.8	3.9	1.02		0.1	77.7	23.3

Notes

Notes

1. In this table, “county” includes parishes in Louisiana, City of Baltimore, Maryland, and independent cities in Virginia.

2. Signifies the fraction of all residents identifying as non-Hispanic Black.

3. Percent of County residents who live below 1m.

4. Ratio of the percentage Black <1m and the percentage Black of county population.

5. The percentage of nationwide residents living below one meter in this county alone.

6. The percentage of nationwide residents living below one meter who live either in this county or counties listed above this county in this table.

7. The number of Hispanic residents as a percentage of all residents for this county and counties listed above this county in the table. For example, 6 million people live in the first 15 counties in this table, of whom 44% are Hispanic.

Table S6-D: Cause of Nationwide Disproportionality of Hispanic Residents below Three Meters— Population in Entire Counties and Below One Meter by County, 2020, ranked by Fraction of County Population below Three Meters

County and States[1]		Population <1m Hispanic	All races	As % of total[3]	%Hispanic[2] County	<1m	Ratio[4] of Dispropor-tionality	% Nationwide Population below 1m his County[5]	Cumulative[6]	Percent Hispanic of Cumulative[7]
Craven	NC	712	12,615	12.5	7.1	5.6	0.79	0.1	77.8	23.2
Citrus	FL	768	19,220	12.5	6.0	4.0	0.67	0.2	78.0	23.1
Kings	NY	44,385	337,809	12.3	18.9	13.1	0.70	2.6	80.6	22.7
Clatsop	OR	580	4,970	12.1	9.4	11.7	1.25	0.0	80.7	22.7
Jefferson Davis	LA	57	3,853	11.9	2.3	1.5	0.65	0.0	80.7	22.7
Chowan	NC	48	1,610	11.7	3.9	3.0	0.77	0.0	80.7	22.6
Duval	FL	8,567	114,057	11.5	11.3	7.5	0.66	0.9	81.6	22.3
Liberty	GA	372	7,417	11.4	11.9	5.0	0.42	0.1	81.7	22.2
Calhoun	TX	422	2,254	11.2	49.0	18.7	0.38	0.0	81.7	22.2
Hillsborough	FL	47,078	162,875	11.2	29.3	28.9	0.99	1.3	83.0	22.6
New York	NY	58,483	185,647	11.0	23.8	31.5	1.33	1.5	84.4	22.6
Middlesex	VA	27	1,161	10.9	2.4	2.3	0.96	0.0	84.4	22.6
Jackson	MS	1,262	15,150	10.6	7.0	8.3	1.19	0.1	84.5	22.6
Pacific	WA	387	2,428	10.4	9.4	15.9	1.70	0.0	84.6	22.6
King William	VA	53	1,822	10.2	2.7	2.9	1.09	0.0	84.6	22.6
Pasco	FL	6,351	55,279	9.8	16.6	11.5	0.69	0.4	85.0	22.4
Kauai	HI	818	7,140	9.7	10.1	11.5	1.14	0.1	85.1	22.4
Westmoreland	VA	58	1,793	9.7	5.7	3.2	0.57	0.0	85.1	22.4
Kent	MD	43	1,825	9.5	5.5	2.3	0.42	0.0	85.1	22.4
Brazoria	TX	15,596	34,807	9.4	31.0	44.8	1.45	0.3	85.4	22.5
Okaloosa	FL	1,431	19,409	9.2	10.7	7.4	0.69	0.2	85.5	22.4
St. Lucie	FL	2,766	28,822	8.8	20.1	9.6	0.48	0.2	85.7	22.4
Monmouth	NJ	7,911	56,321	8.8	12.5	14.0	1.12	0.4	86.2	22.2
Putnam	FL	394	5,984	8.2	10.7	6.6	0.62	0.0	86.2	22.2
Wakulla	FL	46	2,732	8.1	4.7	1.7	0.36	0.0	86.3	22.2
Jasper	SC	255	2,278	7.9	17.6	11.2	0.63	0.0	86.3	22.2
New Hanover	NC	760	17,616	7.8	7.7	4.3	0.56	0.1	86.4	22.1
Richmond	NY	6,251	38,315	7.7	19.6	16.3	0.83	0.3	86.7	22.0
Santa Rosa	FL	761	14,457	7.7	6.3	5.3	0.83	0.1	86.8	22.0
Berkeley	SC	933	17,511	7.6	8.8	5.3	0.60	0.1	87.0	21.9
Queens	NY	35,576	182,564	7.6	27.8	19.5	0.70	1.4	88.4	22.2
Matagorda	TX	718	2,690	7.4	42.6	26.7	0.63	0.0	88.4	22.3
Taylor	FL	98	1,570	7.2	4.0	6.2	1.56	0.0	88.4	22.3
Middlesex	MA	12,073	117,261	7.2	8.8	10.3	1.17	0.9	89.3	21.7
Hendry	FL	1,104	2,827	7.1	55.8	39.1	0.70	0.0	89.4	21.7
Barnstable	MA	604	16,268	7.1	3.5	3.7	1.08	0.1	89.5	21.6
Baldwin	AL	611	16,358	7.1	5.5	3.7	0.68	0.1	89.6	21.5
Pender	NC	209	4,140	6.9	8.3	5.0	0.61	0.0	89.7	21.5
King and Queen	VA	14	444	6.7	2.8	3.1	1.14	0.0	89.7	21.5
Dixie	FL	27	1,105	6.6	4.1	2.4	0.59	0.0	89.7	21.5
St. Martin	LA	108	3,402	6.6	3.2	3.2	0.98	0.0	89.7	21.5
Brunswick	NC	256	8,792	6.4	5.4	2.9	0.54	0.1	89.8	21.4
Clay	FL	1,165	13,645	6.3	10.6	8.5	0.81	0.1	89.9	21.4
Suffolk	NY	13,268	93,648	6.1	21.8	14.2	0.65	0.7	90.6	21.4
Levy	FL	86	2,511	5.9	9.5	3.4	0.36	0.0	90.6	21.4
Essex	VA	13	602	5.7	3.5	2.2	0.63	0.0	90.6	21.4
Maui	HI	1,161	9,286	5.6	10.3	12.5	1.22	0.1	90.7	21.3
Livingston	LA	219	7,700	5.4	6.2	2.8	0.46	0.1	90.8	21.3

Notes

1. In this table, “county” includes parishes in Louisiana, City of Baltimore, Maryland, and independent cities in Virginia.

2. Signifies the fraction of all residents identifying as non-Hispanic Black.

3. Percent of County residents who live below 1m.

4. Ratio of the percentage Black <1m and the percentage Black of county population.

5. The percentage of nationwide residents living below one meter in this county alone.

6. The percentage of nationwide residents living below one meter who live either in this county or counties listed above this county in this table.

7. The number of Hispanic residents as a percentage of all residents for this county and counties listed above this county in the table. For example, 6 million people live in the first 15 counties in this table, of whom 44% are Hispanic.

Table S6-D: Cause of Nationwide Disproportionality of Hispanic Residents below Three Meters— Population in Entire Counties and Below One Meter by County, 2020, ranked by Fraction of County Population below Three Meters

		Population <1m			%Hispanic[2]		Ratio[4] of	% Nationwide Population below 1m			Percent	Notes
County and States[1]		Hispanic	All races	As % of total[3]	County	<1m	Dispropor-tionality		his County[5]	Cumulative[6]	Hispanic of Cumulative[7]	
Alameda	CA	16,223	90,675	5.4	23.4	17.9	0.76		0.7	91.5	21.4	
Escambia	FL	1,406	17,149	5.3	6.5	8.2	1.27		0.1	91.6	21.3	
Bergen	NJ	17,779	50,440	5.3	21.4	35.2	1.65		0.4	92.0	21.3	
Fairfield	CT	12,485	49,505	5.2	21.4	25.2	1.18		0.4	92.4	21.3	
Harrison	MS	890	10,690	5.1	6.7	8.3	1.24		0.1	92.5	21.2	
Glades	FL	79	618	5.1	25.2	12.8	0.51		0.0	92.5	21.2	
St. Mary's	MD	213	5,768	5.1	5.8	3.7	0.64		0.0	92.5	21.2	
Cumberland	NJ	1,106	7,759	5.0	34.4	14.3	0.41		0.1	92.6	21.2	
Nueces	TX	3,312	17,467	4.9	61.5	19.0	0.31		0.1	92.7	21.5	
New Kent	VA	59	1,128	4.9	3.2	5.2	1.64		0.0	92.7	21.5	
Colleton	SC	21	1,869	4.8	3.6	1.2	0.32		0.0	92.7	21.5	
Camden	NJ	10,259	25,141	4.8	18.2	40.8	2.24		0.2	92.9	21.4	
Newport News	VA	548	8,630	4.6	10.4	6.4	0.61		0.1	93.0	21.4	
Newport	RI	328	3,731	4.4	6.5	8.8	1.34		0.0	93.0	21.4	
Plymouth	MA	523	22,676	4.3	4.5	2.3	0.51		0.2	93.2	21.2	
Essex	MA	6,683	33,983	4.2	22.6	19.7	0.87		0.3	93.5	21.2	
Anne Arundel	MD	1,111	24,164	4.1	9.7	4.6	0.48		0.2	93.7	21.1	
Dukes	MA	27	834	4.0	2.6	3.2	1.24		0.0	93.7	21.1	
Mobile	AL	493	16,229	3.9	3.2	3.0	0.94		0.1	93.8	20.9	
Horry	SC	705	13,731	3.9	6.9	5.1	0.74		0.1	93.9	20.8	
Essex	NJ	14,507	33,617	3.9	24.4	43.2	1.77		0.3	94.2	20.9	
Nantucket	MA	47	553	3.9	16.2	8.6	0.53		0.0	94.2	20.9	
Gloucester	NJ	1,046	11,663	3.9	7.3	9.0	1.23		0.1	94.3	20.8	
Middlesex	CT	426	6,179	3.8	7.3	6.9	0.95		0.0	94.3	20.8	
San Patricio	TX	884	2,583	3.8	55.6	34.2	0.62		0.0	94.3	20.8	
Norfolk	MA	1,258	27,162	3.7	5.2	4.6	0.88		0.2	94.5	20.6	
Burlington	NJ	2,044	17,133	3.7	8.7	11.9	1.36		0.1	94.7	20.5	
Washington	RI	126	4,807	3.7	3.5	2.6	0.74		0.0	94.7	20.5	
Lincoln	OR	150	1,734	3.4	9.6	8.6	0.90		0.0	94.7	20.4	
Calvert	MD	133	3,183	3.4	4.5	4.2	0.92		0.0	94.8	20.4	
Caroline	MD	48	1,131	3.4	8.5	4.3	0.51		0.0	94.8	20.4	
Orange	CA	14,123	107,666	3.4	34.1	13.1	0.38		0.8	95.6	21.2	
Island	WA	132	2,906	3.3	8.2	4.5	0.55		0.0	95.6	21.2	
Onslow	NC	740	6,627	3.2	13.5	11.2	0.83		0.1	95.7	21.1	
New Haven	CT	4,769	27,518	3.2	19.7	17.3	0.88		0.2	95.9	21.1	
Acadia	LA	20	1,778	3.1	2.9	1.1	0.39		0.0	95.9	21.1	
Gates	NC	10	321	3.1	1.9	3.2	1.68		0.0	95.9	21.1	
Coos	OR	174	1,951	3.0	6.6	8.9	1.35		0.0	95.9	21.1	
Wicomico	MD	104	3,049	2.9	6.8	3.4	0.50		0.0	96.0	21.0	
Solano	CA	4,224	12,915	2.8	28.3	32.7	1.16		0.1	96.1	21.1	
New London	CT	522	7,519	2.8	11.5	6.9	0.60		0.1	96.1	21.1	
Rockingham	NH	241	8,780	2.8	3.3	2.7	0.84		0.1	96.2	21.0	
Skagit	WA	437	3,608	2.8	18.4	12.1	0.66		0.0	96.2	21.0	
Bristol	MA	2,847	14,695	2.5	9.5	19.4	2.05		0.1	96.3	20.8	
San Francisco	CA	2,928	21,810	2.5	15.6	13.4	0.86		0.2	96.5	20.8	
Richmond	VA	6	221	2.5	6.7	2.7	0.40		0.0	96.5	20.8	
Sacramento	CA	8,450	38,250	2.4	23.6	22.1	0.94		0.3	96.8	20.8	
Humboldt	CA	493	3,178	2.3	13.6	15.5	1.14		0.0	96.8	20.8	

Notes

1. In this table, "county" includes parishes in Louisiana, City of Baltimore, Maryland, and independent cities in Virginia.

2. Signifies the fraction of all residents identifying as non-Hispanic Black.

3. Percent of County residents who live below 1m.

4. Ratio of the percentage Black <1m and the percentage Black of county population.

5. The percentage of nationwide residents living below one meter in this county alone.

6. The percentage of nationwide residents living below one meter who live either in this county or counties listed above this county in this table.

7. The number of Hispanic residents as a percentage of all residents for this county and counties listed above this county in the table. For example, 6 million people live in the first 15 counties in this table, of whom 44% are Hispanic.

Table S6-D: Cause of Nationwide Disproportionality of Hispanic Residents below Three Meters— Population in Entire Counties and Below One Meter by County, 2020, ranked by Fraction of County Population below Three Meters

County and States[1]		Population <1m Hispanic	All races	As % of total[3]	%Hispanic[2] County	<1m	Ratio[4] of Dispropor-tionality	% Nationwide Population below 1m his County[5]	Cumulative[6]	Percent Hispanic of Cumulative[7]
York	ME	147	4,893	2.3	1.9	3.0	1.55	0.0	96.9	20.8
Union	NJ	7,719	13,275	2.3	34.0	58.1	1.71	0.1	97.0	20.9
James City	VA	48	1,796	2.3	6.6	2.6	0.40	0.0	97.0	20.9
Chambers	TX	318	1,038	2.2	23.5	30.6	1.30	0.0	97.0	20.9
Baltimore	MD	1,101	18,966	2.2	7.2	5.8	0.81	0.1	97.1	20.7
Contra Costa	CA	7,300	25,513	2.2	27.0	28.6	1.06	0.2	97.3	20.8
Cecil	MD	81	2,252	2.2	5.3	3.6	0.68	0.0	97.4	20.8
Hernando	FL	221	4,172	2.1	14.9	5.3	0.35	0.0	97.4	20.8
Cameron	TX	5,629	9,022	2.1	89.5	62.4	0.70	0.1	97.5	21.2
Santa Clara	CA	7,004	41,375	2.1	25.2	16.9	0.67	0.3	97.8	21.3
Middlesex	NJ	5,248	18,406	2.1	22.4	28.5	1.27	0.1	97.9	21.3
Bronx	NY	15,180	31,327	2.1	54.8	48.5	0.88	0.2	98.2	22.0
Tillamook	OR	84	569	2.1	10.8	14.7	1.37	0.0	98.2	22.0
Washington	ME	21	619	2.0	2.1	3.4	1.60	0.0	98.2	22.0
West Baton Rouge	LA	17	533	2.0	4.6	3.2	0.71	0.0	98.2	22.0
Kent	DE	374	3,496	1.9	7.7	10.7	1.39	0.0	98.2	22.0
Kent	RI	244	3,143	1.8	5.7	7.7	1.37	0.0	98.2	21.9
Knox	ME	13	724	1.8	1.7	1.8	1.12	0.0	98.2	21.9
Cumberland	ME	133	5,309	1.8	2.6	2.5	0.95	0.0	98.3	21.8
Sagadahoc	ME	16	638	1.7	1.8	2.5	1.44	0.0	98.3	21.8
Jefferson	WA	21	573	1.7	4.0	3.6	0.91	0.0	98.3	21.8
New Castle	DE	759	9,861	1.7	11.1	7.7	0.69	0.1	98.4	21.7
Clallam	WA	107	1,330	1.7	6.1	8.1	1.32	0.0	98.4	21.7
Dorchester	SC	177	2,657	1.6	6.7	6.7	0.99	0.0	98.4	21.7
San Juan	WA	17	286	1.6	7.3	6.0	0.82	0.0	98.4	21.7
Ventura	CA	4,356	13,161	1.6	43.3	33.1	0.76	0.1	98.5	21.9
Lincoln	ME	10	549	1.6	1.1	1.9	1.67	0.0	98.5	21.9
Tangipahoa	LA	87	2,067	1.6	5.4	4.2	0.77	0.0	98.5	21.9
Charles City	VA	1	100	1.5	1.5	1.1	0.77	0.0	98.5	21.9
Willacy	TX	72	294	1.5	87.3	24.5	0.28	0.0	98.5	21.9
Bertie	NC	4	254	1.4	1.8	1.8	0.96	0.0	98.5	21.9
Alexandria	VA	769	2,242	1.4	18.4	34.3	1.86	0.0	98.5	21.9
Baltimore	MD	497	8,088	1.4	7.8	6.1	0.78	0.1	98.6	21.8
Isle of Wight	VA	31	526	1.4	3.1	6.0	1.92	0.0	98.6	21.8
Philadelphia	PA	1,923	21,833	1.4	14.9	8.8	0.59	0.2	98.8	21.6
Suffolk	VA	67	1,284	1.4	4.5	5.2	1.16	0.0	98.8	21.6
Kitsap	WA	456	3,538	1.3	8.8	12.9	1.47	0.0	98.8	21.5
Hancock	ME	18	693	1.2	1.7	2.6	1.47	0.0	98.8	21.5
Douglas	OR	90	1,308	1.2	6.0	6.9	1.15	0.0	98.8	21.5
Surry	VA	3	77	1.2	2.3	3.8	1.68	0.0	98.8	21.5
Seminole	FL	838	5,308	1.1	22.6	15.8	0.70	0.0	98.9	21.5
Whatcom	WA	141	2,555	1.1	10.1	5.5	0.55	0.0	98.9	21.5
Santa Cruz	CA	1,545	2,925	1.1	34.8	52.8	1.52	0.0	98.9	21.5
Okeechobee	FL	39	425	1.1	24.9	9.3	0.37	0.0	98.9	21.5
Charles	MD	61	1,764	1.1	7.0	3.5	0.49	0.0	98.9	21.5
DeSoto	FL	14	334	1.0	29.5	4.2	0.14	0.0	98.9	21.5
Mason	WA	47	644	1.0	11.6	7.4	0.64	0.0	99.0	21.5

Notes

1. In this table, "county" includes parishes in Louisiana, City of Baltimore, Maryland, and independent cities in Virginia.

2. Signifies the fraction of all residents identifying as non-Hispanic Black.

3. Percent of County residents who live below 1m.

4. Ratio of the percentage Black <1m and the percentage Black of county population.

5. The percentage of nationwide residents living below one meter in this county alone.

6. The percentage of nationwide residents living below one meter who live either in this county or counties listed above this county in this table.

7. The number of Hispanic residents as a percentage of all residents for this county and counties listed above this county in the table. For example, 6 million people live in the first 15 counties in this table, of whom 44% are Hispanic.

Table S6-D: Cause of Nationwide Disproportionality of Hispanic Residents below Three Meters— Population in Entire Counties and Below One Meter by County, 2020, ranked by Fraction of County Population below Three Meters

County and States[1]		Population <1m Hispanic	All races	As % of total[3]	%Hispanic[2] County	<1m	Ratio[4] of Dispropor-tionality	% Nationwide Population below 1m his County[5]	Cumulative[6]	Percent Hispanic of Cumulative[7]
Columbia	OR	32	515	1.0	5.8	6.2	1.06	0.0	99.0	21.5
Jackson	TX	18	147	1.0	32.2	12.1	0.37	0.0	99.0	21.5
Westchester	NY	2,265	9,606	1.0	26.8	23.6	0.88	0.1	99.0	21.6
Greene	NY	33	403	0.8	6.5	8.1	1.25	0.0	99.0	21.5
Martin	NC	5	185	0.8	4.1	2.9	0.70	0.0	99.0	21.5
San Diego	CA	5,887	26,294	0.8	33.9	22.4	0.66	0.2	99.2	22.0
Delaware	PA	411	4,524	0.8	4.6	9.1	1.96	0.0	99.3	21.9
Hawaii	HI	124	1,309	0.7	11.1	9.5	0.85	0.0	99.3	21.9
District of Columbia	DC	303	3,802	0.6	11.3	8.0	0.71	0.0	99.3	21.8
Hertford	NC	0	116	0.5	7.3	0.4	0.06	0.0	99.3	21.8
King George	VA	8	140	0.5	5.9	6.0	1.02	0.0	99.3	21.8
Santa Barbara	CA	855	2,278	0.5	47.0	37.5	0.80	0.0	99.3	21.9
Rockland	NY	348	1,694	0.5	19.6	20.5	1.05	0.0	99.4	21.9
Kleberg	TX	43	148	0.5	70.6	29.1	0.41	0.0	99.4	21.9
Providence	RI	380	3,001	0.5	24.3	12.7	0.52	0.0	99.4	22.0
Harford	MD	65	1,161	0.4	5.4	5.6	1.04	0.0	99.4	21.9
Del Norte	CA	18	115	0.4	19.2	15.9	0.83	0.0	99.4	21.9
Strafford	NH	12	507	0.4	3.0	2.3	0.77	0.0	99.4	21.9
Ulster	NY	94	695	0.4	11.6	13.5	1.16	0.0	99.4	21.8
Los Angeles	CA	6,291	35,494	0.4	48.0	17.7	0.37	0.3	99.7	24.6
Harris	TX	3,762	16,558	0.3	43.0	22.7	0.53	0.1	99.8	25.4
Thurston	WA	72	1,017	0.3	9.8	7.1	0.72	0.0	99.8	25.4
Fairfax	VA	465	3,899	0.3	17.3	11.9	0.69	0.0	99.8	25.3
Napa	CA	105	460	0.3	35.4	22.9	0.65	0.0	99.8	25.3
Waldo	ME	4	127	0.3	1.4	3.2	2.26	0.0	99.8	25.3
Lake	FL	118	1,195	0.3	17.0	9.9	0.58	0.0	99.9	25.3
Stafford	VA	54	471	0.3	15.1	11.5	0.76	0.0	99.9	25.3
Snohomish	WA	287	2,414	0.3	11.6	11.9	1.03	0.0	99.9	25.2
Monterey	CA	1,002	1,272	0.3	60.4	78.8	1.30	0.0	99.9	25.3
Sonoma	CA	434	1,391	0.3	28.9	31.2	1.08	0.0	99.9	25.3
Yolo	CA	313	607	0.3	33.1	51.6	1.56	0.0	99.9	25.3
Pitt	NC	73	443	0.3	7.6	16.5	2.16	0.0	99.9	25.3
Bucks	PA	169	1,624	0.3	6.2	10.4	1.69	0.0	99.9	25.2
Pierce	WA	289	2,209	0.2	12.1	13.1	1.08	0.0	99.9	25.1
Columbia	NY	24	146	0.2	5.8	16.3	2.84	0.0	99.9	25.1
San Luis Obispo	CA	64	373	0.1	24.0	17.1	0.71	0.0	99.9	25.1
King	WA	280	2,775	0.1	10.7	10.1	0.94	0.0	100.0	24.7
Lafayette	LA	13	292	0.1	6.6	4.6	0.69	0.0	100.0	24.7
Putnam	NY	9	112	0.1	18.2	7.7	0.42	0.0	100.0	24.7
Liberty	TX	30	102	0.1	33.6	29.5	0.88	0.0	100.0	24.7
Orange	NY	107	435	0.1	22.4	24.6	1.10	0.0	100.0	24.7
Penobscot	ME	8	147	0.1	1.8	5.6	3.12	0.0	100.0	24.7
Dutchess	NY	52	280	0.1	14.3	18.7	1.31	0.0	100.0	24.6
Prince William	VA	51	435	0.1	25.2	11.7	0.46	0.0	100.0	24.6
Multnomah	OR	104	715	0.1	12.7	14.6	1.15	0.0	100.0	24.5
Passaic	NJ	257	402	0.1	42.7	63.8	1.49	0.0	100.0	24.6
Rensselaer	NY	9	107	0.1	5.9	8.7	1.48	0.0	100.0	24.6
Arlington	VA	16	138	0.1	15.7	11.3	0.72	0.0	100.0	24.5
Prince George's	MD	208	482	0.0	21.2	43.2	2.03	0.0	100.0	24.5

Notes

1. In this table, “county” includes parishes in Louisiana, City of Baltimore, Maryland, and independent cities in Virginia.
2. Signifies the fraction of all residents identifying as non-Hispanic Black.
3. Percent of County residents who live below 1m.
4. Ratio of the percentage Black <1m and the percentage Black of county population.
5. The percentage of nationwide residents living below one meter in this county alone.
6. The percentage of nationwide residents living below one meter who live either in this county or counties listed above this county in this table.
7. The number of Hispanic residents as a percentage of all residents for this county and counties listed above this county in the table. For example, 6 million people live in the first 15 counties in this table, of whom 44% are Hispanic.

Table S6-D: Cause of Nationwide Disproportionality of Hispanic Residents below Three Meters— Population in Entire Counties and Below One Meter by County, 2020, ranked by Fraction of County Population below Three Meters

		Population <1m			%Hispanic[2]		Ratio[4] of	% Nationwide Population below 1m			Percent	Notes
County and States[1]		Hispanic	All races	As % of total[3]	County	<1m	Dispropor-tionality	his County[5]		Cumulative[6]	Hispanic of Cumulative[7]	
Lane	OR	12	179	0.0	9.9	6.5	0.66		0.0	100.0	24.5	
East Baton Rouge	LA	10	199	0.0	6.7	5.3	0.79		0.0	100.0	24.4	

The purpose of this table is to illustrate the high proportion of Hispanic residents in localities most vulnerable to sea level rise.

Table S7: Ratios of How Disproportional the Population of Blacks and Hispanics in Vulnerable Zones Is Compared to the Total Population of Blacks and Hispanics.

	Hazard Zone	Nationwide Ratio of Disproportionality[1]					County-Adjusted Ratio of Disproportionality[2]					Percent Change in Population 1990–2020	
		1990	2000	2010	2020		1990	2000	2010	2020		Actual	Counter Factual[3]
**Black**													
Actual	<1m	1.92	1.97	1.70	1.63		0.92	0.92	0.89	0.89		2	6
Δ Census[4]	<1m	1.83	1.90	1.68	1.63		0.91	0.91	0.88	0.89		-6	-3
Δ *Sea Level* [5]	<1m	1.70	1.68	1.65	1.63		0.90	0.89	0.89	0.89		10	12
Actual	<3m	1.43	1.48	1.45	1.38		0.92	0.94	0.93	0.94		28	26
Δ Census[4]	<3m	1.44	1.49	1.46	1.38		0.93	0.94	0.94	0.94		23	22
Δ *Sea Level*	<3m	1.37	1.37	1.38	1.38		0.93	0.94	0.94	0.94		4	4
Except New Orleans[6]	<1m	1.00	1.08	1.14	1.12		0.77	0.78	0.78	0.78		48	45
	<3m	1.24	1.31	1.36	1.29		0.91	0.93	0.93	0.93		41	38
A Zone	All	1.00	1.00	0.99	0.95		0.91	0.91	0.90	0.88		18	22
A Zone	Coastal	1.03	1.05	1.01	0.95		0.81	0.82	0.81	0.80		19	21
A Zone	Inland	0.96	0.96	0.98	0.96		1.01	1.01	1.00	0.98		19	23
X500 Zone	All	1.03	1.00	0.98	0.95		1.09	1.08	1.07	1.03		37	44
**Hispanic**													
Actual	<1m	1.05	0.92	0.94	1.01		0.95	0.91	0.89	0.89		148	167
Δ Census[4]	<1m	1.07	0.93	0.95	1.01		0.95	0.91	0.89	0.89		115	132
Δ *Sea Level* [5]	<1m	1.00	1.00	1.01	1.01		0.89	0.89	0.89	0.89		16	17
Actual	<3m	1.63	1.48	1.38	1.38		0.97	0.96	0.95	0.95		140	146
Δ Census[4]	<3m	1.61	1.47	1.37	1.38		0.97	0.96	0.95	0.95		135	140
Δ *Sea Level* [5]	<3m	1.39	1.39	1.38	1.38		0.95	0.95	0.95	0.95		2	2
A Zone	All	1.24	1.21	1.14	1.08		1.01	1.02	1.00	0.94		132	151
A Zone	Coastal	1.29	1.17	1.10	1.09		0.91	0.89	0.87	0.85		134	152
A Zone	Inland	1.19	1.24	1.16	1.07		1.13	1.14	1.14	1.03		130	151
X500 Zone	All	1.99	1.97	1.75	1.50		1.09	1.09	1.08	0.96		137	167

1. $D = \frac{F^*_{Black}/F^*}{X^*_{Black}/X^*}$, where F^*_{Black} and X^*_{Black} are the nationwide Black population in the hazard zone and total population, respectively, and F^* and X^* are the total population of all races in the hazard zone and nationwide, respectively. This might also be written as $D = \frac{\sum_i F_{Black,i}}{F^* X^*_{Black}/X^*}$ to emphasize that D is the ratio of the vulnerable Black population to what the vulnerable Black population would be if racial composition in hazard zones was the same as the nationwide racial composition.
2. $C = \frac{\sum_i F_{Black,i}}{\sum_i F_i X_{Black,i}/X_i}$, where X_i is the population in county *i*, $X_{Black,i}$ is the Black population in county *i*, F_i is the population in the hazard zone of county i, and $F_{Black,i}$ is the Black population in hazard zones in county *i*. That is, C is computed as the ratio of the total vulnerable Black population to a sum that represents what the vulnerable Black population would be if the fraction of the population in the hazard zone that is Black equaled the fraction of the county population that is Black. Defining $D_i = (F_{Black,i}/F_i)/(X_{Black,i}/X_i)$, then $F_{Black,i}/D_i = F_i X_{Black,i}/X_i$, so:
$$C = \frac{\sum_i F_{Black,i}}{\sum_i F_i/D_i}, \text{ and } \frac{1}{C} = \frac{\sum_i F_i/D_i}{\sum_i F_{Black,i}} = \frac{1}{F^*_{Black}} \sum_i \frac{F_i}{D_i},$$
which means that 1/C is a weighted sum of the $1/D_i$. Thus the county-adjusted ratio of disproportionality is an inverse weighted average of the ratios of disproportionality of each county.
3. This is the percentage change of the alternative data series $\sum_i F_i X_{Black,i}/X_i$, that is, the denominator used to calculate C. This can be interpreted as what the percentage change of the Black population would have been had the racial composition in the hazard zone been the same as the nationwide population at large.
4. *Δ Census* represents population for the Census from the column year within 1m or 3m of sea level for the year 2020. Changes on this line exclude the effects of sea level rise.
5. *Δ Sea Level* shows population based on the 2020 Census within 1m or 3m above sea level for the column year. Changes on this line reflect the effects of sea level rise.
6. Same as "actual" except results exclude New Orleans.

See File of Landscape Tables for S8-A to S9-D

Table S9-E: Summary Statistics Measuring whether Blacks Account for a Disproportionate Share of People Inhabiting Hazard Zones Who Emigrate from Hazard Zones in Counties with Net Emigration

	Low-Lying Lands				Floodplain: X500 and A Zones				
	Nationwide		Exclude New Orleans			Nationwide			
	<1m	<3m	<1m	<3m	X500	X500	Coastal A	Riverine A	A and X500
Population Migrating Out of Low Land (1990-2020)									
All Races[1]	-169,312	-225,584	-68,333	-115,569	-173,306	-235,752	-77,991	-243,390	-557,132
Black[2]	-98,354	-118,795	-15,196	-23,872	-32,904	-80,043	-26,296	-47,047	-153,386
Black (∝vulnerable)[3]	-76,689	-87,289	-11,264	-19,777	-37,009	-79,370	-19,925	-48,128	-147,424
Ratios									
County-Adjusted Disproportionality[4]	1.283	1.361	1.349	1.207	0.889	1.008	1.320	0.978	1.040
Nationwide Disproportionality[5]	2.575	3.134	1.918	1.427	1.755	2.815	2.774	1.709	2.687
Black share[6] population	0.226	0.168	0.116	0.145	0.108	0.121	0.122	0.113	0.119
Black share of Migration[7]	0.581	0.527	0.222	0.207	0.190	0.340	0.337	0.193	0.320

1. Total apparent emigration from hazard zone in counties with net emigration from hazard zone. See final lines of Tables S8-A, S8-B, S8-G, S8-F, and S8-E (for all but “Exclude New Orleans” and “A and X500”).
2. Apparent emigration of Black residents (net of displacements by nonblack residents) from hazard zone in counties with net emigration from hazard zone. See “Total, Excluding Displacement” in Tables S8-C, S8-D, S8-G, S8-F, and S8-E (for all but “Exclude New Orleans” and “A and X500”).
3. The sum across counties of the product of the Black share of the county population times “All Races”, above; that is, the number of Blacks that would have emigrated from the vulnerable zone if Black emigration was proportional to the emigration from all races. This is analogous to the denominator in note 2 of Table S7, except that “X” refers to those inhabiting the hazard zone, while “F” refers to those who emigrate.
4. County-Adjusted Ratio of Disproportionality, see Table S7 of note 2 for the formula, except that in this case, “X” refers to those inhabiting the hazard zone, while “F” refers to apparent emigration.
5. Ratio of Black share of Migration to Black share of residents in the hazard zone.
6. Using population at the beginning of the period, refers to share in hazard zone.
7. Ratio of “Black” to “All Races”.

Table S9-F: Summary Statistics Measuring whether Blacks Account for a Disproportionate Share of Emigration from Vulnerable Areas in Counties with Net Emigration									
	Low-Lying Lands				Floodplain: X500 and A Zones				
	Nationwide		Exclude New Orleans			Nationwide			
	<1m	<3m	<1m	<3m	X500	X500	Coastal A	Riverine A	A and X500
Population Migrating Out of Low Land (1990-2020)									
All Races[1]	-169,312	-225,584	-68,333	-115,569	-173,306	-235,752	-77,991	-243,390	-557,132
Black[2]	-98,354	-118,795	-15,196	-23,872	-32,904	-80,043	-26,296	-47,047	-153,386
Black (∝population)[3]	-72,599	-87,309	-10,613	-19,777	-32,683	-71,015	-21,166	-40,121	-132,302
Ratios									
County-Adjusted Disproportionality[4]	1.355	1.361	1.432	1.207	1.007	1.127	1.242	1.173	1.159
Nationwide Disproportionality[5]	4.945	4.483	1.909	1.773	1.630	2.890	2.870	1.645	2.725
Black share[6] population	0.117	0.117	0.116	0.116	0.116	0.117	0.117	0.117	0.117
Black share of Migration[7]	0.581	0.527	0.222	0.207	0.190	0.340	0.337	0.193	0.320

1. Total apparent emigration from hazard zone in counties with net emigration from hazard zone. See final lines of Tables S8-A, S8-B, S8-G, S8-F, and S8-E (for all but “Exclude New Orleans” and “A and X500”).
2. Apparent emigration of Black residents (net of displacements by nonblack residents) from hazard zone in counties with net emigration from hazard zone. See “Total, Excluding Displacement” in Tables S8-C, S8-D, S8-G, S8-F, and S8-E (for all but “Exclude New Orleans” and “A and X500”).
3. The sum across counties of the product of the Black share of the county population times “All Races”, above; that is, the number of Blacks that would have emigrated from the vulnerable zone if Black emigration was proportional to the emigration from all races. This is analogous to the denominator in note 2 of Table S7, except that “F” refers to those who emigrate.
4. County-Adjusted Ratio of Disproportionality, see Table S7 of note 2 for the formula, except that in this case, “F” refers to apparent emigration.
5. Ratio of Black share of migration to Black share of population.
6. Using population at the beginning of the period, refers to share of all population.
7. Ratio of “Black” to “All Races”.

Table S10: Summary Statistics Measuring the Extent to which Blacks Account for a Disproportionate Share of Residents in, and Emigration from, Vulnerable Areas, 1990 to 2020

		Low-Lying Lands				Floodplain: X500 and A Zones				
		Nationwide		Exclude New Orleans			Nationwide			
		<1m	<3m	<1m	<3m	X500	X500	Coastal A	Riverine A	A and X500
Population Migrating Out of Low Land[1]										
All Races		169,312	225,584	68,333	115,569	173,306	235,752	77,991	243,390	557,132
Black		98,354	118,795	15,196	23,872	32,904	80,043	26,296	47,047	153,386
If Black $\propto$ Vulnerable[2]		76,689	87,289	11,264	19,777	37,009	79,370	19,925	48,128	147,424
If Black $\propto$ Population[3]		72,599	87,309	10,613	19,776	32,683	71,015	21,166	40,121	132,302
Black share of….										
Vulnerable Residents		0.226	0.168	0.116	0.145	0.108	0.121	0.122	0.113	0.119
Emigration[1]		0.581	0.527	0.222	0.207	0.190	0.340	0.337	0.193	0.320
Total Population		0.117	0.116	0.117	0.116	0.116	0.117	0.117	0.117	0.117
Nationwide Ratios of Disproportionality										
Vulnerable Residents[4]	1990	1.92	1.43	1.00	1.24	0.93	1.03	1.03	1.01	1.01
	2020	1.63	1.38	1.12	1.29	0.91	0.95	0.95	0.95	0.95
Emigration/Vulnerable[5]		2.58	3.13	1.92	1.43	1.76	2.82	2.77	1.71	2.69
Emigration/Population[6]		4.94	4.48	1.91	1.77	1.63	2.89	2.87	1.65	2.73
County Weighted Ratio of Disproportionality[7]										
Vulnerable Residents	1990	0.92	0.92	0.77	0.91	1.09	1.09	0.81	0.98	0.98
	2020	0.89	0.94	0.78	0.93	1.02	1.03	0.80	0.91	0.91
Emigration/Vulnerable		1.28	1.36	1.35	1.21	0.89	1.01	1.32	0.98	1.04
Emigration/Population		1.35	1.36	1.43	1.21	1.01	1.13	1.24	1.17	1.16

1. See Table S9-F for details.
2. The sum across counties of the product of the Black share of the county population times "All Races", above; that is, the number of Blacks that would have emigrated from the vulnerable zone if the Black share of emigration in each county was the same as the Black share of residents of the vulnerable area. See Table S9-E, note 3 for details.
3. Similar to "Black $\propto$ Vulnerable" (note 2) except that it represents the number of Blacks that would have emigrated from the vulnerable zone if the share of Black emigration in each county was the same as the Black share of county residents. See Table S9-F, note 3 for details
4. Ratio of Black share of vulnerable residents to Black share of population
5. Ratio of Black share of emigration to Black share of vulnerable residents
6. Ratio of Black share of emigration to Black share of population.
7. County-Adjusted Ratio of Disproportionality, see Table S7 note 2 for the formula for "Vulnerable Residents. See note 4 from Table S9-E and S9-F for the other two lines. This measure filters out the effect of different racial compositions in different counties, and thus is a weighted combination of disproportionalities within counties.

Table S10(i): Summary Statistics Measuring the Extent to which Blacks Account for a Disproportionate Share of Residents Emigration from, Vulnerable Areas 1990 to 2020

	Low-Lying Lands					Floodplain: X500 and A Zones		
	Nationwide		Exclude New Orleans			Nationwide		
	<1m	<3m	<1m	<3m	X500	X500	Coastal A	Riverine A
**Population Migrating Out of Low Land[1]**								
All Races	169,312	225,584	68,333	115,569	173,306	235,752	77,991	243,390
Black	98,354	118,795	15,196	23,872	32,904	80,043	26,296	47,047
**Black share of….**								
Vulnerable Residents	0.226	0.168	0.116	0.145	0.108	0.121	0.122	0.113
Emigration[1]	0.581	0.527	0.222	0.207	0.190	0.340	0.337	0.193
**Nationwide Ratios of Disproportionality**								
Vulnerable Residents[2]	1.63	1.38	1.12	1.29	0.91	0.95	0.95	0.96
Emigration to Vulnerable[3]	2.58	3.13	1.92	1.43	1.76	2.82	2.77	1.71
Emigration to Population[4]	4.94	4.48	1.91	1.77	1.63	2.89	2.87	1.65
**County Weighted Ratio of Disproportionality[5]**								
Vulnerable Residents 2020	0.89	0.94	0.78	0.93	1.02	1.03	0.80	0.98
Emigration to Vulnerable	1.28	1.36	1.35	1.21	0.89	1.01	1.32	0.98
Emigration to Population	1.35	1.36	1.43	1.21	1.01	1.13	1.24	1.17

1. Total across Counties with Net Emigration from the vulnerable zone defined by a given column. (See Tables S-8A to Tables S-8D.)
2. Ratio of Black share of vulnerable residents to Black share of population, 2020
3. Ratio of Black share of emigration 1990-2020 to Black share of vulnerable residents in 1990.
4. Ratio of Black share of emigration 1990-2020 to Black share of population in 1990.
5. County-Adjusted Ratio of Disproportionality, see Table S7 note 2 for the formula for “Vulnerable Residents. See note 4 from Table S9-E and S9-F for the other two lines. This measure filters out the effect of different racial compositions in different counties, and thus is a weighted combination of disproportionalities within counties.

Table S10(ii): Blacks Share of Residents and Emigration from Vulnerable Areas, 1990 to 2020									
	Low-Lying Lands				Floodplain: X500 and A Zones				
	Nationwide		Exclude New Orleans			Nationwide			
	<1m	<3m	<1m	<3m	X500	X500	Coastal A	Riverine A	A and X500
Population Migrating Out of Low Land[1]									
All Races	169,312	225,584	68,333	115,569	173,306	235,752	77,991	243,390	557,132
Black	98,354	118,795	15,196	23,872	32,904	80,043	26,296	47,047	153,386
Black share of….									
Vulnerable Residents	0.226	0.168	0.116	0.145	0.108	0.121	0.122	0.113	0.119
Emigration	0.581	0.527	0.222	0.207	0.190	0.340	0.337	0.193	0.320
Nationwide Ratios of Disproportionality									
Vulnerable Residents[1]	1.63	1.38	1.12	1.29	0.91	0.95	0.95	0.96	0.95
Emigration[2]	4.94	4.48	1.91	1.77	1.63	2.89	2.87	1.65	2.73
County-Weighted Ratio of Disproportionality									
Vulnerable Residents[4]	0.89	0.94	0.78	0.93	1.02	1.03	0.80	0.98	0.91
Emigration	1.35	1.36	1.43	1.21	1.01	1.13	1.24	1.17	1.16

1. Ratio of Black share of residents vulnerable to sea level rise or flooding to Black share of population, 2020
2. Ratio of Black share of emigration to Black share of population.
3. This measure filters out the effect of different racial compositions in different counties. Same as County-By-County disproportionality in Figure 6.

See File of Landscape Tables for S11-A to S12-D

TABLE S13-A

Apparent Emigration 1990–2020 from 100-Year Floodplain in HOLC Red Zone by County[1]

County/Parish	State	Apparent Emigration from Floodplain in Red Zone[2]				Population in 2020, Red Zone		Emigration as % of population in floodplain[3]
		1990 to 2020	1990s	2000s	2010s	Flood-plain	Red Zone	
Orleans	Louisiana	-7,553		-12,029		9,509	92,706	-44
Jefferson[4]	Alabama	-4,318	-2,061	-1,094	-1,163	2,525	77,365	-63
Hinds	Mississippi	-2,010	-1,063	-415	-532	1,411	7,162	-59
Galveston	Texas	-1,503	-1,024	-2,505		5,262	6,303	-22
Montgomery	Alabama	-1,010	-301	-412	-298	604	15,362	-63
Ohio	West Virginia	-866	-361	-533		2,422	5,469	-26
Allegheny	Pennsylvania	-821		-874	-478	2,370	96,958	-26
Portsmouth	Virginia	-670	-271	-506		3,356	12,195	-17
Will	Illinois	-659		-85	-732	2,676	7,291	-20
Bexar	Texas	-654	-95	-347	-212	2,310	85,196	-22
Wayne	Michigan	-621	-326	-200	-95	1,640	218,467	-27
Philadelphia	Pennsylvania	-599	-399	-4	-196	4,961	544,835	-11
Pulaski	Arkansas	-560	-325	-32	-203	264	8,249	-68
Cambria	Pennsylvania	-418	-182	-85	-151	749	5,879	-36
Genesee	Michigan	-394	-365		-254	266	31,846	-60
Kings	New York	-349	-2,516	-708		47,060	998,327	-1
Ingham	Michigan	-335	-96	-10	-229	775	3,433	-30
Niagara	New York	-290	-223	-68		876	3,958	-25
Allen[4]	Indiana	-256	-110	-137	-9	520	2,726	-33
Hampton	Virginia	-239	-62	-36	-141	95	2,011	-72
Scott	Iowa	-238	-100	-188		820	6,645	-23
Los Angeles	California	-235	-339	-174		1,266	1,166,664	-16
Norfolk	Virginia	-227	-257		-399	1,702	37,535	-12
Polk[4]	Iowa	-213		-171	-74	318	37,336	-40
Scioto	Ohio	-205	-203		-72	356	3,404	-37
Duval	Florida	-198	-212	-35		414	36,904	-32
Winnebago	Illinois	-192	-151		-260	775	35,889	-20
Caddo	Louisiana	-175	-51	-16	-108	354	10,129	-33
Kanawha	West Virginia	-167	-76	-50	-41	276	3,634	-38
Sedgwick	Kansas	-159	-14	-143	-2	1,233	49,511	-11
Essex	New Jersey	-142		-231		1,146	163,424	-11
Franklin	Ohio	-135	-34	-54	-47	237	31,842	-36
Lawrence	Pennsylvania	-126	-43	-42	-41	338	1,584	-27
Chatham[4]	Georgia	-124		-276		7	6,268	-95
Total[5]		-28,319	-14,173	-26,991	-8,724	288,217	10,933,204	-10
Excluding New Orleans		-20,766	-14,173	-14,961	-8,724	278,708	10,840,498	-8

1. Ranked by net emigration 1990 to 2020 from the Home Owners Loan Corporation (HOLC) red zone.
2. Change in population of the red lined portion of the 100-year flood plains as mapped by the National Flood Hazard Layer. Includes migration into and out of this land as well as births and deaths. Negative numbers mean emigration out of this county's inland A zone.
3. Emigration from floodplain 1990–2020 divided by floodplain population of 1990.
4. One of 9 counties with at least 100 FEMA buyouts and at least one buyout in a red zone from the HOLC redlining maps. See Zavar and Fisher (2021) at Table 2.
5. Sum of emigration in counties with net emigration from flood-zone for a given time period.

TABLE S13-B
Apparent Emigration 1990–2020 from 100-Year Floodplain in HOLC Yellow Zone by County[1]

	State	Apparent Emigration from Floodplain in Yellow Zone[2]					Population in 2020, Yellow Zone		Emigration as % of 1990 population in floodplain[3]
County/Parish		1990 to 2020	1990s	2000s	2010s		Flood-plain	Entire Zone	
Orleans	LA	-3,055	-286	-4,766			11,475	77,232	-21
Atlantic	NJ	-1,360	-147	-912	-301		3,815	43,272	-26
Wayne	MI	-1,232	-615	-832			5,749	445,602	-18
Cambria	PA	-1,057	-688		-542		2,043	13,093	-34
Los Angeles	CA	-912		-577	-636		10,838	2,435,238	-8
Ohio	WV	-685	-350	-266	-68		1,789	7,390	-28
Will	IL	-623		-342	-535		3,129	35,159	-17
St. Louis	MO	-592	-107	-330	-155		920	58,804	-39
Onondaga	NY	-568	-344	-9	-215		1,649	70,688	-26
Galveston	TX	-555	-148	-313	-94		1,295	1,468	-30
Hinds	MS	-531	-348	-219			182	4,156	-74
Portsmouth	VA	-507	-681	-365			3,781	18,908	-12
Caddo	LA	-475	-104	-23	-349		498	13,397	-49
St. Louis	MO	-334	-23	-303	-7		293	67,536	-53
Summit	OH	-330	-31	-149	-150		696	113,934	-32
Ingham	MI	-316	-515				3,496	69,039	-8
Bexar	TX	-303	-281		-129		2,182	104,883	-12
Allegheny	PA	-299		-434			1,801	171,969	-14
Jefferson[4]	AL	-293	-47	-106	-140		576	50,894	-34
Lucas	OH	-290	-35	-144	-112		1,073	99,728	-21
Jefferson	LA	-264	-53	-95	-117		1,012	13,732	-21
Albany	NY	-246	-290				1,890	35,362	-12
Oneida	NY	-238	-90	-52	-96		699	41,857	-25
Norfolk	VA	-237			-3,344		8,616	60,322	-3
Wayne	WV	-220	-134	-32	-54		419	3,357	-34
Montgomery	AL	-176	-97	-50	-29		182	14,211	-49
Allen[4]	IN	-175	-62	-152			1,191	50,126	-13
Mobile[4]	AL	-166	-39	-105	-22		433	10,333	-28
Total[5]		-18,579	-7,858	-17,457	-10,188		305,465	18,881,010	-0.10
Excluding New Orleans		-15,524	-7,572	-12,691	-10,188		293,990	18,803,778	-0.08

1. Ranked by net emigration 1990 to 2020 from the Home Owners Loan Corporation yellow zone.
2. Change in population of the A zones as mapped by the National Flood Hazard Layer. Includes migration into and out of this land as well as births and deaths. Negative numbers mean emigration out of this county's inland A zone within the yellow HOLC zone.
3. Emigration from floodplain 1990–2020 divided by floodplain population of 1990.
4. One of 12 counties with at least 100 FEMA buyouts and at least one buyout in an HOLC-mapped yellow zone. See Zavar and Fisher (2021) at Table 2.
5. Sum of emigration in counties with net emigration from flood-zone for a given time period.

B

Table S13-C
Measurement Error and Statistical Significance for Emigration from Land Below One Meter

		USA				USA Exclude New Orleans			
Inhabitants Below One meter		1990	2010	2020[1]	2022[2]	1990	2010	2020[1]	2022[2]
Standard Error[3]	Black	1,535	1,246	1,110	1,123	1,010	1,246	759	1,123
	All	17,496	28,311	16,305	27,949	13,630	28,311	13,220	24,864
Standard Error, (per 100,000 total population)[4]	Black	5.3	3.3	2.8	3.0	3.5	3.3	1.9	3.0
	All	7.0	9.1	4.9	8.4	5.5	9.1	3.9	7.4
	Difference	7.2	8.5	4.7	7.9	5.4	8.5	3.8	7.0
**Emigration**		1990 to 2020		2010 to 2020		1990 to 2020		2010 to 2020	
Total Emigration	Black	-98,354		-6,590		-15,196		-6,590	
	All	-169,312		-29,611		-68,333		-29,611	
Standard Error (Persons)[5]	Black	1,567		1,262		1,263		1,678	
	All	19,549		29,864		15,513		28,376	
Emigration (per 100,000)	Black	-336.7		-17.5		-52.0		-17.5	
	All	-54.2		-9.5		-21.9		-9.5	
Significance (t-ratio)	Black	-62.8		-5.22		-12.03		-3.93	
	All	-8.66		-0.991		-4.40		-1.04	
Significance (% upper tail)	Black	<0.00001		0.00006		<0.00001		0.00075	
	All	<0.00001		0.169		0.00029		0.158	
**Disproportionality of Emigration** (per 100,000)									
Black excess Emigration[6]		-282.5		-8.0		-30.1		-8.0	
Standard Error		5.3		5.7		3.9		5.6	
Statistical Significance (t-ratio)		-53.2		-1.40		-7.80		-1.42	
Significance (% upper tail)		<0000001		0.0926		<0000001		0.0887	

1. For counties with net emigration from land below 1m 1990-2020
2. For counties with net emigration from land below 1m 2010-2020
3. Standard Error from measurement/model error of estimated population below one meter in counties that had net emigration from land below one meter from in a period of time starting or ending with year shown. See Supplemental Methods §8.3 for details
4. Same as above divided by US population multiplied by 100,000. Converting Black and all population to a share of total population facilitates evaluation of the statistical differences.
5. Standard error of migration for 2010-2020 calculated using standard error estimates of population measurement error, and estimated correlation of measurement error between 2010 and 2020, the same coefficient is cubed for the 30-year emigration estimate.
6. Black emigration proportion minus all emigration proportion
7. Standard error of Black excess emigration calculated following the standard formula for the variance of (a1-a2) – (b1-b2) using estimates of the covariances from the error of the ratio estimate in the sample data set used to develop ratio estimates. See Supplemental Methods §8.3.
8. Based on Students t-distribution for 14.2 degrees of freedom.

NOTE “Land Below One Meter” means land less than one meter above mean higher high water. A one meter sea level rise would not necessarily submerge most or all lands below one meter.

Table S14-A. Change in Population of Land Less than One Meter Above Sea Level[1] Along US Atlantic Coast, by State and Likelihood of Shore Protection											
	**Where Shore Protection is Unlikely[2]**						**Where Shore Protection is Likely[2]**				
State	**1990**	**2000**	**2010**	**2020**	**Change**		**1990**	**2000**	**2010**	**2020**	**Change**
**MA**	1,080	1,315	1,218	1,146	66		4,369	4,700	3,966	4,436	67
**RI**	391	422	584	340	-52		170	202	210	332	162
**CT**	104	118	77	104	0		304	174	171	225	-79
**NY**	567	896	873	983	415		1,104	1,394	886	1,128	24
**NJ**	2,163	2,178	2,228	2,700	537		5,292	5,708	5,109	5,879	587
**PA**	109	83	13	100	-9		118	157	178	142	25
**DE**	388	324	438	496	108		379	655	1,071	1,987	1,608
**DC**	0	0	0	0	0		0	0	0	0	0
**VA**	2,454	2,569	2,120	2,122	-331		3,947	4,664	3,977	3,931	-16
**MD**	4,702	4,908	4,415	4,265	-437		3,589	3,669	3,550	3,438	-151
**NC**	8,333	8,772	9,538	8,845	512		2,903	2,895	3,466	3,791	888
**SC**	3,170	3,680	3,199	3,641	470		6,366	5,954	7,383	9,207	2,841
**GA**	371	379	390	452	81		1,308	1,380	2,325	2,097	789
**FLE[2]**	806	1,637	1,867	2,446	1,640		4,486	4,769	5,492	6,998	2,512
**Atlantic**	24,638	27,279	26,961	27,639	3,001		34,335	36,322	37,783	43,591	9,256
	**Where Shore Protection is Almost Certain[2]**						**All Lands[4]**				
**MA**	64,653	70,325	74,740	86,990	22,337		71,065	77,256	81,061	93,873	16,617
**RI**	1,099	1,121	814	1,062	-38		1,916	1,995	1,782	1,866	-50
**CT**	11,327	11,382	11,375	12,343	1,016		12,533	12,457	12,588	13,517	984
**NY**	82,459	85,908	87,245	99,868	17,409		88,439	91,910	93,708	105,189	16,750
**NJ**	108,792	114,734	112,359	119,834	11,041		117,627	124,577	121,219	129,567	11,940
**PA**	2,439	1,402	1,457	1,324	-1,115		2,801	1,707	1,858	1,579	-1,222
**DE**	4,148	5,554	6,264	5,749	1,602		5,304	7,644	8,762	9,493	4,189
**DC**	235	103	332	527	292		235	103	332	527	292
**VA**	28,310	29,181	27,276	28,843	533		35,760	37,574	34,433	35,876	116
**MD**	14,933	16,309	16,293	15,803	870		25,328	26,640	26,179	25,491	162
**NC**	19,444	19,970	19,282	18,446	-999		32,330	32,955	33,566	32,101	-229
**SC**	28,462	32,603	35,432	38,551	10,089		38,189	42,380	46,625	51,954	13,765
**GA**	9,036	10,044	10,179	13,036	4,000		13,058	15,608	16,337	19,384	6,325
**FLE[3]**	402,175	428,135	447,608	489,768	87,593		467,866	504,788	525,988	585,192	117,327
**Atlantic**	777,513	826,772	850,657	932,142	154,629		912,451	977,594	1,004,439	1,105,609	193,158

1. Sea level of 2020.
2. Shore Protection Likelihood from Titus, J. G., et al. 2009. State and local governments plan for development of most land vulnerable to rising sea level along the US Atlantic coast. Environmental Research Letters, 4(4), 044008. See Supplemental Methods §6.2. A one meter sea level rise will not submerge areas that are protected.
3. Florida counties along the Atlantic Ocean including Monroe.
4. Includes population of census blocks whose centers are over wetlands or areas where shore protection is prohibited, in addition to the three likelihood categories shown.

The purpose of this table is to compare changes in population within one meter above sea level in areas where shore protection is expected to changes in population where shore protection is unlikely.

Table S14-B. Change In Black Population of Land Less than One Meter Above Sea Level Along US Atlantic Coast, by State and Likelihood of Shore Protection[1]											
	**Where Shore Protection is Unlikely[2]**						**Where Shore Protection is Likely[2]**				
	**1990**	**2000**	**2010**	**2020**	**Change**		**1990**	**2000**	**2010**	**2020**	**Change**
**MA**	15	30	9	12	-3		17	43	49	65	48
**RI**	3	5	7	4	1		1	1	3	3	3
**CT**	0	1	0	1	1		15	7	5	7	-8
**NY**	20	51	145	170	150		11	13	23	25	15
**NJ**	86	66	74	59	-26		326	430	225	267	-59
**PA**	6	29	1	4	-2		8	31	16	6	-3
**DE**	24	28	28	42	18		109	50	44	103	-6
**DC**	0	0	0	0	0		0	0	0	0	0
**VA**	185	156	117	125	-60		563	675	457	493	-70
**MD**	478	376	286	229	-249		394	374	296	214	-180
**NC**	2,002	1,896	1,582	1,159	-843		340	194	255	189	-151
**SC**	823	753	573	478	-346		1,909	1,508	1,369	1,250	-659
**GA**	19	61	137	85	66		93	74	234	206	113
**FLE**	38	78	65	129	92		97	67	66	118	20
**Atlantic**	3,698	3,530	3,024	2,499	-1,200		3,882	3,469	3,042	2,947	-935
	**Where Shore Protection is Almost Certain[2]**						**All Lands[4]**				
**MA**	5,420	4,886	4,859	5,399	-21		5,456	4,974	4,924	5,497	41
**RI**	16	19	9	22	6		21	28	18	30	9
**CT**	1,354	1,342	1,047	1,054	-300		1,380	1,359	1,071	1,090	-290
**NY**	12,771	14,713	13,901	16,568	3,798		12,862	14,880	14,099	16,771	3,910
**NJ**	13,039	12,799	11,617	11,205	-1,833		13,653	13,647	12,046	11,659	-1,994
**PA**	1,303	972	961	870	-434		1,340	1,040	1,037	882	-457
**DE**	452	359	658	580	128		584	466	743	740	156
**DC**	33	27	144	87	53		33	27	144	87	53
**VA**	4,166	4,681	4,440	4,726	560		4,946	5,570	5,087	5,402	456
**MD**	1,648	1,738	1,671	1,635	-14		2,750	2,604	2,362	2,185	-565
**NC**	4,163	3,928	3,273	2,132	-2,031		6,532	6,063	5,131	3,560	-2,972
**SC**	6,042	5,680	5,211	4,211	-1,831		8,827	7,948	7,200	5,970	-2,858
**GA**	979	1,157	1,071	1,191	212		1,320	1,699	1,691	2,057	738
**FLE[3]**	35,344	45,206	50,207	51,059	15,716		38,537	49,175	56,502	58,540	20,002
**Atlantic**	86,729	97,509	99,070	100,740	14,011		98,241	109,479	112,054	114,470	16,229

1. Sea level of 2020
2. Shore Protection Likelihood from Titus, J. G., et al. 2009. State and local governments plan for development of most land vulnerable to rising sea level along the US Atlantic coast. Environmental Research Letters, 4(4), 044008. See Supplemental Methods §6.2 A one meter sea level rise will not submerge areas that are protected.
3. Florida counties along the Atlantic Ocean including Monroe.
4. Includes population of census blocks whose centers are over wetlands or areas where shore protection is prohibited, in addition to the three likelihood categories shown.

.

The purpose of this table is to compare changes in Black population within one meter above sea level in areas where shore protection is expected to changes in population where shore protection is unlikely.

Table S14-C: Change in Population of Land Less than One Meter Above Sea Level[1] in Atlantic Counties with Greatest Emigration,[2] by Likelihood of Shore Protection[3]

		Where Shore Protection is Almost Certain[3]					Where Shore Protection is Likely[3]				
**County**		**1990**	**2000**	**2010**	**2020**	**Change**	**1990**	**2000**	**2010**	**2020**	**Change**
Hyde	NC	1243	1,210	1,213	1,205	-38	0	144	0	0	0
Tyrell	NC	2250	2,446	2,643	1,694	-556	108	125	206	147	39
Somerset	MD	3,812	3,564	3,264	2,869	-944	2,656	2,688	2,812	2,762	106
Pamlico	NC	475.5	453	384	351	-125	72	63	101	99	27
Cape May	NJ	17,869	18,605	15,611	14,827	-3,041	518	422	251	218	-300
Beaufort	NC	3946	3,411	3,077	2,733	-1,213	210	284	309	363	154
Accomack	VA	4,557	4,985	4,095	4,087	-470	531	571	480	407	-124
Dorchester	MD	1,124	964	980	1,081	-42	21	61	19	39	19
Salem	NJ	7,692	6,644	6,903	7,139	-554	705	904	556	553	-152
Mathews	VA	521	434	475	471	-50	567	761	630	794	227
Gloucester	VA	516	464	440	485	-32	1,018	960	928	831	-188
Talbot	MD	954	853	763	647	-307	49	30	21	59	10
Washington	NC	240	180	183	118	-122	80	98	95	69	-11
Pasquotank	NC	1193	1,044	965	880	-313	11	7	5	5	-7
Northumberland	VA	144	216	164	142	-2	130	131	111	76	-54
		**Where Shore Protection is Unlikely[3]**					**Blocks with Centroids Over Wetlands[3]**				
Hyde	NC	2,638	2,520	2,333	1,775	-862	273	109	198	56	-217
Tyrell	NC	232	275	250	108	-124	0	0	4	81	81
Somerset	MD	737	714	501	546	-191	202	228	198	204	1
Pamlico	NC	724	630	560	428	-296	25	21	12	10	-15
Cape May	NJ	453	523	761	1,032	578	102	233	134	47	-55
Beaufort	NC	403	363	436	362	-41	61	59	103	26	-35
Accomack	VA	644	636	423	503	-141	-19	-24	33	13	32
Dorchester	MD	2,471	2,632	2,449	2,073	-398	346	302	175	125	-221
Salem	NJ	410	447	347	334	-76	498	680	319	60	-438
Mathews	VA	547	595	554	301	-246	91	109	13	19	-72
Gloucester	VA	646	583	373	434	-212	1	28	27	17	16
Talbot	MD	97	88	70	96	-2	37	107	106	58	21
Washington	NC	6	10	12	12	6	0	0	0	0	0
Pasquotank	NC	662	700	760	716	54	0	9	0	15	15
Northumberland	VA	11	10	7	7	-4	48	64	35	30	-18

1. Sea Level of 2020
2. Ranked By Total Emigration from Land Below 1 m as a percent of total county population. See Tables 4 and S8-A.
3. Shore Protection Likelihood from Titus, J. G., et al. 2009. State and local governments plan for development of most land vulnerable to rising sea level along the US Atlantic coast. Environmental Research Letters, 4(4), 044008. See Supplemental Methods §6.2. These results are based on the estimated likelihood of shore protection of the centroid of a given block, except for where the centroid of the block is over wetlands or open water.

.

Table S14-C (continued): Change in Population of Land Less than One Meter Above Sea Level in Atlantic Counties with Greatest Emigration,[1] by Likelihood of Shore Protection[2]

		**Where Shore Protection is Almost Certain[2]**					**Where Shore Protection is Likely[2]**				
**County**		**1990**	**2000**	**2010**	**2020**	**Change**	**1990**	**2000**	**2010**	**2020**	**Change**
Atlantic	NJ	24,781	26,636	25,482	23,318	-1,463	731	946	699	615	-116
Lancaster	VA	329	337	329	201	-128	35	43	41	10	-24
Carteret	NC	3128	2,978	2,558	2,373	-755	326	464	535	821	495
Portsmouth	VA	1,581	1,734	1,120	1,346	-236	0	0	0	1	1
Harford	MD	19	20	40	39	20	267	293	19	18	-249
Westmoreland	VA	114	118	122	72	-42	13	16	19	20	6
King George	VA	35	40	35	7	-29	3	4	8	8	4
Newport News	VA	341	283	325	254	-87	424	352	228	201	-223
Middlesex	VA	106	106	104	105	0	43	49	54	28	-16
Ocean	NJ	15,900	17,464	15,182	14,730	-1,170	1,054	1,012	975	1,243	190
Monmouth	NJ	11,858	11,910	10,932	10,882	-976	24	21	16	16	-9
Kent	MD	248	252	191	205	-44	4	8	3	0	-4
Newport	RI	504	489	358	382	-122	13	9	1	6	-7
Nantucket	MA	100	184	45	75	-25	35	13	52	48	13
Caroline	MD	73	62	58	33	-40	7	5	0	3	-3
		**Where Shore Protection is Unlikely[2]**					**Blocks with Centroids Over Wetlands[2]**				
Atlantic	NJ	326	276	306	412	86	157	114	180	168	12
Lancaster	VA	28	5	11	104	76	58	111	37	75	16
Carteret	NC	1,845	2,045	2,266	2,204	359	501	348	312	149	-352
Portsmouth	VA	0	0	0	0	0	172	16	30	33	-138
Harford	MD	13	3	4	4	-9	432	60	15	21	-411
Westmoreland	VA	27	35	20	20	-7	24	21	32	22	-2
King George	VA	0	1	4	1	1	139	145	146	119	-20
Newport News	VA		6		0		20	5	0	-3	-22
Middlesex	VA	12	34	22	27	14	34	28	53	20	-14
Ocean	NJ	375	350	355	498	124	276	362	571	383	107
Monmouth	NJ	19	19	16	25	7	32	55	98	106	74
Kent	MD	50	66	61	45	-4	2	8	17	26	24
Newport	RI	28	41	48	11	-17	50	42	59	74	24
Nantucket	MA	0	45	9	11	11	25	4	12	18	-7
Caroline	MD	56	60	59	63	7	0	1	0	2	2

1. Ranked By Total Emigration from Land Below 1 m as a percent of total county population. See Tables 4 and S8-A.
2. Shore Protection Likelihood from Titus, J. G., et al. 2009. State and local governments plan for development of most land vulnerable to rising sea level along the US Atlantic coast. Environmental Research Letters, 4(4), 044008. See Supplemental Methods §6.2. These results are based on the estimated likelihood of shore protection of the centroid of a given block, except for where the centroid of the block is over wetlands or open water. .

Table S14-D: Change In Black Population of Land Less than One Meter Above Sea Level[2] in Atlantic Counties with Greatest Emigration,[2] by Likelihood of Shore Protection[3]

		Where Shore Protection is Almost Certain[3]					Where Shore Protection is Likely[3]				
**County**		**1990**	**2000**	**2010**	**2020**	**Change**	**1990**	**2000**	**2010**	**2020**	**Change**
Hyde	NC	323	246	186	125	-199	0	8	0	0	0
Tyrell	NC	937	1,056	1,082	479	-458	33	5	92	36	2
Somerset	MD	993	1,000	941	789	-203	289	260	262	173	-116
Pamlico	NC	104	104	60	61	-43	27	20	16	9	-17
Cape May	NJ	1,238	1,032	629	432	-806	1	5	0	1	0
Beaufort	NC	1,576	1,262	943	691	-885	28	19	29	26	-2
Accomack	VA	43	70	51	61	18	6	23	23	17	11
Dorchester	MD	11	32	38	34	23	1	5	0	7	6
Salem	NJ	1,336	1,149	1,342	1,590	254	249	322	124	126	-122
Mathews	VA	23	13	19	9	-14	12	47	23	39	26
Gloucester	VA	14	21	9	19	5	21	13	10	14	-7
Talbot	MD	61	51	32	22	-39	0	1	1	1	1
Washington	NC	147	100	111	58	-89	32	43	24	15	-16
Pasquotank	NC	513	598	463	395	-118	1	1	0	0	-1
Northumberland	VA	4	9	8	11	7	10	6	10	6	-5
		**Where Shore Protection is Unlikely[3]**					**Blocks with Centroids Over Wetlands[3]**				
Hyde	NC	1068	1030	770	497	-571	14	10	4	8	-6
Tyrell	NC	192	188	137	56	-136	0	0	0	53	53
Somerset	MD	78	81	46	66	-13	33	26	14	20	-14
Pamlico	NC	69	65	48	38	-31	0	0	1	0	0
Cape May	NJ	24	20	33	25	1	0	9	-1	8	8
Beaufort	NC	113	67	41	20	-93	2	1	0	1	-1
Accomack	VA	77	64	37	63	-14	0	-1	0	0	0
Dorchester	MD	188	132	115	66	-122	2	6	-5	5	3
Salem	NJ	18	12	12	8	-10	154	288	96	87	-67
Mathews	VA	13	15	8	4	-9	0	9	0	2	2
Gloucester	VA	18	9	17	8	-10	1	1	2	0	0
Talbot	MD	12	8	4	4	-8	4	2	4	0	-4
Washington	NC	0	1	1	1	1	0	0	0	0	0
Pasquotank	NC	151	129	148	129	-21	0	2	0	3	3
Northumberland	VA	1	1	1	1	0	6	6	2	-4	-9

1. Sea level of 2020
2. Ranked By Total Emigration from Land Below 1 m as a percent of total county population. See Tables 4 and S8-A.
3. Shore Protection Likelihood from Titus, J. G., et al. 2009. State and local governments plan for development of most land vulnerable to rising sea level along the US Atlantic coast. Environmental Research Letters, 4(4), 044008. See Supplemental Methods §6.2. These results are based on the estimated likelihood of shore protection of the centroid of a given block, except for where the centroid of the block is over wetlands or open water. A one meter sea level rise will not submerge areas that are protected.

.

Table S14-D (continued): Change In Black Population of Land Less than One Meter Above Sea Level in Atlantic Counties with Greatest Emigration,[1] by Likelihood of Shore Protection[2]

		**Where Shore Protection is Almost Certain[2]**					**Where Shore Protection is Likely[2]**				
**County**		**1990**	**2000**	**2010**	**2020**	**Change**	**1990**	**2000**	**2010**	**2020**	**Change**
Atlantic	NJ	7,896	7,308	6,377	5,569	-2,328	7	8	14	6	-1
Lancaster	VA	6	8	19	15	10	4	3	2	2	-2
Carteret	NC	371	321	202	140	-231	28	35	35	35	7
Portsmouth	VA	557	540	358	463	-94	0	0	0	0	0
Harford	MD	2	2	6	5	3	61	91	0	0	-61
Westmoreland	VA	3	7	9	9	6	3	2	2	2	-1
King George	VA	3	4	3	1	-2	0	0	1	1	1
Newport News	VA	116	75	136	80	-36	289	250	157	152	-136
Middlesex	VA	4	1	2	3	-1	2	2	1	1	-1
Ocean	NJ	67	103	121	100	32	0	1	12	14	13
Monmouth	NJ	293	324	481	583	290	0	0	0	0	0
Kent	MD	15	15	4	7	-7	0	0	0	0	0
Newport	RI	14	16	5	5	-9	0	0	0	0	0
Nantucket	MA	2	65	2	8	6	1	0	5	3	2
Caroline	MD	9	9	12	3	-6	0	0	0	0	0
		**Where Shore Protection is Unlikely[2]**					**Blocks with Centroids Over Wetlands[2]**				
Atlantic	NJ	16	2	11	8	-8	-9	3	-4	9	18
Lancaster	VA	6	3	6	10	4	189	182	249	203	14
Carteret	NC	195	184	247	210	15	-184	-183	-247	-204	-20
Portsmouth	VA	0	0	0	0	0	112	3	2	2	-109
Harford	MD	0	0	0	0	0	15	13	6	5	-10
Westmoreland	VA	12	10	5	3	-9	6	8	7	12	6
King George	VA	0	0	0	0	0	1	0	-3	-1	-1
Newport News	VA		0		0		3	6	4	4	1
Middlesex	VA	3	5	2	4	1	-1	-3	4	0	1
Ocean	NJ	1	1	5	4	3	-1	-1	-4	-2	-1
Monmouth	NJ	0	0	1	3	3	1	4	3	-1	-2
Kent	MD	1	5	3	2	1	-1	-4	-3	-1	0
Newport	RI	0	0	0	0	0	0	4	0	0	0
Nantucket	MA	0	3	0	0	0	13	6	4	7	-6
Caroline	MD	13	9	4	7	-6	-13	-9	-4	-7	6

1. Ranked By Total Emigration from Land Below 1 m as a percent of total county population. See Tables 4 and S8-A.
2. Shore Protection Likelihood from Titus, J. G., et al. 2009. State and local governments plan for development of most land vulnerable to rising sea level along the US Atlantic coast. Environmental Research Letters, 4(4), 044008. See Supplemental Methods §6.2. These results are based on the estimated likelihood of shore protection of the centroid of a given block, except for where the centroid of the block is over wetlands or open water. A one meter sea level rise will not submerge areas that are protected.

Table of Abbreviations Used in these Tables								
**States in these Tables**								
Abbreviation	Meaning		Abbreviation	Meaning		Abbreviation	Meaning
AK	Alaska		KY	Kentucky		NY	New York
AL	Alabama		LA	Louisiana		OH	Ohio
AR	Arkansas		MA	Massachusetts		OK	Oklahoma
AZ	Arizona		MD	Maryland		OR	Oregon
CA	California		ME	Maine		PA	Penslvania
CO	Colorado		MI	Michigan		RI	Rhode Island
CT	Connecticut		MN	Minnesota		SC	South Carolina
DC	District of Columbia		MO	Missouri		SD	South Dakota
DE	Delaware		MS	Mississippi		TN	Tennessee
FL	Florida		MT	Montana		TX	Texas
GA	Georgia		NC	North Carolina		UT	Utah
HI	Hawaii		ND	North Dakota		VA	Virginia
IA	Iowa		NE	Nebraska		VT	Vermont
ID	Idaho		NH	New Hampshire		WA	Washington
IL	Illinois		NJ	New Jersey		WI	Wisconsin
IN	Indiana		NM	New Mexico		WV	West Virginia
KS	Kansas		NV	Nevada		WY	Wyoming
**Other**							
A-zone	FEMA mapped floodplains generally having a 1% chance of flooding each year.						
Below one meter	Less than one meter above mean higher high water						
FLE	Counties in Florida that drain into the Alantic Ocean						
FLW	Counties in Florida that drain into the Gulf of Mexico but not the Atlantic Ocean.						
HOLC	Home Owners Loan Corporation, the government entity that created the redline maps.						
MHHW	Mean Higher High Water						
Sea level	Mean Higher High Water						
V-zone	FEMA mapped floodplains where property may be subjected to waves or strong currents.						
X500 Zone	FEMA mapped floodplains generally having a 0.2% chance of flooding each year.						

References

Federal Emergency Management Agency 2021 National flood hazard layer (available at: www.fema.gov/flood-maps/national-flood-hazard-layer) (Accessed 12 December 2021)

Nelson, R.K., Winling, L., Marciano, R., Connolly, N. and Ayers, E.L., 2021. Mapping inequality. *American panorama*.

Strauss et al., 2012. "Tidally adjusted estimates of topographic vulnerability to sea level rise and flooding for the contiguous United States." Environmental Research Letters 7, no. 1 (2012): 014033

Titus, J.G., 2023. Population in floodplains or close to sea level increased in US but declined in some counties—especially among Black residents. *Environmental Research Letters*, *18*(3), p.034001. https://doi.org/10.1088/1748-9326/acadf5

Titus, J.G., 2023a. Supplemental Tables (Part 2: Landscape) from 'Population in floodplains or close to sea level increased in US but declined in some counties–especially among Black residents'. Environmental Research Letters.

Titus, J. G., et al. 2009. State and local governments plan for development of most land vulnerable to rising sea level along the US Atlantic coast. Environmental Research Letters, 4(4), 044008.

Zavar, E. and Fischer, L.A., 2021. Fractured landscapes: The racialization of home buyout programs and climate adaptation. *Current Research in Environmental Sustainability*, *3*, p.100043.
