# Supplementary material for: Population in floodplains or close to sea level increased in US but declined in some counties—especially among Black residents
Source: Environ Res Lett. Author manuscript; Available in PMC 2025 Mar 14. (PMC11908447; doi:10.1088/1748-9326/acadf5)
Supplement: Supplemental Tables (landscape) [file NIHMS1876714-supplement-Supplemental_Tables__landscape_.pdf]

Supplemental Tables (Part 2: Landscape) from 'Population in floodplains or close to sea level increased in US but declined in some counties–especially among Black residents'

JAMES G TITUS

ENVIRONMENTAL RESEARCH LETTERS

FEBRUARY 2023

Note: This document includes only Tables S8, S11, S12, and part of S9 which were produced in landscape format; Part 1 has the tables formatted in portrait. Most of the tables provide estimates of changes in the population vulnerable to sea level rise or flooding. See Table of Tables in Part 1. For more complete results by state or by county, go to EPA's Science Hub at https://doi.org/10.23719/1527848

TABLE OF SUPPLEMENTAL TABLES

S8-A Apparent Emigration 1990–2020 from Land Below 1 meter by County, Ranked as a Percent of 1990 Population

S8-B Apparent Emigration 1990–2020 from Land Below 3 meters by County, Ranked as a Percent of 1990 Population

S8-C Apparent Black Emigration 1990–2020 from Land Below 1 meter by County, Ranked as a Percent of 1990 Population

S8-D Apparent Black Emigration 1990–2020 from Land Below 3 meters by County, Ranked as a Percent of 1990 Population

S8-E Apparent Emigration 1990–2020 from Inland 100-Year Floodplain by County, Ranked[1] as a Percent of 1990 Population

S8-F Apparent Emigration 1990–2020 from Coastal 100-Year Floodplains by County, Ranked[1] as a Percent of 1990 Population

S8-G Apparent Emigration 1990–2020 from 500-Year Floodplain by County, Ranked[1] by Emigration from X500 Zone

S8-H Apparent Emigration 1990–2020 by County from 100-Year Floodplain, Ranked[1] by Total Emigration

S8-J Apparent Emigration 1990–2020 from Floodplains and Lands Close to Sea Level

S9-A Summary Statistics Measuring whether Blacks Account for a Disproportionate Share of People Inhabiting Low Land Who Emigrate from Low Land, in Counties with Net Emigration from Low Land, by Decade

S9-B Summary Statistics Measuring whether Blacks Account for a Disproportionate Share of People Inhabiting Floodplains Who Emigrate from Floodplains, in Counties with Net Emigration from Floodplains, by Decade

S9-C Summary Statistics Measuring whether Blacks Account for a Disproportionate Share of Emigration from Low Land in Counties with Net Emigration by Decade

S9-D Summary Statistics Measuring whether Blacks Account for a Disproportionate Share of Emigration from Floodplains in Counties with Net Emigration by Decade

S8-A Apparent Emigration 1990–2020 from Land Below 1 meter by County, Ranked as a Percent of 1990 Population

S8-B Apparent Emigration 1990–2020 from Land Below 3 meters by County, Ranked as a Percent of 1990 Population

S8-C Apparent Black Emigration 1990–2020 from Land Below 1 meter by County, Ranked as a Percent of 1990 Population

S8-D Apparent Black Emigration 1990–2020 from Land Below 3 meters by County, Ranked as a Percent of 1990 Population

S8-E Apparent Emigration 1990–2020 from Inland 100-Year Floodplain by County, Ranked[1] as a Percent of 1990 Population

S8-F Apparent Emigration 1990–2020 from Coastal 100-Year Floodplains by County, Ranked[1] as a Percent of 1990 Population

S8-G Apparent Emigration 1990–2020 from 500-Year Floodplain by County, Ranked[1] by Emigration from X500 Zone

S8-H Apparent Emigration 1990–2020 by County from 100-Year Floodplain, Ranked[1] by Total Emigration

S8-J Apparent Emigration 1990–2020 from Floodplains and Lands Close to Sea Level

S9-A Summary Statistics Measuring whether Blacks Account for a Disproportionate Share of People Inhabiting Low Land Who Emigrate from Low Land, in Counties with Net Emigration from Low Land, by Decade

S9-B Summary Statistics Measuring whether Blacks Account for a Disproportionate Share of People Inhabiting Floodplains Who Emigrate from Floodplains, in Counties with Net Emigration from Floodplains, by Decade

S9-C Summary Statistics Measuring whether Blacks Account for a Disproportionate Share of Emigration from Low Land in Counties with Net Emigration by Decade

S9-D Summary Statistics Measuring whether Blacks Account for a Disproportionate Share of Emigration from Floodplains in Counties with Net Emigration by Decade

TABLE S8-A Apparent Emigration 1990–2020 from Land Below 1 meter by County, Ranked as a Percent of 1990 Population

	State	Apparent Migration[1]					Δ Population below 1m [2]					County Population In 2020		Migration as % of county population[3]		
County		1990 to 2020	1990s	2000s	2010s		1990 to 2020	1990s	2000s	2010s		<1m	Total		Total	Black
Cameron	LA	-2,656	-61	-2,255	-340		-1,767	208	-1,825	-149		1,523	5,617		-28.7	-42.9
St. Bernard	LA	-18,356	319	-25,638	6,963		-15,513	1,290	-24,381	7,578		34,743	43,764		-27.5	249.2
Hyde	NC	-1,117	-170	-238	-708		-776	-42	-113	-621		3,036	4,589		-20.6	-43.6
Orleans	LA	-100,979	-11,361	-130,806	41,188		-91,115	-7,862	-127,202	43,949		333,352	383,997		-20.3	-27.3
Tyrrell	NC	-560	257	257	-1,073		-265	348	353	-966		2,030	3,245		-14.5	-34.9
Plaquemines	LA	-2,040	1,076	-3,690	574		-1,677	1,208	-3,581	696		21,610	23,515		-8.0	-15.8
St. Mary	LA	-2,713	-1,997	1,172	-1,889		2,612	-136	2,679	69		21,632	49,406		-4.7	-3.7
Somerset	MD	-1,028	-213	-419	-395		-454	-9	-200	-245		6,380	24,620		-4.4	-3.9
Pamlico	NC	-408	-129	-109	-170		-214	-75	-43	-97		888	12,276		-3.6	-3.1
Cape May	NJ	-2,819	840	-3,025	-634		1,187	2,138	-1,452	501		16,124	95,263		-3.0	-15.3
Beaufort	NC	-1,136	-502	-193	-441		-299	-196	89	-193		3,484	44,652		-2.7	-7.5
Jefferson	TX	-6,393	-2,225	-3,182	-986		-1,377	-683	-1,624	930		29,963	256,526		-2.7	-10.2
Accomack	VA	-703	455	-1,137	-20		148	776	-830	202		5,010	33,413		-2.2	0.1
Dorchester	MD	-642	-3	-335	-305		-256	133	-216	-173		3,318	32,531		-2.1	-1.1
Jefferson	LA	-8,454	5,644	-21,027	6,929		4,952	10,146	-16,267	11,073		391,888	440,781		-1.9	43.3
Salem	NJ	-1,220	-631	-548	-41		158	-133	-99	391		8,086	64,837		-1.9	0.6
Vermilion	LA	-933	-174	-501	-258		402	223	-26	205		2,706	57,359		-1.9	0.5
Mathews	VA	-141	173	-227	-87		269	279	-64	54		1,585	8,533		-1.7	0.4
Dixie	FL	-171	-53	-61	-58		-140	-42	-59	-39		544	16,759		-1.6	0.2
Gloucester	VA	-416	-147	-267	-1		19	12	-113	119		1,767	38,711		-1.4	-0.4
Terrebonne	LA	-1,024	-241	1,991	-2,773		7,561	2,326	4,510	725		38,894	109,580		-1.1	7.1
Talbot	MD	-278	-58	-119	-101		-32	32	-38	-26		859	37,526		-0.9	-0.9
Washington	NC	-127	-39	3	-91		-47	-15	27	-60		199	11,003		-0.9	-1.6
Pasquotank	NC	-250	-105	-31	-113		304	81	154	69		1,616	40,568		-0.8	-1.2
Northumberland	VA	-78	87	-103	-63		-35	98	-86	-47		255	11,839		-0.7	-0.2
Atlantic	NJ	-1,481	1,977	-1,305	-2,153		6,418	4,108	1,832	477		24,514	274,534		-0.7	-6.2
Citrus	FL	-518	-192	-63	-263		52	-17	131	-63		3,958	153,843		-0.6	-2.3
Lancaster	VA	-60	46	-78	-28		31	69	-37	-1		389	10,919		-0.6	0.5

TABLE S8-A Apparent Emigration 1990–2020 from Land Below 1 meter by County, Ranked as a Percent of 1990 Population

County	State	Apparent Migration[1]				Δ Population below 1m [2]				County Population In 2020		Migration as % of county population[3]	
		1990 to 2020	1990s	2000s	2010s	1990 to 2020	1990s	2000s	2010s	<1m	Total	Total	Black
Carteret	NC	-253	34	-164	-124	898	390	191	317	5,547	67,686	-0.5	-4.7
Portsmouth	VA	-374	-3	-600	229	246	206	-323	363	1,379	97,915	-0.4	-0.2
Harford	MD	-649	-354	-299	4	-582	-294	-298	10	82	260,924	-0.4	-1.1
St. Martin	LA	-130	104	-148	-86	256	246	0	10	1,244	51,767	-0.3	0.0
Westmoreland	VA	-44	12	2	-59	-16	18	12	-46	134	18,477	-0.3	-0.1
Jefferson Davis	LA	-82	-65	-26	9	48	-2	7	43	229	32,250	-0.3	-1.4
Iberia	LA	-173	15	-214	26	315	122	-13	206	1,155	69,929	-0.3	0.1
King George	VA	-33	-3	16	-47	-30	-1	16	-45	15	26,723	-0.2	-0.1
Bay	FL	-298	33	-14	-317	-72	76	55	-203	1,292	175,216	-0.2	0.1
Jackson	MS	-269	282	-475	-77	-51	299	-350	0	1,003	143,252	-0.2	0.3
Newport News	VA	-333	-138	-93	-101	-193	-73	-46	-74	452	186,247	-0.2	-0.3
Aransas	TX	-35	118	-96	-56	151	140	-4	15	442	23,830	-0.2	-1.0
Middlesex	VA	-16	22	15	-53	15	27	28	-40	179	10,625	-0.2	0.0
Ocean	NJ	-749	1,583	-2,105	-227	5,053	3,103	232	1,717	16,855	637,229	-0.2	0.4
Monmouth	NJ	-904	73	-944	-33	1,894	1,069	3	822	11,028	643,615	-0.2	0.6
Hancock	MS	-50	917	-977	10	326	913	-710	122	1,433	46,053	-0.2	0.3
Kent	MD	-28	31	-63	5	47	56	-37	28	276	19,198	-0.2	-0.2
Knox	ME	-54	-5	-22	-26	-42	1	-19	-23	102	40,607	-0.1	-0.1
Newport	RI	-121	-13	-115	7	-20	22	-80	37	474	85,643	-0.1	-0.3
Nantucket	MA	-8	86	-127	33	13	79	-102	36	152	14,255	-0.1	6.3
Iberville	LA	-40	105	-55	-89	302	176	93	33	729	30,241	-0.1	0.0
Caroline	MD	-34	-7	-11	-15	-15	-2	-5	-8	102	33,293	-0.1	-0.3
Northampton	VA	-16	27	-49	6	52	42	-15	26	217	12,282	-0.1	-0.2
Sarasota	FL	-331	-276	-572	517	1,778	435	149	1,194	12,160	434,006	-0.1	0.2
Solano	CA	-397	-257	-69	-70	-301	-189	-69	-42	622	453,491	-0.1	-0.1
New London	CT	-295	-244	-27	-24	-120	-172	20	31	775	268,555	-0.1	-0.1
Gloucester	NJ	-238	-130	-93	-15	56	-1	7	51	1,613	302,294	-0.10	0.0
Norfolk	VA	-229	-628	-472	870	1,891	-5	309	1,588	7,850	238,005	-0.09	0.5

TABLE S8-A Apparent Emigration 1990–2020 from Land Below 1 meter by County, Ranked as a Percent of 1990 Population

	State	Apparent Migration[1]				Δ Population below 1m [2]				County Population In 2020		Migration as % of county population[3]	
County		1990 to 2020	1990s	2000s	2010s	1990 to 2020	1990s	2000s	2010s	<1m	Total	Total	Black
Levy	FL	-23	-4	-19	1	31	17	-8	23	557	42,915	-0.09	0.1
Pinellas	FL	-739	1,412	-1,359	-792	8,341	4,191	1,809	2,340	43,872	959,107	-0.09	0.6
Okaloosa	FL	-119	97	68	-285	13	126	106	-219	768	211,668	-0.08	0.0
Cumberland	NJ	-111	-50	-2	-58	127	34	68	25	1,249	154,152	-0.08	0.0
Multnomah	OR	-464	51	-35	-480	-464	51	-35	-480	655	815,428	-0.08	0.0
Philadelphia	PA	-1,191	-1,049	193	-335	-602	-709	321	-214	1,176	1,603,797	-0.08	-0.1
Hertford	NC	-17	7	-25	1	-12	9	-24	3	61	21,552	-0.08	0.0
Isle of Wight	VA	-18	16	13	-47	-10	17	16	-44	58	38,606	-0.07	0.0
Hancock	ME	-30	6	-12	-25	-25	7	-10	-22	87	55,478	-0.06	0.2
Mendocino	CA	-52	-48	-5	2	-51	-48	-3	0	3	91,601	-0.06	0.0
Lincoln	ME	-18	-4	-11	-4	-14	-2	-7	-5	102	35,237	-0.06	5.9
Jefferson	WA	-12	-4	-1	-7	-12	-4	-1	-7	25	32,977	-0.06	0.4
Orange	CA	-1,360	-908	-490	37	-68	-505	-77	515	20,016	3,186,989	-0.06	0.1
Escambia	FL	-144	-112	-125	93	215	43	-26	199	1,129	321,905	-0.05	0.0
Surry	VA	-3	4	-5	-2	2	4	-5	3	49	6,561	-0.05	0.0
Washington	ME	-18	5	-4	-19	-14	7	-4	-17	51	31,095	-0.05	1.7
Brazoria	TX	-91	1,519	-679	-930	796	1,637	-285	-556	10,955	372,031	-0.05	-2.7
Orange	TX	-38	9	-84	37	140	58	-4	86	362	84,808	-0.05	0.0
Mason	WA	-18	-10	-13	6	-17	-10	-13	6	41	65,726	-0.05	0.0
Columbia	OR	-17	48	-45	-19	-15	49	-45	-19	177	52,589	-0.04	2.4
Anne Arundel	MD	-187	139	-127	-199	490	298	203	-10	2,350	588,261	-0.04	-0.1
Charles City	VA	-3	0	-3	0	-1	0	-2	1	15	6,773	-0.04	-0.1
Craven	NC	-34	4	36	-75	73	35	64	-26	314	100,720	-0.04	-0.1
Hampton	VA	-55	784	-942	103	980	930	-404	454	2,937	137,148	-0.04	0.5
Napa	CA	-45	4	-39	-10	-42	5	-39	-9	128	138,019	-0.04	0.0
Washington	RI	-45	81	-115	-11	51	111	-79	19	582	129,839	-0.04	0.0
New Haven	CT	-306	-140	27	-193	187	41	193	-47	3,383	864,835	-0.04	0.1
Bertie	NC	-6	2	0	-8	2	5	4	-6	32	17,934	-0.03	0.0

TABLE S8-A Apparent Emigration 1990–2020 from Land Below 1 meter by County, Ranked as a Percent of 1990 Population

	State	Apparent Migration[1]				Δ Population below 1m [2]				County Population In 2020		Migration as % of county population[3]	
County		1990 to 2020	1990s	2000s	2010s	1990 to 2020	1990s	2000s	2010s	<1m	Total	Total	Black
Kent	DE	-33	-4	7	-37	83	37	51	-5	801	181,851	-0.03	0.1
Middlesex	NJ	-167	-225	159	-101	68	-184	257	-5	831	863,162	-0.02	0.1
Island	WA	-14	-3	4	-15	-11	-2	5	-14	31	86,857	-0.02	0.0
Burlington	NJ	-87	-39	-65	17	305	95	53	158	2,101	461,860	-0.02	0.2
Clatsop	OR	-7	-45	-24	62	-41	-55	-35	49	462	41,072	-0.02	0.3
Nassau	NY	-262	261	-906	383	6,694	2,425	1,524	2,745	29,109	1,395,774	-0.02	1.2
Thurston	WA	-32	-3	-1	-28	-31	-3	-1	-27	17	294,793	-0.02	0.0
Chowan	NC	-2	8	-1	-10	31	15	13	3	148	13,708	-0.02	0.0
Douglas	OR	-15	-38	20	3	-5	-31	21	6	44	111,201	-0.02	0.0
Humboldt	CA	-17	-59	22	20	83	-29	59	53	195	136,463	-0.01	-0.3
Kleberg	TX	-4	-5	0	0	-4	-4	0	0	0	31,040	-0.01	0.0
Jackson	TX	-1	0	-2	1	-1	0	-1	1	2	14,988	-0.01	0.0
Orange	NY	-29	-4	-30	5	-24	-3	-26	5	7	401,310	-0.01	0.0
Union	NJ	-44	-95	35	16	5	-83	41	47	163	575,345	-0.009	0.0
Waldo	ME	-3	-7	3	2	-2	-7	3	2	14	39,607	-0.008	0.0
New Castle	DE	-34	-359	351	-26	187	-271	299	160	1,134	570,719	-0.008	0.0
Clark	WA	-18	-12	-33	27	-18	-12	-33	27	33	503,311	-0.008	0.0
Lane	OR	-19	-10	-17	8	-19	-10	-17	8	14	382,971	-0.007	0.0
Washington	AL	-1	-1	1	-1	-1	-1	1	-1	1	15,388	-0.007	0.0
Rockland	NY	-17	79	12	-108	38	110	14	-86	319	338,329	-0.007	0.0
Greene	NY	-3	12	-11	-4	10	18	-7	-2	36	47,931	-0.006	-0.2
Wayne	GA	-1	-1	0	0	-1	-1	0	0	0	30,144	-0.004	0.0
Hanover	VA	-2	-1	0	-1	-2	-1	0	-1	1	109,979	-0.004	0.0
Los Angeles	CA	-317	10	-14	-314	-169	50	36	-255	1,095	10,014,009	-0.004	0.0
Bucks	PA	-19	-38	-17	37	7	-34	-3	44	147	646,538	-0.004	0.0
Jones	NC	0	2	-1	-1	0	2	0	-1	4	9,172	-0.003	0.0
Pierce	WA	-18	188	-116	-89	-15	188	-116	-88	263	921,130	-0.003	0.0
Liberty	TX	-2	0	-3	2	0	0	-2	2	6	91,628	-0.003	0.0

TABLE S8-A Apparent Emigration 1990–2020 from Land Below 1 meter by County, Ranked as a Percent of 1990 Population

County	State	Apparent Migration[1]					Δ Population below 1m [2]					County Population In 2020			Migration as % of county population[3]	
		1990 to 2020	1990s	2000s	2010s		1990 to 2020	1990s	2000s	2010s		<1m	Total		Total	Black
Delaware	PA	-14	-6	-25	17		59	15	6	38		254	576,830		-0.003	0.0
Pacific	WA	0	-3	-1	4		0	-3	-1	4		25	23,365		-0.003	0.3
San Luis Obispo	CA	-6	-5	0	-1		-5	-5	0	-1		2	282,424		-0.003	0.0
King	WA	-37	45	-64	-18		-36	45	-64	-18		186	2,269,675		-0.002	0.0
Rensselaer	NY	-3	-1	-1	-1		-2	-1	-1	-1		1	161,130		-0.002	0.0
Washington	FL	0	0	0	0		0	0	0	0		3	25,318		-0.002	0.0
Refugio	TX	0	-2	1	0		1	-1	1	0		2	6,741		-0.002	0.0
Petersburg	VA	0	0	0	0		0	0	0	0		0	33,458		-0.001	0.0
Prince George's	MD	-8	-1	1	-7		-7	-1	1	-7		3	967,201		-0.001	0.0
Penobscot	ME	-1	2	-6	3		-1	2	-6	3		6	152,199		-0.001	0.0
Lafayette	LA	-1	1	-2	0		-1	1	-2	0		5	241,753		-0.001	0.0
Harris	TX	-18	-179	-102	263		125	-127	-73	325		865	4,731,145		-0.001	0.0
Santa Barbara	CA	-2	4	-12	6		-2	4	-12	6		12	448,229		-0.001	0.0
Total[4]		-169,312	-24,887	-226,444	-29,611		381,764	169,396	-50,889	263,257		2,441,514	117,596,849		-0.07	-0.37

1. Change in population of land below or less than one meter above the sea level of 2020. Includes migration into and out of this land as well as births and deaths. Negative numbers mean apparent emigration out of this county's land below 1m. Calculations use building-based density assumption.
2. The difference between the population of land below or less than one meter above sea level at the end of the period and the population at the beginning of the period. Like apparent migration, it includes migration into and out of this land as well as births and deaths; but it also includes the population of the land that was more than 1m above sea level at the beginning of the period but less than 1m above sea level at the end of the period.
3. Equal to "Apparent Migration" for 1990 to 2020 as a percent of the population of 1990, with numerator and denominator using the same racial classification in both cases. The numerators reflect population changes whether positive or negative, and Black emigration in this case can be greater than total migration, in effect, including cases where Black people replace—or are replaced by—people of other races. Tables calculating Black composition of emigration, by contrast, exclude replacement and hence Black migration would be between zero and total migration.
4. Sum of "Apparent Migration" includes only those counties with net emigration from <1m for the particular decade(s) of the column. The sum of "Δ Population below 1m" includes all counties. Although these totals by definition only include coastal counties, for consistency across tables, the denominators for the migration % are national populations.

The purpose of this table is to identify counties where the population vulnerable to sea level rise is declining.

TABLE S8-B Apparent Emigration 1990–2020 from Land Below 3 meters by County, Ranked as a Percent of 1990 Population

County	State	Apparent Migration[1]				Δ Population below 3m [2]				County Population In 2020		Migration as % of county population[3]	
		1990 to 2020	1990s	2000s	2010s	1990 to 2020	1990s	2000s	2010s	<1m	Total	Total	Black
Cameron	LA	-3,739	518	-3,133	-1,123	-3,631	533	-3,081	-1,084	5,008	5,617	-40.4	-80.7
St. Bernard	LA	-22,258	-65	-29,976	7,783	-22,233	-55	-29,964	7,786	43,552	43,764	-33.4	267.9
Orleans	LA	-110,016	-15,948	-135,326	41,259	-109,671	-15,810	-135,195	41,334	375,491	383,997	-22.1	-31.1
Tyrrell	NC	-641	218	310	-1,169	-634	220	305	-1,160	3,128	3,245	-16.6	-40.4
Hyde	NC	-869	340	-24	-1,184	-864	340	-22	-1,182	4,456	4,589	-16.1	-36.0
St. Mary	LA	-7,373	-4,130	1,337	-4,580	-6,136	-3,654	1,743	-4,225	45,606	49,406	-12.7	-13.1
Plaquemines	LA	-1,928	1,344	-3,624	353	-1,926	1,342	-3,622	353	22,802	23,515	-7.5	-15.6
Washington	NC	-1,002	-96	-176	-729	-708	-8	-65	-635	3,032	11,003	-7.2	-9.0
Cape May	NJ	-6,337	2,470	-6,785	-2,021	-4,977	2,834	-6,323	-1,489	50,241	95,263	-6.7	-24.2
Somerset	MD	-1,346	184	-816	-714	-1,137	165	-654	-648	10,864	24,620	-5.7	-11.4
Salem	NJ	-3,367	-2,506	-377	-485	-2,713	-2,276	-165	-272	28,001	64,837	-5.2	2.7
Vermilion	LA	-2,444	777	-827	-2,393	391	1,662	193	-1,463	26,309	57,359	-4.9	-8.3
Beaufort	NC	-1,764	-358	-258	-1,148	-1,077	-128	-35	-914	14,118	44,652	-4.2	-14.3
Hampton	VA	-5,069	4,787	-8,625	-1,230	-487	6,373	-7,128	269	78,656	137,148	-3.8	9.5
Mathews	VA	-271	429	-364	-336	-127	467	-314	-281	5,963	8,533	-3.2	-32.1
Pamlico	NC	-363	619	-211	-770	-223	645	-150	-718	5,698	12,276	-3.2	-32.9
Jackson	MS	-3,262	862	-4,047	-77	-236	1,814	-3,012	962	15,150	143,252	-2.8	3.9
Northampton	VA	-336	-236	-248	148	-245	-218	-214	186	1,974	12,282	-2.6	-9.0
Portsmouth	VA	-2,624	-3,467	-809	1,651	2,302	-1,553	746	3,109	54,032	97,915	-2.5	2.5
Atlantic	NJ	-5,393	5,365	-5,705	-5,053	-4,747	5,536	-5,464	-4,819	75,931	274,534	-2.4	-11.9
Gulf	FL	-273	-62	-216	6	-100	18	-166	47	3,772	14,192	-2.4	-10.3
Iberville	LA	-649	555	-69	-1,135	-157	553	167	-877	4,565	30,241	-2.1	-2.2
Accomack	VA	-657	554	-1,450	238	-492	615	-1,398	291	9,749	33,413	-2.1	-4.2
Orange	TX	-1,620	-149	-3,951	2,480	1,895	909	-2,709	3,695	25,486	84,808	-2.0	-5.0
Jefferson	LA	-8,041	5,947	-20,728	6,741	-6,879	6,300	-20,377	7,198	432,407	440,781	-1.8	46.9
Harrison	MS	-2,921	67	-4,514	1,526	-921	783	-3,726	2,022	10,690	208,621	-1.8	-2.5
Dixie	FL	-186	-119	-42	-24	-163	-111	-38	-13	1,105	16,759	-1.8	0.5
Talbot	MD	-489	226	-584	-131	-209	310	-489	-31	8,864	37,526	-1.6	-9.5
Newport News	VA	-2,441	-1,695	-1,056	310	-1,438	-1,311	-753	626	8,630	186,247	-1.4	-1.9

TABLE S8-B Apparent Emigration 1990–2020 from Land Below 3 meters by County, Ranked as a Percent of 1990 Population

County	State	Apparent Migration[1]				Δ Population below 3m [2]				County Population In 2020		Migration as % of county population[3]	
		1990 to 2020	1990s	2000s	2010s	1990 to 2020	1990s	2000s	2010s	<1m	Total	Total	Black
Dorchester	MD	-414	29	-169	-274	-159	123	-101	-181	6,911	32,531	-1.4	0.0
Jefferson Davis	LA	-418	-289	-164	36	-115	-182	-62	129	3,853	32,250	-1.4	-1.5
Lancaster	VA	-125	122	-161	-86	-63	138	-137	-64	1,387	10,919	-1.2	-0.4
Hancock	MS	-319	2,669	-3,209	221	69	2,704	-2,985	350	6,466	46,053	-1.0	3.4
Kent	MD	-179	63	-228	-13	-73	115	-202	14	1,825	19,198	-1.0	-2.4
Newport	RI	-776	-5	-671	-100	-567	68	-607	-28	3,731	85,643	-0.9	-2.3
Westmoreland	VA	-103	58	-235	74	69	114	-170	124	1,793	18,477	-0.7	-0.2
Burlington	NJ	-2,459	-503	1,231	-3,187	-1,540	-174	1,510	-2,876	17,133	461,860	-0.6	2.2
Bertie	NC	-119	-62	17	-75	-60	-35	32	-57	254	17,934	-0.6	0.0
Gloucester	NJ	-1,279	-1,164	50	-165	-755	-1,012	234	23	11,663	302,294	-0.6	1.0
Mobile	AL	-1,933	474	-2,480	72	-402	876	-1,823	546	16,229	414,809	-0.5	0.4
New London	CT	-1,234	-1,116	59	-177	-772	-893	169	-48	7,519	268,555	-0.5	-1.5
Camden	NJ	-2,383	-1,950	-28	-405	-801	-1,488	535	152	25,141	523,485	-0.5	-1.8
Cumberland	NJ	-641	-41	-501	-99	-290	18	-300	-8	7,759	154,152	-0.5	-1.4
Jefferson	TX	-1,054	-1,132	-3,534	3,612	971	-567	-3,077	4,615	78,343	256,526	-0.4	-3.8
St. Mary's	MD	-331	-573	85	157	-57	-464	166	241	5,768	113,777	-0.4	-2.1
Washington	ME	-144	-30	-58	-55	-104	-13	-44	-47	619	31,095	-0.4	10.3
Washington	RI	-424	282	-774	68	-173	364	-684	147	4,807	129,839	-0.4	0.7
Harford	MD	-654	-488	-192	26	-577	-465	-180	68	1,161	260,924	-0.4	-0.7
Onslow	NC	-483	-1,917	-158	1,592	-105	-1,789	-14	1,698	6,627	204,576	-0.3	-2.7
Caroline	MD	-80	-42	22	-60	-25	-20	42	-46	1,131	33,293	-0.3	-3.1
St. Martin	LA	-107	314	-100	-321	266	413	22	-169	3,402	51,767	-0.2	0.14
Orange	CA	-5,647	-3,935	-2,681	969	-3,628	-3,292	-1,975	1,639	107,666	3,186,989	-0.2	0.56
Mendocino	CA	-184	-110	-70	-4	-182	-109	-70	-2	29	91,601	-0.2	0.02
Acadia	LA	-127	-40	90	-178	312	105	203	5	1,778	57,576	-0.2	-0.32
Baltimore	MD	-1,256	-1,348	-680	771	-67	-886	-378	1,197	18,966	854,535	-0.2	0.09
Franklin	VA	-11	-5	5	-11	-10	-4	5	-11	0	8,180	-0.14	-0.16
Philadelphia	PA	-2,074	-4,166	2,344	-253	-36	-3,351	2,774	540	21,833	1,603,797	-0.13	-0.04
Del Norte	CA	-30	-72	37	6	-31	-73	36	6	115	27,743	-0.13	-0.05
Hertford	NC	-26	13	-38	-2	-24	14	-37	-2	116	21,552	-0.12	-0.13
New Haven	CT	-884	307	-307	-883	310	693	69	-452	27,518	864,835	-0.11	0.86

TABLE S8-B Apparent Emigration 1990–2020 from Land Below 3 meters by County, Ranked as a Percent of 1990 Population																
	State	Apparent Migration[1]					Δ Population below 3m [2]					County Population In 2020		Migration as % of county population[3]		
County		1990 to 2020	1990s	2000s	2010s		1990 to 2020	1990s	2000s	2010s		<1m	Total		Total	Black
King George	VA	-14	17	-6	-25		1	22	1	-22		140	26,723		-0.10	0.10
Greene	NY	-44	92	-131	-5		-24	98	-125	2		403	47,931		-0.10	-0.70
Kent	RI	-135	173	-501	192		70	228	-418	260		3,143	170,363		-0.08	2.69
Southampton	VA	-14	-12	-2	1		-7	-7	-2	2		81	17,996		-0.08	-0.13
Multnomah	OR	-435	76	-1	-510		-433	76	-1	-509		715	815,428		-0.07	0.00
Dutchess	NY	-188	158	318	-664		-178	158	327	-663		280	295,911		-0.07	0.04
Douglas	OR	-65	-399	248	86		-49	-390	249	92		1,308	111,201		-0.07	2.55
Hampton	SC	-11	-5	-5	0		-10	-5	-5	0		12	18,561		-0.06	-0.14
Hawaii	HI	-69	550	-665	46		4	590	-662	75		1,309	200,629		-0.06	-1.81
Knox	ME	-21	43	-80	16		11	55	-66	22		724	40,607		-0.06	9.84
Yolo	CA	-76	-86	-56	66		-59	-79	-54	74		607	216,403		-0.05	0.12
Jones	NC	-4	4	1	-9		0	5	2	-7		66	9,172		-0.05	-0.35
Clarke	AL	-12	-3	-6	-2		-11	-2	-6	-2		1	23,087		-0.04	-0.09
Santa Barbara	CA	-158	1,542	-1,787	88		-59	1,548	-1,726	120		2,278	448,229		-0.04	-0.07
Surry	VA	-3	6	-6	-3		-2	6	-6	-3		77	6,561		-0.04	0.05
Washington	AL	-7	4	-12	2		-4	2	-8	2		18	15,388		-0.04	-0.20
Liberty	TX	-21	27	-42	-6		1	32	-30	-1		102	91,628		-0.04	0.01
Lincoln	ME	-12	12	-56	31		8	19	-50	39		549	35,237		-0.04	27.6
San Luis Obispo	CA	-52	7	-85	26		-23	18	-72	31		373	282,424		-0.02	0.01
Newton	TX	-2	3	-1	-4		1	3	0	-2		5	12,217		-0.02	0.00
Bucks	PA	-84	-162	-117	196		89	-98	-52	238		1,624	646,538		-0.02	0.19
Rensselaer	NY	-21	-17	-10	6		8	-11	-1	21		107	161,130		-0.01	0.12
Waldo	ME	-4	1	-4	-1		0	2	-2	1		127	39,607		-0.01	0.22
Allen	LA	-2	1	-1	-3		-2	1	-1	-3		0	22,750		-0.01	-0.01
Martin	NC	-2	39	-10	-31		16	41	-5	-20		185	22,031		-0.01	-0.02
Clark	WA	-21	-13	-38	30		-21	-13	-38	30		36	503,311		-0.01	0.000
Kleberg	TX	-2	-22	-10	29		26	-11	-2	39		148	31,040		-0.01	0.000
Lane	OR	-23	-40	15	2		-15	-37	17	5		179	382,971		-0.01	-0.004
Jasper	TX	-2	5	-8	1		-1	5	-7	1		4	32,980		-0.01	0.000
Mercer	NJ	-23	-29	11	-6		-19	-26	12	-5		13	387,340		-0.01	0.000
Albany	NY	-19	-10	-7	-2		-15	-10	-4	-1		41	314,848		-0.01	-0.002

TABLE S8-B Apparent Emigration 1990–2020 from Land Below 3 meters by County, Ranked as a Percent of 1990 Population																
	State	Apparent Migration[1]					Δ Population below 3m [2]					County Population In 2020		Migration as % of county population[3]		
County		1990 to 2020	1990s	2000s	2010s		1990 to 2020	1990s	2000s	2010s		<1m	Total		Total	Black
Los Angeles	CA	-450	-3,378	1,507	1,421		519	-3,003	1,808	1,714		35,494	10,014,009		-0.01	-0.008
Fredericksburg	VA	-1	0	-1	0		-1	0	-1	0		0	27,982		-0.005	0.000
Refugio	TX	0	6	-12	5		3	7	-10	6		40	6,741		-0.004	0.160
Hanover	VA	-2	0	-2	0		-2	0	-2	0		4	109,979		-0.004	0.002
Washington	FL	-1	0	0	0		2	1	0	1		7	25,318		-0.004	-0.040
Liberty	FL	0	0	0	0		0	0	0	0		0	7,974		-0.002	0.000
Lenoir	NC	-1	-1	0	0		-1	-1	0	0		1	55,122		-0.002	0.000
Henrico	VA	-4	-2	1	-2		-4	-2	1	-2		6	334,389		-0.002	0.000
Total[4]		-225,584	-63,714	-301,171	-63,151		3,010,540	1,239,649	669,098	1101794		12,775,499	120,181,538			

1. Change in population of land below or less than three meters above the sea level of 2020. Includes migration into and out of this land as well as births and deaths. Negative numbers mean emigration out of this county's land below 3m. Calculations use building-based density assumption.
2. The difference between the population of land below or less than three meters above sea level at the end of the period and the population at the beginning of the period. Like apparent migration, it includes migration into and out of this land as well as births and deaths; but it also includes the population of the land that was more than 3m above sea level at the beginning of the period but less than 3m above sea level at the end of the period.
3. Equal to "Apparent Migration" for 1990 to 2020 as a percent of the population of 1990, with numerator and denominator using the same racial classification in both cases.
4. Sum of "Apparent Migration" includes only those counties with net emigration from <3m for the particular decade(s) of the column. The sum of Δ Population below 3m includes all counties.

The purpose of this table is to identify counties where the population vulnerable to sea level rise is declining.

Beg

TABLE S8-C Apparent Black Emigration 1990–2020 from Land Below 1 meter by County, Ranked as a Percent of 1990 Population

County	State	Apparent Migration[1]				Δ Population below 1m [2]				Apparent Migration All Races	County Black Population (2020)	Migration as % of county population[3]	
		1990 to 2020	1990s	2000s	2010s	1990 to 2020	1990s	2000s	2010s			Black	All Races
Hyde	NC	-775	-111	-335	-330	-659	-53	-295	-310	-1,117	1,152	-43.6	-20.6
Cameron	LA	-214	-68	-152	6	-158	-34	-132	9	-2,656	71	-42.9	-28.7
Tyrrell	NC	-539	88	62	-688	-467	104	88	-659	-560	934	-34.9	-14.5
Orleans	LA	-83,159	18,785	-107,584	5,640	-80,412	19,809	-106,458	6,238	-100,979	205,876	-27.3	-20.3
Plaquemines	LA	-931	345	-1,511	235	-816	408	-1,479	255	-2,040	4,863	-15.8	-8.0
Cape May	NJ	-797	-197	-404	-196	-621	-119	-339	-163	-2,819	3,305	-15.3	-3.0
Jefferson	TX	-7,519	-1,623	-3,893	-2,003	-6,057	-1,225	-3,397	-1,436	-6,393	83,856	-10.2	-2.7
Beaufort	NC	-980	-370	-335	-276	-692	-249	-236	-207	-1,136	10,195	-7.5	-2.7
Atlantic	NJ	-2,335	-605	-912	-818	186	336	-40	-110	-1,481	39,022	-6.2	-0.7
Carteret	NC	-206	-54	-49	-104	-116	-13	-28	-74	-253	3,208	-4.7	-0.5
St. Johns	FL	-286	-132	-118	-36	-168	-77	-76	-15	6,901	12,940	-3.9	8.2
San Mateo	CA	-1,324	-694	-245	-385	-1,082	-566	-177	-339	13,421	14,701	-3.9	2.1
Somerset	MD	-345	-27	-103	-215	-253	3	-66	-191	-1,028	9,449	-3.9	-4.4
St. Mary	LA	-677	-554	628	-752	864	-2	1,012	-146	-2,713	14,950	-3.7	-4.7
Pamlico	NC	-91	-11	-64	-16	-17	18	-33	-2	-408	2,055	-3.1	-3.6
Marin	CA	-221	-123	-100	2	-175	-93	-89	6	3,614	6,120	-2.9	1.6
Brazoria	TX	-419	48	-252	-216	-377	47	-222	-202	-91	53,668	-2.7	0.0
Currituck	NC	-41	-33	-18	11	-12	-18	-10	16	868	1,377	-2.6	6.3
Charleston	SC	-2,339	-849	-637	-853	-1,187	-406	-249	-532	6,757	91,746	-2.3	2.3
Citrus	FL	-49	-20	-8	-22	-23	-9	1	-15	-518	3,891	-2.3	-0.6
Washington	NC	-105	-34	-8	-62	-73	-22	3	-54	-127	5,350	-1.6	-0.9
Camden	NC	-24	-13	7	-18	-2	-6	14	-10	182	1,049	-1.6	3.1
Beaufort	SC	-399	-22	3	-380	-251	25	59	-335	4,370	27,545	-1.6	5.1
Jefferson Davis	LA	-79	-58	-12	-9	-23	-24	0	1	-82	5,130	-1.4	-0.3
Pasquotank	NC	-137	65	-118	-84	105	131	-12	-14	-250	14,316	-1.2	-0.8
Harford	MD	-167	-79	-88	0	-154	-67	-88	1	-649	36,837	-1.1	-0.4
Dorchester	MD	-90	-27	-27	-36	-68	-20	-20	-29	-642	9,017	-1.1	-2.1

TABLE S8-C Apparent Black Emigration 1990–2020 from Land Below 1 meter by County, Ranked as a Percent of 1990 Population

County	State	Apparent Migration[1]				Δ Population below 1m [2]				Apparent Migration All Races	County Black Population (2020)	Migration as % of county population[3]	
		1990 to 2020	1990s	2000s	2010s	1990 to 2020	1990s	2000s	2010s			Black	All Races
McIntosh	GA	-38	-21	57	-73	-30	-16	57	-70	58	3,176	-1.0	0.7
Aransas	TX	-3	-3	1	0	0	-1	1	0	-35	241	-1.0	-0.2
Talbot	MD	-51	-15	-22	-14	-39	-10	-17	-12	-278	4,186	-0.9	-0.9
Livingston	LA	-34	-26	11	-19	-18	-17	14	-14	858	11,178	-0.9	1.2
Franklin	FL	-10	-10	-6	7	-4	-9	-2	7	99	1,403	-0.9	1.1
Georgetown	SC	-169	-23	-84	-62	-118	-5	-66	-47	728	18,051	-0.8	1.6
San Patricio	TX	-6	-3	0	-2	-3	-1	0	-1	9	994	-0.7	0.0
Perquimans	NC	-19	-10	-3	-7	-9	-5	0	-4	55	2,686	-0.6	0.5
Camden	GA	-32	-7	-7	-18	-23	-6	-3	-13	229	9,497	-0.5	0.8
Fairfield	CT	-369	-30	-308	-31	-144	36	-212	32	1,439	99,992	-0.5	0.2
Suffolk	MA	-651	-792	-185	326	-277	-617	-109	448	14,914	135,255	-0.5	2.2
Gloucester	VA	-13	-9	-7	3	0	-3	-4	7	-416	2,706	-0.4	-1.4
Dare	NC	-3	-7	5	0	38	8	15	16	1,060	678	-0.4	4.7
Humboldt	CA	-3	-4	0	1	2	0	1	1	-17	1,729	-0.3	0.0
San Juan	WA	0	0	0	0	0	0	0	0	4	48	-0.3	0.0
Newport News	VA	-174	-80	-35	-59	-88	-41	-8	-39	-333	76,870	-0.3	-0.2
Queen Anne's	MD	-11	7	-7	-11	-1	8	-3	-6	433	2,775	-0.3	1.3
Caroline	MD	-12	-4	-2	-6	-11	-4	-1	-5	-34	4,368	-0.3	-0.1
Newport	RI	-8	3	-11	0	-7	3	-9	0	-121	2,645	-0.3	-0.1
Chesapeake	VA	-91	-97	-43	49	394	27	159	209	1,265	70,885	-0.2	0.8
Worcester	MD	-16	46	-19	-43	38	56	2	-20	1,300	6,166	-0.2	3.7
Northumberland	VA	-7	0	0	-6	-3	2	0	-5	-78	2,673	-0.2	-0.7
Portsmouth	VA	-98	-27	-183	111	183	69	-52	166	-374	51,586	-0.2	-0.4
Kent	MD	-7	4	-12	2	-4	4	-10	2	-28	2,751	-0.2	-0.2
Onslow	NC	-55	-27	-27	-1	-26	-13	-16	3	311	26,939	-0.2	0.2
Santa Rosa	FL	-6	-15	1	8	1	-10	2	9	342	10,690	-0.2	0.4
Northampton	VA	-10	2	-17	5	-2	4	-12	7	-16	3,756	-0.2	-0.1
Greene	NY	-3	8	-8	-3	-1	8	-7	-3	-3	2,159	-0.2	0.0
Santa Clara	CA	-82	139	-171	-49	-61	151	-165	-46	1,951	42,148	-0.2	0.1
Solano	CA	-53	-75	10	12	-41	-67	9	17	-397	60,051	-0.12	-0.12
Middlesex	CT	-7	-5	-1	-1	-6	-5	0	-1	145	8,001	-0.12	0.10

TABLE S8-C Apparent Black Emigration 1990–2020 from Land Below 1 meter by County, Ranked as a Percent of 1990 Population

County	State	Apparent Migration[1]				Δ Population below 1m [2]				Apparent Migration All Races	County Black Population (2020)	Migration as % of county population[3]	
		1990 to 2020	1990s	2000s	2010s	1990 to 2020	1990s	2000s	2010s			Black	All Races
Craven	NC	-24	-1	-11	-12	-9	4	-8	-5	-34	19,903	-0.11	-0.04
Westmoreland	VA	-5	1	-5	-1	-1	1	-3	1	-44	4,470	-0.10	-0.29
New London	CT	-11	-13	-1	3	-9	-12	-1	3	-295	14,422	-0.10	-0.12
King and Queen	VA	-2	0	-1	-1	-1	0	0	-1	4	1,561	-0.09	0.06
Anne Arundel	MD	-38	-9	-28	-1	5	2	-7	10	-187	102,555	-0.08	-0.04
Philadelphia	PA	-470	-296	-19	-156	-118	-141	94	-71	-1,191	613,835	-0.08	-0.08
Charles City	VA	-3	0	-2	-1	-3	0	-2	-1	-3	2,836	-0.07	-0.04
Liberty	GA	-14	8	-22	0	-11	10	-21	1	166	27,309	-0.07	0.32
Westchester	NY	-77	-59	-58	40	-34	-23	-53	41	19	131,010	-0.07	0.00
King George	VA	-2	1	2	-5	-1	1	2	-4	-33	3,919	-0.06	-0.25
Knox	ME	0	0	0	0	0	0	0	0	-54	256	-0.05	-0.15
Isle of Wight	VA	-3	1	0	-5	-3	0	1	-4	-18	8,579	-0.04	-0.07
Bertie	NC	-5	1	-8	2	-2	2	-6	3	-6	10,674	-0.04	-0.03
Napa	CA	0	0	1	-1	0	0	1	-1	-45	2,300	-0.04	-0.04
Jefferson	FL	-2	-2	0	0	-1	-1	0	0	1	4,600	-0.03	0.01
Berkeley	SC	-9	3	-19	7	11	13	-19	18	903	51,784	-0.03	0.70
Iberville	LA	-4	0	-2	-2	1	2	0	-1	-40	13,313	-0.03	-0.13
Jackson	TX	0	0	0	0	0	0	0	0	-1	937	-0.03	-0.01
Middlesex	VA	-1	1	-2	1	0	1	-2	1	-16	1,519	-0.03	-0.19
Washington	AL	-1	-1	0	0	-1	-1	0	0	-1	3,318	-0.02	-0.01
Washington	FL	0	0	0	0	0	0	0	0	0	3,236	-0.02	0.00
Multnomah	OR	-5	-4	4	-6	-5	-4	4	-6	-464	43,793	-0.02	-0.08
Hertford	NC	-2	19	-22	0	0	19	-20	1	-17	12,215	-0.014	-0.08
Virginia Beach	VA	-7	-8	34	-33	38	4	48	-14	310	82,583	-0.013	0.08
Escambia	FL	-7	6	-13	1	-4	6	-12	2	-144	68,148	-0.013	-0.05
Union	NJ	-11	-20	10	-1	5	-16	16	5	-44	112,261	-0.012	-0.01
San Francisco	CA	-9	-13	1	3	-7	-11	1	3	119	45,071	-0.012	0.02
DeSoto	FL	0	0	-1	0	0	0	0	0	26	4,203	-0.011	0.11
Orange	NY	-2	0	-1	-1	-2	0	-1	-1	-29	41,341	-0.009	-0.01
Gloucester	NJ	-2	10	-32	21	41	25	-12	28	-238	31,517	-0.009	-0.10
Santa Cruz	CA	0	0	0	0	0	0	0	0	11	2,850	-0.004	0.01

TABLE S8-C Apparent Black Emigration 1990–2020 from Land Below 1 meter by County, Ranked as a Percent of 1990 Population

County	State	Apparent Migration[1]				Δ Population below 1m [2]				Apparent Migration All Races	County Black Population (2020)	Migration as % of county population[3]	
		1990 to 2020	1990s	2000s	2010s	1990 to 2020	1990s	2000s	2010s			Black	All Races
Petersburg	VA	0	0	0	0	0	0	0	0	0	24,530	-0.002	-0.001
Essex	NJ	-5	-41	-24	60	30	-27	-16	74	173	324,081	-0.002	0.022
Whatcom	WA	0	0	0	0	0	0	0	0	0	2,221	-0.001	0.000
Rensselaer	NY	0	0	-1	0	0	0	0	0	-3	11,800	-0.001	-0.002
King	WA	-1	3	4	-7	-1	3	4	-7	-37	147,831	-0.001	-0.002
Los Angeles	CA	-6	53	-57	-2	-4	52	-55	-1	-317	760,689	-0.001	-0.004
Thurston	WA	0	0	0	0	0	0	0	0	-32	8,893	-0.001	-0.020
Prince George's	MD	-2	1	2	-5	-2	1	2	-5	-8	571,866	0.000	-0.001
Total[4]	US	-107,025	-8,091	-121,451	-12,035	381,764	169,396	-50,889	263,257	-169,312	15,826,130		
Total, Excluding Displacement[5]	US	-98,354	-3,829	-117,097	-6,590								

1. Change in Black population of land below or less than one meter above the sea level of 2020. Includes migration into and out of this land as well as births and deaths. Negative numbers mean emigration out of this county's land below 1m. All calculations use the building-based density assumption.
2. The difference between the (Black) population of land below or less than one meter above sea level at the end of the period and the population at the beginning of the period. Like apparent migration, it includes migration into and out of this land as well as births and deaths; but it also includes the population of the land that was more than 1m above sea level at the beginning of the period but less than 1m above sea level at the end of the period. All calculations use the building-based density assumption.
3. Equal to "Apparent Migration" for 1990 to 2020 as a percent of the population of 1990, with numerator and denominator using the same racial classification in both cases.
4. Sum of emigration from counties with net Black emigration from land <1m.
5. Sum of emigration of Black residents or total Emigration by county, whichever is less. (If Black emigration is greater than total emigration, the difference represents displacement of Black people by people from other racial groups.)

The purpose of this table is to identify counties where the population of Black residents vulnerable to sea level rise is declining.

TABLE S8-D Apparent Black Emigration 1990–2020 from Land Below 3 meters by County, Ranked as a Percent of 1990 Population

County	State	Apparent Migration[1]				Δ Population below 3m [2]				Apparent Migration All Races	County Black Population (2020)	Migration as % of county population[3]	
		1990 to 2020	1990s	2000s	2010s	1990 to 2020	1990s	2000s	2010s			Black	All Races
Cameron	LA	-402	-113	-250	-39	-400	-114	-249	-38	-3,739	71	-80.7	-40.4
Tyrrell	NC	-623	67	64	-754	-623	67	63	-753	-641	934	-40.4	-16.6
Hyde	NC	-640	226	-213	-653	-640	226	-213	-653	-869	1,152	-36.0	-16.1
Pamlico	NC	-966	-18	-544	-403	-931	-16	-522	-392	-363	2,055	-32.9	-3.2
Mathews	VA	-373	-134	-167	-72	-330	-113	-154	-63	-271	658	-32.1	-3.2
Orleans	LA	-94,923	16,203	-114,145	3,019	-94,840	16,226	-114,102	3,036	-110,016	205,876	-31.1	-22.1
Camden	NC	-376	-208	32	-199	-346	-196	37	-187	2,848	1,049	-25.4	48.2
Cape May	NJ	-1,266	-181	-557	-528	-1,225	-171	-543	-511	-6,337	3,305	-24.2	-6.7
Dare	NC	-193	-43	-10	-140	-180	-40	-4	-136	8,714	678	-23.9	38.3
Carteret	NC	-847	-225	-84	-538	-793	-208	-60	-524	2,357	3,208	-19.4	4.5
Galveston	TX	-6,076	-1,143	-4,059	-874	-4,546	-691	-3,485	-370	4,343	43,120	-16.2	2.0
Charleston	SC	-16,373	-3,197	-4,242	-8,934	-14,077	-2,313	-3,464	-8,300	58,071	91,746	-16.0	19.7
San Mateo	CA	-5,329	-2,629	-1,557	-1,144	-5,117	-2,506	-1,506	-1,104	34,598	14,701	-15.7	5.3
Plaquemines	LA	-919	409	-1,522	194	-918	408	-1,520	194	-1,928	4,863	-15.6	-7.5
Beaufort	NC	-1,882	-389	-729	-763	-1,671	-328	-647	-695	-1,764	10,195	-14.3	-4.2
St. Mary	LA	-2,389	-866	681	-2,204	-1,862	-647	839	-2,054	-7,373	14,950	-13.1	-12.7
St. Johns	FL	-945	-246	-301	-397	-903	-236	-286	-382	29,562	12,940	-13.0	35.3
Atlantic	NJ	-4,496	-943	-1,966	-1,587	-4,457	-954	-1,939	-1,563	-5,393	39,022	-11.9	-2.4
Somerset	MD	-1,009	107	-565	-551	-906	73	-453	-526	-1,346	9,449	-11.4	-5.7
Citrus	FL	-233	-18	-148	-68	-227	-18	-144	-65	1,454	3,891	-10.8	1.6
Gulf	FL	-221	-115	-48	-57	-165	-84	-35	-46	-273	1,692	-10.3	-2.4
Talbot	MD	-521	-211	-193	-116	-466	-181	-177	-107	-489	4,186	-9.5	-1.6
Washington	NC	-571	-94	-153	-324	-437	-62	-101	-274	-1,002	5,350	-9.0	-7.2
Northampton	VA	-542	-238	-230	-74	-515	-229	-218	-68	-336	3,756	-9.0	-2.6
Vermilion	LA	-578	-102	-138	-337	361	275	158	-72	-2,444	7,859	-8.3	-4.9
Franklin	FL	-59	-41	-21	4	-57	-43	-20	6	739	1,403	-5.3	8.2
Orange	TX	-340	-99	-837	595	176	22	-633	787	-1,620	7,981	-5.0	-2.0
King William	VA	-165	-99	-3	-62	-147	-93	1	-55	187	2,585	-5.0	1.7
Currituck	NC	-77	-139	35	27	-22	-115	40	54	9,510	1,377	-5.0	69.2

TABLE S8-D Apparent Black Emigration 1990–2020 from Land Below 3 meters by County, Ranked as a Percent of 1990 Population													
	State	Apparent Migration[1]				Δ Population below 3m [2]				Apparent Migration All Races	County Black Population (2020)	Migration as % of county population[3]	
County		1990 to 2020	1990s	2000s	2010s	1990 to 2020	1990s	2000s	2010s			Black	All Races
Marin	CA	-353	-159	-315	120	-233	-125	-237	128	9,808	6,120	-4.7	4.3
Craven	NC	-954	-116	6	-844	-656	10	94	-759	336	19,903	-4.6	0.4
Accomack	VA	-455	-135	-263	-57	-395	-104	-247	-45	-657	8,639	-4.2	-2.1
Jefferson	TX	-2,807	421	-3,131	-97	-2,734	392	-3,207	80	-1,054	83,856	-3.8	-0.4
San Patricio	TX	-28	10	-45	7	-24	9	-41	8	201	994	-3.2	0.3
Perquimans	NC	-108	-106	78	-80	24	-57	115	-34	1,766	2,686	-3.2	16.9
Fairfield	CT	-2,418	-1,085	-636	-697	-2,158	-987	-562	-609	2,780	99,992	-3.1	0.3
Caroline	MD	-136	-91	-21	-24	-126	-85	-19	-22	-80	4,368	-3.1	-0.3
Gloucester	VA	-96	-25	-79	8	-86	-22	-76	11	193	2,706	-2.9	0.6
Onslow	NC	-778	-512	-222	-44	-706	-478	-197	-32	-483	26,939	-2.7	-0.3
Norfolk	VA	-2,673	4,147	-274	-6,545	4,069	5,931	2,299	-4,161	2,471	93,553	-2.6	0.9
Worcester	MD	-195	106	-237	-65	-99	145	-211	-33	11,321	6,166	-2.6	32.3
Hendry	FL	-104	18	-124	2	-68	43	-114	3	904	4,195	-2.5	3.5
Harrison	MS	-788	-244	-911	367	-121	12	-663	530	-2,921	51,143	-2.5	-1.8
Kent	MD	-83	-14	-80	12	-61	1	-76	14	-179	2,751	-2.4	-1.0
Newport	RI	-77	87	-134	-29	-68	90	-134	-24	-776	2,645	-2.3	-0.9
Iberville	LA	-321	110	39	-470	-158	70	136	-364	-649	13,313	-2.2	-2.1
San Juan	WA	0	0	0	-1	0	0	0	-1	80	48	-2.1	0.8
St. Mary's	MD	-212	-159	-39	-14	-191	-148	-35	-9	-331	15,994	-2.1	-0.4
Newport News	VA	-1,060	-585	-191	-284	-439	-377	-4	-57	-2,441	76,870	-1.9	-1.4
Camden	NJ	-1,432	-378	-197	-857	-946	-311	16	-651	-2,383	95,135	-1.8	-0.5
Hawaii	HI	-10	-13	0	2	-10	-12	0	2	-69	1,159	-1.8	-0.1
Livingston	LA	-65	-14	2	-54	-37	-6	13	-43	2,634	11,178	-1.7	3.7
Brevard	FL	-488	-56	-482	50	-402	-37	-442	77	13,613	56,498	-1.6	3.4
New London	CT	-179	-158	-17	-4	-125	-123	-8	5	-1,234	14,422	-1.5	-0.5
Jefferson Davis	LA	-88	-83	-9	4	-63	-76	2	12	-418	5,130	-1.5	-1.4
New York	NY	-3,759	-3,835	-2,243	2,320	-1,338	-2,763	-1,402	2,828	55,155	199,592	-1.4	3.7
McIntosh	GA	-53	71	162	-286	-17	73	166	-256	734	3,176	-1.4	8.5
Cumberland	NJ	-310	132	-547	105	-203	130	-454	121	-641	26,375	-1.4	-0.5

TABLE S8-D Apparent Black Emigration 1990–2020 from Land Below 3 meters by County, Ranked as a Percent of 1990 Population														
	State	Apparent Migration[1]				Δ Population below 3m [2]				Apparent Migration All Races	County Black Population (2020)	Migration as % of county population[3]	
County		1990 to 2020	1990s	2000s	2010s	1990 to 2020	1990s	2000s	2010s			Black	All Races
Willacy	TX	-1	-2	0	1	-1	-2	0	1	30	445	-1.4	0.2
Beaufort	SC	-320	1,449	31	-1,800	-86	1,499	109	-1,694	27,664	27,545	-1.3	32.0
Rockingham	NH	-29	-18	-8	-3	-27	-17	-8	-2	1,736	2,076	-1.3	0.7
Wicomico	MD	-161	-144	-84	67	-129	-130	-73	75	384	27,586	-1.0	0.5
Matagorda	TX	-43	16	-36	-23	-12	27	-25	-15	184	3,734	-0.9	0.5
Georgetown	SC	-172	85	105	-362	71	147	194	-271	3,977	18,051	-0.9	8.6
Santa Cruz	CA	-20	1	-34	13	-19	1	-33	13	129	2,850	-0.9	0.1
St. James	LA	-86	145	549	-780	499	250	781	-532	800	9,486	-0.8	3.8
Alexandria	VA	-186	-64	-47	-75	-109	-22	-23	-64	615	31,314	-0.8	0.6
Harford	MD	-110	-92	-37	19	-102	-95	-37	31	-654	36,837	-0.7	-0.4
Greene	NY	-14	27	-33	-8	-12	28	-32	-8	-44	2,159	-0.7	-0.1
New Kent	VA	-15	4	15	-33	-13	2	15	-31	461	2,714	-0.7	4.4
Middlesex	CT	-39	-34	1	-6	-35	-32	2	-5	207	8,001	-0.7	0.1
Chambers	TX	-17	-1	-29	13	16	10	-18	23	215	3,148	-0.7	1.1
Ventura	CA	-91	178	-285	16	-61	182	-271	28	60	13,704	-0.6	0.0
Anne Arundel	MD	-306	-9	-65	-232	-255	11	-51	-215	1,691	102,555	-0.6	0.4
Colleton	SC	-93	35	-154	26	-58	47	-137	32	829	13,401	-0.6	2.4
Honolulu	HI	-144	-360	-158	375	-95	-343	-135	383	33,320	19,356	-0.6	4.0
King and Queen	VA	-14	6	-17	-3	-6	10	-16	0	60	1,561	-0.5	1.0
Queen Anne's	MD	-20	16	6	-42	4	26	13	-35	3,062	2,775	-0.5	9.0
Brazoria	TX	-80	176	31	-287	119	241	85	-206	2,645	53,668	-0.5	1.4
Northumberland	VA	-15	-25	-32	42	14	-14	-22	50	232	2,673	-0.5	2.2
Aransas	TX	-1	6	-27	20	13	8	-17	23	562	241	-0.4	3.1
Island	WA	-6	-4	-10	8	-5	-3	-10	8	330	2,265	-0.4	0.5
Lancaster	VA	-12	-22	-2	12	-3	-21	2	15	-125	2,946	-0.4	-1.2
Jones	NC	-13	-1	-4	-8	-12	0	-4	-8	-4	2,564	-0.4	0.0
Bergen	NJ	-134	438	-828	256	-2	446	-753	305	14,834	49,909	-0.4	1.8
Escambia	FL	-166	-170	49	-44	-44	-110	78	-12	4,972	68,148	-0.3	1.9
Acadia	LA	-32	-17	-23	8	-12	-7	-19	14	-127	9,946	-0.3	-0.2

TABLE S8-D Apparent Black Emigration 1990–2020 from Land Below 3 meters by County, Ranked as a Percent of 1990 Population

County	State	Apparent Migration[1]				Δ Population below 3m [2]				Apparent Migration All Races	County Black Population (2020)	Migration as % of county population[3]	
		1990 to 2020	1990s	2000s	2010s	1990 to 2020	1990s	2000s	2010s			Black	All Races
Nueces	TX	-26	-6	-62	42	15	9	-43	49	8,812	12,651	-0.2	3.0
Westmoreland	VA	-11	16	-54	27	8	22	-47	33	-103	4,470	-0.2	-0.7
Suffolk	MA	-285	-1,713	1,514	-86	-1	-1,646	1,616	28	64,463	135,255	-0.2	9.7
Monmouth	NJ	-94	78	-19	-153	122	145	83	-105	151	39,178	-0.2	0.0
Washington	AL	-9	-1	-8	0	-7	0	-7	0	-7	3,318	-0.2	0.0
Franklin	VA	-7	-3	6	-10	-6	-2	6	-10	-11	4,610	-0.2	-0.1
Assumption	LA	-11	-19	62	-55	135	45	97	-7	105	5,973	-0.2	0.5
Hampton	SC	-14	-6	-6	-2	-14	-6	-6	-2	-11	9,536	-0.1	-0.1
Hertford	NC	-17	29	-38	-7	-17	28	-38	-7	-26	12,215	-0.1	-0.1
Southampton	VA	-10	-7	3	-7	-8	-5	3	-6	-14	5,908	-0.1	-0.1
Clarke	AL	-10	-3	-4	-2	-9	-2	-4	-2	-12	10,223	-0.1	0.0
Jefferson	FL	-4	-4	0	0	-4	-4	0	0	3	4,600	-0.1	0.0
Chowan	NC	-4	29	-34	1	37	41	-24	20	226	4,376	-0.1	1.7
Santa Barbara	CA	-6	25	-42	11	-6	24	-41	11	-158	6,467	-0.1	0.0
DeSoto	FL	-2	0	-3	0	-2	0	-3	0	202	4,203	-0.1	0.8
Del Norte	CA	0	-1	0	0	0	-1	0	0	-30	841	-0.1	-0.1
Westchester	NY	-52	-647	97	497	15	-598	106	507	2,065	131,010	0.0	0.2
Washington	FL	-1	-1	0	0	2	1	0	1	-1	3,236	0.0	0.0
Philadelphia	PA	-230	393	-20	-603	-81	397	37	-516	-2,074	613,835	0.0	-0.1
Fairfax	VA	-19	-23	-22	26	-9	-20	-18	29	159	108,339	0.0	0.0
Bertie	NC	-3	29	-27	-5	10	32	-22	0	-119	10,674	0.0	-0.6
Martin	NC	-2	47	-24	-25	8	46	-20	-17	-2	8,868	0.0	0.0
Monterey	CA	-3	2	3	-8	-3	2	2	-7	363	9,051	0.0	0.1
Prince George's	MD	-43	30	-50	-24	-28	34	-44	-18	18	571,866	0.0	0.0
Allen	LA	0	0	0	0	0	0	0	0	-2	4,016	0.0	0.0
Los Angeles	CA	-75	-316	65	176	16	-269	83	203	-450	760,689	0.0	0.0
Washington	DC	-32	25	302	-359	210	84	401	-275	1,139	282,066	0.0	0.2
Total[4]	US	-166,675	-25,372	-149,369	-83,766	461,111	328,602	76,446	56,063	-225,584	15,826,130		

TABLE S8-D   Apparent Black Emigration 1990–2020 from Land Below 3 meters by County, Ranked as a Percent of 1990 Population

	State	Apparent Migration[1]					Δ Population below 3m [2]				Apparent Migration All Races		County Black Population (2020)	Migration as % of county population[3]		
County		1990 to 2020	1990s	2000s	2010s		1990 to 2020	1990s	2000s	2010s					Black	All Races
Total, Excluding Displacement[5]	US	-118,795	-9,065	-133,632	-21,802											

1. Change in Black population of land below or less than 3m above the sea level of 2020. Includes migration into and out of this land as well as births and deaths. Negative numbers mean emigration out of this county's land below 3m. All calculations use the building-based density assumption.
2. The difference between the (Black) population of land below or less than three meters above sea level at the end of the period and the population at the beginning of the period. Like apparent migration, it includes migration into and out of this land as well as births and deaths; but it also includes the population of the land that was more than 3m above sea level at the beginning of the period but less than 3m above sea level at the end of the period. All calculations use the building-based density assumption.
3. Equal to "Apparent Migration" for 1990 to 2020 as a percent of the population of 1990, with numerator and denominator using the same racial classification in both cases,
4. Sum of emigration from counties with net Black emigration from land <3m.
5. Sum of Black emigration or total Emigration by county, whichever is less. (If Black emigration is greater than total emigration, the difference represents displacement of Black residents by people from other racial groups.

The purpose of this table is to identify counties where the population of Black residents vulnerable to sea level rise is declining.

3

beg

TABLE S8-E Apparent Emigration 1990–2020 from Inland 100-Year Floodplain by County, Ranked[1] as a Percent of 1990 Population

	State	Apparent Migration All Races[2]				Apparent Migration, Black[2]				Population in 2020, Riverine A Zone[3]		Migration as % of county population[4]	
County		1990 to 2020	1990s	2000s	2010s	1990 to 2020	1990s	2000s	2010s	All Races	Black	All Races	Black
Quitman	MS	-2,367	-573	-973	-821	-1,518	-318	-684	-517	2,489	1,862	-22.6	-24.5
Alexander	IL	-2,392	-671	-525	-1,197	-589	-181	-42	-366	2,107	614	-22.5	-16.9
Sharkey	MS	-1,440	-317	-774	-349	-847	-158	-492	-196	1,189	789	-20.4	-18.2
Brooks	TX	-1,194	-499	-415	-281	5	2	5	-2	3,729	6	-14.6	254.7
Mineral	NV	-942	-691	-149	-101	-106	-71	-40	6	1,841	88	-14.5	-31.0
Lander	NV	-868	-592	-169	-108	4	0	0	4	1,724	5	-13.9	44.4
Issaquena	MS	-224	-33	-365	173	-50	48	-203	105	683	349	-11.7	-4.7
Edwards	KS	-430	-219	-219	7	4	3	-5	6	957	6	-11.3	101.1
McDowell	WV	-2,915	-1,334	-866	-716	-836	-433	-230	-173	2,998	407	-8.3	-17.7
Tallahatchie	MS	-1,236	-272	-101	-863	-950	-222	-269	-459	1,750	1,282	-8.1	-10.8
Gallatin	IL	-505	-131	-218	-155	-2	-2	3	-3	485	0	-7.3	-4.8
Jackson	AR	-1,166	-569	-248	-349	-121	-65	-31	-26	2,203	222	-6.2	-4.4
Madison	LA	-742	214	-605	-352	-423	123	-393	-153	1,519	997	-6.0	-5.7
Washington	MS	-3,996	-504	-2,277	-1,215	564	1,790	-353	-873	10,319	7,501	-5.9	1.4
Wetzel	WV	-1,127	-302	-326	-499	6	3	-4	7	2,599	7	-5.9	34.8
Walsh	ND	-786	-302	-365	-119	31	3	3	25	3,778	36	-5.7	182.9
Pembina	ND	-520	-94	-271	-155	4	1	3	0	1,165	4	-5.6	27.7
Jackson	NC	-1,491	-2,173	460	222	-111	-117	-9	15	2,416	47	-5.6	-26.2
Phillips	AR	-1,598	-189	-572	-838	-795	35	-209	-621	2,276	1,595	-5.5	-5.1
Humphreys	MS	-654	-260	-192	-202	-420	-155	-131	-133	580	376	-5.4	-5.1
Logan	WV	-2,225	-1,181	-201	-844	-209	-72	-72	-65	6,336	140	-5.2	-15.4
Harmon	OK	-193	-122	-23	-48	-43	-49	-5	10	257	25	-5.1	-15.4
Clay	AR	-902	-220	-318	-364	7	18	-2	-8	1,722	9	-5.0	148.5
Kittson	MN	-281	-150	-97	-33	0	0	0	0	287	0	-4.9	*
Clay	WV	-477	-97	-156	-223	3	2	-3	3	1,137	3	-4.8	309.7

TABLE S8-E Apparent Emigration 1990–2020 from Inland 100-Year Floodplain by County, Ranked[1] as a Percent of 1990 Population

	State	Apparent Migration All Races[2]				Apparent Migration, Black[2]				Population in 2020, Riverine A Zone[3]		Migration as % of county population[4]	
County		1990 to 2020	1990s	2000s	2010s	1990 to 2020	1990s	2000s	2010s	All Races	Black	All Races	Black
Liberty	GA	-2,507	-1,301	-699	-507	-1,076	-297	-736	-43	4,371	1,479	-4.8	-5.3
Arkansas	AR	-1,008	-11	-577	-419	-299	108	-236	-171	2,446	897	-4.7	-6.3
Desha	AR	-777	-177	-346	-254	-117	114	-142	-89	1,851	911	-4.6	-1.6
Harlan	KY	-1,666	-354	-810	-502	-22	-39	-13	30	5,094	103	-4.6	-1.8
Cameron	PA	-266	-1	-174	-91	3	0	0	3	756	3	-4.5	44.4
Boone	WV	-1,083	-414	-224	-445	-41	-26	-11	-4	5,282	29	-4.2	-19.0
Mingo	WV	-1,372	-1,402	265	-236	-34	-23	-7	-3	4,097	40	-4.1	-4.1
Wyoming	WV	-1,143	-590	-350	-203	-22	-10	-11	0	2,556	10	-3.9	-9.2
Leflore	MS	-1,403	-163	-587	-653	-522	64	-194	-392	3,519	2,257	-3.8	-2.3
Buchanan	VA	-1,150	-614	-95	-441	11	1	20	-10	2,220	11	-3.7	20.0
Martin	KY	-452	15	-181	-286	75	1	74	0	2,269	77	-3.6	940.5
Boyd	NE	-101	-54	-23	-25	0	0	0	0	163	0	-3.6	*
Ransom	ND	-210	22	-96	-136	0	1	-1	0	436	1	-3.6	9.3
Ohio	WV	-1,795	-694	-1,174	73	201	113	61	27	5,955	348	-3.5	11.9
Stanley	SD	-85	15	-57	-43	6	1	-1	5	414	6	-3.5	566.8
Bolivar	MS	-1,443	-571	-729	-143	-1,128	-346	-663	-119	3,854	2,909	-3.4	-4.3
Henderson	IL	-278	-60	-176	-42	0	0	2	-2	145	0	-3.4	0.0
Geary	KS	-1,038	-635	102	-506	-309	-259	56	-106	781	129	-3.4	-4.4
Calhoun	WV	-265	-101	26	-189	0	1	2	-3	704	0	-3.4	-11.4
Calhoun	FL	-370	-268	-64	-38	-428	-253	-79	-96	836	252	-3.4	-25.9
White Pine	NV	-307	-187	42	-162	-17	-20	3	0	818	4	-3.3	-9.4
Norman	MN	-264	-112	-92	-59	2	0	1	1	708	2	-3.3	50.2
Lawrence	IL	-521	-131	-249	-141	-4	-8	-4	7	1,129	12	-3.3	-3.0
Caldwell	LA	-309	6	-69	-247	-158	-75	-34	-49	1,901	117	-3.2	-9.0
Gallia	OH	-970	-494	-305	-171	-31	-19	-15	3	2,131	59	-3.1	-3.6
Bell	KY	-960	-287	-219	-454	-35	-37	-30	32	2,958	133	-3.0	-4.4
White	IL	-490	-172	-113	-204	-3	-3	2	-2	587	4	-3.0	-7.9
Hale	TX	-1,011	-236	-395	-380	-324	-118	-129	-78	2,241	169	-2.9	-18.3

TABLE S8-E Apparent Emigration 1990–2020 from Inland 100-Year Floodplain by County, Ranked[1] as a Percent of 1990 Population

	State	Apparent Migration All Races[2]				Apparent Migration, Black[2]				Population in 2020, Riverine A Zone[3]		Migration as % of county population[4]	
County		1990 to 2020	1990s	2000s	2010s	1990 to 2020	1990s	2000s	2010s	All Races	Black	All Races	Black
Lenoir	NC	-1,668	-1,589	-74	-5	-1,071	-1,107	-53	89	1,984	998	-2.9	-4.8
Duval	TX	-373	-50	-134	-189	0	0	0	0	844	0	-2.9	0.0
Pendleton	WV	-233	-74	-50	-108	-6	-2	-2	-2	706	9	-2.9	-3.6
Yazoo	MS	-728	1,509	-1,651	-587	-143	562	-489	-216	1,529	956	-2.9	-1.1
Barnes	ND	-353	40	-307	-86	21	2	-4	23	1,297	30	-2.8	79.4
Dougherty	GA	-2,686	-1,389	-104	-1,192	-994	-669	243	-568	9,349	7,201	-2.8	-2.1
Calhoun	IL	-148	-120	14	-42	0	0	3	-3	285	0	-2.8	-11.3
Daviess	MO	-218	-215	-26	24	0	0	0	0	96	0	-2.8	0.0
Holmes	MS	-599	-217	-250	-132	-419	-153	-146	-120	952	827	-2.8	-2.6
Leavenworth	KS	-1,727	-1,700	-54	27	-669	-664	-28	23	639	43	-2.7	-9.7
Eureka	NV	-41	-42	26	-26	0	0	0	0	111	0	-2.7	-2.7
Owsley	KY	-135	-42	-46	-47	0	0	-1	1	324	1	-2.7	3.1
Baker	GA	-97	-16	-91	10	-65	-16	-43	-6	242	87	-2.7	-3.5
Ziebach	SD	-57	21	-70	-8	0	0	0	0	134	0	-2.6	-8.3
Wilkin	MN	-189	-61	-96	-31	2	1	-1	2	416	2	-2.5	107.6
Warren	MS	-1,200	-506	-235	-458	-807	-212	-257	-338	1,953	940	-2.5	-4.3
Magoffin	KY	-323	9	-19	-313	0	0	1	-1	1,427	2	-2.5	1.9
Pendleton	KY	-297	-320	-24	46	-3	12	-22	7	1,325	13	-2.5	-6.6
Grenada	MS	-529	-98	-305	-127	-289	-141	-103	-45	1,834	965	-2.5	-3.3
Ottawa	KS	-135	71	-120	-87	1	3	4	-6	860	1	-2.4	34.8
Pike	KY	-1,705	-710	-341	-654	18	5	1	12	6,246	41	-2.3	6.5
Jersey	IL	-482	-344	-52	-86	-2	-2	-3	2	374	5	-2.3	-2.4
Dyer	TN	-815	-43	-237	-535	-12	44	30	-86	1,217	164	-2.3	-0.3
Butler	MO	-902	-276	-119	-507	-90	-47	-24	-19	4,755	147	-2.3	-4.6
Floyd	KY	-1,009	-91	-208	-711	-35	72	-91	-16	5,471	27	-2.3	-11.4
Merrick	NE	-184	-20	-48	-116	6	0	2	4	1,464	6	-2.3	299.2
Webster	WV	-246	-85	-108	-53	1	1	1	-1	1,266	1	-2.3	29.3
Meigs	OH	-526	-193	-15	-318	0	-16	30	-14	2,570	61	-2.3	0.2

TABLE S8-E Apparent Emigration 1990–2020 from Inland 100-Year Floodplain by County, Ranked[1] as a Percent of 1990 Population													
	State	Apparent Migration All Races[2]				Apparent Migration, Black[2]				Population in 2020, Riverine A Zone[3]		Migration as % of county population[4]	
County		1990 to 2020	1990s	2000s	2010s	1990 to 2020	1990s	2000s	2010s	All Races	Black	All Races	Black
Blaine	MT	-153	-4	-85	-64	0	3	-3	0	462	0	-2.3	0.0
Calhoun	GA	-114	-32	-75	-7	-75	-34	-40	-1	250	204	-2.3	-2.6
Dallas	AL	-1,088	138	-618	-608	-529	230	-257	-502	4,166	3,378	-2.3	-1.9
Fayette	WV	-1,061	-199	-387	-475	-204	-88	-54	-62	1,585	95	-2.2	-6.8
Bracken	KY	-170	-130	4	-44	3	-1	0	3	465	4	-2.2	6.2
Calhoun	MS	-324	-34	-101	-190	-273	8	-79	-202	832	410	-2.2	-6.8
Custer	MT	-255	-16	-148	-91	25	1	4	19	6,277	32	-2.2	226.2
Miller	AR	-835	-466	-211	-158	-463	-229	-147	-87	1,921	756	-2.2	-5.4
Ottawa	OK	-663	-150	-425	-88	-8	-42	11	23	3,795	137	-2.2	-4.3
Scott	IL	-122	-17	-26	-79	0	1	-1	0	240	0	-2.2	0.0
Hinds	MS	-5,496	-1,882	-743	-2,871	1,139	1,951	1,289	-2,102	19,074	15,125	-2.2	0.9
Jefferson	AR	-1,842	-395	-798	-649	-1,022	-329	-295	-398	4,323	2,326	-2.2	-2.8
Forrest	MS	-1,460	-701	-572	-187	-1,020	-499	-180	-341	4,641	2,019	-2.1	-4.9
Nicholas	WV	-570	-182	-205	-183	1	0	2	-1	1,219	1	-2.1	26.6
Washington	OH	-1,317	125	-1,226	-216	-2	8	7	-17	5,400	66	-2.1	-0.2
Pocahontas	WV	-190	-99	-43	-48	-2	7	4	-12	741	2	-2.1	-2.3
Evangeline	LA	-700	-135	-275	-290	123	277	154	-308	4,121	1,458	-2.1	1.4
Franklin	NE	-80	-36	-14	-30	0	0	0	0	75	0	-2.0	0.4
Young	TX	-369	-82	-149	-137	-49	-8	-26	-16	1,044	20	-2.0	-19.0
Iron	MO	-213	-7	42	-248	21	9	3	9	1,117	26	-2.0	43.5
Massac	IL	-293	-44	12	-262	-25	-19	-8	3	842	80	-2.0	-2.8
Tyler	WV	-194	-18	-2	-174	1	0	1	0	952	1	-2.0	22.3
Claiborne	MS	-224	-44	-112	-69	-196	-44	-92	-60	237	223	-2.0	-2.1
St. Landry	LA	-1,576	160	-882	-853	-48	426	-265	-210	7,188	3,732	-2.0	-0.2
Edgecombe	NC	-1,109	-1,000	405	-514	-1,089	-846	182	-425	2,645	1,829	-2.0	-3.5
Allen	KS	-285	-16	-318	48	-19	6	-12	-12	627	4	-1.9	-7.0
Iroquois	IL	-581	182	-319	-444	20	13	2	5	2,986	28	-1.9	12.1
Chicot	AR	-296	234	38	-567	-124	165	21	-310	675	247	-1.9	-1.4

TABLE S8-E Apparent Emigration 1990–2020 from Inland 100-Year Floodplain by County, Ranked[1] as a Percent of 1990 Population

County	State	Apparent Migration All Races[2]				Apparent Migration, Black[2]				Population in 2020, Riverine A Zone[3]		Migration as % of county population[4]	
		1990 to 2020	1990s	2000s	2010s	1990 to 2020	1990s	2000s	2010s	All Races	Black	All Races	Black
Union	IL	-332	-58	-127	-147	-2	-1	-2	1	879	2	-1.9	-1.7
Schoharie	NY	-599	-356	159	-402	-1	-5	27	-23	1,325	16	-1.9	-0.3
Clinton	PA	-696	-332	-277	-87	21	-1	16	6	3,416	31	-1.9	14.6
Gilmer	WV	-144	-133	210	-221	3	0	121	-118	662	4	-1.9	10.1
Marshall	WV	-697	-237	-134	-325	30	0	10	20	2,185	38	-1.9	14.8
Athens	OH	-1,110	-4,134	4,227	-1,203	539	-258	235	563	11,929	1,067	-1.9	32.6
Marion	SC	-620	-94	-157	-369	-541	-181	-158	-202	839	453	-1.8	-2.9
Butler	IA	-282	-29	-244	-8	1	1	5	-5	1,353	1	-1.8	30.4
Sunflower	MS	-587	61	-389	-260	-400	-26	-204	-169	1,133	650	-1.8	-1.9
Delaware	NY	-837	-169	-193	-475	12	6	9	-3	2,714	29	-1.8	2.7
Kanawha	WV	-3,671	-833	-890	-1,948	291	199	27	65	19,859	1,242	-1.8	2.1
Kingfisher	OK	-233	-16	2	-219	-39	-14	-27	2	944	30	-1.8	-12.8
Wayne	MO	-203	-22	-72	-109	12	1	2	9	1,203	12	-1.8	168.2
Southampton	VA	-305	-23	-264	-18	-269	1	-203	-67	693	324	-1.7	-3.4
Lowndes	MS	-1,027	38	-872	-193	547	648	-130	29	5,414	3,125	-1.7	2.5
Cloud	KS	-191	-102	-67	-22	0	-3	-1	4	519	5	-1.7	0.1
Haywood	TN	-336	-110	-166	-60	-304	-135	-114	-54	769	486	-1.7	-3.2
Bedford	VA	-105	5	-59	-51	-47	6	-39	-14			-1.7	-3.5
Gibson	TN	-782	-258	-432	-92	-839	-249	-420	-170	1,046	616	-1.7	-9.4
Labette	KS	-399	-107	-229	-63	-30	-13	0	-18	1,123	53	-1.7	-3.0
Chariton	MO	-153	-87	-38	-28	11	6	-6	11	242	20	-1.7	3.2
Montgomery	IA	-201	-88	-166	53	2	1	2	-1	1,089	2	-1.7	40.0
Spencer	IN	-320	-108	-67	-145	1	0	-2	2	752	7	-1.6	0.7
Van Buren	IA	-126	-12	-98	-16	1	-1	2	0	655	2	-1.6	10.4
Tucker	WV	-125	-70	-28	-28	1	0	1	0	507	1	-1.6	25.5
Thayer	NE	-108	-7	-58	-42	1	0	0	0	236	1	-1.6	13.0
Hancock	TN	-109	59	-135	-34	-73	-73	-2	2	462	6	-1.6	-61.2
Franklin	LA	-360	-137	-60	-163	-118	-67	-4	-47	1,022	297	-1.6	-1.7

TABLE S8-E Apparent Emigration 1990–2020 from Inland 100-Year Floodplain by County, Ranked[1] as a Percent of 1990 Population

County	State	Apparent Migration All Races[2]				Apparent Migration, Black[2]				Population in 2020, Riverine A Zone[3]		Migration as % of county population[4]	
		1990 to 2020	1990s	2000s	2010s	1990 to 2020	1990s	2000s	2010s	All Races	Black	All Races	Black
Marion	MO	-443	-293	-71	-79	-50	-31	15	-33	646	49	-1.6	-4.0
Musselshell	MT	-66	-17	6	-55	0	0	0	0	237	0	-1.6	0.0
Stanton	NE	-99	42	-62	-79	0	2	-2	0	262	0	-1.6	0.0
Modoc	CA	-152	-53	-12	-87	-4	-4	0	-1	776	3	-1.6	-5.0
McLean	KY	-151	-30	-112	-8	3	0	0	3	477	3	-1.6	5.8
Choctaw	AL	-250	-46	-142	-61	-94	11	-62	-43	791	411	-1.6	-1.3
Carroll	OH	-410	-49	-186	-175	-8	1	3	-11	807	9	-1.5	-5.6
Garvin	OK	-411	-86	-367	42	-97	-41	-4	-52	3,504	107	-1.5	-13.5
Forest	PA	-72	-53	1	-20	-7	-7	0	0	204	0	-1.5	-17.4
Carlisle	KY	-78	-29	-18	-31	0	0	-1	1	128	1	-1.5	0.0
Mercer	ND	-146	-100	-76	29	1	0	1	1	713	1	-1.5	11.9
Clayton	IA	-284	-134	-120	-30	2	1	0	1	409	2	-1.5	27.2
Lincoln	MO	-429	-255	-45	-129	9	15	-25	19	1,360	42	-1.5	1.5
Ashley	AR	-358	23	-258	-123	-59	-9	-27	-23	882	115	-1.5	-0.9
Wirt	WV	-76	20	-34	-63	2	3	-3	2	539	2	-1.5	47.7
Drew	AR	-256	-116	-50	-89	-261	-160	-58	-44	710	221	-1.5	-5.5
Montgomery	AL	-3,043	-790	-1,024	-1,228	-2,301	-99	-686	-1,517	6,917	4,995	-1.5	-2.6
Poinsett	AR	-359	38	-266	-131	-27	-5	-3	-18	1,428	59	-1.5	-1.5
Belmont	OH	-1,032	-295	-603	-134	5	27	-1	-21	2,647	44	-1.5	0.4
Raleigh	WV	-1,092	-297	-307	-488	-118	-10	-86	-22	3,245	73	-1.4	-2.0
Greenbrier	WV	-482	-161	-193	-128	-75	-17	-16	-42	1,591	65	-1.4	-5.9
Ouachita	AR	-421	-175	-171	-75	-92	0	-91	-2	892	312	-1.4	-0.9
Dundy	NE	-35	-12	-30	6	0	0	0	0	108	0	-1.4	*
Dickenson	VA	-240	-80	-61	-99	-20	-10	-12	3	576	12	-1.4	-28.7
Wyoming	PA	-381	-148	-86	-147	11	-2	3	9	1,000	15	-1.4	8.5
Cambria	PA	-2,206	-1,209	-625	-373	255	-52	92	214	5,200	705	-1.4	6.9
Robeson	NC	-1,420	101	229	-1,750	-1,168	31	-281	-919	5,343	1,534	-1.4	-4.5
Hancock	WV	-475	-349	-66	-61	-35	-32	-3	1	722	11	-1.3	-3.8

TABLE S8-E Apparent Emigration 1990–2020 from Inland 100-Year Floodplain by County, Ranked[1] as a Percent of 1990 Population

County	State	Apparent Migration All Races[2]				Apparent Migration, Black[2]				Population in 2020, Riverine A Zone[3]		Migration as % of county population[4]	
		1990 to 2020	1990s	2000s	2010s	1990 to 2020	1990s	2000s	2010s	All Races	Black	All Races	Black
Carroll	MO	-145	-99	-20	-26	-14	-11	-3	1	141	5	-1.3	-6.5
Hocking	OH	-344	-79	-114	-151	-22	-10	-10	-2	2,173	17	-1.3	-9.6
Greene	IL	-206	-159	-7	-40	0	0	1	-1	152	0	-1.3	1.4
Jefferson	OH	-1,078	-495	-352	-230	27	20	-4	11	2,227	61	-1.3	0.6
Hettinger	ND	-46	-34	-15	3	0	0	0	0	34	0	-1.3	*
Saline	IL	-354	-147	-176	-32	-26	-38	-8	19	1,430	73	-1.3	-2.8
Shoshone	ID	-186	42	-424	196	-1	-2	1	-1	3,557	4	-1.3	-8.6
DeWitt	TX	-248	-73	-78	-97	-65	-36	-21	-9	1,262	73	-1.3	-3.3
Pawnee	NE	-44	-39	-7	3	0	0	0	0	76	0	-1.3	0.0
Bullock	AL	-144	14	-95	-63	-147	2	-74	-75	350	256	-1.3	-1.8
Lee	VA	-320	-21	-143	-156	5	3	1	1	874	6	-1.3	5.1
Knox	NE	-123	-79	-20	-23	0	0	0	0	605	0	-1.3	*
Braxton	WV	-167	28	-21	-173	0	2	2	-3	861	2	-1.3	0.8
Tioga	NY	-669	-183	-103	-383	15	11	17	-14	3,922	34	-1.3	5.0
Lake	SD	-134	-63	-7	-64	3	0	3	-1	434	5	-1.3	22.1
Posey	IN	-328	72	-275	-125	4	0	4	0	1,024	4	-1.3	1.3
Lake	TN	-89	-44	-24	-22	-65	-38	2	-29	133	40	-1.3	-3.8
Buchanan	IA	-259	-139	-96	-23	2	3	0	0	1,010	3	-1.2	6.5
Webster	KY	-173	-63	10	-119	0	-3	2	2	868	9	-1.2	0.1
Wilcox	AL	-166	-24	-74	-69	-246	-107	-26	-113	703	452	-1.2	-2.6
Jenkins	GA	-100	-83	0	-17	-81	-70	7	-19	296	153	-1.2	-2.4
Pennington	SD	-988	-896	3	-95	-158	-146	10	-22	2,529	20	-1.2	-12.6
Hancock	OH	-795	-99	-435	-261	15	-18	34	0	3,983	110	-1.2	2.7
McCook	SD	-68	-12	-23	-33	0	0	0	0	162	0	-1.2	*
Gasconade	MO	-167	17	-97	-87	0	2	-2	0	386	0	-1.2	1.2
Adams	OH	-302	-161	-43	-98	4	0	1	3	935	6	-1.2	7.8
Nance	NE	-50	-12	-25	-14	0	0	0	0	151	0	-1.2	0.0
Grant	LA	-206	67	-96	-177	-279	-85	-105	-89	1,224	308	-1.2	-11.0

TABLE S8-E Apparent Emigration 1990–2020 from Inland 100-Year Floodplain by County, Ranked[1] as a Percent of 1990 Population

	State	Apparent Migration All Races[2]				Apparent Migration, Black[2]				Population in 2020, Riverine A Zone[3]		Migration as % of county population[4]	
County		1990 to 2020	1990s	2000s	2010s	1990 to 2020	1990s	2000s	2010s	All Races	Black	All Races	Black
Jackson	IL	-717	-318	123	-522	20	-8	72	-44	1,472	59	-1.2	0.3
Wise	VA	-462	-21	-100	-341	-1	6	-4	-4	2,650	45	-1.2	-0.2
Bay	MI	-1,297	72	-1,051	-318	26	37	-11	0	7,040	60	-1.2	2.1
Obion	TN	-368	-48	-176	-143	-174	-101	-10	-63	1,051	131	-1.2	-5.4
Greene	AL	-117	37	-40	-114	-147	-30	-44	-74	571	393	-1.2	-1.8
Ellsworth	KS	-75	-73	-32	30	0	-1	1	0	183	1	-1.1	0.3
Traill	ND	-100	-54	-50	3	0	0	0	0	272	0	-1.1	2.3
Chenango	NY	-589	-197	-224	-167	35	1	32	3	3,476	54	-1.1	10.0
Martin	IN	-117	11	-104	-24	1	0	1	0	421	1	-1.1	10.2
Sullivan	PA	-69	-24	-59	15	0	1	0	-1	295	0	-1.1	-0.1
Greene	PA	-442	-24	-185	-234	-2	-2	3	-2	1,015	4	-1.1	-0.5
Wilson	KS	-115	-42	-96	23	1	1	-2	2	228	2	-1.1	4.3
Morgan	IL	-404	-237	-5	-161	-26	-28	-4	5	1,153	61	-1.1	-1.7
Lee	KY	-81	-138	5	52	-6	-7	1	0	417	2	-1.1	-22.7
Bottineau	ND	-87	-47	-44	3	0	0	0	0	98	0	-1.1	0.0
Crawford	WI	-170	5	-137	-38	2	0	2	-1	545	2	-1.1	3.4
Clay	KY	-229	341	-422	-147	25	30	-11	6	2,050	28	-1.1	7.6
Morgan	OH	-148	49	-69	-129	-1	8	-7	-2	617	13	-1.0	-0.2
Marlboro	SC	-306	-154	-125	-28	-366	-159	-156	-51	553	370	-1.0	-2.6
Hickman	KY	-58	-18	-24	-15	-24	-8	-5	-11	125	30	-1.0	-4.8
Tazewell	VA	-476	-47	-225	-204	-44	7	-16	-35	2,400	38	-1.0	-3.7
Montgomery	NY	-535	-331	34	-238	-10	-28	19	0	1,179	22	-1.0	-2.8
Fulton	KY	-84	-11	-24	-49	-26	16	-9	-34	226	53	-1.0	-1.7
Osage	MO	-122	-26	-31	-65	-1	-1	0	0	402	0	-1.0	-1.8
Caddo	OK	-298	-144	-118	-36	6	5	-1	1	630	7	-1.0	0.8
Norton	VA	-43	-44	11	-10	0	3	2	-5	185	7	-1.0	0.0
Upshur	WV	-228	-152	-15	-61	7	3	1	3	1,841	18	-1.0	6.0
Berrien	GA	-141	-107	37	-71	-152	-71	1	-82	620	176	-1.0	-9.3

TABLE S8-E Apparent Emigration 1990–2020 from Inland 100-Year Floodplain by County, Ranked[1] as a Percent of 1990 Population														
	State	Apparent Migration All Races[2]				Apparent Migration, Black[2]				Population in 2020, Riverine A Zone[3]		Migration as % of county population[4]	
County		1990 to 2020	1990s	2000s	2010s	1990 to 2020	1990s	2000s	2010s	All Races	Black	All Races	Black
Keith	NE	-85	5	-94	4	1	0	2	0	210	1	-1.0	14.7
Lawrence	AR	-173	4	-101	-76	17	4	33	-21	1,699	22	-1.0	18.9
Garden	NE	-24	-35	-13	23	0	0	0	0	238	0	-1.0	*
Lincoln	NV	-37	-24	54	-67	9	1	11	-3	413	9	-1.0	11.9
Cass	IL	-132	-15	-58	-60	8	5	4	-1	639	8	-1.0	52.4
McKean	PA	-461	-299	-94	-69	7	-20	13	15	2,122	31	-1.0	1.6
Harrison	KY	-159	-74	-34	-51	-132	-73	-18	-40	841	80	-1.0	-27.7
Hancock	IL	-208	-185	0	-24	2	0	1	2	278	2	-1.0	7.6
Gage	NE	-222	-135	-106	20	6	2	-2	6	753	7	-1.0	12.4
Newton	IN	-131	70	-147	-54	2	0	0	2	375	2	-1.0	25.0
Siskiyou	CA	-418	-192	-213	-13	-1	9	-11	1	2,920	17	-1.0	-0.1
Dallas	AR	-92	22	-69	-44	-55	22	-46	-32	148	47	-1.0	-1.5
Jefferson	AL	-6,219	-2,506	-2,284	-1,429	-4,454	-1,628	-1,497	-1,330	11,387	6,130	-1.0	-2.0
Lycoming	PA	-1,096	-299	-457	-340	30	57	-43	16	7,223	56	-0.9	1.1
Schuylkill	PA	-1,398	-518	-340	-540	58	6	68	-16	6,868	67	-0.9	7.2
Black Hawk	IA	-1,089	-663	-422	-3	28	30	6	-8	3,003	253	-0.9	0.3
Washington	PA	-1,671	-695	-662	-314	2	8	18	-24	5,077	190	-0.8	0.0
Fayette	PA	-1,173	-350	-558	-265	8	-26	34	0	3,091	116	-0.8	0.2
Rock Island	IL	-1,081	-387	-542	-152	161	6	77	78	4,347	218	-0.7	1.6
St. Charles	MO	-1,409	-1,699	74	216	153	30	37	86	3,791	181	-0.7	3.1
Ingham	MI	-1,763	-2,354	218	373	95	-152	-108	356	7,710	974	-0.6	0.3
Linn	IA	-1,004	-19	-1,646	661	136	67	-44	113	2,665	214	-0.6	4.1
Luzerne	PA	-1,822	-422	-979	-422	393	61	155	178	5,606	453	-0.6	10.4
Richmond	GA	-1,030	-452	-325	-253	-397	-121	-80	-196	2,398	1,218	-0.5	-0.5
Westmoreland	PA	-1,391	-290	-755	-346	-57	-31	-15	-11	5,154	127	-0.4	-0.8
Will	IL	-1,158	531	-458	-1,230	-1,992	-647	-614	-730	9,866	1,590	-0.3	-5.3
Mobile	AL	-1,181	-456	-359	-366	-589	-95	-357	-138	3,527	1,693	-0.3	-0.5
Allegheny	PA	-3,057	-1,256	-1,824	23	-212	124	-289	-47	12,569	1,706	-0.2	-0.1

TABLE S8-E Apparent Emigration 1990–2020 from Inland 100-Year Floodplain by County, Ranked[1] as a Percent of 1990 Population

	State	Apparent Migration All Races[2]				Apparent Migration, Black[2]				Population in 2020, Riverine A Zone[3]		Migration as % of county population[4]	
County		1990 to 2020	1990s	2000s	2010s	1990 to 2020	1990s	2000s	2010s	All Races	Black	All Races	Black
El Paso	TX	-1,227	-408	-240	-579	-23	8	-17	-13	5,298	53	-0.2	-0.1
St. Louis	MO	-1,928	-764	-1,015	-149	480	905	-175	-249	9,193	4,686	-0.2	0.3
Wayne	MI	-1,710	-2,261	-774	1,324	1,060	-233	746	546	32,570	4,762	-0.1	0.1
Total[5]		-243,390	-117,775	-145,833	-130,283	-66,005	-28,873	-39,811	-44,087	4,970,822	569,828	0.10	0.02
Total, Excluding Displacements[6]						-47,047	-20,130	-27,940	-32,011				

1. Ranked by "Migration as % of county population" where that percentage is greater than 0.95, plus other counties where Apparent Migration is greater than 1000.
2. Change in population of the A zones as mapped by the National Flood Hazard Layer. In states with tidal waters, these calculations only include Census blocks that are entirely above the highest storm-surge base flood elevation in the state. Includes migration into and out of this land as well as births and deaths. Negative numbers mean emigration out of this county's inland A zone. All calculations use the building-based density assumption.
3. The population of the inland A zone.
4. Equal to "Apparent Migration" for 1990 to 2020 as a percent of the population of 1990, with numerator and denominator using the same racial classification in both cases.
5. Sum of emigration by counties with net emigration from flood-zone for stated racial classification.
6. Sum of Black emigration or total Emigration, whichever is less, by county. (If Black emigration is greater than total emigration, the difference represents displacement of Black residents by people from other racial groups.

* The 1990 Black population for this county was zero.

The purpose of this table is to identify inland counties where the population vulnerable to flooding is declining.

Begin

TABLE S8-F Apparent Emigration 1990–2020 from Coastal 100-Year Floodplains by County, Ranked[1] as a Percent of 1990 Population

	State	Apparent Migration All Races[2]				Apparent Migration, Black[2]				Population in 2020, Coastal A Zone		Migration as % of county population[3]	
County		1990 to 2020	1990s	2000s	2010s	1990 to 2020	1990s	2000s	2010s	All Races	Black	All Races	Black
Cameron	LA	-3,773	242	-3,081	-934	-398	-107	-274	-17	4,306	64	-40.7	-79.9
Tyrrell	NC	-540	264	241	-1,045	-527	88	45	-659	2,523	747	-14.0	-34.2
Hyde	NC	-710	274	69	-1,053	-549	182	-150	-581	4,208	1,038	-13.1	-30.9
Cape May	NJ	-6,117	1,526	-6,593	-1,050	-1,151	-187	-588	-377	30,607	763	-6.4	-22.0
St. Bernard	LA	-4,252	-358	-5,097	1,203	1,205	44	399	762	5,276	1,384	-6.4	39.0
St. Mary	LA	-2,928	-1,562	-16	-1,350	-1,222	-460	-123	-639	12,455	3,985	-5.0	-6.7
Somerset	MD	-1,163	-282	-429	-451	-620	-113	-216	-292	7,836	1,322	-5.0	-7.0
Beaufort	NC	-1,841	-534	-102	-1,205	-1,852	-390	-566	-896	12,933	3,506	-4.4	-14.1
Orleans	LA	-16,892	2,524	-27,912	8,496	-14,760	6,254	-21,051	38	53,414	29,602	-3.4	-4.8
Accomack	VA	-862	663	-1,507	-17	-237	-78	-154	-4	5,708	314	-2.7	-2.2
Atlantic	NJ	-5,981	19	-3,897	-2,103	-111	10	-43	-79	15,413	264	-2.7	-0.3
Acadia	LA	-1,470	106	107	-1,683	-43	193	58	-294	13,034	2,391	-2.6	-0.4
Newton	TX	-325	53	-138	-240	13	0	11	3	640	13	-2.4	0.4
Washington	NC	-329	6	-43	-291	-232	-45	-55	-132	872	298	-2.3	-3.7
Glades	FL	-165	-91	164	-238	-119	-126	-7	14	617	24	-2.2	-12.9
Harrison	MS	-3,553	617	-6,746	2,576	-489	276	-1,326	561	24,448	7,362	-2.1	-1.5
Hampton	VA	-2,778	3,904	-7,387	706	1,353	2,220	-1,794	926	24,532	9,226	-2.1	2.6
Dorchester	MD	-605	15	-372	-248	-65	-19	-24	-21	2,722	87	-2.0	-0.8
Iberville	LA	-611	938	328	-1,876	-387	486	128	-1,001	3,790	374	-2.0	-2.7
Salem	NJ	-1,264	-978	-63	-223	406	43	66	296	10,470	1,371	-1.9	4.3
Pamlico	NC	-211	223	97	-531	-696	-160	-309	-227	4,005	675	-1.9	-23.7
Mathews	VA	-148	207	-307	-48	-9	33	-42	1	2,289	86	-1.8	-0.8
Gloucester	VA	-329	-213	-269	153	-2	-11	-4	13	3,110	89	-1.1	-0.1
Aransas	TX	-161	493	-335	-318	-9	-11	2	0	1,984	20	-0.9	-3.1
Newport	RI	-745	-111	-567	-66	-71	73	-131	-13	3,554	144	-0.9	-2.2
Newport News	VA	-1,422	-602	-831	11	-567	-195	-328	-44	1,741	1,080	-0.8	-1.0
Grays Harbor	WA	-517	-228	-304	15	90	15	79	-5	12,601	123	-0.8	77.2

TABLE S8-F Apparent Emigration 1990–2020 from Coastal 100-Year Floodplains by County, Ranked[1] as a Percent of 1990 Population

County	State	Apparent Migration All Races[2]					Apparent Migration, Black[2]				Population in 2020, Coastal A Zone		Migration as % of county population[3]	
		1990 to 2020	1990s	2000s	2010s		1990 to 2020	1990s	2000s	2010s	All Races	Black	All Races	Black
Talbot	MD	-243	-87	-115	-40		-77	-27	-33	-17	849	30	-0.8	-1.4
Bertie	NC	-161	-66	41	-136		-35	16	-6	-45	229	101	-0.8	-0.3
Southampton	VA	-124	-34	-79	-11		-106	-44	-31	-30	420	184	-0.7	-1.4
Portsmouth	VA	-709	-833	-1,025	1,149		-343	-266	-336	259	10,794	5,137	-0.7	-0.7
Jefferson	LA	-2,434	1,381	-10,368	6,552		10,108	6,928	1,486	1,694	144,048	33,269	-0.5	12.9
Del Norte	CA	-120	-195	93	-17		-3	-2	0	-1	457	0	-0.5	-0.3
Mobile	AL	-1,867	930	-2,622	-176		446	1,127	-477	-203	16,544	5,294	-0.5	0.4
Nantucket	MA	-29	144	-212	39		7	106	-103	5	229	12	-0.5	5.0
Franklin	VA	-36	-88	87	-35		12	-39	78	-27	154	109	-0.5	0.3
Kent	MD	-71	39	-115	5		-45	-6	-40	1	515	36	-0.4	-1.3
Gloucester	NJ	-860	-617	-152	-92		-51	-26	-20	-6	3,036	408	-0.4	-0.3
Washington	RI	-394	117	-535	25		-5	-4	-20	18	4,512	34	-0.4	-0.5
Wharton	TX	-137	-137	-1	1		-4	-4	0	0	4	0	-0.3	-0.1
Kleberg	TX	-92	-94	17	-15		1	0	0	1	148	1	-0.3	0.1
Lancaster	VA	-31	60	-106	15		25	1	9	15	482	43	-0.3	0.8
Yolo	CA	-375	-95	-420	139		-1	5	-9	3	1,868	8	-0.3	0.0
Caroline	MD	-71	-40	-4	-27		-96	-61	-9	-25	737	201	-0.3	-2.2
Mendocino	CA	-203	-174	-75	46		0	2	-3	1	146	1	-0.3	0.1
Washington	AL	-41	25	-63	-3		-50	10	-42	-18	141	73	-0.2	-1.1
Jackson	MS	-273	2,688	-3,137	176		3,253	2,414	233	606	34,213	8,494	-0.2	13.8
St. James	LA	-49	-48	43	-44		-20	-34	34	-21	1,033	396	-0.2	-0.2
Jefferson	FL	-25	-22	-4	1		-17	-19	0	2	5	2	-0.2	-0.4
Cumberland	NJ	-298	5	-267	-36		-144	79	-229	6	1,635	186	-0.2	-0.6
Lenoir	NC	-118	-88	34	-64		-39	-14	-4	-20	232	93	-0.2	-0.2
Clarke	AL	-54	-3	-44	-7		-58	-17	-28	-13	35	22	-0.2	-0.5
Hardin	TX	-80	125	-86	-119		13	2	6	6	783	18	-0.2	0.4
Northampton	VA	-22	33	-73	18		-7	7	-23	9	236	22	-0.2	-0.1
Humboldt	CA	-204	-52	-21	-131		2	-2	19	-14	2,019	26	-0.2	0.3

TABLE S8-F Apparent Emigration 1990–2020 from Coastal 100-Year Floodplains by County, Ranked[1] as a Percent of 1990 Population

	State	Apparent Migration All Races[2]				Apparent Migration, Black[2]				Population in 2020, Coastal A Zone		Migration as % of county population[3]	
County		1990 to 2020	1990s	2000s	2010s	1990 to 2020	1990s	2000s	2010s	All Races	Black	All Races	Black
New London	CT	-431	-452	3	18	-26	-74	34	14	5,852	123	-0.2	-0.2
Pitt	NC	-180	-360	409	-230	-159	-246	222	-135	2,646	959	-0.2	-0.4
Camden	NJ	-719	157	-933	57	-1,182	81	-737	-526	9,075	2,975	-0.14	-1.5
Washington	ME	-49	-6	-23	-20	2	0	0	2	211	2	-0.14	2.6
Hertford	NC	-30	9	-39	0	-11	24	-30	-4	95	21	-0.13	-0.08
Edgecombe	NC	-75	-71	6	-10	2	-7	7	1	30	18	-0.13	0.00
Kent	RI	-194	86	-438	158	17	10	-4	11	2,264	40	-0.12	1.69
Orange	CA	-2,674	-1,622	-2,314	1,262	-41	-9	-8	-25	17,212	76	-0.11	-0.11
Kennebec	ME	-124	-91	3	-36	5	-3	1	7	401	10	-0.11	2.13
Dutchess	NY	-260	93	-41	-312	4	4	-8	8	297	26	-0.10	0.02
Harford	MD	-180	-272	63	29	-43	-70	11	15	475	89	-0.10	-0.28
Williamsburg	SC	-34	-22	-14	2	-29	-17	-9	-3	99	43	-0.09	-0.12
Sonoma	CA	-356	-425	44	25	-3	-3	4	-5	1,429	8	-0.09	-0.06
Greene	NY	-40	73	-98	-15	-15	27	-31	-11	330	20	-0.09	-0.77
Middlesex	NJ	-538	22	58	-618	-64	-118	49	5	3,720	578	-0.08	-0.13
Mercer	NJ	-260	-18	-244	2	-18	23	-39	-2	37	12	-0.08	-0.03
Jackson	FL	-30	-23	-7	0	-1	-1	0	0	3	0	-0.07	-0.01
Jones	NC	-7	14	11	-32	-32	-6	-8	-17	174	47	-0.07	-0.86
San Luis Obispo	CA	-149	495	-942	299	14	12	-13	16	1,581	21	-0.07	0.33
Sutter	CA	-41	-26	-96	81	0	0	0	0	228	0	-0.06	0.00
San Joaquin	CA	-267	536	-1,689	886	30	492	-30	-432	22,210	1,480	-0.06	0.12
Evangeline	LA	-18	86	-95	-9	-11	29	-36	-4	84	8	-0.05	-0.12
Arlington	VA	-80	24	-104	0	-19	3	-22	0	2	0	-0.05	-0.11
Waldo	ME	-15	-15	1	-1	0	0	0	0	41	0	-0.05	-0.20
Philadelphia	PA	-622	-2,137	1,584	-69	-411	79	-52	-438	8,221	3,713	-0.04	-0.07
Rockland	NY	-96	90	-111	-75	0	0	-6	5	346	22	-0.04	0.00
Hampton	SC	-6	17	-26	3	-27	-9	-8	-10	53	19	-0.03	-0.28
Greene	NC	-5	-4	5	-6	-1	-3	5	-2	14	9	-0.03	-0.02

TABLE S8-F Apparent Emigration 1990–2020 from Coastal 100-Year Floodplains by County, Ranked[1] as a Percent of 1990 Population													
	State	Apparent Migration All Races[2]				Apparent Migration, Black[2]				Population in 2020, Coastal A Zone		Migration as % of county population[3]	
County		1990 to 2020	1990s	2000s	2010s	1990 to 2020	1990s	2000s	2010s	All Races	Black	All Races	Black
Douglas	OR	-30	-173	74	68	-3	-3	2	-2	345	0	-0.03	-1.86
Monroe	AL	-7	-2	-2	-3	-2	-6	-1	4	15	5	-0.03	-0.02
Orange	NY	-77	-51	-44	18	2	1	1	0	116	6	-0.03	0.01
Osceola	FL	-24	-19	-11	6	0	0	0	0	10	0	-0.02	0.00
Passaic	NJ	-100	105	-218	12	24	17	0	7	954	73	-0.02	0.04
Halifax	NC	-11	-11	-1	1	-9	-9	0	0	1	0	-0.02	-0.03
Delaware	PA	-110	-38	827	-899	242	70	540	-367	1,680	591	-0.02	0.40
Calhoun	FL	-2	10	-9	-3	0	0	0	0	64	0	-0.02	0.00
Jasper	TX	-6	1	10	-16	0	0	0	0	175	0	-0.02	0.00
Bucks	PA	-99	-108	-187	196	44	39	-5	9	1,418	80	-0.02	0.30
Bee	TX	-4	-4	0	0	0	0	0	0	0	0	-0.02	0.00
Los Angeles	CA	-1,172	-737	-424	-11	54	38	-27	43	12,896	244	-0.01	0.01
**Total[4]**	US	-77,991	-19,779	-136,056	-28,597	-53,165	-14,693	-44,378	-24,568	4,382,377	495,262	-0.03	-0.01
**Total, Excluding displacement[6]**	US					-26,296	-3,196	-35,005	-9,301				

1. Ranked by "Migration as % of county population". All calculations use the building-based density assumption.
2. Change in population of the A zones as mapped by the National Flood Hazard Layer. These calculations only include Census blocks with land below the highest storm-surge base flood elevation in the state. Includes migration into and out of this land as well as births and deaths. Negative numbers mean emigration out of this county's coastal A zone.
3. Equal to "Apparent Migration" for 1990 to 2020 as a percent of the population of 1990, with numerator and denominator using the same racial classification in both cases.
4. Sum of emigration by counties with net emigration from coastal A zone for stated racial classification.
5. Sum of Black emigration or total Emigration, whichever is less, by county. (If Black emigration is greater than total emigration, the difference represents displacement of Black residents by people from other racial groups.)

The purpose of this table is to identify coastal counties where the population vulnerable to flooding is declining.

Begin

TABLE S8-G Apparent Emigration 1990–2020 from 500-Year Floodplain by County, Ranked[1] by Emigration from X500 Zone

	State	Apparent Migration All Races out of …[2]						Apparent Migration[2] 1990 to 2020			Population in 2020, X500 Zone[3]				Migration as % of county population[4]		
		500 Year Floodplain (X500 zone)				A Zone											
County		1990s	2000s	2010s	1990 to 2020	1990 to 2020		Hispanic	Black		All Races	Black	Hispanic		All Races	Black	Hisp.
Orleans	LA	-9,107	-81,532	28,193	-62,445	-16,892		5,306	-47,140		192,093	125,533	13,370		-12.6	30.8	-15.5
St. Bernard	LA	783	-25,048	6,617	-17,647	-4,252		1,935	7,082		38,246	9,969	5,195		-26.5	46.2	229.1
Mississippi	AR	-5,354	-4,971	-5,502	-15,827	-425		921	-1,653		38,745	13,881	1,629		-27.5	123.7	-10.3
Kanawha	WV	-4,904	-2,321	-2,258	-9,483	-3,671		447	-1,273		36,805	4,340	696		-4.6	49.9	-9.3
Bolivar	MS	-1,088	-5,174	-2,774	-9,036	-1,443		404	-5,519		26,912	16,258	720		-21.6	107.0	-21.1
Phillips	AR	-685	-1,950	-2,148	-4,784	-1,598		11	-2,612		5,464	2,929	95		-16.6	4.6	-16.6
Wayne	MI	-1,666	-2,504	-367	-4,537	-1,710		1,350	1,637		32,840	10,503	2,180		-0.2	2.7	0.2
El Paso	TX	-1,497	-1,144	-1,533	-4,174	-1,227		-3,104	-77		18,062	142	17,079		-0.7	-0.8	-0.4
St. Mary	LA	-2,516	1,627	-3,046	-3,935	-2,928		2,453	-1,218		30,685	8,579	3,246		-6.8	217.5	-6.7
Montgomery	AL	-1,318	76	-1,146	-2,387	-3,043		208	-1,194		6,671	4,539	289		-1.1	12.8	-1.4
Edgecombe	NC	-2,735	1,930	-1,429	-2,234	-1,184		155	-2,317		3,652	2,775	180		-3.9	60.8	-7.3
Acadia	LA	-454	-587	-1,104	-2,144	-1,437		149	-953		6,694	2,704	208		-3.8	40.1	-9.4
Montgomery	OH	-704	-1,249	8	-1,945	-381		362	418		8,680	1,319	423		-0.3	8.0	0.4
Northumberland	PA	-877	-591	-363	-1,831	-509		850	278		10,617	335	1,024		-1.9	159.8	89.3
Mobile	AL	-251	-1,274	-232	-1,757	-3,049		221	-891		10,905	5,080	315		-0.5	7.0	-0.8
Hampton	VA	1,523	-2,353	-618	-1,448	-2,356		780	1,037		19,607	8,896	1,237		-1.1	29.6	2.0
Dallas	AL	-352	-365	-545	-1,262	-1,088		4	-801		2,150	1,799	13		-2.6	2.7	-2.9
Dougherty	GA	-334	-483	-401	-1,218	-2,686		35	-867		2,098	1,691	55		-1.3	4.3	-1.8
Barton	KS	-234	303	-1,231	-1,162	-242		2,748	-11		14,107	205	3,341		-4.0	336.8	-3.3
Camden	NJ	-1,438	434	-123	-1,127	-310		1,477	-424		12,459	3,733	4,233		-0.2	4.1	-0.5
Caddo	LA	-512	-136	-462	-1,111	1,166		122	-845		4,489	1,736	161		-0.4	4.7	-0.9
Willacy	TX	364	-895	-567	-1,097	-46		-769	5		5,547	42	5,151		-6.2	-5.1	6.5
Mingo	WV	-795	-28	-274	-1,097	-1,372		8	-18		2,025	55	18		-3.3	6.5	-2.2
Miami	IN	-176	-605	-281	-1,061	57		58	-80		3,816	70	104		-2.9	10.7	-7.4
Floyd	KY	-534	-175	-319	-1,028	-1,009		46	-48		4,131	22	62		-2.4	36.0	-15.8
Lenoir	NC	-285	-44	-617	-946	-1,786		142	-933		2,686	1,519	170		-1.7	30.7	-4.2
St. John the Baptist	LA	716	1,307	-2,967	-943	3,963		1,640	5,024		32,533	18,244	2,360		-2.4	171.9	35.0
Hinds	MS	257	-782	-416	-941	-5,496		121	1,242		7,212	5,031	158		-0.4	10.5	1.0

TABLE S8-G Apparent Emigration 1990–2020 from 500-Year Floodplain by County, Ranked[1] by Emigration from X500 Zone

	State	Apparent Migration All Races out of …[2]						Apparent Migration[2] 1990 to 2020			Population in 2020, X500 Zone[3]				Migration as % of county population[4]		
		500 Year Floodplain (X500 zone)				A Zone											
County		1990s	2000s	2010s	1990 to 2020	1990 to 2020		Hispanic	Black		All Races	Black	Hispanic		All Races	Black	Hisp.
Cambria	PA	-252	-307	-340	-899	-2,206		31	87		1,377	193	58		-0.6	3.2	2.4
Luzerne	PA	-237	-459	-165	-861	-1,822		205	101		4,347	110	218		-0.3	10.1	2.7
Washington	PA	-482	-241	-127	-850	-1,671		47	12		1,976	101	62		-0.4	4.0	0.2
Pike	KY	-499	-197	-150	-846	-1,705		52	22		3,691	27	62		-1.2	28.1	7.9
Jefferson	AL	-421	-318	-89	-829	-6,219		325	-1,067		5,331	2,570	344		-0.1	11.8	-0.5
Salem	NJ	-645	-259	83	-821	-1,251		945	-100		9,545	2,356	1,264		-1.3	65.8	-1.1
New London	CT	-679	155	-297	-821	-197		568	-314		10,161	508	1,187		-0.3	6.7	-2.7
Prowers	CO	572	-1,221	-151	-801	-43		602	19		5,111	31	2,503		-6.0	19.4	49.0
St. Louis	MO	-224	-186	-365	-775	-1,928		345	1,656		11,457	4,881	457		-0.1	3.5	1.2
Crittenden	AR	567	864	-2,204	-773	-336		1,029	4,944		46,054	24,742	1,342		-1.5	306.2	23.2
Fayette	WV	-419	-27	-324	-770	-1,061		6	-58		1,920	129	21		-1.6	2.4	-1.9
Cook	IL	-758	-612	630	-739	3,396		10,908	4,041		79,448	22,319	15,099		0.0	1.6	0.3
St. Louis	MO	-60	-335	-336	-732	-136		36	198		1,653	720	65		-0.2	0.7	0.1
Portsmouth	VA	-1,649	426	517	-706	-709		695	848		19,592	9,552	991		-0.7	51.0	1.7
Newport	RI	-611	122	-196	-685	-730		165	-78		3,856	138	256		-0.8	9.6	-2.4
Saline	KS	13	-232	-466	-685	-110		1,511	49		14,552	482	1,885		-1.4	123.7	3.3
Butler	MO	-53	-65	-547	-664	-902		43	-118		2,659	177	61		-1.7	19.9	-6.0
Wayne	NC	254	-557	-353	-657	-444		232	-518		1,683	804	245		-0.6	17.1	-1.5
Osage	OK	-162	-168	-308	-637	-5		26	-41		1,526	18	69		-1.5	3.9	-1.0
Pulaski	AR	-616	142	-161	-635	792		560	257		8,576	4,426	601		-0.2	17.5	0.3
Jim Wells	TX	-518	224	-333	-627	-170		-265	18		4,599	31	4,047		-1.7	-1.0	9.5
Cape May	NJ	180	-458	-337	-615	-6,117		203	-99		4,250	100	275		-0.6	10.9	-1.9
Anne Arundel	MD	67	-816	149	-600	386		88	-38		2,773	277	181		-0.1	1.3	-0.1
Mills	IA	6	-24	-568	-586	-52		27	2		532	2	27		-4.4	37.2	7.2
Trumbull	OH	115	-602	-96	-583	-420		47	-180		3,011	375	63		-0.3	3.2	-1.2
Baltimore	MD	-387	-319	160	-546	291		249	-105		4,940	1,299	293		-0.1	3.1	-0.1
Potter	TX	1	-188	-338	-526	-55		261	68		2,075	211	688		-0.5	1.4	0.8
Kent	RI	128	-584	-68	-524	-343		393	67		7,197	141	474		-0.3	22.6	6.6
Yazoo	MS	-197	70	-392	-519	-728		-4	-472		2,174	2,095	15		-2.0	-3.6	-3.5
McDowell	WV	-303	-119	-92	-513	-2,915		5	-28		852	78	11		-1.5	2.6	-0.6

TABLE S8-G Apparent Emigration 1990–2020 from 500-Year Floodplain by County, Ranked[1] by Emigration from X500 Zone

	State	Apparent Migration All Races out of …[2]					Apparent Migration[2] 1990 to 2020		Population in 2020, X500 Zone[3]			Migration as % of county population[4]		
		500 Year Floodplain (X500 zone)				A Zone								
County		1990s	2000s	2010s	1990 to 2020	1990 to 2020	Hispanic	Black	All Races	Black	Hispanic	All Races	Black	Hisp.
Leflore	MS	152	-355	-302	-504	-1,403	73	-214	709	437	76	-1.4	50.3	-0.9
Forrest	MS	-203	-154	-126	-483	-1,460	158	61	2,687	998	173	-0.7	31.7	0.3
Atlantic	NJ	577	-776	-254	-453	-5,971	211	62	3,764	255	395	-0.2	1.3	0.2
Rock Island	IL	-590	427	-288	-451	-1,081	215	-125	2,889	343	356	-0.3	2.7	-1.2
St. James	LA	502	900	-1,849	-447	-49	212	-745	18,891	8,901	312	-2.1	197.7	-7.2
Tuscarawas	OH	252	-363	-329	-440	-337	110	-106	5,479	60	128	-0.5	48.0	-17.0
Grant	LA	-291	-86	-53	-430	-206	5	-417	1,799	901	23	-2.5	3.5	-16.5
Bay	FL	-168	-121	-135	-423	6,611	103	31	2,222	226	158	-0.3	4.6	0.2
Jefferson	AR	-249	81	-254	-422	-1,842	12	161	1,064	755	24	-0.5	2.8	0.4
Belmont	OH	-44	-288	-88	-420	-1,032	8	9	1,527	51	17	-0.6	4.0	0.7
Jefferson	LA	293	-1,365	661	-411	-2,434	4,015	3,600	31,659	8,514	6,235	-0.1	15.1	4.6
Lowndes	MS	-181	-35	-192	-408	-1,027	36	378	2,709	1,865	70	-0.7	7.4	1.7
Armstrong	PA	-45	-228	-135	-407	-620	1	-1	2,023	29	13	-0.6	0.6	-0.2
Westmoreland	PA	-203	-101	-104	-407	-1,391	37	12	2,707	123	49	-0.1	2.7	0.2
Gallia	OH	-104	-279	-22	-405	-970	8	-8	1,464	61	20	-1.3	5.2	-1.0
Pottawattamie	IA	21	-169	-255	-402	26	509	119	4,423	163	661	-0.5	33.6	26.2
Jefferson Davis	LA	-194	-78	-130	-402	127	25	-70	2,198	281	31	-1.3	12.3	-1.2
Union	KY	-97	-124	-167	-388	-77	10	-146	1,545	102	21	-2.3	8.2	-5.8
Fayette	PA	-142	-157	-87	-386	-1,173	2	-32	894	55	13	-0.3	0.5	-0.6
Logan	WV	-244	46	-185	-384	-2,225	-4	-16	1,727	20	17	-0.9	-1.5	-1.2
Bremer	IA	-189	-23	-166	-378	-96	25	0	1,055	7	31	-1.7	35.0	-0.6
Otsego	NY	-134	-46	-190	-370	-260	85	10	1,700	36	102	-0.6	11.8	1.3
Kankakee	IL	-58	-145	-167	-370	-197	209	-421	2,477	533	273	-0.4	10.7	-2.9
Meade	SD	-171	-222	27	-366	-123	48	21	1,696	23	67	-1.7	12.4	4.1
Scott	MO	-133	-178	-53	-363	-102	115	335	5,050	463	155	-0.9	55.8	9.6
McKean	PA	-295	22	-90	-363	-461	13	-5	1,376	25	23	-0.8	2.6	-1.1
Wilkin	MN	-208	-224	70	-362	-189	59	16	2,082	18	86	-4.8	138.3	788.1
Tioga	NY	-197	-73	-85	-354	-669	25	3	1,290	15	37	-0.7	6.9	0.9
Lincoln	MT	-237	-82	-31	-351	16	18	1	1,793	1	41	-2.0	9.2	13.0
Scott	IA	13	-188	-168	-343	-649	160	222	3,359	326	279	-0.2	3.8	2.8

TABLE S8-G Apparent Emigration 1990–2020 from 500-Year Floodplain by County, Ranked[1] by Emigration from X500 Zone

	State	Apparent Migration All Races out of …[2]						Apparent Migration[2] 1990 to 2020			Population in 2020, X500 Zone[3]				Migration as % of county population[4]		
		500 Year Floodplain (X500 zone)				A Zone											
County		1990s	2000s	2010s	1990 to 2020	1990 to 2020		Hispanic	Black		All Races	Black	Hispanic		All Races	Black	Hisp.
Washington	OH	-58	87	-366	-337	-1,317		14	13		2,062	24	29		-0.5	6.1	1.7
Buchanan	VA	-192	-80	-56	-328	-1,150		4	4		685	4	10		-1.0	1.5	7.6
Lycoming	PA	-206	-193	77	-322	-1,096		52	215		5,542	265	72		-0.3	8.1	7.7
Santa Cruz	AZ	-129	-174	-19	-321	39		-380	1		1,076	3	836		-1.1	-1.6	1.0
Coles	IL	-205	4	-117	-318	16		6	15		920	23	22		-0.6	1.5	1.6
Schuylkill	PA	10	-153	-170	-313	-1,398		118	17		1,832	19	121		-0.2	17.5	2.1
Ashley	AR	-86	-126	-98	-311	-358		4	-278		140	69	6		-1.3	1.8	-4.2
Delaware	NY	-85	-81	-133	-299	-837		67	5		1,068	11	77		-0.6	12.4	1.1
Richland	ND	-214	-115	31	-298	-44		38	16		1,206	16	52		-1.6	83.4	73.8
Jones	MS	-95	-96	-103	-294	86		23	-287		667	585	23		-0.5	11.0	-1.9
Androscoggin	ME	-119	-94	-76	-289	-124		12	58		532	68	15		-0.3	1.5	12.6
Oneida	NY	-141	-62	-86	-289	-812		33	21		1,495	33	45		-0.1	0.6	0.2
Total, All Races[5]		-89,788	-201,793	-91,556	-235,752			-6,427	-81,688		11,675,209	1,407,975			-0.09	-0.03	-0.003
Black[5]		-36,406	-106,500	-115,891	-172,749												
Black, exclude displacements[6]		-13,147	-87,679	-27,720	-80,043												

1. Ranked by "Apparent Migration All Races out of 500-year floodplain 1990 to 2020.
2. Change in population of the X500 and A zones as mapped by the National Flood Hazard Layer. Includes migration into and out of this land as well as births and deaths. Negative numbers mean emigration out of this county's flood zone. All calculations use the building-based density assumption.
3. The population of the X500 zone.
4. Equal to "Apparent Migration" for 1990 to 2020 as a percent of the population of 1990, with numerator and denominator using the same racial classification.
5. Sum of emigration by counties with net emigration from flood-zone for stated racial classification.
6. Sum of Black emigration or total Emigration, whichever is less, by county. (If Black emigration is greater than total emigration, the difference represents displacement of Black residents by people from other racial groups.

The purpose of this table is to identify counties where the population vulnerable to a 500-year flood is declining.

TABLE S8-H Apparent Emigration 1990–2020 by County from 100-Year Flooplain, Ranked[1] by Total Emigration

County	State	Change 1990–2020 [1]		By Decade						Change 1990–2020					
				Inland Floodplains			Coastal Floodplains			Total Change		% of County Population			
		Inland	Coastal	1990s	2000s	2010s	1990s	2000s	2010s	Black	Hispanic	Total	Black	Hispanic	
Orleans	LA	0	-16,892	0	0	0	2,524	-27,912	8,496	-14,760	-291	-4.4	-4.8	-0.2	
Jefferson	AL	-6,219	0	-2,506	-2,284	-1,429	0	0	0	-4,454	701	-0.9	-2.0	2.6	
Cape May	NJ	0	-6,117	0	0	0	1,526	-6,593	-1,050	-1,151	485	-6.4	-22.0	2.6	
Atlantic	NJ	10	-5,981	129	-56	-63	19	-3,897	-2,103	-104	-729	-2.2	-0.3	-0.5	
Hinds	MS	-5,496	0	-1,882	-743	-2,871	0	0	0	1,139	92	-2.4	0.9	0.8	
St. Bernard	LA	0	-4,252	0	0	0	-358	-5,097	1,203	1,205	-503	-9.7	39.0	-1.2	
Washington	MS	-3,996	0	-504	-2,277	-1,215	0	0	0	564	-40	-8.9	1.4	-1.0	
Cameron	LA	0	-3,773	0	0	0	242	-3,081	-934	-398	-196	-67.2	-79.9	-13.7	
Kanawha	WV	-3,671	0	-833	-890	-1,948	0	0	0	291	142	-2.0	2.1	1.6	
Harrison	MS	198	-3,553	336	-48	-90	617	-6,746	2,576	-413	929	-1.6	-1.3	3.2	
Allegheny	PA	-3,057	0	-1,256	-1,824	23	0	0	0	-212	102	-0.2	-0.1	0.1	
Mobile	AL	-1,181	-1,867	-456	-359	-366	930	-2,622	-176	-143	63	-0.7	-0.1	0.2	
Montgomery	AL	-3,043	0	-790	-1,024	-1,228	0	0	0	-2,301	371	-1.3	-2.6	2.3	
St. Mary	LA	0	-2,928	0	0	0	-1,562	-16	-1,350	-1,222	562	-5.9	-6.7	5.0	
McDowell	WV	-2,915	0	-1,334	-866	-716	0	0	0	-836	-11	-15.3	-17.7	-0.6	
Hampton	VA	423	-2,778	19	377	26	3,904	-7,387	706	1,661	-220	-1.7	3.2	-0.8	
Dougherty	GA	-2,686	0	-1,389	-104	-1,192	0	0	0	-994	-14	-3.1	-2.1	-0.2	
Orange	CA	12,599	-2,674	12,562	1,792	-1,755	-1,622	-2,314	1,262	-755	-31,207	0.3	-1.9	-0.6	
Liberty	GA	-2,507	792	-1,301	-699	-507	222	506	64	-902	-490	-2.6	-4.4	-1.5	
Jefferson	LA	0	-2,434	0	0	0	1,381	-10,368	6,552	10,108	9,786	-0.6	12.9	3.7	
Alexander	IL	-2,392	0	-671	-525	-1,197	0	0	0	-589	-34	-45.7	-16.9	-6.3	
Quitman	MS	-2,367	0	-573	-973	-821	0	0	0	-1,518	-44	-38.3	-24.5	-9.2	
Logan	WV	-2,225	0	-1,181	-201	-844	0	0	0	-209	-44	-6.8	-15.4	-1.6	
Cambria	PA	-2,206	0	-1,209	-625	-373	0	0	0	255	-26	-1.7	6.9	-0.3	
St. Louis	MO	-1,928	0	-764	-1,015	-149	0	0	0	-4,206	184	-0.2	-3.0	0.2	
Jefferson	AR	-1,842	0	-395	-798	-649	0	0	0	-1,022	44	-2.7	-2.8	1.0	
Beaufort	NC	23	-1,841	-7	24	5	-534	-102	-1,205	-1,847	284	-4.1	-14.0	14.4	
Luzerne	PA	-1,822	0	-422	-979	-422	0	0	0	393	752	-0.6	10.4	3.7	
Ohio	WV	-1,795	0	-694	-1,174	73	0	0	0	201	74	-4.2	11.9	5.0	
Lenoir	NC	-1,668	-118	-1,589	-74	-5	-88	34	-64	-1,110	28	-3.2	-4.9	0.6	
Ingham	MI	-1,763	0	-2,354	218	373	0	0	0	95	-205	-0.6	0.3	-0.2	

TABLE S8-H Apparent Emigration 1990–2020 by County from 100-Year Flooplain, Ranked[1] by Total Emigration

County	State	Change 1990–2020 [1]		By Decade						Change 1990–2020				
				Inland Floodplains			Coastal Floodplains			Total Change		% of County Population		
		Inland	Coastal	1990s	2000s	2010s	1990s	2000s	2010s	Black	Hispanic	Total	Black	Hispanic
Leavenworth	KS	-1,727	0	-1,700	-54	27	0	0	0	-669	-114	-2.1	-9.7	-0.5
Wayne	MI	-1,710	0	-2,261	-774	1,324	0	0	0	1,060	806	-0.1	0.1	0.2
Pike	KY	-1,705	0	-710	-341	-654	0	0	0	18	13	-2.9	6.5	0.7
Washington	PA	-1,671	0	-695	-662	-314	0	0	0	2	59	-0.8	0.0	0.5
Harlan	KY	-1,666	0	-354	-810	-502	0	0	0	-22	6	-6.2	-1.8	0.5
Phillips	AR	-1,598	0	-189	-572	-838	0	0	0	-795	-23	-9.6	-5.1	-1.0
St. Landry	LA	-1,576	1,072	160	-882	-853	711	78	283	-248	65	-0.6	-0.8	1.0
Newport News	VA	-76	-1,422	10	-88	3	-602	-831	11	-573	-181	-0.8	-1.0	-0.4
Jackson	NC	-1,491	0	-2,173	460	222	0	0	0	-111	245	-3.0	-60.9	15.8
Acadia	LA	33	-1,470	143	25	-135	106	107	-1,683	16	184	-2.5	0.2	4.9
Forrest	MS	-1,460	0	-701	-572	-187	0	0	0	-1,020	174	-1.9	-4.9	3.5
Bolivar	MS	-1,443	0	-571	-729	-143	0	0	0	-1,128	-10	-4.7	-4.3	-0.3
Sharkey	MS	-1,440	0	-317	-774	-349	0	0	0	-847	-23	-37.9	-18.2	-4.8
Robeson	NC	-1,420	0	101	229	-1,750	0	0	0	-1,168	94	-1.2	-4.5	1.3
St. Charles	MO	-1,409	0	-1,699	74	216	0	0	0	-29	167	-0.3	-0.6	0.7
Leflore	MS	-1,403	0	-163	-587	-653	0	0	0	-522	77	-5.0	-2.3	5.3
Schuylkill	PA	-1,398	0	-518	-340	-540	0	0	0	58	346	-1.0	7.2	5.1
Westmoreland	PA	-1,391	0	-290	-755	-346	0	0	0	-57	34	-0.4	-0.8	0.2
Mingo	WV	-1,372	0	-1,402	265	-236	0	0	0	-34	5	-5.8	-4.1	0.4
Washington	OH	-1,317	0	125	-1,226	-216	0	0	0	-2	-2	-2.2	-0.2	-0.1
Bay	MI	-1,297	0	72	-1,051	-318	0	0	0	26	-140	-1.2	2.1	-0.4
Salem	NJ	13	-1,264	2	7	4	-978	-63	-223	396	554	-1.9	4.2	3.9
Tallahatchie	MS	-1,236	0	-272	-101	-863	0	0	0	-950	-27	-9.7	-10.8	-3.3
El Paso	TX	-1,227	0	-408	-240	-579	0	0	0	-23	-6,203	-0.1	-0.1	-0.2
Warren	MS	-1,200	0	-506	-235	-458	0	0	0	-807	2	-2.7	-4.3	0.1
Brooks	TX	-1,194	0	-499	-415	-281	0	0	0	5	-5,187	-16.9	254.7	-7.1
Edgecombe	NC	-1,109	-75	-1,000	405	-514	-71	6	-10	-1,087	149	-2.4	-3.4	5.8
Fayette	PA	-1,173	0	-350	-558	-265	0	0	0	8	20	-0.9	0.2	0.4
Los Angeles	CA	1,546	-1,172	867	1,031	-353	-737	-424	-11	-2,958	-17,945	0.0	-0.3	-0.1
Jackson	AR	-1,166	0	-569	-248	-349	0	0	0	-121	44	-7.0	-4.4	6.7
Somerset	MD	0	-1,163	0	0	0	-282	-429	-451	-620	175	-4.7	-7.0	7.6
Will	IL	-1,158	0	531	-458	-1,230	0	0	0	-1,992	-3,305	-0.2	-5.3	-1.7
Buchanan	VA	-1,150	0	-614	-95	-441	0	0	0	11	-8	-5.7	20.0	-0.3

TABLE S8-H Apparent Emigration 1990–2020 by County from 100-Year Flooplain, Ranked[1] by Total Emigration

County	State	Change 1990–2020 [1]		By Decade						Change 1990–2020				
				Inland Floodplains			Coastal Floodplains			Total Change		% of County Population		
		Inland	Coastal	1990s	2000s	2010s	1990s	2000s	2010s	Black	Hispanic	Total	Black	Hispanic
Wyoming	WV	-1,143	0	-590	-350	-203	0	0	0	-22	-10	-5.3	-9.2	-1.2
Wetzel	WV	-1,127	0	-302	-326	-499	0	0	0	6	27	-7.8	34.8	7.3
Athens	OH	-1,110	0	-4,134	4,227	-1,203	0	0	0	539	178	-1.8	32.6	4.1
Lycoming	PA	-1,096	0	-299	-457	-340	0	0	0	30	48	-1.0	1.1	0.7
Raleigh	WV	-1,092	0	-297	-307	-488	0	0	0	-118	29	-1.5	-2.0	0.9
Black Hawk	IA	-1,089	0	-663	-422	-3	0	0	0	28	24	-0.8	0.3	0.3
Dallas	AL	-1,088	0	138	-618	-608	0	0	0	-529	-24	-2.8	-1.9	-1.9
Boone	WV	-1,083	0	-414	-224	-445	0	0	0	-41	5	-5.0	-19.0	1.0
Rock Island	IL	-1,081	0	-387	-542	-152	0	0	0	161	31	-0.7	1.6	0.0
Jefferson	OH	-1,078	0	-495	-352	-230	0	0	0	27	26	-1.7	0.6	0.6
Fayette	WV	-1,061	0	-199	-387	-475	0	0	0	-204	-19	-2.6	-6.8	-0.8
Geary	KS	-1,038	0	-635	102	-506	0	0	0	-309	-47	-2.8	-4.4	-0.3
Belmont	OH	-1,032	0	-295	-603	-134	0	0	0	5	2	-1.6	0.4	0.1
Richmond	GA	-1,030	0	-452	-325	-253	0	0	0	-397	24	-0.5	-0.5	0.1
Lowndes	MS	-1,027	0	38	-872	-193	0	0	0	547	22	-1.7	2.5	0.5
Hale	TX	-1,011	0	-236	-395	-380	0	0	0	-324	-2,450	-3.1	-18.3	-1.7
Floyd	KY	-1,009	0	-91	-208	-711	0	0	0	-35	1	-2.8	-11.4	0.1
Arkansas	AR	-1,008	0	-11	-577	-419	0	0	0	-299	12	-5.9	-6.3	2.0
Linn	IA	-1,004	0	-19	-1,646	661	0	0	0	136	27	-0.4	4.1	0.2
Pennington	SD	-988	0	-896	3	-95	0	0	0	-158	-30	-0.9	-12.6	-0.2
St. Clair	MI	-983	0	495	-894	-584	0	0	0	31	-72	-0.6	1.1	-0.3
Gallia	OH	-970	0	-494	-305	-171	0	0	0	-31	-34	-3.3	-3.6	-2.2
Bell	KY	-960	0	-287	-219	-454	0	0	0	-35	27	-4.0	-4.4	3.6
Mineral	NV	-942	0	-691	-149	-101	0	0	0	-106	-151	-20.7	-31.0	-2.8
Honolulu	HI	-925	16,771	-412	-196	-317	-659	15,266	2,165	193	-266	1.6	0.8	0.0
Butler	MO	-902	0	-276	-119	-507	0	0	0	-237	22	-2.1	-12.0	1.0
Clay	AR	-902	0	-220	-318	-364	0	0	0	7	7	-6.2	148.5	1.1
Ouachita	LA	-890	0	-912	9	12	0	0	0	2,308	232	-0.6	5.2	1.9
Lander	NV	-868	0	-592	-169	-108	0	0	0	4	-409	-15.1	44.4	-5.2
Accomack	VA	0	-862	0	0	0	663	-1,507	-17	-237	120	-2.6	-2.2	2.6
Gloucester	NJ	808	-860	-1	96	713	-617	-152	-92	58	229	0.0	0.3	0.6
Delaware	NY	-837	0	-169	-193	-475	0	0	0	12	62	-1.9	2.7	1.2
Miller	AR	-835	0	-466	-211	-158	0	0	0	-463	28	-2.0	-5.4	0.9

TABLE S8-H Apparent Emigration 1990–2020 by County from 100-Year Flooplain, Ranked[1] by Total Emigration

		Change 1990–2020 [1]		By Decade						Change 1990–2020				
				Inland Floodplains			Coastal Floodplains			Total Change		% of County Population		
County	State	Inland	Coastal	1990s	2000s	2010s	1990s	2000s	2010s	Black	Hispanic	Total	Black	Hispanic
Dyer	TN	-815	0	-43	-237	-535	0	0	0	-12	11	-2.2	-0.3	0.8
Oneida	NY	-812	0	-520	-75	-217	0	0	0	-322	19	-0.3	-2.6	0.0
Hancock	OH	-795	0	-99	-435	-261	0	0	0	15	-130	-1.1	2.7	-0.8
Walsh	ND	-786	0	-302	-365	-119	0	0	0	31	157	-7.4	182.9	3.6
Gibson	TN	-782	0	-258	-432	-92	0	0	0	-839	12	-1.5	-9.4	0.7
Desha	AR	-777	0	-177	-346	-254	0	0	0	-117	26	-6.8	-1.6	1.7
Onondaga	NY	-771	0	-322	-456	7	0	0	0	-389	93	-0.2	-1.1	0.1
Greene	OH	-752	0	-252	-502	2	0	0	0	-135	-2	-0.4	-1.4	0.0
Newport	RI	15	-745	10	-3	7	-111	-567	-66	-70	73	-0.9	-2.2	0.4
Madison	LA	-742	0	214	-605	-352	0	0	0	-423	-16	-7.4	-5.7	-1.2
Yazoo	MS	-728	0	1,509	-1,651	-587	0	0	0	-143	-962	-2.7	-1.1	-93.4
Camden	NJ	409	-719	219	58	132	157	-933	57	-1,019	-1,365	-0.1	-1.3	-0.4
Evangeline	LA	-700	-18	-135	-275	-290	86	-95	-9	112	40	-2.2	1.3	1.5
Monroe	MI	-717	0	227	-529	-415	0	0	0	169	-6	-0.5	7.3	0.0
Jackson	IL	-717	0	-318	123	-522	0	0	0	20	-3	-1.4	0.3	0.0
Hyde	NC	0	-710	0	0	0	274	69	-1,053	-549	182	-15.5	-30.9	42.2
Portsmouth	VA	0	-709	0	0	0	-833	-1,025	1,149	-343	320	-0.7	-0.7	2.3
Marshall	WV	-697	0	-237	-134	-325	0	0	0	30	-10	-2.3	14.8	-0.4
Clinton	PA	-696	0	-332	-277	-87	0	0	0	21	42	-1.9	14.6	4.6
Butler	OH	-670	0	-523	-187	40	0	0	0	-8	263	-0.2	-0.1	1.8
Tioga	NY	-669	0	-183	-103	-383	0	0	0	15	-7	-1.4	5.0	-0.2
Ottawa	OK	-663	0	-150	-425	-88	0	0	0	-8	15	-2.2	-4.3	0.4
Humphreys	MS	-654	0	-260	-192	-202	0	0	0	-420	-16	-8.4	-5.1	-2.3
Scott	IA	-649	0	-242	-597	190	0	0	0	202	71	-0.4	2.6	0.2
San Joaquin	CA	-379	-267	-506	105	22	536	-1,689	886	-202	-7,389	-0.1	-0.8	-0.7
Bucks	PA	-546	-99	-330	-500	284	-108	-187	196	92	276	-0.1	0.6	0.3
Philadelphia	PA	547	-622	-120	299	369	-2,137	1,584	-69	-329	120	0.0	-0.1	0.0
Armstrong	PA	-620	0	-185	-326	-109	0	0	0	-59	5	-0.9	-10.4	0.3
Marion	SC	-620	29	-94	-157	-369	61	7	-40	-543	-3	-2.0	-2.9	-0.3
Sutter	CA	-578	-41	-328	101	-351	-26	-96	81	13	-961	-0.6	1.3	-0.9
Iberville	LA	0	-611	0	0	0	938	328	-1,876	-387	-82	-2.0	-2.7	-1.4
Dorchester	MD	14	-605	-1	3	13	15	-372	-248	-57	24	-1.8	-0.7	1.4
Schoharie	NY	-599	0	-356	159	-402	0	0	0	-1	-10	-2.0	-0.3	-0.2

TABLE S8-H Apparent Emigration 1990–2020 by County from 100-Year Flooplain, Ranked[1] by Total Emigration

County	State	Change 1990–2020 [1]		By Decade						Change 1990–2020				
				Inland Floodplains			Coastal Floodplains			Total Change		% of County Population		
		Inland	Coastal	1990s	2000s	2010s	1990s	2000s	2010s	Black	Hispanic	Total	Black	Hispanic
Holmes	MS	-599	0	-217	-250	-132	0	0	0	-419	-12	-3.5	-2.6	-1.9
Chenango	NY	-589	0	-197	-224	-167	0	0	0	35	34	-1.2	10.0	0.7
Sunflower	MS	-587	0	61	-389	-260	0	0	0	-400	-91	-2.3	-1.9	-5.0
Scioto	OH	-585	0	-311	-124	-150	0	0	0	-139	-5	-0.8	-5.7	-0.2
Iroquois	IL	-581	0	182	-319	-444	0	0	0	20	30	-2.1	12.1	0.5
Nicholas	WV	-570	0	-182	-205	-183	0	0	0	1	4	-2.3	26.6	0.7
Tyrrell	NC	0	-540	0	0	0	264	241	-1,045	-527	105	-16.6	-34.2	95.5
Middlesex	NJ	992	-538	385	419	188	22	58	-618	247	585	0.1	0.5	0.1
Montgomery	NY	-535	0	-331	34	-238	0	0	0	-10	-11	-1.1	-2.8	0.0
Grenada	MS	-529	0	-98	-305	-127	0	0	0	-289	-9	-2.4	-3.3	-1.0
Meigs	OH	-526	0	-193	-15	-318	0	0	0	0	2	-2.4	0.2	0.3
Clermont	OH	-524	0	-628	-202	307	0	0	0	-37	67	-0.3	-2.9	0.9
Lawrence	IL	-521	0	-131	-249	-141	0	0	0	-4	-18	-3.4	-3.0	-3.2
Pembina	ND	-520	0	-94	-271	-155	0	0	0	4	-33	-7.6	27.7	-3.8
Grays Harbor	WA	237	-517	140	-59	157	-228	-304	15	93	709	-0.4	80.2	6.0
Harrison	WV	-517	0	-143	-42	-331	0	0	0	-29	11	-0.8	-3.0	0.1
Northumberland	PA	-509	0	-120	-169	-220	0	0	0	24	115	-0.6	7.7	2.2
Gallatin	IL	-505	0	-131	-218	-155	0	0	0	-2	-4	-10.2	-4.8	-2.3
**Total[5]**		-243,390	-77,991	-117,75	-145,83	-130,23	-19,779	-136,056	-28,597	-73,570[6]	-3,490[6]			

1. Ranked by the sum of Change 1990-2020 Inland and Coastal 100-year floodplains (A zone).
2. Change of the A zones as mapped by the National Flood Hazard Layer. All calculations use the building-based density assumption.
3. Census blocks with land in the A-zone are deemed as coastal if they have land below the highest storm-surge base level in a given state.
4. Numerator and denominator use the same racial classification.
5. Sum of emigration by counties with net emigration from flood-zone for stated racial classification.
6. Sum of Black (Hispanic) emigration or total emigration, whichever is less, by county. (If a minority emigration is greater than total emigration, the difference represents displacement of that minority by people from other racial groups.

End

The purpose of this table is to rank the counties where the population vulnerable to flooding is declining.

TABLE S8-J Apparent Emigration 1990–2020 from Floodplains and Lands Close to Sea Level										
	State	Apparent Migration All Races[2]					Apparent Migration, Black[3]			
County or Class of Hazard Zone		1990 to 2020	1990s	2000s	2010s		1990 to 2020	1990s	2000s	2010s
Inland 100-year floodplain[1]		-243,390	-117,775	-145,833	-130,283		-47,047	-20,130	-27,940	-32,030
500-year floodplain		-235,752	-89,788	-201,793	-91,556		-80,043	-13,147	-87,679	-27,720
Land below 3m		-225,584	-63,714	-301,171	-63,151		-118,795	-9,065	-133,632	-21,802
Less: 500-year floodplains possibly below 3m[4]		-96,078	-17,994	-115,635	-6,825		-49,365	-695	-67,961	-1,374
Less: below 3m possibly in 500-year floodplain[5]		-174	-67	-49	-204		-69	-21	-66	-25
**Total Apparent Emigration**		**-608,473**	**-253,217**	**-533,112**	**-277,961**		**-196,451**	**-41,626**	**-181,224**	**-80,153**
500-year floodplains possibly below 3m[4]										
Orleans	LA	-62,445	-9,107	-81,532			-47,140		-67,032	
St. Bernard	LA	-17,647		-25,048						
St. Mary	LA	-3,935	-2,516		-3,046					
Mobile	AL	-1,757	-251	-1,274	-232				-55	
Hampton	VA	-1,448		-2,353	-618					
Camden	NJ	-1,127	-1,438		-123		-867	-152	-333	-382
Salem	NJ	-821	-645	-259						
New London	CT	-821	-679		-297		-829	-421	-216	-89
Portsmouth	VA	-706	-1,649							
Newport	RI	-685	-611		-196				-29	-336
Cape May	NJ	-615		-458	-337					-161
Baltimore	MD	-546	-387	-319						-3
Kent	RI	-524		-584	-68		-105	-79	-54	
Atlantic	NJ	-453		-776	-254		-214		-110	-125
Jefferson	LA	-411		-1,365						-105
Jefferson Davis	LA	-402	-194	-78	-130		-8		-21	-3
Bucks	PA	-224	-78	-363						

TABLE S8-J Apparent Emigration 1990–2020 from Floodplains and Lands Close to Sea Level										
	State	Apparent Migration All Races[2]					Apparent Migration, Black[3]			
County or Class of Hazard Zone		1990 to 2020	1990s	2000s	2010s		1990 to 2020	1990s	2000s	2010s
Washington	NC	-224	-5	-102	-116					
Washington	RI	-205		-418			-82	-12	-53	
Somerset	MD	-161	-11	-56	-94					
Gloucester	NJ	-160	-213		-87			-30		-117
Tyrrell	NC	-125	-59	-8	-59		-22		-55	-41
Accomack	VA	-104		-222						0
Bertie	NC	-104	-84		-92				-1	
Northampton	VA	-94	-33	-81			-95			-8
Pamlico	NC	-81		-72	-109					-1
Talbot	MD	-62		-53	-58				-1	
Vermilion	LA	-54		-174	-649			-1		
Greene	NY	-33			-121					
Mathews	VA	-33			-88		0	0		
Lancaster	VA	-29		-22	-27					
Rensselaer	NY	-28	-15		-15					-1
Kent	MD	-5		-18			-3		-1	-1
Surry	VA	-4	-3	-1	0					
Hanover	VA	-2	-17						0	0
Hyde	NC	-2			-11					
Total		-96,078	-17,994	-115,635	-6,825		-49,365	-695	-67,961	-1,374
Land below 3m possibly in 500-year floodplain[5]										
Acadia	LA	-127	-40		-178		-32	-17	-23	
Hertford	NC	-26		-38	-2		-17		-38	-7
Jones	NC	-4			-9		-13	-1	-4	-8
Lenoir	NC	-1	-1		0				0	0
Kleberg	TX	-2	-22	-10					0	
Newton	TX	-2		-1	-4					
Franklin	VA	-11	-5		-11		-7	-3		-10

TABLE S8-J Apparent Emigration 1990–2020 from Floodplains and Lands Close to Sea Level

	State	Apparent Migration All Races[2]					Apparent Migration, Black[3]			
County or Class of Hazard Zone		1990 to 2020	1990s	2000s	2010s		1990 to 2020	1990s	2000s	2010s
Total		-174	-67	-49	-204	0	-69	-21	-66	-25

1. Change in population of the A zones as mapped by the National Flood Hazard Layer. For coastal states, these calculations only include Census blocks that are entirely above the highest storm-surge base flood elevation in the state. Includes migration into and out of this land as well as births and deaths. Negative numbers mean emigration out of this county's inland A zone.
2. Sum of emigration by counties with net emigration from flood-zone for stated racial classification.
3. Sum of Black emigration or total Emigration, whichever is less, by county. (If Black emigration is greater than total emigration, the difference represents displacement of Black residents by people from other racial groups.
4. Apparent migration from X-zone for counties in which apparent migration from the <3m is greater than from X-zone. In effect, to avoid any chance of double-counting, we assume that emigration from <3m and from the X500-zone should not both be added to the table. Because not all (and probably not most) emigration from X-zone represents emigration from the <3m, this assumption results in an undercount.
5. Apparent migration from land <3m for counties in which apparent migration from the X500-zone is greater than from land<3m. In effect, to avoid any chance of double-counting, we assume that emigration from <3m and from the X500-zone should not both be added to the table. Because not (and probably not most) emigration from <3m represents emigration from the X500-zone, this assumption results in an undercount.

The purpose of this table is to tally the total apparent emigration from all counties where population is declining in either floodplains or land vulnerable to sea level rise.

Begin

Table S9-A: Summary Statistics Measuring whether Black Residents Account for a Disproportionate Share of People Inhabiting Low Land Who Emigrate from Low Land, in Counties with Net Emigration from Low Land, by Decade										
Population Migrating Out of Low Land[1]						Ratios				
	1990s	2000s	2010s	1990 to 2020		1990s	2000s	2010s	1990 to 2020	
Land Below 1 meter										
All Races	-24,887	-226,444	-29,611	-169,312		0.353	1.114	1.425	1.283	County-Adjusted Disproportionality[3]
Black	-3,829	-117,097	-6,590	-98,354		0.682	2.178	1.086	2.575	Nationwide Disproportionality[4]
Black (proportional)[2]	-10,851	-105,081	-4,626	-76,689		0.226	0.237	0.205	0.226	Black share[5] of population <1m
						0.154	0.517	0.223	0.581	Black share of Migration[6]
Land Below 1 meter (excluding New Orleans)[7]										
All Races	-13,526	-95,638	-29,611	-68,333		1.097	0.760	1.425	1.349	County-Adjusted Disproportionality[3]
Black	-3,829	-9,514	-6,590	-15,196		2.441	0.769	1.630	1.918	Nationwide Disproportionality[4]
Black (proportional)	-3,490	-12,512	-4,626	-11,264		0.116	0.129	0.137	0.116	Black share[5] of population <1m
						0.283	0.099	0.223	0.222	Black share of Migration[6]
Land Below 3 meters										
All Races	-63,714	-301,171	-63,151	-225,584		0.453	1.175	1.701	1.361	County-Adjusted Disproportionality[3]
Black	-9,065	-133,632	-21,802	-118,795		0.847	2.480	1.970	3.134	Nationwide Disproportionality[4]
Black (proportional)	-19,990	-113,749	-12,820	-87,289		0.168	0.179	0.175	0.168	Black share[5] of population <3m
						0.142	0.444	0.345	0.527	Black share of Migration[6]
Land Below 3 meters (Excluding New Orleans) [7]										
All Races	-47,766	-165,844	-63,151	-115,569		0.888	0.839	1.701	1.207	County-Adjusted Disproportionality[3]
Black	-9,065	-19,486	-21,802	-23,872		1.311	0.748	2.121	1.427	Nationwide Disproportionality[4]
Black (proportional)	-10,204	-23,229	-12,820	-19,777		0.145	0.157	0.163	0.145	Black share[5] of population <3m
						0.190	0.117	0.345	0.207	Black share of Migration[6]

1. National Total of Counties with Net Emigration from land below the specified elevation (See Tables S-8A to Tables S-8D.)
2. The sum across counties with emigration, of the product of the Black share of the county population in hazard zone, times emigration from all races in the county; that is, the number of Black residents that would have emigrated from the vulnerable zone if Black emigration was proportional to the emigration from all races. This is analogous to the denominator in note 2 of Table S7, except that "X" refers to those inhabiting the hazard zone, while "F" refers to those who emigrate.
3. County-Adjusted Ratio of Disproportionality, see Table S7 of note 2 for the formula, except that in this case, "X" refers to those inhabiting the hazard zone, while "F" refers to apparent emigration.
4. Ratio of Black share of Migration to Black share of population in hazard zone.
5. Using population at the beginning of the period.
6. Ratio of "Black" to "All Races" for the corresponding period of time.
7. Excluding New Orleans does not change totals for 2010 because low land in New Orleans had net immigration in the 2010s.

The purpose of this table is estimate the extent to which Black residents of land vulnerable to sea level rise are leaving those lands at a rate greater than people of all races, at both the national and county scale.

Table S9-B: Summary Statistics Measuring whether Black Residents Account for a Disproportionate Share of People Inhabiting Floodplains Who Emigrate from Floodplains, in Counties with Net Emigration from Floodplains, by Decade

Population Migrating Out of Low Land[1]						Ratios				
	1990s	2000s	2010s	1990 to 2020		1990s	2000s	2010s	1990 to 2020	
100-Year Coastal Floodplain (A Zone)										
All Races	-19,779	-136,056	-28,597	-77,991		0.800	1.050	1.863	1.320	County-Adjusted Disproportionality[3]
Black	-3,196	-35,005	-9,301	-26,296		1.330	2.030	2.660	2.774	Nationwide Disproportionality[4]
Black (proportional)[2]	-3,996	-33,340	-4,993	-19,925		0.122	0.127	0.122	0.122	Black share[5] of population in Floodplain
						0.162	0.257	0.325	0.337	Black share of Migration[6]
100-year Inland Floodplain (A Zone)										
All Races	-117,775	-145,833	-130,283	-243,390		0.993	1.112	1.276	0.978	County-Adjusted Disproportionality[3]
Black	-20,130	-27,940	-32,030	-47,047		1.511	1.646	2.090	1.709	Nationwide Disproportionality[4]
Black (proportional)[2]	-20,269	-25,119	-25,108	-48,128		0.113	0.116	0.118	0.113	Black share[5] of population in Floodplain
						0.171	0.192	0.246	0.193	Black share of Migration[6]
500-year Floodplain (X500 Zone)										
All Races	-89,788	-201,793	-91,556	-235,752		0.555	1.079	1.178	1.008	County-Adjusted Disproportionality[3]
Black	-13,147	-87,679	-27,720	-80,043		1.214	3.609	2.559	2.815	Nationwide Disproportionality[4]
Black (proportional)[2]	-23,707	-81,270	-23,539	-79,370		0.121	0.120	0.118	0.121	Black share[5] of population in Floodplain
						0.146	0.435	0.303	0.340	Black share of Migration[6]
500-year Floodplain (excluding New Orleans)										
All Races	-80,682	-120,261	-91,556	-173,306		0.750	1.030	1.178	0.889	County-Adjusted Disproportionality[3]
Black	-13,147	-20,647	-27,720	-32,904		1.506	1.574	2.705	1.755	Nationwide Disproportionality[4]
Black (proportional)[2]	-17,529	-20,050	-23,539	-37,009		0.108	0.109	0.112	0.108	Black share[5] of population in Floodplain
						0.163	0.172	0.303	0.190	Black share of Migration[6]
100-year and 500-year Floodplains										
All Races	-227,343	-483,681	-250,436	-557,132		0.760	1.078	1.287	1.040	County-Adjusted Disproportionality[3]
Black	-36,472	-150,623	-69,051	-153,386		1.347	2.581	2.320	2.687	Nationwide Disproportionality[4]
Black (proportional)[2]	-47,972	-139,729	-53,641	-147,424		0.119	0.121	0.119	0.119	Black share[5] of population in Floodplain
						0.160	0.311	0.276	0.320	Black share of Migration[6]

1. National Total of Counties with Net Emigration from land below the specified floodplain (See Tables S-8E to Tables S-8H.)
2. Sum across counties with emigration, of the product of the Black share of the county population in hazard zone, times emigration from all races in the county; i.e, the number of Black residents that would have emigrated from vulnerable zone if Black emigration was proportional to the emigration from all races. This is analogous to the denominator in note 2 of Table S7, except that "X" refers to those inhabiting the hazard zone, while "F" refers to those who emigrate.
3. County-Adjusted Ratio of Disproportionality. See note 3, Table S9-A for more details.
4. Ratio of Black share of migration to Black share of population in hazard zone.
5. Using population at the beginning of the period.
6. Ratio of "Black" to "All Races" for the corresponding period of time.

Table S9-C: Summary Statistics Measuring whether Black Residents Account for a Disproportionate Share of Emigration from Low Land in Counties with Net Emigration, by Decade

Population Migrating Out of Low Land[1]						Ratios				
	1990s	2000s	2010s	1990 to 2020		1990s	2000s	2010s	1990 to 2020	
Land Below 1 meter										
All Races	-24,887	-226,444	-29,611	-169,312		0.382	1.158	1.329	1.355	County-Adjusted Disproportionality[3]
Black	-3,829	-117,097	-6,590	-98,354		1.310	4.286	1.845	4.945	Nationwide Disproportionality[4]
Black (proportional)[2]	-10,019	-101,116	-4,960	-72,599		0.117	0.121	0.121	0.117	Black share[5] of population
						0.154	0.517	0.223	0.581	Black share of Migration[6]
Land Below 1 meter (excluding New Orleans)[7]										
All Races	-13,526	-95,638	-29,611	-68,333		1.257	0.688	1.329	1.432	County-Adjusted Disproportionality[3]
Black	-3,829	-9,514	-6,590	-15,196		2.430	0.831	1.853	1.909	Nationwide Disproportionality[4]
Black (proportional)	-3,046	-13,838	-4,960	-10,613		0.116	0.120	0.120	0.116	Black share[5] of population
						0.283	0.099	0.223	0.222	Black share of Migration[6]
Land Below 3 meters										
All Races	-63,714	-301,171	-63,151	-225,584		0.458	1.167	1.535	1.361	County-Adjusted Disproportionality[3]
Black	-9,065	-133,632	-21,802	-118,795		1.211	3.678	2.862	4.483	Nationwide Disproportionality[4]
Black (proportional)	-19,793	-114,484	-14,201	-87,309		0.117	0.121	0.121	0.117	Black share[5] of population
						0.142	0.444	0.345	0.527	Black share of Migration[6]
Land Below 3 meters (Excluding New Orleans) [7]										
All Races	-47,766	-165,844	-63,151	-115,569		0.906	0.806	1.535	1.207	County-Adjusted Disproportionality[3]
Black	-9,065	-19,486	-21,802	-23,872		1.629	0.982	2.875	1.773	Nationwide Disproportionality[4]
Black (proportional)	-10,003	-23,229	-12,820	-19,777		0.116	0.120	0.120	0.116	Black share[5] of population
						0.190	0.117	0.345	0.207	Black share of Migration[6]

1. National Total of Counties with Net Emigration from land below the specified elevation (See Tables S-8A to Tables S-8D.)
2. The sum across counties with emigration, of the product of the Black share of the county population, times emigration from all races in the county; that is, the number of Black residents that would have emigrated from the vulnerable zone if Black emigration was proportional to the emigration from all races. This is analogous to the denominator in note 2 of Table S7. This is analogous to the denominator in note 2 of Table S7, except that "F" refers to those who emigrate.
3. County-Adjusted Ratio of Disproportionality. See Table S7 of note 2 for the formula, except that in this case, "F" refers to apparent emigration.
4. Ratio of Black share of migration to Black share of population.
5. Using population at the beginning of the period.
6. Ratio of "Black" to "All Races" for the corresponding period of time.
7. Excluding New Orleans does not change totals for 2010 because low land in New Orleans had net immigration in the 2010s.

Table S9-D: Summary Statistics Measuring whether Black Residents Account for a Disproportionate Share of Emigration from Floodplains in Counties with Net Emigration by Decade

Population Migrating Out of Low Land[1]						Ratios				
	1990s	2000s	2010s	1990 to 2020		1990s	2000s	2010s	1990 to 2020	
100-Year Coastal Floodplain (A Zone)										
All Races	-19,779	-136,056	-28,597	-77,991		0.856	1.002	1.452	1.242	County-Adjusted Disproportionality[3]
Black	-3,196	-35,005	-9,301	-26,296		1.375	2.133	2.697	2.870	Nationwide Disproportionality[4]
Black (proportional)[2]	-3,736	-34,925	-6,404	-21,166		0.117	0.121	0.121	0.117	Black share[5] of population
						0.162	0.257	0.325	0.337	Black share of Migration[6]
100-year Inland Floodplain (A Zone)										
All Races	-117,775	-145,833	-130,283	-243,390		1.184	1.233	1.403	1.173	County-Adjusted Disproportionality[3]
Black	-20,130	-27,940	-32,030	-47,047		1.455	1.588	2.038	1.645	Nationwide Disproportionality[4]
Black (proportional)[2]	-17,003	-22,660	-22,835	-40,121		0.117	0.121	0.121	0.117	Black share[5] of population
						0.171	0.192	0.246	0.193	Black share of Migration[6]
500-year Floodplain (X500 Zone)										
All Races	-89,788	-201,793	-91,556	-235,752		0.648	1.207	1.311	1.127	County-Adjusted Disproportionality[3]
Black	-13,147	-87,679	-27,720	-80,043		1.246	3.602	2.510	2.890	Nationwide Disproportionality[4]
Black (proportional)[2]	-20,295	-72,668	-21,152	-71,015		0.117	0.121	0.121	0.117	Black share[5] of population
						0.146	0.435	0.303	0.340	Black share of Migration[6]
500-year Floodplain (excluding New Orleans)										
All Races	-80,682	-120,261	-91,556	-173,306		0.894	1.130	1.311	1.007	County-Adjusted Disproportionality[3]
Black	-13,147	-20,647	-27,720	-32,904		1.399	1.434	2.521	1.630	Nationwide Disproportionality[4]
Black (proportional)[2]	-14,704	-18,267	-21,152	-32,683		0.116	0.120	0.120	0.116	Black share[5] of population
						0.160	0.311	0.276	0.320	Black share of Migration[6]
100-year and 500-year Floodplains										
All Races	-227,343	-483,681	-250,436	-557,132		0.889	1.156	1.370	1.159	County-Adjusted Disproportionality[3]
Black	-36,472	-150,623	-69,051	-153,386		1.366	2.581	2.286	2.725	Nationwide Disproportionality[4]
Black (proportional)[2]	-41,033	-130,252	-50,391	-132,302		0.117	0.121	0.121	0.117	Black share[5] of population
						0.117	0.121	0.121	0.117	Black share of Migration[6]

1. National Total of Counties with Net Emigration from land below the specified elevation (See Tables S-8A to Tables S-8D.)
2. Sum across counties with emigration, of the product of the Black share of the county population in hazard zone, times emigration from all races in the county; i.e, the number of Black residents that would have emigrated from vulnerable zone if Black emigration was proportional to the emigration from all races. Analogous to the denominator in note 2 of Table S7. This is analogous to the denominator in note 2 of Table S7, except that “F” refers to those who emigrate.
3. See Table S7 of note 2 for the formula, except that in this case, “F” refers to apparent emigration.
4. Ratio of Black share of migration to Black share of population in hazard zone.
5. Using population at the beginning of the period.
6. Ratio of “Black” to “All Races” for the corresponding period of time.

TABLE 8A(i) Counties where Apparent Emigration[1] from Land Below 1 meter was at least 1% of County Population

County and State		1990 to 2020	1990s	2000s	2010s		% County[2] Total	Black
Cameron	LA	-2,656	-61	-2,255	-340		-28.7	-42.9
St. Bernard	LA	-18,356	319	-25,638	6,963		-27.5	249.2
Hyde	NC	-1,117	-170	-238	-708		-20.6	-43.6
Orleans	LA	-100,979	-11,361	-130,806	41,188		-20.3	-27.3
Tyrrell	NC	-560	257	257	-1,073		-14.5	-34.9
Plaquemines	LA	-2,040	1,076	-3,690	574		-8.0	-15.8
St. Mary	LA	-2,713	-1,997	1,172	-1,889		-4.7	-3.7
Somerset	MD	-1,028	-213	-419	-395		-4.4	-3.9
Pamlico	NC	-408	-129	-109	-170		-3.6	-3.1
Cape May	NJ	-2,819	840	-3,025	-634		-3.0	-15.3
Beaufort	NC	-1,136	-502	-193	-441		-2.7	-7.5
Jefferson	TX	-6,393	-2,225	-3,182	-986		-2.7	-10.2
Accomack	VA	-703	455	-1,137	-20		-2.2	0.1
Dorchester	MD	-642	-3	-335	-305		-2.1	-1.1
Jefferson	LA	-8,454	5,644	-21,027	6,929		-1.9	43.3
Salem	NJ	-1,220	-631	-548	-41		-1.9	0.6
Vermilion	LA	-933	-174	-501	-258		-1.9	0.5
Mathews	VA	-141	173	-227	-87		-1.7	0.4
Dixie	FL	-171	-53	-61	-58		-1.6	0.2
Gloucester	VA	-416	-147	-267	-1		-1.4	-0.4
Terrebonne	LA	-1,024	-241	1,991	-2,773		-1.1	7.1
Total USA		-169,312	-24,887	-226,444	-29,611		-0.07	-0.37

1. Change in population of land below or less than one meter above the sea level of 2020. Includes migration into and out of this land as well as births and deaths. Calculations use building-based density assumption.
2. Equal to “Apparent Migration” for 1990 to 2020 as a percent of total and Black population of 1990, respectively.

TABLE S11-A: Impact on Black Population below One Meter by County from the Implicit Racial Segregation Captured By HOLC Mapping, Ranked by Impact in 2020

County	State	2020 Population of HOLC Mapped Areas[1]		By how much does the Black population below 1m exceed what it would be without racial segregation?[2]						Percent of 2020 Population Below 1m [3]		Percent Population In 2020 that was Black [4]			
				---2020---					1990					Below 1m [5]	
		Black	Total	All Zones	Red	Yellow	Blue	Green	All Zones	Red Zone	Other Zones	Red Zone	Other Zones	Red Zone	Other Zones
Orleans	LA	76,990	158,924	902	12,628	-4,847	-4,867	-2,012	1862	76.5	85.0	58.4	29.4	67.9	32.8
Atlantic	NJ	4,444	16,525	456	1,366	-244	-652	-15	767	28.8	21.9	52.9	17.4	63.7	15.3
Middlesex	MA	1,078	8,774	369	359	17	-6	-1	522	5.34	0.66	11.3	5.6	15.5	6.5
Queens	NY	7,477	18,630	184	128	149	-93	0	165	1.22	0.94	20.7	15.2	15.6	46.3
Jefferson	TX	6,725	12,252	79	9	98	-755	727	53	0.69	28.3	72.5	45.6	73.0	54.8
Kings	NY	2,586	11,264	73	362	-288	0	0	43	0.77	0.24	30.0	22.5	31.8	4.2
Hillsborough	FL	263	2,446	34	94	0	-31	-28	-8	3.05	1.32	39.6	12.9	15.7	1.9
New York	NY	404	3,827	34	82	0	-37	-11	26	0.38	0.14	14.8	7.0	12.3	3.6
Fairfield	CT	285	2,481	33	11	32	0	-9	170	1.42	2.29	26.3	10.2	10.1	11.6
[6]															
Nassau	NY	21	210	-27	-27	0	0	0	0	53.3	0.00	5.6	36.2	10.0	n/a
Philadelphia	PA	854	1,141	-28	-28	0	0	0	82	0.21	0.00	35.8	47.0	76.3	2.8
Hampton	VA	99	370	-29	5	8	0	-42	-26	0.86	3.30	78.1	42.5	71.0	24.7
Baltimore	MD	8	146	-33	-33	0	0	0	0	0.12	0.00	44.6	63.3	5.7	n/a
Portsmouth	VA	376	1,025	-49	42	-87	-3	-1	-98	2.59	3.36	77.1	45.3	51.1	30.3
Hudson	NJ	570	18,482	-150	-151	1	0	0	127	7.31	0.10	9.9	11.3	3.1	2.5
Jefferson	LA	2,567	18,930	-187	920	-604	-230	-273	39	79.3	87.4	40.9	8.3	38.8	7.2
Pinellas	FL	997	5,291	-192	19	-53	-16	-143	-223	2.02	6.96	34.9	22.2	9.7	20.7
Duval	FL	151	1,617	-409	10	-56	-183	-179	-495	0.39	2.69	76.4	25.3	18.4	8.5
Norfolk	VA	1,176	4,856	-525	107	-247	-349	-35	-792	0.80	5.71	71.6	31.2	92.0	19.7
Suffolk	MA	2,662	46,792	-1,737	-1,457	164	-444	0	-1,071	10.1	4.68	12.8	21.0	6.6	4.5
Miami-Dade	FL	3,213	78,575	-5,495	276	-1,916	-2,303	-1,552	-9,548	2.95	25.0	30.7	6.9	13.6	3.6
Total		114,30	433,946	-6,679	14,827	-7,954	-9,978	-3,574	-8,403	2.46	0.91	23.1	21.5	38.4	19.3
Exclude New Orleans		37,315	275,021	-7,580	2,199	-3,107	-5,111	-1,562	-10,265	1.39	0.62	22.4	17.1	145.0	12.9

TABLE S11-A: Impact on Black Population below One Meter by County from the Implicit Racial Segregation Captured By HOLC Mapping, Ranked by Impact in 2020

County	State	2020 Population of HOLC Mapped Areas[1]			By how much does the Black population below 1m exceed what it would be without racial segregation?[2]							Percent of 2020 Population Below 1m [3]			Percent Population In 2020 that was Black			
					--------------------------2020-------------------------					1990					[4]		Below 1m [5]	
		Black	Total		All Zones	Red	Yellow	Blue	Green	All Zones		Red Zone	Other Zones		Red Zone	Other Zones	Red Zone	Other Zones
Exclude NO and Miami		34,102	196,447		-2,085	1,923	-1,191	-2,808	-10	-717		1.36	0.37		22.44	17.33	15.03	19.15

1. The population in areas in the mapped HOLC zones below 1m. In the equations associated with note 2, the two columns are represented by $F_{Black,i}$ and $F_{i.}$
2. For each county *i* and HOLC color zone *j*, we first calculate a counterfactual Black population $X^{Counter}_{Black,i,j}$ based on the assumption that fraction of the population that is Black is the same in each zone: $X^{Counter}_{Black,i,j} = X_{i,j,}\ (X_{Black,i}\ /\ X_i)$ where $X_{i,j,}$ is the total population in HOLC zone j in county I, $X_{Black,i}$ is the total Black population across all the HOLC zones in county I, and X_i is the total population across all HOLC zones in county i. We then calculate a counterfactual Black population <1m, $F^{Counter}_{Black,i,j}$ based on the assumption that if $X^{Counter}_{Black,i,j} < X_{i,j,}$ then the Black population <1m in the counterfactual decreases in proportion to the decline in Black population in the HOLC zone (relative to the actual value), that is

   $$F^{Counter}_{Black,i,j} = F_{Black,i,j} - a_{i,j}\ (X_{i,j,} - X^{Counter}_{Black,i,j})$$

   where $a_{i,j} = F_{Black,i,j} / X_{i,j,Black}$, and $F_{Black,i,j}$ is the actual Black population <1m for HOLC zone *j* in county *i.* On the other hand, we assume that if $X^{Counter}_{Black,i,j} > X_{i,j,}$ then the non-Black population <1m decreases in proportion to the decline in the nonblack population in the HOLC zone, that is:

   $$F^{Counter}_{Black,i,j} = F_{Black,i,j} + b_{i,j}\ (X^{Counter}_{Black,i,j} - X_{i,j,})$$

   where $b_{i,j} = (F_{i,j,} - F_{i,j,Black}) / (X_{i,j} - X_{i,j,Black})$. The color-specific columns here are simply the difference between the estimated and counterfactual populations. For example:

   $$Red_i = F_{Black,i,j} - F^{Counter}_{Black,i,j} \quad \text{for j = “Red”}$$
3. In the equations associated with note 2, the two columns are represented by $F_{,i,red}/X_{i,red}$ and $(F_{,i} - F_{i,red})/(X_i - X_{i,red})$
4. In the equations associated with note 2, the two columns are represented by $X_{Black,i,red}/X_{i,red}$ and $(X_{Black,i} - X_{Black,i,red})/(X_i - X_{i,red})$
5. In the equations associated with note 2, the two columns are represented by $F_{Black,i,red}/F_{i,red}$ and $(F_{Black,i} - F_{Black,i,red})/(F_i - F_{i,red})$
6. This table omits counties where the net impact of segregation on Black residents <1m is negligible.

The purpose of this table is estimate the extent to which racial segregations as reflected by the redlining maps of the Home Owners Loan Corporation, has increased or decreased the population of Black residents in lands vulnerable to sea level rise, by county.

TABLE S11-B: Impact on Black Population below Three Meters by County from the Implicit Racial Segregation Captured By HOLC Mapping, Ranked by Impact in 2020

County	State	2020 Population of HOLC Mapped Areas[1]		By how much does the Black population below 3m exceed what it would be without racial segregation?[2]						Percent of 2020 Population Below 3m [3]		Percent Population In 2020 that was Black [4]		Below 3m [5]	
				2020					1990						
		Black	Total	All Zones	Red	Yellow	Blue	Green	All Zones	Red Zone	Other Zones	Red Zone	Other Zones	Red Zone	Other Zones
Middlesex	MA	8,538	77,927	2,577	2,463	211	-88	-9	2,990	46.2	6.1	11.3	5.6	12.3	8.7
New York	NY	13,573	87,589	2,265	2,856	1	-537	-54	2,704	9.6	1.9	14.8	7.0	17.1	3.9
Queens	NY	39,907	107,808	1,579	2,810	582	-1,812	0	1,056	9.3	5.0	20.7	15.2	44.9	34.2
Camden	NJ	4,202	13,789	938	720	-2	-74	294	746	24.3	6.4	32.6	15.1	31.2	30.1
Alameda	CA	2,452	15,904	445	504	-21	-39	0	467	6.2	1.5	20.5	13.8	21.8	5.1
Union	NJ	2,126	11,332	323	353	-29	0	0	589	9.3	0.9	27.5	19.8	19.1	17.7
Mobile	AL	939	1,242	276	276	0	0	0	316	9.9	0.3	83.8	34.1	74.9	99.6
Atlantic	NJ	10,171	39,358	263	3,086	-479	-2,154	-191	251	66.3	52.8	52.9	17.4	62.4	14.8
Newport News	VA	2,517	3,049	201	91	115	-6	0	223	9.5	21.8	87.7	64.0	87.0	81.4
Bronx	NY	3,856	25,296	169	207	26	-64	0	-47	1.0	2.2	35.2	24.3	23.5	13.7
[6]															
Miami-Dade	FL	49,373	350,762	-131	17,012	-7,971	-5,319	-3,853	-2,091	84.6	81.6	30.7	6.9	29.3	7.4
Hampton	VA	2,802	6,795	-138	228	198	-55	-509	-91	39.0	56.4	78.1	42.5	75.7	36.7
Portsmouth	VA	17,380	30,590	-138	2,147	-1,606	-250	-430	-762	88.8	93.6	77.1	45.3	75.9	46.4
Baltimore	MD	295	1,915	-389	-389	0	0	0	-11	1.5	0.0	44.6	63.3	15.4	21.3
Hudson	NJ	5,452	63,760	-416	-461	45	0	0	1,200	23.6	1.5	9.9	11.3	8.6	8.2
Hillsborough	FL	1,690	18,781	-883	543	-3	-464	-958	-1,527	14.6	16.9	39.6	12.9	19.0	2.3
Pinellas	FL	4,707	20,669	-953	195	-105	-465	-578	-1,045	15.7	21.5	34.9	22.2	12.9	28.0
Jefferson	TX	9,799	23,774	-1,193	12	99	-2,081	777	-2,112	1.0	55.0	72.5	45.6	68.4	41.2
Philadelphia	PA	4,445	18,649	-1,374	-1,375	1	0	0	300	3.2	0.1	35.8	47.0	25.1	0.5
Duval	FL	1,722	10,393	-2,109	268	-396	-873	-1,109	-2,383	3.9	16.3	76.4	25.3	46.2	11.7
Norfolk	VA	31,847	79,194	-3,368	4,866	-2,511	-4,870	-853	-4,322	48.4	76.5	71.6	31.2	69.9	31.4
Suffolk	MA	11,816	156,860	-5,736	-5,288	498	-945	0	-2,778	37.9	13.4	12.8	21.0	9.3	4.6
Total		385,643	1,811,566	-8,007	54,166	-25,307	-26,298	-10,567	-7,392	12.5	5.8	22.5	19.5	26.8	16.4
Exclude New Orleans		303,166	1,620,677	-8,042	40,310	-19,329	-20,608	-8,415	-7,323	11.4	5.3	0.0	19.4	0.0	14.9
Exclude NO and Miami		253,794	1,269,915	-7,911	23,298	-11,358	-15,289	-4,562	-5,232	10.0	3.8	0.0	19.5	0.0	17.9

TABLE S11-B: Impact on Black Population below Three Meters by County from the Implicit Racial Segregation Captured By HOLC Mapping, Ranked by Impact in 2020

	State	2020 Population of HOLC Mapped Areas[1]			By how much does the Black population below 3m exceed what it would be without racial segregation?[2]							Percent of 2020 Population Below 3m [3]			Percent Population In 2020 that was Black [4]		Below 3m [5]	
					--------------------------2020-------------------------					1990								
County		Black	Total		All Zones	Red	Yellow	Blue	Green	All Zones		Red Zone	Other Zones		Red Zone	Other Zones	Red Zone	Other Zones

1. The population in areas in the mapped HOLC zones below 3m. In the equations associated with note 2, the two columns are represented by $F_{Black,i}$ and F_i
2. For each county *i* and HOLC color zone *j*, we first calculate a counterfactual Black population $X^{Counter}_{Black,i,j}$ based on the assumption that fraction of the population that is Black is the same in each zone: $X^{Counter}_{Black,i,j} = X_{i,j,} (X_{Black,i} / X_i)$ where $X_{i,j,}$ is the total population in HOLC zone j in county I, $X_{Black,i}$ is the total Black population across all the HOLC zones in county I, and X_i is the total population across all HOLC zones in county i. We then calculate a counterfactual Black population <3m, $F^{Counter}_{Black,i,j}$ based on the assumption that if $X^{Counter}_{Black,i,j} < X_{i,j,}$ then the Black population <1m decreases in proportion to the decline in Black population in the HOLC zone, that is

   $$F^{Counter}_{Black,i,j} = F_{Black,i,j} - a_{i,j} \left(X_{i,j,} - X^{Counter}_{Black,i,j} \right)$$

   where $a_{i,j} = F_{Black,i,j} / X_{i,j,Black}$, and $F_{Black,i,j}$ is the Black population <3m for HOLC zone j in county I. On the other hand, we assume that if $X^{Counter}_{Black,i,j} > X_{i,j,}$ then the non-Black population <3m decreases in proportion to the decline in the nonblack population in the HOLC zone, that is:

   $$F^{Counter}_{Black,i,j} = F_{Black,i,j} + b_{i,j} (X^{Counter}_{Black,i,j} - X_{i,j,})$$

   where $b_{i,j} = (F_{i,j,} - F_{i,j,Black}) / (X_{i,j} - X_{i,j,Black})$. The color-specific columns here are simply the difference between the estimated and counterfactual populations. For example:

   $$Red_i = F_{Black,i,j} - F^{Counter}_{Black,i,j}$$ for j = "Red"
3. In the equations associated with note 2, the two columns are represented by $F_{i,red}/X_{i,red}$ and $(F_i - F_{i,red})/(X_i - X_{i,red})$
4. In the equations associated with note 2, the two columns are represented by $X_{Black,i,red}/X_{i,red}$ and $(X_{Black,i} - X_{Black,i,red})/(X_i - X_{i,red})$
5. In the equations associated with note 2, the two columns are represented by $F_{Black,i,red}/F_{i,red}$ and $(F_{Black,i} - F_{Black,i,red})/(F_i - F_{i,red})$
6. This table omits counties where the net impact of segregation on Black residents below 3m is less than 130 people.

The purpose of this table is estimate the extent to which racial segregations as reflected by the redlining maps of the Home Owners Loan Corporation, has increased or decreased the population of Black residents in lands vulnerable to sea level rise, by county.

TABLE S11-C: Impact on Black Population in the 100-year Floodplain by County from the Implicit Racial Segregation Captured By HOLC Mapping, Ranked by Impact in 2020

County	State	2020 Population Floodplain in HOLC Mapped Areas[1]		By how much does the Black population in the floodplain exceed what it would be without racial segregation?[2]						Percent of 2020 Population in the Floodplain[3]		Percent Population In 2020 that was Black			
				2020					1990			[4]		Floodplain[5]	
		Black	Total	All Zones	Red	Yellow	Blue	Green	All Zones	Red Zone	Other Zones	Red Zone	Other Zones	Red Zone	Other Zones
New York	NY	5,587	30,887	1174	1208	0	-26	-8	1500	3.8	0.1	14.8	7.0	18.3	4.7
Union	NJ	5,709	18,420	516	630	-56	-32	-26	1752	8.3	3.2	27.5	19.8	38.1	26.1
Kings	NY	10,503	62,275	384	1519	-1136	1	0	1031	4.7	1.0	30.0	22.5	21.7	1.9
Mobile	AL	1,673	2,481	361	418	-41	-15	0	205	16.5	3.6	83.8	34.1	68.0	65.0
Jefferson	AL	2,601	3,442	346	509	-26	-116	-21	1015	3.3	1.1	79.3	43.0	86.6	45.1
Will	IL	1,063	6,049	295	319	-16	-8	0	1169	36.7	5.7	32.1	15.8	26.3	10.6
Queens	NY	15,217	39,674	295	393	289	-387	0	0	2.8	2.0	20.7	15.2	21.1	43.0
Allegheny	PA	705	4,338	225	223	16	-13	0	331	2.4	0.5	35.8	17.8	23.6	7.4
Mercer	NJ	1,190	2,934	194	1	-119	3	309	64	0.1	2.6	52.2	34.1	24.8	40.6
Essex	NJ	3,940	13,017	177	70	623	-481	-35	-112	0.7	2.0	44.8	37.0	44.5	28.9
Portsmouth	VA	4,071	7,344	168	620	-378	-18	-56	329	27.5	18.9	77.1	45.3	70.7	42.6
Camden	NJ	713	3,187	155	161	-1	-18	13	218	5.1	1.5	32.6	15.1	33.0	17.2
St. Louis	MO	1,121	2,121	138	20	151	28	-61	139	2.5	1.3	58.4	31.7	54.7	52.8
Ramsey	MN	260	1,122	117	117	0	0	0	0	2.5	0.0	28.5	12.4	23.2	n/a
Onondaga	NY	782	1,744	112	1	116	-4	-1	305	0.1	1.3	33.3	24.4	35.0	44.9
Hinds	MS	1,832	2,401	109	261	30	33	-215	463	19.7	5.5	85.6	61.2	90.6	55.9
Dutchess	NY	833	1,826	108	3	108	-4	0	304	2.6	5.8	37.3	31.9	22.2	47.3
San Diego	CA	373	6,705	99	112	-8	-4	0	434	3.4	0.5	11.5	5.5	6.2	2.7
Pulaski	AR	299	459	90	86	15	-11	0	151	3.2	0.6	83.5	41.9	82.6	41.4
Montgomery	AL	716	940	85	114	-3	-26	0	363	3.9	1.6	69.5	44.6	91.6	48.4
Stark	OH	178	337	80	80	0	0	0	219	9.6	0.0	45.6	23.9	53.8	3.8
Allen	IN	275	1,756	78	65	19	-5	-1	49	19.1	1.8	42.1	18.8	23.4	12.4
Shawnee	KS	301	1,969	75	77	0	0	-1	305	11.2	0.1	14.9	8.9	15.4	6.5
McLennan	TX	547	1,930	71	93	-15	-6	-1	105	8.5	2.7	30.1	19.4	39.0	20.2
Luzerne	PA	333	2,242	58	-2	0	-1	61	12	0.4	3.7	3.7	10.2	15.4	14.8

TABLE S11-C: Impact on Black Population in the 100-year Floodplain by County from the Implicit Racial Segregation Captured By HOLC Mapping, Ranked by Impact in 2020

County	State	2020 Population Floodplain in HOLC Mapped Areas[1]		By how much does the Black population in the floodplain exceed what it would be without racial segregation?[2]						Percent of 2020 Population in the Floodplain[3]		Percent Population In 2020 that was Black			
				------------------------2020--------------------					1990			[4]		Floodplain[5]	
		Black	Total	All Zones	Red	Yellow	Blue	Green	All Zones	Red Zone	Other Zones	Red Zone	Other Zones	Red Zone	Other Zones
Ingham	MI	898	5,137	57	29	81	-37	-17	80	22.6	4.3	22.9	17.9	18.1	17.4
Niagara	NY	304	1,407	56	57	-1	0	0	150	22.1	1.6	29.7	22.9	31.8	4.8
Black Hawk	IA	139	315	55	58	0	-2	0	105	3.6	0.2	41.6	18.5	50.1	16.3
Bronx	NY	1,151	10,061	50	58	8	0	-16	-62	0.3	0.9	35.2	24.3	20.9	10.1
Ohio	WV	303	4,773	49	78	-8	-20	-1	-37	44.3	17.3	10.3	4.1	7.5	5.2
Galveston	TX	5,098	24,165	48	570	22	-197	-348	530	83.5	79.0	31.4	16.9	29.7	18.7
Kanawha	WV	406	1,418	47	44	2	13	-12	130	7.6	4.9	28.2	15.9	42.0	25.4
Newport News	VA	718	886	45	8	38	-1	0	42	1.0	7.4	87.7	64.0	80.7	81.1
Winnebago	IL	348	1,192	45	50	-4	0	0	8	2.2	0.9	30.6	19.8	32.3	23.5
Summit	OH	204	1,054	44	71	-27	0	0	65	2.3	0.3	46.3	22.1	43.7	8.2
Sedgwick	KS	291	1,266	42	43	0	0	-1	107	2.5	0.1	15.6	9.7	23.5	4.4
Los Angeles	CA	1,322	27,416	42	-8	21	31	-2	193	0.1	0.8	7.7	8.5	9.5	4.6
Dallas	TX	469	1,992	36	29	57	-24	-26	92	0.3	0.7	33.8	13.4	46.9	22.1
Lawrence	PA	83	487	31	29	2	0	0	65	21.3	0.9	24.8	11.5	17.4	16.3
Middlesex	MA	248	4,672	29	56	9	-8	-28	-16	1.0	0.8	11.3	5.6	13.3	3.1
[6]															
Albany[6]	NY	527	2,764	16	162	-146	0	0	-91	3.5	2.7	54.0	17.2	36.8	10.9
Sacramento[7]	CA	0	0	0	0	0	0	0	0		0.0	6.7	7.0		
[6]															
Guilford	NC	267	475	-30	5	8	-4	-39	-56	0.2	1.2	82.6	37.0	54.3	56.3
Davidson	TN	278	1,564	-33	87	-1	-10	-108	-171	0.9	0.7	32.9	10.4	29.1	4.9
Dauphin	PA	875	3,225	-41	-25	0	11	-28	-174	4.3	5.7	36.6	40.6	20.1	28.9
Nassau	NY	22	385	-51	-51	0	0	0	0	97.2	0.4	5.6	36.2	5.7	0.0
Richmond	NY	272	11,975	-57	63	-118	-2	0	-44	7.5	1.7	18.0	6.8	1.9	3.4
Somerset	NJ	349	2,044	-59	-70	-1	13	0	-11	30.9	6.8	8.4	20.4	12.4	19.4
Fulton	GA	408	1,132	-60	36	-51	-14	-31	-70	0.2	0.8	58.9	24.9	81.6	31.0
Norfolk	MA	540	11,016	-63	12	-79	5	-1	-30	11.5	4.7	5.9	5.0	4.1	5.1

TABLE S11-C: Impact on Black Population in the 100-year Floodplain by County from the Implicit Racial Segregation Captured By HOLC Mapping, Ranked by Impact in 2020

County	State	2020 Population Floodplain in HOLC Mapped Areas[1]		By how much does the Black population in the floodplain exceed what it would be without racial segregation?[2]						Percent of 2020 Population in the Floodplain[3]		Percent Population In 2020 that was Black			
				------------------------2020--------------------					1990			[4]		Floodplain[5]	
		Black	Total	All Zones	Red	Yellow	Blue	Green	All Zones	Red Zone	Other Zones	Red Zone	Other Zones	Red Zone	Other Zones
Macomb	MI	17	417	-64	0	-5	-59	0	-1		0.7	44.3	17.5	n/a	2.4
Harris	TX	697	4,944	-65	49	-162	-42	91	-18	0.7	2.9	43.0	10.6	36.0	13.1
Westchester	NY	532	4,109	-79	18	10	-19	-89	-174	0.8	1.3	28.3	20.6	18.7	12.3
Hampton	VA	615	1,865	-102	21	52	-6	-170	-60	4.7	16.6	78.1	42.5	58.7	31.6
Jefferson	LA	179	1,911	-133	32	-51	-42	-72	-113	2.4	10.4	40.9	8.3	44.9	7.1
Baltimore	MD	969	2,175	-142	-123	18	18	-56	-69	0.9	0.4	44.6	63.3	52.7	36.6
Caddo	LA	739	1,530	-142	62	91	13	-308	-147	3.5	4.0	82.0	51.3	63.2	43.8
Philadelphia	PA	3,412	5,454	-163	-165	2	1	0	260	0.9	0.1	35.8	47.0	67.9	8.4
Cook	IL	924	8,077	-178	86	-83	-4	-178	-272	0.5	0.2	32.7	23.9	11.8	11.2
Duval	FL	623	1,995	-224	90	-71	-179	-64	-306	1.1	2.9	76.4	25.3	54.2	25.2
Wayne	MI	2,345	7,549	-264	-43	-222	0	0	-219	0.8	0.9	46.2	52.1	68.6	20.7
Hudson	NJ	2,241	35,229	-277	-278	2	0	0	586	14.0	0.2	9.9	11.3	6.4	3.3
Marion	IN	2,023	17,563	-452	-256	257	-212	-241	-223	7.0	3.1	26.3	29.7	5.5	16.3
Atlantic	NJ	151	6,181	-618	0	-120	-438	-60	-1587	n/a	10.8	52.9	17.4	n/a	2.4
Suffolk	MA	675	16,585	-623	-657	55	-21	0	-202	4.4	1.2	12.8	21.0	3.6	5.2
Pinellas	FL	3,425	16,560	-667	188	-88	-237	-530	-742	13.8	16.4	34.9	22.2	14.1	24.7
Orleans	LA	12,798	26,735	-680	1853	-806	-1100	-627	-1082	10.3	16.6	58.4	29.4	74.3	33.3
Hillsborough	FL	1,216	15,092	-813	379	-3	-343	-846	-1433	11.4	13.8	39.6	12.9	16.9	2.4
Norfolk	VA	3,918	14,493	-1576	563	-588	-1444	-107	-1970	4.5	16.0	71.6	31.2	86.3	19.2
Miami-Dade	FL	5,596	132,577	-8437	440	-3787	-2639	-2451	7408	11.0	39.7	30.7	6.9	5.8	4.0
Total		130,653	766,213	-9,668	11,450	-6,171	-8,287	-6,660	-9847	2.63	1.59	26.2	19.5	21.3	14.5
Exclude New Orleans		117,855	739,478	-8,988	9,596	-5,365	-7,186	-6,033	-8,766	2.57	1.54	26.4	19.4	19.5	13.8
Exclude New Orleans & Miami		112,259	606,900	-551	9,157	-1,579	-4,548	-3,582	4,662	2.47	1.15	25.9	19.5	20.2	17.2

TABLE S11-C: Impact on Black Population in the 100-year Floodplain by County from the Implicit Racial Segregation Captured By HOLC Mapping, Ranked by Impact in 2020

County	State	2020 Population Floodplain in HOLC Mapped Areas[1]			By how much does the Black population in the floodplain exceed what it would be without racial segregation?[2]							Percent of 2020 Population in the Floodplain[3]			Percent Population In 2020 that was Black			
					------------------------2020--------------------					1990					[4]		Floodplain[5]	
		Black	Total		All Zones	Red	Yellow	Blue	Green	All Zones		Red Zone	Other Zones		Red Zone	Other Zones	Red Zone	Other Zones

1. The population in areas that are both within the mapped 100-year floodplain (A zone) and the mapped HOLC zones. In the equations associated with note 2, the two columns are represented by $F_{Black,i}$ and F_i
2. For each county *i* and HOLC color zone *j*, we first calculate a counterfactual Black population $X^{Counter}_{Black,i,j}$ based on the assumption that the fraction of the population that is Black is the same in each zone: $X^{Counter}_{Black,i,j} = X_{i,j}\ (X_{Black,i}\ /\ X_i)$ where $X_{i,j,}$ is the total population in HOLC zone j in county i, $X_{Black,i}$ is the total Black population across all the HOLC zones in county I, and X_i is the total population across all HOLC zones in county i. We then calculate a counterfactual Black population in the floodplain $F^{Counter}_{Black,i,j}$ based on the assumption that if $X^{Counter}_{Black,i,j} < X_{i,j,}$ then the Black population in the floodplain decreases in proportion to the decline in Black population in the HOLC zone, that is
$$F^{Counter}_{Black,i,j} = F_{Black,i,j} - a_{i,j}\ (X_{i,j,} - X^{Counter}_{Black,i,j})$$
where $a_{i,j} = F_{Black,i,j} / X_{i,j,Black}$, $= F_{Black,i,j}$ is the Black population in the floodplain for HOLC zone j in county I. On the other hand, we assume that if $X^{Counter}_{Black,i,j} > X_{i,j,}$ then the non-Black population in the floodplain decreases in proportion to the decline in the nonblack population in the HOLC zone, that is:
$$F^{Counter}_{Black,i,j} = F_{Black,i,j} + b_{i,j}\ (X^{Counter}_{Black,i,j} - X_{i,j,})$$
where $b_{i,j} = (F_{i,j,} - F_{i,j,Black}) / (X_{i,j} - X_{i,j,Black})$. The color-specific columns here are simply the difference between the estimated and counterfactual populations. For example:
$$Red_i = F_{Black,i,j} - F^{Counter}_{Black,i,j} \quad \text{for j = “Red”}$$
3. In the equations associated with note 2, the two columns are represented by $F_{i,red}/X_{i,red}$ and $(F_i - F_{i,red})/(X_i - X_{i,red})$
4. In the equations associated with note 2, the two columns are represented by $X_{Black,i,red}/X_{i,red}$ and $(X_{Black,i} - X_{Black,i,red})/(X_i - X_{i,red})$
5. In the equations associated with note 2, the two columns are represented by $F_{Black,i,red}/F_{i,red}$ and $(F_{Black,i} - F_{Black,i,red})/(F_i - F_{i,red})$
6. This table omits counties where the net impact of segregation on Black residents in the floodplain is less than 29, except Sacramento (see note 7) and Albany, where the impact on the red and yellow zones is greater than 100 in spite of the small net impact.
7. A negligible portion of Sacramento’s population is in the mapped A zone this table, though a large part of the city is in the shaded X zone representing areas that would be vulnerable to flooding but for protection by levees. Significant creek flooding does occur in these areas. See Katz, I. “A Racist Past, a Flooded Future, Formerly Redlines Areas have $107 Billion worth of Homes Facing High Flood Risk.” Redfin News (March 14, 2021). https://www.redfin.com/news/redlining-flood-risk/

The purpose of this table is estimate the extent to which racial segregations as reflected by the redlining maps of the Home Owners Loan Corporation, has increased or decreased the population of Black residents in lands vulnerable to flooding, by county.

TABLE S12-A: Impact on Black Population below One Meter, by HOLC Zone, from the Implicit Racial Segregation Captured By HOLC Mapping (thousands)

		Red Zone				Yellow Zone				Total: All Four HOLC Zones			
		1990	2000	2010	2020	1990	2000	2010	2020	1990	2000	2010	2020
1	Coastal Population	5,650	5,839	5,967	6,367	8,881	9,519	9,544	9,913	19,400	20,349	20,458	21,428
2	Black Coastal Population	1,956	1,830	1,618	1,503	2,028	2,057	1,918	1,770	4,909	4,712	4,516	4,201
3	Population <1m	200	196	142	160	175	177	164	180	481	475	403	434
4	Black Population <1m	132	123	62	62	61	65	47	42	210	204	122	114
**Percentages**													
5	Black share of Population	34.6	31.3	27.1	23.6	22.8	21.6	20.1	17.9	25.3	23.2	22.1	19.6
6	Black share <1m[1]	66.2	62.8	43.5	38.4	34.6	36.5	28.7	23.2	43.7	42.9	30.4	26.3
7	Population Residing <1m [2]	3.54	3.35	2.37	2.51	1.97	1.86	1.72	1.81	2.48	2.33	1.97	2.03
8	Residing <1m: Black[3]	6.77	6.72	3.81	4.09	2.99	3.14	2.46	2.35	4.28	4.33	2.71	2.72
9	Residing <1m non-Black[4]	1.83	1.82	1.84	2.03	1.67	1.50	1.54	1.70	1.87	1.73	1.76	1.86
**Ratios of Disproportionality**													
	Black Residents to be <1m[5]	1.91	2.00	1.60	1.63	1.52	1.69	1.43	1.30	1.73	1.85	1.38	1.34
	Residents <1m to be in zone[6]	1.43	1.44	1.20	1.24	0.79	0.79	0.87	0.90	1.00	1.00	1.00	1.00
	Black Residents <1m to be in zone[7]	1.58	1.55	1.40	1.50	0.70	0.73	0.91	0.86	1.00	1.00	1.00	1.00
	Residents <1m to be Black & in zone[8]	2.73	2.88	1.93	2.02	1.21	1.34	1.25	1.16	1.73	1.85	1.38	1.34
	Black Residents to be in this zone[9]	1.37	1.35	1.23	1.20	0.90	0.93	0.91	0.91	1.00	1.00	1.00	1.00
**Total Desegregation Counterfactual: County-By-County[10]**													
	Black Coastal Population	1,546	1,439	1,385	1,316	2,053	2,028	1,914	1,764	4909	4712	4516	4201
	Black Residents <1m	101	96	47	47	76	76	54	50	219	211	131	121
	Effect of segregation on Black pop	32	27	14	15	-16	-11	-7	-8	-8.4	-6.8	-8.4	-6.7
	Same as above as a ratio	1.32	1.28	1.31	1.32	0.80	0.85	0.88	0.84	0.96	0.97	0.94	0.94
**Total Desegregation Counterfactual: Nationwide aggregation [11]**													
	Black Coastal Population	1,430	1,352	1,317	1,248	2,247	2,204	2,107	1,943	4,909	4,712	4,516	4,201
	Black Residents <1m	97	91	50	51	64	67	50	45	186	181	117	109
	Effect of segregation on Black pop	36	32	11	10	-3.7	-2.2	-2.9	-2.9	24	23	5.7	5.3
	Same as above as a ratio	1.37	1.35	1.23	1.20	0.94	0.97	0.94	0.93	1.13	1.13	1.05	1.05

Notes
1. Line 4/Line 3
2. Line 3/Line 1
3. Line 4/Line 2
4. (Line 3 – Line 4) /(Line 1 – Line 2)
5. Line 6/Line 5
6. (Line 7, this zone)/(Line 7, of all zones)
7. (Line 8, this zone)/(Line 8, of all zones)
8. (Line 8, this zone)/(Line 7, of all zones)
9. $\frac{Line\_2\_this\_zone/Line\_2\_all\_zones}{Line\_1\_this\_zone/Line\_1\_all\_zones}$
10. See Table S11-A for Calculations

NOTE: See Table S12-D for further explanation about Ratios of Disproportionality and Counterfactuals

TABLE S12-A: Impact on Black Population below One Meter, by HOLC Zone, from the Implicit Racial Segregation Captured By HOLC Mapping (thousands)

		Blue Zone				Green Zone				Total: All Four HOLC Zones[12]			
		1990	2000	2010	2020	1990	2000	2010	2020	1990	2000	2010	2020
1	Coastal Population	4,036	4,149	4,125	4,290	834	842	822	859	19,400	20,349	20,458	21,428
2	Black Coastal Population	831	708	862	815	94	117	117	112	4,909	4,712	4,516	4,201
3	Population <1m	75	73	70	67	31	29	27	27	481	475	403	434
4	Black Population <1m	10	10	9	7	7.5	6.4	5.1	4.2	210	204	122	114
**Percentages**													
5	Black share of Population	20.6	17.1	20.9	19.0	11.3	13.9	14.3	13.0	25.3	23.2	22.1	19.6
6	Black share <1m[1]	12.9	13.6	12.3	10.3	24.4	22.1	19.0	15.6	43.7	42.9	30.4	26.3
7	Population Residing <1m [2]	1.86	1.77	1.70	1.56	3.69	3.46	3.27	3.17	2.48	2.33	1.97	2.03
8	Residing <1m: Black[3]	1.16	1.41	1.00	0.84	8.00	5.52	4.36	3.79	4.28	4.33	2.71	2.72
9	Residing <1m non-Black[4]	2.04	1.84	1.89	1.72	3.15	3.13	3.09	3.08	1.87	1.73	1.76	1.86
**Ratios of Disproportionality**													
	Black Residents to be <1m[5]	0.63	0.80	0.59	0.54	2.17	1.60	1.33	1.20	1.73	1.85	1.38	1.34
	Residents <1m to be in zone[6]	0.75	0.76	0.86	0.77	1.49	1.48	1.66	1.57	1.00	1.00	1.00	1.00
	Black Residents <1m to be in zone[7]	0.27	0.33	0.37	0.31	1.87	1.27	1.61	1.39	1.00	1.00	1.00	1.00
	Residents <1m to be Black & in zone[8]	0.47	0.61	0.51	0.42	3.23	2.36	2.21	1.87	1.73	1.85	1.38	1.34
	Black Residents to be in this zone[9]	0.81	0.74	0.95	0.97	0.45	0.60	0.65	0.66	1.00	1.00	1.00	1.00
**Total Desegregation Counterfactual: County-By-County (thousands)** [10]													
	Black Population	1,094	1,040	1,022	941	216	205	194	180	4909	4712	4516	4201
	Black Residents <1m	28	26	21	17	14	12	9	8	219	211	131	121
	Effect of segregation on Black pop	-18	-16	-12	-10	-6.6	-6.0	-4.2	-3.6	-8.4	-6.8	-8.4	-6.7
	Same as above as a ratio	0.35	0.38	0.42	0.41	0.53	0.52	0.55	0.54	0.96	0.97	0.94	0.94
**Total Desegregation Counterfactual: Nationwide aggregation (thousands)** [11]													
	Black Population	1,021	961	910	841	211	195	181	168	4,909	4,712	4,516	4,201
	Black Residents <1m	14	15	10	7	11	9	7	6	186	181	117	109
	Effect of segregation on Black pop	-3.9	-4.7	-0.9	-0.4	-3.7	-2.4	-2.0	-1.7	24	23	5.7	5.3
	Same as above as a ratio	0.71	0.68	0.90	0.94	0.67	0.72	0.72	0.71	1.13	1.13	1.05	1.05

Notes (continued)

11. Same approach as Table S11-A, except that aggregation of categories is national not county
12. Totals for all HOLC zones are shown on both pages.
13. Population of counties with at least one resident living in a block with some land in the area mapped by HOLC and at least part of a building below 1m.

The purpose of this table is estimate the extent to which racial segregations as reflected by the redlining maps of the Home Owners Loan Corporation, has increased or decreased the population of Black residents in lands vulnerable to sea level rise looding, at the national scale.

TABLE S12-B: Impact on Black Population below Three Meters, by HOLC Zone, from the Implicit Racial Segregation Captured By HOLC Mapping, (thousands)

		Red Zone					Yellow Zone					Total: All Four HOLC Zones			
		1990	2000	2010	2020		1990	2000	2010	2020		1990	2000	2010	2020
1	Coastal Population	6,071	6,243	6,364	6,768		9,412	10,025	10,037	10,401		20,829	21,732	21,816	22,777
2	Black Coastal Population	1,999	1,864	1,644	1,526		2,057	2,082	1,942	1,794		4,989	4,782	4,578	4,263
3	Population <3m	771	787	768	848		571	592	629	661		1,648	1,684	1,698	1,812
4	Black Population <3m	321	307	235	227		142	149	141	127		505	499	412	386
**Percentages**															
5	Black share of Population	32.9	29.9	25.8	22.5		21.9	20.8	19.3	17.2		24.0	22.0	21.0	18.7
6	Black share <3m[1]	41.6	39.0	30.6	26.8		24.9	25.2	22.4	19.2		30.6	29.6	24.3	21.3
7	Population Residing <3m [2]	12.70	12.60	12.06	12.54		6.07	5.90	6.27	6.36		7.91	7.75	7.78	7.95
8	Residing <3m: Black[3]	16.05	16.48	14.27	14.90		6.91	7.17	7.25	7.08		10.12	10.44	9.00	9.05
9	Residing <3m non-Black[4]	11.05	10.95	11.29	11.85		5.83	5.57	6.04	6.21		7.21	6.99	7.46	7.70
**Ratios of Disproportionality**															
10	Black Residents to be <3m[5]	1.26	1.31	1.18	1.19		1.14	1.22	1.16	1.11		1.28	1.35	1.16	1.14
11	Residents <3m to be in zone[6]	1.60	1.59	1.53	1.58		0.77	0.76	0.81	0.80		1.00	1.00	1.00	1.00
12	Black Residents <3m to be in zone[7]	1.59	1.58	1.59	1.65		0.68	0.69	0.81	0.78		1.00	1.00	1.00	1.00
13	People <3m to be Black & in zone[8]	2.03	2.13	1.83	1.87		0.87	0.93	0.93	0.89		1.28	1.35	1.16	1.14
14	Black Residents to be in this zone[9]	1.37	1.30	1.18	1.10		0.91	0.94	0.92	0.92		1.00	1.00	1.00	1.00
**Total Desegregation Counterfactual: County-By-County[10]**															
	Black Population	1,570	1,458	1,400	1,331		2,084	2,056	1,939	1,789		4989	4782	4578	4263
	Black Residents <3m	229	227	174	173		182	183	168	152		512	506	421	394
	Effect of segregation on Black pop	92	80	60	54		-40	-34	-27	-25		-7.4	-6.6	-9.2	-8.0
	Same as above as a ratio	1.40	1.35	1.35	1.31		0.78	0.82	0.84	0.83		0.99	0.99	0.98	0.98
**Total Desegregation Counterfactual: Nationwide aggregation [11]**															
	Black Population	1,454	1,374	1,335	1,267		2,254	2,206	2,106	1,947		4,989	4,782	4,578	4,263
	Black Residents <3m	233	226	191	189		154	156	151	136		451	447	387	364
	Effect of segregation on Black pop	87	81	44	39		-11.5	-6.9	-9.9	-9.5		54	53	24.4	21.8
	Same as above as a ratio	1.37	1.36	1.23	1.20		0.93	0.96	0.93	0.93		1.12	1.12	1.06	1.06

Notes

1. Line 4/Line 3
2. Line 3/Line 1
3. Line 4/Line 2
4. (Line 3 – Line 4) /(Line 1 – Line 2)
5. Line 6/Line 5
6. (Line 7, this zone)/(Line 7, of all zones)
7. Line 8, this zone)/(Line 8, of all zones)
8. (Line 8, this zone)/(Line 7, of all zones)
9. $\frac{Line\_2\_this\_zone/Line\_2\_all\_zones}{Line\_1\_this\_zone/Line\_1\_all\_zones}$
10. See Table S11-A for Calculations

NOTE: See Table S12-D for further explanation about Ratios of Disproportionality and Counterfactuals

TABLE S12-B: Impact on Black Population below Three Meters, by HOLC Zone, from the Implicit Racial Segregation Captured By HOLC Mapping, (thousands)													
		Blue Zone				Green Zone				Total: All Four HOLC Zones[12]			
		1990	2000	2010	2020	1990	2000	2010	2020	1990	2000	2010	2020
1	Coastal Population	4,432	4,545	4,516	4,674	913	918	900	934	20,829	21,732	21,816	22,777
2	Black Coastal Population	838	718	874	830	95	117	118	113	4,989	4,782	4,578	4,263
3	Population <3m	232	232	229	228	74	73	71	74	1,648	1,684	1,698	1,812
4	Black Population <3m	31	32	27	23	10.4	10.4	9.6	8.4	505	499	412	386
**Percentages**													
5	Black share of Population	18.9	15.8	19.4	17.8	10.3	12.8	13.1	12.1	24.0	22.0	21.0	18.7
6	Black share <3m[1]	13.6	13.9	11.8	10.1	14.1	14.3	13.4	11.4	30.6	29.6	24.3	21.3
7	Population Residing <3m [2]	5.23	5.11	5.08	4.88	8.08	7.96	7.95	7.91	7.91	7.75	7.78	7.95
8	Residing <3m: Black[3]	3.75	4.51	3.08	2.77	10.99	8.90	8.10	7.46	10.12	10.44	9.00	9.05
9	Residing <3m non-Black[4]	5.58	5.23	5.55	5.34	7.74	7.83	7.92	7.98	7.21	6.99	7.46	7.70
**Ratios of Disproportionality**													
10	Black residents in Zone to be <3m[5]	0.72	0.88	0.61	0.57	1.36	1.12	1.02	0.94	1.28	1.35	1.16	1.14
11	People <3m to be in zone[6]	0.66	0.66	0.65	0.61	1.02	1.03	1.02	1.00	1.00	1.00	1.00	1.00
12	Black Residents <3m to be in zone[7]	0.37	0.43	0.34	0.31	1.09	0.85	0.90	0.82	1.00	1.00	1.00	1.00
13	People <3m to be Black & in zone[8]	0.47	0.58	0.40	0.35	1.39	1.15	1.04	0.94	1.28	1.35	1.16	1.14
14	Black Residents to be in this zone[9]	0.79	0.72	0.92	0.95	0.43	0.58	0.63	0.64	1.00	1.00	1.00	1.00
**Total Desegregation Counterfactual: County-By-County (thousands)** [10]													
	Black Population	1,115	1,060	1,041	960	219	208	197	183	4989	4782	4578	4263
	Black Residents <3m	74	70	57	49	28	26	22	19	512	506	421	394
	Effect of segregation on Black pop	-42	-38	-30	-26	-17.9	-15.7	-12.1	-10.6	-7.4	-6.6	-9.2	-8.0
	Same as above as a ratio	0.43	0.46	0.47	0.47	0.37	0.40	0.44	0.44	0.99	0.99	0.98	0.98
**Total Desegregation Counterfactual: Nationwide aggregation (thousands)** [11]													
	Black Population	1,062	1,000	948	875	219	202	189	175	4,989	4,782	4,578	4,263
	Black Residents <3m	44	47	31	25	20	17	15	13	451	447	387	364
	Effect of segregation on Black pop	-12.5	-14.7	-4.1	-2.4	-9.6	-6.6	-5.6	-5.0	54	53	24.4	21.8
	Same as above as a ratio	0.72	0.69	0.87	0.91	0.52	0.61	0.63	0.63	1.12	1.12	1.06	1.06

Notes (continued)

11. Same approach as Table S11-A, except that aggregation of categories is national not county
12. Totals for all HOLC zones are shown on both pages.
13. Population of counties with at least one resident living in a block with some land in the area mapped by HOLC and at least part of a building below 3m.

The purpose of this table is estimate the extent to which racial segregations as reflected by the redlining maps of the Home Owners Loan Corporation, has increased or decreased the population of Black residents in lands vulnerable to sea level rise, at the national scale.

TABLE S12-C: Impact on Black Population in the 100-year Floodplain, by HOLC Zone, from the Implicit Racial Segregation Captured By HOLC Mapping (thousands)

		Red Zone				Yellow Zone				Total: All Four HOLC Zones			
		1990	2000	2010	2020	1990	2000	2010	2020	1990	2000	2010	2020
1	Population	10,778	10,722	10,515	10,933	18,713	19,167	18,537	18,881	40,760	41,168	40,022	41,009
2	Black Population	3,946	3,631	3,153	2,867	4,549	4,645	4,241	3,902	10,420	10,162	9,434	8,719
3	Population in floodplain	284	287	273	288	270	277	281	305	737	745	728	766
4	Black Population in floodplain	96	89	66	61	56	59	55	53	173	170	140	131
**Percentages**													
5	Black share of Population	36.6	33.9	30.0	26.2	24.3	24.2	22.9	20.7	25.6	24.7	23.6	21.3
6	Black share of Pop in floodplain[1]	33.8	31.1	24.3	21.3	20.7	21.5	19.7	17.5	23.4	22.7	19.2	17.1
7	Floodplain share of Population[2]	2.64	2.68	2.59	2.64	1.44	1.44	1.52	1.62	1.81	1.81	1.82	1.87
8	Reside in floodplain: Black share[3]	2.44	2.46	2.10	2.14	1.22	1.28	1.31	1.37	1.66	1.67	1.48	1.50
9	Reside in floodplain non-Black share[4]	2.75	2.79	2.80	2.81	1.51	1.50	1.58	1.68	1.86	1.86	1.92	1.97
**Ratios of Disproportionality**													
10	Black in Zone to be in floodplain [5]	0.92	0.92	0.81	0.81	0.85	0.89	0.86	0.85	0.92	0.92	0.82	0.80
11	People in floodplain to be in zone[6]	1.46	1.48	1.43	1.41	0.80	0.80	0.83	0.87	1.00	1.00	1.00	1.00
12	Black in floodplain to be in zone[7]	1.47	1.48	1.27	1.29	0.74	0.77	0.88	0.91	1.00	1.00	1.00	1.00
13	People in floodpl to be Black & in zone[8]	1.35	1.36	1.16	1.14	0.68	0.71	0.72	0.73	0.92	0.92	0.82	0.80
14	Black Residents to be in this zone[9]	1.43	1.37	1.27	1.23	0.95	0.98	0.97	0.97	1.00	1.00	1.00	1.00
**Total Desegregation Counterfactual: County-By-County**[9]													
	Black Population	3,008	2,810	2,574	2,383	4,784	4,731	4,369	4,015	10,673	10,325	9,529	8,778
	Black Residents in floodplain	71	67	53	50	65	67	61	60	182	176	150	140
	Effect of segregation on Black pop	25	22	14	11	-9	-7	-6	-6	-9.8	-6.2	-10.2	-9.7
	Same as above as a ratio	1.36	1.32	1.26	1.23	0.86	0.89	0.90	0.90	0.95	0.96	0.93	0.93
**Total Desegregation Counterfactual: Nationwide aggregation** [10]													
	Black Population	2,755	2,646	2,479	2,325	4,784	4,731	4,369	4,015	10,420	10,261	9,652	9,201
	Black Residents in floodplain	67	65	52	50	59	61	57	55	165	164	142	137
	Effect of segregation on Black pop	29	24	14	12	-3.6	-1.3	-2.0	-1.9	8	5	-2.2	-6.7
	Same as above as a ratio	1.43	1.37	1.27	1.23	0.94	0.98	0.96	0.97	1.05	1.03	0.98	0.95

Notes

1. Line 4/Line 3
2. Line 3/Line 1
3. Line 4/Line 2
4. (Line 3 – Line 4) /(Line 1 – Line 2)
5. Line 5/Line 6
6. (Line 7, this zone)/(Line 7, of all zones)
7. (Line 8, this zone)/(Line 8, of all zones)
8. (Line 8, this zone)/(Line 7, of all zones)
9. $\frac{Line\_2\_this\_zone/Line\_2\_all\_zones}{Line\_1\_this\_zone/Line\_1\_all\_zones}$
10. See Table S11-A for Calculations

NOTE: See Table S12-D for further explanation about Ratios of Disproportionality and Counterfactuals

TABLE S12-C: Impact on Black Population in the 100-year Floodplain, by HOLC Zone, from the Implicit Racial Segregation Captured By HOLC Mapping (thousands)

		Blue Zone				Green Zone				Total: All Four HOLC Zones			
		1990	2000	2010	2020	1990	2000	2010	2020	1990	2000	2010	2020
1	Population	8,890	8,940	8,661	8,814	2,379	2,339	2,309	2,382	40,760	41,168	40,022	41,009
2	Black Population	1,687	1,609	1,758	1,670	238	277	283	280	10,420	10,162	9,434	8,719
3	Population in floodplain	131	130	124	120	52	51	51	52	737	745	728	766
4	Black Population in floodplain	16	16	14	12	4.3	4	4	4	173	170	140	131
**Percentages**													
5	Black share of Population	19.0	18.0	20.3	18.9	10.0	11.8	12.2	11.7	25.6	24.7	23.6	21.3
6	Black share of Pop in floodplain[1]	12.5	12.7	11.4	9.9	8.4	8.5	8.2	7.6	23.4	22.7	19.2	17.1
7	Floodplain share of Population[2]	1.48	1.45	1.43	1.36	2.18	2.20	2.20	2.19	1.81	1.81	1.82	1.87
8	Reside in floodplain: Black share[3]	0.97	1.02	0.80	0.71	1.82	1.58	1.47	1.41	1.66	1.67	1.48	1.50
9	Reside in floodplain non-Black share[4]	1.59	1.54	1.59	1.52	2.22	2.28	2.30	2.30	1.86	1.86	1.92	1.97
**Ratios of Disproportionality**													
10	Black in Zone to be in floodplain [5]	0.66	0.71	0.56	0.52	0.84	0.72	0.67	0.64	0.92	0.92	0.82	0.80
11	People in floodplain to be in zone[6]	0.82	0.80	0.78	0.73	1.20	1.21	1.21	1.17	1.00	1.00	1.00	1.00
12	Black in floodplain to be in zone[7]	0.59	0.61	0.54	0.48	1.10	0.95	0.99	0.94	1.00	1.00	1.00	1.00
13	People in floodpl to be Black & in zone[8]	0.54	0.57	0.44	0.38	1.01	0.87	0.81	0.76	0.92	0.92	0.82	0.80
14	Black Residents to be in this zone[9]	0.74	0.73	0.86	0.89	0.39	0.48	0.52	0.55	1.00	1.00	1.00	1.00
**Total Desegregation Counterfactual: County-By-County (thousands)[9]**													
	Black Population	2,273	2,207	2,041	1,874	608	577	544	506	10,673	10,325	9,529	8,778
	Black Residents in floodplain	31	28	24	20	15	14	12	11	182	176	150	140
	Effect of segregation on Black pop	-15	-12	-10	-8	-10.8	-9.3	-7.9	-6.7	-9.8	-6.2	-10.2	-9.7
	Same as above as a ratio	0.52	0.59	0.59	0.59	0.29	0.32	0.34	0.37	0.95	0.96	0.93	0.93
**Total Desegregation Counterfactual: Nationwide aggregation (thousands)[10]**													
	Black Population	2,273	2,285	2,214	2,253	608	598	590	609	10,420	10,261	9,652	9,201
	Black Residents in floodplain	26	27	21	21	13	12	11	12	165	164	142	137
	Effect of segregation on Black pop	-9.3	-10.4	-7.2	-8.8	-8.2	-7.3	-7.1	-7.6	8	5	-2.2	-6.7
	Same as above as a ratio	0.64	0.61	0.66	0.57	0.35	0.37	0.37	0.34	1.05	1.03	0.98	0.95

Notes (continued)

11. Same approach as Table S11-A, except that aggregation of categories is national not county
12. Totals for all HOLC zones are shown on both pages.
13. Population of counties with at least some land in the area mapped by HOLC.

The purpose of this table is estimate the extent to which racial segregations as reflected by the redlining maps of the Home Owners Loan Corporation, has increased or decreased the population of Black residents in lands vulnerable to flooding, at the national scale

TABLE S12-D: Impact on Black Population in Hazard Zones, by HOLC Zone, from the Implicit Racial Segregation Captured By HOLC Mapping: Additional Explanations of Calculations from Tables S12-A, S12-B, and S12-C.				
Line Number		Value for year 2020 in Table S12-A for:		Meaning in Plain English
	Line Name	Red Zone	All Zones	
**Ratios of Disproportionality**				
10	Black in Zone to be in floodplain	1.63	1.34	This is the national ratio of disproportionality defined in the article text. Black people living in the red zone are 63% more likely to live below 1m than the general population of the red zone; across all HOLC zones, black residents are 34% more likely to live <1m than the general population.
11	People in floodplain to be in zone	1.24	1.00	People in the red zone are 24% more likely to live <1m than the general population across all the HOLC mapped zones (equivalently, people in the floodplain are 24% more likely to live in the red zone than the general population across all HOLC zones.
12	Black in floodplain to be in zone	1.50	1.00	Blacks who reside in the red zone are 50%t more likely to live <1m as Blacks across all HOLC zones.
13	People in floodpl to be Black & in zone	2.02	1.34	People who are both Black and in the red zone are 2.02 times as likely to be in the floodplain as the general population of all races across all HOLC zones.
14	Black Residents to be in this zone	1.20	1.00	Blacks are 20 percent more likely to live in the red zone than the general population of all HOLC mapped areas in counties with at least one person living below 1m.
**Total Desegregation Counterfactual: County-By-County**				
	Black Coastal Population	1,316	4201	If each county had the same racial composition in all the HOLC zones, the population of blacks in the HOLC red zones of coastal counties would be 1,316,000 (as opposed to the actual estimate of 1,503,000).
	Black Residents in floodplain	47	121	If each county had the same racial composition in all the HOLC zones, the population of blacks <1m the HOLC red zones would be 47,000 (as opposed to the actual estimate of 62,000).
	Effect of segregation on Black pop	15	-6.7	If each county had the same racial composition in all the HOLC zones, the population of blacks <1m the HOLC red zones would be 15,000 less, and the population of blacks <1m across all HOLC zones would be 6,700 more.
	Same as above as a ratio	1.32	0.94	The effect of segregation within counties, reflected by the HOLC mapping is to increase the number of black residents living below 1 meter by 32% in red zones, and to decrease the number of black residents below 1m by 6% across all of the HOLC zones.
**Total Desegregation Counterfactual: Nationwide aggregation**				
The plain meanings are analogous to “Total Desegregation Counterfactual: County-By-County” , except substitute “nation” for “county”.				

Table of Abbreviations Used in these Tables							
**States in these Tables**							
Abbreviation	Meaning		Abbreviation	Meaning		Abbreviation	Meaning
AL	Alabama		MA	Massachusetts		OK	Oklahoma
AR	Arkansas		MD	Maryland		OR	Oregon
CA	California		ME	Maine		PA	Pennsylvania
CO	Colorado		MN	Minnesota		RI	Rhode Island
CT	Connecticut		MO	Missouri		SC	South Carolina
DE	Delaware		MS	Mississippi		SD	South Dakota
FL	Florida		MT	Montana		TN	Tennessee
IA	Iowa		NC	North Carolina		TX	Texas
IL	Illinois		ND	North Dakota		VA	Virginia
IN	Indiana		NE	Nebraska		WA	Washington
KS	Kansas		NJ	New Jersey		WI	Wisconsin
KY	Kentucky		NV	Nevada		WV	West Virginia
LA	Louisiana		OH	Ohio			
**Other**							
A-zone	FEMA mapped floodplains generally having a 1% chance of flooding each year.						
Below one meter	Less than one meter above mean higher high water						
HOLC	Home Owners Loan Corporation, the government entity that created the redline maps.						
MHHW	Mean Higher High Water						
Sea level	Mean Higher High Water						
V-zone	FEMA mapped floodplains where property may be subjected to waves or strong currents.						
X500 Zone	FEMA mapped floodplains generally having a 0.2% chance of flooding each year.						

L

References

Federal Emergency Management Agency 2021 National flood hazard layer (available at: www.fema.gov/flood-maps/national-flood-hazard-layer) (Accessed 12 December 2021)

National Flood Insurance Rate maps (which form the basis of the National Flood Hazard Layer) . https://www.fema.gov/flood-maps/national-flood-hazard-layer

Nelson, R.K., Winling, L., Marciano, R., Connolly, N. and Ayers, E.L., 2021. Mapping inequality. *American panorama*.

Strauss et al., 2012. "Tidally adjusted estimates of topographic vulnerability to sea level rise and flooding for the contiguous United States." Environmental Research Letters 7, no. 1 (2012): 014033

Titus, J.G., 2023. Population in floodplains or close to sea level increased in US but declined in some counties—especially among Black residents. *Environmental Research Letters*, *18*(3), p.034001. https://doi.org/10.1088/1748-9326/acadf5

Titus, J.G., 2023. Supplemental Tables (Part 1: Portrait) from 'Population in floodplains or close to sea level increased in US but declined in some counties–especially among Black residents'. Environmental Research Letters. erlacadf5supp4.pdf (cfn-live-content-bucket-iop-org.s3.amazonaws.com).

Titus, J. G., et al. 2009. State and local governments plan for development of most land vulnerable to rising sea level along the US Atlantic coast. Environmental Research Letters, 4(4), 044008.

Zavar, E. and Fischer, L.A., 2021. Fractured landscapes: The racialization of home buyout programs and climate adaptation. *Current Research in Environmental Sustainability*, *3*, p.100043.
