# Supplementary material for: Population in floodplains or close to sea level increased in US but declined in some counties—especially among Black residents
Source: Environ Res Lett. Author manuscript; Available in PMC 2025 Mar 14. (PMC11908447; doi:10.1088/1748-9326/acadf5)
Supplement: County Population below One Meter [file NIHMS1876714-supplement-County_Population_below_One_Meter.pdf]

Supplemental County Population Tables from ‘Population in floodplains or close to sea level increased in US but declined in some counties–especially among Black residents’

JAMES G TITUS

ENVIRONMENTAL RESEARCH LETTERS

FEBRUARY 2023

Note: This document contains one of many county population tables created in the analysis described in the aforementioned paper in Environmental Research Letters. To obtain the other tables, maps, or underlying data whence those maps were created, go to EPA’s Science Hub at https://doi.org/10.23719/1527848

Population of land less than one meter above mean higher high water

	COUNTY	1990	2000	2010	2020	Change 1990s	2000s	2010s	1990-2020	since 2000
**005**	Cumberland County	280	366	246	310	86	-120	64	30	-56
**009**	Hancock County	111	119	109	87	7	-10	-22	-25	-32
**011**	Kennebec County	7	6	11	8	0	5	-3	2	2
**013**	Knox County	144	144	126	102	1	-19	-23	-42	-42
**015**	Lincoln County	116	114	107	102	-2	-7	-5	-14	-12
**019**	Penobscot County	7	8	3	6	2	-6	3	-1	-3
**023**	Sagadahoc County	107	119	125	146	12	6	21	38	26
**027**	Waldo County	16	9	12	14	-7	3	2	-2	5
**029**	Washington County	65	72	68	51	7	-4	-17	-14	-21
**031**	York County	604	734	649	739	130	-84	89	135	5
**ME**	Maine	1,457	1,692	1,456	1,565	236	-236	109	108	-127

For estimates of population in floodplains, below other elevations, or Black and Hispanic population, go to https://doi.org/10.1088/1748-9326/acadf5 or https://doi.org/10.23719/1527848

Population of land less than one meter above mean higher high water

COUNTY	1990	2000	2010	2020	Change 1990s	2000s	2010s	1990-2020	since 2000
Rockingham County	1,766	2,228	2,137	2,499	462	-91	362	733	271
Strafford County	22	20	26	43	-2	5	17	21	23
New Hampshire	1,788	2,248	2,162	2,542	460	-86	380	754	294

Population of land less than one meter above mean higher high water

	COUNTY	1990	2000	2010	2020	Change 1990	2000s	2010s	1990-2020	since 2000
**001**	Barnstable County	1,293	1,560	1,331	1,798	267	-229	467	505	238
**005**	Bristol County	849	899	945	1,224	50	46	280	376	325
**007**	Dukes County	76	80	122	144	4	41	22	68	64
**009**	Essex County	3,515	4,207	4,502	5,406	692	295	904	1,891	1,199
**017**	Middlesex County	8,361	9,775	12,105	16,068	1,413	2,330	3,963	7,707	6,293
**019**	Nantucket County	139	218	116	152	79	-102	36	13	-66
**021**	Norfolk County	3,951	4,159	4,056	4,858	208	-103	802	907	699
**023**	Plymouth County	4,548	5,186	4,949	5,227	638	-237	277	679	41
**025**	Suffolk County	39,693	44,982	49,488	58,996	5,288	4,507	9,508	19,303	14,015
**MA**	Massachusetts	62,426	71,066	77,614	93,873	8,640	6,548	16,260	31,448	22,807

ttps://doi.org/10.1088/1748-9326/acadf5 or https://doi.org/10.23719/1527848

Population of land less than one meter above mean higher high water

COUNTY	1990	2000	2010	2020	Change	PCTchange
Bristol County	405.1347	421.8015	500.9852	549.9277	144.7930	35.7395
Kent County	112.8605	141.5706	156.7388	186.7323	73.8718	65.4541
Newport County	494.1565	516.3017	436.6838	473.7038	-20.4527	-4.1389
Providence County	22.2604	39.5737	15.4973	73.6435	51.3831	-9,999.0000
Washington County	531.1967	642.3726	563.2152	582.3097	51.1130	9.6222
Rhode Island	1,565.6088	1,761.6201	1,673.1202	1,866.3169	300.7081	19.2071

Population of land less than one meter above mean higher high water

COUNTY	1990	2000	2010	2020	Change 199(	2000s	2010s	1990-2020	since 2000
Fairfield County	5,983	6,618	7,145	8,628	635	527	1,483	2,645	2,010
Hartford County	0	0	0	0	0	0	0	0	0
Middlesex County	484	543	597	730	59	54	134	247	188
New Haven County	3,196	3,237	3,430	3,383	41	193	-47	187	146
New London County	895	723	743	775	-172	20	31	-120	52
Connecticut	10,557	11,120	11,915	13,517	563	795	1,601	2,959	2,397

Population of land less than one meter above mean higher high water

COUNTY	1990	2000	2010	2020	Change 199(	2000s	2010s	1990-2020	since 2000
Albany County	1	1	1	1	0	0	0	0	0
Bronx County	461	839	755	909	378	-84	154	447	70
Columbia County	16	12	34	39	-4	22	5	23	27
Dutchess County	32	34	110	48	1	76	-62	16	14
Greene County	26	44	37	36	18	-7	-2	10	-8
Kings County	7,348	9,680	10,876	14,803	2,332	1,196	3,927	7,455	5,123
Nassau County	22,415	24,840	26,364	29,109	2,425	1,524	2,745	6,694	4,269
New York County	4,202	4,877	7,462	13,388	675	2,585	5,926	9,186	8,512
Orange County	31	28	1	7	-3	-26	5	-24	-21
Putnam County	8	9	20	19	1	11	-1	11	10
Queens County	13,431	15,128	16,538	21,501	1,697	1,410	4,963	8,070	6,373
Rensselaer County	3	3	2	1	-1	-1	-1	-2	-2
Richmond County	3,515	4,645	6,035	5,807	1,129	1,390	-228	2,292	1,163
Rockland County	281	391	406	319	110	14	-86	38	-72
Saratoga County	0	0	0	0	0	0	0	0	0
Suffolk County	14,520	15,888	16,383	18,223	1,368	495	1,840	3,703	2,335
Ulster County	55	107	128	201	53	20	74	147	94
Westchester County	598	565	536	779	-34	-29	242	180	214
New York	66,944	77,090	85,687	105,189	10,146	8,597	19,502	38,245	28,099

ttps://doi.org/10.1088/1748-9326/acadf5 or https://doi.org/10.23719/1527848

Population of land less than one meter above mean higher high water

COUNTY	1990	2000	2010	2020	Change	PCTchange		Change 199(
Atlantic Co	18,096	22,204	24,037	24,514	6417.541	35.46385		4,108
Bergen Cou	8,357	9,331	10,185	11,966	3608.657	43.18066		974
Burlington	1,795	1,890	1,943	2,101	305.478	17.01467		95
Camden Co	684	917	860	1,237	553.4191	80.95958		234
Cape May (	14,937	17,075	15,623	16,124	1186.528	7.943464		2,138
Cumberlan	1,122	1,156	1,224	1,249	126.9575	11.31788		34
Essex Coun	500	597	445	868	367.9549	73.54149		97
Gloucester	1,557	1,556	1,563	1,613	56.21215	3.610064		-1
Hudson Co	13,951	17,162	22,256	32,919	18968.16	135.9608		3,211
Mercer Cou	0	1	8	8	7.6715	-9999		1
Middlesex	762	579	836	831	68.27099	8.955757		-184
Monmouth	9,134	10,203	10,207	11,028	1894.379	20.73963		1,069
Morris Cou	0	0		0	0	-9999		0
Ocean Cou	11,802	14,905	15,138	16,855	5052.804	42.81271		3,103
Passaic Cou	1	2	3	6	4.260235	-9999		0
Salem Cour	7,928	7,794	7,695	8,086	158.3184	1.997073		-133
Somerset C	0	0	0	0	0	-9999		0
Union Cour	158	75	116	163	4.975176	3.149309		-83
New Jersey	90,785	105,449	112,138	129,567	38781.59	42.71793		14,663

ttps://doi.org/10.1088/1748-9326/acadf5 or https://doi.org/10.23719/1527848

Population of land less than one meter above mean higher high water

2000s	2010s	1990-2020	since 2000
1,832	477	6,418	2,309
855	1,780	3,609	2,635
53	158	305	210
-58	377	553	320
-1,452	501	1,187	-951
68	25	127	93
-153	424	368	271
7	51	56	57
5,094	10,664	18,968	15,757
7	0	8	7
257	-5	68	252
3	822	1,894	825
0	0	0	0
232	1,717	5,053	1,950
2	2	4	4
-99	391	158	292
0	0	0	0
41	47	5	88
6,689	17,429	38,782	24,118

ttps://doi.org/10.1088/1748-9326/acadf5 or https://doi.org/10.23719/1527848

Population of land less than one meter above mean higher high water

COUNTY	1990	2000	2010	2020	Change 1990s	2000s	2010s	1990-2020	since 2000
Bucks County	141	107	103	147	-34	-3	44	7	40
Delaware County	195	209	216	254	15	6	38	59	44
Montgomery County	0	0	0	2	0	0	2	2	2
Northampton County	0	0	0	0	0	0	0	0	0
Philadelphia County	1,777	1,068	1,389	1,176	-709	321	-214	-602	108
Pennsylvania	2,113	1,384	1,709	1,579	-728	324	-130	-534	194

ttps://doi.org/10.1088/1748-9326/acadf5 or https://doi.org/10.23719/1527848

Population of land less than one meter above mean higher high water

COUNTY	1990	2000	2010	2020
Kent County	719	755	806	801
New Castle Count	947	676	974	1,134
Sussex County	2,728	5,242	6,349	7,558
Delaware	4,394	6,673	8,129	9,493

Change 1990s	2000s	2010s	1990-2020	since 2000
37	51	-5	83	46
-271	299	160	187	458
2,514	1,107	1,210	4,830	2,316
2,279	1,456	1,364	5,100	2,821

ttps://doi.org/10.1088/1748-9326/acadf5 or https://doi.org/10.23719/1527848

Population of land less than one meter above mean higher high water

	COUNTY	1990	2000	2010	2020	Change 1990s	2000s	2010s	1990-2020
**001**		0				0	0	0	0
**003**	Anne Arundel County	1,860	2,158	2,361	2,350	298	203	-10	490
**005**	Baltimore County	1,780	2,017	2,362	2,549	237	345	187	769
**009**	Calvert County	598	782	934	897	184	152	-37	299
**011**	Caroline County	117	116	110	102	-2	-5	-8	-15
**015**	Cecil County	172	187	250	274	15	63	24	102
**017**	Charles County	131	157	182	166	26	25	-16	35
**019**	Dorchester County	3,574	3,707	3,491	3,318	133	-216	-173	-256
**025**	Harford County	664	370	72	82	-294	-298	10	-582
**027**	Howard County	0	0	0	0	0	0	0	0
**029**	Kent County	229	285	248	276	56	-37	28	47
**031**	Montgomery County	0	0	0	0	0	0	0	0
**033**	Prince George's County	10	9	10	3	-1	1	-7	-7
**035**	Queen Anne's County	661	873	1,190	1,344	212	316	155	683
**037**	St. Mary's County	872	908	1,003	1,143	37	94	140	272
**039**	Somerset County	6,834	6,825	6,625	6,380	-9	-200	-245	-454
**041**	Talbot County	891	923	885	859	32	-38	-26	-32
**045**	Wicomico County	356	418	407	569	62	-12	162	213
**047**	Worcester County	2,036	3,248	3,887	4,380	1,212	640	493	2,344
**510**	Baltimore city	113	289	481	796	176	192	315	683
**MD**	Maryland	20,900	23,274	24,499	25,491	2,374	1,226	991	4,590

ttps://doi.org/10.1088/1748-9326/acadf5 or https://doi.org/10.23719/1527848

Population of land less than one meter above mean higher high water

since 2000
0
193
532
115
-13
87
9
-389
-288
0
-9
0
-6
471
235
-445
-64
151
1,133
507
2,217

ttps://doi.org/10.1088/1748-9326/acadf5 or https://doi.org/10.23719/1527848

	FIPSSTCO	COUNTY	1990	2000	2010	2020
**001**	11001	District of Columbia	225.0742	99.99075	329.8087	527.0123
**DC**			225.0742	99.99075	329.8087	527.0123

Population of land less than one meter above mean higher high water

COUNTY	1990	2000	2010	2020	Change 1990	2000s	2010s	1990-2020	since 2000
Accomack County	4,862	5,638	4,808	5,010	776	-830	202	148	-628
Arlington County	1	7	3	32	6	-4	29	31	25
Caroline County	0	0	0	0	0	0	0	0	0
Charles City County	16	17	15	15	0	-2	1	-1	-2
Chesterfield County	8	11	12	10	4	1	-3	2	-2
Dinwiddie County	0	0	0	0	0	0	0	0	0
Essex County	79	123	94	134	44	-29	39	54	11
Fairfax County	83	85	141	177	2	56	36	95	93
Gloucester County	1,748	1,760	1,647	1,767	12	-113	119	19	7
Greensville County	0	0	0	0	0	0	0	0	0
Hanover County	3	2	1	1	-1	0	-1	-2	-1
Henrico County	3	3	3	2	-1	0	-1	-1	-1
Isle of Wight County	68	86	101	58	17	16	-44	-10	-28
James City County	85	110	136	157	25	26	21	72	47
King and Queen Cou	27	37	40	49	10	3	9	22	12
King George County	45	44	60	15	-1	16	-45	-30	-29
King William County	93	106	95	116	13	-11	21	23	10
Lancaster County	359	428	390	389	69	-37	-1	31	-38
Loudoun County	0	0	0	0	0	0	0	0	0
Mathews County	1,315	1,594	1,530	1,585	279	-64	54	269	-9
Middlesex County	164	192	219	179	27	28	-40	15	-13
New Kent County	21	26	39	43	5	12	4	22	17
Northampton Count	165	207	192	217	42	-15	26	52	10
Northumberland Co	290	387	302	255	98	-86	-47	-35	-133
Prince George Count	2	2	2	3	1	0	1	2	1
Prince William Coun	9	8	13	29	-1	5	16	20	21
Richmond County	27	34	37	43	7	3	7	17	9
Southampton Count	10	16	16	19	6	0	3	9	3
Spotsylvania County	0	0	0	0	0	0	0	0	0
Stafford County	33	41	54	37	8	13	-17	4	-4
Surry County	48	52	46	49	4	-5	3	2	-3

ttps://doi.org/10.1088/1748-9326/acadf5 or https://doi.org/10.23719/1527848

Population of land less than one meter above mean higher high water

Westmoreland Coun	150	168	180	134	18	12	-46	-16	-34
York County	856	1,020	1,238	1,412	164	218	174	556	392
Alexandria city	118	204	131	179	86	-73	48	61	-25
Chesapeake city	1,461	1,931	2,541	3,578	469	610	1,037	2,117	1,647
Colonial Heights city	5	7	6	6	2	0	0	2	0
Franklin city	0	0	0	0	0	0	0	0	0
Fredericksburg city	0	0	0	0	0	0	0	0	0
Hampton city	1,957	2,887	2,483	2,937	930	-404	454	980	50
Hopewell city	3	3	9	14	0	6	5	10	11
Newport News city	645	573	526	452	-73	-46	-74	-193	-120
Norfolk city	5,959	5,953	6,262	7,850	-5	309	1,588	1,891	1,897
Petersburg city	1	1	0	0	0	0	0	0	0
Poquoson city	2,327	2,730	3,321	3,835	404	591	514	1,509	1,105
Portsmouth city	1,133	1,340	1,017	1,379	206	-323	363	246	40
Richmond city	0	0	0	0	0	0	0	0	0
Suffolk city	57	87	136	74	31	48	-61	18	-13
Virginia Beach city	2,265	2,799	3,310	3,632	534	512	321	1,367	833
Williamsburg city	0	0	0	0	0	0	0	0	0
Virginia	26,501	30,717	31,159	35,876	4,216	442	4,716	9,375	5,159

ttps://doi.org/10.1088/1748-9326/acadf5 or https://doi.org/10.23719/1527848

Population of land less than one meter above mean higher high water

COUNTY	1990	2000	2010	2020	Change 1990	2000s	2010s	1990-2020	since 2000
Beaufort County	3,783	3,587	3,677	3,484	-196	89	-193	-299	-103
Bertie County	30	35	39	32	5	4	-6	2	-2
Bladen County	0	0	0	0	0	0	0	0	0
Brunswick County	714	855	925	1,430	141	69	505	716	575
Camden County	324	431	613	637	107	182	24	313	206
Carteret County	4,649	5,039	5,230	5,547	390	191	317	898	508
Chowan County	117	132	145	148	15	13	3	31	16
Columbus County	0	0	0	0	0	0	0	0	0
Craven County	241	276	340	314	35	64	-26	73	38
Currituck County	1,124	1,479	1,830	2,310	355	351	479	1,186	830
Dare County	3,359	4,114	5,006	5,494	755	892	488	2,135	1,380
Duplin County	0	0	0	0	0	0	0	0	0
Edgecombe County	0	0	0	0	0	0	0	0	0
Gates County	29	44	51	59	15	7	8	29	15
Greene County	0	0	0	0	0	0	0	0	0
Halifax County	0	0	0	1	0	0	1	1	1
Hertford County	73	82	58	61	9	-24	3	-12	-21
Hyde County	3,812	3,770	3,657	3,036	-42	-113	-621	-776	-734
Jones County	4	6	5	4	2	0	-1	0	-2
Lenoir County	0	0	0	0	0	0	0	0	0
Martin County	2	1	5	5	0	4	0	3	4
Nash County	0	0	0	0	0	0	0	0	0
New Hanover County	1,813	1,984	2,288	2,554	170	304	266	741	570
Northampton County	0	0	0	0	0	0	0	0	0
Onslow County	766	804	757	1,246	37	-47	488	479	442
Pamlico County	1,102	1,027	984	888	-75	-43	-97	-214	-139
Pasquotank County	1,312	1,393	1,547	1,616	81	154	69	304	223
Pender County	312	443	531	732	131	88	201	420	289
Perquimans County	163	202	262	264	40	59	3	102	62
Pitt County	5	6	8	11	1	2	3	6	5
Richmond County	0	0	0	0	0	0	0	0	0

ttps://doi.org/10.1088/1748-9326/acadf5 or https://doi.org/10.23719/1527848

Population of land less than one meter above mean higher high water

Sampson County	0	0	0	0	0	0	0	0	0
Tyrrell County	2,295	2,642	2,996	2,030	348	353	-966	-265	-613
Washington County	247	232	259	199	-15	27	-60	-47	-33
North Carolina	26,277	28,585	31,213	32,101	2,308	2,628	888	5,824	3,516

ttps://doi.org/10.1088/1748-9326/acadf5 or https://doi.org/10.23719/1527848

Population of land less than one meter above mean higher high water

COUNTY	1990	2000	2010	2020	Change 1990s	2000s	2010s	1990-2020	since 2000
Beaufort County	13,761	15,256	17,178	18,993	1,495	1,922	1,814	5,232	3,736
Berkeley County	252	263	535	1,216	11	272	680	964	952
Charleston County	14,377	17,395	20,921	24,785	3,018	3,526	3,865	10,408	7,391
Clarendon County	0	0	0	0	0	0	0	0	0
Colleton County	343	624	454	957	282	-171	503	614	332
Dorchester County	23	26	24	54	3	-1	30	32	29
Florence County	0	0	0	0	0	0	0	0	0
Georgetown County	3,273	3,511	3,760	4,168	238	249	407	894	656
Hampton County	0	0	0	0	0	0	0	0	0
Horry County	1,049	1,086	1,155	1,586	37	69	431	537	500
Jasper County	57	90	135	195	33	45	61	139	106
Marion County	0	0	1	0	0	1	-1	0	0
Orangeburg County	0	0	0	0	0	0	0	0	0
Williamsburg County	0	0	0	0	0	0	0	0	0
South Carolina	33,134	38,252	44,164	51,954	5,118	5,912	7,790	18,820	13,702

ttps://doi.org/10.1088/1748-9326/acadf5 or https://doi.org/10.23719/1527848

Population of land less than one meter above mean higher high water

COUNTY	1990	2000	2010	2020	Change 199	2000s	2010s	1990-2020	since 2000
Brantley County	0	0	0	0	0	0	0	0	0
Bryan County	92	171	179	422	79	7	243	330	251
Camden County	691	1,009	854	1,019	318	-155	165	328	9
Charlton County	0	0	0	0	0	0	0	0	0
Chatham County	4,092	5,747	6,341	8,644	1,655	594	2,304	4,552	2,897
Effingham County	0	0	0	0	0	0	0	0	0
Glynn County	6,001	6,542	7,310	8,508	542	767	1,199	2,508	1,966
Liberty County	301	398	421	509	97	23	88	208	111
Long County	0	0	0	0	0	0	0	0	0
McIntosh County	201	234	407	281	33	173	-126	80	47
Screven County	0	0	0	0	0	0	0	0	0
Wayne County	1	0	0	0	-1	0	0	-1	0
Georgia	11,379	14,102	15,511	19,384	2,723	1,409	3,872	8,005	5,282

ttps://doi.org/10.1088/1748-9326/acadf5 or https://doi.org/10.23719/1527848

Population of land less than one meter above mean higher high water

COUNTY	1990	2000	2010	2020	Change 1990s	2000s	2010s	1990-2020	since 2000
Brevard County	8,062	9,384	10,537	11,535	1,322	1,153	998	3,473	2,151
Broward County	101,401	120,988	136,608	172,398	19,587	15,619	35,791	70,997	51,410
Clay County	586	913	1,003	1,140	326	90	137	554	227
	175,922				-175,922	0	0	-175,922	0
Duval County	7,933	9,275	9,119	10,556	1,342	-156	1,437	2,623	1,281
Flagler County	1,451	2,270	2,517	2,982	819	247	465	1,531	712
Indian River County	1,852	2,164	2,432	3,000	312	268	568	1,148	836
Lake County	483	520	524	623	37	5	98	140	103
Marion County	3	7	5	4	3	-2	-1	1	-3
Martin County	2,801	3,459	3,853	4,273	658	394	420	1,472	814
Miami-Dade County	175,922	192,654	222,719	259,118	16,732	30,065	36,400	83,196	66,465
Monroe County	49,431	53,288	49,952	57,820	3,857	-3,336	7,869	8,389	4,532
Nassau County	801	1,355	1,775	1,973	553	420	198	1,172	618
Okeechobee County	0	0	0	0	0	0	0	0	0
Orange County	1	0	0	0	-1	0	0	-1	0
Osceola County	0	0	0	0	0	0	0	0	0
Palm Beach County	14,308	17,371	19,810	23,334	3,064	2,439	3,524	9,026	5,963
Putnam County	1,227	1,308	1,309	1,401	80	1	92	174	93
St. Johns County	9,065	14,240	15,203	17,676	5,174	964	2,472	8,610	3,436
St. Lucie County	5,193	6,444	6,702	7,587	1,251	259	885	2,394	1,143
Seminole County	109	152	168	227	43	16	58	117	74
Volusia County	7,505	8,371	8,898	9,545	867	527	648	2,041	1,174
Atlantic Florrida	388,137	444,163	493,134	585,192	56,026	48,971	92,058	197,055	141,029

ttps://doi.org/10.1088/1748-9326/acadf5 or https://doi.org/10.23719/1527848

Population of land less than one meter above mean higher high water

COUNTY	1990	2000	2010	2020	Change 199	2000s	2010s	1990-2020	since 2000
Bay County	1,364	1,440	1,495	1,292	76	55	-203	-72	-148
Calhoun County	0	0	0	0	0	0	0	0	0
Charlotte County	8,526	9,610	11,019	13,214	1,084	1,408	2,196	4,688	3,604
Citrus County	3,906	3,889	4,020	3,958	-17	131	-63	52	69
Collier County	10,047	14,064	14,929	18,350	4,017	865	3,421	8,302	4,286
Columbia County	0	0	0	0	0	0	0	0	0
DeSoto County	21	45	34	59	24	-11	25	38	14
Dixie County	684	643	583	544	-42	-59	-39	-140	-99
Escambia County	914	957	930	1,129	43	-26	199	215	172
Franklin County	303	321	310	446	18	-11	136	143	125
Gilchrist County	2	4	4	4	3	0	0	2	-1
Glades County	0	1	0	1	1	-1	1	1	0
Gulf County	105	100	142	181	-5	43	39	76	81
Hardee County	0	0	0	0	0	0	0	0	0
Hendry County	54	70	130	119	15	60	-10	65	50
Hernando County	1,484	1,636	1,655	1,758	152	19	103	274	122
Highlands County	0	0	0	0	0	0	0	0	0
Hillsborough Count	6,157	7,081	7,832	9,716	924	751	1,884	3,559	2,635
Holmes County			0	0	0	0	0	0	0
Jackson County	0	0	0	0	0	0	0	0	0
Jefferson County	2	0	0	4	-2	0	4	2	4
Lafayette County	0	0	0	0	0	0	0	0	0
Lee County	19,451	24,413	27,382	29,829	4,962	2,969	2,447	10,378	5,416
Leon County	0	0	0	0	0	0	0	0	0
Levy County	526	542	535	557	17	-8	23	31	15
Liberty County	0	0	0	0	0	0	0	0	0
Manatee County	15,244	17,307	16,184	16,818	2,062	-1,123	634	1,574	-488
Okaloosa County	755	881	986	768	126	106	-219	13	-113
Pasco County	6,264	7,095	7,592	9,049	831	497	1,457	2,786	1,954
Pinellas County	35,532	39,723	41,532	43,872	4,191	1,809	2,340	8,341	4,150
Polk County	0	0	0	0	0	0	0	0	0

ttps://doi.org/10.1088/1748-9326/acadf5 or https://doi.org/10.23719/1527848

Population of land less than one meter above mean higher high water

Santa Rosa County	705	1,089	1,252	1,227	384	163	-25	522	138
Sarasota County	10,383	10,818	10,966	12,160	435	149	1,194	1,778	1,343
Suwannee County	0	0	0	0	0	0	0	0	0
Taylor County	272	357	364	363	85	7	-1	91	6
Wakulla County	365	527	473	488	162	-53	14	123	-39
Walton County	376	766	1,133	1,732	390	367	599	1,356	965
Washington County	3	3	3	3	0	0	0	0	0
Gulf Florida	123,445	143,382	151,488	167,641	19,936	8,106	16,153	44,195	24,259

ttps://doi.org/10.1088/1748-9326/acadf5 or https://doi.org/10.23719/1527848

Population of land less than one meter above mean higher high water

COUNTY	1990	2000	2010	2020	Change 1990	2000s	2010s	1990-2020	since 2000
Baldwin County	2,298	3,404	3,712	4,387	1,106	308	675	2,089	983
Choctaw County	0	0	0	0	0	0	0	0	0
Clarke County	0	0	0	0	0	0	0	0	0
Mobile County	1,189	1,443	1,245	1,527	254	-198	282	338	84
Monroe County	0	0	0	0	0	0	0	0	0
Washington County	2	1	2	1	-1	1	-1	-1	0
Alabama	3,489	4,848	4,959	5,915	1,359	111	956	2,426	1,067

ttps://doi.org/10.1088/1748-9326/acadf5 or https://doi.org/10.23719/1527848

Population of land less than one meter above mean higher high water

COUNTY	1990	2000	2010	2020	Change 1990s	2000s	2010s	1990-2020	since 2000
Hancock County	1,108	2,021	1,311	1,433	913	-710	122	326	-588
Harrison County	441	723	547	639	282	-176	92	198	-84
Jackson County	1,054	1,353	1,003	1,003	299	-350	0	-51	-350
Pearl River County	0	0	0	0	0	0	0	0	0
Mississippi	2,603	4,097	2,861	3,076	1,494	-1,236	215	473	-1,021

ttps://doi.org/10.1088/1748-9326/acadf5 or https://doi.org/10.23719/1527848

Population of land less than one meter above mean higher high water

COUNTY	1990	2000	2010	2020	Change 1990s	2000s	2010s	1990-2020	since 2000
Aransas County	291	431	427	442	140	-4	15	151	11
Brazoria County	10,159	11,796	11,510	10,955	1,637	-285	-556	796	-841
Calhoun County	59	108	94	105	49	-14	11	46	-3
Cameron County	399	808	1,017	784	409	209	-233	385	-24
Chambers County	64	91	57	106	27	-33	48	41	15
Fort Bend County	0	0	0	0	0	0	0	0	0
Galveston County	5,254	7,276	7,556	10,455	2,022	280	2,899	5,200	3,179
Hardin County	0	0	0	0	0	0	0	0	0
Harris County	740	613	541	865	-127	-73	325	125	252
Hidalgo County	0	0	0	0	0	0	0	0	0
Jackson County	3	3	1	2	0	-1	1	-1	-1
Jasper County	0	0	0	0	0	0	0	0	0
Jefferson County	31,340	30,657	29,033	29,963	-683	-1,624	930	-1,377	-694
Jim Wells County	0	0	0	0	0	0	0	0	0
Kenedy County	0	0	0	0	0	0	0	0	0
Kleberg County	5	0	0	0	-4	0	0	-4	0
Liberty County	5	5	3	6	0	-2	2	0	0
Matagorda County	218	281	292	324	63	12	31	106	43
Newton County	0	0	0	0	0	0	0	0	0
Nueces County	821	1,210	1,602	1,570	390	391	-32	749	359
Orange County	223	280	276	362	58	-4	86	140	82
Refugio County	1	0	2	2	-1	1	0	1	2
San Patricio County	105	182	212	228	77	30	17	123	46
Victoria County	0	0	0	1	0	0	1	1	1
Willacy County	6	16	12	13	10	-3	1	8	-2
Texas	49,692	53,758	52,637	56,182	4,066	-1,121	3,545	6,490	2,424

ttps://doi.org/10.1088/1748-9326/acadf5 or https://doi.org/10.23719/1527848

Population of land less than one meter above mean higher high water

COUNTY	1990	2000	2010	2020	Change 1990s	2000s	2010s	1990-2020	since 2000
Acadia Parish	5	5	12	17	-1	7	5	11	12
Allen Parish	0	0	0	0	0	0	0	0	0
Ascension Parish	1,215	2,065	2,927	3,325	850	862	398	2,110	1,261
Assumption Parish	4,210	5,124	6,021	5,934	914	897	-87	1,724	809
Beauregard Parish	0	0	0	0	0	0	0	0	0
Calcasieu Parish	636	830	887	1,154	194	57	268	518	325
Cameron Parish	3,290	3,498	1,673	1,523	208	-1,825	-149	-1,767	-1,975
East Baton Rouge Parish	5	3	6	8	-2	2	2	3	4
Iberia Parish	840	962	949	1,155	122	-13	206	315	193
Iberville Parish	427	603	696	729	176	93	33	302	126
Jefferson Parish	386,936	397,082	380,815	391,888	10,146	-16,267	11,073	4,952	-5,194
Jefferson Davis Parish	181	179	186	229	-2	7	43	48	50
Lafayette Parish	6	7	5	5	1	-2	0	-1	-2
Lafourche Parish	36,698	39,833	44,030	42,766	3,134	4,197	-1,263	6,068	2,934
Livingston Parish	1,300	1,568	2,351	2,431	269	783	80	1,131	863
Orleans Parish	424,467	416,604	289,402	333,352	-7,862	-127,202	43,949	-91,115	-83,253
Plaquemines Parish	23,287	24,495	20,914	21,610	1,208	-3,581	696	-1,677	-2,885
Pointe Coupee Parish	0	0	0	0	0	0	0	0	0
St. Bernard Parish	50,256	51,546	27,165	34,743	1,290	-24,381	7,578	-15,513	-16,803
St. Charles Parish	15,793	20,027	22,553	23,875	4,234	2,526	1,323	8,083	3,849
St. James Parish	1,104	1,341	1,580	1,722	237	240	142	618	382
St. John the Baptist Parish	450	645	1,048	1,425	195	403	377	975	780
St. Landry Parish	0	0	0	0	0	0	0	0	0
St. Martin Parish	988	1,234	1,234	1,244	246	0	10	256	10
St. Mary Parish	19,020	18,884	21,563	21,632	-136	2,679	69	2,612	2,748
St. Tammany Parish	2,902	3,628	4,941	6,961	726	1,313	2,020	4,059	3,333
Tangipahoa Parish	190	185	193	341	-5	8	148	151	156
Terrebonne Parish	31,332	33,659	38,169	38,894	2,326	4,510	725	7,561	5,235
Vermilion Parish	2,304	2,527	2,501	2,706	223	-26	205	402	179
West Baton Rouge Parish	2	6	6	6	3	0	0	4	0
Louisiana	1,007,845	1,026,540	871,828	939,677	18,695	-154,712	67,849	-68,168	-86,863

ttps://doi.org/10.1088/1748-9326/acadf5 or https://doi.org/10.23719/1527848

Population of land less than one meter above mean higher high water

COUNTY	1990	2000	2010	2020	Change 1990s	2000s	2010s	1990-2020	since 2000
Alameda County	880	1,029	1,042	1,155	149	13	113	275	126
Contra Costa County	2,495	2,401	2,583	3,215	-93	181	633	721	814
Del Norte County	0	0	0	0	0	0	0	0	0
Humboldt County	112	84	142	195	-29	59	53	83	111
Los Angeles County	1,264	1,314	1,350	1,095	50	36	-255	-169	-219
Marin County	6,368	9,861	10,345	10,787	3,492	485	441	4,418	926
Mendocino County	54	6	3	3	-48	-3	0	-51	-3
Monterey County	11	21	18	24	10	-3	6	13	3
Napa County	170	175	136	128	5	-39	-9	-42	-47
Orange County	20,083	19,578	19,501	20,016	-505	-77	515	-68	437
Sacramento County	2,969	3,043	3,489	3,664	74	446	175	695	621
San Diego County	395	1,026	6,525	1,423	631	5,499	-5,102	1,028	397
San Francisco County	116	110	128	251	-6	17	123	134	140
San Joaquin County	21,714	23,552	21,973	24,267	1,837	-1,578	2,294	2,553	716
San Luis Obispo County	8	3	3	2	-5	0	-1	-5	-1
San Mateo County	43,261	49,328	54,894	60,094	6,067	5,566	5,200	16,833	10,766
Santa Barbara County	14	18	6	12	4	-12	6	-2	-6
Santa Clara County	4,673	6,871	5,992	6,985	2,198	-879	994	2,312	114
Santa Cruz County	6	3	3	18	-3	0	15	12	15
Solano County	922	733	664	622	-189	-69	-42	-301	-111
Sonoma County	26	46	55	64	20	9	9	38	18
Ventura County	76	87	93	116	11	6	23	40	30
Yolo County	122	140	96	127	18	-45	32	5	-13
California	105,742	119,429	129,040	134,263	13,687	9,611	5,224	28,521	14,835

ttps://doi.org/10.1088/1748-9326/acadf5 or https://doi.org/10.23719/1527848

Population of land less than one meter above mean higher high water

COUNTY	1990	2000	2010	2020	Change 1990s	2000s	2010s	1990-2020	since 2000
Clackamas County	0	0	0	0	0	0	0	0	0
Clatsop County	503	448	413	462	-55	-35	49	-41	15
Columbia County	193	242	196	177	49	-45	-19	-15	-64
Coos County	171	150	163	179	-20	13	16	8	29
Curry County	4	5	2	6	1	-3	4	2	1
Douglas County	48	17	38	44	-31	21	6	-5	26
Lane County	33	23	6	14	-10	-17	8	-19	-9
Lincoln County	43	41	43	44	-2	2	1	1	3
Multnomah County	1,118	1,169	1,134	655	51	-35	-480	-464	-515
Tillamook County	8	11	13	12	3	2	-1	4	1
Oregon	2,120	2,106	2,008	1,592	-14	-98	-416	-528	-514

ttps://doi.org/10.1088/1748-9326/acadf5 or https://doi.org/10.23719/1527848

Population of land less than one meter above mean higher high water

COUNTY	1990	2000	2010	2020	Change 1990	2000s	2010s	1990-2020	since 2000
Clallam County	99	82	105	128	-17	23	23	29	46
Clark County	51	39	6	33	-12	-33	27	-18	-6
Cowlitz County	1,852	2,104	2,079	2,275	252	-26	197	423	171
Grays Harbor County	120	153	157	158	34	4	1	39	5
Island County	42	40	45	31	-2	5	-14	-11	-9
Jefferson County	37	33	33	25	-4	-1	-7	-12	-8
King County	222	267	203	186	45	-64	-18	-36	-82
Kitsap County	471	977	419	831	507	-559	412	360	-147
Mason County	58	48	35	41	-10	-13	6	-17	-7
Pacific County	25	22	22	25	-3	-1	4	0	3
Pierce County	278	466	350	263	188	-116	-88	-15	-203
San Juan County	30	38	29	33	8	-9	4	4	-4
Skagit County	906	935	1,053	1,149	29	118	96	243	214
Skamania County	0	0	0	0	0	0	0	0	0
Snohomish County	447	554	495	509	107	-59	14	62	-45
Thurston County	49	46	45	17	-3	-1	-27	-31	-29
Wahkiakum County	89	97	94	95	8	-3	1	6	-2
Whatcom County	8	10	7	8	2	-3	1	0	-2
Washington	4,783	5,912	5,176	5,808	1,128	-735	632	1,025	-104

ttps://doi.org/10.1088/1748-9326/acadf5 or https://doi.org/10.23719/1527848

Population of land less than one meter above mean higher high water

COUNTY	1990	2000	2010	2020	Change 1990	2000s	2010s	1990-2020	since 2000
Hawaii County	92	176	103	119	85	-74	17	28	-57
Honolulu County	10,619	10,547	14,600	16,319	-72	4,053	1,719	5,700	5,772
Kalawao County	0	0	0	0	0	0	0	0	0
Kauai County	189	226	460	516	37	234	56	327	290
Maui County	548	451	605	690	-97	154	86	143	240
Hawaii	11,447	11,400	15,767	17,645	-47	4,367	1,878	6,197	6,244

ttps://doi.org/10.1088/1748-9326/acadf5 or https://doi.org/10.23719/1527848

Bibliography

NOAA Sea level rise viewer DEM (available at: https://coast.noaa.gov/htdata/raster2/elevation/SLR_viewer_DEM_6230/)(Accessed 19 November 2021)

Manson S, Schroeder J, Van Riper D, Kugler T and Ruggles S 2021 IPUMS National Historical Geographic Information System: version 16.0 [dataset] (Minneapolis, MN) 10.18128/D050.V16.0

Titus, J.G., 2023a. Population in floodplains or close to sea level increased in US but declined in some counties—especially among Black residents. *Environmental Research Letters*, *18*(3), p.034001. https://doi.org/10.1088/1748-9326/acadf5

Titus, J.G., 2023b. Supplemental Methods from ‘Population in floodplains or close to sea level increased in US but declined in some counties–especially among Black residents. *Environmental Research Letters:* Supplementary Data
