# Supplementary material for: Population in floodplains or close to sea level increased in US but declined in some counties—especially among Black residents
Source: Environ Res Lett. Author manuscript; Available in PMC 2025 Mar 14. (PMC11908447; doi:10.1088/1748-9326/acadf5)
Supplement: Supplemental Methods [file NIHMS1876714-supplement-Supplemental_Methods.pdf]

SUPPLEMENTAL METHODS: POPULATION IN FLOODPLAINS OR CLOSE TO SEA LEVEL INCREASED IN US BUT DECLINED IN SOME COUNTIES–ESPECIALLY AMONG BLACK RESIDENTS:

JAMES G TITUS

ENVIRONMENTAL RESEARCH LETTERS

FEBRUARY 2023

SUPPLEMENTAL METHODS

Creation of these geographic population vulnerability indicators involves four tasks.

1. Define and map the vulnerability zone (i.e., hazard zone[1]),
2. Obtain data that maps the target population and how it is changing,
3. Overlay the two data sets to estimate the target population within the vulnerable zone, and
4. Calculate and display summary statistics at the state and county level.

This supplemental methods section describes those steps and lists the data sources. It also discusses two auxiliary presentations of the data for evaluating whether—and if so how--racial minorities disproportionately inhabit floodplains or land close to sea level:

5. A county-weighted ratio of disproportionality measuring whether—at the county level—Black and Hispanic people inhabit vulnerable areas more than people of other races, and
6. Overlays of the block vulnerability results with (a) the so-called "redline maps" originally commissioned by the federal Home Owners' Loan Corporation, and (b) an analysis of the likelihood of shore protection along the US Atlantic Coast conducted by EPA in the 2000s.

And finally,

7. Miscellaneous Caveats about these Methods
    - 7.1 Using 30-meter grid for elevation data
    - 7.2 Using building footprint data for a single year
    - 7.3 Accounting for Subsidence
    - 7.4 Ratio Estimator for Total Population Below 1m
    - 7.5 Limitations of the Study Design
8. Uncertainty and Statistical Significance.

At the outset, we had to decide whether to conduct the overlay analysis using polygon or cell-based calculations. Data on tidal water levels and land elevations are generally published in a raster format, with cell sizes on the order of 10 meters. But population data generally are associated with polygons such as city blocks, census tracts, and other enumeration districts whose boundaries follow natural features, streets, or other infrastructure. Thus an overlay requires us to either convert the elevation data to polygons, or convert the population data to a raster format.[2]

Our approach is to create polygon vulnerability zones from the raster data, rather than convert the population data to a raster format. A polygon representing the land below 1 meter, for example, can be defined with the same precision and accuracy as the cells themselves, while the information associated with the Census polygons is retained during the overlay. The alternative approach of

[1] This supplemental methods description uses the terms *zone of vulnerability, vulnerability zone, and hazard zone* interchangeably.

[2] The common data format is needed because the target variable that we are counting is associated with larger polygons. For some polygons, the raster data are used to measure the portion of the polygon in the vulnerable zone; that information is then used to estimate the portion of the polygon's population that is in the vulnerable zone. If the target variable was raster and vulnerable zone polygon, then putting the data in a common format would not be necessary because ordinary GIS tools can count the number of cells within a polygon.

converting population data to raster data requires an assumption about how to allocate the population of a polygon to the individual cells within that polygon. Although the common assumption that population is uniformly distributed within a given polygon may be reasonable in some cases, that is only one of several possible assumptions, and not especially realistic for land vulnerable to flooding because people generally avoid building in the most floodprone part of their land when possible.

Because the analysis of land close to sea level looks at both a changing vulnerability zone and changing population for four decadal censuses, we potentially have 16 combinations of year-specific results, so that it is possible to look at the effects of sea level rise and population changes separately. As a matter of notation, a variable "population_X_Y" here means vulnerable population using the census of year **X** given sea level for the year **Y**. To save processing time and data storage, we omit the two combinations of (2000,2010) and (2010,2000) entirely. Thus, we can hold either population or sea level constant to either their 1990 or 2020 values and examine the trend; but we cannot look at the entire trend while holding these values at their 2000 or 2010 values. Because we calculate population for four different vulnerability zones (e.g. 0m, 1m, 2m, and 3m), we potentially have 4 X 14 = 56 combinations of census year, sea level year, and vulnerability zone. To further save resources, we made calculations for all four vulnerability zones for the 10 combinations where either X=Y, X=2020, or Y=2020; but we only evaluate 1m for the remaining combinations (1990,2000), (1990, 2010), (2000,1990), and (2010,1990).

1. Define Vulnerability Zones

This analysis uses two classes of vulnerability zones: land close to sea level and floodplains. For each vulnerability class, we can define similar zones with different vulnerability, such as different elevations above sea level and different types of floodplains. Here we provide (1.1) an overview of the approach which expands on the description in the main paper, followed (1.2) the specific steps taken.

1.1 Overview.

Two widely distributed US elevation datasets are the USGS National Elevation Dataset[3] and NOAA's sea level rise viewer dataset.[4] Where possible, we use the latter data, which NOAA developed with a focus on coastal elevations. Although the NOAA data are available at a resolution of 10 meter, we resampled them to a 30-meter grid to save data storage and processing time. See Supplemental Methods §§1.1 & 7.1. Because the NOAA data do not extend far enough inland to capture all low land, we used the USGS data wherever the NOAA data are unavailable. We omit Alaska from the elevation analysis because data with sufficient vertical precision were not available.

The published datasets each provide a grid (raster) data layer with the elevation of each pixel relative to the fixed North American Vertical Datum of 1988 (NAVD). To define elevations relative to sea level, one must subtract a raster data layer that defines sea level in a given year relative to NAVD. NOAA publishes a raster layer known as VDatum[5], which quantifies the height of sea level relative to

---

[3] United States Geological Survey. 3 DEP Elevation Program. https://www.usgs.gov/core-science-systems/ngp/3dep (Accessed 11/20/2021).
[4] NOAA Sea Level Rise Viewer DEM. https://coast.noaa.gov/htdata/raster2/elevation/SLR_viewer_DEM_6230/ , (Accessed 11/19/2021).
[5] National Oceanic and Atmospheric Administration. Vertical Datum Transformation https://vdatum.noaa.gov/welcome.html ((Accessed 11/19/2021).

NAVD for the tidal epoch of 1983–2001 for all *tidal waters*. We derived a raster layer representing the height of sea level *over land* relative to NAVD, which we call $\boldsymbol{L_{xy}^{VDatum}}$, by interpolating VDatum over adjacent lands. See Supplemental Methods §1.1. Because 1992 is the midpoint of the tidal epoch, as a shorthand, we treat this layer as elevation above the sea level of 1992.

As sea level rises, land elevations relative to sea level are generally decreasing. To capture the effect of sea level rise, we created a raster surface representing the annual rate of sea level rise by interpolating rates of sea level rise measured by tide gauges provided by the NOAA's National Ocean Service,[6] and then calculated raster layers of land elevations relative to sea level for each **year**:

$$L_{\mathrm{xy}}(\mathrm{year}) = L_{xy}^{NAVD} - L_{xy}^{VDatum} - (year - 1992)\ SLR_{xy}$$

where L_{xy}^{NAVD} is the raster layer of published elevations, L_{xy}^{VDatum} was previously defined, and SLR_{xy} is the raster layer of annual rates of sea level rise. One can then define (for example) the one-meter hazard zone for a given year as everywhere that $L_{\mathrm{xy}}(\mathrm{year})$ is less than one meter. More generally for any given *height*, the hazard zone can be defined with an indicator variable V_{xy}^{height} :

(1) $$V_{xy}^{height}(year) = \begin{Bmatrix} 1 & if\ L_{xy}(year) < height \\ 0 & \text{otherwise} \end{Bmatrix}$$

Like previous studies,[7,8] because few people live in wetlands or open water, we consider only the vulnerable dry land, by excluding wetlands and open water from the hazard zone.

1.2 Specific Analytical Steps:

1.2.1 Land Close to Sea Level

This part of the analysis creates elevation masks (polygons) that define area within a given elevation above mean higher high water (MHHW) for a given year.[9] The steps to create the elevation masks were as follows:

1. Create a 30-meter resolution raster data set of elevations (relative to NAVD) for multi-state regions. See Caveats §7.1
    a. Downloaded USGS 30-meter data for 1 X 1 degree tiles with land below 10 meters. (See Table SM-1.)
    b. Used the ArcGIS **Mosaic** tool to create USGS elevation data sets for separate regions.
    c. Downloaded state-specific NOAA LIDAR data sets. (See Table SM-1.)

---

[6]National Ocean Service, CO-OPS. NOS_Observations/CO_OPS_Products (FeatureServer), Sea Level Trends Stations. https://idpgis.ncep.noaa.gov/arcgis/rest/services/NOS_Observations/CO_OPS_Products/FeatureServer (Accessed 11/19/2021).
[7] Strauss, BH, R Ziemlinski, JL Weiss, and JT Overpeck. 2012. Tidally adjusted estimates of topographic vulnerability to sea level rise and flooding for the contiguous United States Environmental Research Letters 7, 014033.
[8] 2009: Gill, S. K., Wright, R., Titus, J. G., Kafalenos, R., & Wright, K. 2009. Population, land use, and infrastructure. In: Climate Change Science Program. Coastal Sensitivity to Sea-Level Rise: A Focus on the Mid-Atlantic Region. Climate Change Science Program, Washington DC, pp. 105-116.
[9] In the present analysis, we are using 0, 1, 2, and 3 meters for the years 1990, 2000, 2010, and 2020.

   d. Used the ArcGIS **Resample** tool with the NOAA lidar to create smaller data sets using the same cell geometry as the USGS data set.
   e. Used the ArcGIS **Mosaic** tool to mosaic the USGS and NOAA data sets, retaining the NOAA data for any cell where both data sets have data. Call this raster "Elevation".
2. Create a raster data layer representing the elevation of mean higher high water
   a. Downloaded the NOAA Vdatum data for the US coastal zone. (See Table SM-1.)
   b. Used the ArcGIS **Mosaic** tool to create regional Vdatum raster files.
   c. Converted the Vdatum raster files to a point feature class using **Raster to Point**, with a point in the center of each cell.
   d. Created a line feature data set by hand along watershed divides between bodies of water with very different tidal ranges, typically between the ocean and estuaries. Call this data set "barrier".
   e. Used the ArcGIS **Inverse Distance Weighting** tool with the Vdatum point feature class and the barrier file, to define an interpolated surface representing mean higher high water over both open water and dry land. Call this raster "IDW".
   f. Use the ArcGIS **Minus** tool to subtract IDW from Elevation, to create a raster that represents land elevations relative to mean higher high water. Call this raster data set "MHHW".
3. Create a raster data layer representing the rate of sea level rise.
   a. Downloaded the NOAA point data set representing rates of sea level rise (See Table SM-1).
   b. Used the ArcGIS **Inverse Distance Weighting** tool with the sea level rise point feature class, to define an interpolated surface representing the rate of sea level rise. Call this raster SLR.
4. Create elevation masks 0, 1, 2, and 3 meters above MHHW for the years 1990, 2000, 2010, and 2020, using a python program that loops through regions and elevations:
   a. Assume that sea level as defined by VDATUM is sea level for 1992, which is the midpoint of the tidal epoch it used. Define duration = year – 1992.
   b. For each year, use ArcGIS **raster arithmetic** to define

      transient_MHHW(year) = MHHW – duration * SLR

   c. For each of the years, for each of the four target elevations, use the ArcGIS tool **Reclass by ASCII File** to derive a raster file with the value 1 for all cells below the target elevation.
5. Use the ArcGIS **raster to polygon** tool to convert the reclassified raster into a set of polygons, using a python program that loops through regions and elevations: We use the "nonsimplify" option so that the elevation contours defined by these polygons has the same gridded look as the raster data. These are the year-specific elevation masks.

Table SM-1: Data Sources for Vulnerability Zones

a. Rising Sea Level

Purpose	Type of Data	Source	Data Name	Summary
Land Elevation relative to NAVD	Raster	NOAA[1]	Sea Level Rise Viewer, DEM	Elevations based on LIDAR
Land Elevation relative to NAVD	Raster	USGS[2]	National Map 3DEP	Elevations based on best available data
Sea Level Relative to NAVD	Raster	NOAA[3]	VDatum	Modeled elevation of tidal water levels.
Rate of Sea Level Rise	Points	NOAA[4]		Regression coefficients of sea level trends at stations with more than 50 years of data

b. Floodplains

Purpose	Type of Data	Source	Data Name	Summary
Floodplain Vulnerability Zone	Polygons	FEMA[5]	National Flood Hazard Layer	Digital Flood Insurance Rate Maps
Base Flood Elevation in Coastal Floodplains	n/a	FEMA[6]	NFHL Viewer	Create table of coastal base flood elevations

Notes

1. https://coast.noaa.gov/slrdata/ (cited 7/12/2021) . Data download. Used where available.
2. https://www.usgs.gov/core-science-systems/ngp/tnm-delivery (downloaded 2018). Used to cover areas where the NOAA LIDAR was not available, generally areas inland or upstream.
3. Vertical Datum Transformation https://vdatum.noaa.gov/ (downloaded 2017). Used the Mean Higher High Water values.
4. NOAA Tides and Currents, COOPS, Sea Level Trends. Data downloaded https://idpgis.ncep.noaa.gov/arcgis/rest/services/NOS_Observations/CO_OPS_Products/FeatureServer (cited 7/20/2021)
5. National Flood Hazard Layer, FEMA Flood Map Service Center, https://msc.fema.gov/portal/advanceSearch (cited 7/12/2021)
6. Maps reviewed, typical highest base flood elevation noted and recorded for each state. https://www.fema.gov/flood-maps/national-flood-hazard-layer NFHL Viewer.

1.2.2 Land in a Floodplain

1. Create a geodatabase with state-specific layers that define the A, X500, and V zones, land protected by a levee or dike, and floodways.
    a. Download FEMA's National Flood Hazard Layer for each state.
    b. For each state find the layer called S_FLD_HAZ_AR, which in some cases was a shapefile and in other cases a feature class in a geodatabase. Copy the layer into a new geodatabase with one feature class per state.
2. Reduce the 24 flood categories in FEMA's S_FLD_HAZ_AR layers to 10 categories of flood zone.
    a. The FEMA flood layers have fields entitled "Flood Zone" and "ZONE SUBTY". Although some values for these fields are common across states (e.g. Flood Zone = A, or Flood Zone = X with ZONE SUBTY = 500), many fields are limited to a smaller number of states (e.g. Flood Zone = A or X and ZONE SUBTY = Area of Special Consideration.
    b. Based on FEMA's documentation of the mapping categories, defined a field called "Flood Zone" and used ArcGIS **field calculator** to define the values in this field as a function of Flood Zone and ZONE SUBTY, as shown in the first three columns of Table SM-2.
3. Based on the values of Flood Zone, created a geodatabase with separate zone-specific masks (feature classes) defining the A zone, X500 zone, V zone, Levee Zone, and Floodway, as shown in the third and fourth columns of Table SM-2.

TABLE SM-2 MAPPING OF CLASSES IN FEMA'S ORIGINAL DATA TO Floodzone.gdb, AND THEN FURTHER SIMPLIFIED FOR THE MASKS

FEMA_floodplain.gdb		Floodzone.gdb	Mask categories	
FLd_ZONE	ZONE SUBTY	Flood_Zone	Map_Zone	Details about this classification/subclassification
A	<null>	A	A_zone	
AO		AE	A_zone	
AE	" ", <null>	AE	A_zone	
AE	…floodway	FLOODWAY	floodway	Regulatory floodway—restricts building
AE	Encroachment	FLOODWAY	floodway	Regulatory floodway—restricts building
AE	Coastal, Riverine	AE	A_zone	
AE	Special consideration	X500	X500	
X	Special consideration	X500	X500	Along a lake where known but unquantified risk exists. KS and ND
X	0.2 pct	X500	X500	
	1 pct drainage	X500	X500	
X	floodway	FLOODWAY	floodway	Regulatory floodway—restricts building
X	LEVEE	LEVEE	Levee	Land protected by levee for 1% flood
X	1 pct future	future	X500	Probably already in 500-year floodplain; future 1 pct.
X	<1 ft	shallow	X500	The 100-year flood has less than 1 foot
AH		shallow	X500	The 100-year flood has less than 1 foot
A99		LEVEE	Levee	Levee under construction that will protect 1% flood
X	Contained in structure or channel	X	--	Generally these are polygons that are not to scale so they show up on a map, but the actual flooded area is smaller than the polygon.
AE	Contained in structure or channel	X	--	
D		X	--	Old classification for unknown risk
X	Minimal hazard	X	--	New classification for unknown risk
VE		VE	V_zone	Velocity zone, coastal waves
V		VE	V_zone	
"OPEN WATER"			--	
"AREA NOT INCLUDED"			--	

2. Data that Map Vulnerable Population

This analysis uses US Census block data for total population, as well as Hispanic, non-Hispanic Black, non-Hispanic White, as well as housing units, rental units, and owner-occupied units. See Table SM-3 for the sources of the data. In each case, the data source provides a geodatabase or shapefile with the geometry of every census block, and a separate flat file (e.g. csv file) with the population data for each block. To avoid double counting, all calculations of Hispanic population sum all the Hispanic categories in the census data, while calculations of Black population only include variables entitled, "Not of Hispanic origin >> Black" (1990) or "Not Hispanic or Latino >> Black or African American alone" (after 1990).

Table SM-3: Sources of Population Data: Specific Census Data Name for Variable in this Analysis

		----------------------------------Year---------------------------------------			
Variable		1990[1]	2000[1]	2010[1]	2020[2]
Population					
	Total	ET1001	FXS001	H7V001	U7C001
	Black	ET2002	FMS002	H7Z004	U7C006
	Hispanic	Derived[3]	Derived[4]	H7Z010	U7C002
	White	ET2001	FMS001	H7Z003	U7C005
Housing Units					
	Total	ESA001	HV5001	IFC001/IFP001	U7G001
	Rented	derived[5]	FKN002	IFP010	n/a
	Owner-Occupied	derived[6]	FKN001	IFP002	n/a

1. Source: Steven Manson, Jonathan Schroeder, David Van Riper, Tracy Kugler, and Steven Ruggles. IPUMS National Historical Geographic Information System: Version 16.0 [dataset]. Minneapolis, MN: IPUMS. 2021. http://doi.org/10.18128/D050.V16.0
2. Data Tables are from Manson et al., supra note 1. Block geometry shapefiles downloaded from Census website.
3. ET2006 + ET2007 + ET2008 + ET2009 + ET2010
4. FMS008 + FMS009 + FMS010 + FMS011 + FMS012 + FMS013 + FMS014
5. ET0006 + ET0007 + ET0008 + ET0009 + ET0010
6. ET0001 + ET0002 + ET0003 + ET0004 + ET0005

The available census block data on race since 1990 has also included Native Americans and Asian/Pacific Islander, but this study did not analyze those populations. Doing so would have required more work than one might expect because, as mentioned below, the python programs used to join population data and create tables were written to only accept two population variables. Therefore, adding one or both of those variables would have required as much time and effort as evaluating the impact on Black and

Hispanic populations. And doing so would be an entirely severable task, that is, it would be just as easy to do it later if the results are sought as to include it in the initial analysis reported here.

Our judgment was that it made more sense to defer the analysis of Asian/Pacific Islander and Native American populations in these hazard zones. Asian/Pacific Islanders were less than 3 percent of the US population in 1990, and less than 2 percent of the population of the Atlantic and Gulf Coast states most vulnerable to sea level rise. Moreover, at the national level, Asian/Pacific Islanders are not economically disadvantaged compared to Whites or the total population (see Table SM-4). Hence this racial class is not as good of a proxy for social vulnerability as Black or Hispanic.

TABLE SM-4: Per Capita Income by Race or Ethnicity

	1990[1]	2020[2]
White	15,687	38,945
Black	8859	24,454
Native American	8328	21,673
Asian/Pacific Islander[3]	13638	41,808
White Not Hispanic		41,758
Other	7340	20,231
Two Races		23,563
Hispanic	8400	21,846

1. US Census, 1990. Per Capita Income in 1989 by Race, Source code NP114A, and Per Capita Income in 1989: Persons of Hispanic Origin, Source Code NP116A.
2. US Census 2020 American Community Survey: 5-Year Data [2016-2020, Block Groups & Larger Areas]: Per Capita Income in the Past 12 Months. Source codes B19301A to B19301I
3. For 2020, Weighted average of source codes B19301D and B19301E with weights from Total Population, Source Code B02001 columns on “Asian alone” and “Native Hawaiian and Other Pacific Islander alone”.

All data downloaded from IPUMS NHGIS, University of Minnesota, www.nhgis.org.

3. Overlay Vulnerable Zone with Vulnerable Population

3.1 Overview

Our population data have less horizontal resolution than the 30-meter raster elevation data. The boundaries of the blocks themselves are precisely defined, but census data only tell us how many people live in an entire block, which is rarely smaller than 100 X 100 meters and usually much larger. This lack of precision is not a problem where blocks are either entirely within or entirely outside the hazard zone. For example, in New Orleans, 364,000 people live in blocks with at least some land less than one meter above sea level, and 313,000 (85%) of them are in blocks that are entirely below that elevation. But outside of Louisiana, more than 10 million people live in blocks with land below one meter, while only 230,000 (2%) are in blocks entirely below that elevation. (See Table 1, columns 1 and 2)

Previous US studies[10] generally have assumed that the fraction of population of each block[11] [12] (or other census areal unit[13,14]) within a hazard zone equals the fraction of dry land within the hazard zone. Some[15] operationalized this assumption by using (or creating) a raster layer of population density based on the assumption that the population within a given Census block is uniformly distributed throughout the block. With both the elevation and population data using the same grid, those studies calculated the vulnerable population:

$$(2) \quad Population_{heigh} = \sum_{x,y} V_{xy}^{heigh} \; D_{xy} \; A^{grid}$$

---

[10] Outside of the United States, most studies use population data with a 1km grid, and assume uniform density within the cells. E.g. Kulp, S. A., & Strauss, B. H. 2019. New elevation data triple estimates of global vulnerability to sea-level rise and coastal flooding. Nature communications, 10(1), 1-12; Neumann, Barbara, Athanasios T. Vafeidis, Juliane Zimmermann, and Robert J. Nicholls. 2015. "Future coastal population growth and exposure to sea-level rise and coastal flooding-a global assessment." PloS one 10, no. 3 (2015): e0118571;and Mondal, P., & Tatem, A. J. 2012. Uncertainties in measuring populations potentially impacted by sea level rise and coastal flooding. PLoS One, 7(10), e48191.Those data are often created by interpolating larger (e.g., county-scale) population data using remote-sensing indicators of population density, such as land use or night-time light. McMichael, C., Dasgupta, S., Ayeb-Karlsson, S., & Kelman, I. 2020. A review of estimating population exposure to sea-level rise and the relevance for migration. Environmental Research Letters, 15(12), 123005; and Yang, X., Yao, C., Chen, Q., Ye, T., & Jin, C. 2019. Improved estimates of population exposure in low-elevation coastal zones of China. International Journal of Environmental Research and Public Health, 16(20), 4012.

[11] Radeloff, V.C., D. P. Helmers, H. A. Kramer, M. H. Mockrin, P. M. Alexandre, A. Bar-Massada, T. J. Hawbaker, S Martinuzzi, A D. Syphard, and S. I. Stewart. 2018. "Rapid growth of the US wildland-urban interface raises wildfire risk." PNAS 115 (13) 3314-3331.

[12] Strauss et al., supra note 7.

[13] Smiley, K. T. 2020. Social inequalities in flooding inside and outside of floodplains during Hurricane Harvey. Environmental Research Letters, 15(9), 0940b3 (block groups).

[14] Schneider, S. H., & Chen, R. S. 1980. Carbon dioxide warming and coastline flooding: physical factors and climatic impact. Annual review of energy, 5(1), 107-140 (counties).

[15] Hauer, M. E., Hardy, D., Kulp, S. A., Mueller, V., Wrathall, D. J., & Clark, P. U. 2021. Assessing population exposure to coastal flooding due to sea level rise. *Nature communications*, *12*(1), 1-9; Strauss et al., supra note 7; Radeloff et al., supra note 11.

where A^{grid} is the area of a grid cell, D_{xy} is the population density of the cell with coordinates (x,y), and V_{xy}^{height} is again the indicator variable that equals 1 within the zone of dry land with an elevation less than *height*. (For purposes of this section, "population" can refer to total residents, housing units, Black, Hispanic, or any other population variable.)

Instead of converting the polygon-based census block data to a grid to match the elevation data, this study (like some older studies[16]) defines the hazard zones as a set of polygons representing the land with elevation less than the threshold *height* under consideration. Then for each block *i*, the vulnerable area $\boldsymbol{A_i^{height}}$ can be defined as the area of block *i* less than ***height*** above sea level. Geospatial programs such as ArcGIS can readily "clip" the intersection between the block and elevation polygons, and calculate the area of the intersection $\boldsymbol{A_i^{height}}$. The vulnerable population can then be calculated:

$$(3)\ \ Population_{height} = \sum_i (A_i^{height}/A_i)\, P_i$$

where P_i is the population of the ith block, $\boldsymbol{A_i}$ is the area of dry land in that block, and $\boldsymbol{A_i^{height}}$ is the area of dry land below ***height***. Although the grid and polygon approaches handle the data differently, they are mathematically equivalent aside from rounding error: if ***X***% of a block is below *height*, then ***X****%* of the population of that block is assumed to live below ***height***.

Assuming a uniform population distribution within a census block is reasonable in some areas, but significantly overstates the vulnerable population in others. In urban areas where census blocks are city blocks, development usually is fairly uniform within a given block. But wherever residential lots are large, people usually build on the high part of their lots. And in rural areas, the high ground may be more developable due to septic tank or floodplain regulations. (See Figure 1 in the main paper.)

A priori, one does not know whether the upward bias of a population estimate from uniform density is significant. This study considers an alternative approach. Using building footprint data,[17] an alternative assumption to uniform density is:

$$(4)\ \ Population_{height} = \sum_i (B_i^{height}/B_i)\ P_i$$

where $\boldsymbol{P_i}$ is the population of the ith block, B_i is the number of buildings in that block, and B_i^{height} is the number of buildings on land below ***height*** in that block. That is, if ***X***% of the buildings in a block are below ***height***, then ***X****%* of the residents of that block live below ***height***. It seems reasonable to assume that buildings are a better indicator than land area of the fraction of a block's population living in the hazard zone. (Others have considered road centerlines[18] or

---

[16] Schneider and Chen, supra note 14; and Gill et al., supra note 8.

[17] Microsoft US Building Footprints. https://github.com/Microsoft/USBuildingFootprints (accessed November 19, 2021).

[18] Kulp, S., Strauss, B.H. Rapid escalation of coastal flood exposure in US municipalities from sea level rise. 2017. *Climatic Change* **142,** 477–489. p. 479. (2017). https://doi.org/10.1007/s10584-017-1963-7

occupied parcels.[1913]) In addition, Equation 4 avoids some spurious trends: Blocks from an early census are often subdivided in a later census into a developed block and an undeveloped block. Such remapping changes the population estimate from Equation 3 while having no effect on Equation 4, since B_i^{height}/B_i is zero in the new undeveloped block and the same as before in the developed block.[20]

3.2 Specific Analytical Steps

Our calculations assume that people do not live in wetlands nor in floodways, therefore the overlays only consider "dry land", that is land that is neither wetland nor water nor (in the case of floodplains) floodway. With that assumption, the following steps described in this section are undertaken so that we will have the ability to use four approaches for calculating vulnerable population in each census block:

a. Count the entire population if the dry land in the block is entirely (99.5%) in the vulnerable zone, otherwise do not count this block (an extreme lower bound estimate of vulnerable population).[21]
b. Uniform Density: Calculate the percentage of the dry land that is in the vulnerable zone; assume that this is also the percentage of the block's population that is in the vulnerable zone.
c. Building-based density: Obtain a data base of building footprints. Count and then calculate the percentage of buildings in the block that are in the vulnerable zone, and assume that this is also the percentage of the block's population that is in the vulnerable zone.
d. Footprint-based density:[22] Using the same database of building footprints, calculate the area of all buildings and then the percentage of building footprints in the block that are in the vulnerable zone, and assume that this is also the percentage of the block's population that is in the vulnerable zone.

3.2.1 Create tables with the area of dry land, area of land below various elevations, and population for each census block.

1. Create a simplified coastal wetlands data set
    a. Download National Wetlands Inventory data.
    b. Create a 10-meter NAVD mask using the same data as used to define the vulnerability zone.
    c. **Select** polygons in wetlands data set that overlap the 10-meter mask.

[19] Smiley, supra note 13
[20] Cf. Radeloff et al., supra note 11, p. 3318 (addressing the same challenge a different way).
[21]Results from this assumption are reported in Table SM-1. This result is useful for understanding the practical importance of assumptions regarding population distribution within a census block, this population is in the vulnerable zone for any such assumption. In addition, when calculating a ratio estimate of the total vulnerable population, the ratio estimator only applies to the blocks that are not entirely in the vulnerable zone.
[22] Although these results are calculated, the paper does not report them because they generally track the calculations based on the number of buildings.

d. For purposes of visual maps, define a field of wetland classifications that divides wetlands into tidal wetlands, nontidal wetlands, tidal open water, nontidal open water.
e. **Dissolve** the wetlands based on the four coastal wetland categories.

2. Create a geodatabase of state-specific feature classes of "low dry blocks", that is, of the dry portions of all blocks with any land less than 10 meters above NAVD.
   a. Using the census data in Table SM-3, **Select** polygons with any land that overlaps the 10-meter NAVD mask. Call this feature class "low blocks"
   b. Create a feature class of "low dry blocks" with the ArcGIS tool **erase**, with low blocks as the target feature and the coastal wetlands as the erase feature.
   c. Create a field with the Area_of_block using **add field** and **field calculator**.
3. Create geodatabases of state-specific feature classes consisting of the portions of census blocks below 0, 1, 2, and 3 meters (MHHW) for specific years of interest, and calculate the area of the block below those four target elevations.
   a. **Clip** the "low dry block" feature classes using the 0-, 1-, 2-, and 3-meter masks calculated for specific years.
   b. For each of these clipped feature classes, create a field with the areas of the polygons, called "Area_below_3m" …. "Area_below_0m" using f**ield calculator**
   c. **Join** the fields with the polygon areas from the 0-, 1-, and 2- meter clips to the 3-meter clip feature class. Call this feature class "blocks_below_3m".
   d. Use **repair geometry** on the blocks before and after the clips.
4. **Join** population tables from the Census data to the blocks_below_3m feature class. Call this "below_3m_joined". The python program written to carry out this task allows for only two population variables because initially we only anticipated needing population (the key reported result) and housing units (used for the preliminary test of the method). That program was later adapted so that it can take any two variables.

3.2.2 Create tables with the area of dry land, number of buildings, area of land below various elevations, number of buildings below various elevations, and population for each census block.

1. Create a geodatabase with state-specific feature classes of all the buildings within any census blocks with any land below 3 meters, and a field with the areas of each building. Unless otherwise stated, we undertake the following tasks for each state:
   a. Downloaded the Microsoft Building footprint data.[23]
   b. Used JSON to Features tool to get the feature class in a geodatabase.
   c. **Select** "very_low_blocks" for each census year. The previously created "low blocks" are the target and the 3-meter mask for the year 2020 is the mask used for the selection. This defines very_low_blocks for each year of census data.
   d. For each census year, **Merge** the very low blocks for all states in a given region along with the three-meter mask for the year 2020, to create a three-meter-very-low-block mask for each census year for each region.

[23] data from https://github.com/Microsoft/USBuildingFootprints. Images from various vintages of data averaging around 2012.

e. **Union** the very_low_blocks for each of the four years to define the building study area, which we call union-very-low-block-mask.
f. Create a geodatabase of state-specific feature classes ("buildings in lowblocks") with all the buildings that might be used in the analysis: **Select** all the buildings that are at least partly within the union-very-low-block mask. Call this selection "buildings in lowblocks."
g. Add a field to the buildings-in-lowblocks feature class with the footprint of each building using **field calculator** to calculate the area of each building.

2. Create geodatabases of state-specific feature classes consisting of the portions of buildings below 0, 1, 2, and 3 meters (MHHW) for specific years of interest; and calculate the area of the building below those four target elevations.
   a. **Clip** the "buildings in lowblocks" feature classes using the 0-, 1-, 2-, and 3-meter masks calculated for specific years 1990, 2000, 2010, 2020
   b. For each of these 16 clipped feature classes, create a field with the areas of the building polygons, called "footprint_below_3m" (in the case of the 3-meter clip) or equivalent name based on 0m, 1m, or 2m, using **field calculator.** For each building polygon in the feature class, this field provides the area of that building below a certain elevation relative to MHHW for the specified year.
   c. For each of the 16 clipped feature classes, create a field for number_of_buildings_below_[X]m where [X] is 0, 1, 2, or 3 based on the clip elevation, by dividing the footprint below the focus elevation by the total footprint of the building. This will usually be either 0 or 1, but it will be a decimal fraction in the case of buildings through which a given elevation contour runs.
   d. **Join** the fields with the polygon areas from the 0-, 1-, 2-, and 3-meter clips to the "buildings in lowblocks_year" feature classes. Call this feature class "buildings_in_3m_blocks_year" which is a bit of a misnomer, because the feature class has all the low blocks and associated "number_of_buildings" and "building_footprint" fields. But in a later step, all of the buildings will be associated with a block that has land below 3m for the year.

3. Create feature classes of buildings that state the census block the building is in: **Spatial join** of buildings to census blocks. The join involves the following iteration within an iteration.
   a. For each year of the analysis, the target of the join is the buildings_in_3m_block_year feature class for that year, that is, the feature class with a table stating the area of each building (and number of buildings) below the various focus elevations (0, 1, 2, 3 meters) for that year of the analysis.
   b. For each target feature class ("buildings_in_3m_block_year"), conduct a separate **spatial join** for each of the four years, corresponding to the census blocks for each of the years. The join feature is the feature class state_very_low_blocks_year. The goal of the spatial join is to add a field of census block identifiers to the building feature class, so that for each building in a feature class for a given block year, the field indicates the census block in which the building is found.
   c. Thus, there are 16 **spatial joins** corresponding to 16 combinations for the census year and year of sea level rise. For a given combination of (block_year, mask_year), the output of the join consists of the buildings in the study area. For each building, the table includes a field of census block identifiers which shows which census block the building

was in for the census of **block_year** and the fraction of the building below each focus elevation for the sea level of **mask_year**

   d. The join has a search radius of 100 meters and matches to the closest census block. We follow this approach because some census blocks have been drawn so that buildings near wetlands were shown outside of the census block in 1990, while the population of the building was associated with the census block. We must balance that problem with the risk of including building footprints for navigational structures.
   e. After doing the join, a filter removes all buildings that are not in the three-meter very low block mask for a given census year. With approach, every building will be in or close to a block with land below 3m. Call the output: STATE_buildings_in_3m_blocks_joined_BLOCK_YEAR_MASK_YEAR

4. Create feature classes of census blocks with the total area of the block, total number of buildings, and total building footprints, and the values of those quantities below focus elevations, for all combinations of year-specific census blocks and sea level rise.
   a. For each census and each year of sea level rise, run some statistics on the buildings_in_3m_blocks feature using the **statistics** tool, which is similar to pandas groupby. Create a table with a different row for each census block, with a count of the total number of buildings and the total area of building footprints, as well as the number of buildings and area of footprints below the given elevations.
   b. **Join** each of the 16 tables just calculated to the very low block feature class for the census year. Thus, for each census, there will be four different tables representing the sea level rise for the four different years. Call the result "building_count_by_block" for each of the 16 combinations.
   c. Create the feature class with all the results: **join** the "building_count_by_block" feature class to the "below_3m_joined_table, which has areas and population by block. The python program written to carry out this task allows for only two population variables because initially we only anticipated needing population (the key reported result) and housing units (used for the preliminary test of the method). That program was later adapted so that it can take any two variables.

5. Export the table from the aforementioned feature class to csv tables, using the t**able to table** tool. The python program written to carry out this task allows for only two population variables because initially we only anticipated needing population (the key reported result) and housing units (used for the preliminary test of the method). That program was later adapted so that it can take any two variables.

3.2.3 Analysis of Floodplains.

The floodplain analysis is essentially the same, with the following differences:

1. The different vulnerability zones (A zone, X500 zone, V zone, levee zone) are mutually exclusive rather than cumulative.
2. The flood zones do not change over time, so there are only 4 rather than 16 sets of clips and results.

3. For the same reason, we have only one set of results rather than three sets of trends. That is: for sea level rise we look at (a) the actual number of people below 1 meter for each census, (b) the effect of population change alone, i.e., number of people below 1 meter above the fixed sea level of 2020 for each census, and (c) the effect of sea level rise alone, i.e., the number of people below 1 meter for each of the years, only using the 2020 census.
4. For coastal states, we subdivide floodplains into those dominated by storm surge—often called coastal floodplains—and those dominated by other processes such as precipitation, snowmelt, and lake storms.
    a. Examine the digital flood insurance rate maps, which show the elevation of the 100-year storm and the velocity zone. Create a table of state-specific highest base flood elevations. See Table SM-5.
    b. Create an elevation raster mask representing all land with an elevation lower than the base flood elevation of the state, analogous to step 4(c) in §1.1, except that the input elevation for reclass by asci is the base flood elevation rather than (for example) one meter.
    c. Convert the raster to polygons, analogous to step 5 in §1.1.
    d. **Select** all floodplain blocks with dry land that intersects the base flood elevation mask, and call these the storm-surge floodplains. Assign all other floodplain polygons as riverine floodplains.

TABLE SM-5
Highest Typical Base Flood Elevation, By State
(feet above NAVD)

State	BFE		State	BFE		State	BFE		State	BFE
ME	19		NJ	15		NC	15		LA	22
NH	19		PA	12		SC	15		TA	19
MA*	16		DE	15		GA	12		CA	25
RI	16		MD	13		FL*	16		OR*	30
CT	17		DC*	17		AL	15		WA	22
NY	18		VA	12		MS	23		HI	8

* Based on A-zone for Boston/Falmouth (MA), Georgetown (DC), Taylor County (FL) and Portland (OR). For all other states, based on coastal V-zone elevations.

3.2.4 Python Program and Error Handling

Most of the tasks described below must be repeated many times, using standard ArcGIS tools. Therefore, we have small python programs that combine a few related tasks and loop through all the combinations of state, census year, sea level rise year, and vulnerability zone. Because of quirks in

ArcGIS, a program would usually not be able to loop through all the states without an error occurring, virtually all processing takes place within try-except blocks, so that—at the very least—errors one state would not prevent the (typically overnight) completion of a processing step.

The error handling also takes care of some of the most typical errors. For example, census blocks are in an Albers equal area format, while elevation data are generally in a geographic coordinate system (GCS). The ArcGIS **clip** often fails when it attempts to "clip on the fly" the census blocks with a mask based on a geographic coordinate system, which is usually remedied using the ArcGIS project **tool** to convert the census blocks to the GCS, then clip the blocks, and then re-**project** the blocks back to the Albers equal-area format which is necessary to calculate the area of the blocks. Moreover, sometimes a standalone tool in a python program can trigger an error, while the same tool as part of an ArcGIS model called by the same python program does not cause an error. And sometimes when either of those alternatives cause an error, the reprojection as part of a model does not cause an error. Similarly, errors caused by a failure of **join** after a clip sometimes work when the clip and join are called in a model.

Our exceptions handlings thus have several alternatives that may work, each of which are attempted before the program gives up on a state. These alternative approaches can alter the calculated area for a block. Although the differences in area estimates are small, the changes in the area of a block from a decade's worth of sea level rise are also small—and sometimes the difference between the area below (e.g.) 2m and 1m can be small. To prevent spurious trends from being caused by the error handling, whenever an error was triggered for a given calculation, the error handling starts over for the state, so that all calculations for a given state use the same procedure. When the error handling also failed for a given state, usually running the program on that state the next day and/or on a different computer has worked.

4. Calculate and Display Summary Statistics

A python program inputs csv files for each state for each combination of census year and sea level rise year, to tabulate results for alternate assumptions of how population is distributed within a census block, for each of four hazard zones (e.g. land <0m, <1m, <2m, <3m).

a. Entire: One set of results are based on adding only those blocks that are entirely (99.5%) in the defined hazard zone. A parameter can be set so that instead the results are based on adding all the blocks with any land in the hazard zone. (Both of these results are reported in Table SM-1).
b. Uniform Density: Another set of results assumes that the population below a given elevation equals the population of the block times the area of dry land in the block below the elevation divided by the total area of dry land in the block.
c. Building-based density: assumes that the population below a given elevation equals the population of the block times the number of buildings in the block below the elevation divided by the total number of buildings in the block.
d. Footprint-based density: assumes that the population below a given elevation equals the population of the block times the footprint of buildings in the block below the elevation divided by the total footprint of buildings in the block. (The paper does not report these results.)

The program also provides at the state level various diagnostic results useful for quality control of the analysis, some of which are limited to a focus hazard zone that can be selected (e.g., land<1m). The diagnostics include area of land within the hazard zones by state, area of land in populated blocks, total area of blocks with land in the focus hazard zone, and total area of populated blocks with land in the focus hazard zone. The program also provides the aforementioned results at the county level for a specified focus hazard zone.

The program has various parameters for choosing between the many permutations available from the csv tables. Each run of the program loops through the set of years a single time, so for evaluating land close to sea level, one must choose whether to keep sea level or population fixed to a particular year or estimate the actual vulnerable population by using 1990 sea level for the 1990 census block, 2000 sea level for the 2000 census, etc. As previously mentioned, if sea level or census is kept fixed to 1990, the results are only available for the 1m hazard zone; but if the fixed year is 2020 results will be available for all the vulnerable scenarios. Another specified parameter must be set to select between total population, Black, Hispanic, housing units, rental units, or owner-occupied units.

5. Measures of Disproportionality

5.1 Nationwide and County-Weighted Disproportionality

In the year 2000, Black people accounted for 24 percent of the people living below 1 meter, but only about 12 percent of the national population. (See Titus 2023b, Table S6-A). Approximately 1.6 percent of Black US residents lived below one meter, while only 0.8 percent of all US residents lived below 1 meter. Thus, Black people appear to be twice as likely to live below 1 meter as the population at large. We might define the ratio of disproportionality D as follows:

$$(1) \quad D = \frac{F^*_{Black}/X^*_{Black}}{F^*/X^*},$$

where F^*_{Black} and X^*_{Black} are the Black population in the relevant floodplain (e.g. <1m) and the nationwide, respectively, and F^* and X^* are the total population of all races in the floodplain and nationwide, respectively.

This might also be written as

$$(2) \quad D = \frac{F^*_{Black}/F^*}{X^*_{Black}/X^*},$$

In the first case, the ratio is 24 percent to 12 percent from the first sentence of this section, while the second ratio is 1.6 to 0.8 from the second sentence.

For some purposes, this nationwide ratio of disproportionality is a potentially significant thing to know. But the nationwide ratio provides little insight about the causes of the disparity. Did exclusionary zoning or local land-use policies increase the number of Black people living in hazardous areas? Clearly, the extreme disproportionality is largely driven by New Orleans, which accounted for 294,000 of the 530,000 Black people—and 400,000 of the 2.2 million people of all races—living below 1m. Katrina reduced the Black population more than 100,000 so that by 2010, Black people were only 20.5 percent of the people living below 1m, and the ratio of disproportionality declined to 1.7. Excluding New Orleans, the ratio of disproportionality has been about 1.1 since the year 2000. Thus, New Orleans alone raised the nationwide ratio of disproportionality from 1.1 to 2.0. And yet, the ratio of disproportionality for New Orleans itself was only 1.06, because Black people accounted for 71 percent of the population living below 1m and 67 percent of the total population, or put another way, 91 percent of the black population lived below 1m compared with 86 percent of the entire population of this city. New Orleans raised the nationwide ratio of disproportionality because it is disproportionately vulnerable to sea level rise (86 percent below 1m compared with 0.8 percent nationwide) and disproportionately Black (66 percent compared with 12 percent nationwide), not because Black residents within the city are disproportionately vulnerable. As a result, New Orleans accounted for 56 percent of the Black population below 1m, while only 19 percent of the total population below 1m.

Strictly speaking, Black people could account for a disproportionate share of the nationwide vulnerable population without being disproportionately vulnerable anywhere. Table SM-6 provides a numerical illustration: County A's population, like New Orleans, is both majority Black and the majority is below 1m. County B is more typical, with 20 percent of the population being Black, and less than 1 percent of the population living below 1m. In each county, the fraction of the Black population living below 1m is only 90 percent of the fraction of the total population below 1m, so as one would expect, at the county scale the ratio of disproportionality is 0.9. Yet the aggregate nationwide ratio of

disproportionality is 1.48, because 65 percent of the Black residents live below 1m while only 45.45 percent of all US residents live below 1m. Thus, in this example, Black people are disproportionately vulnerable nationwide even though Black people are not disproportionately vulnerable in any county.

TABLE SM-6 Numerical Illustration								
	Population (thousands)						Ratio of Dispro-portionality	Counter-factual Black Population
	Total Population		Population <1m		Percent <1m			
	Black	Total	Black	Total	Black	Total		
Town A	800	1,000	648	900	81	90	0.9	720
Town B	200	1,000	1.62	9	0.81	0.90	0.9	1.8
Nation-wide	1,000	2,000	650	909	65	45.45	1.43	721.8
County-by-County Ratio		=	650/721.8		=		0.9	

Our analysis calculates the county-level ratio of disproportionality *C* as follows

$$(3)\quad C = \frac{\sum_i F_{Black,i}}{\sum_i F_i X_{Black,i}/X_i},$$

where X_i is the population in county *i*, $X_{Black,i}$ is the Black population in county *i*, F_i is the population below 1m of county i, and $F_{Black,i}$ is the Black population below 1m in the county *i*. That is, C is computed as the ratio of the total Black population below 1m, to a sum that represents what the Black population below 1m would be if in every county, the Black share of the population below 1m equaled the Black share of the overall county population. The denominator of this ratio is calculated in the final column of Table SM-4, and in this case C=650/721.8 = 0.9, which is intuitively what we might hope given that the county-specific ratios of disproportionality were both 0.9.

Note that if $X_{Black,i}/X_i$ is constant across all counties, then the denominator can be re-expressed:

$$(4)\quad \sum_i F_i\, X_{Black,i}/X_i = \sum_i F_i\ \sum_i X_{Black,i}\,/\,\sum_i X_i, \text{ and}$$

$$(5)\quad C = \frac{\sum_i F_{Black,i}}{\sum_i F_i\ \sum_i X_{Black,i}/\ \sum_i X_i} = \frac{\sum_i F_{Black,i}/\sum_i X_{Black,i}}{\sum_i F_i\ /\ \sum_i X_i} = \frac{F^*_{Black}/X^*_{Black}}{F^*/X^*} = D,$$

That is, the county-level ratio of disproportionality will be the same as the nationwide ratio of disproportionality if all counties have the same racial composition. The same would apply if, instead, F_i/X_i is the same in all counties.

The county measure might be called an inverse weighted sum of the county-specific ratios of disproportionality. If we define $D_i = (F_{Black,i}/F_i)/(X_{Black,i}/X_i)$, then $F_{Black,i}/D_i = F_i X_{Black,i}/X_i$, so:

$$(6)\quad C = \frac{\sum_i F_{Black,i}}{\sum_i F_i/D_i},\ \text{and}\ \frac{1}{C} = \frac{\sum_i F_i/D_i}{\sum_i F_{Black,i}} = \frac{1}{F^*_{Black}}\sum_i \frac{F_i}{D_i},$$

which means that 1/C is a weighted sum of the $1/D_i$.

5.2 An Alternate Measure that May Be More Appropriate for Communicating to the General Public.

A different way of explaining the New Orleans example in the first paragraph of §5.1 would be:

> In the year 2000, Black people accounted for 24 percent of the people living below 1 meter, but only about 12 percent of the national population. Approximately 1.6 percent of Black US residents lived below one meter, while only 0.69 percent of people from other races lived below 1 meter. Thus, Black people appear to be 2.3 times as likely to live below 1 meter as people who are not Black.

This description would lead us to define a ratio of disproportionality D_{alt} as follows:

$$(7)\quad D_{alt} = \frac{F^*_{Black}/X^*_{Black}}{(F^* - F^*_{Black})/(X^* - X^*_{Black})}\ .$$

The alternative approach of Equation 7 may be more intuitively appealing because for some purposes it is more informative to compare one group to another group, rather than to the general population—especially when only one or two results are being communicated. Nevertheless, this paper does not use that approach, for three reasons.

- Equation 1 is simpler
- Equation 1 has the same denominator F^*/X^* for both Black and Hispanic disproportionality, while Equation 7 has different denominators.
- The county-weighted measure in Equation 3 would not be an inverse-weighted sum of the ratios of disproportionality.

6 Overlays with Two Other Data Sets.

6.1 Redlining Layer Based on Maps from the Home Owners' Loan Corporation

6.1.1 Data Sources

The Home Owners' Loan Corporation divided portions of about 239 cities into four zones representing different levels of risk to mortgage lenders. The University of Richmond has created a data layer by digitizing old hard copy maps from 101 of these communities[24] (hereinafter HOLC data). Figure SM-1 shows an example overlay of those data with Census blocks data for Baltimore, Maryland and its suburbs, with the HOLC polygons depicted in the colors generally associated with the so-called "redlining".

[24] Robert K. Nelson, LaDale Winling, Richard Marciano, Nathan Connolly, et al., "Mapping Inequality," American Panorama, ed. Robert K. Nelson and Edward L. Ayers, https://dsl.richmond.edu/panorama/redlining/ (updated 12/17.2020). Accessed February 1, 2022
See https://dsl.richmond.edu/panorama/redlining/#loc=5/47.04/-83.145&text=downloads for the list of cities.

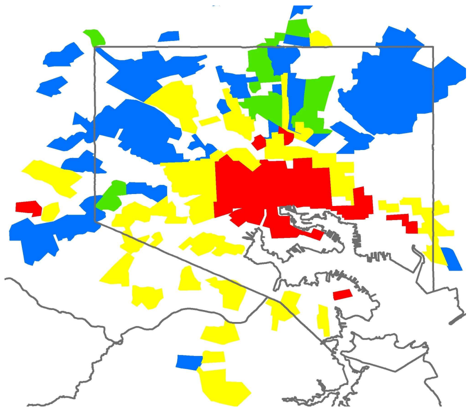

Figure SM-1. Mapped HOLC zones for Baltimore, Maryland.

- Green: Risk category A, which was viewed as "Best", represented the least risk, and was often homes in new neighborhoods.
- Blue: Risk category B, which was also viewed as "still desirable" but with some risk.
- Yellow: Risk category C, which was viewed as "Declining". The buildings tended to be older, some lacked utilities, and residents were often working class and/or first or second European immigrants.
- Red: Risk category D, which was viewed as "Hazardous" and viewed as the riskiest for mortgage lenders, often because they were in floodplains, near industrial areas, and/or populated by Black people or other minorities.

As the map shows, parts of Baltimore and most of its suburbs were not included in the HOLC maps.

6.1.2 Analysis

For each of these categories, we created feature classes comparable to those developed above in section 3.2 (4)(c) and tables comparable to section 3.2(4)(d) to estimate the population close to sea level for each of the zones, as follows:

1. Define the Census blocks close to sea level that can be associated with each of the HOLC categories.
    a. Start with
        i. the feature class developed above in 3.2 (4)(c). Recall that this feature class has the geometry of census blocks clipped by the 3-meter contour. The attribute tables display the area of dry land, number of buildings, and population variables in the entire block, as well as the number of buildings and area of dry land below 0, 1, 2, and 3m. meters.
        ii. A zone-specific HOLC mask (e.g. red zone) from the University of Richmond Data.
        iii. The wetlands data set from 3.1(1) above.
    b. For each HOLC risk zone (e.g. red zone), use ArcGIS Selects tool to select all the blocks that have any part land over the red zone. Call this the overinclusive selection since it includes blocks that are mostly in a different HOLC zone.
    c. Select from the overinclusive section all of those blocks with centers in the (e.g.) red zone.
    d. Select from the overinclusive selection all of those blocks with centers over the wetland/water layer. Call this the wetland-centered red blocks.
    e. Select from the wetland-centered red blocks, using the "remove from selection" option blocks that intersect any color other than red.
    f. Merge the selections from steps c and e.
    g. Export the table from the aforementioned feature class to csv tables, using the t**able-to-table** tool.

2. Create a county-specific table of the population the Census blocks associated with each of the HOLC categories, regardless of elevation or vulnerability to flooding.
    a. Start with
        i. The census block data described in Table SM-3.
        ii. A zone-specific HOLC mask (e.g. red zone) from the University of Richmond Data.
        iii. The wetlands data set from 3.1(1) above.
    b. Create feature classes with the census blocks within (e.g.) the red zone following the approach of steps 1(b) to 1(f) above.
    c. Join the census block data to the feature classes.
    d. Use the Statistics tool to create tables with the population grouped by county.
3. Calculate various results using the same programs as those in Section 4 ("Calculate and Display Summary Statistics"), above.
4. For blocks within floodplains, follow the same approach as enumerated here for blocks close to sea level in step 1.

See Tables S11 to S14 Titus (2023b and 2023c) for results.

6.2 Portion of Populations inhabiting land almost certain, likely, and unlikely to be protected from rising sea level.

6.2.1 Data Sources

A study funded by the US Environmental Protection Agency to support the US Climate Change Science Program developed maps depicting the likelihood of shore protection for lands below the 20-foot contour along the US Atlantic Coast from Massachusetts to Florida,[25] based on interviews with local planners in the 14 states. The maps divide coastal lands below the 20-foot contour into four categories: protection almost certain, protection likely, protection unlikely, and no shore protection. EPA makes the data underlying those maps available either as shapefiles based on the original data sources of the analysis, and as a 30-meter raster data set that generally simplifies the finer-scale shapefiles. This study uses the raster data.

6.2.2 Analysis

The approach was similar to the approach described above for the HOLC data set in §6.1.2, but someone simpler.

We started out with the same feature class developed above in 3.2 (4)(c). Recall that this feature class has the geometry of census blocks clipped by the 3-meter contour. The attribute tables display the area of dry land, number of buildings, and population variables in the entire block, as well as the number of buildings and area of dry land below 0, 1, 2, and 3m.

For each of the four shore-protection categories (scenarios) use ArcGIS Selects tool to select all the clipped blocks with their centers in that particular category, and save those blocks in a scenario-

[25] Titus, J. G., et al. 2009. State and local governments plan for development of most land vulnerable to rising sea level along the US Atlantic coast. Environmental Research Letters, 4(4), 044008.

specific geodatabase. Clipped blocks whose centers are over wetlands or waters were not selected by this approach but are saved in a separate geodatabase of "unclassified" blocks.

We then exported the tables from the five aforementioned feature class geodatabase to csv tables, using the t**able-to-table** tool, and tabulated the resulting statistics in the same manner as described in §3. Because areas where all shore protection is prohibited are generally either conservation lands or narrow strips of land along cliffs, the population of blocks in the no protection category was generally negligible except where a clipped block where shore protection is allowed coincidentally had its center in the no-protection zone. Therefore, we do not report results for that area separately and instead include it along with the blocks with centers over wetlands and water.

It would have been possible to subdivide the unclassified blocks based on the protection category that overlapped parts of those blocks, as we did for the HOLC analysis. This additional refinement was needed more for the HOLC analysis because a much larger portion of the blocks in the HOLC analysis would have remained unclassified if that analysis was based entirely on the centroids of the clipped blocks. While the shore-protection analysis included all coastal lowlands in the states examined, the HOLC maps only included selected communities. Thus, blocks along the borders of the HOLC maps may have centers inland of the HOLC maps, or along the shore, in addition to being over wetlands or water.

7 Miscellaneous Caveats about these Methods

7.1 Using 30-meter grid for elevation data

As described in §1.1, this analysis resampled elevation data to a 30-meter grid, rather than using the 10-meter grid. Doing so saved processing time and data storage in the creation of time-dependent elevation masks, and in the clipping of census blocks. Here is a list of reasons for expecting that the resulting loss of accuracy is unlikely to be important at the scale of this analysis.

1. Strauss et al. 2012 looked at this issue and found that using a 10-meter grid increased the estimated area <1m by 0.2%, and the area <3m by less than 0.05%. It increased the population <1m by 0.9% and the population <3m by 0.3%.
2. In relatively flat areas,
    a. if a 30-meter cell is entirely below 1m, then all nine of the contained 10-meter cells are also below 1m.
    b. if the 1-meter contour runs through a 30-meter cell, then some of the 10-meter cells will be <1m and some will be >1m. Because the grids are established independently of the landscape, one would generally expect that more than half the cell being <1m and more than half the cell being >1m are equally likely; and one would also expect that the average elevation being <1m and >1m are equally likely. Thus, the total area of 30-m blocks that average <1m is likely to be close to the total area of 10-m blocks that average <1m.
3. In areas along bluffs, the 30-m cells would miss the 10-m cells that are entirely below the bluff, and thereby understate land <1m. However, this would make relatively little difference for population because in such cases, the buildings are generally above the bluff. The same logic would apply along streams.

7.2 Using building footprint data for a single year

The Microsoft footprint data provides a single snapshot of where buildings were, with various vintages for different areas but generally averaged around the year 2012. Therefore, our building-based approach is likely to be less accurate for 1990, 2000, and 2020 than for 2010. If there has been a trend away from (or toward) building in the vulnerable portions of census blocks, our approach misses that and hence we would overstate (understate) the population for 1990 and thus overstate (understate) the increase over the period. The uniform density approach also would have the same tendencies because it assumes that the fraction of population in a given vulnerable zone is equal to the fraction of the area of the block in the zone. Therefore, having building data for only a single snapshot introduces makes the building density approach less accurate than it would be if we had footprint data for each decade; but that error is the same for uniform density.

How much less accurate depends on how the fraction of buildings in the vulnerable zone changed over time. For example, if the number of buildings doubled but the percent of buildings in the vulnerable zone remained the same, then no error is introduced. Conversely, if all the buildings in the vulnerable zone were built around the year 2000, then the vulnerable population in 1990 should be zero; so our approach overstates the vulnerable population for 1990 by assuming that the fraction of population in the vulnerable zone remained the same.[26]

Let us define "trend bias" as the ratio of the percent of buildings in the vulnerable zone in 1990 to the percent of buildings in the vulnerable zone in the year 2012. If the post-1990 buildings tended to be in the vulnerable zone, then this ratio would be <1, and our approach overstates the population in the vulnerable zone for 1990, and thus understates the trend in population.

Referring to Figure 1 in the letter,

a. The rural blocks depicted could see additional homes in any portion of a block, so there is no reason *a priori* to think that the trend bias is positive or negative, though some error would be introduced. We can hope that these errors offset, but if new development is prohibited in a vulnerable zone, then the estimate of vulnerable population for 1990 is higher than would be the case if we had building data for all years, and there would be a positive trend bias from this lack of data. This would be equally true for uniform density: both approaches ignore any within-block changes in the fraction of population in the vulnerable zone.

b. In a relatively dense block, where the low land is along the shoreline additional development should not alter the fraction of buildings in the hazard zone. As before, any trend bias would be the same for both uniform density and building-based density since neither approach measures the within-block changes.

[26] We are assuming here that the vulnerable zone is not changing over time, as is the case when we are looking at either a fixed elevation contour or the floodplains. If the changing vulnerability zone increased the number of buildings in our footprint data by X percent, then the same logic applies though the analysis assumes that the fraction of population in the vulnerable zone increased by X%.

c. The same story applies if streets account for the low land.

d. In a block developed in the 1950s and 1960s, the number of buildings generally does not change though small houses may be replaced with larger houses and occupied by more year-round residents.

The trend bias is likely to be greater for inland floodplains than for coastal floodplains and land close to sea level. Local regulations often prohibit construction in riverine floodplains or require structural fortifications that are costly compared to the cost of finding alternative construction sites out of the floodplain. In coastal floodplains, however, the building elevation requirements usually do not prevent development, because of the demand to live near coastal waters. Nevertheless, in those areas where low homes are being abandoned as sea level rises, buildings that have been removed are not accounted for and hence emigration from land close to sea level would be understated.

7.3 Accounting for Subsidence

Relative sea level rise as measured by a tide gauge accounts for both subsidence and sea level rise. In some areas, subsidence may diverge significantly from the subsidence at the tide gauge. This study did not account for such variations in subsidence. Similarly, this study does not account for subsidence that occurred during the interim where map dates differ.

7.4 Ratio Estimator for Total Population Below 1m

The ratio estimator derived in the Supplemental Methods Appendix (““Testing Assumptions about Population Distributions within Census Blocks Close to Sea Level”) has two advantages over simply adding all the block-specific estimates: it removes the upward bias from using either of our density assumptions, and it provides an uncertainty range. The ratio estimator is 0.804 with a standard error of 0.129 (Table SA-9), which would imply a coefficient of variation of 0.16 (for the blocks to which it applies, i.e., those that are partly below one meter). The range of 0.804$\pm$0.16 implies that 1.0 is barely more than one standard deviation above the ratio estimator, so if we had a strongly held hypothesis that population below 1 meter is proportional to buildings below 1m, we could not reject such a null hypothesis. But we did not start with such a hypothesis; rather we expected the ratio estimator to be less than 1.0. In some blocks, the footprint data finds a greater proportion of buildings below 1m than a data set created with visual observation, because the building footprint data include a disproportionate number of structures near the shore that are not residential, such as large sheds, docks, boathouses, and piers. Hence there is no need to use the range of ratio estimators for testing a hypothesis; rather we simply use it to estimate the most appropriate multiple. Nevertheless, the use of a simple interpolation approach is more practical, especially for making estimates for which we lack an independent data set, such as areas beyond the mid-Atlantic.

We cannot say for certain how reasonable those ratio estimators are for other regions. The Maryland and rest-of-Mid-Atlantic ratio estimators are similar. That does not necessarily mean that ratio estimators would be same outside the mid-Atlantic; Maryland’s development patterns, flood heights, and regulations may be more similar to those in adjacent states than to those in states elsewhere. Nevertheless, lacking an independent data source for the rest of the United States, the only practical alternative to the combine ratio estimator is the simple building-based density assumption, which in effect, assumes that the ratio estimator is 1.0 with no uncertainty. The ratio estimator for the

mid-Atlantic seems more plausible. The uninhabited structures near the shore that keep the ratio estimator below 1.0 are found in states outside the mid-Atlantic as well. Therefore, our approach is to present both the ratio estimates and estimates based on the straight proportionality of the building-based density assumption. When an uncertainty analysis is needed or useful (see §8), we use the parameters estimated from the independent data set; but for the most part, the function of the ratio estimators and related parameters was to show that the building-based assumption provides a reasonably accurate estimate.

Tables 1 and S1-A show that the population of blocks entirely below 1m for the six mid-Atlantic states is 29,000, while the total estimate below 1m is 203,000; that is, blocks entirely below 1m where the ratio estimate does not apply are only 14 percent of the total. Roughly the same ratio applies to the entire coast excluding Louisiana, Texas, and California, so the coefficient of variation for all blocks is in the 14–16 percent range for most states. See Table S2-A. Since the ratio estimator only applies to the blocks that are partly below 1m, the coefficient of variation (uncertainty range as a percent) will be less for the nationwide estimate than for the mid-Atlantic. Nationwide, blocks entirely below 1m are approximately 40 percent of the total estimated population below 1m; so the coefficient of variation nationwide is less than 8 percent.

We did not estimate ratio estimators for population above 1 meter. The results in Table SA-5 (in in the Test of Method) show that all three approaches provide very similar estimates in Maryland for both the population of units between 1–2m and 2–3m; while Table SA-6 shows that the building-based density approach overestimates units between 1 and 2m by about 16 percent, while understating units between 2–3m by about 8 percent. Combining Maryland and the rest of the mid-Atlantic, the estimate from building-based density for population below 3m is 5.3 percent greater than from the count, and the estimate for land between 1 and 3m is 5.3 percent greater. Hence, deriving and reporting ratio estimates above 1m did not seem worthwhile.

7.5 Limitations of the Study Design

As discussed in both the Methods Section and the Preliminary Test of Method, this study was designed and intended to (a) determine whether estimating populations based on buildings would improve estimates of population close to sea level and (b) measure population trends at various geographical scales using Census block data. The Preliminary Test of Method demonstrated that building density provides a reasonable approach for estimating the portion of a block's housing units that are within a hazard zone. Because units within a block are usually homogeneous, it follows that the buildings also provide a good estimate of the portion of population in the hazard zone. To make the results as useful as possible, we also report changes in populations in hazard zones and ratios of disproportionality between one race and the total population. Although changes and ratios are simple math, measurement errors can compound.

Reasons to be concerned that the reported ratios and changes are less reliable than the totals include:

- As a general rule, the variance of the difference between estimates of two variables is the sum of their variances, unless the errors are correlated. And because the difference is smaller than the original variables, the coefficient of variation is even greater.

- The strata selected for the Preliminary Test of Method were designed to overweight the strata with the greatest populations and severely underweight the strata of unpopulated blocks. That selection looked at total population, rather than Black or Hispanic population. So a significant fraction of populated blocks had no Black population, and the Hispanic population was too small for even a rough assessment of measurement error.
- Maryland, which had about half the blocks in the preliminary test, has a disproportionately low share of black residents living below one meter.

On the other hand, there are some reasons to hope that the reformatted results are about as valid as the original calculations:

- In the sample blocks, Black residents have a greater tendency to inhabit urban areas than rural areas, so by overweighting populated areas we may have over-weighted the areas with the largest concentrations of Black residents, even though our lower density strata included many blocks with no Black population.
- The greater tendency to inhabit urban areas would also make the building density assumption more accurate because that assumption is more accurate in densely populated areas than rural areas.
- Perhaps as a result, the ratio estimator for the Black population is close to 1.0 with a small variance.
- We were able to assess the confidence of the estimated changes following the approach of the Welch's t-test.

Previous studies have generally focused on total population (of all races), which is the most important population variable to measure for many purposes. Nevertheless, because population by race is also reported at the Census block level, it took minimal effort to also calculate the Black and Hispanic population in the various hazard zones. But it is not always easy to interpret the significance of several series of data that are all rising. To make the results more comprehensible, we present various ratios—such as the proportion of people in the hazard zone—and even ratios of ratios, such as the relative proportions of the Black population in the hazard zone compared with the proportion of the total population in the hazard zone (which we call the ratio of disproportionality). But our ability to display results in a format more useful than the original calculations does not necessarily mean that the reformatted results are as reliable as the original calculations. The ability to reformat results in a more meaningful form, such as a ratio, is not the same thing as designing the study to evaluate possible changes in that ratio.

The following §8 describes our error assessment to determine how reliable some of the reformatted results are. While we do derive confidence intervals and significance tests, had we intended to investigate the disproportional habitation and emigration of Black and Hispanic residents from hazard zones, we could have designed the sampling approach differently:

- Instead of merely over-weighting blocks with high population density, our initial stratification for the preliminary assessment could have incorporated racial categories; and blocks with no black residents would have been underweighted.

- Given the rapid rise in Hispanic populations, the second phase of the preliminary assessment would have evaluated alternate approaches to assess measurement error of Hispanic populations in hazard zones.
- The initial stratification over-weighted areas where dikes enable substantial areas of dry land below sea level. We would have instead (or additionally) over-weighted areas with suspected net emigration from low coastal lands.
- The first phase of the preliminary assessment looked at whether single anomalous observations had skewed the ratio estimate (and thus error) of total population for any strata, and whether that situation warranted additional observations for such strata. We could have calculated the measurement error of the various *ratios*, so that we could assess whether single observations skewed the estimated means, variances, or covariances for such strata and whether that warranted additional observations for such strata.

8 Uncertainty and Statistical Significance

The Results section (§3) of the letter whose methods are supplemented here presents results based on the building-density assumption, rather than ratio estimates. While the ratio estimates *should* be more accurate than the building assumption (which seemed to have a mild upward bias in the mid-Atlantic), the building-density calculations flow directly from the input data while the ratio estimates depend on the validity of the preliminary assessment. Given the relatively small set of observations and the limited geographical scope, it seemed more prudent for the results to flow directly from the data, and use the preliminary assessment primarily as a means of validating the method. In this section we go somewhat farther.

An anonymous reviewer of the draft of the letter stated:

> "Black residents are 63% more likely to live below 1m than the general population nationwide" Whether this ratio is statistically different. If not, is it appropriate as a conclusion. Similar descriptions throughout the text also need to be clarified.

In effect, those comments challenged us to provide at least some sense of the uncertainty associated with our results. Because our results are based on a census rather than a sample, statistical significance in the ordinary sense does not apply; but in the larger sense the concept does apply because our results are subject to measurement or model error, rather than sampling error. And the preliminary analysis did obtain a sample of observations for assessing model error. Accordingly, this section evaluates the measurement/model error of the ratio estimates (described in the Preliminary Test) as a means for assessing the error of our various results.[27] Note that tables with the prefix "S" are found in a separate document of supplemental tables.

---

[27]Approaches to assessing measurement error typically involve one of two situations: In the first case, one has independent sets of measurements that are each prone to the same type of error, so that the difference in measurements provides an estimate of the variance of measurement error and the average is presumed to be the best estimate. See e.g. Hansen, M., Hurwitz, W.N., and Pritzker, L. (1964). *The Estimation and Interpretation of Gross Differences and the Simple Response Variance*. In CONTRIBUTIONS TO STATISTICS (presented to P.C. Mahalanobis on the occasion of his 70th birthday), C.R. Rao (ed.). Calcutta: Statistical Publishing Society. In the second case, a more limited set of observations is deemed to be "true". In this case, we are comparing a model to observations,

The overall implications are as follows:

- The coefficient of variation for total population below one meter is approximately 9 percent, though it varies from state-to-state according to the portion of the low-lying population that inhabits blocks that are entirely below one meter: (Table S2-A.) Approximately 3 percent in Louisiana, 7 percent in Texas 9 percent in California, and 13–17 percent in most states.
- The coefficient of variation for Black population is only about 1.5 percent nationwide and for the state of Texas, 0.5 percent for Louisiana, 2.5 percent for California, and between 3 and 4 percent for Maryland and North Carolina, and between 4 and 5 percent for every other state. The apparently greater precision for the Black population results from the greater concentration of Black residents in urban areas where the building density assumption is more accurate.
- The standard error of changes in population below 1m from one decade to the next is about 4 percent of the population at the national scale, about 40 percent of the change in population. In all but five states, the standard error for population change is 6 to 9 percent of the total population, and between 20 and 100 percent of the population change. (§8.1, Table S2-B). Thus increases in population are statistically significant at the national scale, but only for some states.
- The standard error of changes in population below 1 meter over the 30-year period is about 7 percent of the population at the national scale, and about one third of the nationwide population change (excluding New Orleans) over the same period. For 16 states, the standard error of the change in population is between 25 and 50 percent of the estimated change in population, that is, the t-statistic for the population change is between 2.0 and 4.0.
- Approximately 1.175 percent of the US Black population, and 0.65 percent of all US residents live below 1 meter.
- The standard error of the difference between these two proportions is 0.054, making this disproportionality very statistically significant. Outside of New Orleans, however, the proportions are closer: 0.69 and 0.55 percent. This difference is statistically significant as well at a 95% level but not 99%. In 5 states, this disproportionality is statistically significant, in 7 states it is not, and in 12 states, the proportion of Black residents below 1 meter is statistically *less* than the proportion of all residents. (§8.2, Tables S2-C, S2-D, and S-2E).
- Among all counties with apparent net emigration from land below 1 meter, the standard errors of Black residents and all residents are about 1 and 15 percent of the apparent emigration during the last 30 years. The standard errors of the estimates of apparent emigration are 1567 and 19,549, compared with apparent emigration of 98,354 and 169,312, respectively. Thus, emigration is statistically significant for both Blacks and all races. (§8.3, Table S13-C).
- The rate of Black emigration from land below 1m has been 340 and 18 per 100,000 during the last 30 and 10 years, respectively. By contrast, the overall rate of emigration has been 54 and 9.5 per 100,000. The standard error of the difference between these two proportions is 5.3 and 5.7 per 100,000. Thus, the (282) difference for the period 1990–2020 is highly significant, while the difference (8.0) for the decade 2010–2020 is not significant except at the 80% confidence level. While the disproportionality is less when New Orleans is excluded, statistical confidence is about the same (§8.3, Table S13-C).
- In general, the difference between the proportions of Black and total population are statistically significant when the ratio of disproportionality is greater than 1.2 (or less than 0.8). But because the ratios of disproportionality based on the building-density assumption are generally about 10 percent less than the disproportionality based on the ratio estimates, as a general rule, ratios of

---

which we treat as comparable to the latter situation, with observations much more likely to be accurate than the modeled estimate.

disproportionality in tables S7 to S10 or reported in the main paper are likely to be statistically different from 1.0 unless they are between 0.7 and 1.1.

Additional details follow in the next three subsections.

8.1 Uncertainty Regarding Trends

As Table S2-A shows, the coefficient of variation for total population is about 9 percent nationwide but 13 to 16 percent for most states. Population below 1m increased about 12 percent nationwide between 2010 and 2020, and increased by less than the coefficient of variation for all but six states. Thus the measurement error for population below one meter is greater than the decadal population increase for most states, and more than half even for the nationwide population estimate. If the measurement error was entirely random, we might be concerned that our estimated changes are dwarfed by the measurement error. But the measurement errors at a particular block are correlated from decade to decade because the primary source of error (uncertainty about where people live in a given block) largely depends on the location of residential units, which changes slowly. According to a standard theorem, correlation reduces the variance of a difference between two variables:

$$\text{variance}(e_1 - e_2) = \text{variance}(e_1) + \text{variance}(e_2) - 2\ \text{covariance}(e_1, e_2)$$

$$= \text{variance}(e_1) + \text{variance}(e_2) - 2\rho_{1,2}\sqrt{(variance(e_1)variance(e_2)}$$

where $\rho_{1,2}$ is the correlation between e_1 and e_2. In this context, e_1 and e_2 are the model error of our ratio estimates of the populations below 1 meter for the years 2010 and 2020, respectively in Table S2-A, but we still need to estimate $\rho_{1,2}$. Given the linear estimator, the difference in model errors for the two variables is equal to the model error of the difference.

The main paper distinguishes two separate reasons that population below one meter changes from decade to decade: (a) 75% of the increase resulted from sea level rise and (b) 25 percent resulted from people moving into and out of the land below one meter. On average, sea level rise should not change overall measurement error.[28] Therefore, the change in measurement error results from people moving into and out of the blocks.

We calculated the model error for every block in our Mid-Atlantic data set using the ratio estimates for the years 2010 and the data from the sample blocks in the preliminary test of method. Because block boundaries change from census to census, we used the NHGIS estimates of 2020 population by 2010 census block, and calculated model error for each of the blocks.[29] See Tables SM-7 and SM-8. The correlation coefficient between the error estimates for the two years was 0.873. Table S2-B shows the resulting standard error estimates for the change in population between 2010 and 2020. For most states, the standard error for the change in population is approximately half the standard error for the population estimates for 2010 and 2020. The standard error for the nationwide population

---

[28] The percent of each block inundated across all blocks should be uniformly distributed if blocks are drawn without regard to elevation, so as long as there is a general cumulative distribution function F(x) where x is the percent of the block below 1 m and F(x) is the percent of the block's population below 1 m, as sea level increases the distribution across all blocks that are only partially below 1m will remain the same, and thus so will the total of all F(x).

[29] Schroeder, J. P. (2007). "Target-density weighting interpolation and uncertainty evaluation for temporal analysis of census data." Geographical Analysis 39(3), 311–335. http://dx.doi.org/10.1111/j.1538-4632.2007.00706.

change is 4.2% of the estimated 2020 population below 1m, and about 40 percent of the population change for 2010–2020. For all but 4 states, the standard error of the population change is between 5 and 9 percent of the 2020 population. For 10 states, the standard error of population change is less than 50 percent of the estimated population change for the decade (i.e., a t-statistic greater than 2.0).

Given these calculations, when it is important to indicate the uncertainty range of changes in population, it may be reasonable to indicate that the uncertainty is on the order of 5 to 9 percent of the population of 2020. For the 30-year period, a similar logic implies a correlation of 0.66 and thus a standard error for the nationwide change equal to about 9% of the population of 2010, and 10 to 14 percent of the 2020 population for most states (Table S2-B).

Table SM-7 Sample Statistics for Error of Ratio Estimate of Population for 2010 and 2020

		2020[1]			2010			Correlation between the two years		
Stratum[2]	n_r[3]	Mean Error	Intra-stratum sample variance[4]	Interstrata sample variance[4]	Mean Error	Intra-stratum sample variance[4]	Inter-strata sample variance[4]	Sample Intrastrata covariance	Sample Interstrata covariance	sample ρ[4]
A1bi	39	3.307	10,446.9	10.94	20.7	33,403	427.6	16,437.0	68.38	0.880
A1bii	40	-0.653	21.8	0.43	-2.50	44.0	6.3	28.4	1.63	0.917
A2bi	15	0.275	0.9	0.08	0.01	0.0	0.0	0.0	0.00	0.042
A2bii	7	-1.227	7.6	1.50	-1.78	8.3	3.2	7.74	2.19	0.976
C	19	-3.633	153.9	13.20	-7.44	410.9	55.3	222.8	27.02	0.886
2	22	-0.246	1.2	0.06	-1.52	27.5	2.3	3.9	0.37	0.666
4	25	4.787	424.28	22.91	2.84	166.7	8.0	265.2	13.57	0.997
5	57	0.292	1.33	0.085	-0.04	1.4	0.0	1.14	-0.01	0.846
Total	224	0.000			0.00					

1. Error in projecting 2020 population of 2010 census blocks.
2. See Preliminary Test of Approach Appendix for definitions of the strata.
3. n_r is the relevant sample size for the comparison reported in this table.
4. Intrastrata variance is the sample variance within a stratum, including the finite correction factor. Interstrata variance is the square of the difference between the sample mean of the strata and the weighted population mean.
5. Sample correlation coefficient for a given stratum, based on intrastratum covariance and variance.

Table SM-8: Estimates of Total Strata Error of Ratio Estimate of Population for 2010 and 2020[1]										
				2020			2010			strata
Stratum[2]		$\widehat{N_r}$[3]		Strata Error[4]	Contribution to Variance[5]		Strata Error[4]	Contribution to Variance[5]		contribution to covariance[6]
A1bi		1502		4,967	520.33		31,058	1683.3		821.2
A1bii		8705		-5,685	6.42		-21,769	14.5		8.7
A2bi		10258		2,819	0.33		84	0.0004		0.001
A2bii		4710		-5,777	1.42		-8,395	1.8		1.5
C		350		-1,271	1.94		-2,603	5.4		2.9
2		414		-102	0.02		-628	0.4		0.06
4		847		4,055	12.55		2,402	4.9		7.8
5		3402		994	0.16		-150	0.156		0.13
Total		30188		0.000	543.18		0	1,710		842
**Weighted Correlation Coefficient Between Error for 2010 and 2020: 0.874**[7]										

1. The relevance to these statistics is that they are used to properly weight the strata to derive the weighted error correlation coefficient.
2. See Preliminary Test of Approach Appendix for definitions of the strata.
3. $\widehat{N_r}$ is estimated number of blocks with some land below one meter.
4. Calculated as the mean error times $\widehat{N_r}$
5. Calculated as the sum of intrastratum variance plus interstrata variance, times the ratio of $\widehat{N_r}$ for the strata to the sum of $\widehat{N_r}$ across all strata.
6. Calculated as the sum of intrastratum covariance plus interstrata covariance, times the ratio of $\widehat{N_r}$ for the strata to the sum of $\widehat{N_r}$ across all strata.
7. Calculated as 842 $(1{,}700 \text{ X } 543.18)^{1/2}$

8.2 Statistical Significance of the Disproportionality

This section focuses on an anonymous reviewer's comment about a statement in the abstract: "'Black residents are 63% more likely to live below 1m than the general population nationwide,'" and suggested that the paper should clarify "…[w]hether this ratio is statistically different. If not, is it appropriate as a conclusion?"

Similar studies on populations potentially exposed to climate change[30] and other environmental hazards have generally provided best estimates—often using alternative assumptions—but not assessed statistical significance of their results. In one sense, because these studies (including this study) are based on a census, the concept of statistical significance as commonly applied is not relevant. (Or equivalently, because the sampling fraction is 100% the finite population correction reduces the variance to zero). Yet the results are not precisely accurate, because of measurement and model error [hereinafter "measurement error".]

As discussed elsewhere in this paper and supplementary material, we have estimated the error from the building-based density and uniform density assumptions, as well as from ratio estimates based on area and building below a given elevation. The Preliminary Test of Method (Appendix) develops ratio estimators $\hat{R}$ for the equations we use to estimate total and Black populations below one meter as a function of buildings below one meter in blocks that are partly below one meter.

[30] E.g. papers cited in notes 7,8, & 11.

$$Population_{1m} = \hat{R} \sum_i (B_i^{1m}/B_i)\ P_i\ ,$$

where $\boldsymbol{P_i}$ is the population of the ith block, B_i is the number of buildings in that block, and B_i^{1m} is the number of buildings on land below 1m in that block. Table SA-7 and SA-9 (in the Preliminary Test) report values of $\hat{R}$ for total and Black population of 0.804 and 0.961, respectively, with standard deviations of 0.129 and 0.044.[31] Table S2-A provides state-by-state ratio estimates of the total population below one meter, as well as the standard deviations of those estimates. Because the ratio estimates only apply to blocks that are partly below one meter, the coefficient of variation in Table S2-A is small in states where most of the population below 1 meter resides in blocks that are entirely below one meter, such as Louisiana. (Because the ratio estimator for the Black population is close to 1.0 with a small variance, such a table for the Black population would be similar to Table S5-A, which shows the Black population based on the building density assumption.)

Table S2-C shows the comparable ratio estimates of the total ("All") and Black populations below 1 meter as a percentage of the total and Black populations at all elevations for each of the states (as well as the United States including or excluding New Orleans). Because the total population at all elevations is essentially a scalar based on Census data, the statistical properties of these proportions are essentially the same as the ratio estimates of total population. The ratio of disproportionality discussed in the main letter and this supplement is the ratio of the "Black" to the "All" proportion. For the United States, including and excluding New Orleans for the year 2020, for example, these ratios would be 1.82 (i.e., 1.177/0.645) and 1.27 (i.e.,0.694/0.547), respectively. (These ratios are higher than the building-based ratios of disproportionality reported in the main paper because the ratio estimate of total population below one meter is about 20 percent less than the estimate from building based density, and almost all the results in the main paper are based on the building density assumption rather than the ratio estimate.)

The starting point for evaluating whether the differences between the "Black" and "All" proportions are statistically significant is the familiar theorem on the variance of a difference<:

$$\text{variance}(y_1-y_2) = \text{variance}(y_1) + \text{variance}(y_2) - 2\ \text{covariance}(y_1, y_2)$$

$$= \text{variance}(y_1) + \text{variance}(y_2) - 2\rho_{1,2} \sqrt{(variance(y_1) variance(y_2))}$$

where $\rho_{1,2}$ is the simple correlation coefficient between y_1 and y_2. In this case, we can use y_1 to represent the total population while y_2 represents the Black population. Although we have estimates of the variances of y_1 and y_2 (e.g., Table S2-A) we do not have an estimate of $\rho_{1,2}$. A priori, we would expect a positive correlation, however, because (a) total population is the sum of Black population and nonblack population, and (b) the population of blocks varies greatly, from rural blocks with only a few people, to urban blocks with hundreds of people. Thus heavily populated blocks will tend to have more Blacks and nonblacks than sparsely populated blocks.

A convenient but *ad hoc* approach is to estimate $\rho_{1,2}$ by calculating the cross products of the estimated total and Black population below one meter across all census blocks (See Table S2-C). Nationwide, the correlation is about 0.5. We say "ad hoc" because calculating a sample correlation

[31] The tables also report ratio estimators based on the uniform density assumption, which are much less accurate.

coefficient would be the standard practice if one was measuring the variance of sampling error; but because we have the universe of all census blocks sampling error is not the problem. Rather the variance of our ratio estimates results from measurement error, so we need to know the covariance of measurement error, that is, we need to calculate the correlation and covariance of e_1 and e_2, were

$$e_{1,i} = y_{1,i} - \hat{R}_1 x_{1,i} \,,$$

and $y_{1,i}$ is the total population below one meter for the ith block, $x_{1,i}$ is the estimated total population below one meter for the ith block based on building-based density, $\hat{R}_1$ is the combined ratio estimator from Table SA-7, and thus $\hat{R}_1 x_1$ is the ratio estimate of total population for the ith block, and $e_{2,I}$ is defined analogously for Black population. In this context, the variance of the ratio estimates is the same as the variance of the error of the ratio estimates; the standard deviations reported in Table S2-A are simply the square roots of those variances. If population measurement error tends to be proportional to population, and thus also proportional to the ratio estimate, we would expect the correlation of our ratio estimates to be a good measure of the correlation of the errors. The left portion of Table S2-D displays the correlation between estimated Black and total population. Nationwide, the correlation gradually declined from 0.57 to 0.52 from 1990 to 2020, though it varies across states.

The stratified sample of observations from remote sensing used to derive the ratio estimators in the Preliminary Test is also a sample for estimating the covariance and correlation of e_1 and e_2. Tables SM-9 displays sample error statistics for the various strata, while Table SM-10 displays the estimated total variance and covariance by stratum, the total of which is used to calculate the overall correlation

Table SM-9 Sample Statistics for Error of Ratio Estimate of Black and Total Population

		Black Population			All Races Population					
Stratum[1]	n_r [2]	Mean Error	Intra-strata sample variance[3]	Inter-strata sample variance[3]	Mean Error	Intra-strata sample variance[3]	Inter-strata variance[3]	Sample Intrastratum covariance	Sample Interstrata covariance	sample ρ[4]
A1bi	25	2.051	23.6	4.21	16.73	6,842.5	262.9	135.9	33.26	0.338
A1bii	15	-0.616	6.5	0.38	-1.74	20.1	5.0	10.5	1.38	0.925
A2bii	1	-0.226	0.1	0.05	-0.82	0.7	1.8	0.18	0.30	1.000
C	9	0.251	2.5	0.06	-2.53	50.8	9.2	6.6	-0.76	0.592
2	9	-0.445	1.5	0.20	-1.41	6.9	3.7	3.0	0.85	0.937
4	8	0.244	0.47	0.06	7.61	528.5	50.5	15.8	1.73	0.999
5	18	0.037	0.04	0.001	-0.25	0.7	0.6	0.10	-0.03	0.569
Total[5]		0.000			0.51					

1. See Preliminary Test of Approach Appendix for definitions of the strata.
2. n_r is the relevant sample size for the Black-to-All comparison reported in this table. Blocks with no Black population are excluded.
3. Intrastratum variance is the sample variance within a stratum, including the finite correction factor. Interstrata variance is the square of the difference between the sample mean of the strata and the weighted population mean.
4. Sample correlation coefficient for a given stratum, based on intrastratum covariance and variance.
5. The nonzero mean error results from the fact that blocks with no Black population are excluded from this comparison, were included in the calculation of the ratio estimator. In addition, calculations are based on our reported ratio estimate, which was originally based on housing unit population.

coefficient of the error for Black and All populations, which is 0.3384.[32] The left portion of Table S2-E displays the difference between the proportion of Black residents living below one meter and the proportion of all residents living below one meter, along with our standard error of the estimate of that difference.

Table SM-10 Estimates of Total Strata Error of Ratio Estimates for Black and Total Population[1]										
				Black Population			All Races			strata
Stratum[2]		$\widehat{N_r}$[3]		Strata Error[4]	Contribution to Variance[5]		Strata error[4]	Contribution to Variance[5]		contribution to total covariance[6]
A1bi		963		1,975	2.74		16,103	699.5		16.65
A1bii		3264		-2,012	2.28		-5,665	8.4		3.98
A2bii		673		-152	0.01		-548	0.2		0.03
C		158		40	0.04		-398	1.0		0.09
2		414		-184	0.07		-582	0.4		0.16
4		847		207	0.05		6,451	50.2		1.52
5		3462		127	0.02		-855	0.4		0.02
Total		9780		0.000	5.21		14,505	760		22
**Correlation Coefficient: 0.3570**										

1. The relevance to these statistics is that they are used to properly weight the strata to derive the weighted error correlation coefficient.
2. See Preliminary Test of Approach Appendix for definitions of the strata.
3. $\widehat{N_r}$ is estimated number of blocks with Black population, calculated as N n_r/n for each stratS2--Bum.
4. Calculated as the mean error times $\widehat{N_r}$
5. Calculated as the sum of intrastratum variance plus interstrata variance, times $\widehat{N_r}$ for the strata divided by the sum of $\widehat{N_r}$ across all strata.
6. Calculated as the sum of interstrata covariance plus interstrata covariance, times $\widehat{N_r}$ for the strata divided by the sum of $\widehat{N_r}$ across all strata.
7. Calculated as the total covariance divided by the square root of the product of the variances for the two data series.

Given our estimate of the means and the standard deviation of the difference, we can test the statistical significance of the difference in the two mean proportions using Welch's-t test and the Student's t-distribution. Because we have stratified sampling, the degrees of freedom for purposes of evaluating statistical significance is not simply the number of observations minus the number of free parameters[33], but instead requires one to use the Welch–Satterthwaite equation, which yields 14.19 degrees of

[32] The estimated correlation of 0.34 is surprisingly low, perhaps unrealistically so. This low correlation probably results from the fact that for purposes of calculating correlation, we use housing units (which we observe directly) rather than the resulting population estimate. Areas with unusually high population density per unit would also have greater human population error, but not greater unit population error. This underestimate of the correlation would cause the variance and standard error to be an overestimate, which is acceptable for our purposes.

[33] See Lohr, S.L., 2021. Sampling: design and analysis (second edition). Chapman and Hall/CRC. p. 48 (citing Satterthwaite, F. E. (1946), "An Approximate Distribution of Estimates of Variance Components.", *Biometrics Bulletin*, 2 (6): 110–114).

freedom. Applying the Welch's t-test in this case merely requires us to look up the appropriate ratio values from the Student's t-distribution for 14 degrees of freedom, which are 1.7613 and 2.1448 for 90 and 95% confidence (somewhat greater than the 1.65 and 1.96 in the standard normal distribution). Thus, wherever the difference in means is at least 1.7613 or 2.1448 times the estimated standard deviation, the difference in means is statistically significant at the 90 and 95% levels of confidence, respectively.

The center four columns of Table S2-D and S2-E display the t-ratios (ratio of the standard deviation of the difference to the difference) for each state. for each year. in those cases where the t-ratio is greater than 1.763, for the two methods of calculating the error correlation. Table S2-E shows, for example, that the portion of Black residents living below 1 meter is significantly greater than the portion of all residents below 1 meter in New York, Pennsylvania, Louisiana, Texas, and the United States as a whole, including or excluding New Orleans. Nationwide, the t-ratio is approximately 10. Conversely, the t-ratios are significantly negative for New Hampshire, Rhode Island, and every state from Delaware to Mississippi, as well as Oregon.

The right four columns of Tables S2-D and S2-E show the comparable ratios of disproportionality (based on the ratio estimates) where the differences between the proportions are statistically significant. In general, the differences are statistically significant when the ratio of disproportionality is greater than 1.2 (or less than 0.8). Note, however, that the ratios of disproportionality based on the building-density assumption are generally about 10 percent less than the disproportionality based on the ratio estimates. (The projected ratio estimates of total population are 10 to 15 percent less than the building-based estimate, while the ratio estimates of Black population is only a few percent less than the building based estimate). Therefore, as a general rule, ratios of disproportionality in tables S7 to S10 or reported in the main paper are likely to be statistically different from 1.0 unless they are between 0.7 and 1.1.

8.3 Disproportionality of Emigration from Land Below 1 meter

The impact of measurement on the estimates of apparent emigration from land below one meter is similar to that for changes in population discussed in §8.1. The apparent emigration is simply the change in population after netting out the change in population due to sea level rise. Although sea level rise is responsible for 75% of the population change, it has a negligible contribution to the measurement error from our ratio estimate[34], so excluding that contribution does not reduce the measurement error. Therefore confidence intervals of migration are possible based on the standard errors discussed in §8.1.

As Table S13-C shows, the standard error for residents below 1m in counties with net emigration ranges between 1110–1535 for Black residents and 16,305–27,949 for all residents for the four decades, and the standard error for changes in population are 1567 and 19,549. Estimated emigration of Blacks and all residents was 98,354 and 169,312 for the period 1990–2020, several times the standard errors of

[34] The measurement error results from the inherent mismatch between where buildings are located within a census block, and where people actually live. The fraction of census blocks below 1m is fairly uniformly distributed, so rising sea level will both eliminate measurement error in blocks that convert from partly below 1m to entirely below 1m, and add measurement error as blocks that had been entirely above 1m become partly below 1m; in addition, the vast majority of people within a block either remain above or remain below 1m. By contrast, population changes from decade to decade for all other reasons result from both changes in block boundaries and possibly development at elevations not previously developed within the block, adding error to any attempt to measure the true change in population below 1m.

the change in population. Emigration is statistically significant (<0.00001) over the 1990–2020 period for both black and all residents, even excluding New Orleans (0.00029 for all races). Given the well-known exodus after Hurricane Katrina, this result would be expected. For the period 2010 to 2020, however, the results are less clear: Black emigration is still very significant (0.000739) but for all residents the standard error is about as great as the estimated emigration. The result is still suggestive, but the total emigration is statistically significant only at the 70% level of confidence.

The main letter also reports that Black residents are almost five times as likely to have emigrated from land below one meter as the population as residents of all races. Because evaluating the statistical significance of a ratio is cumbersome, we convert all the population estimates to proportions as a share of total population, as in §8.2. We can then evaluate statistical significance by defining change in disproportionality (ΔD) as:

$$\Delta D = (p_{Black,2020} - p_{All,2020}) - (p_{Black,2010} - p_{All,2010})$$

where p_{Black} and p_{all} are the number of Black residents and residents of all races below 1 meter in the relevant counties, divided by the nationwide total of Black and all residents, respectively. Calculating this variance requires estimates of various correlation coefficients. Sections 8.1 and 8.2 already discuss our sample estimates for covariance($p_{All,2020}$ $p_{All,2010}$) and covariance($p_{Black,2010}$,$p_{All,2010}$), respectively.[35] We assume that the correlation for ($p_{Black,2020}$ $p_{Black,2010}$) is the same as ($p_{All,2020}$ $p_{Allk,2010}$), and that the correlation between black and all residents is the same in other decades as for 2010. That leaves us with having to estimate the correlation($p_{all,2020}$ $p_{Black,2010}$) and ($p_{Black,2020}$ $p_{,2010}$), which we assume to be the same. Tables SM-11 and S-12 show the results of comparing the relevant measurement error. Surprisingly, we get a correlation of 0.43, which is greater than our estimate of correlation of ($p_{Black,2010}$,$p_{All,2010}$). This anomalous result suggests a limitation in the data set we used to assess error, possibly created by the disproportionate impact of a few densely populated blocks on the sample results, but more likely resulting from the correlation of of ($p_{Black,2010}$,$p_{All,2010}$) being too low.[36] The effect of the unexpectedly high correlation is to modestly increase the estimated variance since both variables have the same sign in the sum that defines ΔD, and thereby reduce statistical significance.

The bottom of Table 13-C shows estimates of the disproportionate apparent emigration of Black residents from land below 1m. Over the 1990–2020 period, the rate of Black emigration was 337 per 100,000 residents, while the rate for all residents was only 54.2. Thus the "excess rate of Black emigration" (i.e. ΔD), was 282.5, much greater than the standard error (ΔD) which was only 5.3. Excluding New Orleans, ΔD was only 30.1, which is still very significant given the standard error of 3.9. Considering only the period 2010 to 2020, however, ΔD is only 8 residents per 100,000, which is not much greater than the standard error of 5.6, and hence this result is only significant at the 0.1 level of significance (e.g. 80 percent confidence interval).

[35] The correlation of these proportions is the same as correlation for the actual values.

[36] See note 32.

Table SM-11 Sample Statistics for Error of Ratio Estimate of Black (2010) and Total Population (2020)										
		Black Population			All Races Population					
Stratum[1]	n_r [2]	Mean Error	Intra-strata sample variance[3]	Inter-strata sample variance[3]	Mean Error	Intra-strata sample variance[3]	Inter-strata variance[3]	Sample Intrastratum covariance	Sample Interstrata covariance	sample ρ[4]
A1bi	25	2.051	23.6	4.21	23.19	8,515.3	505.1	189.5	45.69	0.422
A1bii	15	-0.616	6.5	0.38	-1.61	56.4	5.4	16.6	1.56	0.870
A2bii	1	-0.226	0.1	0.05	-1.67	2.8	5.6	0.38	0.58	1.000
C	9	0.251	2.5	0.06	-4.77	137.5	30.0	10.4	-1.43	0.563
2	9	-0.445	1.5	0.20	-0.68	1.0	1.9	1.1	0.71	0.863
4	8	0.244	0.47	0.06	12.99	1,367.8	150.8	25.4	2.95	1.000
5	18	0.037	0.04	0.001	0.00	1.4	0.5	0.18	-0.03	0.750
Total[5]		0.000			0.91					

1. See Preliminary Test of Approach Appendix for definitions of the strata.
2. n_r is the relevant sample size for the Black-to-All comparison reported in this table. Blocks with no Black population are excluded.
3. Intrastratum variance is the sample variance within a stratum, including the finite correction factor. Interstrata variance is the square of the difference between the sample mean of the strata and the weighted population mean.
4. Sample correlation coefficient for a given stratum, based on intrastratum covariance and variance.
5. The nonzero mean error results from the fact that blocks with no Black population are excluded from this comparison, but were included in the calculation of the ratio estimator for total population.

Table SM-12 Estimates of Total Error of Ratio Estimates for Black (2010) and Total Population (2020)[1]						
		Black Population		All Races		strata contribution to total covariance[6]
Stratum[2]	$\widehat{N_r}$ [3]	Strata Error[4]	Contribution to Variance[5]	Strata error[4]	Contribution to Variance[5]	
A1bi	963	1,975	2.74	22,325	150182.6	23.15
A1bii	3264	-2,012	2.28	-5,260	245.7	6.06
A2bii	673	-152	0.01	-1,122	0.3	0.07
C	158	40	0.04	-751	15.8	0.14
2	414	-184	0.07	-281	0.5	0.08
4	847	207	0.05	11,008	2302.1	2.45
5	3462	127	0.02	0	0.0	0.05
Total	9780	0.000	5.21	25,919	152,747	32
**Correlation Coefficient: 0.434079**						

1. The relevance to these statistics is that they are used to properly weight the strata to derive the weighted error correlation coefficient, needed to estimate confidence intervals and statistical significance.
2. See Preliminary Test of Approach Appendix for definitions of the strata.
3. $\widehat{N_r}$ is estimated number of blocks with Black population, calculated as N n_r/n for each stratS2--Bum.
4. Calculated as the mean error times $\widehat{N_r}$
5. Calculated as the sum of intrastratum variance plus interstrata variance, times $\widehat{N_r}$ for the strata divided by the sum of $\widehat{N_r}$ across all strata.
6. Calculated as the sum of interstrata covariance plus interstrata covariance, times $\widehat{N_r}$ for the strata divided by the sum of $\widehat{N_r}$ across all strata.
7. Calculated as the total covariance divided by the square root of the product of the variances for the two data series.

References.

Gill, S. K., Wright, R., Titus, J. G., Kafalenos, R., & Wright, K. 2009. Population, land use, and infrastructure. In: Climate Change Science Program. Coastal Sensitivity to Sea-Level Rise: A Focus on the Mid-Atlantic Region. Climate Change Science Program, Washington DC, pp. 105-116.

Hansen, M., Hurwitz, W.N., and Pritzker, L. (1964). *The Estimation and Interpretation of Gross Differences and the Simple Response Variance*. In CONTRIBUTIONS TO STATISTICS (presented to P.C. Mahalanobis on the occasion of his 70th birthday), C.R. Rao (ed.). Calcutta: Statistical Publishing Society.

Hauer, M. E., Hardy, D., Kulp, S. A., Mueller, V., Wrathall, D. J., & Clark, P. U. 2021. Assessing population exposure to coastal flooding due to sea level rise. *Nature communications*, *12*(1), 1-9; Strauss et al., supra note 7; Radeloff et al., supra note 11.

Kulp, S. A., & Strauss, B. H. 2019. New elevation data triple estimates of global vulnerability to sea-level rise and coastal flooding. Nature communications, 10(1), 1-12;

Lohr, S.L., 2021. Sampling: design and analysis (second edition). Chapman and Hall/CRC. p. 48 (citing Satterthwaite, F. E. (1946), "An Approximate Distribution of Estimates of Variance Components.", *Biometrics Bulletin*, **2** (6): 110–114).

McMichael, C., Dasgupta, S., Ayeb-Karlsson, S., & Kelman, I. 2020. A review of estimating population exposure to sea-level rise and the relevance for migration. Environmental Research Letters, 15(12), 123005; and

Microsoft US Building Footprints. https://github.com/Microsoft/USBuildingFootprints (accessed November 19, 2021).

Mondal, P., & Tatem, A. J. 2012. Uncertainties in measuring populations potentially impacted by sea level rise and coastal flooding. PLoS One, 7(10), e48191.

NOAA Sea Level Rise Viewer DEM. https://coast.noaa.gov/htdata/raster2/elevation/SLR_viewer_DEM_6230/ , (Accessed 11/19/2021).

National Ocean Service, CO-OPS. NOS_Observations/CO_OPS_Products (FeatureServer), Sea Level Trends Stations. https://idpgis.ncep.noaa.gov/arcgis/rest/services/NOS_Observations/CO_OPS_Products/FeatureServer (Accessed 11/19/2021).

National Oceanic and Atmospheric Administration. Vertical Datum Transformation https://vdatum.noaa.gov/welcome.html ((Accessed 11/19/2021).

Robert K. Nelson, LaDale Winling, Richard Marciano, Nathan Connolly, et al., “Mapping Inequality,” American Panorama, ed. Robert K. Nelson and Edward L. Ayers, https://dsl.richmond.edu/panorama/redlining/ (updated 12/17.2020). Accessed February 1, 2022

Neumann, Barbara, Athanasios T. Vafeidis, Juliane Zimmermann, and Robert J. Nicholls. 2015. "Future coastal population growth and exposure to sea-level rise and coastal flooding-a global assessment." PloS one 10, no. 3 (2015): e0118571;

Radeloff, V.C., D. P. Helmers, H. A. Kramer, M. H. Mockrin, P. M. Alexandre, A. Bar-Massada, T. J. Hawbaker, S Martinuzzi, A D. Syphard, and S. I. Stewart. 2018. “Rapid growth of the US wildland-urban interface raises wildfire risk.” PNAS 115 (13) 3314-3331.

Schneider, S. H., & Chen, R. S. 1980. Carbon dioxide warming and coastline flooding: physical factors and climatic impact. Annual review of energy, 5(1), 107-140 (counties).

Schroeder, J. P. (2007). "Target-density weighting interpolation and uncertainty evaluation for temporal analysis of census data." Geographical Analysis 39(3), 311–335. http://dx.doi.org/10.1111/j.1538-4632.2007.00706.

Smiley, K. T. 2020. Social inequalities in flooding inside and outside of floodplains during Hurricane Harvey. Environmental Research Letters, 15(9), 0940b3 (block groups).

Strauss, BH, R Ziemlinski, JL Weiss, and JT Overpeck. 2012. Tidally adjusted estimates of topographic vulnerability to sea level rise and flooding for the contiguous United States Environmental Research Letters 7, 014033.

Titus, J. G., et al. 2009. State and local governments plan for development of most land vulnerable to rising sea level along the US Atlantic coast. Environmental Research Letters, 4(4), 044008.

Titus, J.G., 2023a. Population in floodplains or close to sea level increased in US but declined in some counties—especially among Black residents. *Environmental Research Letters*, *18*(3), p.034001.

Titus, J.G., 2023b. Supplemental Tables (Part 1: Portrait) from 'Population in floodplains or close to sea level increased in US but declined in some counties–especially among Black residents'. Environmental Research Letters.

Titus, J.G., 2023c. Supplemental Tables (Part 2: Landscape) from 'Population in floodplains or close to sea level increased in US but declined in some counties–especially among Black residents'. Environmental Research Letters.

United States Geological Survey. 3 DEP Elevation Program. https://www.usgs.gov/core-science-systems/ngp/3dep (Accessed 11/20/2021).

Yang, X., Yao, C., Chen, Q., Ye, T., & Jin, C. 2019. Improved estimates of population exposure in low-elevation coastal zones of China. International Journal of Environmental Research and Public Health, 16(20), 4012.
