# Supplementary material for: Population in floodplains or close to sea level increased in US but declined in some counties—especially among Black residents
Source: Environ Res Lett. Author manuscript; Available in PMC 2025 Mar 14. (PMC11908447; doi:10.1088/1748-9326/acadf5)
Supplement: County Thematic Maps [file NIHMS1876714-supplement-County_Thematic_Maps.pdf]

Supplemental Thematic County Maps from 'Population in floodplains or close to sea level increased in US but declined in some counties–especially among Black residents'

JAMES G TITUS

ENVIRONMENTAL RESEARCH LETTERS

FEBRUARY 2023

Note: This document contains a subset of the thematic county maps created in the analysis described in the aforementioned paper in Environmental Research Letters. To obtain the other maps or underlying data whence those maps were created, go to EPA's Science Hub at https://doi.org/10.23719/1527848

Change in Population Vulnerable to Sea Level Rise by County

1990 to 2020

These maps show county-specific estimates of the change in populations less than one meter or less than three meters above sea level, as defined and estimated in the paper for which this document provides supplemental maps. Because the sea level rise rising, the population changes in these maps reflect both the effect of apparent migration (net change from migration, births, and deaths) and homes that were more than one (or three) meters above sea level in 1990 but less than one (or three) meters above sea level by 2020. For further details, see the main paper and the Supplemental Methods associated with this analysis.

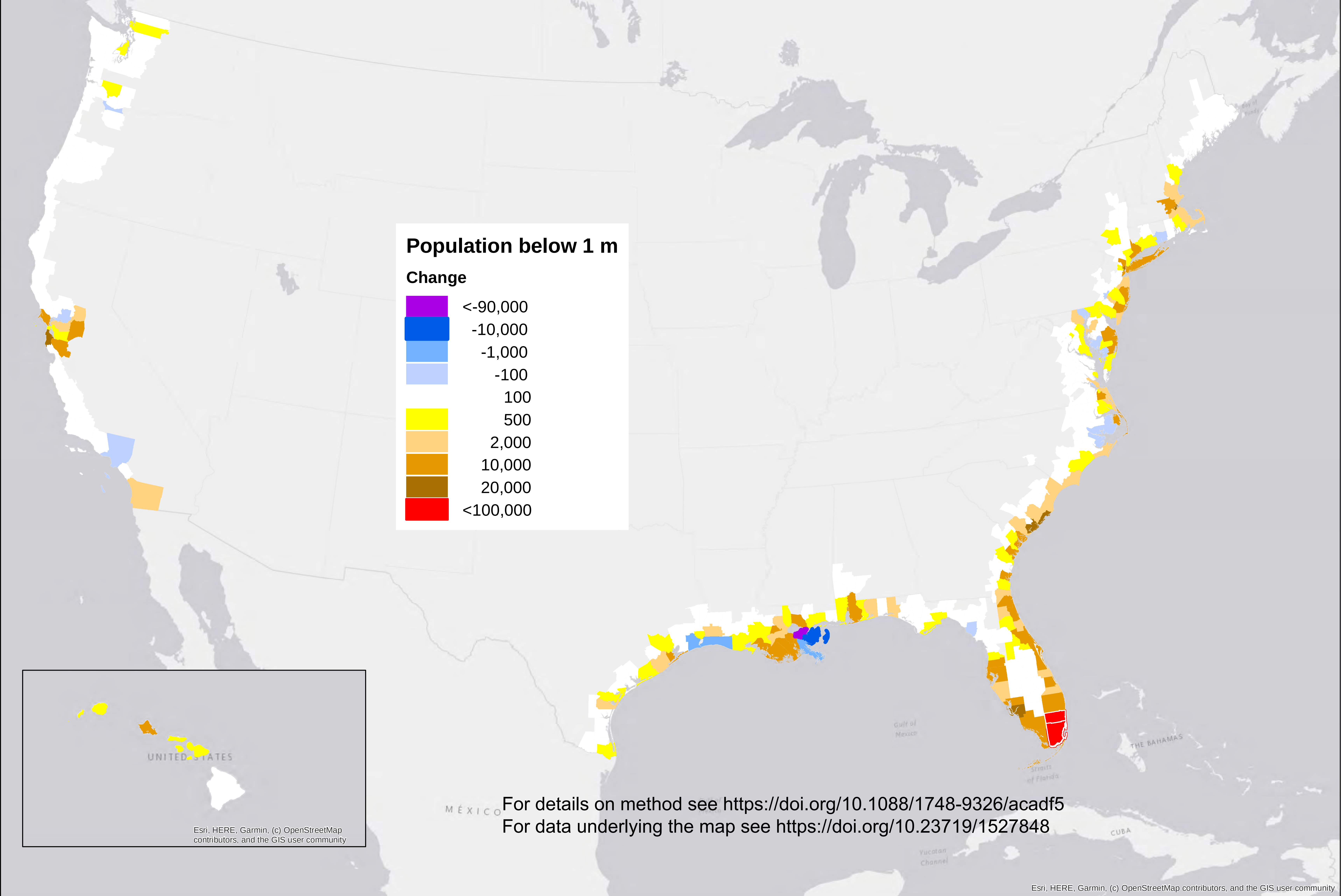
Population below 1 m
Change
<-90,000
-10,000
-1,000
-100
100
500
2,000
10,000
20,000
<100,000
UNITED STATES
MÉXICO
THE BAHAMAS
CUBA
For details on method see https://doi.org/10.1088/1748-9326/acadf5
For data underlying the map see https://doi.org/10.23719/1527848
Esri, HERE, Garmin, (c) OpenStreetMap contributors, and the GIS user community
Esri, HERE, Garmin, (c) OpenStreetMap contributors, and the GIS user community

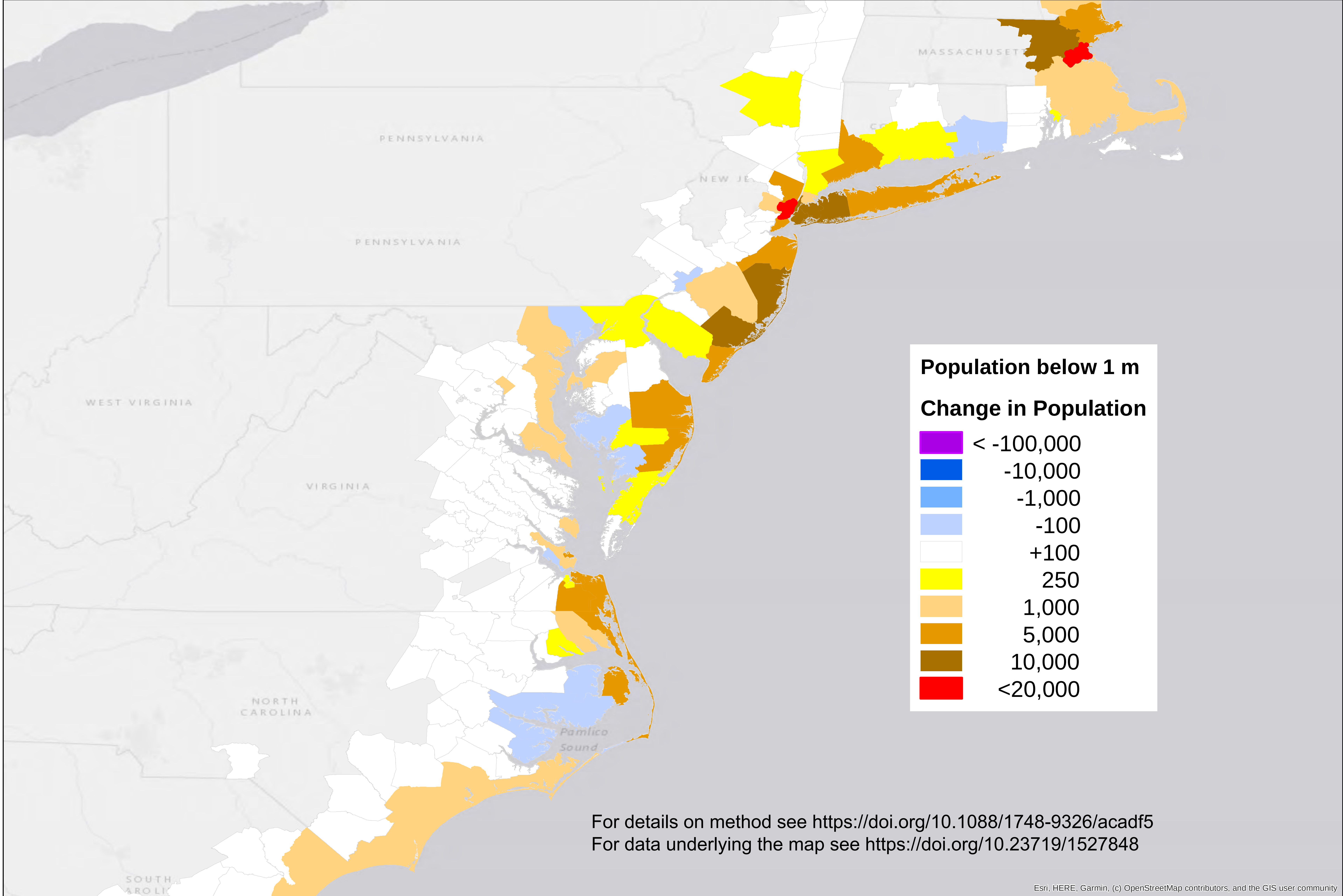
PENNSYLVANIA
PENNSYLVANIA
NEW JE
MASSACHUSETT
WEST VIRGINIA
VIRGINIA
NORTH CAROLINA
SOUTH CAROLI
Pamlico Sound
Population below 1 m
Change in Population
< -100,000
-10,000
-1,000
-100
+100
250
1,000
5,000
10,000
<20,000
For details on method see https://doi.org/10.1088/1748-9326/acadf5
For data underlying the map see https://doi.org/10.23719/1527848
Esri, HERE, Garmin, (c) OpenStreetMap contributors, and the GIS user community

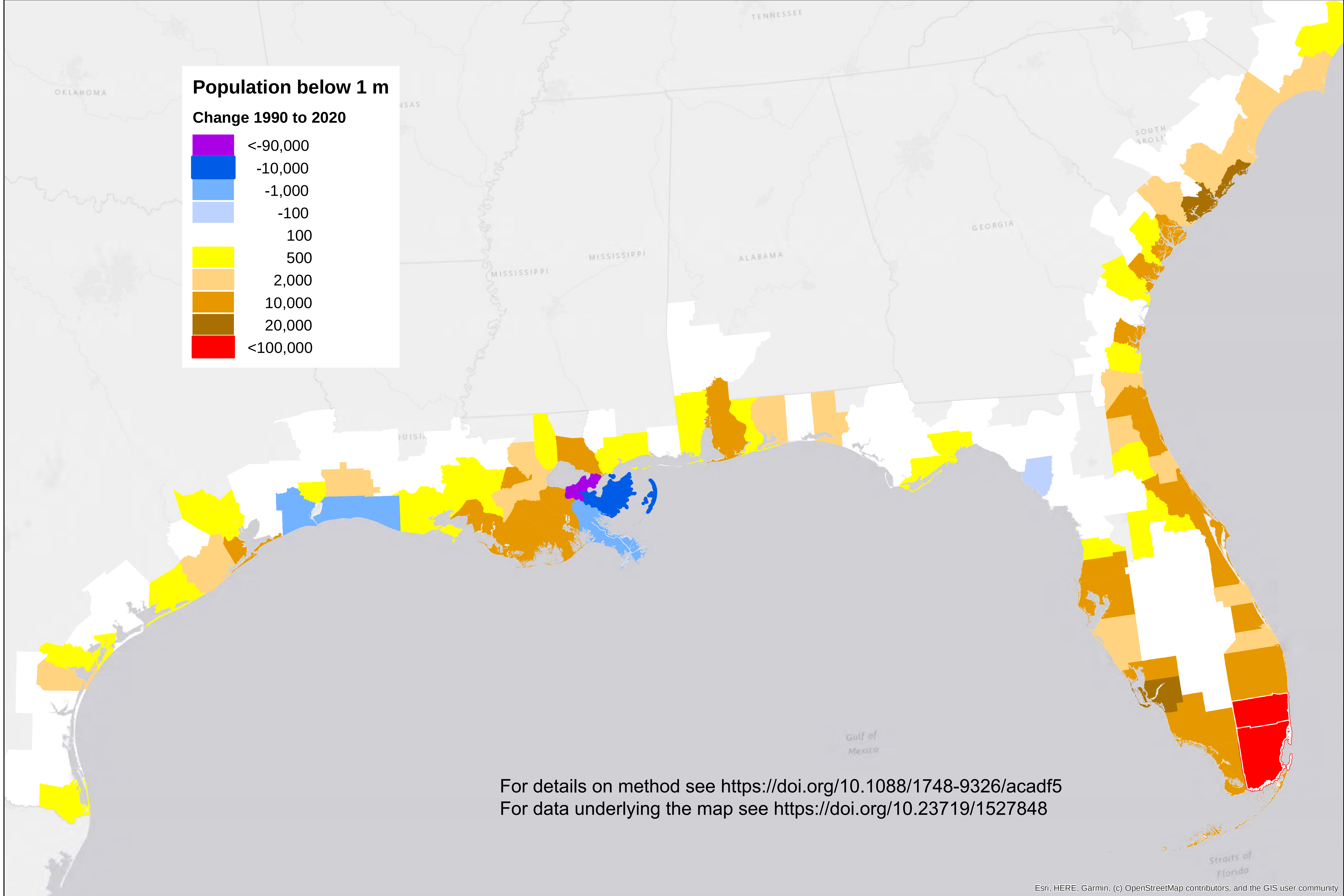
Population below 1 m
Change 1990 to 2020
<-90,000
-10,000
-1,000
-100
100
500
2,000
10,000
20,000
<100,000
OKLAHOMA
TENNESSEE
MISSISSIPPI
MISSISSIPPI
ALABAMA
GEORGIA
Gulf of Mexico
Straits of Florida
For details on method see https://doi.org/10.1088/1748-9326/acadf5
For data underlying the map see https://doi.org/10.23719/1527848
Esri, HERE, Garmin, (c) OpenStreetMap contributors, and the GIS user community

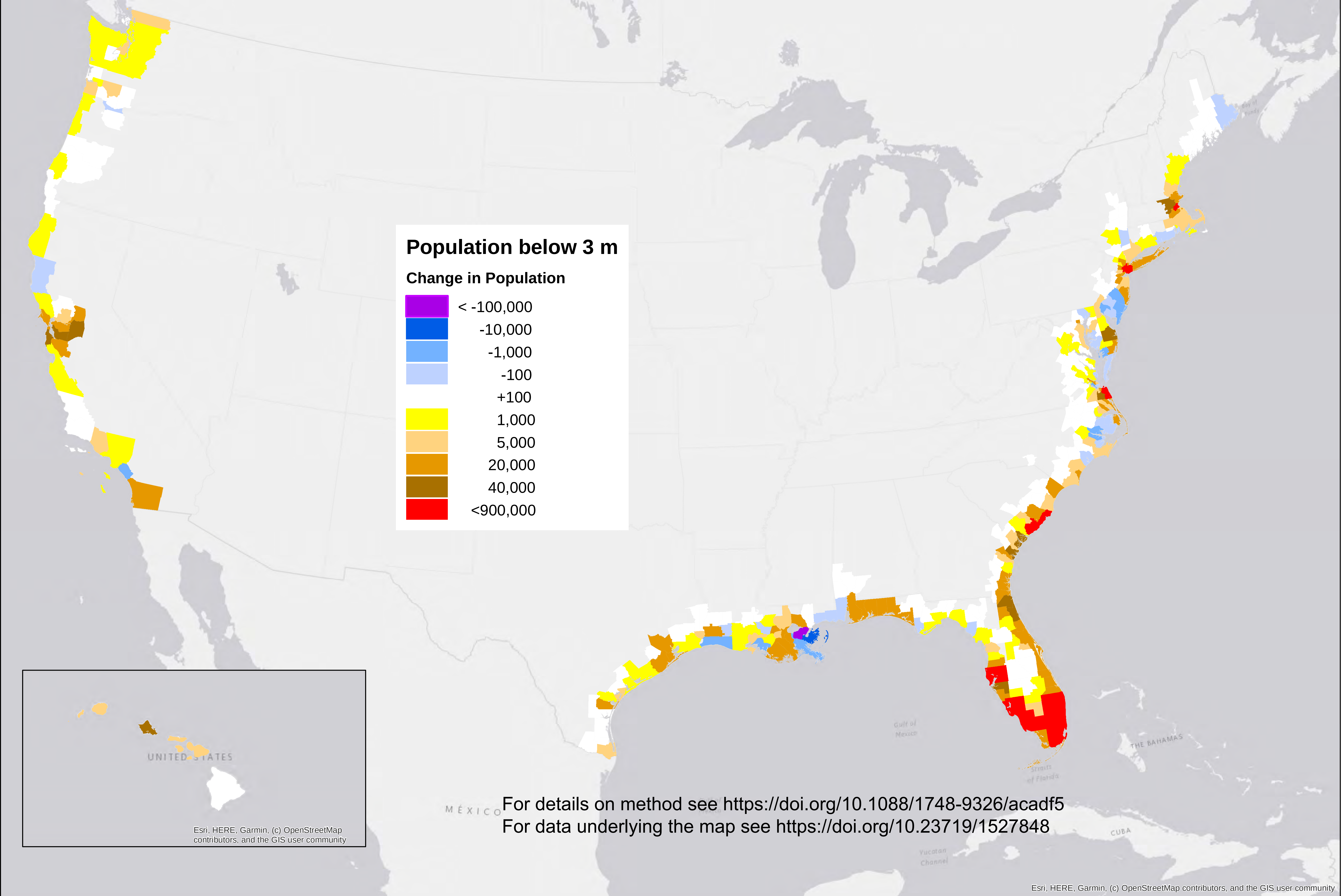
Population below 3 m
Change in Population
< -100,000
-10,000
-1,000
-100
+100
1,000
5,000
20,000
40,000
<900,000
UNITED STATES
MÉXICO
THE BAHAMAS
CUBA
Esri, HERE, Garmin, (c) OpenStreetMap contributors, and the GIS user community
For details on method see https://doi.org/10.1088/1748-9326/acadf5
For data underlying the map see https://doi.org/10.23719/1527848
Esri, HERE, Garmin, (c) OpenStreetMap contributors, and the GIS user community

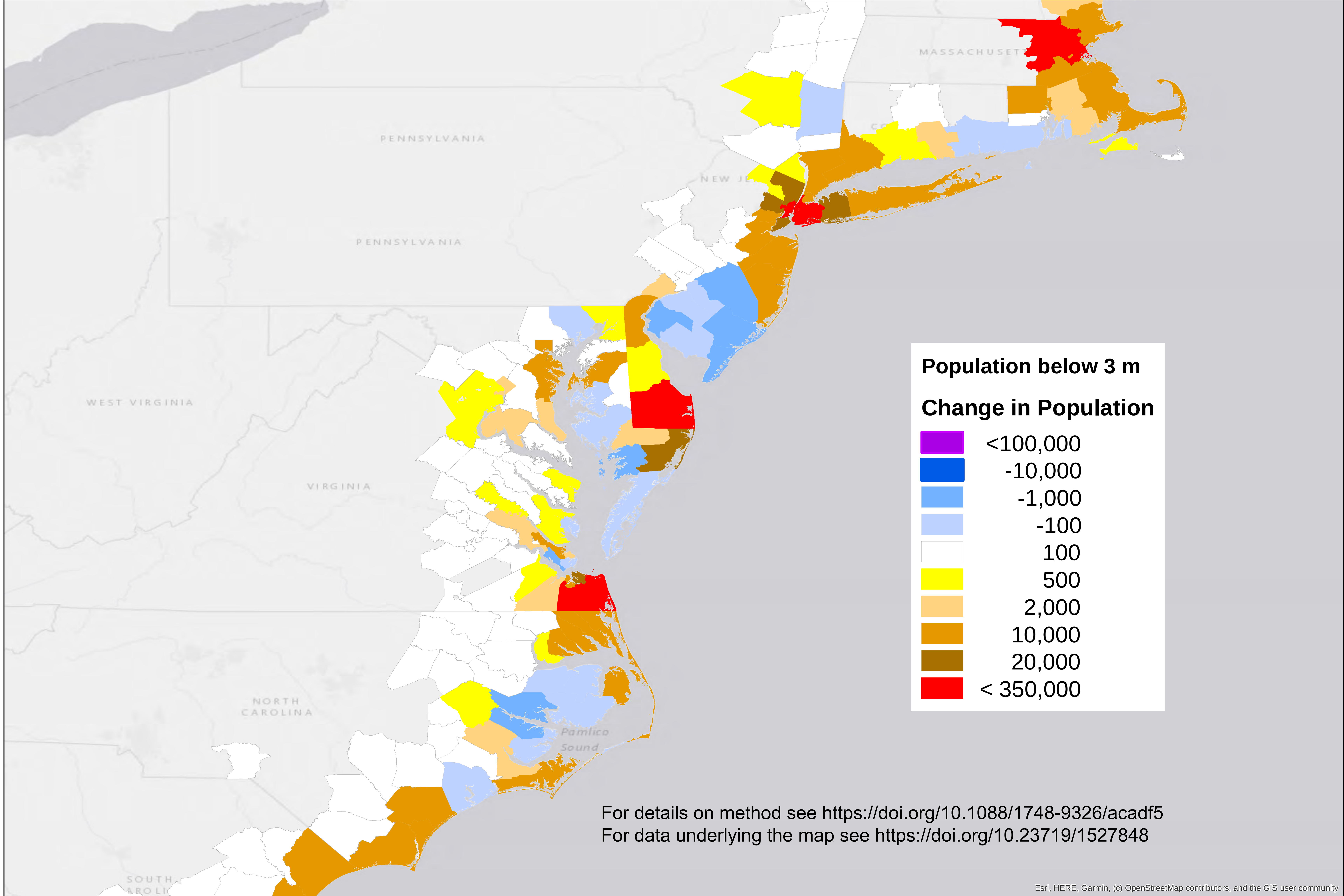
Population below 3 m
Change in Population
<100,000
-10,000
-1,000
-100
100
500
2,000
10,000
20,000
< 350,000
PENNSYLVANIA
PENNSYLVANIA
WEST VIRGINIA
VIRGINIA
NORTH CAROLINA
Pamlico Sound
For details on method see https://doi.org/10.1088/1748-9326/acadf5
For data underlying the map see https://doi.org/10.23719/1527848
Esri, HERE, Garmin, (c) OpenStreetMap contributors, and the GIS user community

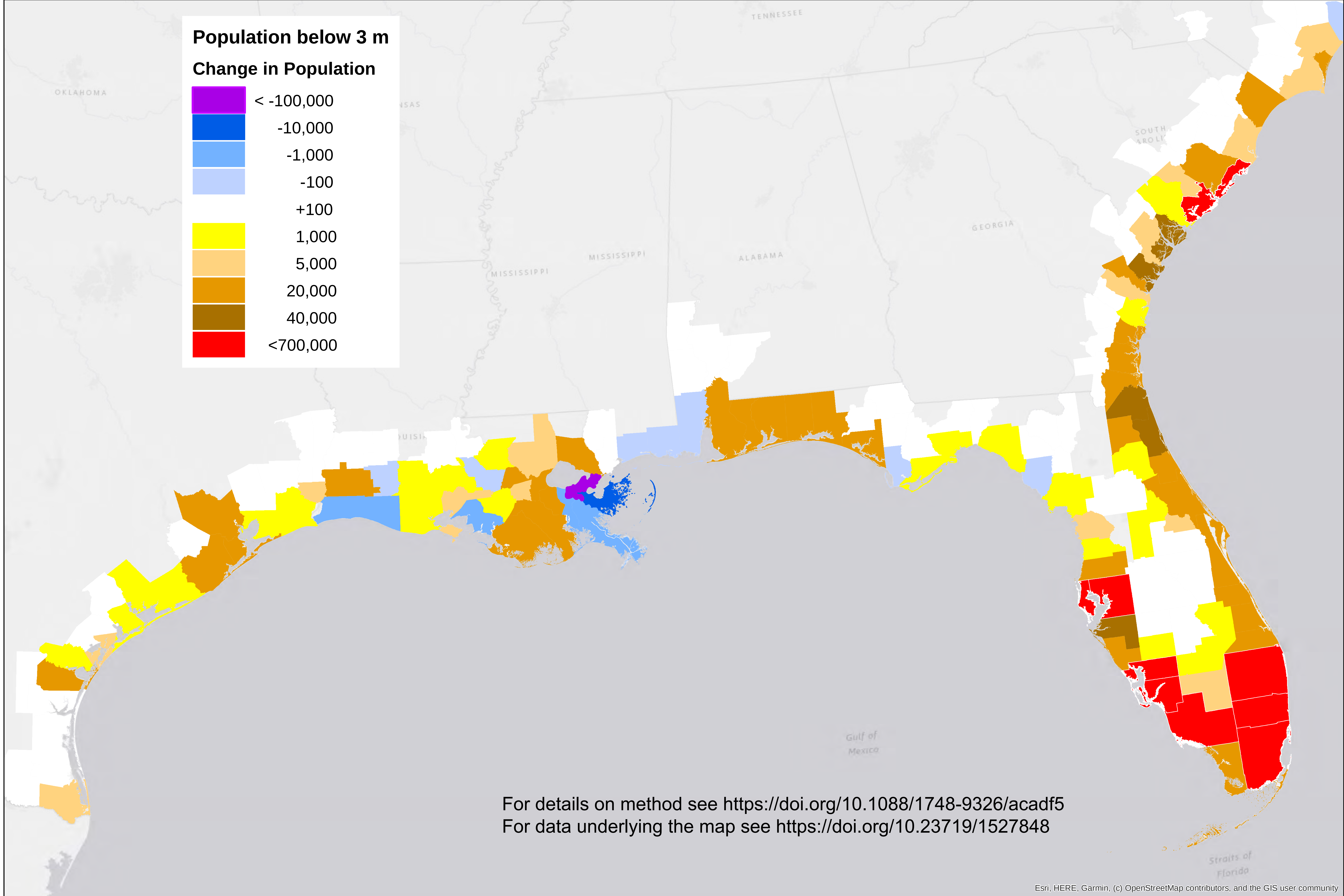

For details on method see https://doi.org/10.1088/1748-9326/acadf5
For data underlying the map see https://doi.org/10.23719/1527848

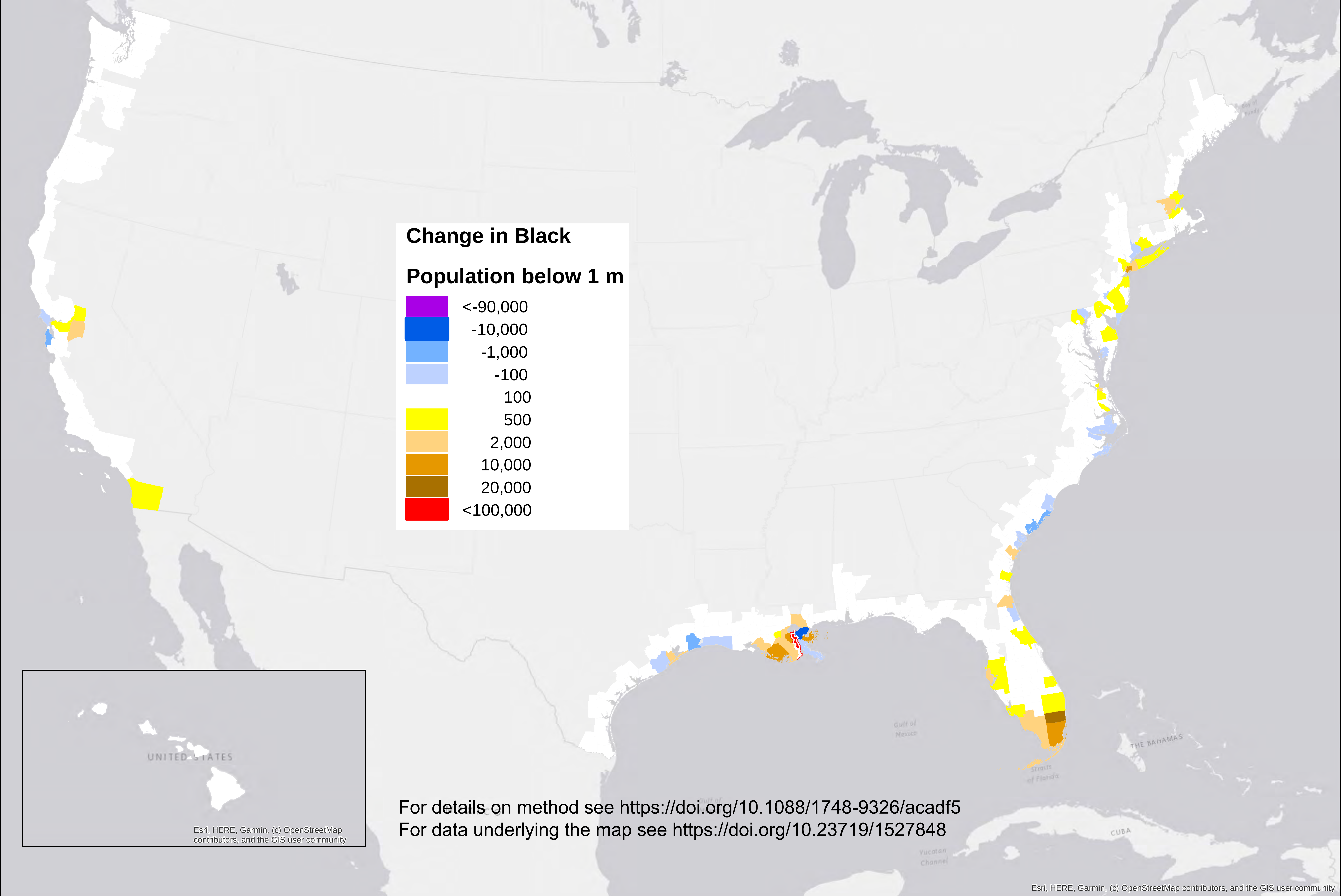
Change in Black
Population below 1 m
<-90,000
-10,000
-1,000
-100
100
500
2,000
10,000
20,000
<100,000
UNITED STATES
Esri, HERE, Garmin, (c) OpenStreetMap contributors, and the GIS user community
THE BAHAMAS
CUBA
For details on method see https://doi.org/10.1088/1748-9326/acadf5
For data underlying the map see https://doi.org/10.23719/1527848
Esri, HERE, Garmin, (c) OpenStreetMap contributors, and the GIS user community

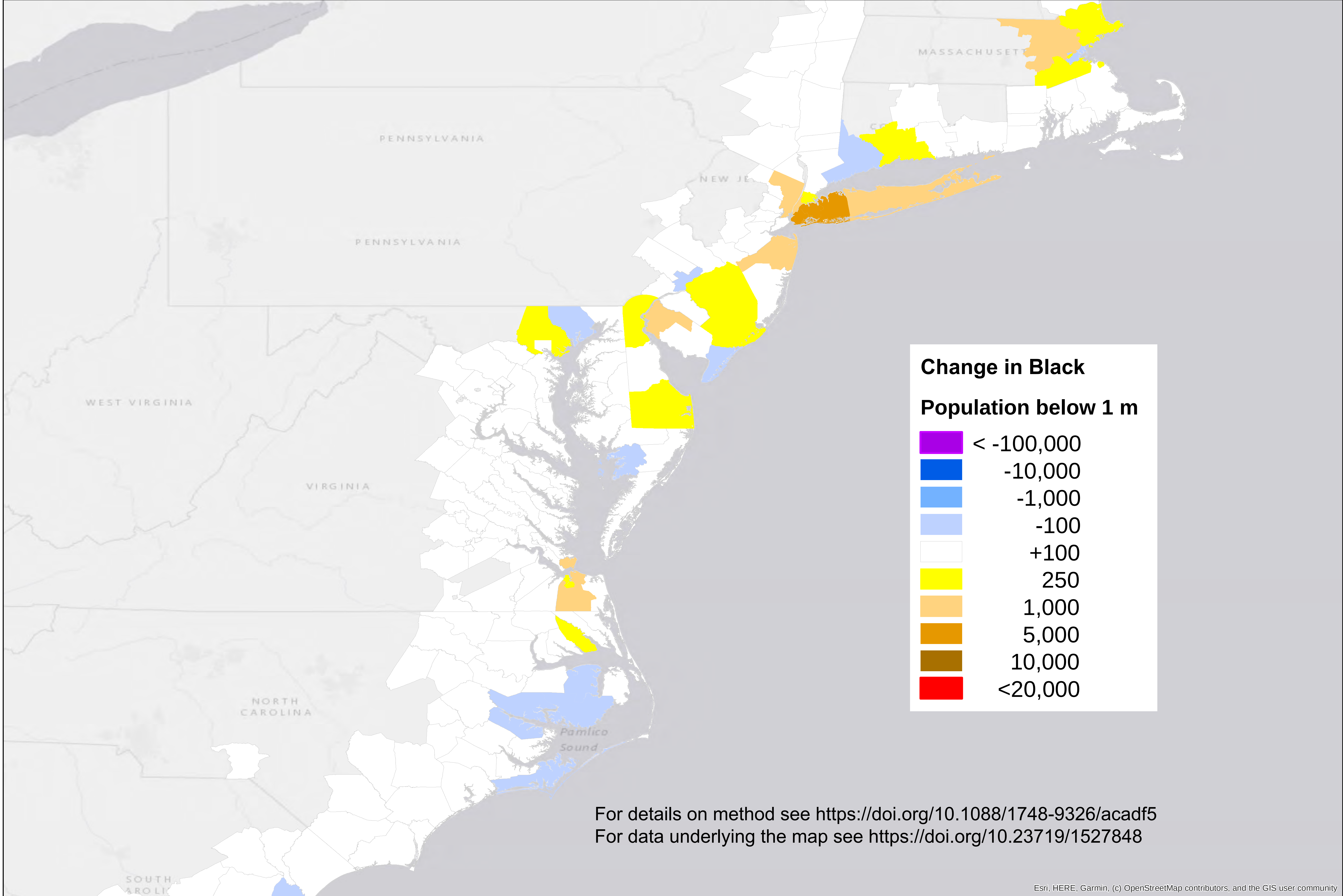
Change in Black
Population below 1 m
< -100,000
-10,000
-1,000
-100
+100
250
1,000
5,000
10,000
<20,000
PENNSYLVANIA
PENNSYLVANIA
NEW JE
MASSACHUSETT
WEST VIRGINIA
VIRGINIA
NORTH CAROLINA
Pamlico Sound
For details on method see https://doi.org/10.1088/1748-9326/acadf5
For data underlying the map see https://doi.org/10.23719/1527848
Esri, HERE, Garmin, (c) OpenStreetMap contributors, and the GIS user community

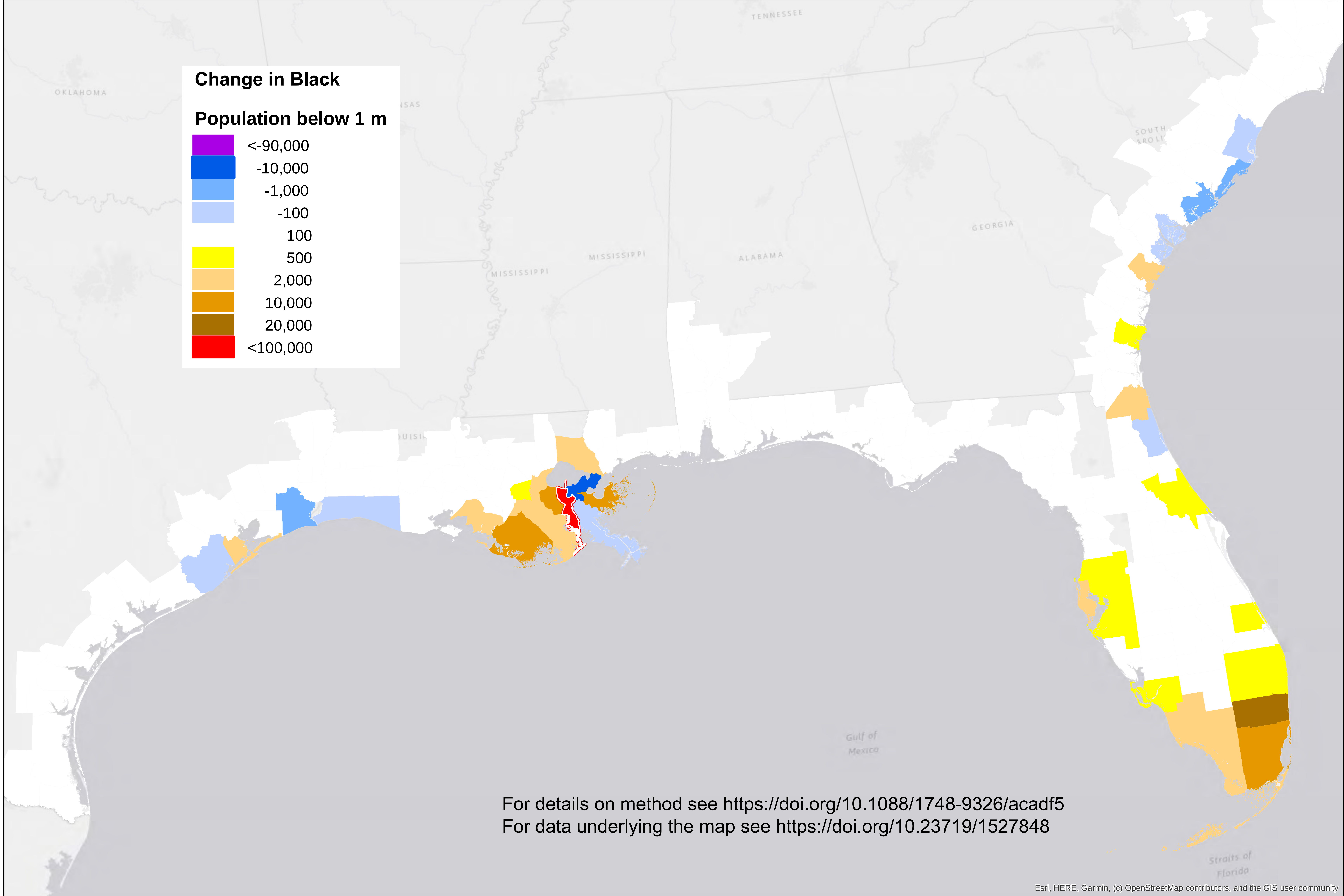
Change in Black
Population below 1 m
<-90,000
-10,000
-1,000
-100
100
500
2,000
10,000
20,000
<100,000
OKLAHOMA
TENNESSEE
MISSISSIPPI
MISSISSIPPI
ALABAMA
GEORGIA
Gulf of Mexico
Straits of Florida
For details on method see https://doi.org/10.1088/1748-9326/acadf5
For data underlying the map see https://doi.org/10.23719/1527848
Esri, HERE, Garmin, (c) OpenStreetMap contributors, and the GIS user community

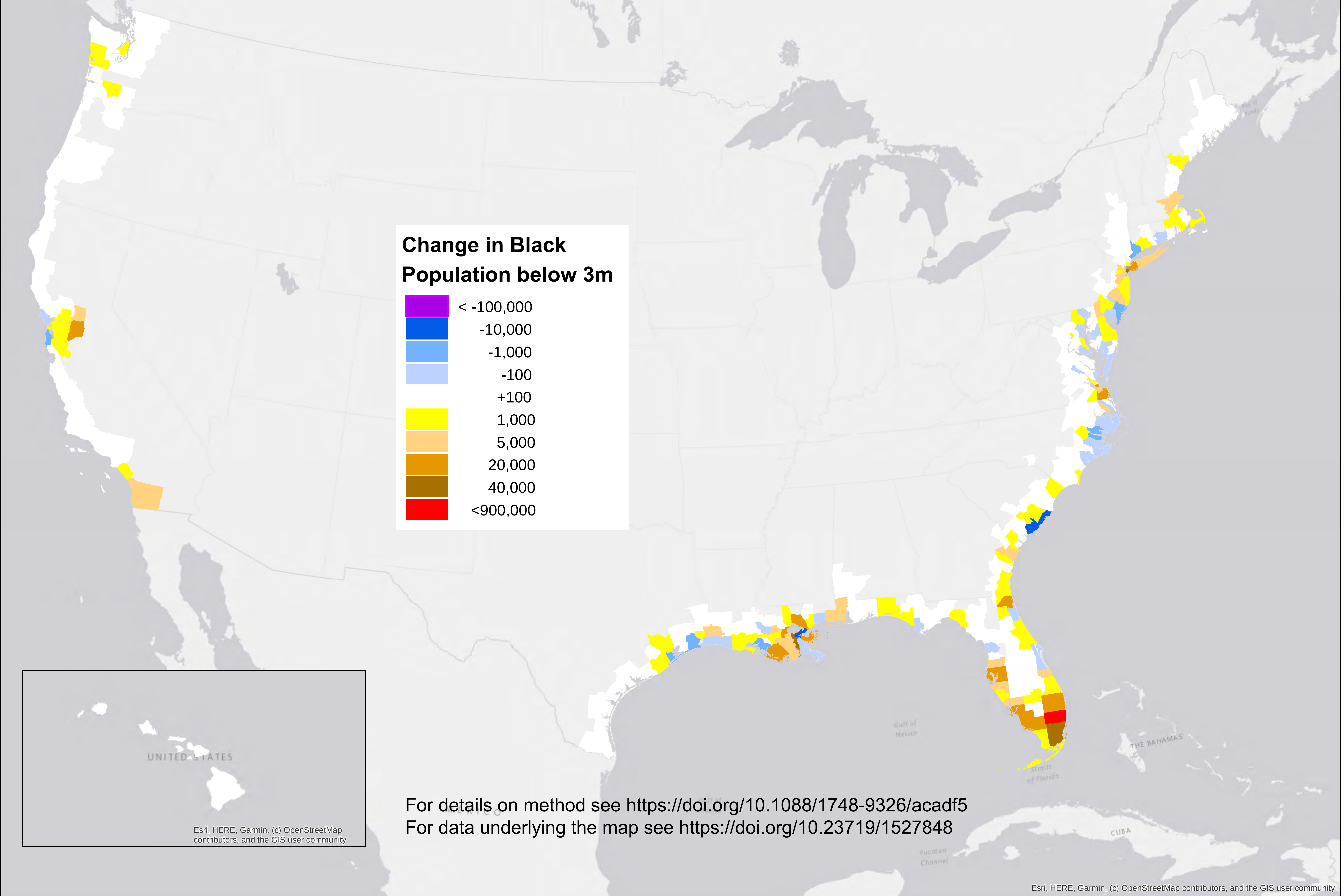
Change in Black
Population below 3m
< -100,000
-10,000
-1,000
-100
+100
1,000
5,000
20,000
40,000
<900,000
UNITED STATES
THE BAHAMAS
CUBA
Esri, HERE, Garmin, (c) OpenStreetMap contributors, and the GIS user community
For details on method see https://doi.org/10.1088/1748-9326/acadf5
For data underlying the map see https://doi.org/10.23719/1527848
Esri, HERE, Garmin, (c) OpenStreetMap contributors, and the GIS user community

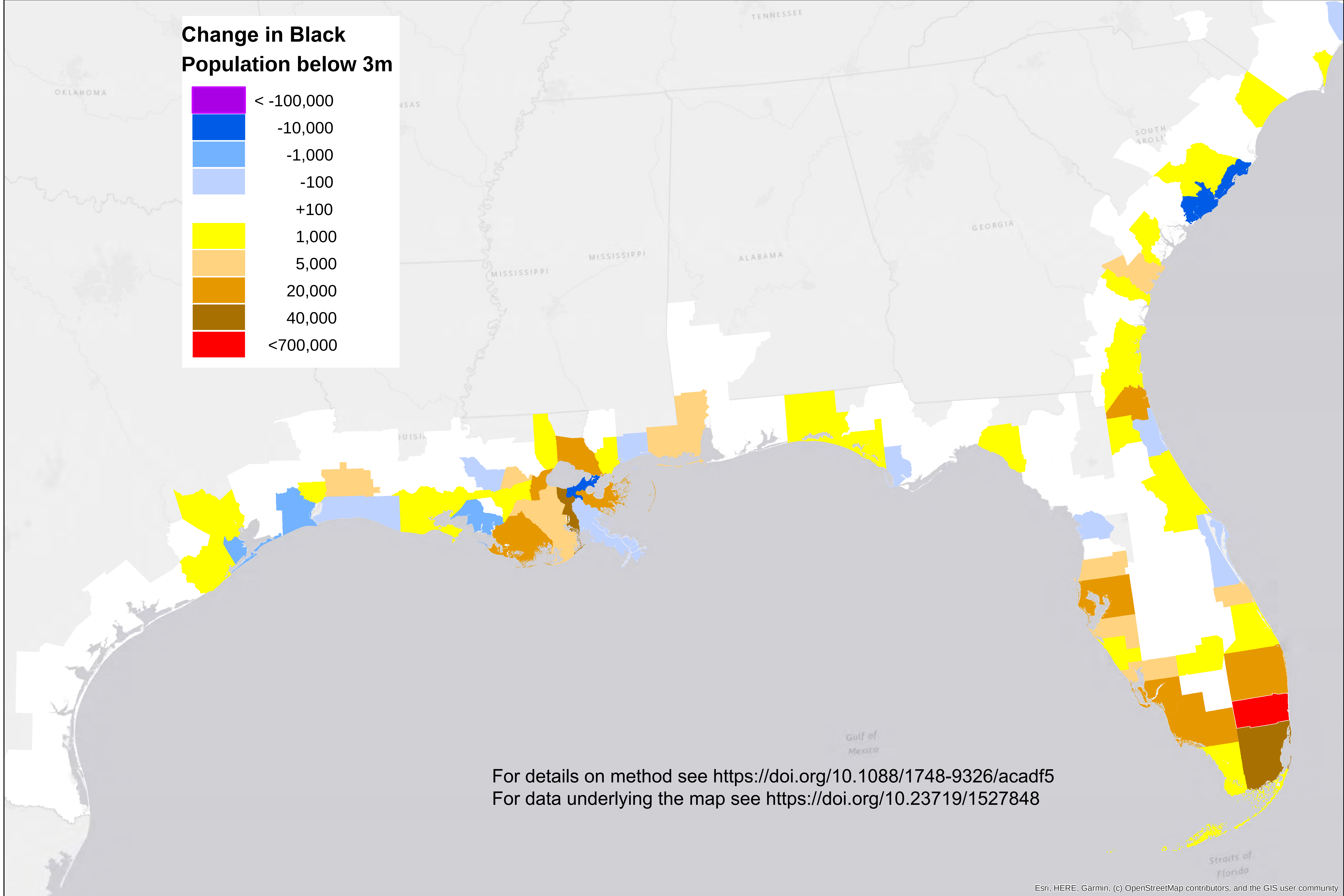
Change in Black Population below 3m
< -100,000
-10,000
-1,000
-100
+100
1,000
5,000
20,000
40,000
<700,000
OKLAHOMA
TENNESSEE
GEORGIA
ALABAMA
MISSISSIPPI
MISSISSIPPI
Gulf of Mexico
Straits of Florida
For details on method see https://doi.org/10.1088/1748-9326/acadf5
For data underlying the map see https://doi.org/10.23719/1527848
Esri, HERE, Garmin, (c) OpenStreetMap contributors, and the GIS user community

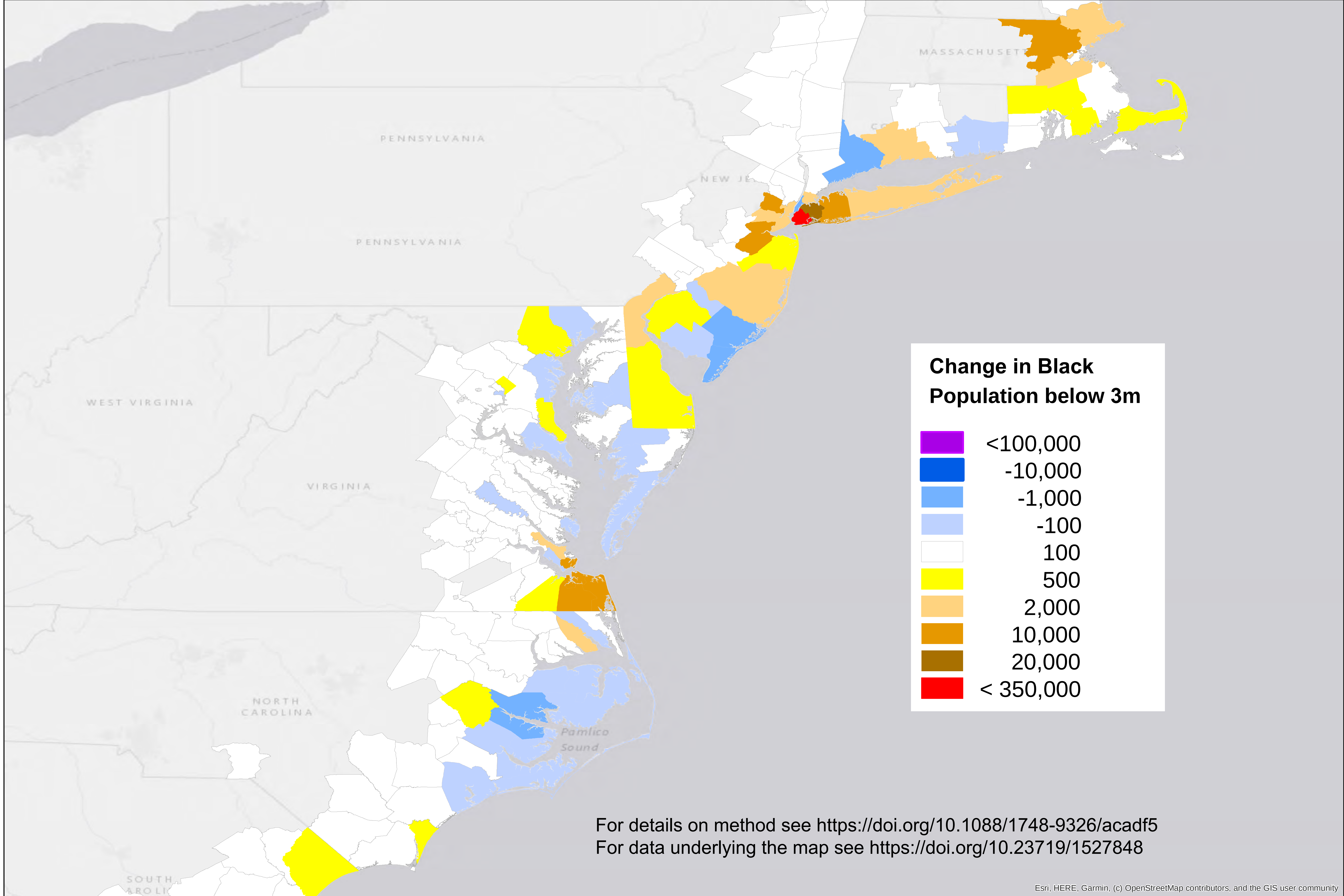
PENNSYLVANIA
PENNSYLVANIA
WEST VIRGINIA
VIRGINIA
NORTH CAROLINA
MASSACHUSETT
NEW JE
Pamlico Sound
Change in Black Population below 3m
<100,000
-10,000
-1,000
-100
100
500
2,000
10,000
20,000
< 350,000
For details on method see https://doi.org/10.1088/1748-9326/acadf5
For data underlying the map see https://doi.org/10.23719/1527848
Esri, HERE, Garmin, (c) OpenStreetMap contributors, and the GIS user community

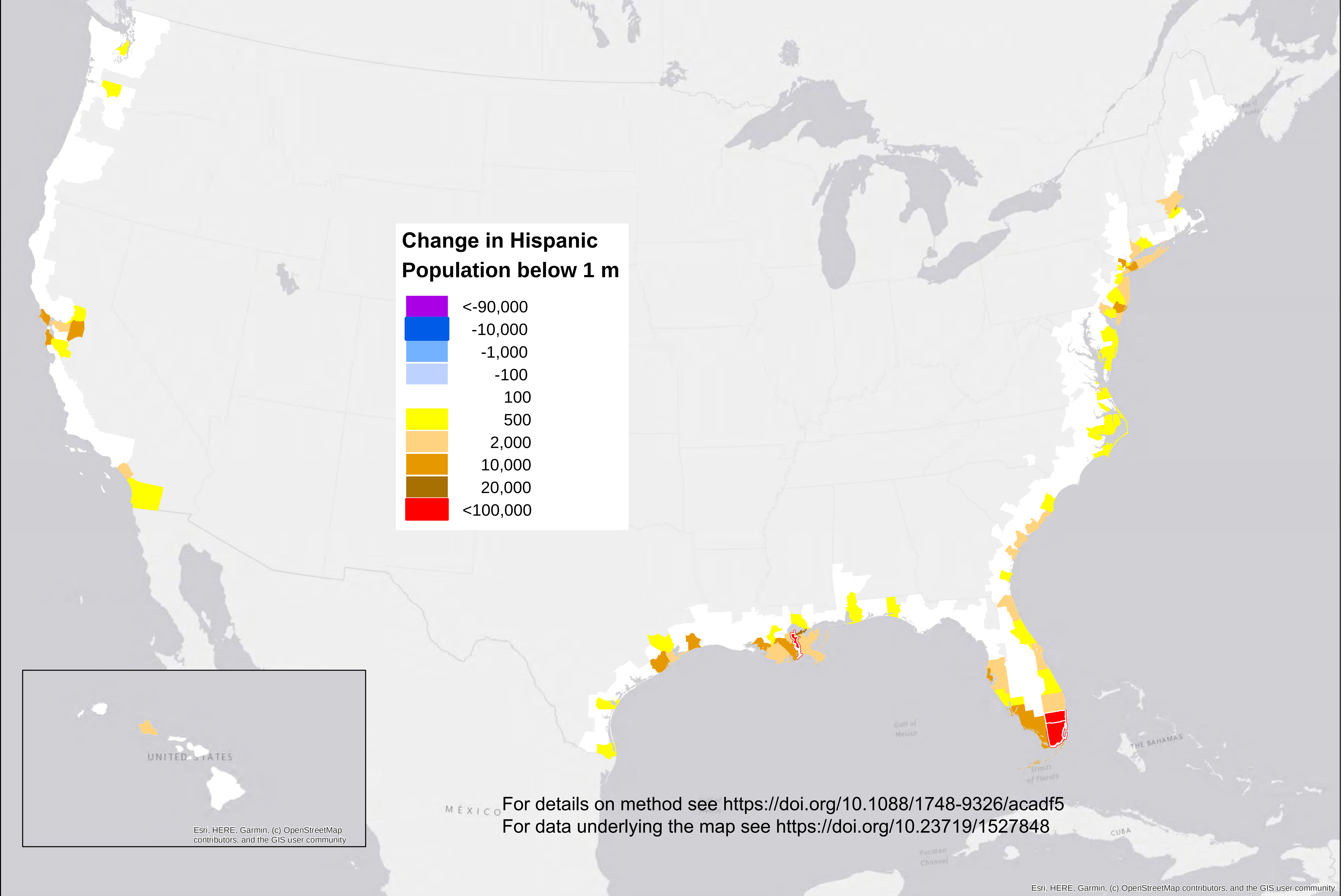

For details on method see https://doi.org/10.1088/1748-9326/acadf5
For data underlying the map see https://doi.org/10.23719/1527848

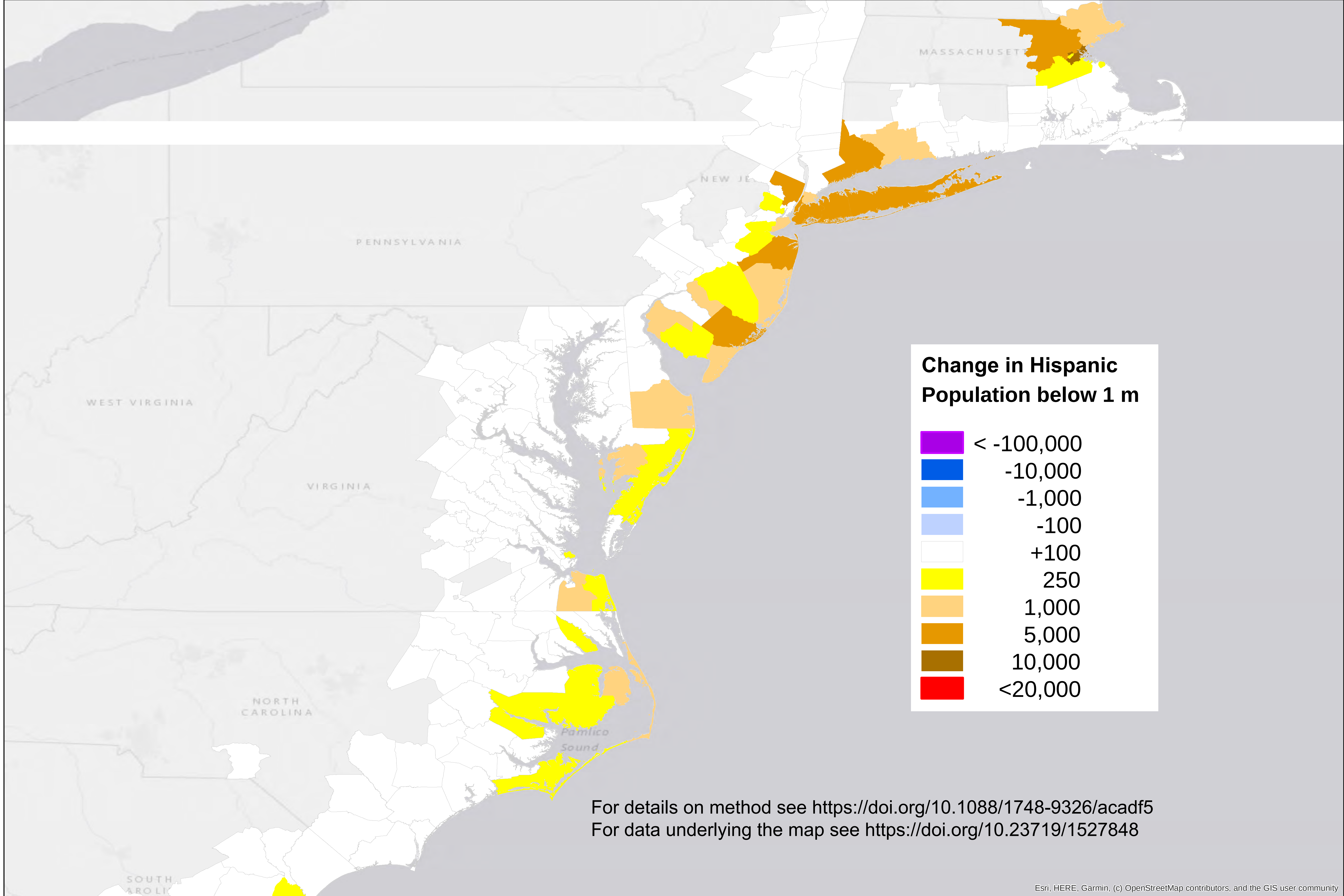
Change in Hispanic Population below 1 m
< -100,000
-10,000
-1,000
-100
+100
250
1,000
5,000
10,000
<20,000
MASSACHUSETT
NEW JE
PENNSYLVANIA
WEST VIRGINIA
VIRGINIA
NORTH CAROLINA
Pamlico Sound
SOUTH
For details on method see https://doi.org/10.1088/1748-9326/acadf5
For data underlying the map see https://doi.org/10.23719/1527848
Esri, HERE, Garmin, (c) OpenStreetMap contributors, and the GIS user community

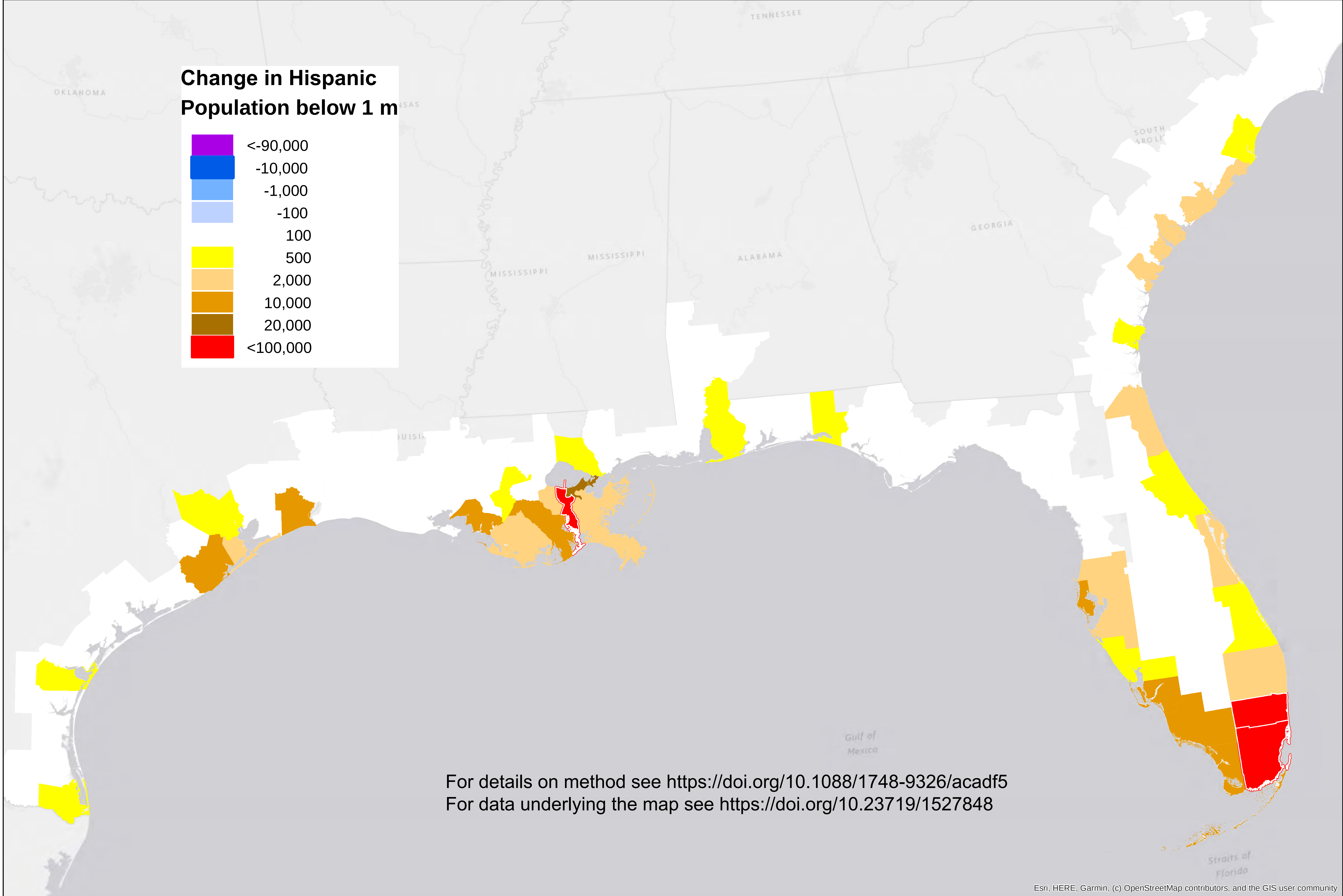
Change in Hispanic
Population below 1 m
<-90,000
-10,000
-1,000
-100
100
500
2,000
10,000
20,000
<100,000
OKLAHOMA
TENNESSEE
MISSISSIPPI
MISSISSIPPI
ALABAMA
GEORGIA
Gulf of
Mexico
Straits of
Florida
For details on method see https://doi.org/10.1088/1748-9326/acadf5
For data underlying the map see https://doi.org/10.23719/1527848
Esri, HERE, Garmin, (c) OpenStreetMap contributors, and the GIS user community

Apparent Migration into and out of Land Vulnerable to Sea Level Rise by County 1990 to 2020

These maps show county-specific estimates of the change in populations less than one meter above the sea level of 2020, as estimated in the paper for which this document provides supplemental maps. The population changes in these maps reflect only the net change from migration, births, and deaths from the land below one meter, and do not include the homes that were more than one meter above sea level in 1990 but less than one meter above sea level by 2020. For further details, see the main paper and the Supplemental Methods associated with this analysis.

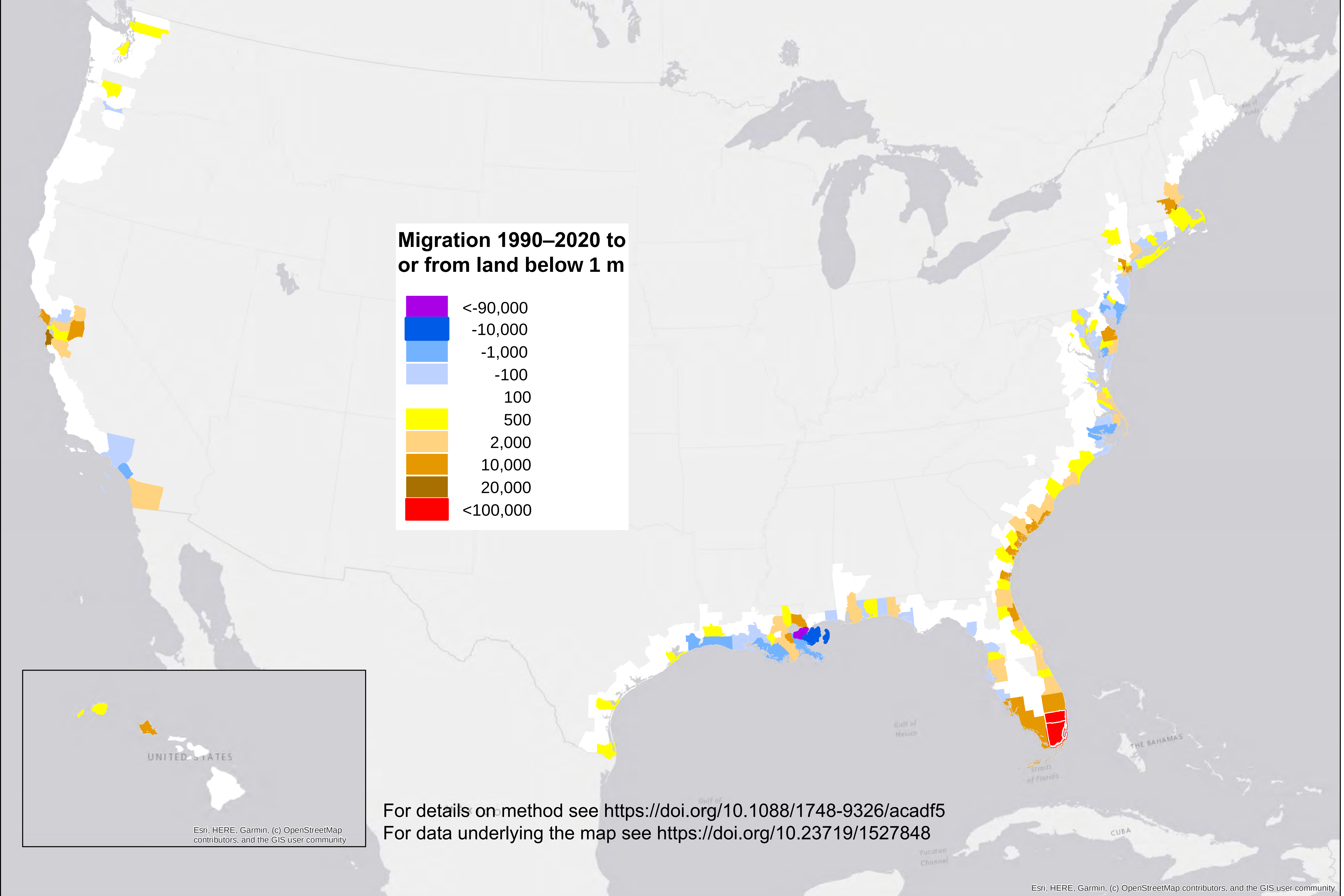
Migration 1990–2020 to or from land below 1 m
<-90,000
-10,000
-1,000
-100
100
500
2,000
10,000
20,000
<100,000
UNITED STATES
THE BAHAMAS
CUBA
For details on method see https://doi.org/10.1088/1748-9326/acadf5
For data underlying the map see https://doi.org/10.23719/1527848
Esri, HERE, Garmin, (c) OpenStreetMap contributors, and the GIS user community
Esri, HERE, Garmin, (c) OpenStreetMap contributors, and the GIS user community

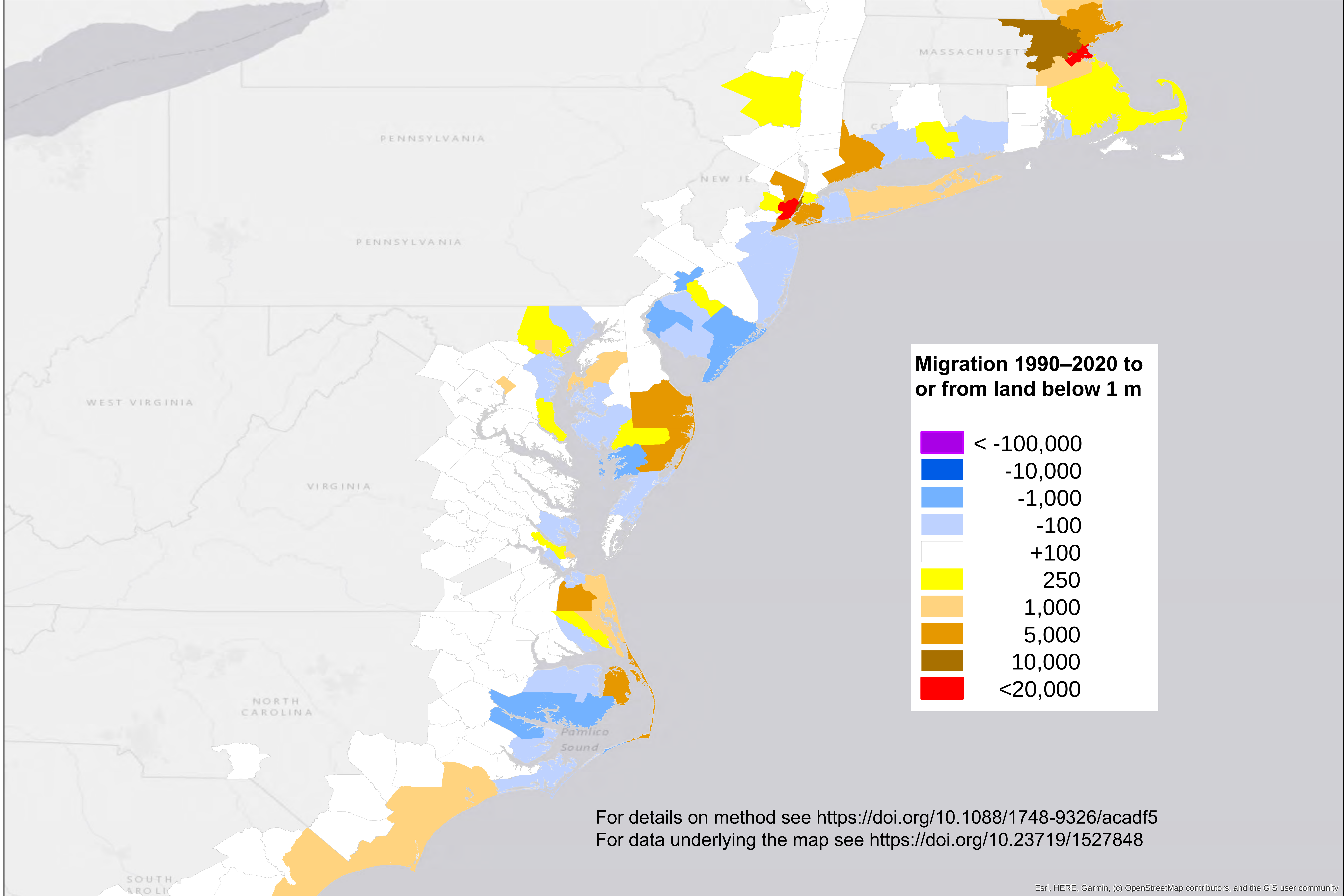
MASSACHUSETT
PENNSYLVANIA
PENNSYLVANIA
NEW JE
WEST VIRGINIA
VIRGINIA
NORTH
CAROLINA
Pamlico
Sound
SOUTH
AROLI
Migration 1990–2020 to
or from land below 1 m
< -100,000
-10,000
-1,000
-100
+100
250
1,000
5,000
10,000
<20,000
For details on method see https://doi.org/10.1088/1748-9326/acadf5
For data underlying the map see https://doi.org/10.23719/1527848
Esri, HERE, Garmin, (c) OpenStreetMap contributors, and the GIS user community

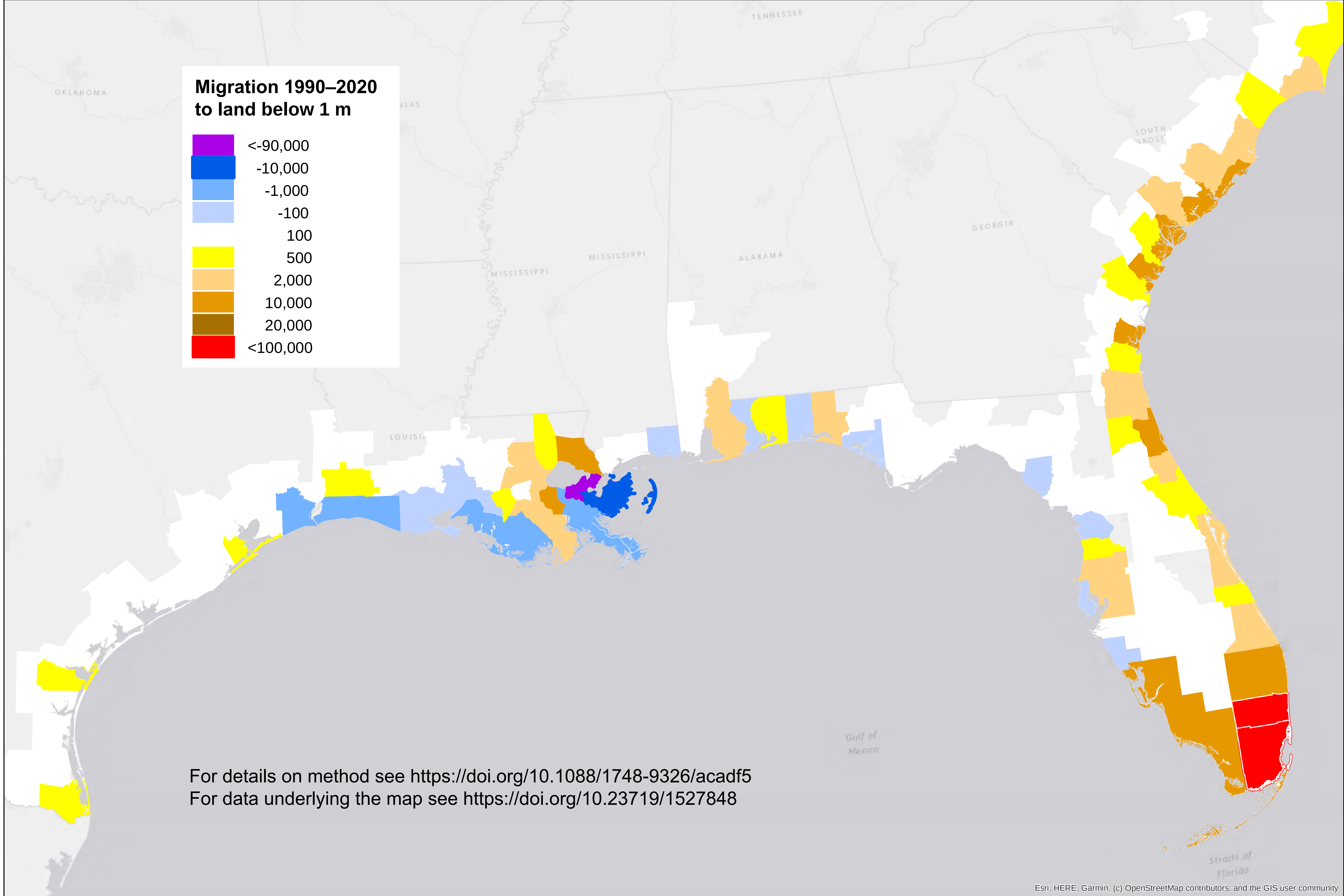
Migration 1990–2020
to land below 1 m
<-90,000
-10,000
-1,000
-100
100
500
2,000
10,000
20,000
<100,000
OKLAHOMA
TENNESSEE
MISSISSIPPI
MISSISSIPPI
ALABAMA
GEORGIA
Gulf of
Mexico
Straits of
Florida
For details on method see https://doi.org/10.1088/1748-9326/acadf5
For data underlying the map see https://doi.org/10.23719/1527848
Esri, HERE, Garmin, (c) OpenStreetMap contributors, and the GIS user community

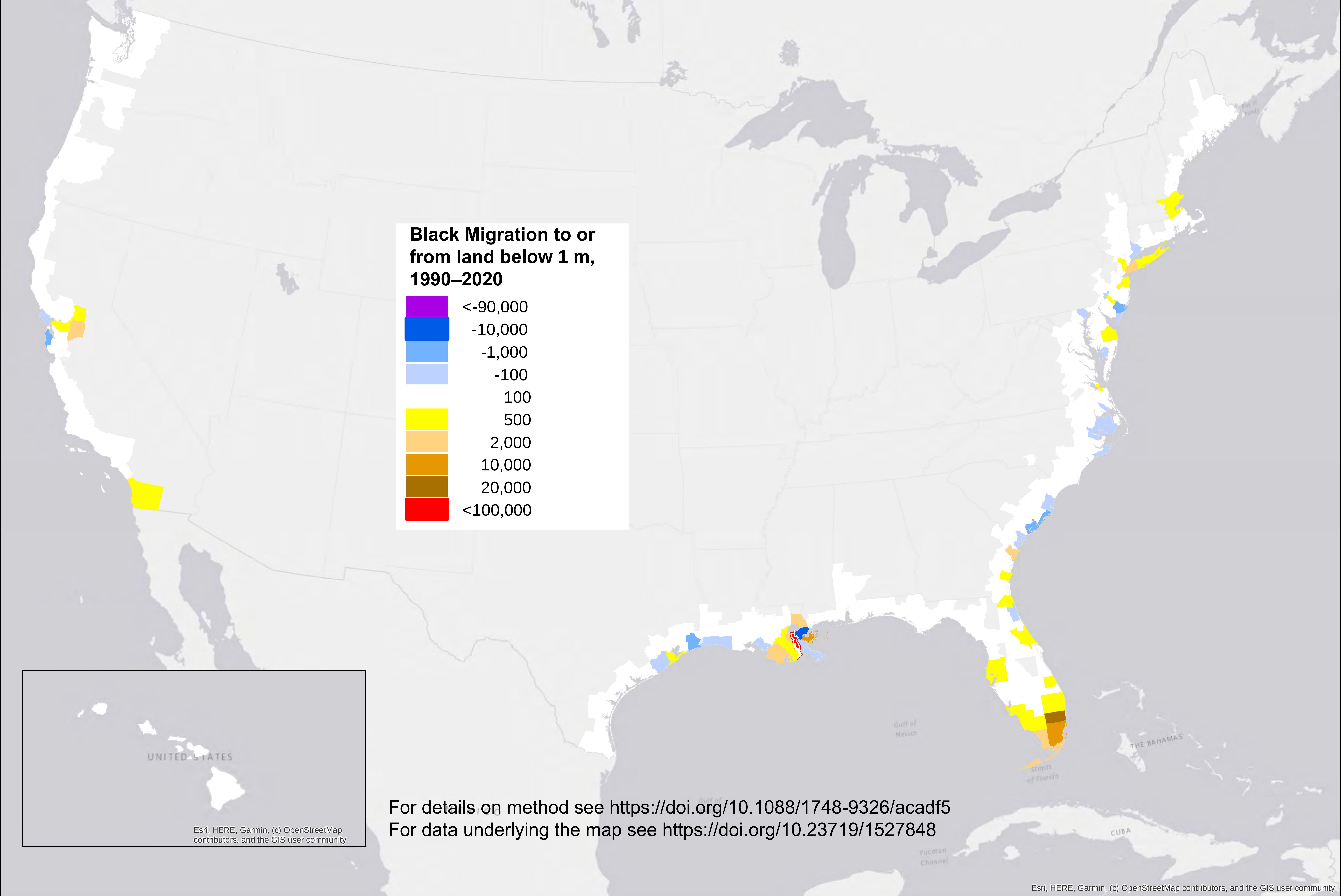

For details on method see https://doi.org/10.1088/1748-9326/acadf5
For data underlying the map see https://doi.org/10.23719/1527848

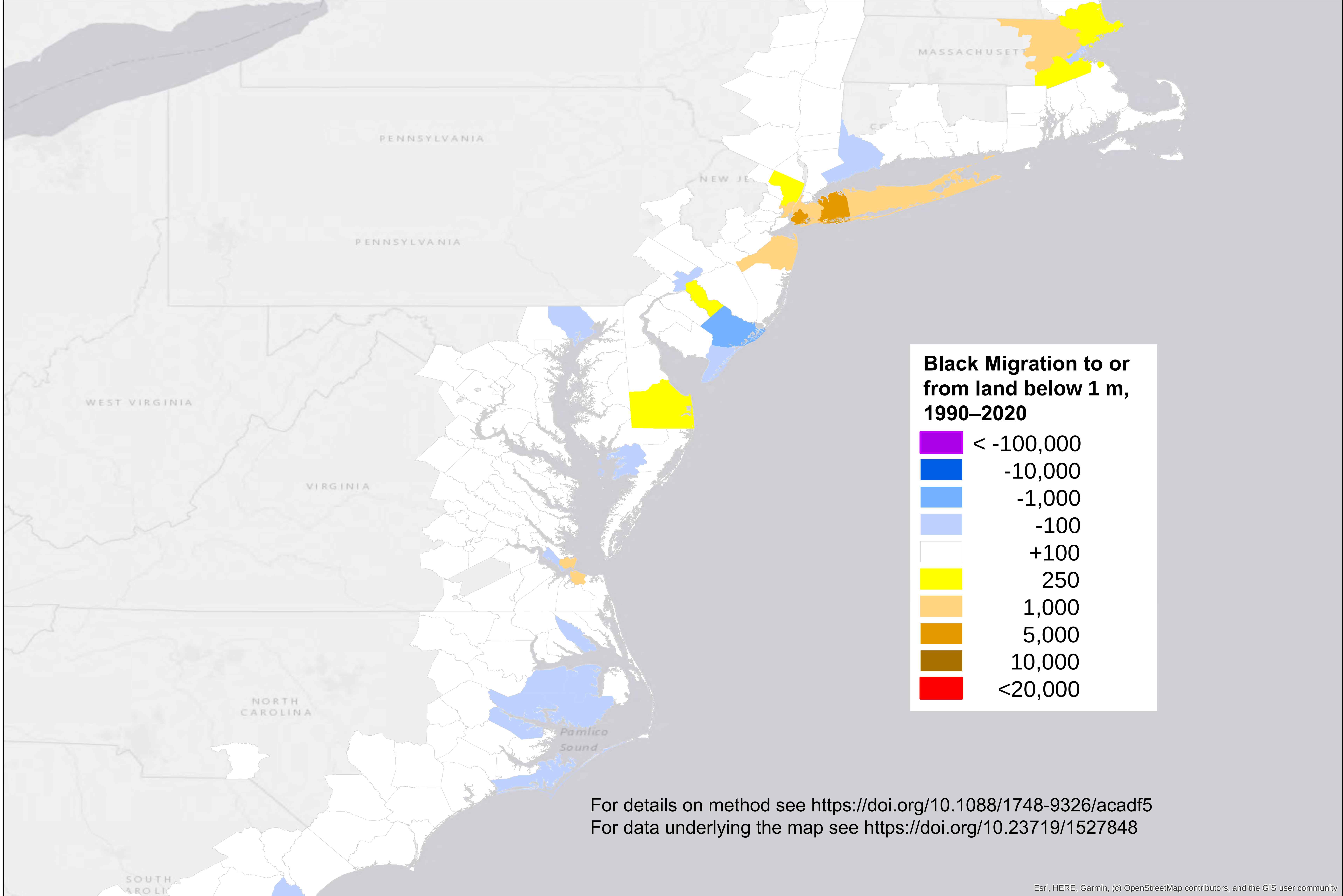
PENNSYLVANIA
PENNSYLVANIA
WEST VIRGINIA
VIRGINIA
NORTH CAROLINA
Pamlico Sound
Black Migration to or from land below 1 m, 1990–2020
< -100,000
-10,000
-1,000
-100
+100
250
1,000
5,000
10,000
<20,000
For details on method see https://doi.org/10.1088/1748-9326/acadf5
For data underlying the map see https://doi.org/10.23719/1527848
Esri, HERE, Garmin, (c) OpenStreetMap contributors, and the GIS user community

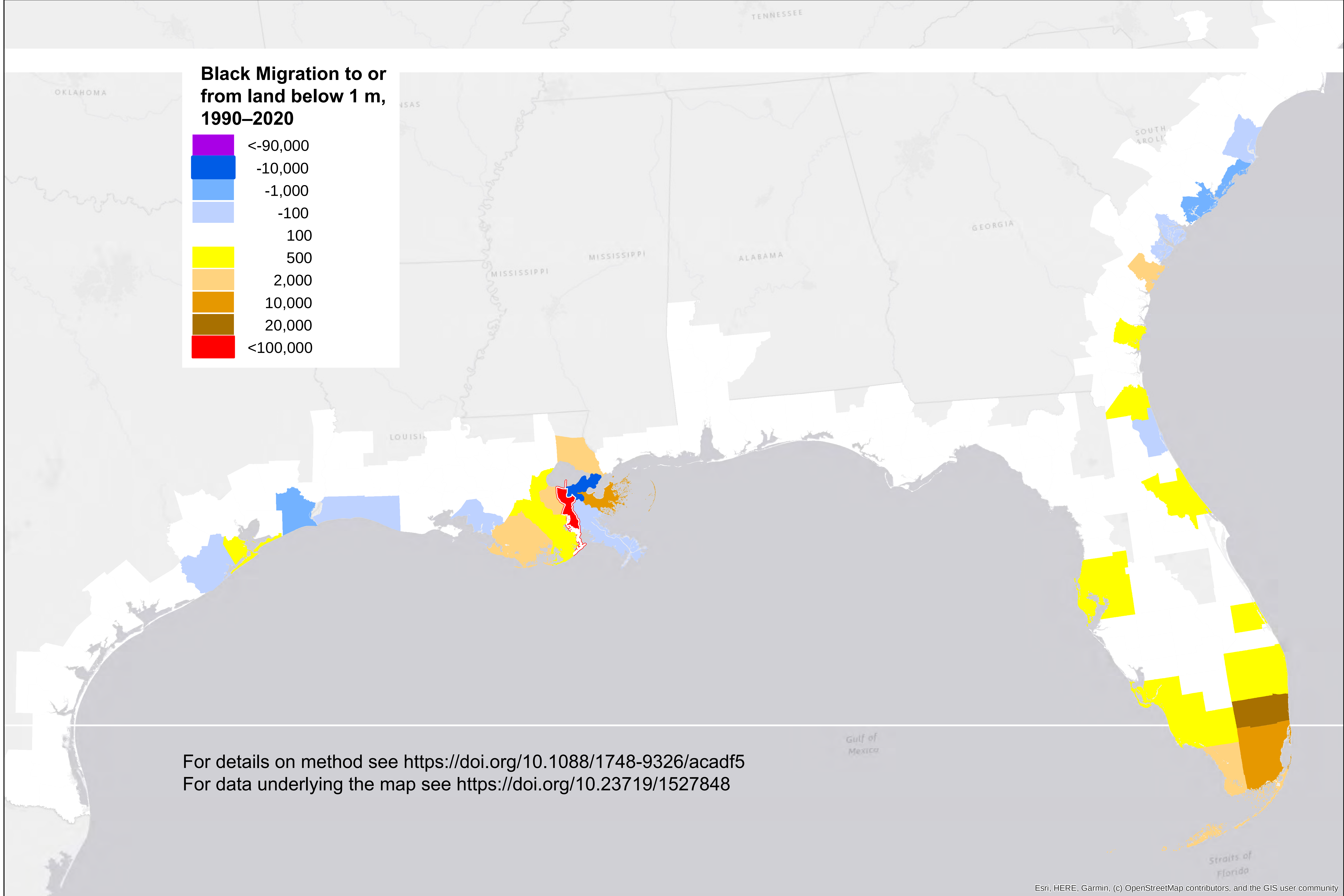
Black Migration to or from land below 1 m, 1990–2020
<-90,000
-10,000
-1,000
-100
100
500
2,000
10,000
20,000
<100,000
OKLAHOMA
TENNESSEE
SOUTH CAROLI
MISSISSIPPI
MISSISSIPPI
ALABAMA
GEORGIA
LOUISI
Gulf of Mexico
Straits of Florida
For details on method see https://doi.org/10.1088/1748-9326/acadf5
For data underlying the map see https://doi.org/10.23719/1527848
Esri, HERE, Garmin, (c) OpenStreetMap contributors, and the GIS user community

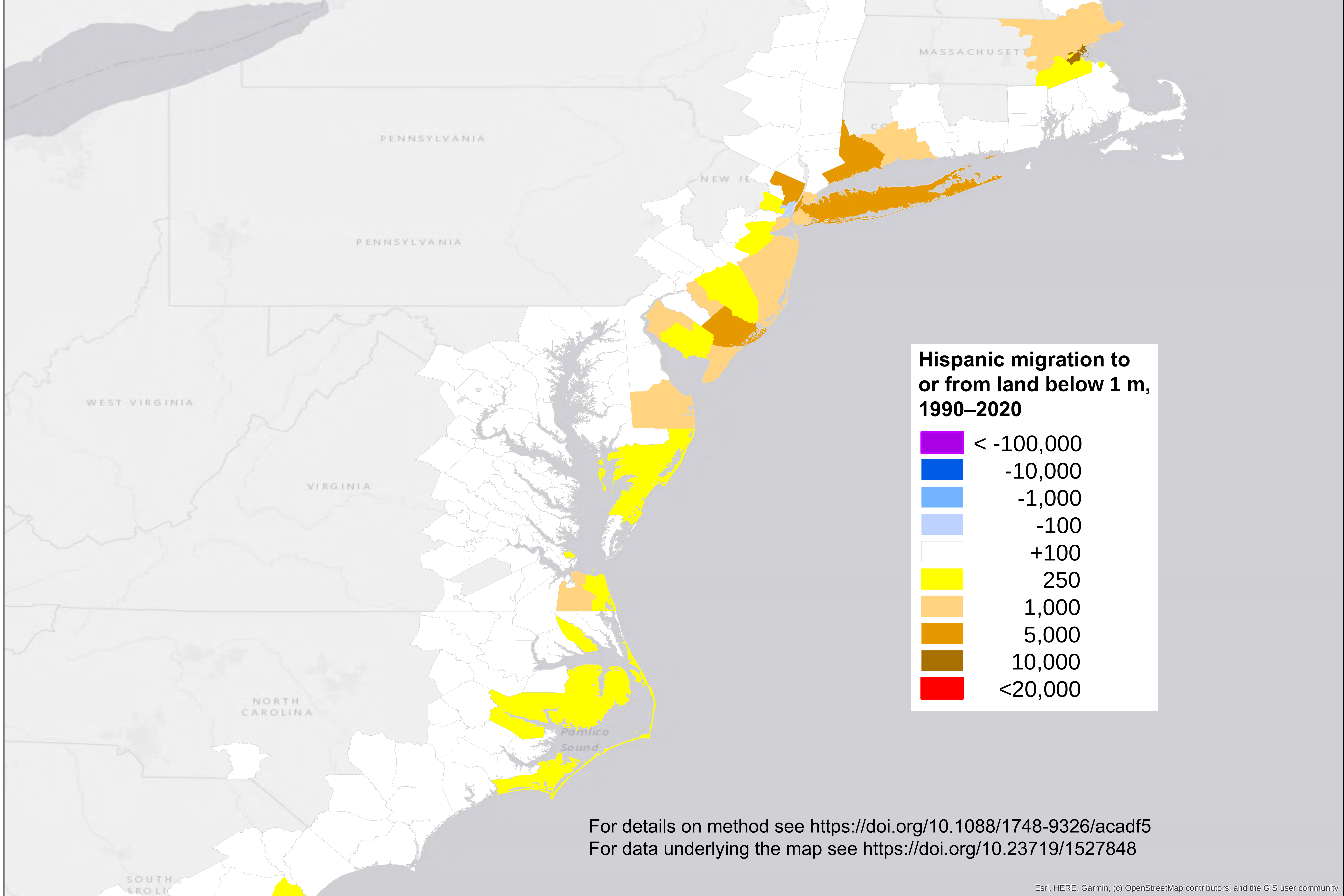

For details on method see https://doi.org/10.1088/1748-9326/acadf5
For data underlying the map see https://doi.org/10.23719/1527848

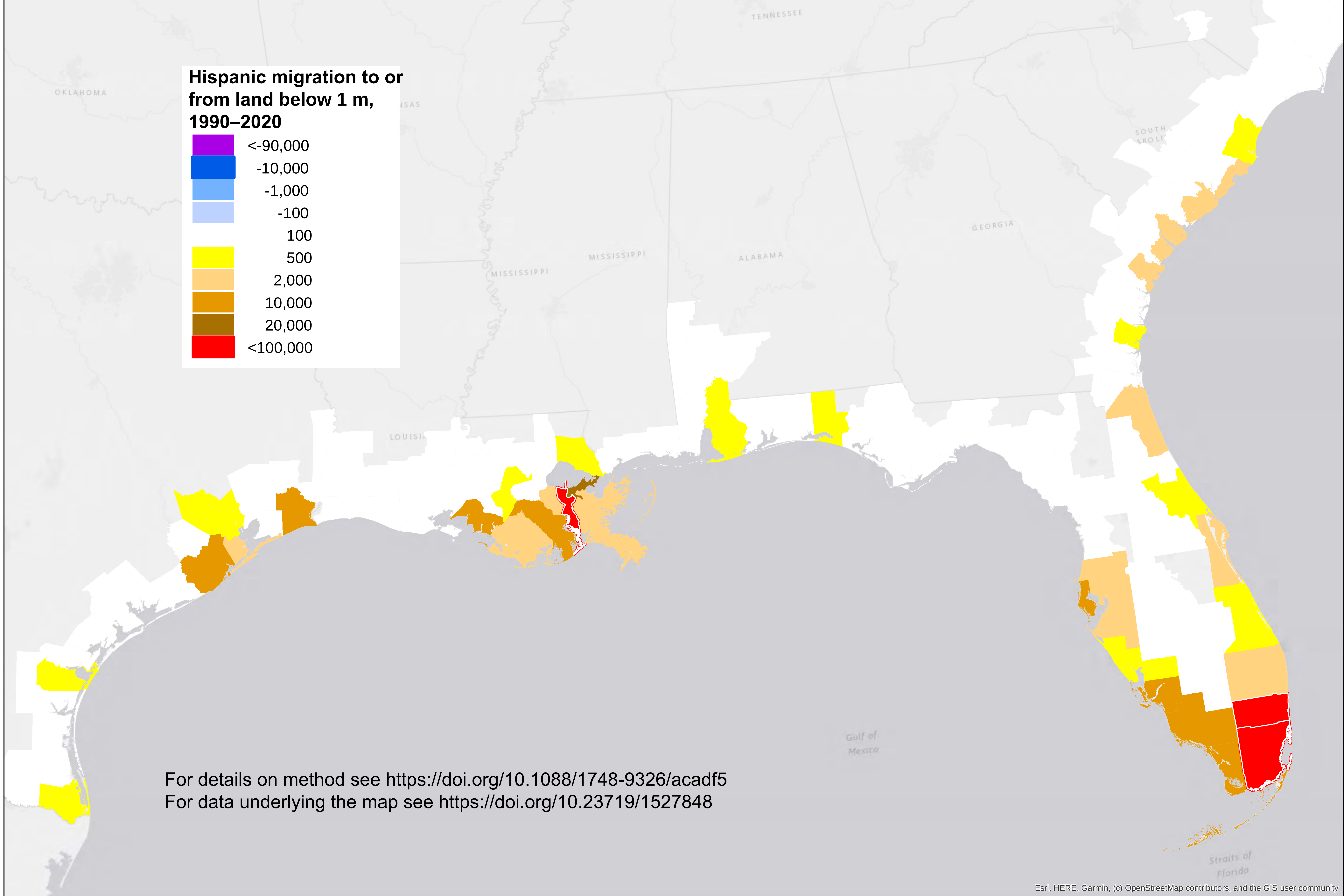

For details on method see https://doi.org/10.1088/1748-9326/acadf5
For data underlying the map see https://doi.org/10.23719/1527848

Change in Population Below One Meter by County Caused Solely By Sea Level Rise

1990 to 2020

These maps show county-specific estimates of the change in populations within one meter above sea level due solely to the effect of sea level rise, as estimated in the paper for which this document provides supplemental maps. The population changes in these maps area all based on the 2020 Census and thus do not reflect the changes from migration, births, and deaths; they only include the population of homes that were less than one meter above sea level by 2020, but are above the level the sea had reached by 1990. For further details, see the main paper and the Supplemental Methods associated with this analysis.

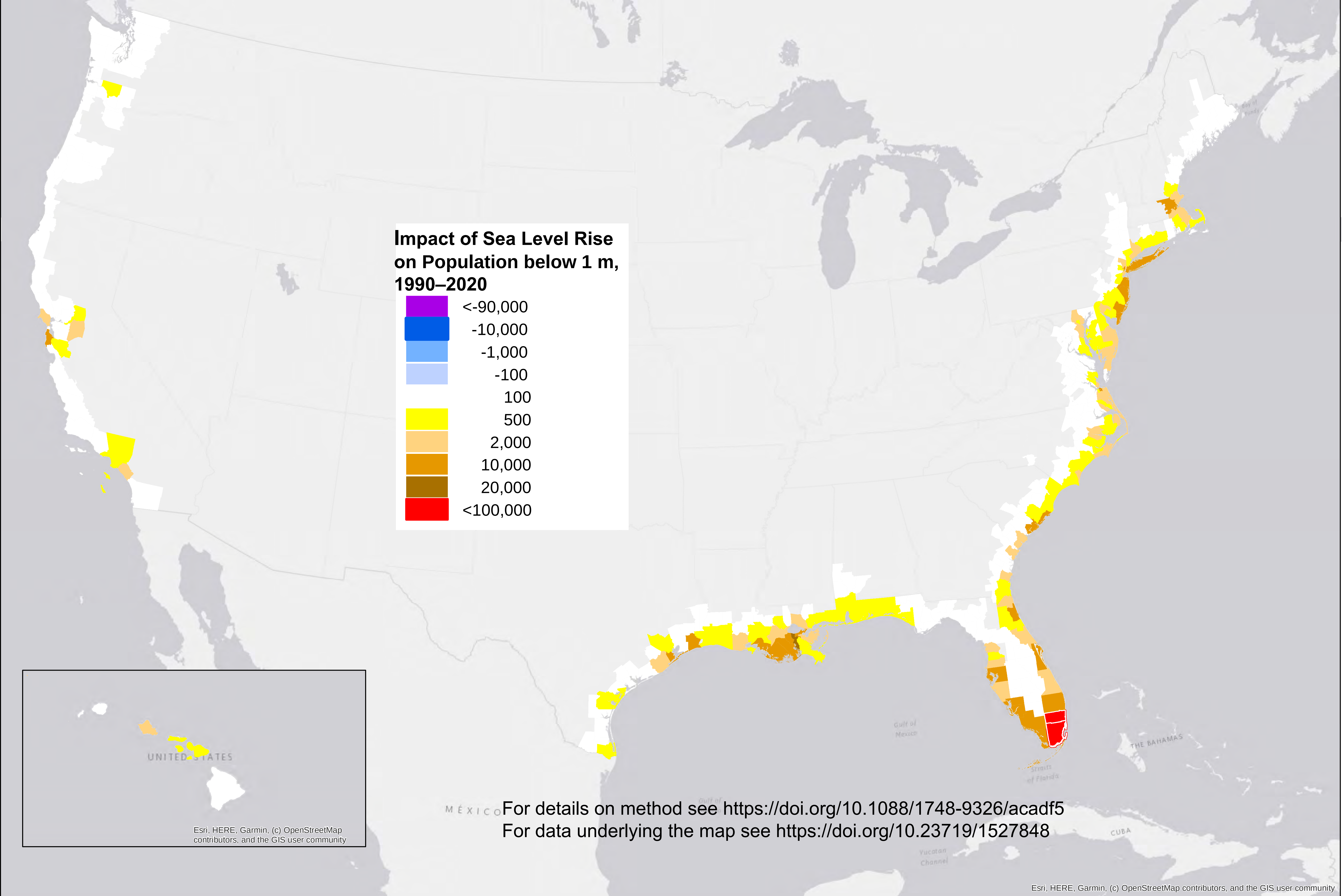
Impact of Sea Level Rise on Population below 1 m, 1990–2020
<-90,000
-10,000
-1,000
-100
100
500
2,000
10,000
20,000
<100,000
UNITED STATES
Esri, HERE, Garmin, (c) OpenStreetMap contributors, and the GIS user community
THE BAHAMAS
MÉXICO
CUBA
For details on method see https://doi.org/10.1088/1748-9326/acadf5
For data underlying the map see https://doi.org/10.23719/1527848
Esri, HERE, Garmin, (c) OpenStreetMap contributors, and the GIS user community

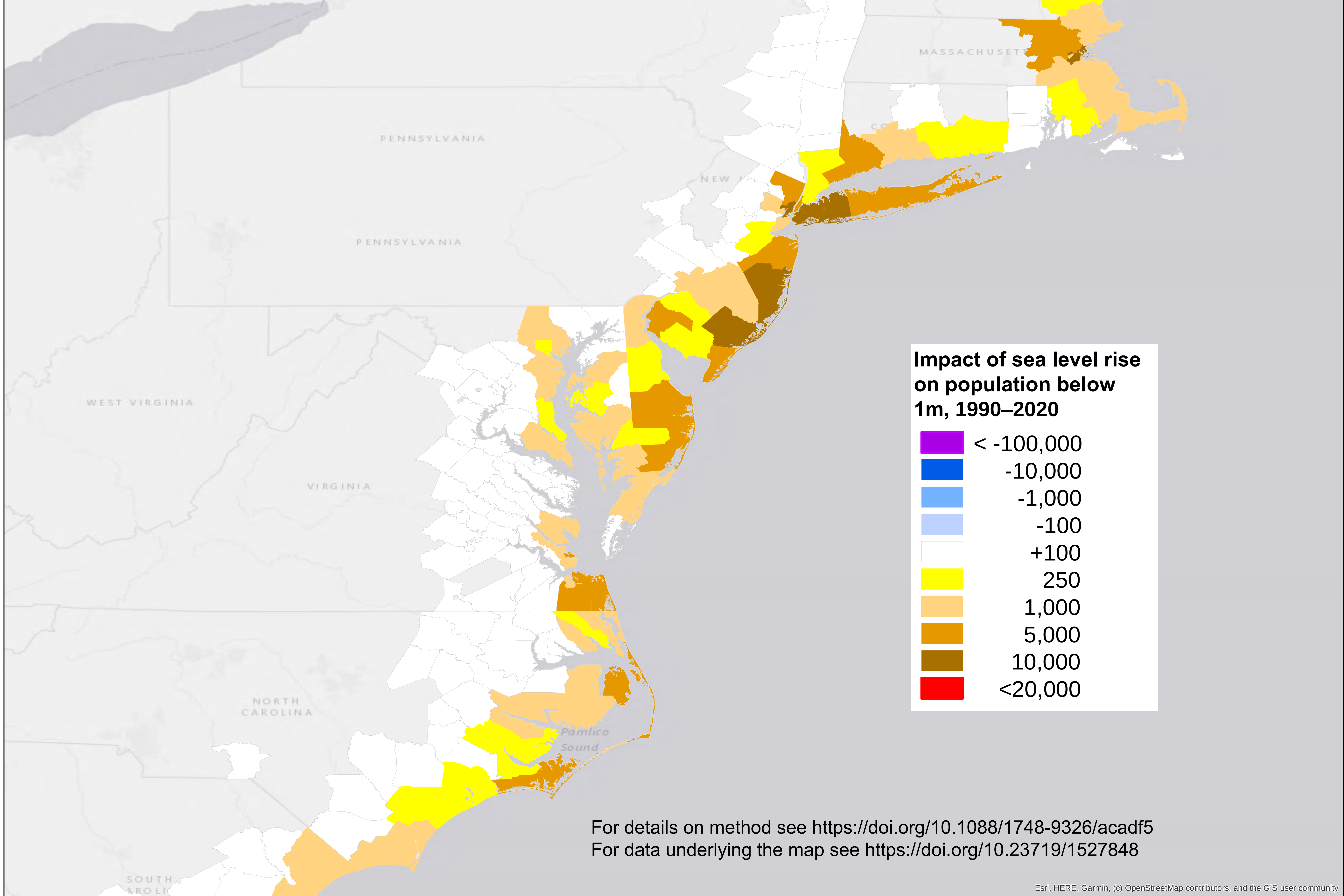
PENNSYLVANIA
PENNSYLVANIA
WEST VIRGINIA
VIRGINIA
NORTH CAROLINA
SOUTH CAROLINA
MASSACHUSETT
NEW J
Pamlico Sound
Impact of sea level rise on population below 1m, 1990–2020
< -100,000
-10,000
-1,000
-100
+100
250
1,000
5,000
10,000
<20,000
For details on method see https://doi.org/10.1088/1748-9326/acadf5
For data underlying the map see https://doi.org/10.23719/1527848
Esri, HERE, Garmin, (c) OpenStreetMap contributors, and the GIS user community

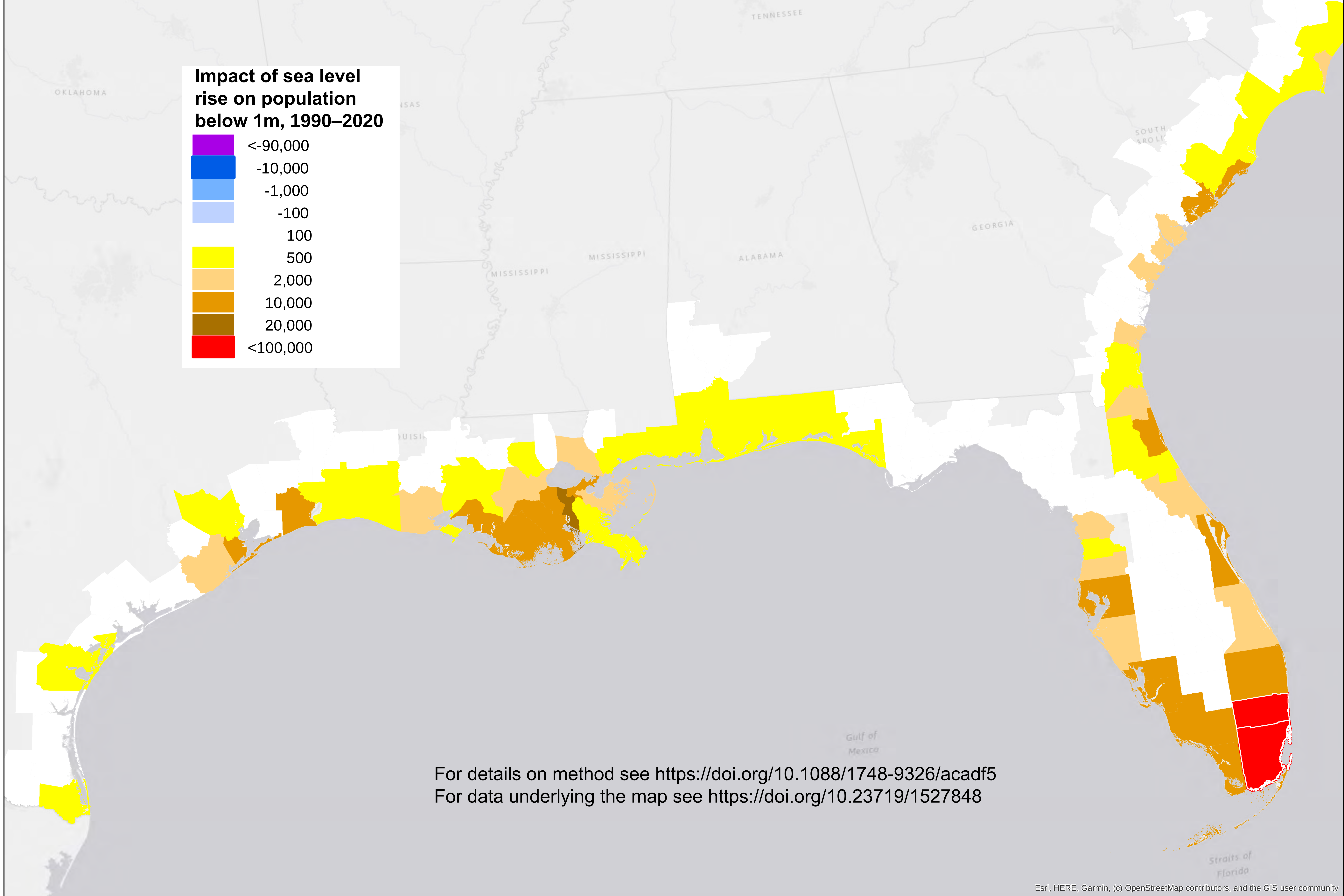
Impact of sea level rise on population below 1m, 1990–2020
<-90,000
-10,000
-1,000
-100
100
500
2,000
10,000
20,000
<100,000
OKLAHOMA
TENNESSEE
MISSISSIPPI
ALABAMA
GEORGIA
Gulf of Mexico
Straits of Florida
For details on method see https://doi.org/10.1088/1748-9326/acadf5
For data underlying the map see https://doi.org/10.23719/1527848
Esri, HERE, Garmin, (c) OpenStreetMap contributors, and the GIS user community

Change in Floodplain Population by County

These maps show county-specific estimates of the change in populations in 100-year (A zone) and 500-year (X500 zone) floodplains, as defined and estimated in the paper for which this document provides supplemental maps. Because the mapped flood zone boundaries did not change, the population changes reflect the net change of migration, births, and deaths. For further details see the Supplemental Methods section associated with this analysis. Maps that differentiate riverine and storm surge flooding are available on request.

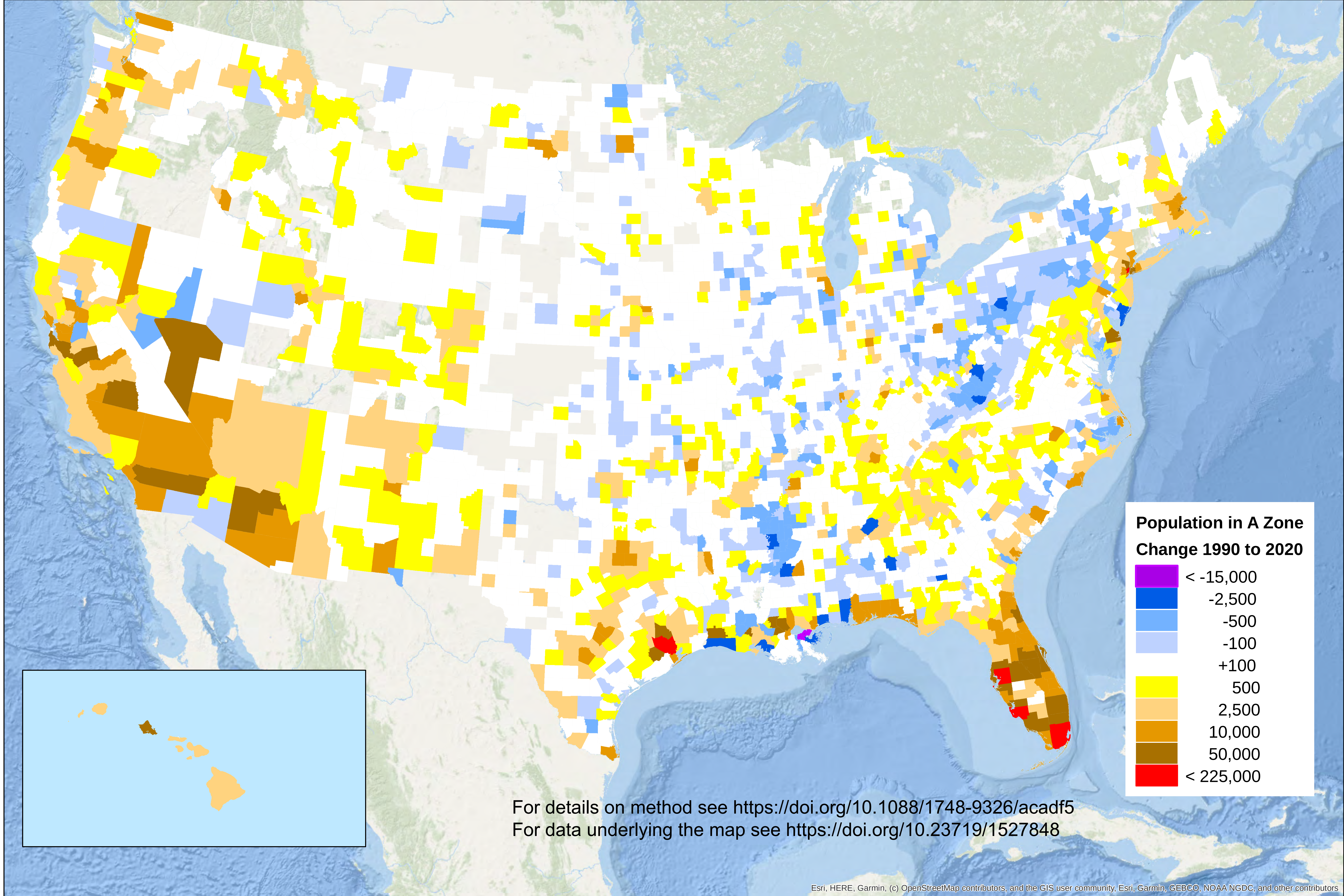

Population in A Zone
Change 1990 to 2020
< -15,000
-2,500
-500
-100
+100
500
2,500
10,000
50,000
< 225,000
For details on method see https://doi.org/10.1088/1748-9326/acadf5
For data underlying the map see https://doi.org/10.23719/1527848
Esri, HERE, Garmin, (c) OpenStreetMap contributors, and the GIS user community, Esri, Garmin, GEBCO, NOAA NGDC, and other contributors

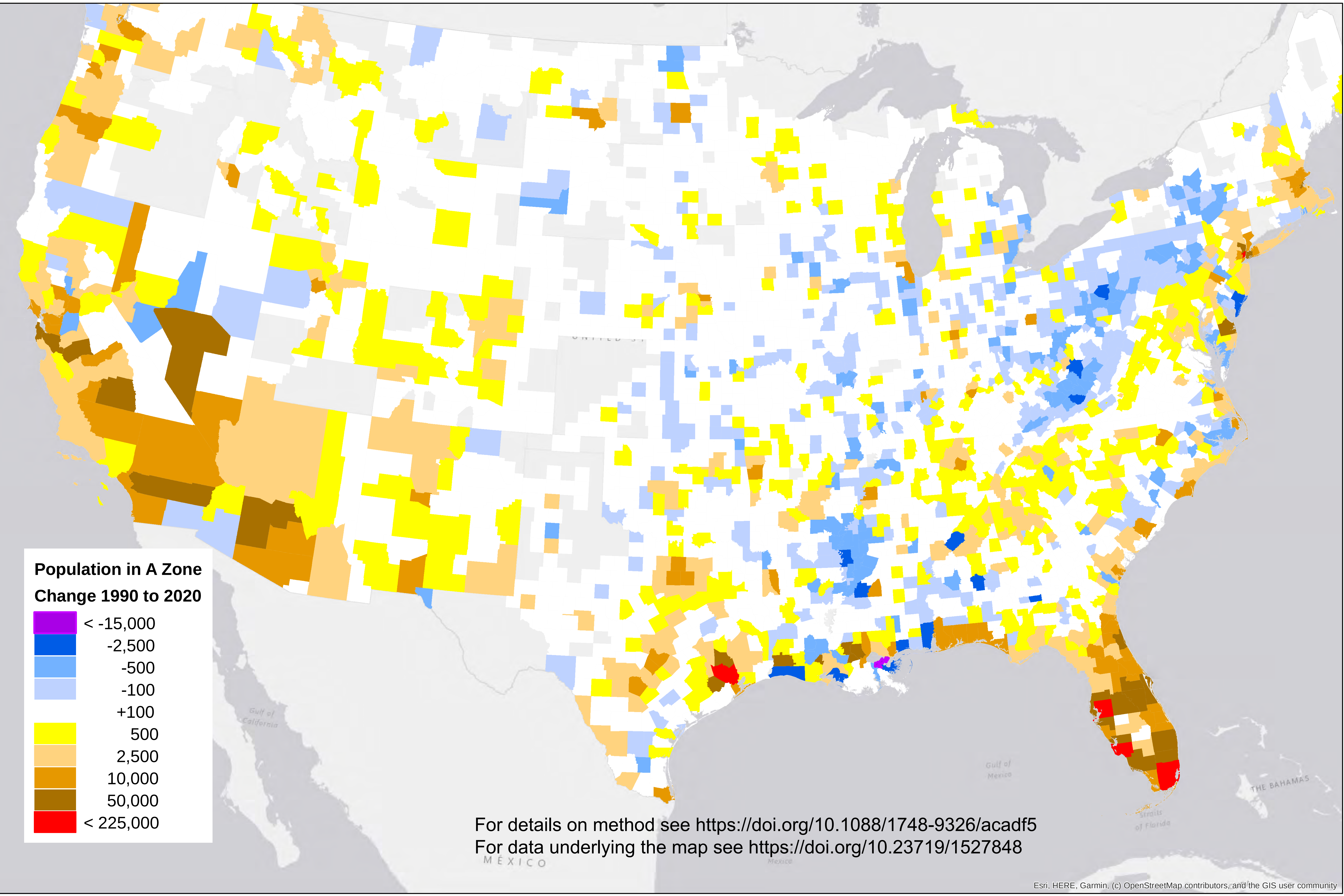

For details on method see https://doi.org/10.1088/1748-9326/acadf5
For data underlying the map see https://doi.org/10.23719/1527848

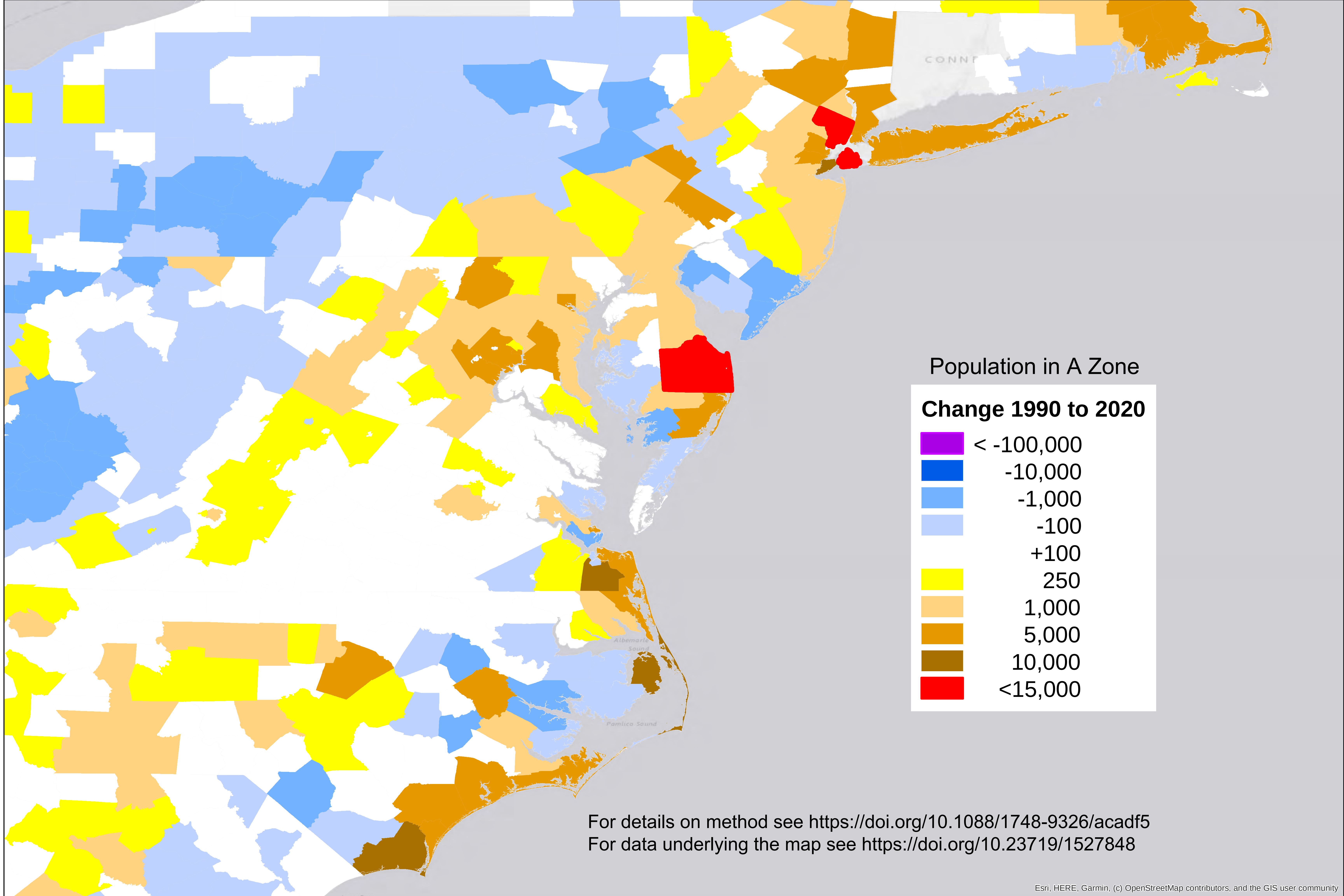

Population in A Zone
Change 1990 to 2020
< -100,000
-10,000
-1,000
-100
+100
250
1,000
5,000
10,000
<15,000
For details on method see https://doi.org/10.1088/1748-9326/acadf5
For data underlying the map see https://doi.org/10.23719/1527848
Esri, HERE, Garmin, (c) OpenStreetMap contributors, and the GIS user community

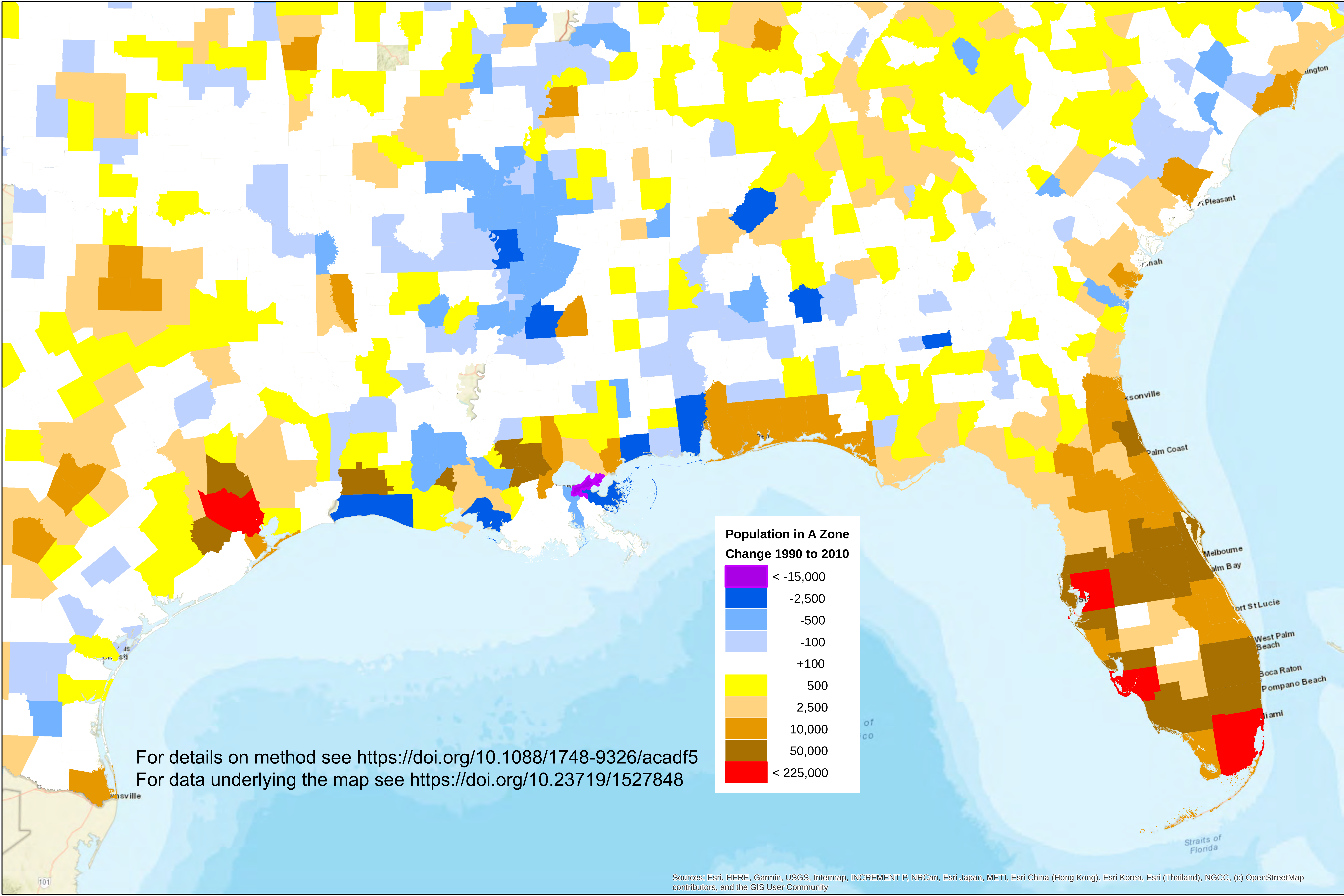
Population in A Zone
Change 1990 to 2010
< -15,000
-2,500
-500
-100
+100
500
2,500
10,000
50,000
< 225,000
For details on method see https://doi.org/10.1088/1748-9326/acadf5
For data underlying the map see https://doi.org/10.23719/1527848
Palm Coast
Melbourne
West Palm Beach
Boca Raton
Pompano Beach
Straits of Florida
Sources: Esri, HERE, Garmin, USGS, Intermap, INCREMENT P, NRCan, Esri Japan, METI, Esri China (Hong Kong), Esri Korea, Esri (Thailand), NGCC, (c) OpenStreetMap contributors, and the GIS User Community

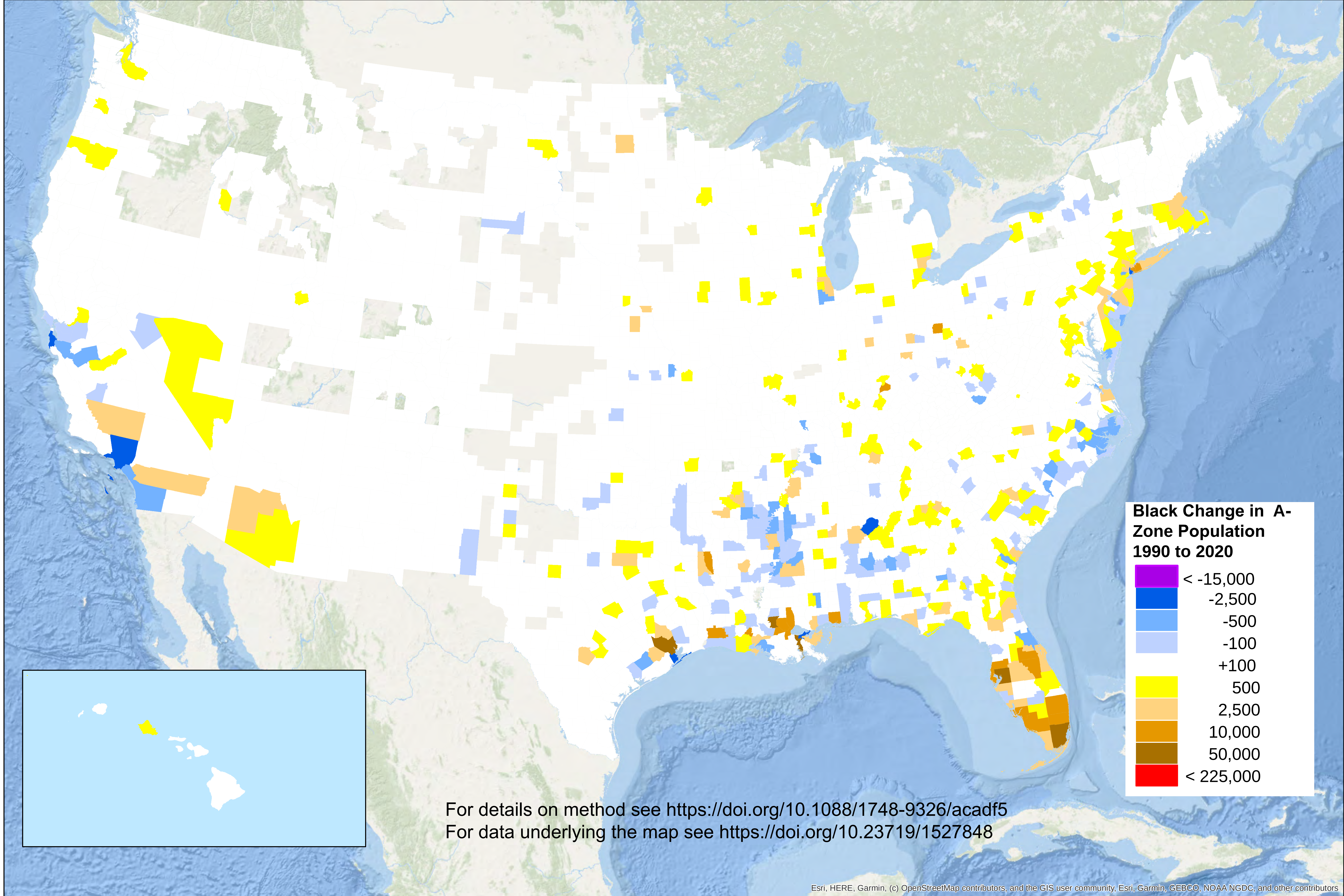
Black Change in A-Zone Population 1990 to 2020
< -15,000
-2,500
-500
-100
+100
500
2,500
10,000
50,000
< 225,000
For details on method see https://doi.org/10.1088/1748-9326/acadf5
For data underlying the map see https://doi.org/10.23719/1527848
Esri, HERE, Garmin, (c) OpenStreetMap contributors, and the GIS user community, Esri, Garmin, GEBCO, NOAA NGDC, and other contributors

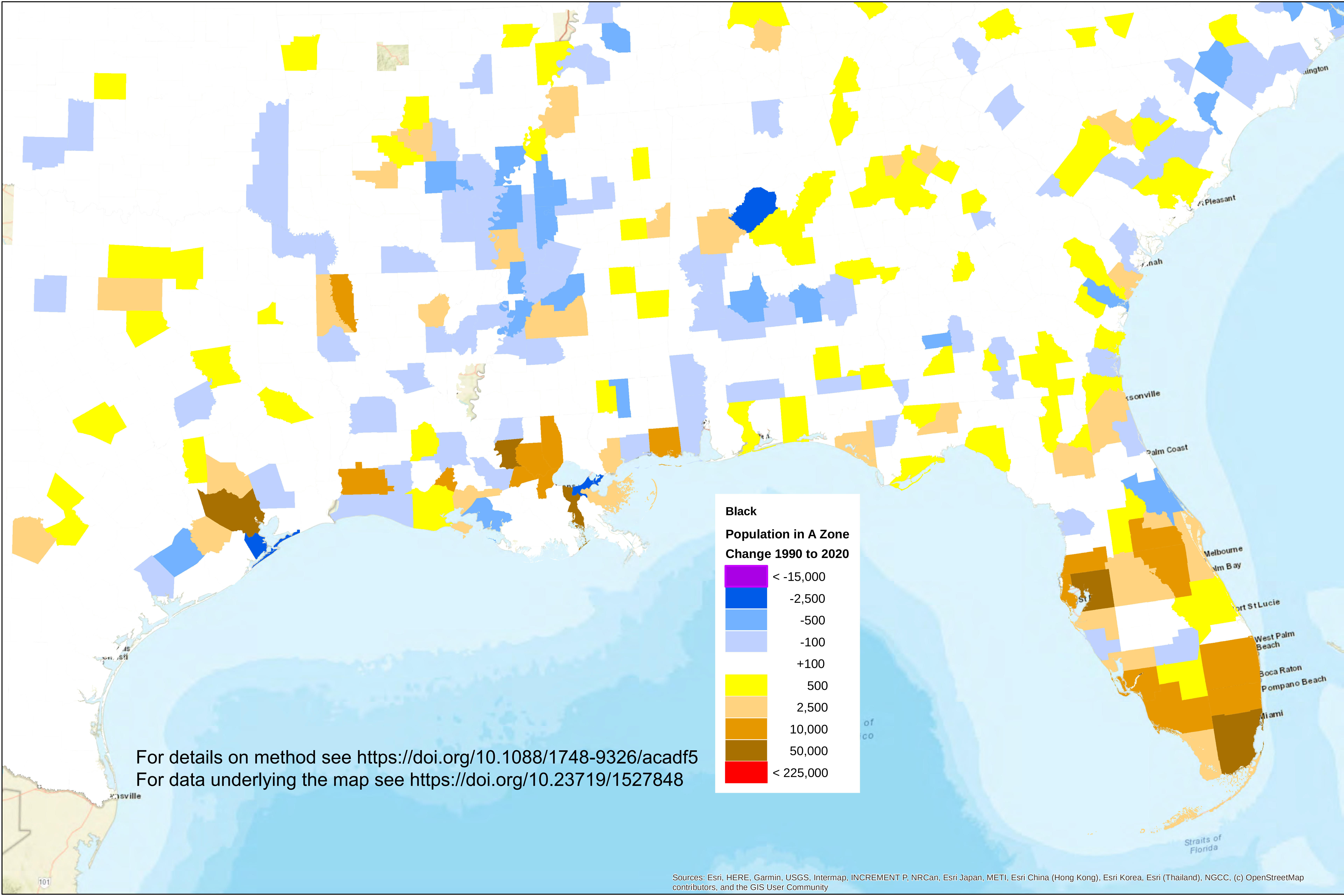
Black
Population in A Zone
Change 1990 to 2020
< -15,000
-2,500
-500
-100
+100
500
2,500
10,000
50,000
< 225,000
For details on method see https://doi.org/10.1088/1748-9326/acadf5
For data underlying the map see https://doi.org/10.23719/1527848
Palm Coast
Melbourne
West Palm Beach
Boca Raton
Pompano Beach
Straits of Florida
Sources: Esri, HERE, Garmin, USGS, Intermap, INCREMENT P, NRCan, Esri Japan, METI, Esri China (Hong Kong), Esri Korea, Esri (Thailand), NGCC, (c) OpenStreetMap contributors, and the GIS User Community

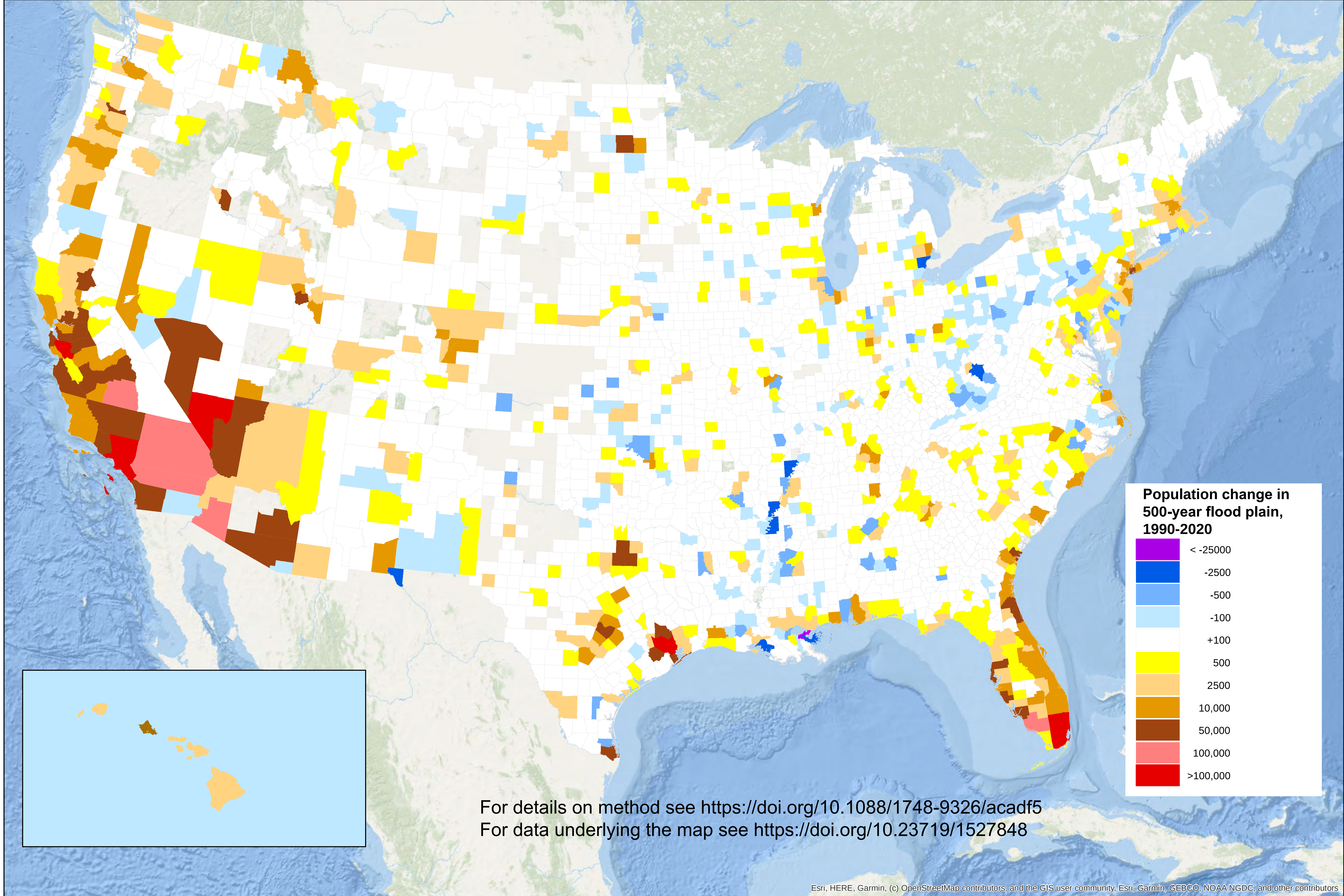
Population change in 500-year flood plain, 1990-2020
< -25000
-2500
-500
-100
+100
500
2500
10,000
50,000
100,000
>100,000
For details on method see https://doi.org/10.1088/1748-9326/acadf5
For data underlying the map see https://doi.org/10.23719/1527848
Esri, HERE, Garmin, (c) OpenStreetMap contributors, and the GIS user community, Esri, Garmin, GEBCO, NOAA NGDC, and other contributors

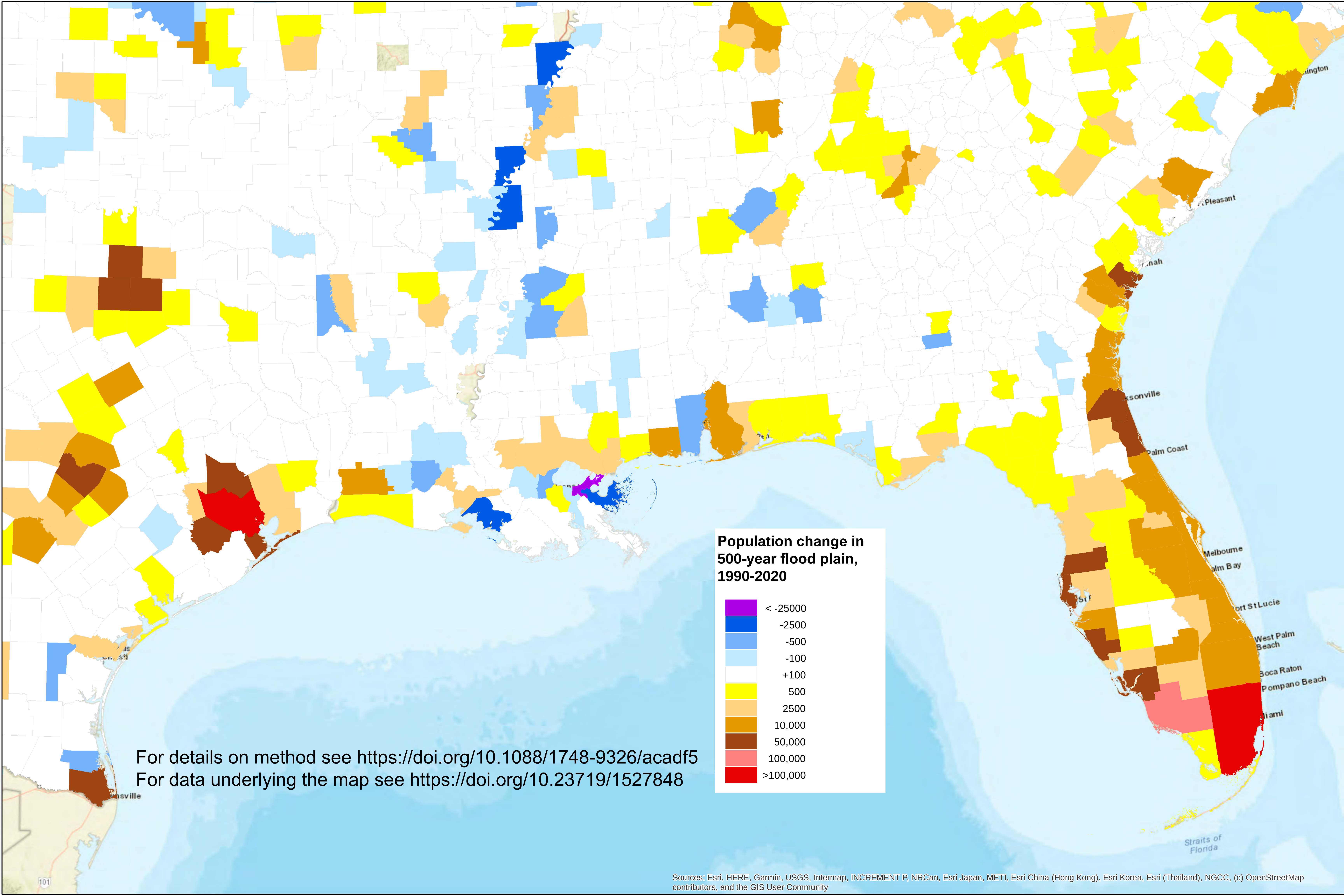
Population change in 500-year flood plain, 1990-2020
< -25000
-2500
-500
-100
+100
500
2500
10,000
50,000
100,000
>100,000
Palm Coast
Melbourne
West Palm Beach
Boca Raton
Pompano Beach
Straits of Florida
For details on method see https://doi.org/10.1088/1748-9326/acadf5
For data underlying the map see https://doi.org/10.23719/1527848
Sources: Esri, HERE, Garmin, USGS, Intermap, INCREMENT P, NRCan, Esri Japan, METI, Esri China (Hong Kong), Esri Korea, Esri (Thailand), NGCC, (c) OpenStreetMap contributors, and the GIS User Community

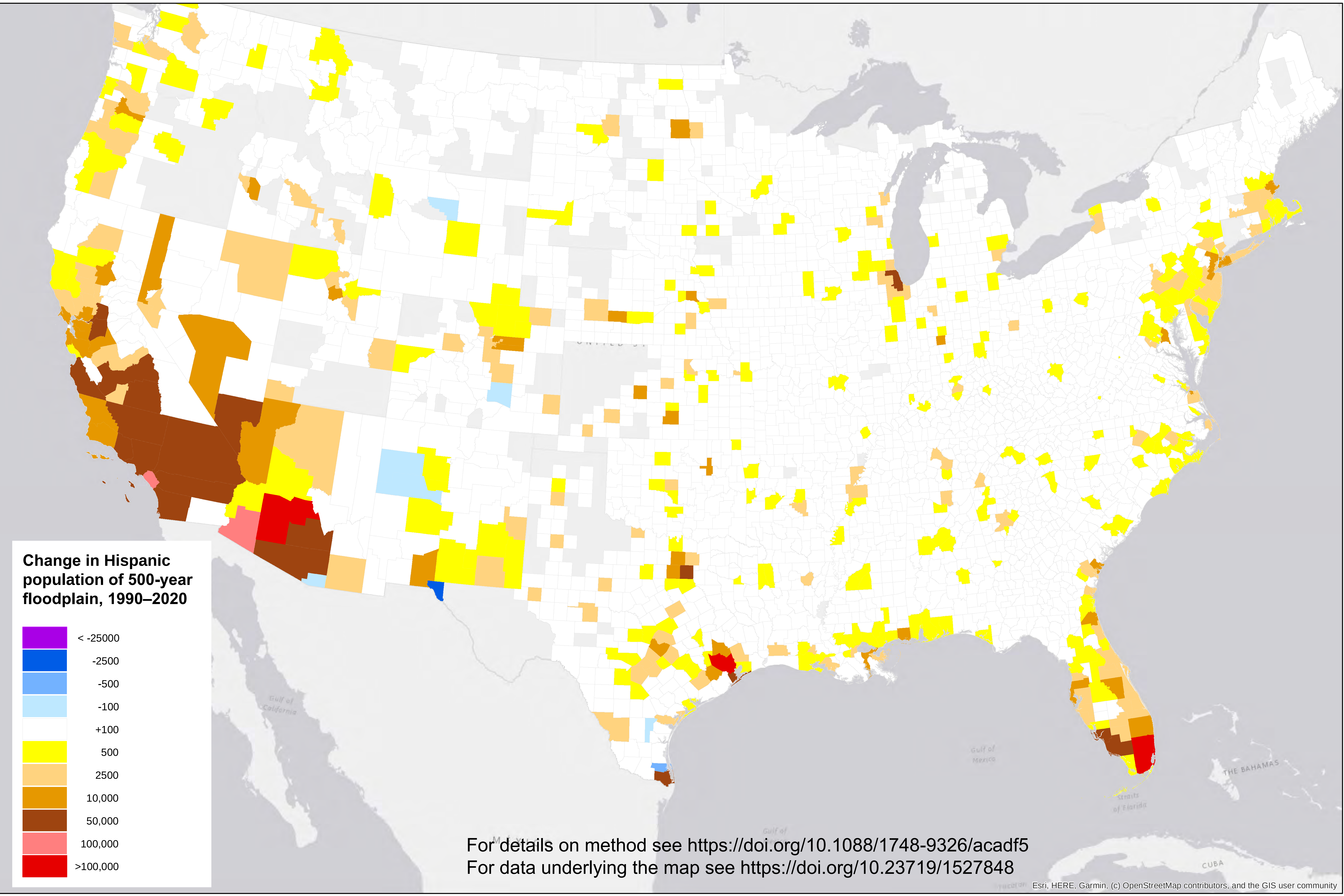

Change in Hispanic population of 500-year floodplain, 1990–2020
< -25000
-2500
-500
-100
+100
500
2500
10,000
50,000
100,000
>100,000
For details on method see https://doi.org/10.1088/1748-9326/acadf5
For data underlying the map see https://doi.org/10.23719/1527848
THE BAHAMAS
CUBA
Esri, HERE, Garmin, (c) OpenStreetMap contributors, and the GIS user community

Ratios of Disproportionality by County

These maps show county-specific “ratios of disproportionality” as defined in the paper for which this document provides supplemental maps, expressed as a percentage. For each county, this ratio is calculated as the percentage of Black (or Hispanic) residents of a county who live below one meter, divided by the percentage of all residents in the county who live below one meter (times 100). The legend uses the term “Relative Habitation” to conserve space. When the ratio is 100, there is no disproportionality. If the ratio is greater (or less) than 100, then the Black (or Hispanic) population is more (or less) exposed to sea level rise than the general population of the county (ignoring different likelihoods of shore protection). For further details see the Supplemental Methods.

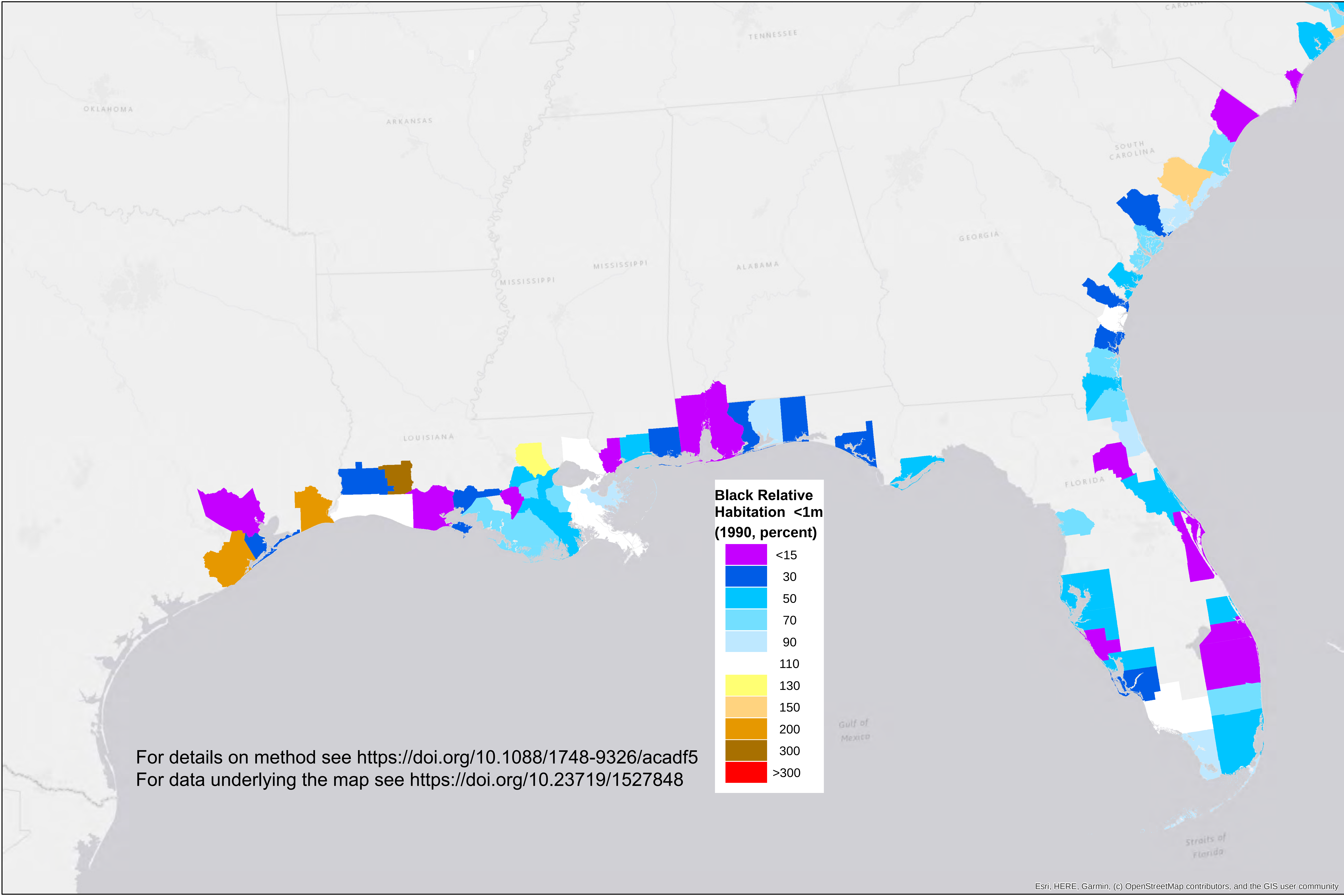
Black Relative Habitation <1m (1990, percent)
<15
30
50
70
90
110
130
150
200
300
>300
For details on method see https://doi.org/10.1088/1748-9326/acadf5
For data underlying the map see https://doi.org/10.23719/1527848
OKLAHOMA
ARKANSAS
TENNESSEE
MISSISSIPPI
MISSISSIPPI
ALABAMA
GEORGIA
SOUTH CAROLINA
LOUISIANA
FLORIDA
Gulf of Mexico
Straits of Florida
Esri, HERE, Garmin, (c) OpenStreetMap contributors, and the GIS user community

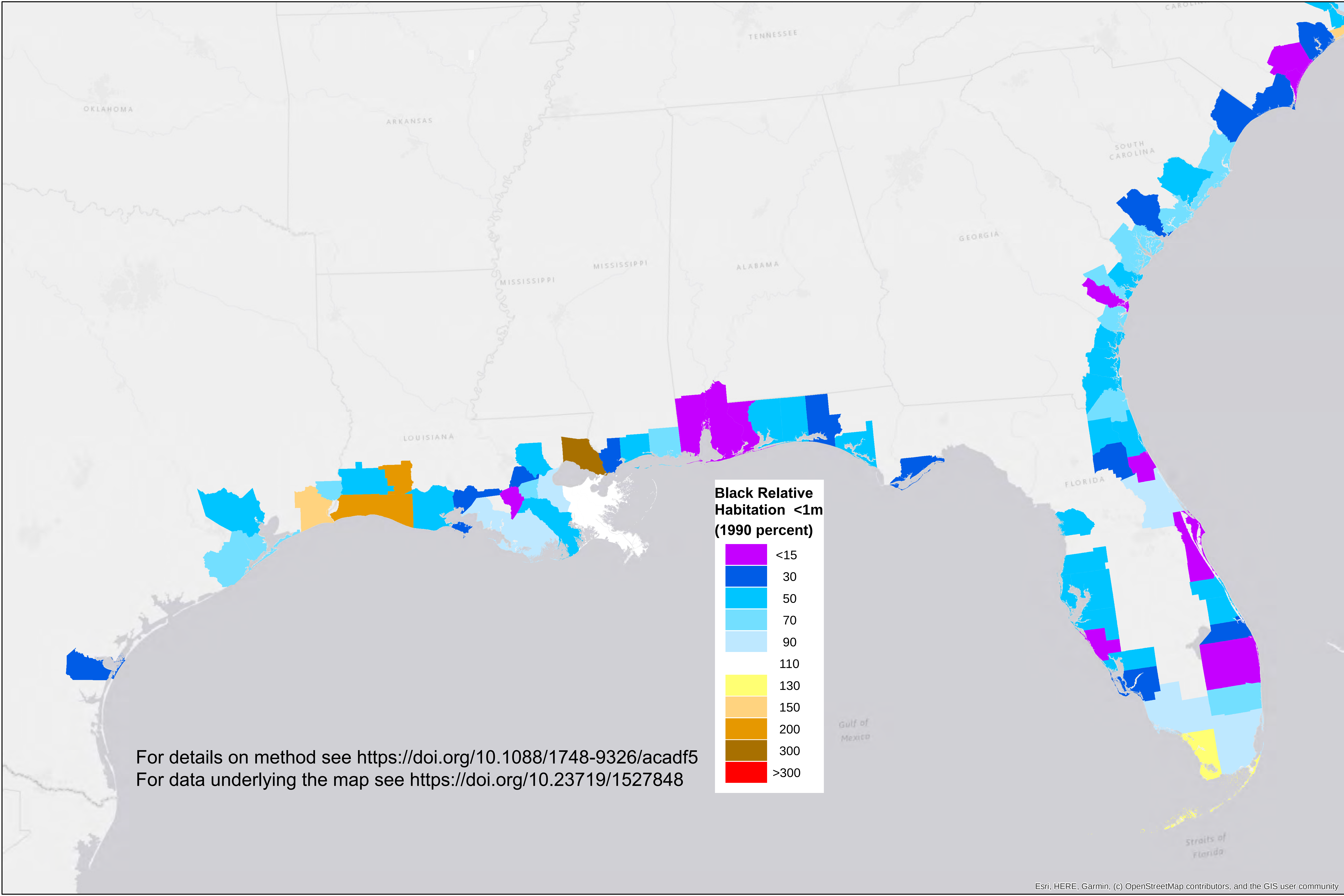
Black Relative Habitation <1m (1990 percent)
<15
30
50
70
90
110
130
150
200
300
>300
For details on method see https://doi.org/10.1088/1748-9326/acadf5
For data underlying the map see https://doi.org/10.23719/1527848
OKLAHOMA
ARKANSAS
TENNESSEE
MISSISSIPPI
ALABAMA
GEORGIA
SOUTH CAROLINA
LOUISIANA
FLORIDA
Gulf of Mexico
Straits of Florida
Esri, HERE, Garmin, (c) OpenStreetMap contributors, and the GIS user community

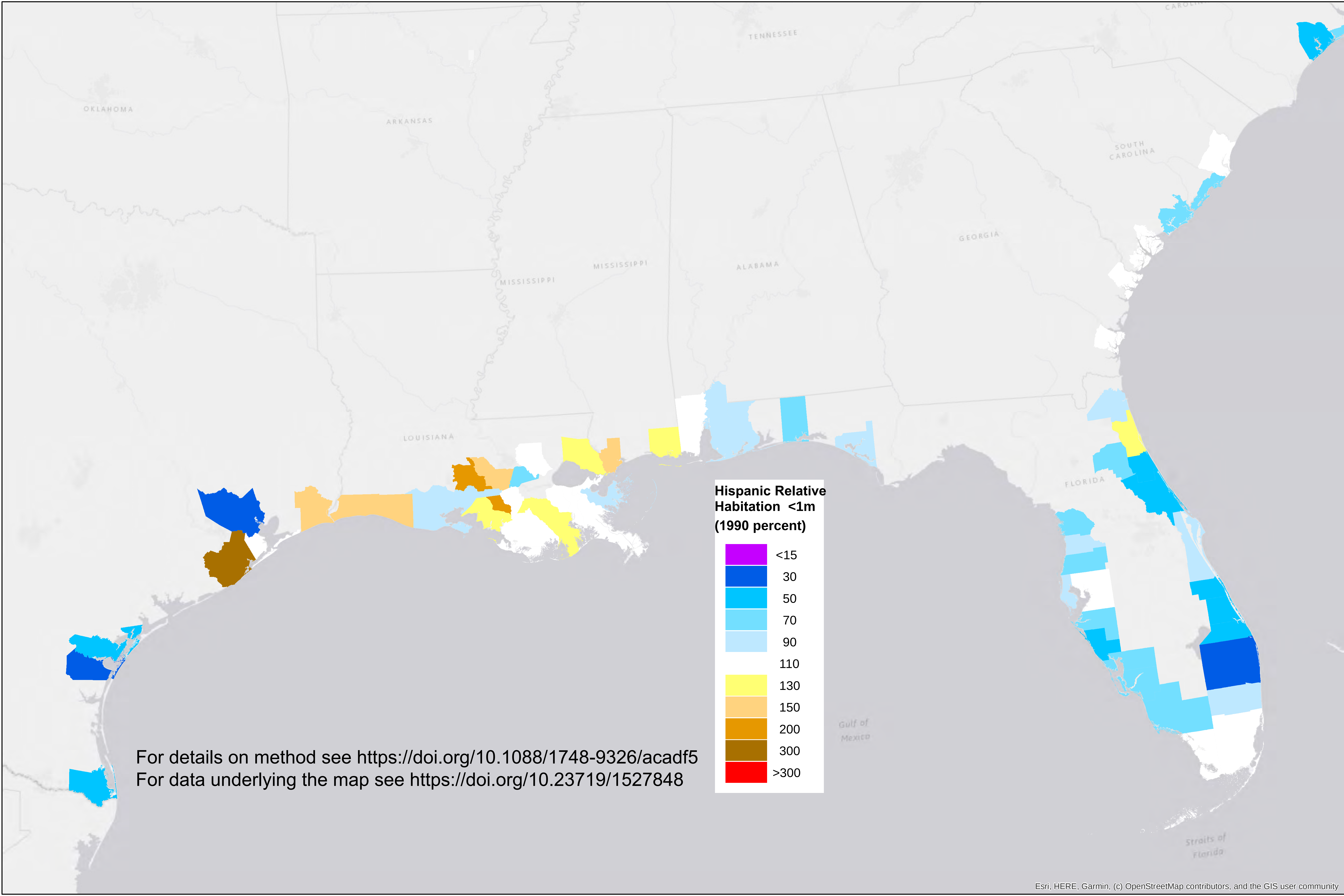
TENNESSEE
OKLAHOMA
ARKANSAS
SOUTH CAROLINA
GEORGIA
MISSISSIPPI
MISSISSIPPI
ALABAMA
LOUISIANA
FLORIDA
Gulf of Mexico
Straits of Florida
Hispanic Relative Habitation <1m
(1990 percent)
<15
30
50
70
90
110
130
150
200
300
>300
For details on method see https://doi.org/10.1088/1748-9326/acadf5
For data underlying the map see https://doi.org/10.23719/1527848
Esri, HERE, Garmin, (c) OpenStreetMap contributors, and the GIS user community

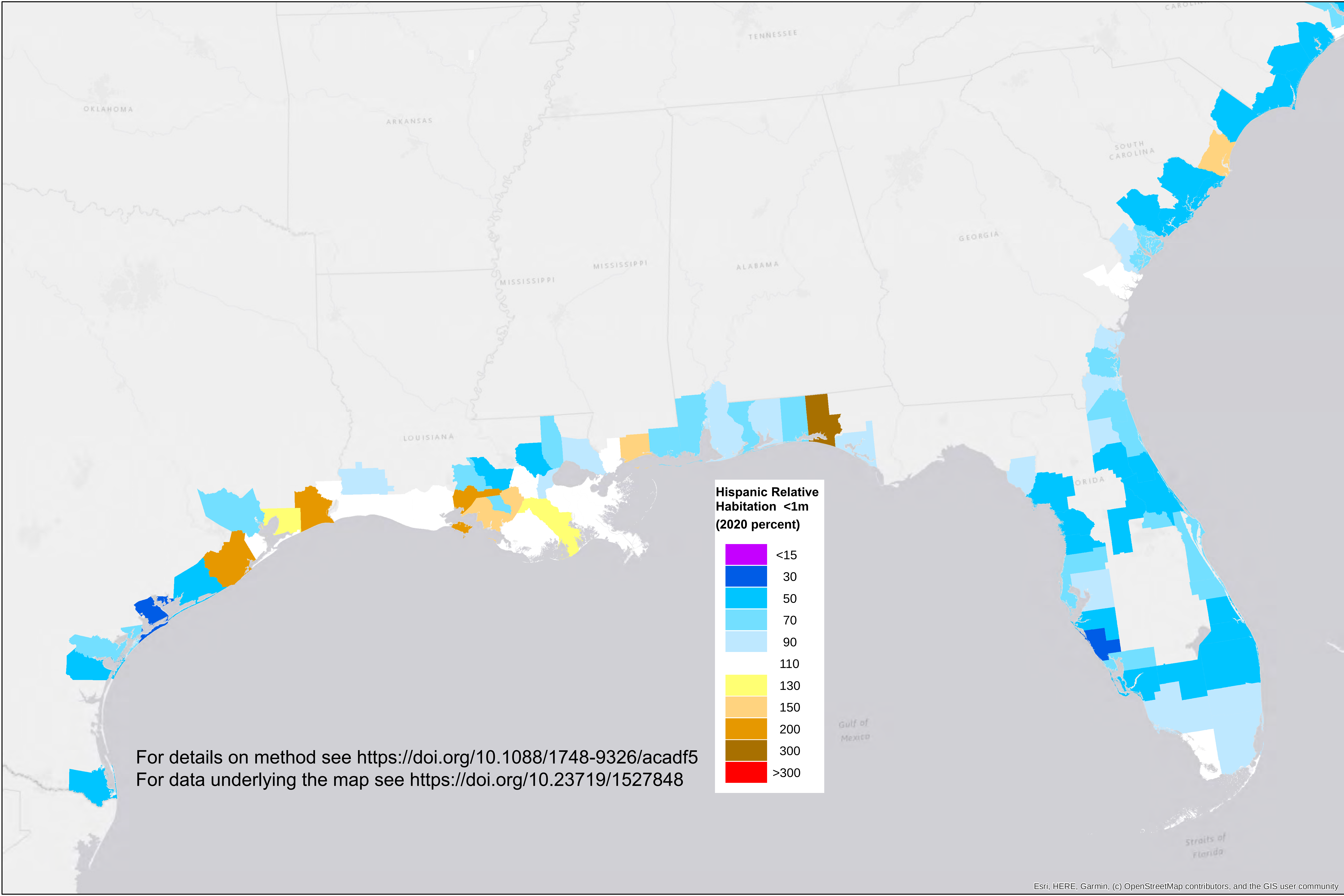

OKLAHOMA
ARKANSAS
TENNESSEE
MISSISSIPPI
ALABAMA
GEORGIA
SOUTH CAROLINA
LOUISIANA
Hispanic Relative Habitation <1m
(2020 percent)
<15
30
50
70
90
110
130
150
200
300
>300
Gulf of Mexico
Straits of Florida
For details on method see https://doi.org/10.1088/1748-9326/acadf5
For data underlying the map see https://doi.org/10.23719/1527848
Esri, HERE, Garmin, (c) OpenStreetMap contributors, and the GIS user community

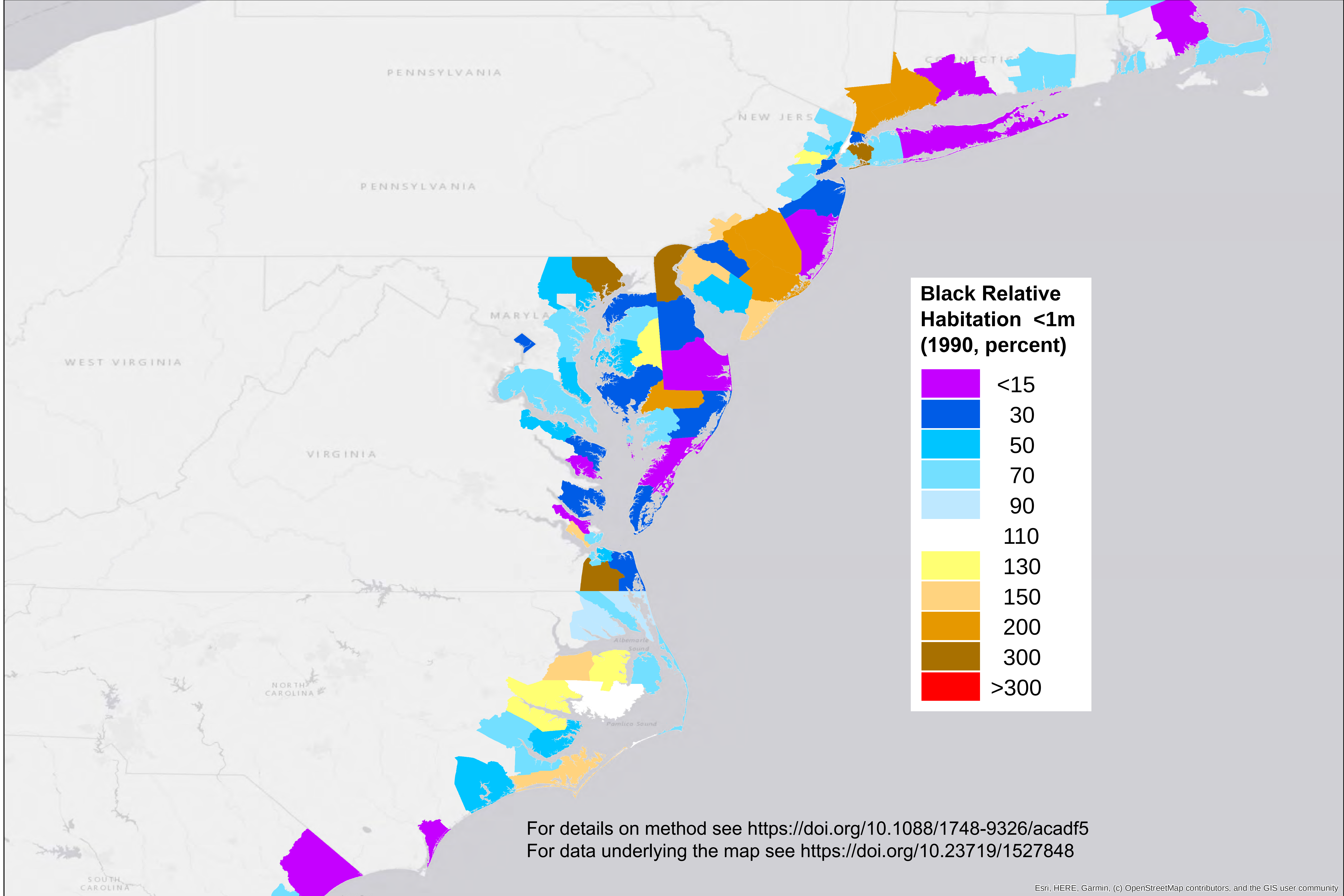
PENNSYLVANIA
PENNSYLVANIA
NEW JERS
CONNECTI
MARYLA
WEST VIRGINIA
VIRGINIA
NORTH CAROLINA
SOUTH CAROLINA
Albemarle Sound
Pamlico Sound
Black Relative Habitation <1m (1990, percent)
<15
30
50
70
90
110
130
150
200
300
>300
For details on method see https://doi.org/10.1088/1748-9326/acadf5
For data underlying the map see https://doi.org/10.23719/1527848
Esri, HERE, Garmin, (c) OpenStreetMap contributors, and the GIS user community

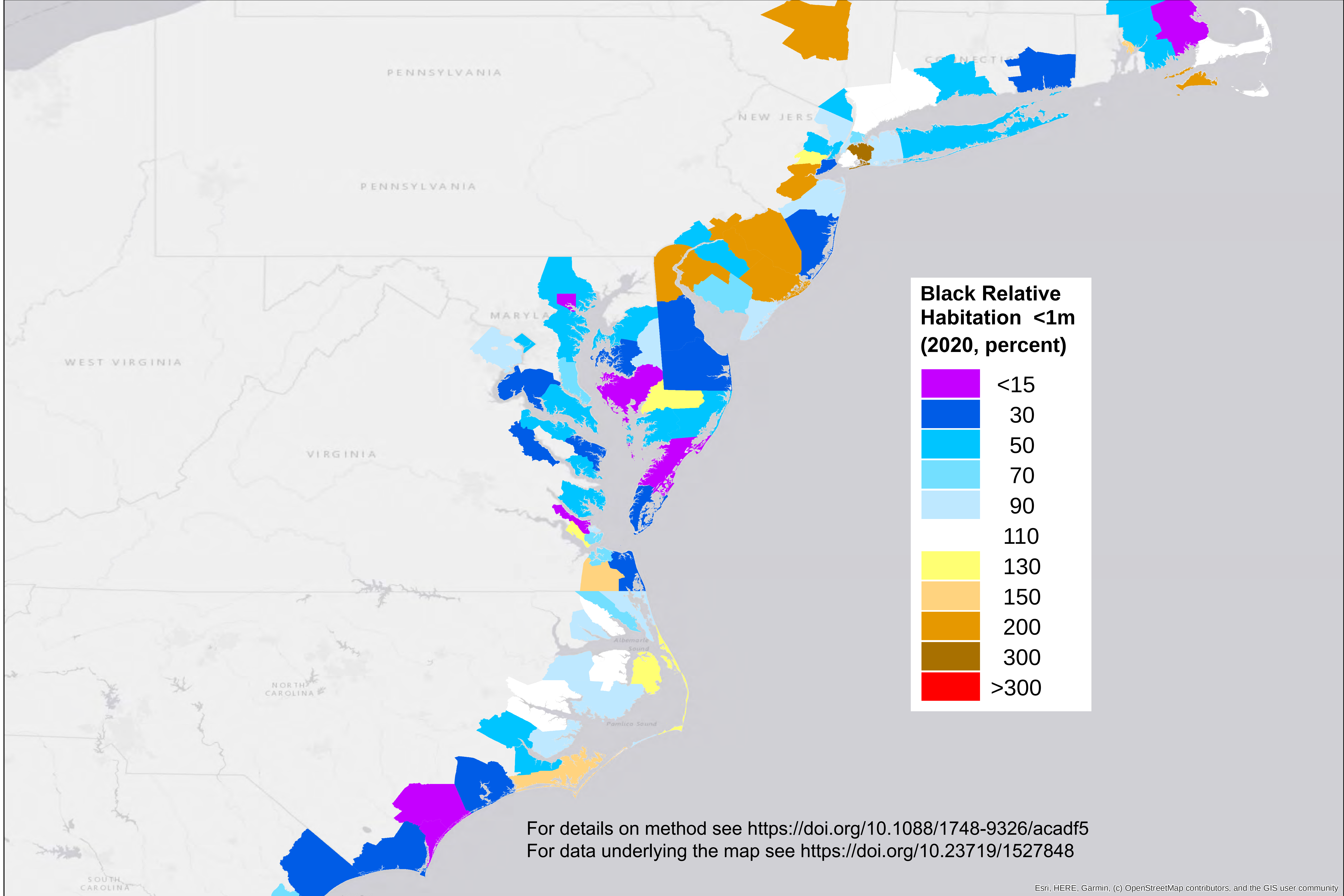

For details on method see https://doi.org/10.1088/1748-9326/acadf5
For data underlying the map see https://doi.org/10.23719/1527848

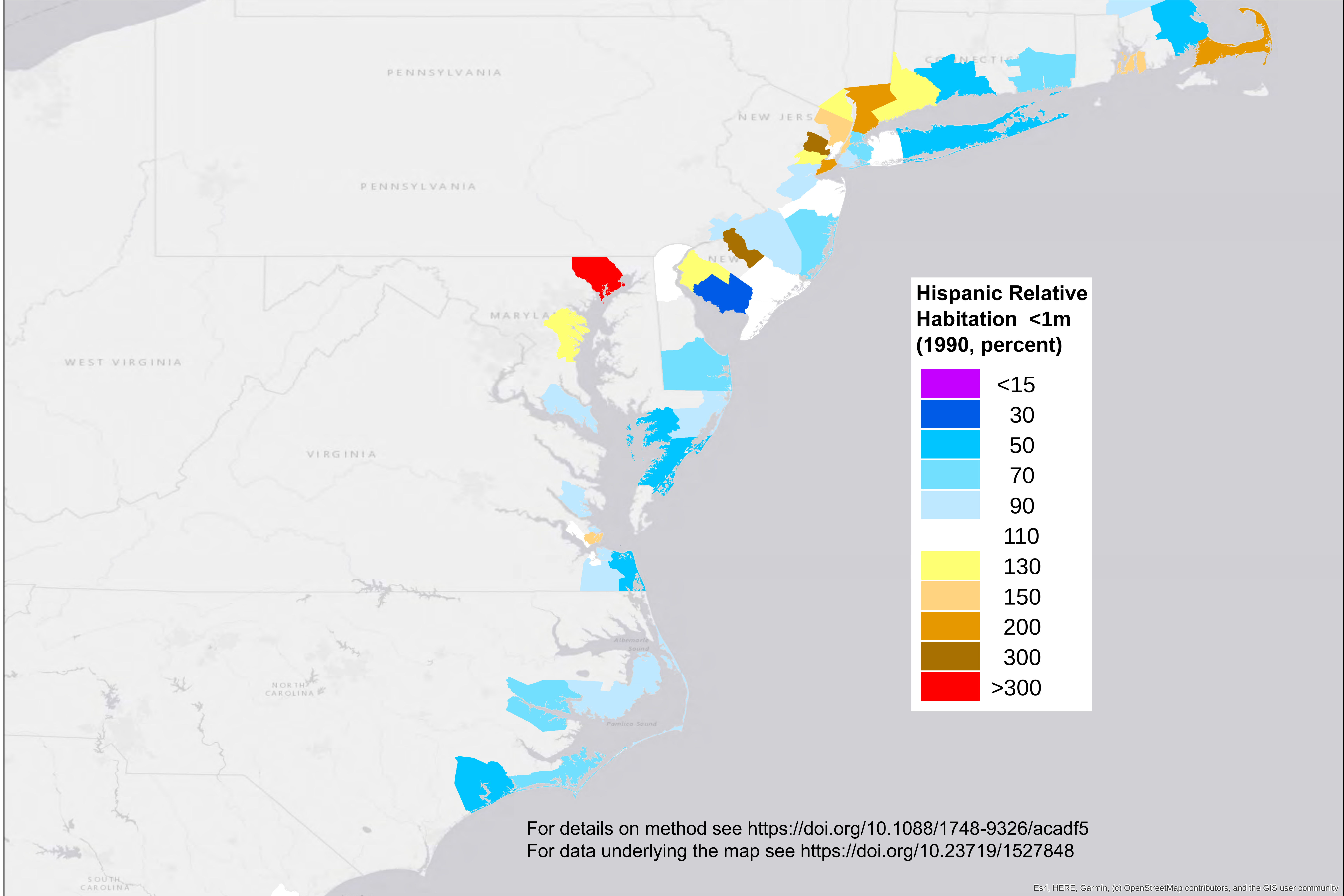

For details on method see https://doi.org/10.1088/1748-9326/acadf5
For data underlying the map see https://doi.org/10.23719/1527848

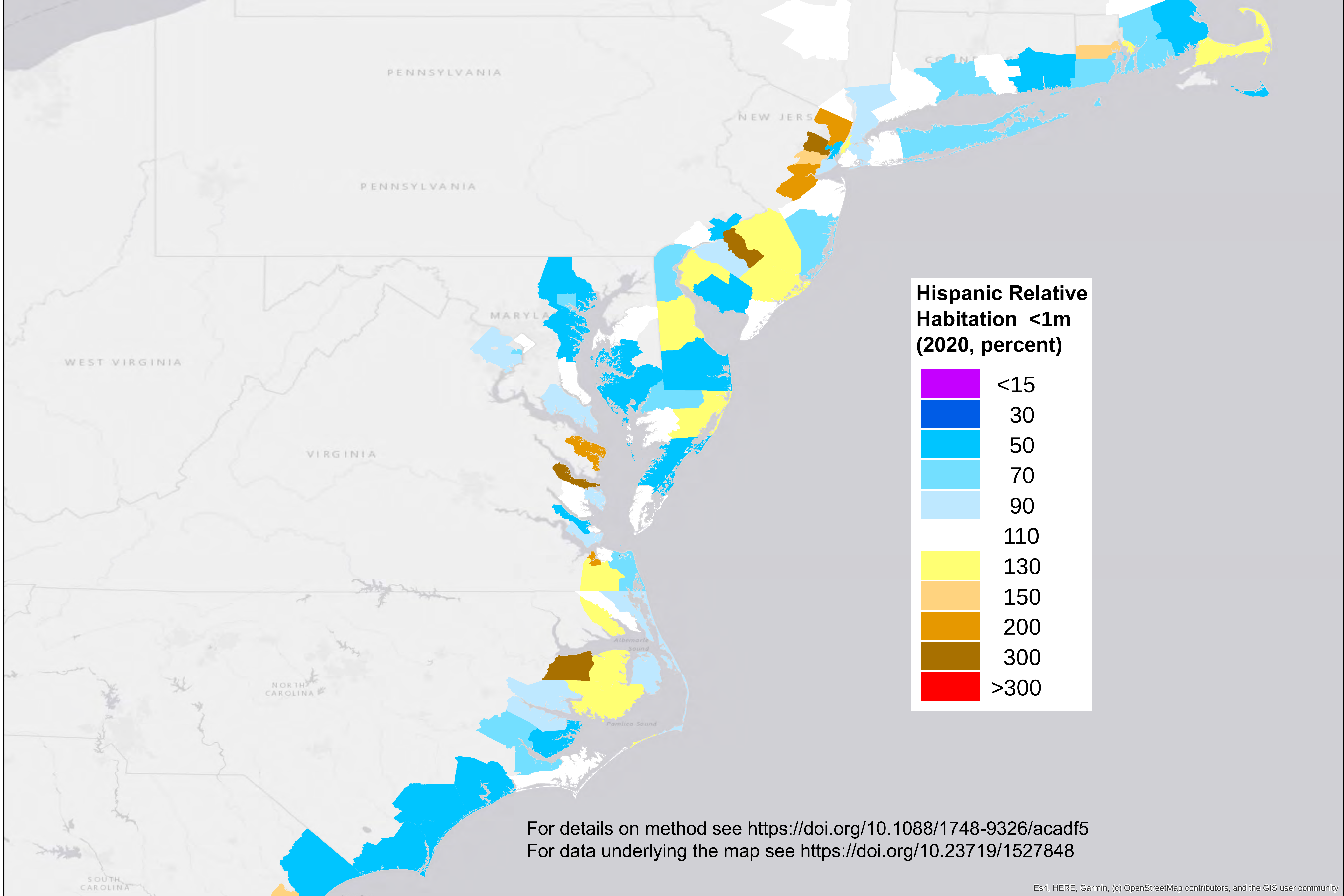
PENNSYLVANIA
PENNSYLVANIA
WEST VIRGINIA
VIRGINIA
NORTH CAROLINA
SOUTH CAROLINA
Albemarle Sound
Pamlico Sound
Hispanic Relative Habitation <1m (2020, percent)
<15
30
50
70
90
110
130
150
200
300
>300
For details on method see https://doi.org/10.1088/1748-9326/acadf5
For data underlying the map see https://doi.org/10.23719/1527848
Esri, HERE, Garmin, (c) OpenStreetMap contributors, and the GIS user community

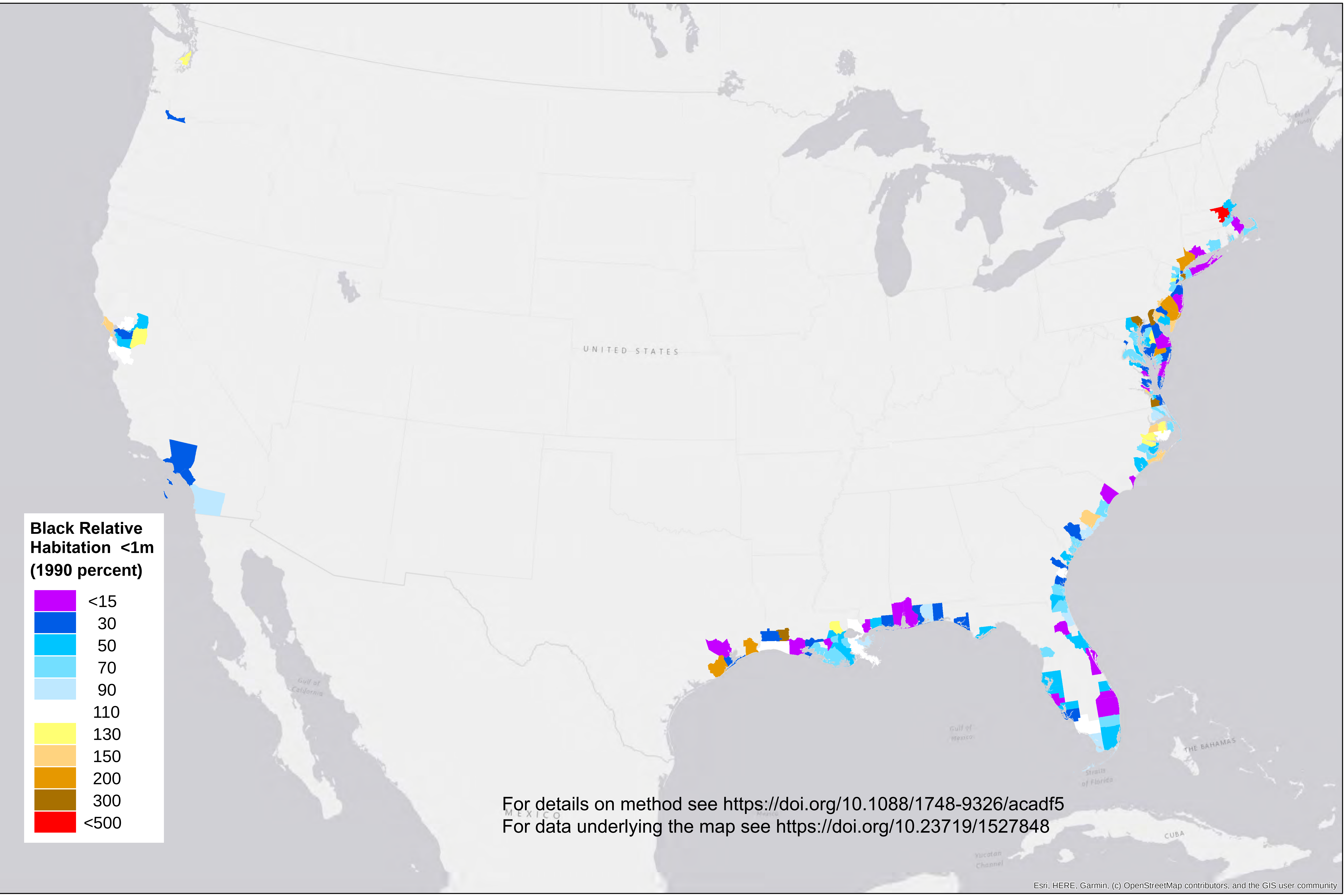
Black Relative Habitation <1m (1990 percent)
<15
30
50
70
90
110
130
150
200
300
<500
UNITED STATES
MEXICO
THE BAHAMAS
CUBA
For details on method see https://doi.org/10.1088/1748-9326/acadf5
For data underlying the map see https://doi.org/10.23719/1527848
Esri, HERE, Garmin, (c) OpenStreetMap contributors, and the GIS user community

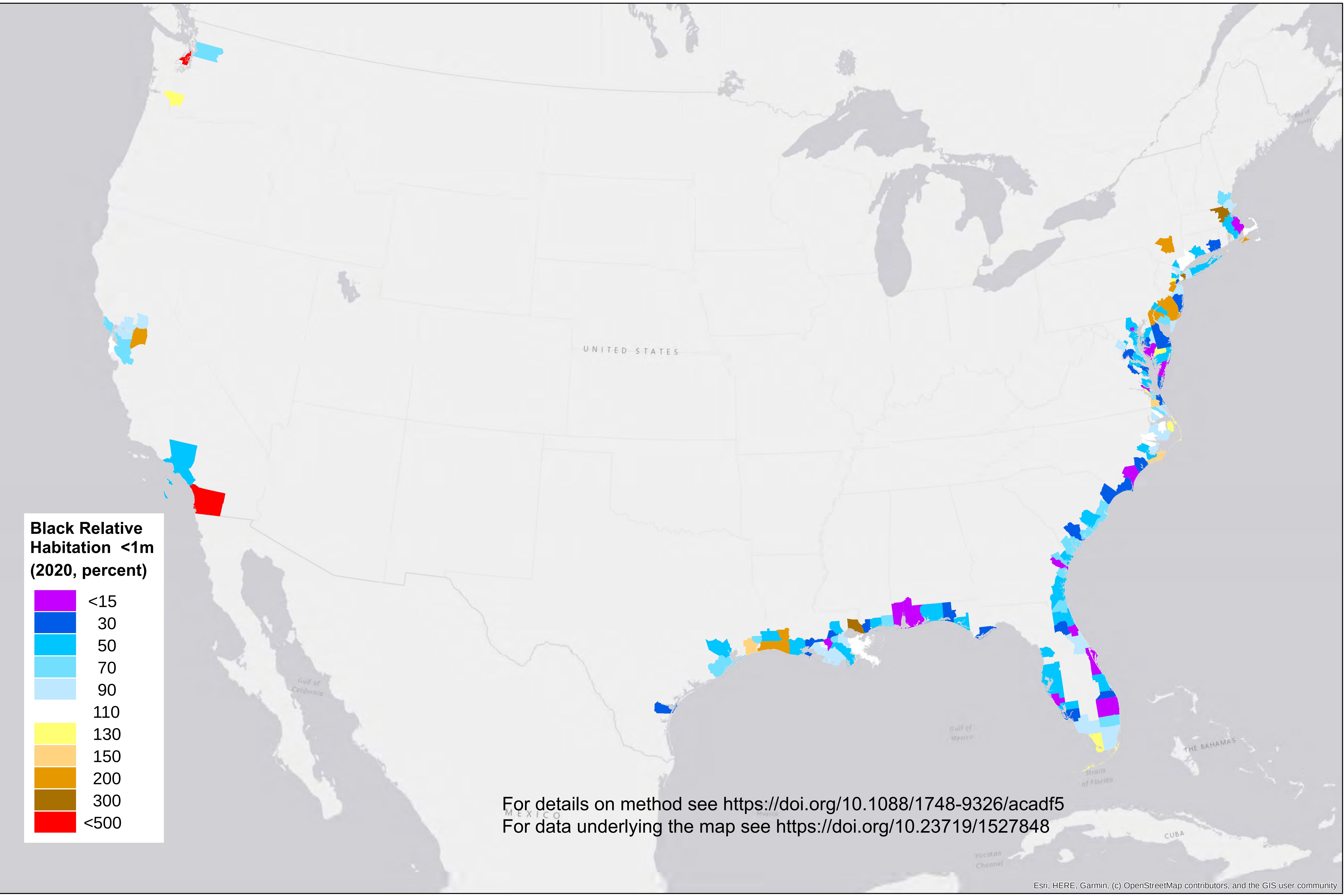
Black Relative Habitation <1m (2020, percent)
<15
30
50
70
90
110
130
150
200
300
<500
UNITED STATES
MEXICO
THE BAHAMAS
CUBA
For details on method see https://doi.org/10.1088/1748-9326/acadf5
For data underlying the map see https://doi.org/10.23719/1527848
Esri, HERE, Garmin, (c) OpenStreetMap contributors, and the GIS user community

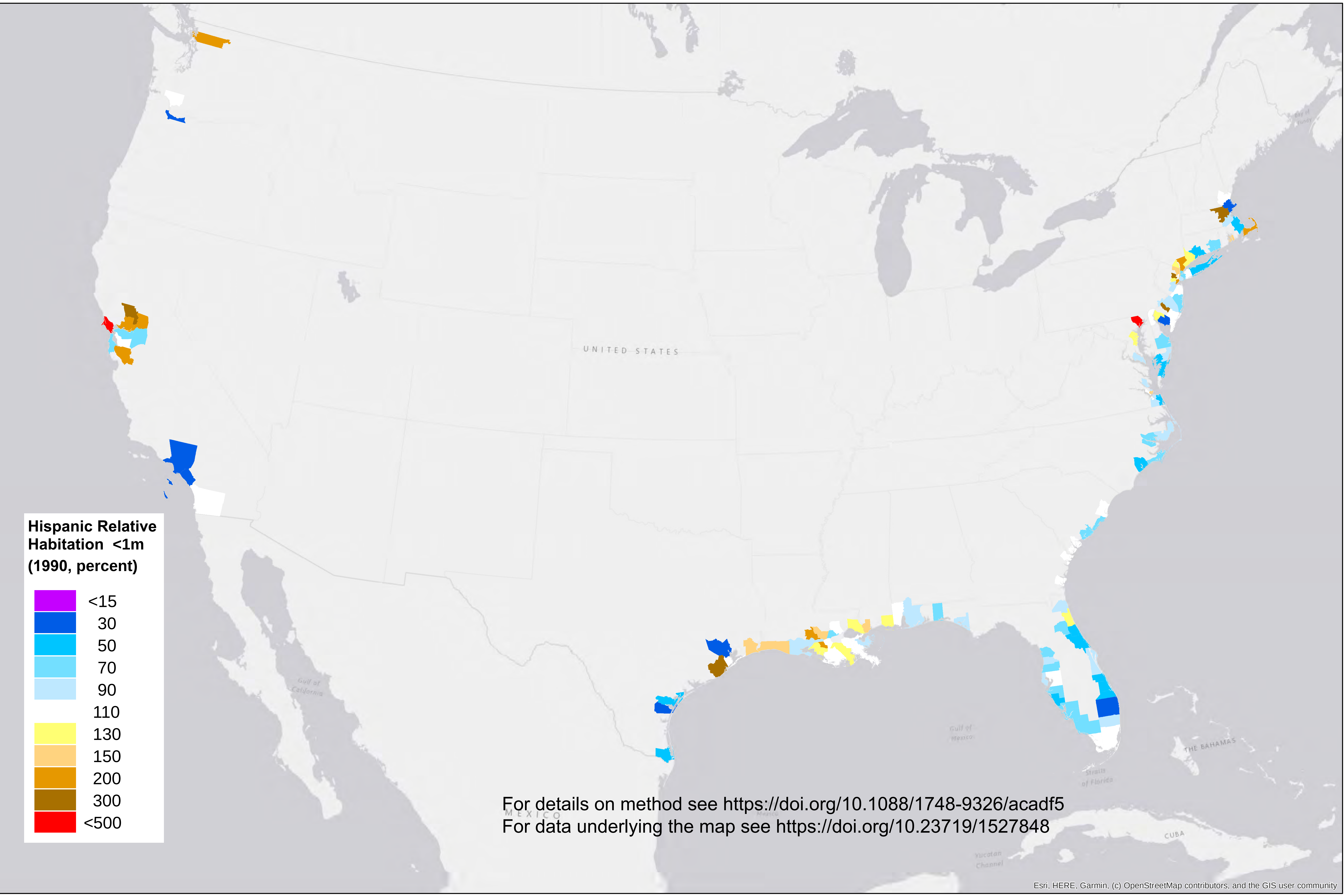
Hispanic Relative Habitation <1m (1990, percent)
<15
30
50
70
90
110
130
150
200
300
<500
UNITED STATES
MEXICO
THE BAHAMAS
CUBA
For details on method see https://doi.org/10.1088/1748-9326/acadf5
For data underlying the map see https://doi.org/10.23719/1527848
Esri, HERE, Garmin, (c) OpenStreetMap contributors, and the GIS user community

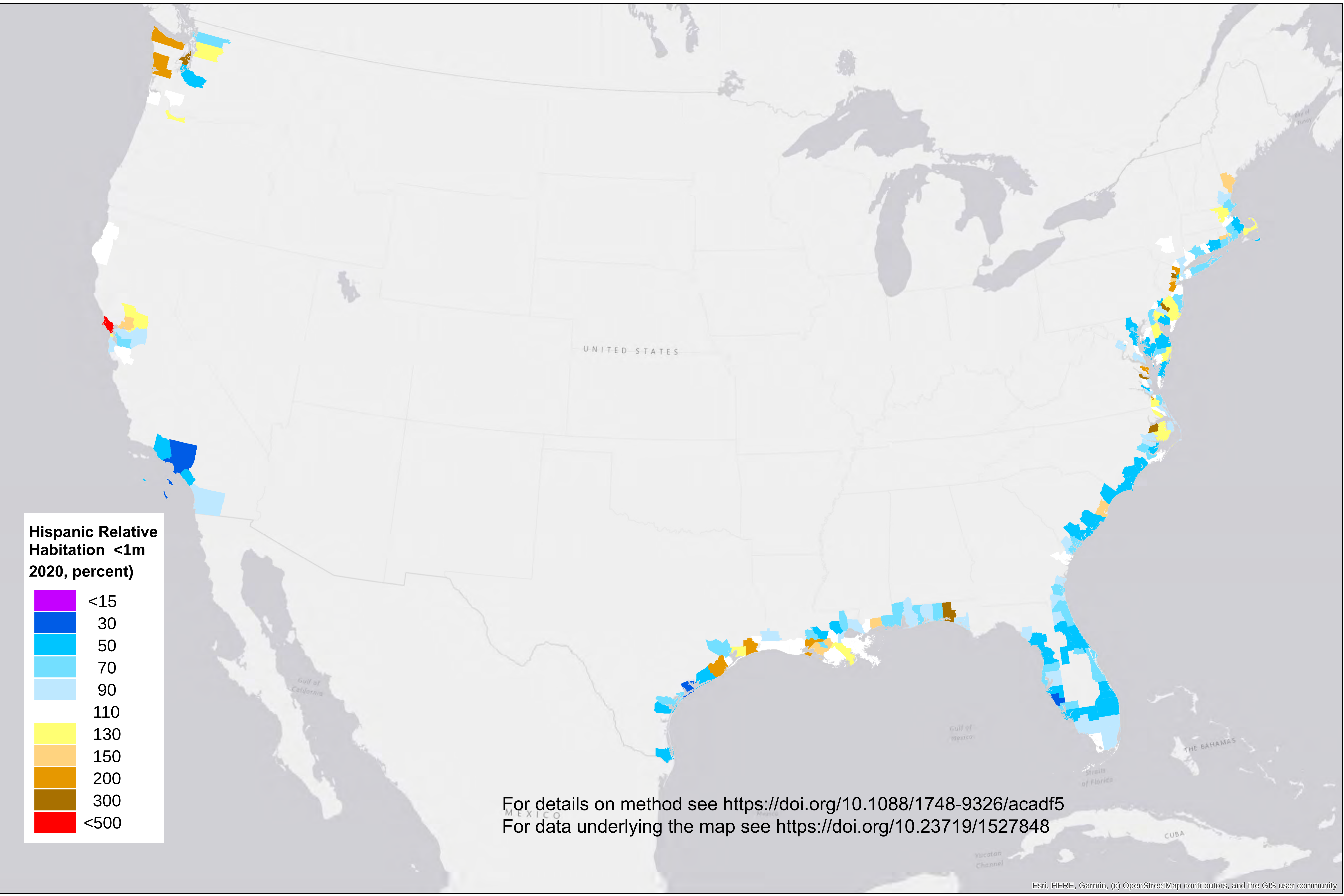
Hispanic Relative Habitation <1m 2020, percent)
<15
30
50
70
90
110
130
150
200
300
<500
UNITED STATES
MEXICO
THE BAHAMAS
CUBA
For details on method see https://doi.org/10.1088/1748-9326/acadf5
For data underlying the map see https://doi.org/10.23719/1527848
Esri, HERE, Garmin, (c) OpenStreetMap contributors, and the GIS user community

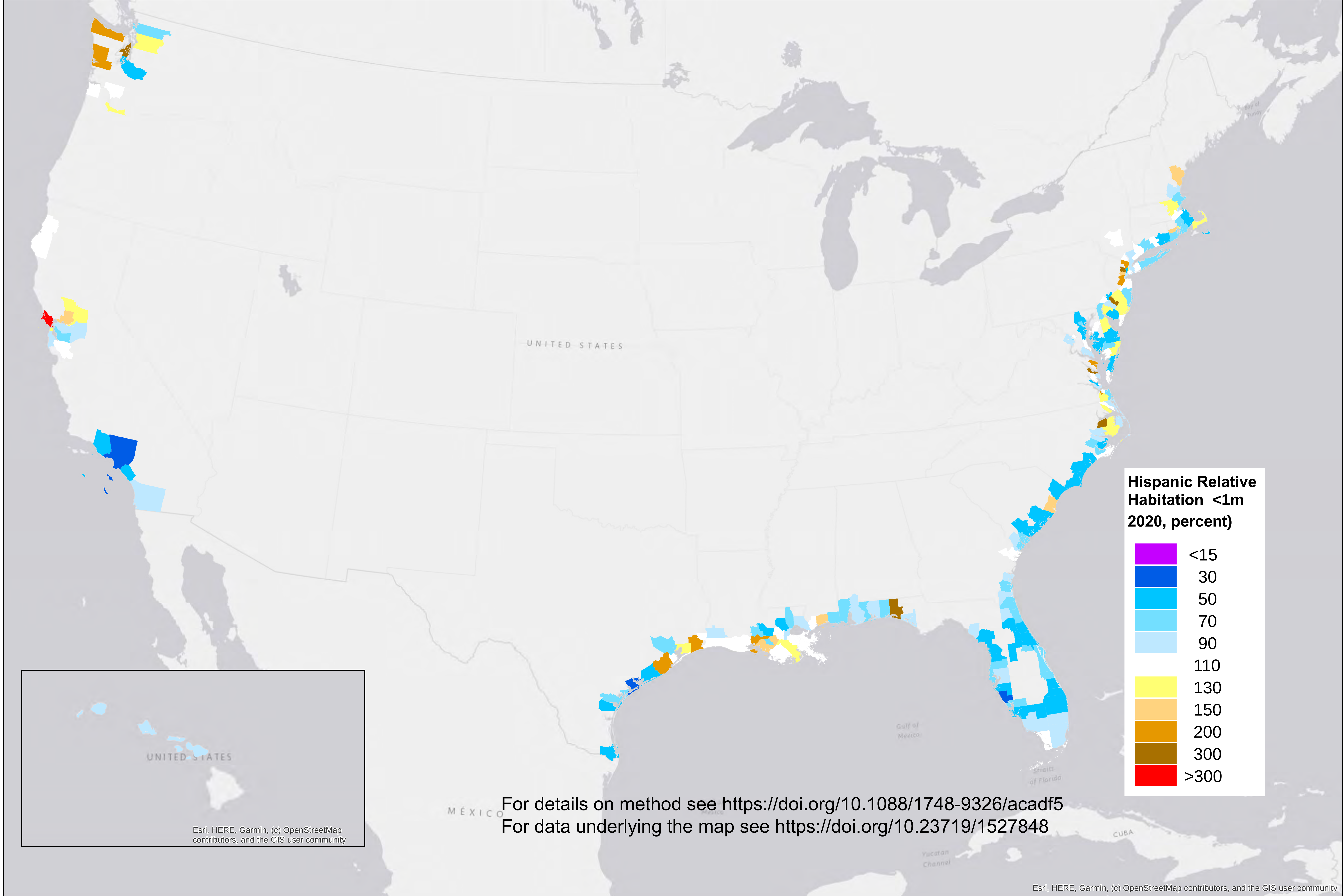
UNITED STATES
MÉXICO
CUBA
Hispanic Relative Habitation <1m 2020, percent)
<15
30
50
70
90
110
130
150
200
300
>300
For details on method see https://doi.org/10.1088/1748-9326/acadf5
For data underlying the map see https://doi.org/10.23719/1527848
Esri, HERE, Garmin, (c) OpenStreetMap contributors, and the GIS user community
Esri, HERE, Garmin, (c) OpenStreetMap contributors, and the GIS user community

Magnitude of the Extent to Which Black Residents Inhabit Land Vulnerable to Sea Level Rise

The maps of county-specific "ratios of disproportionality" presented elsewhere show the extent that Black or Hispanic people reside in lands vulnerable to sea level rise in particular counties, but the nationwide significance is hard to discern because counties have different populations. The maps here, in effect, weight those ratios by each county's population below one meter. More precisely, these maps show the difference between the actual Black population below one meter, and a "counterfactual", i.e., what the Black population below one meter would be if the percentage of Black county residents below one meter was equal to the percentage of all residents below one meter. The county-scale ratio of disproportionality (discussed in the paper for which these maps are a supplement) is calculated by adding those counterfactuals across all counties and comparing that sum to the total Black population in this hazard zone. For further details see the Supplemental Methods section.

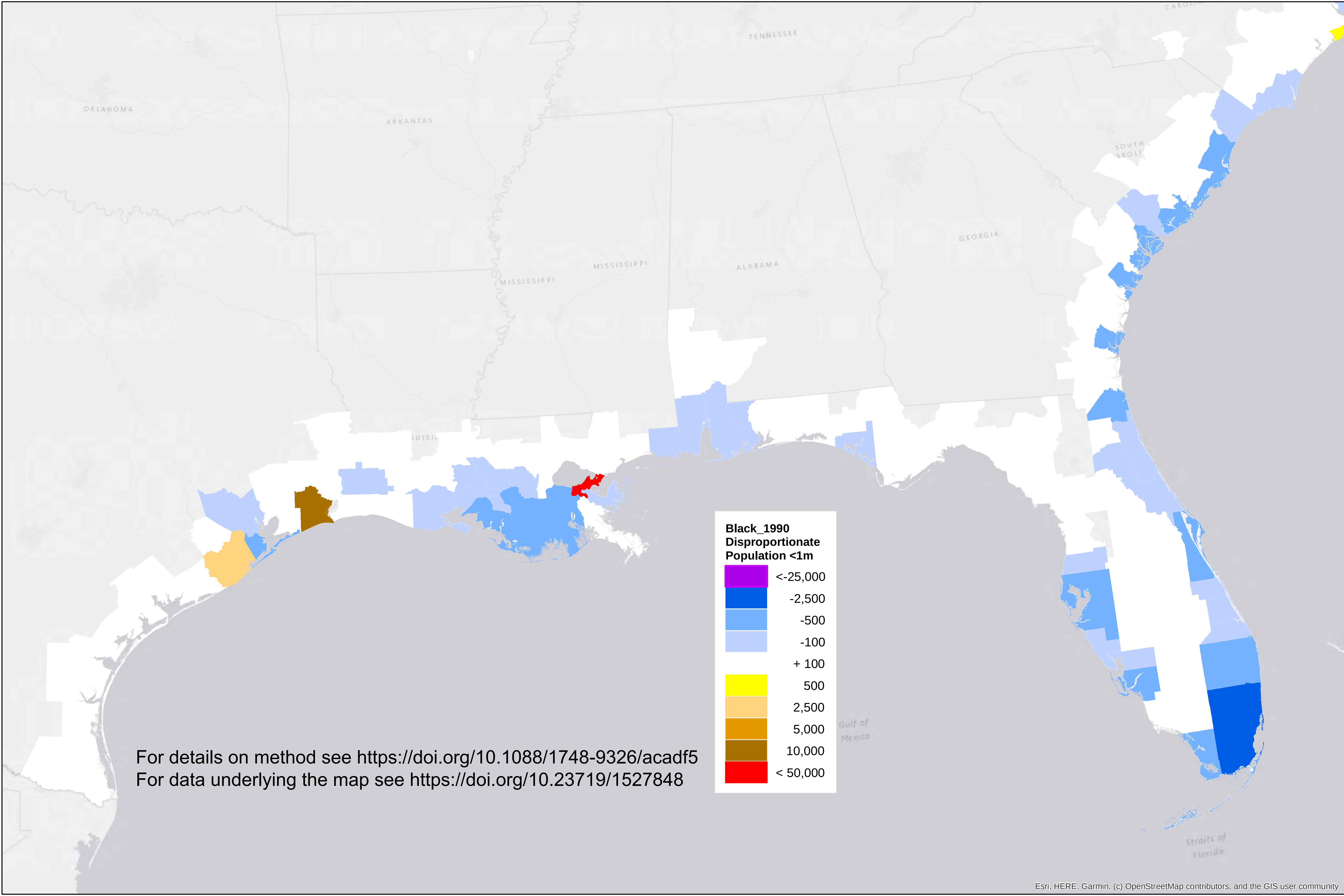
TENNESSEE
OKLAHOMA
ARKANSAS
SOUTH CAROLINA
GEORGIA
MISSISSIPPI
MISSISSIPPI
ALABAMA
Gulf of Mexico
Straits of Florida
Black_1990
Disproportionate
Population <1m
<-25,000
-2,500
-500
-100
+ 100
500
2,500
5,000
10,000
< 50,000
For details on method see https://doi.org/10.1088/1748-9326/acadf5
For data underlying the map see https://doi.org/10.23719/1527848
Esri, HERE, Garmin, (c) OpenStreetMap contributors, and the GIS user community

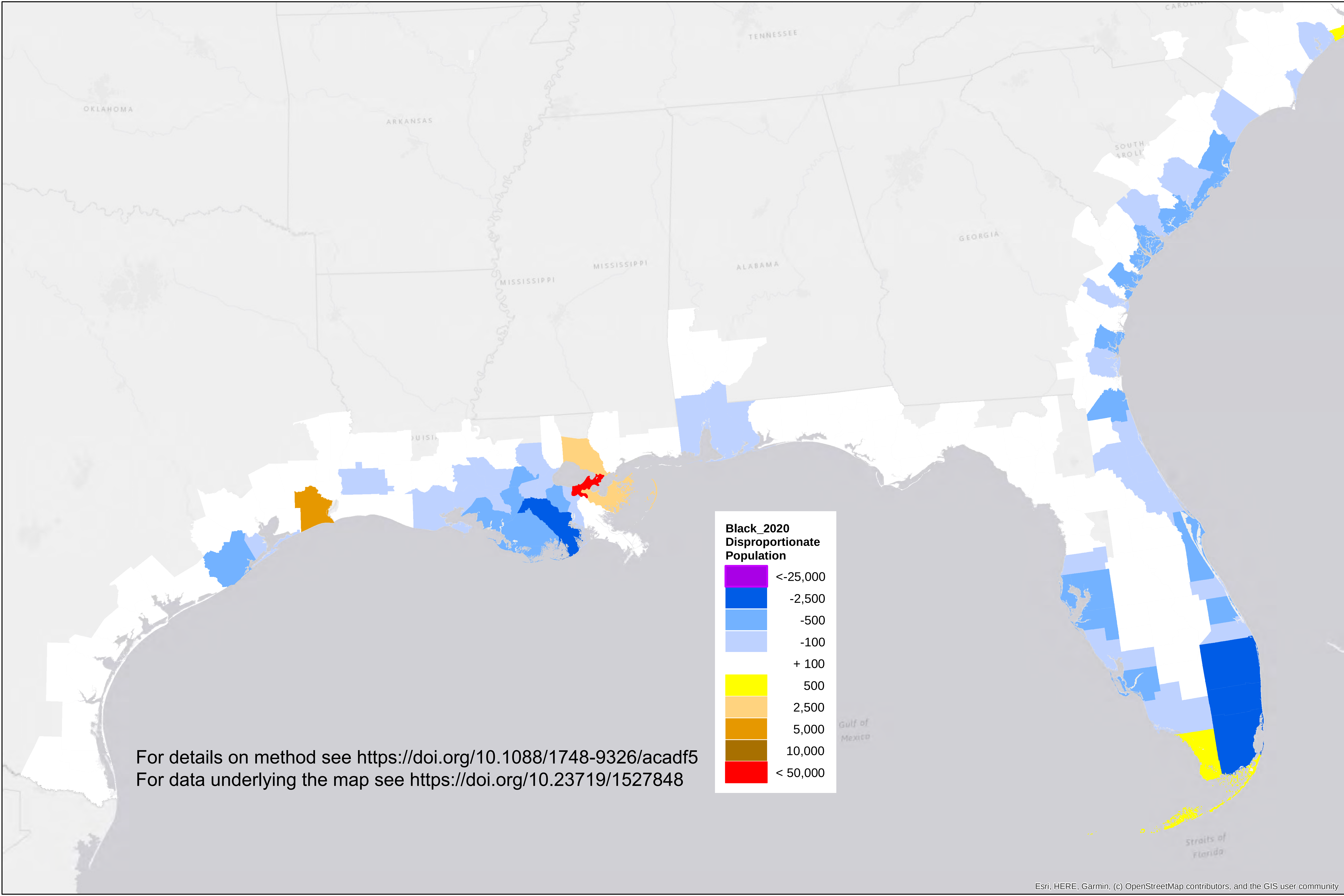
OKLAHOMA
ARKANSAS
TENNESSEE
MISSISSIPPI
MISSISSIPPI
ALABAMA
GEORGIA
SOUTH CAROLINA
Black_2020 Disproportionate Population
<-25,000
-2,500
-500
-100
+ 100
500
2,500
5,000
10,000
< 50,000
Gulf of Mexico
Straits of Florida
For details on method see https://doi.org/10.1088/1748-9326/acadf5
For data underlying the map see https://doi.org/10.23719/1527848
Esri, HERE, Garmin, (c) OpenStreetMap contributors, and the GIS user community

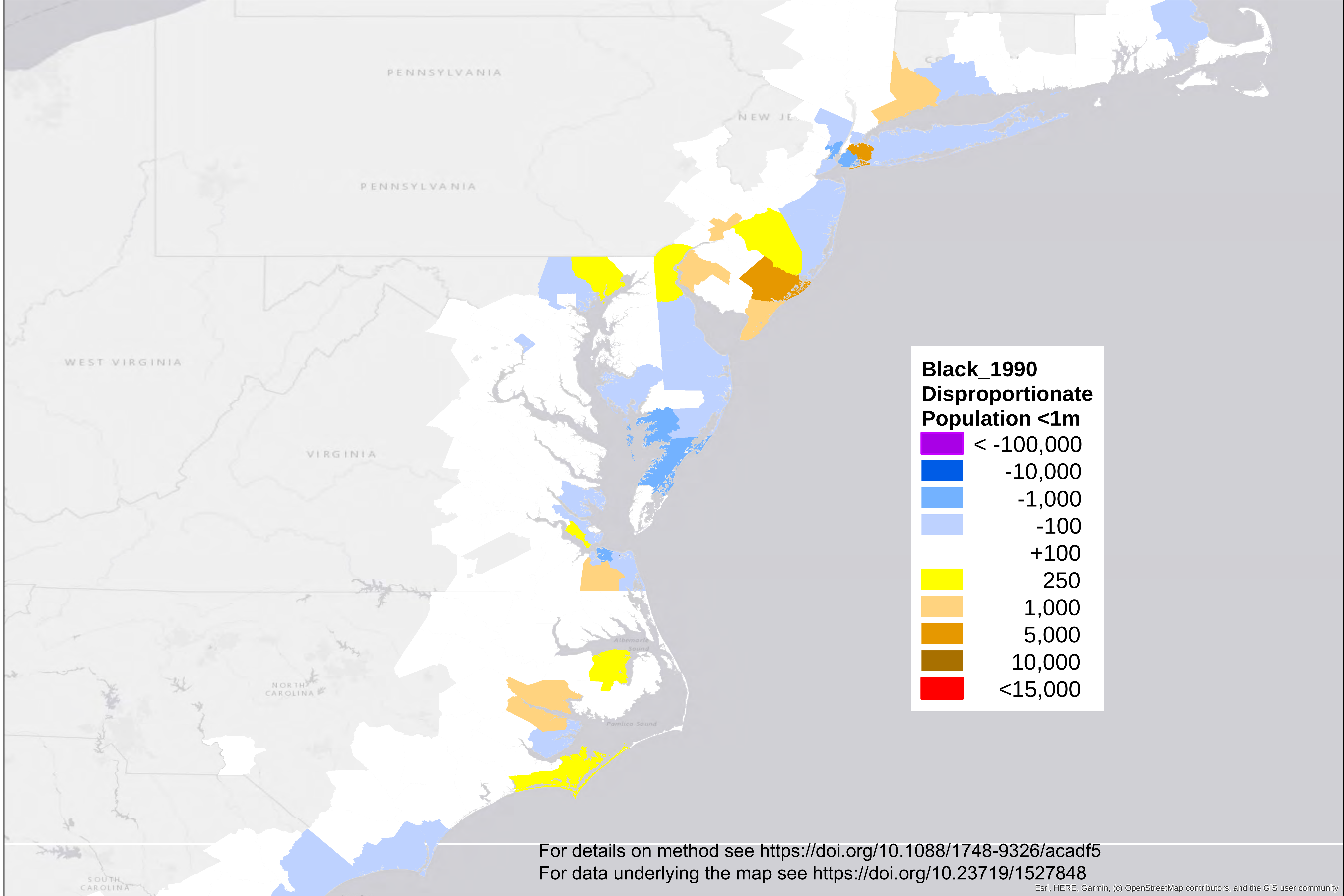

For details on method see https://doi.org/10.1088/1748-9326/acadf5
For data underlying the map see https://doi.org/10.23719/1527848

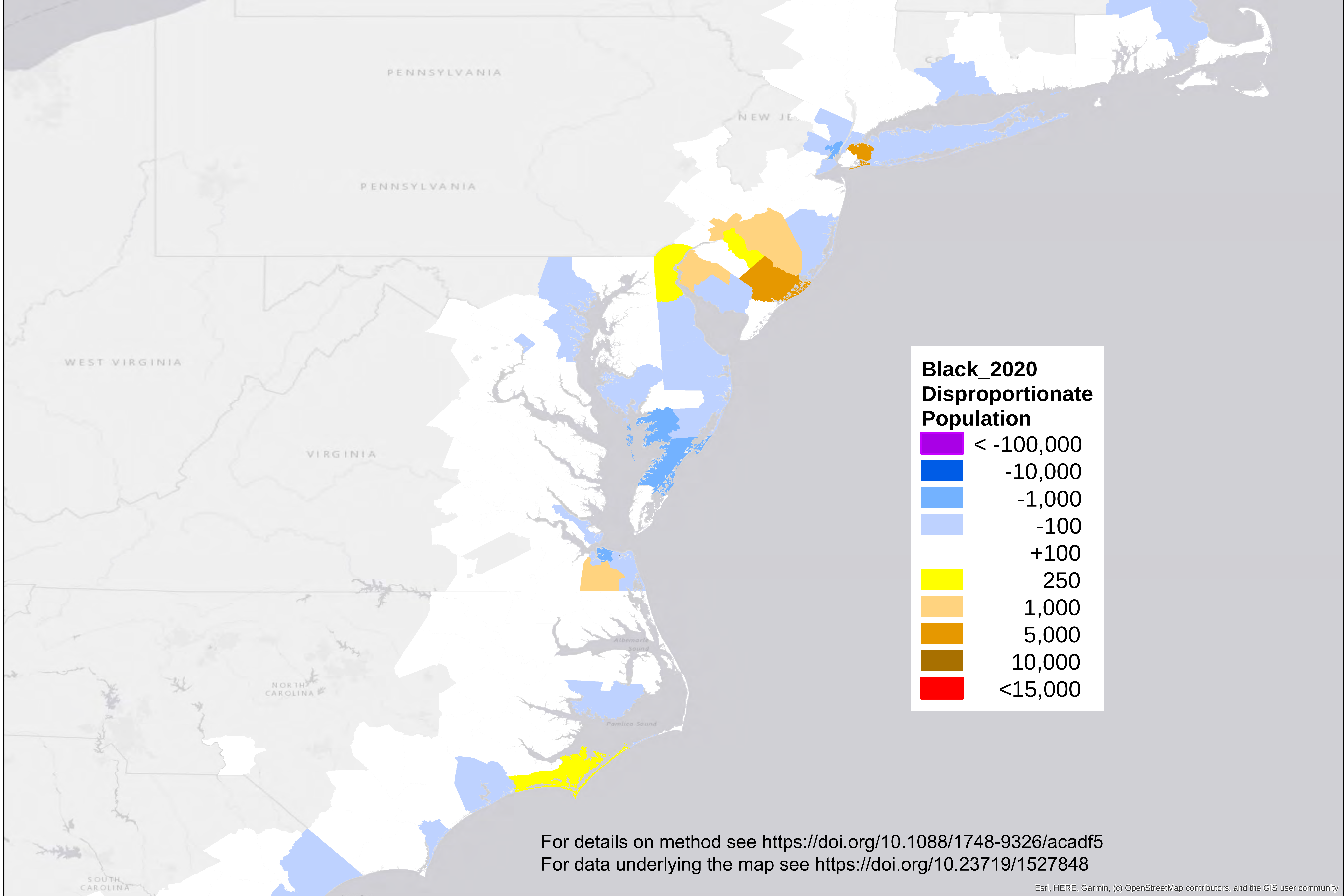

For details on method see https://doi.org/10.1088/1748-9326/acadf5
For data underlying the map see https://doi.org/10.23719/1527848

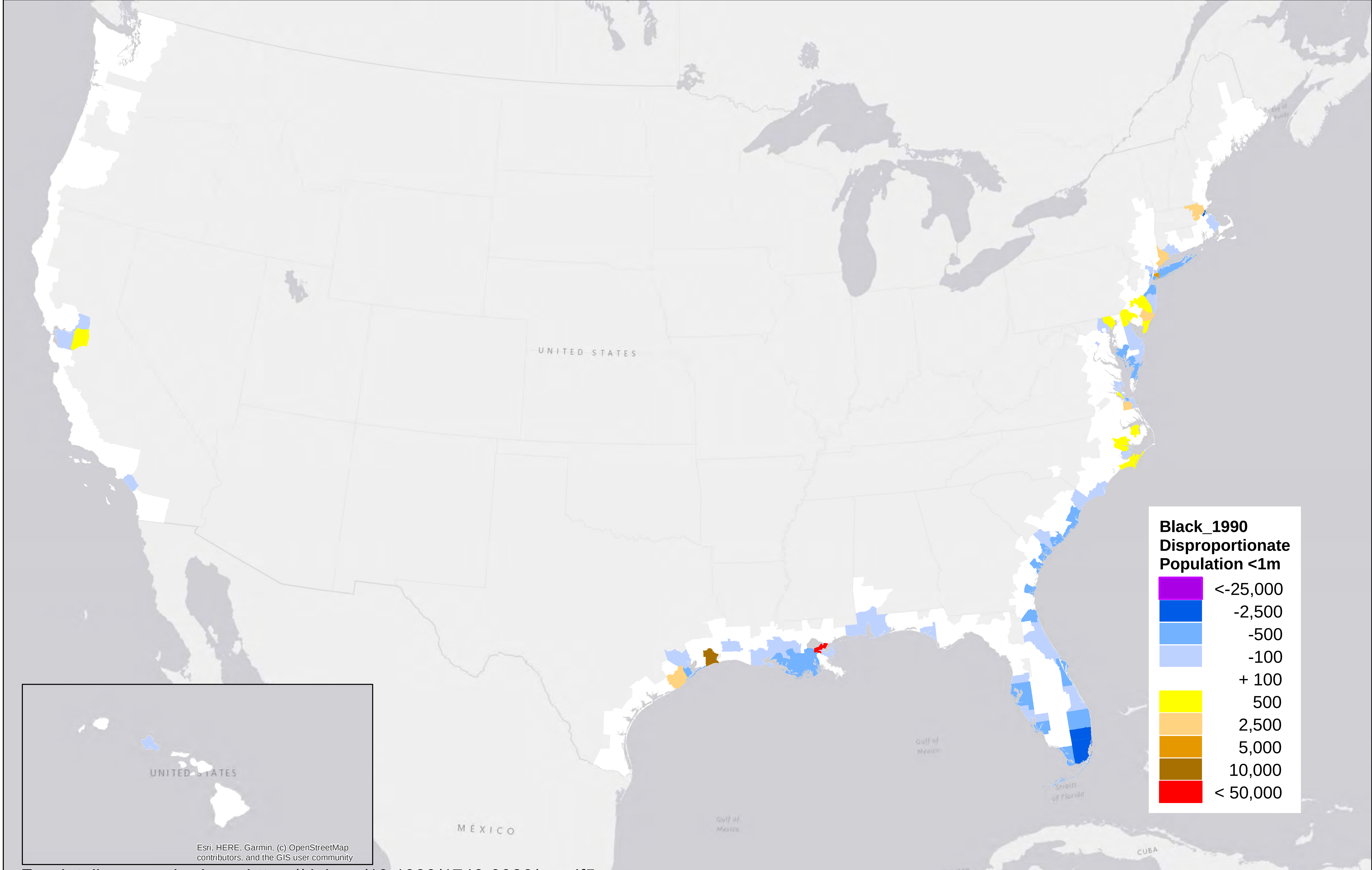

For details on method see https://doi.org/10.1088/1748-9326/acadf5
For data underlying the map see https://doi.org/10.23719/1527848

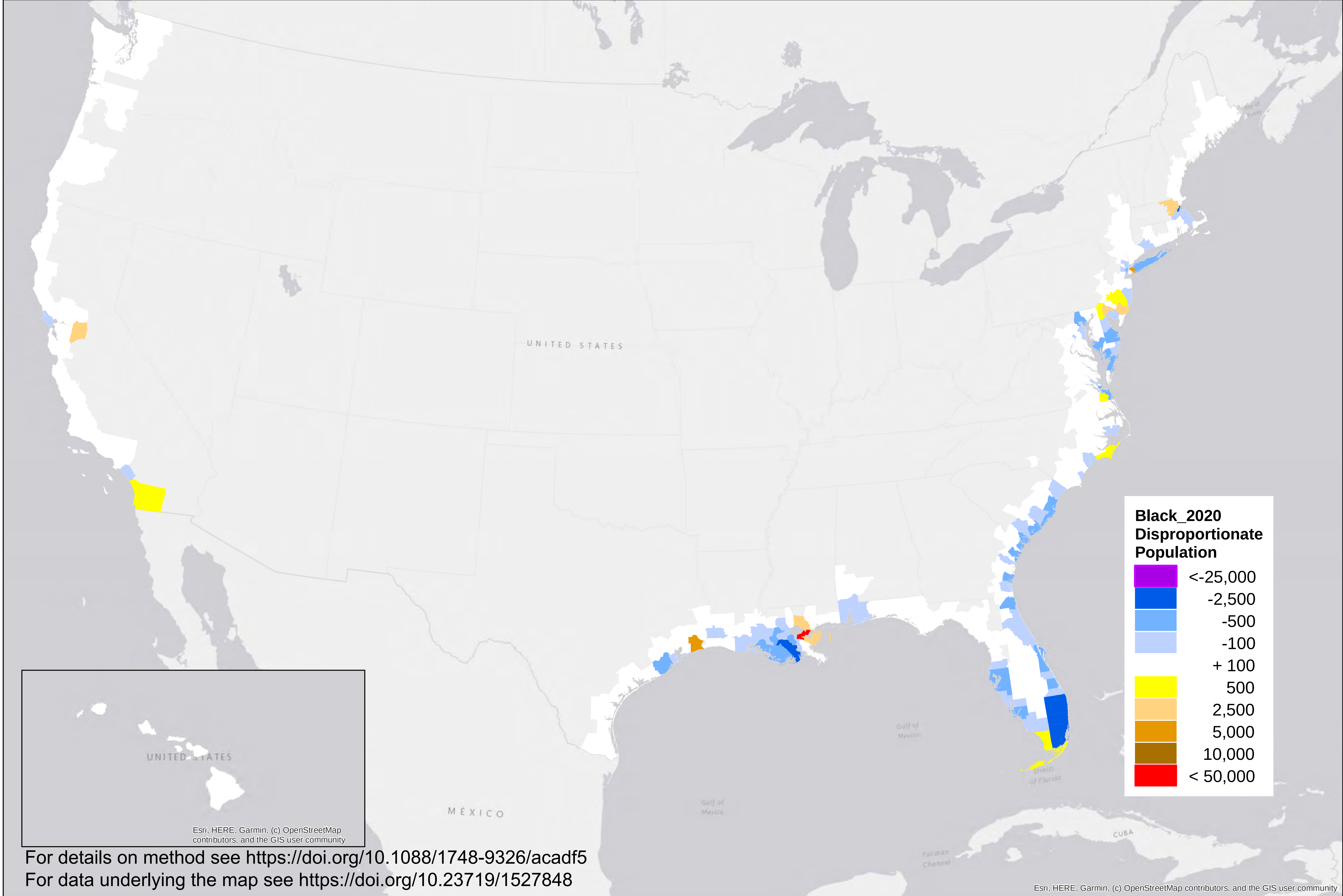

For details on method see https://doi.org/10.1088/1748-9326/acadf5
For data underlying the map see https://doi.org/10.23719/1527848

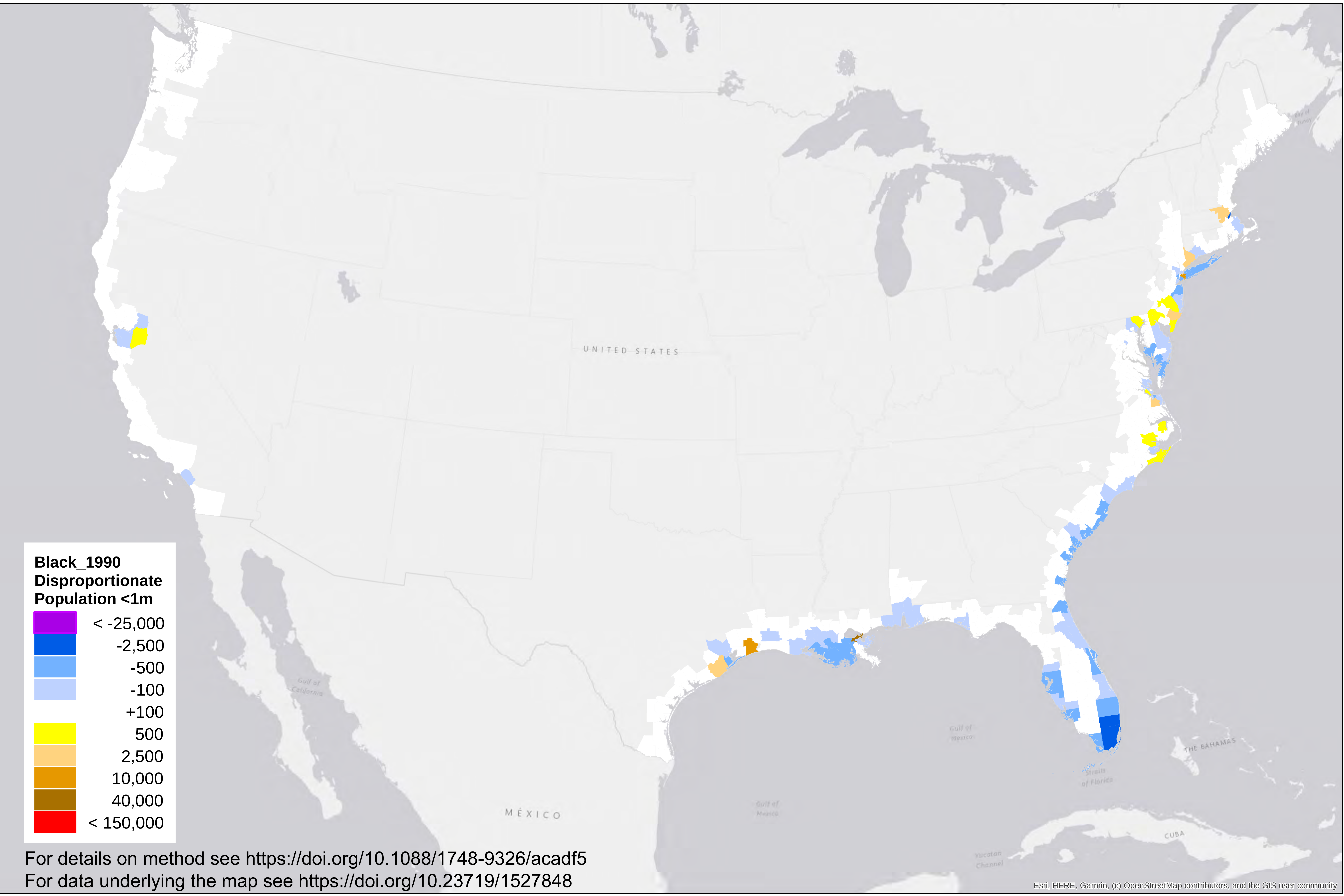

For details on method see https://doi.org/10.1088/1748-9326/acadf5
For data underlying the map see https://doi.org/10.23719/1527848

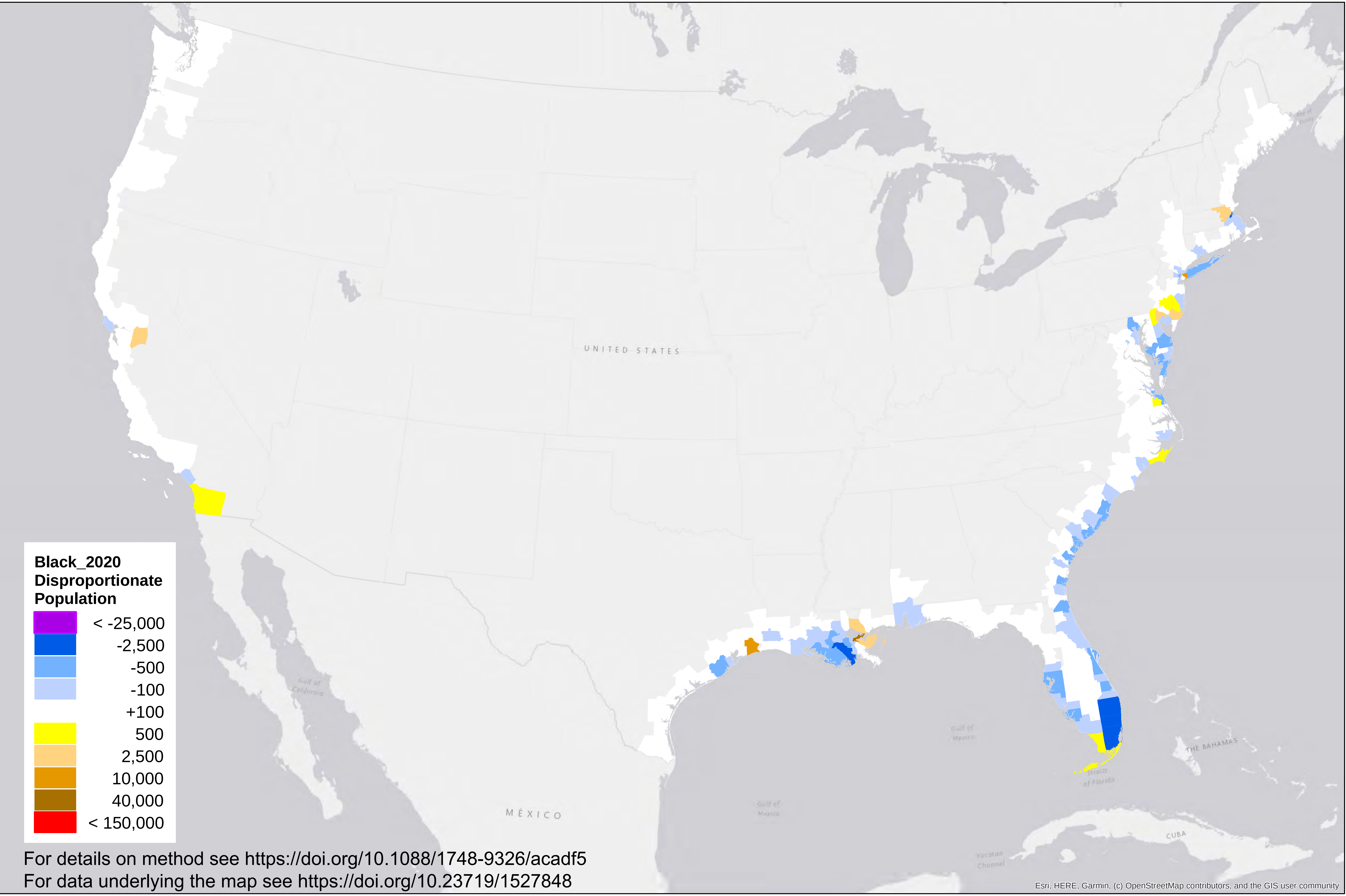

For details on method see https://doi.org/10.1088/1748-9326/acadf5
For data underlying the map see https://doi.org/10.23719/1527848

Bibliography

Federal Emergency Management Agency 2021 National flood hazard layer (available at: www.fema.gov/flood-maps/national-flood-hazard-layer) (Accessed 12 December 2021)

NOAA Sea level rise viewer DEM (available at: https://coast.noaa.gov/htdata/raster2/elevation/SLR_viewer_DEM_6230/)(Accessed 19 November 2021)

Manson S, Schroeder J, Van Riper D, Kugler T and Ruggles S 2021 IPUMS National Historical Geographic Information System: version 16.0 [dataset] (Minneapolis, MN) 10.18128/D050.V16.0

Titus, J.G., 2023a. Population in floodplains or close to sea level increased in US but declined in some counties—especially among Black residents. *Environmental Research Letters*, *18*(3), p.034001. https://doi.org/10.1088/1748-9326/acadf5

Titus, J.G., 2023b. Supplemental Methods from ‘Population in floodplains or close to sea level increased in US but declined in some counties–especially among Black residents. *Environmental Research Letters:* Supplementary Data
